# Apolipoprotein C‐II induces EMT to promote gastric cancer peritoneal metastasis via PI3K/AKT/mTOR pathway

**DOI:** 10.1002/ctm2.522

**Published:** 2021-08-09

**Authors:** Chao Wang, Zhi Yang, En Xu, Xiaofei Shen, Xingzhou Wang, Zijian Li, Heng Yu, Kai Chen, Qiongyuan Hu, Xuefeng Xia, Song Liu, Wenxian Guan

**Affiliations:** ^1^ Department of General Surgery, Nanjing Drum Tower Hospital The Affiliated Hospital of Nanjing University Medical School Nanjing China; ^2^ Department of General Surgery Nanjing Drum Tower Hospital Clinical College of Nanjing Medical University Nanjing China

**Keywords:** APOC2, CD36, EMT, gastric cancer, metabolism, peritoneal metastasis, PI3K/AKT/mTOR

## Abstract

**Background:**

Peritoneal metastasis (PM) occurs frequently in patients with gastric cancer (GC) and confers poor survival. Lipid metabolism acts as a non‐negligible regulator in epithelial–mesenchymal transition (EMT), which is crucial for the metastasis of GC. As apolipoprotein C2 (APOC2) is a key activator of lipoprotein lipase for triglyceride metabolism, the exact mechanism of APOC2 remains largely unknown in GC.

**Methods:**

Tandem mass tags identified differentially expressed proteins between human PM and GC tissues, and showed that APOC2 overexpressed in PM tissues, which was further confirmed by immunoblotting, immunohistochemistry, and ELISA. Global gene expression changes were identified in *APOC2* knockdown cells via RNA‐sequencing. The role of APOC2 in lipid metabolism of GC cells was assessed via the Seahorse XF analyzer and lipid staining assays. The biological role of APOC2 in GC cells was determined by 3D Spheroid invasion, apoptosis, colony formation, wound healing, transwell assay, and mouse models. The interaction between APOC2 and CD36 was analyzed by co‐immunoprecipitation and biolayer interferometry. The underlying mechanisms were investigated using western blot technique.

**Results:**

APOC2 overexpressed in GC PM tissues. Upregulation of APOC2 correlated with a poor prognosis in GC patients. APOC2 promoted GC cell invasion, migration, and proliferation via CD36‐mediated PI3K/AKT/mTOR signaling activation. Furthermore, APOC2‐CD36 axis upregulated EMT markers of GC cells via increasing the phosphorylation of PI3K, AKT, and mTOR. Knockdown either *APOC2* or *CD36* inhibited the malignant phenotype of cancer cells, and delayed GC PM progression in murine GC models.

**Conclusion:**

APOC2 cooperates with CD36 to induce EMT to promote GC PM via PI3K/AKT/mTOR pathway. APOC2‐CD36 axis may be a potential target for the treatment of aggressive GC.

## Graphical abstract

Tandem Mass Tags identified differentially expressed proteins (DEPs) between peritoneal metastasis (PM) and gastric cancer tissues, and showed that APOC2 was highly expressed in PM tissues.

APOC2 cooperates with CD36 to regulate EMT process via PI3K/AKT/mTOR signaling, which eventually promotes tumor progression and peritoneal metastasis in gastric cancer.

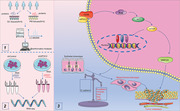

**ABBREVIATIONS**
PM: peritoneal metastasis
GC: gastric cancer
APOC2: apolipoprotein C‐II
IHC: immunohistochemistry
LC‐MS/MS: liquid chromatography‐tandem mass spectrometry
EMT: epithelial to mesenchymal transition
PI3K: phosphoinositide 3‐kinase
AKT: protein kinase B
mTOR: mammalian target of rapamycin
siRNA: small interfering RNA
TMT: tandem mass tag
DEP: differentially‐expressed proteins
co‐IP: co‐immunoprecipitation
BLI: biolayer interferometry
GSEA: gene set enrichment analysis
GSVA: gene set variation analysis

## 1 INTRODUCTION

As a major global health problem, especially in eastern Asia, gastric cancer (GC) ranks third in cancer‐related deaths globally and is associated with huge health burdens, largely because of the high incidence of recurrence and metastasis.^1^, ^2^ Peritoneal metastasis (PM) is a frequent pattern of metastasis in patients with advanced GC^3^ and represents a major challenge to both patients and clinicians.^4^, ^5^, ^6^ However, molecular mechanism underlying PM has not been fully revealed.

APOC2 is essential in the regulation of triglyceride‐rich lipproteins (TRLs),^7^ such as high‐density lipoproteins, very low‐density lipoproteins, and chylomicrons.^8^ The human *APOC2* gene is located in the *APOE‐APOC1‐APOC4‐APOC2* gene cluster and its transcriptional control is very complicated.^9^, ^10^ APOC2 is synthesized primarily by liver to bind lipids and lipoprotein lipase (LPL);^11^ however, it is also expressed in other tissues, including intestine, where it regulates local lipolysis.^12^, ^13^, ^14^ As a key regulator of the TRL metabolism, APOC2 can guide lipoproteins to active LPL sites and act as a physiological activator of LPL, which is the main enzyme of plasma triglycerides (TG) hydrolysis.^15^, ^16^, ^17^ Therefore, APOC2 can provide energy transport and storage for cells by regulating lipid metabolism.^11^ APOC2 deficiency can cause severe hypertriglyceridemia and lead to cardiovascular disease.^18^, ^19^ However, study toward the role of APOC2 in cancer is scarce.

CD36 is a multiligand class B scavenger receptor that functions in lipid metabolism and is involved in various cellular processes.^20^ In GC cells, fatty acids can upregulate CD36 expression to trigger the epithelial to mesenchymal transition (EMT) process, thereby promoting tumor growth and omental metastasis.^21^, ^22^, ^23^ It has been previously reported that APOC2 interacts with CD36 to trigger downstream signaling in acute myeloid leukemia (AML) and atherosclerosis.^11^, ^24^ However, it is unclear whether APOC2 interacts with CD36 to regulate EMT in GC.

EMT has been considered as a nonignorable factor for tumor invasion and metastasis.^25^, ^26^ In EMT, GC cells lose their original cell identity and acquire active mesenchymal phenotype, corresponding to the upregulation of Snail, Slug, Twist1, Vimentin, N‐cadherin, and matrix metalloproteinases (MMPs) and downregulation of E‐Cadherin.^27^, ^28^ Current literature suggests that PI3K‐AKT‐mTOR pathway is essential for EMT of malignant tumors.^29^ Activation of PI3K‐AKT‐mTOR pathway contributes to an aggressive cancer phenotype, including apoptosis resistance, invasion, and metastasis.^30^ CD36 has been reported to regulate PI3K/AKT signaling in tumor progression,^31^, ^32^ but the exact interaction between APOC2 and CD36 as well as subsequent effect on PI3K/AKT/mTOR signaling in GC have not been investigated.

In this article, we identified differentially expressed proteins (DEPs) possibly involved in GC PM using tandem mass spectrometry‐based proteomics and bioinformatic analyses. We revealed that APOC2 is a potential biomarker for PM in GCs. Furthermore, we compared transcriptome profiles between *APOC2* knockdown and untreated AGS cells to identify differentially regulated pathways after APOC2 interference. We revealed that APOC2 interference affected PI3K/AKT signaling in GC cells. Notably, we integrated proteome and transcriptome data with *in vitro* and *in vivo* assays, and discovered that APOC2 cooperated with CD36 to regulate EMT of GC cells and caused aberrant phosphorylation of PI3K, AKT and mTOR to promote PM progression in GC.

## 2 MATERIALS AND METHODS

### 2.1 Patient samples

Surgical specimens of five GC tissues and corresponding five PM tissues used for mass spectrometry detection were obtained from Nanjing Drum Tower Hospital (Nanjing, China). For immunohistochemical (IHC) analysis of APOC2 protein expression, the study enrolled 111 patients with GC from January 2014 to December 2014 at Nanjing Drum Tower Hospital. For ELISA analysis of serum APOC2 protein level in GC patient, the study enrolled 66 patients who were diagnosed with primary GC from February 2021 to April 2021 at Nanjing Drum Tower Hospital. All patients provided written informed consent. This study was performed in full accordance with the principles of the Declaration of Helsinki. The Ethics Committee of Nanjing Drum Tower Hospital approved this study.

### 2.2 Cell culture

MGC‐803, MKN‐45, AGS, BGC‐823, SNU‐16, and GES‐1 were purchased from Shanghai Cafa Biological Technology Co., Ltd. (Shanghai, China). Cells were maintained in Dulbecco's modified Eagle's medium (DMEM, Gibco, Waltham, MA, USA) with 10% fetal bovine serum (Gibco, Waltham, MA, USA).

### 2.3 Tandem mass tag quantification proteomic analysis

The samples were prepared and DEPs were detected via LC‐MS/MS‐based proteomic and bioinformatic analyses at the Beijing Genomics Institution. For details, see Supplementary Materials and Methods.

### 2.4 siRNA assay

Cells were treated with APOC2‐siRNAs (siRNA#1, 5′‐UCCUCCUGGUAUUGGGAUUTT‐3′, 5′‐AAUCCCAAUACCAGGAGGATT‐3′; siRNA#2, 5′‐CCCAGAACCUGUACGAGAATT‐3′, 5′‐UUCUCGUACAGGUUCUGGGTT‐3′; siRNA#3, 5′‐GCCAUGAGCACUUACACAGTT‐3′, 5′‐CUGUGUAAGUGCUCAUGGCTT‐3′) or control‐siRNA. These siRNAs were provided by Shanghai GenePharma Company (Shanghai, China). 2 × 10^5^ GC cells per well plated into a six‐well plate were treated with siRNA (1‐2 µg) encapsulated by the interferin reagent (Polyplus, New York, NY, USA) based on the protocol. Western blot was used to evaluate the knockdown efficiency.

### 2.5 Plasmid construction and cell transfection

Control‐shRNA and CD36‐shRNA plasmids were obtained from Shanghai Genechem Co., Ltd. The shRNA sequences for CD36 were as follows: (shRNA#1, gcCATAATCGACACATATAAA; shRNA#2 TTGCCATAATCGACACATATA; shRNA#3 ccATTGGTGATGAGAAGGCAA). Lipofectamine 2000 (Invitrogen) was used for the plasmid transfections. Western blotting was used to evaluate the shRNAs knockdown efficiency after transfection for 48 h.

### 2.6 Lentivirus transductions

Short hairpin *APOC2* (shAPOC2) lentiviral particles and the corresponding shControl lentiviral particles were purchased from Shanghai Genechem Co., Ltd. Short hairpin *CD36* (shCD36) lentiviral particles and the corresponding shControl lentiviral particles were purchased from OBiO Technology Corp., Ltd. (Shanghai, China). To overexpress APOC2 and CD36, lentiviral particles containing human full‐length *APOC2* and *CD36* were prepared by OBiO Technology Corp., Ltd. and used to infect GC cells. Cells were transinfected with the lentiviral particles and were selected using Blasticidin S (30 µg/ml) (Thermo Fisher Scientific, Waltham, MA, USA) or puromycin (1 mg/ml) (Thermo Fisher Scientific) for 3 weeks.

### 2.7 Western blot

Proteins were extracted from cell samples and tissues using Radioimmunoprecipitation assay lysis buffer as previously described.^33^ Antibody information is as follows: APOC2 (Abcam, ab76452), GAPDH (Abcam, ab9485), Phospho‐PI3 Kinase (Cell Signaling Technology, #17366S), Phospho‐AKT (Cell Signaling Technology, #4060S), AKT (Cell Signaling Technology, #4685S), Phospho‐mTOR (Cell Signaling Technology, # 5536S), mTOR (Cell Signaling Technology, #2983S), E‐Cadherin (Cell Signaling Technology, #3195S), N‐Cadherin (Cell Signaling Technology, #13116S), Vimentin (Cell Signaling Technology, #5741S), Snail (Cell Signaling Technology, #S3879), Slug (Cell Signaling Technology, #9585S), TWIST1 (Cell Signaling Technology, #69366), MMP2 (ProteinTech Group, #10373‐2‐AP), MMP9 (ProteinTech Group, #10375‐2‐AP), CD36 (Affinity Biosciences, DF13262), FLAG M2 (Sigma, #F1804), and His TAG (Cell Signaling Technology, #12698S).

### 2.8 IHC staining and scoring

The detailed steps were conducted as described previously.^33^ The indicated sections were separately incubated with APOC2 antibody (Abcam, ab76452). Both the staining intensity and the proportion of positively stained tumor cells were used to evaluate the total score of each sample. Based on the intensity of staining, the specimens were divided into four groups: 3 (brownish‐yellow), 2 (yellow brown), 1 (light staining) and 0 (no staining), while the proportion of tumor cells was graded as follows: 3 (> 50%), 2 (10–50%),1 (< 10%), and 0 (no positively stained cells). The final expression was calculated as the proportion grade multiplied by the staining intensity score. A sample score of 0–2 was defined it as low expression, and 3–9 represented it as high expression.

### 2.9 ELISA

Procedures were as described previously.^34^ An ELISA (MEIMIAN, China) kit was used to measure the level of serum APOC2 in the GC patient.

### 2.10 Co‐immunoprecipitation

The detailed steps were performed as previously described.^35^ Briefly, cells were lysed in co‐immunoprecipitation (co‐IP) buffer (pH 7.5, 1% Triton X‐100, 150 mM NaCl, protease inhibitor cocktail, 20 mM Tris, and 1 mM EDTA) on ice for 35 min. Cell supernatant was collected and then incubated with GammaBind Plus Sepharose (GE Healthcare, Chicago, IL, USA) and primary antibodies. Twelve hours later, cold co‐IP buffer was used to wash the complex six times and then using western blot to assess the complex.

### 2.11 Biolayer interferometry assay

The Octet RED96E instrument (ForteBio, Pall Life Sciences, Port Washington, NY, USA) was used to conduct biolayer interferometry (BLI) assay. In brief, Recombinant CD36 (Sino Biological, #10752‐H08H) diluted to 5 µg/ml with PBS was immobilized on the Aminopropylsilane biosensor. Various concentrations of APOC2 (MedChemExpress, #HY‐P7529) diluted with SD buffer (PBS, 0.1% BSA, and 0.02% Tween 20) were applied in the mobile phase. ForteBio Data Analysis 10.0 software was used to analyze the data. The dissociation rate constant (*K*_dis_), dissociation equilibrium constant (*K_D_*), and binding rate constant (*K*_on_) were obtained by curve fitting of the association and dissociation phases of sensorgrams using 1:1 ligand model.

### 2.12 RT‐qPCR

Procedures were as described previously.^33^ Total RNA was extracted using Trizol reagent (Invitrogen,Waltham, MA, USA). Gene expression was normalized against human GAPDH and was presented by the 2^–ΔΔCt^ method. The primers were as followed: APOC2 (forward: TGTCCTCCTGGTATTGGGATTT; reverse: TGTCTTCTCGTACAGGTTCTGG), GAPDH (forward: GGAGTCCACTGGCGTCTTCA; reverse: GTCATGAGTCCTTCCACGATACC).

### 2.13 Seahorse and cellular metabolic analysis

For cell energy phenotype analysis, Seahorse XF Real‐Time ATP Rate Assay Kit (Agilent, Santa Clara, CA, USA) was performed to evaluate the oxygen consumption rate (OCR) and extracellular acidification rate (ECAR). Briefly, 2000 *APOC2* knockdown cells and untreated cells were seeded into each well of Poly‐D‐Lysine (10 µg/ml, Sigma, #P6407) coated 96‐well Agilent Seahorse XF Cell Culture Microplate, separately. Oligomycin and Rotenone + antimycin A were dissolved in assay medium and loaded into the sensor cartridge at final concentrations of 1.5 and 0.5 µM, respectively. For the ECAR analysis, Seahorse XF Glycolysis Stress Test Kit (Agilent) was used to assess ECAR under various conditions. Briefly, *APOC2* knockdown cells (2 × 10^4^) and untreated cells (2 × 10^4^) were planted into 24‐well plate, followed by the measurement of baseline. At the indicated time points, the glucose (10 mM), oligomycin (1 µM), and 2‐deoxy‐D‐glucose (2‐DG; 100 mM) were added into per well, respectively.

### 2.14 RNA sequencing analysis

The mRNA of the AGS cells was extracted and sent to Shanghai Genechem Co., Ltd. for mRNA sequencing. Differential genes expression analysis was completed using the R package limma (version 3.44.3). The fold change (FC) of each gene was log2 transformed and further analyzed using the R package clusterProfiler (version 3.16.1). Then, gene set enrichment analysis of canonical pathways was conducted to find differentially regulated pathways.

### 2.15 Apoptosis assay

The Apoptosis Detection Kit (BioLegend, #640914) was performed to evaluate the cell apoptosis. Cells (100 µl) were incubated with 400 µl Annexin V binding buffer, including 10 µl of PI solution and 5 µl FITC Annexin V, for 15 min. A flow cytometer was conducted to analyze cells apoptosis.

### 2.16 Wound healing assay

Cells were maintained in a 6‐well plate until they reached confluence and wounded with a sterile pipette tip. After 48 h, the cell migration distance was determined via a microscope. Cell migration was calculated as the percentage of wound closure.

### 2.17 Cell migration assay

Cells (5 × 10^4^) were seeded into the upper chamber (Costar, Cambridge, MA, USA) with 200 µl serum‐free DMEM, DMEM containing 10% fetal bovine serum was added into the lower chamber (Costar, Cambridge, MA, USA). After 24 h, cells adhering to the lower surface were fixed with methanol and stained with crystal violet and observed under microscope.

### 2.18 Colony formation assay

Four hundred treated GC cells were plated into a 6‐well plate and incubated for 14 days. Then, cells were fixed with methanol and stained with 0.1% crystal violet. Cell colonies containing more than 50 cells were counted.

### 2.19 Cell invasion assay

The Transwell chamber (Costar, Cambridge, MA, USA) was precoated with Matrigel. Cells (1 × 10^5^) were then seeded into the upper chamber (Costar, Cambridge, MA, USA) with 200 µl serum‐free DMEM, DMEM containing 10% fetal bovine serum was added into the lower chamber (Costar, Cambridge, MA, USA). After incubation at 37°C for 24 h, cells adhering to the lower surface were fixed with methanol and stained with crystal violet and observed under microscope.

### 2.20 Lipid staining assay

Cells were incubated with Oil Red O solution (Nanjing Jiancheng Institute of Bioengineering, China) for 15 min at RT and observed under a light microscope. For confocal analysis, cells were incubated BODIPY 493/503 (Thermo Fisher Scientific, #D3922) and DAPI (BioFroxx, #1155MG010) for 30 and 5 min at RT, respectively, and observed under fluorescence microscope.

### 2.21 Spheroid cell invasion assay

A 3D sphere BME cell invasion test (Trevigen, Gaithersburg, MD, USA) was used to evaluate the cell invasion. The detailed experimental method was as described in the previous publication.^36^ Cells were stained with DiL (Beyotime, China). Images were analyzed using Image J software.

### 2.22 In‐vivo study

Male BALB/c nude mice, 3–4 weeks, were obtained from the Model Animal Research Center of Nanjing University (Nanjing, China). Animals were kept in specific pathogen‐free environment. Peritoneal dissemination was assessed via intraperitoneal injection. Briefly, 3 × 10^6^ AGS‐shAPOC2, AGS‐OE APOC2, AGS‐OE CD36/shAPOC2, AGS‐OE APOC2/shCD36, and the corresponding control cells in 400 µl PBS were injected into the nude mice peritoneal cavity. After 6 days, PI3K/Akt signaling pathway agonist 740Y‐P (20 mg/kg) or PI3K/AKT signaling pathway inhibitor LY294002 (50 mg/kg) was injected intraperitoneally every 3 days for 9 days. PM mice (6 mice/group) were analyzed when the mice were euthanized at 15 days post injection.

For subcutaneous tumor growth assay, 5 × 10^6^ AGS‐shCDAPOC2, AGS‐OE APOC2, AGS‐OE CD36/shAPOC2, AGS‐OE APOC2/shCD36, and the corresponding control cells in 300 µl of PBS were injected into nude mouse. Then, caliper was used to measure tumors volume every 5 days via the following formula: tumor volume = (length × width^2^)/2. After 7 days, 740Y‐P (20 mg/kg) or LY294002 (50 mg/kg) was intraperitoneally injected into nude mice every 3 days for 18 days. The mice were euthanized on day 25, and the tumor volume and weight were analyzed. All animal procedures were approved by the Ethics Committee of Nanjing Drum Tower Hospital.

### 2.23 Statistical analysis

SPSS 19.0 software (IBM Corp.) was used for all statistical analyses. Continuous data were presented as means ± standard deviation (SD), and the differences among the experimental groups were analyzed using one‐way ANOVA or Student's *t*‐test. Frequencies of categorical variables were compared using the Pearson's χ^2^ test. Survival curve was generated using the Kaplan–Meier method and compared by log‐rank test. *p* < 0.05 was considered statistically significant.

## 3 RESULTS

### 3.1 Quantitative proteomic results demonstrate significant differences between GC and PM

**FIGURE 1:**
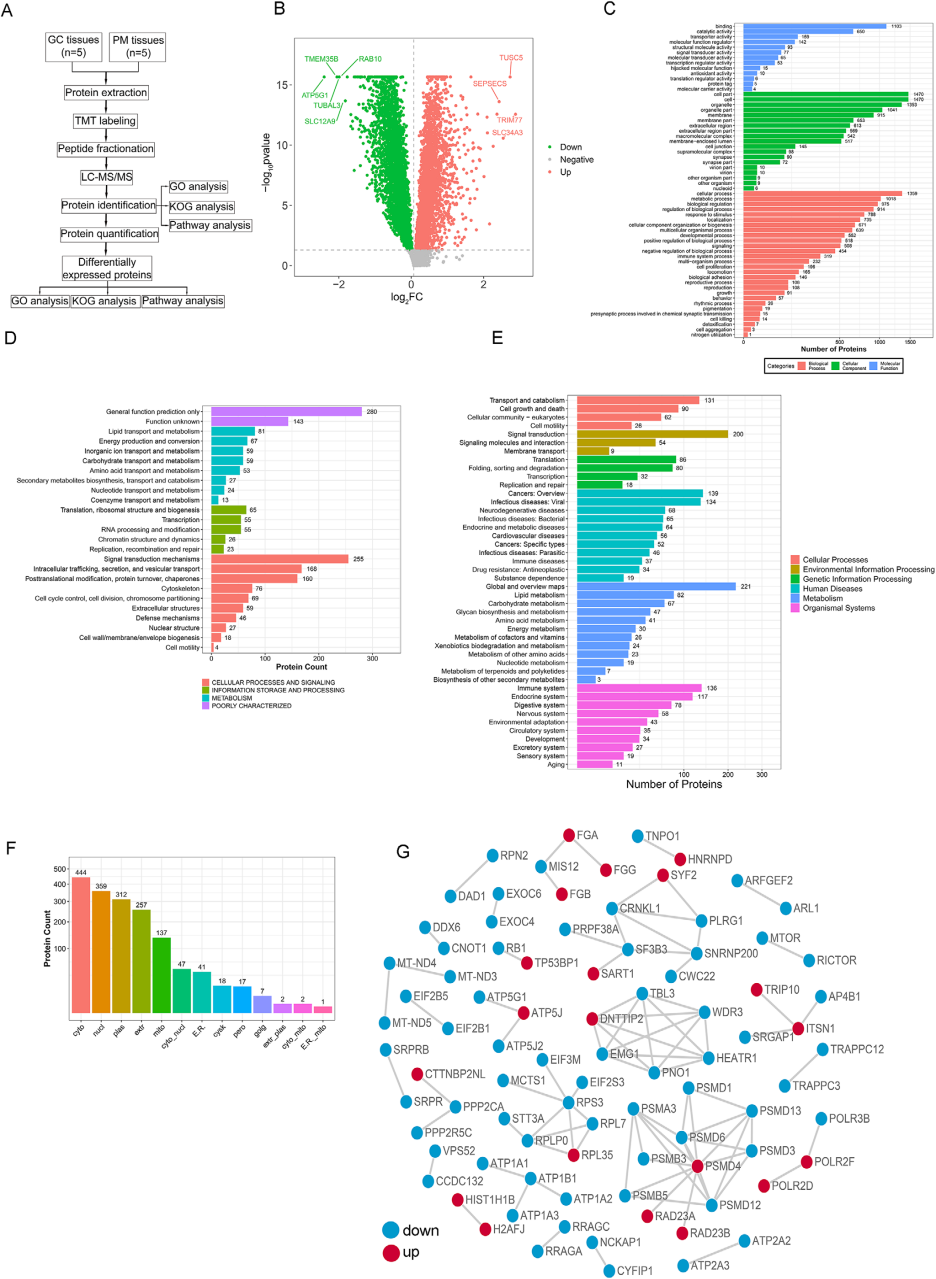
Identification of DEPs between GC tissue and PM tissue based on TMT‐labeled quantitative proteomics analysis. (A) Schematic diagram of the workflow for the discovery and verification of cancer biomarkers for patients with GC PM. (B) Volcano of differentially expressed proteins. (C) GO enrichment analysis of all the DEPS. (D) The potential functions of DEPs annotated by KOG. (E) KEGG enrichment analysis to characterize the biological functions of the DEPs. (F) The subcellular localization of the identified DEPs. (G) STRING database (STRING 11.0) was used to analyze the DEPs to conduct network interaction analysis of protein–protein relationships. Red and blue nodes indicate upregulated and downregulated proteins, respectively

GC and PM types have not been compared systematically at the tissue proteomic level, and the differentiation between PM and primary GC is of fundamental clinical importance for therapeutic stratification; we explored the possibility of identifying biomarkers for proteomic diagnosis. The experimental process of the strategy used to identify the DEPs in PM tissues is shown briefly in Figure 1A. To find meaningful protein alterations in GC and PM tissues, we extracted GC tissue and PM tissue from five patients through surgery for proteome profiling. A total of 7638 proteins were identified at 1% false discovery rate (Table S1). For the experimental design with more than one replicate, the DEPs were defined with a 1.5‐FC and a *p*‐value less than 0.05. Compared with the primary GC, 595 proteins in the PM tissues were upregulated among these DEPs (Table S2), while 1050 proteins were downregulated (Table S3). Figure 1B briefly summarizes the identification results for all 7638 proteins.

For GO enrichment analysis, Table S4 and Figure S1A provid in detail all identified protein‐specific information and visualization results, respectively. GO enrichment analysis indicated that the most abundant biological processes mainly included cellular processes and metabolic processes. As to molecular function, the most enriched categories included “binding” and “catalytic activity.” Then, we performed GO enrichment analysis for the DEPs (Table S5). All DEPs are illustrated through our GO function classification map (Figure 1C). GO function classification map also distinguished between upregulated and downregulated proteins (Figure S2A). We found that both upregulated and downregulated DEPs were involved in common structural or functional processes. Some GO biological processes and molecular functions were specifically enriched for upregulated proteins (such as cell aggregation and protein tag) or downregulated proteins (such as nitrogen utilization and hijacked molecular function) (Figure S2A). Next, we used the database of protein orthologs classification (EuKaryotic orthologous group (KOG)) to predict the potential functions of all identified proteins (Table S6 and Figure S1B). And among the identified DEPs (Table S7), the highly enriched KOG category was “cellular process and signal transduction," which suggested that DEPs were mainly related to signal transduction mechanisms and post‐translational modification (Figure 1D). Next, KEGG enrichment analysis was performed on all identified proteins (Table S8 and Figure S1C) and characterized the biological functions involving these DEPs(Table S9). The analysis demonstrated that the upregulated proteins were related to 25 main pathways, of which “metabolic pathway” was the most enriched category (Figure S2B). Figure 1E and Table S9 demonstrat that DEPs in PM tissues were mainly enriched in metabolic processes, especially lipid metabolism. Among them, “cholesterol metabolism” was enriched in APOC2, LIPL, SORC2, IBP6, CD36, and APOA. To understand the biological function of these genes, the R package GSVA (version 1.36.2) was used to score all the gene ontology biological process for 375 GC samples in TCGA‐STAD cohort. The expression level of LIPL, SORC2, IBP6, CD36, and APOA was correlated with normalized score of each gene sets by the R package Hmisc (version 4.4.1), the top 20 most related gene sets were shown by the R package ggplot2 (version 3.3.0). Figure S3 shows detailed analysis of the functions of these genes in GC from TCGA STAD data set. Although these proteins (LIPL, SORC2, IBP6, CD36, and APOA) were highly expressed in GC PM tissues (Figure S4A and S4B) and the expression levels of these genes were negatively correlated with the prognosis of GC patients (Figure S4C‐G), only LIPL and CD36 could regulate EMT of GC (Figure S5A‐O).

We further predicted subcellular localizations of the DEPs via bioinformatic tools (WoLF PSORT) (Figure 1F). The analysis showed that intracellular, extracellular, and mitochondria were the most representative structures (Table S10). In addition, the STRING database (STRING 11.0) was used to analyze the DEPs to conduct network interaction analysis of protein‐protein relationships (Figure 1G).

### 3.2 APOC2 is upregulated in patients with GC PM

**FIGURE 2:**
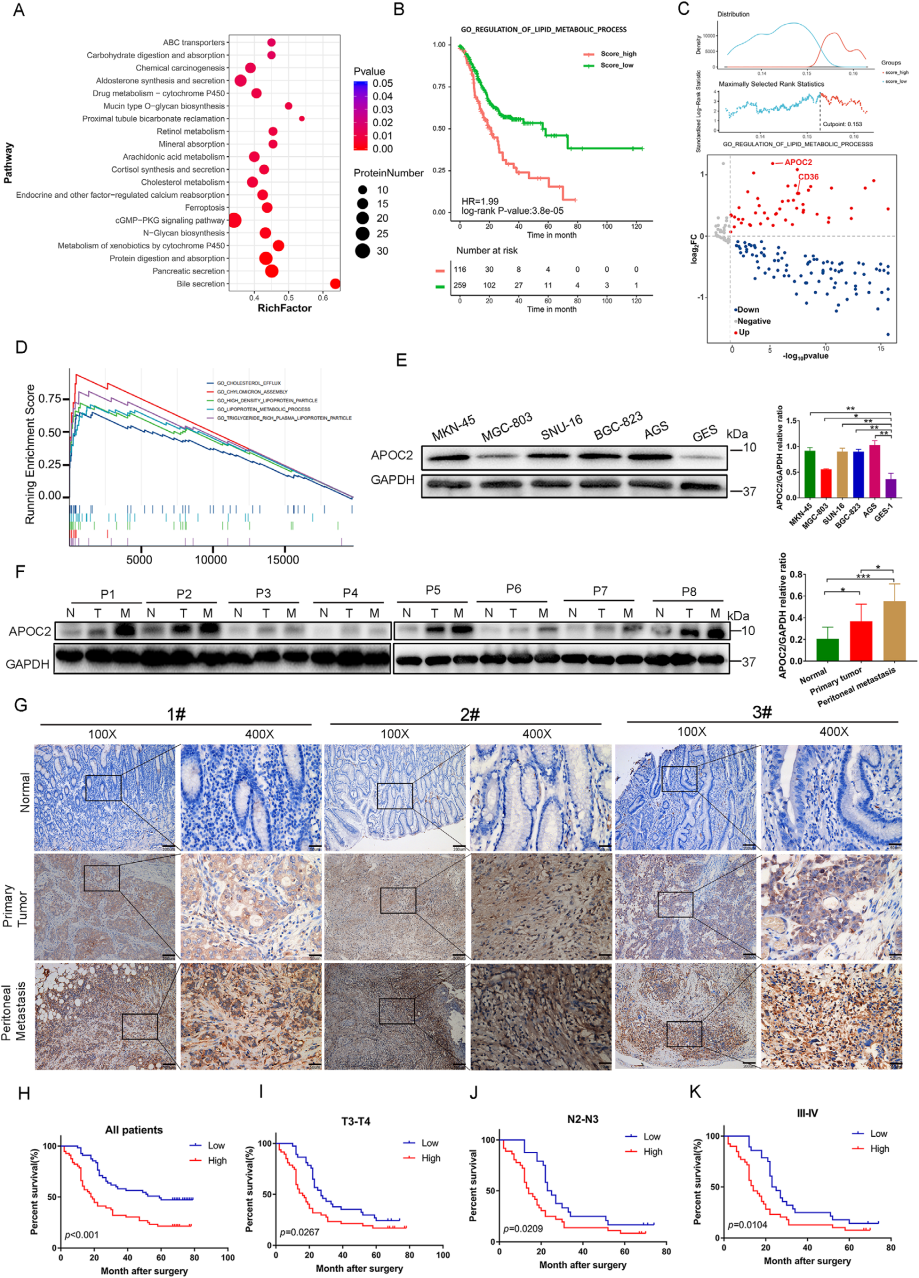
APOC2 is upregulated in GC patients with PM. (A) KEGG pathway enrichment analysis of DEGs. (B) Lipid metabolism gene expression was negatively correlated with survival prognosis according to TCGA STAD data set (HR = 1.99 [95%IC:1.43–2.78]; log‐rank *p* value = 3.8×10^–5^). GSVA algorithm with the R package GSVA (version 1.36.2) was used to score the gene set for 375 cancer samples in TCGA‐STAD cohort.The normalized score, ranging from 0.1184 to 0.1836, along with the clinical prognosis data of TCGA‐STAD cohort was further submitted to the R package survminer (version 0.4.8) to find the best cutoff point to plot the Kaplan–Meier survival curve. TCGA‐STAD cohort was subdivided into lipid metabolism active group (*n* = 116) and lipid metabolism inactive group (*n* = 259) based on the best cutoff point. (C) Volcano plot showed the fold change of identified proteins between PM tissues (*n* = 5) and GC tissues (*n* = 5) in the gene set of “regulation of lipid metabolic process.” (D) GSEA analysis revealed that APOC2 was mainly related to lipid absorption, transport, and metabolism of cells based on TCGA STAD database. (E) Western blot analysis of APOC2 protein levels in various GC cell lines and normal gastric epithelial cell line GES‐1. (F) Western blot was used to detect protein expression of APOC2 from eight patients with GC in tumor tissues, adjacent noncancerous gastric tissues (ANTs), and PM tissues. (G) Immunohistochemical (IHC) staining analysis of APOC2 protein levels in patients with GC. Representative images of APOC2 levels in ANTs, GC tissues, and GC PM tissues are shown. (H–K) The overall survival of patients with high or low APOC2 expression in GC tissues. Kaplan–Meier test was used to analyze *p* values. Data represent mean ± SD, *p < 0.05, **p < 0.01, ***p < 0.001, ****p < 0.0001, based on Student's *t*‐test

Through KEGG biological enrichment analysis, we found that the DEPs identified in PM samples were mainly related cholesterol metabolism and transport (Figure 2A). Therefore, we assumed that lipid metabolism acted as an essential regulator in PM. To further verify the association between lipid metabolism‐related genes and survival of patients with primary GC, the gene set “GO_REGULATION_OF_LIPID_METABOLIC_PROCESS” (*n* = 383, Table S11) downloaded from mSigDB was chosen representing the activity of lipid metabolism, which includes all genes regulating lipid metabolism. Then, we scored the gene set for 375 cancer samples in TCGA‐STAD cohort utilizing the GSVA Algorithm with the R package GSVA (version 1.36.2). The normalized score, ranging from 0.1184 to 0.1836, along with the clinical prognosis data of TCGA‐STAD cohort, was further submitted to the R package survminer (version 0.4.8) to find the best cutoff point, which is 0.153, and plot the Kaplan‐Meier survival curve. Then, the TCGA‐STAD cohort was further subdivided into lipid metabolism active group (*n* = 116) and lipid metabolism inactive group (*n* = 259) based on the best cutoff point. We observed that in patients with GC, lipid metabolism gene expression was negatively correlated with survival prognosis, which further indicated that lipid metabolism might influence GC progression (Figure 2B, HR = 1.99 [95%IC:1.43‐2.78]; log‐rank *p* value = 3.8×10^–5^). To identify possible core protein of lipid metabolism in PM, we visualized the FC of proteins in the gene set of “regulation of lipid metabolic process” using a volcano plot, and found that the protein with the highest log2FC was APOC2 in PM tissues (*n* = 5) compared with GC tissues (*n* = 5) (Figure 2C). In order to further verify the function of APOC2 in GC, we analyzed the relevant GC sequencing data from the TCGA STAD database. According to the level of *APOC2* mRNA expression, we divided 375 GC tissue samples into two groups: High_APOC2 expression (*n* = 188) and Low_APOC2 expression (*n* = 187). After performing the differential gene expression analysis between two groups and the downstream gene set enrichment analysis, we observed that APOC2 was mainly related to lipid absorption, transport, and metabolism of cells (Figure 2D). This finding was consistent with the results of our proteomics analysis. Based on the analysis of TCGA STAD data set, we also found that *APOC2* mRNA expression was higher in samples from 375 GC patients than in 32 normal samples (Figure S6A). To illustrate whether the upregulation of APOC2 in GC is mediated by epigenetic mechanisms, the DNA methylation data of 27 paracancerous tissues and 443 cancerous tissues were downloaded from UCSC Xena. Only one DNA methylation probe, cg13119609, was identified as probe for APOC2. The DNA methylation data were further normalized by the R package CHAMP (version 2.18.3). The expression level of APOC2 and the beta value of cg13119609 were visualized through boxplot using the R package ggpubr (version 0.4.0). Wilcoxon Signed‐Rank test was used to determine statistical significance. Interestingly, we found that the *APOC2* methylation β value of the GC samples was significantly lower than that of the normal samples (Figure S6B). In order to clarify the *APOC2* gene methylation levels in normal gastric mucosal tissues, GC tissues, and PM tissues, we extracted the patient's normal gastric mucosal tissues, GC tissues, and PM tissues for next generation sequencing (NGS)‐based DNA methylation profiling. Then, referring to previous research on *APOC2* gene methylation detection,^11^ we performed NGS‐based DNA methylation profiling for the *APOC2* gene region (hg19_dna range = chr19:45447831‐45449574), including the CpG site (MAPINFO:45449297). The results showed that compared with normal tissues, the methylation ratio of some *APOC2* gene methylation sites (MAPINFO: 45449258, MAPINFO: 45449354, and MAPINFO: 45449392) in GC tissues was significantly reduced, and the methylation ratio of these sites was the lowest in PM tissues (Figure S6C). This may partly explain why *APOC2* was highly expressed in GC.

We further revealed that APOC2 was higher in GC cell lines, including AGS, MKN‐45, MGC‐803, SNU‐16, and BGC‐823, than in gastric epithelial cell line GES‐1 (Figure 2E). In addition, the mRNA and protein levels of APOC2 in tissues from operation (eight samples) were assessed via RT‐qPCR and Western blot. The results indicated that compared with adjacent the normal gastric mucosal tissue (ANTs, N), APOC2 in GC tissue (T) was significantly increased, while PM lesions (M) showed the highest expression of APOC2 (Figure 2F and Figure S6D). Representative IHC experiment demonstrated similar results (Figure 2G). Larger cohort containing 111 pairs of GC and adjacent tissues generated consistent expression pattern of APOC2 in GC (Figure S6E). Table 1 summarizes the clinicopathological features of 111 patients. The higher expression of APOC2 was related to shorter overall survival (OS) in GC patients (Figure 2H). Subgroup analysis demonstrated that higher APOC2 expression was associated with shorter OS as well (Figure 2I‐K). As APOC2 is a secretory protein, we also analyzed the serum APOC2 levels of in 66 patients with GC using ELISA. APOC2 was overexpressed in GC serum specimens that was positively associated with AJCC Stage, M stage and T stage, N stage. Table S12 summarizes the clinicopathological features of these patients.

**TABLE 1:** Associations between APOC2 expression and clinical pathological characteristics in patients with GC

Variable	*N*	Low (APOC2)	High (APOC2)	*p* value
Age				0.241
<60	34	14	20	
≥60	77	41	36	
Gender				0.2556
Male	82	38	44	
Female	29	17	12	
Differentiation				0.3689
Well	32	18	14	
Poor	79	37	42	
Lauren type				0.2406
Intestinal	41	18	23	
Diffuse	34	15	19	
Mixed	36	22	14	
Depth of invasion (T)				0.0409*
T1+T2	27	18	9	
T3+T4	84	37	47	
Lymph node metastasis (N)				0.0291*
N0+N1	51	31	20	
N2+N3	60	24	36	
TNM stage				0.0437*
I+ II	44	27	17	
III + IV	67	28	39	

^*^ *p* < 0.05.

### 3.3 Knockdown of APOC2 inhibits the biological behavior, apoptosis resistance, and EMT in GC

**FIGURE 3:**
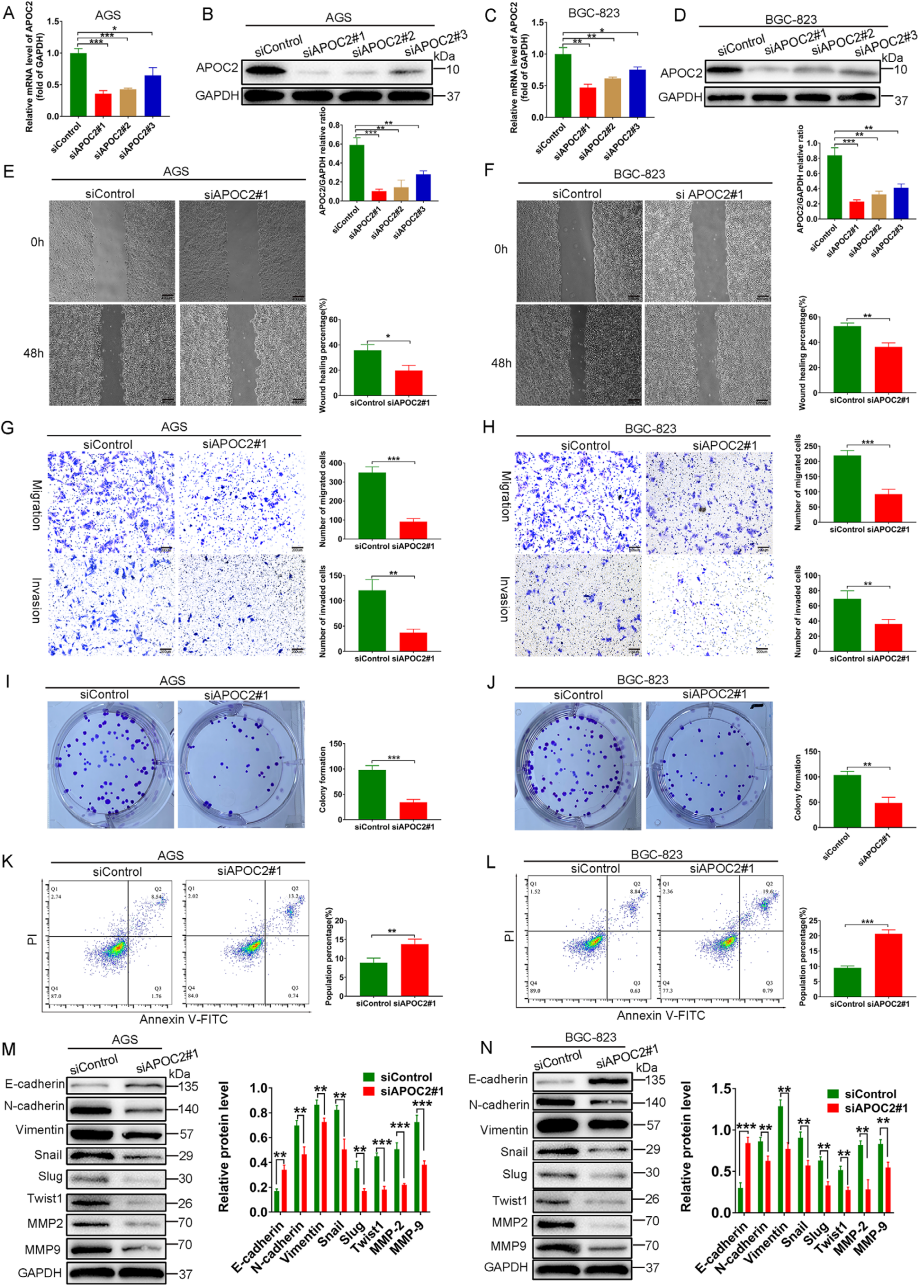
Knockdown of APOC2 inhibits the biological behavior, apoptosis resistance, and EMT in GC. (A–D) PCR and western blot were used to evaluate the knockout efficiency of the APOC2‐specific small interfering RNAs (siRNAs) (siAPOC2#1, siAPOC2#2, and siAPOC2#3) in AGS and BGC‐823 cells. (E, F) Cell mobility analyzed using a wound healing assay. (G, H) Migration and invasion of siAPOC2#1 and siControl cells were detected using Transwell migration and invasion assays. (I, J) Colony formation assay of AGS and BGC‐823 cells transfected with siAPOC2#1 and siControl. (K, L) Propidium iodide (PI) and Annexin V staining levels were determined by flow cytometry to assess cell apoptosis in AGS and BGC‐823 cells transfected with siAPOC2#1 and siControl. (M, N) Western blot analysis of the protein levels of E‐cadherin, N‐cadherin, vimentin, Snail, Slug, Twist1, MMP‐2, and MMP‐9 in AGS and BGC‐823 cells transfected with siAPOC2#1 and siControl. Data are shown as mean ± SD; *p < 0.05, **p < 0.01, ***p < 0.001, based on Student's *t*‐test

To explore APOC2's biological function in GC, we transfected AGS and BGC‐823 cells with control siRNA and *APOC2*‐siRNAs (Si1, Si2, and Si3). After 72 h treatment, siAPOC2#1 was the most efficient in suppressing APOC2 in both cell lines (Figure 3A‐D). Knockdown of *APOC2* significantly inhibited cell wound healing (Figure 3E, F), migration (Figure 3G, H), invasion (Figure 3G, H), clonal formation (Figure 3I, J), and apoptosis resistance (Figure 3K, L) of both AGS and BGC‐823 cells. Moreover, *APOC2* knockdown significantly induced E‐cadherin, while inhibited N‐cadherin, vimentin, Snail, Slug, Twist1, MMP‐2, and MMP‐9 levels (Figure 3M, N). Together, these data indicated that APOC2 inhibited the biological behavior, apoptosis resistance, and EMT in GC.

### 3.4 Knockdown of APOC2 suppresses metabolic activity and inhibits PI3K/AKT/mTOR signaling in GC

**FIGURE 4:**
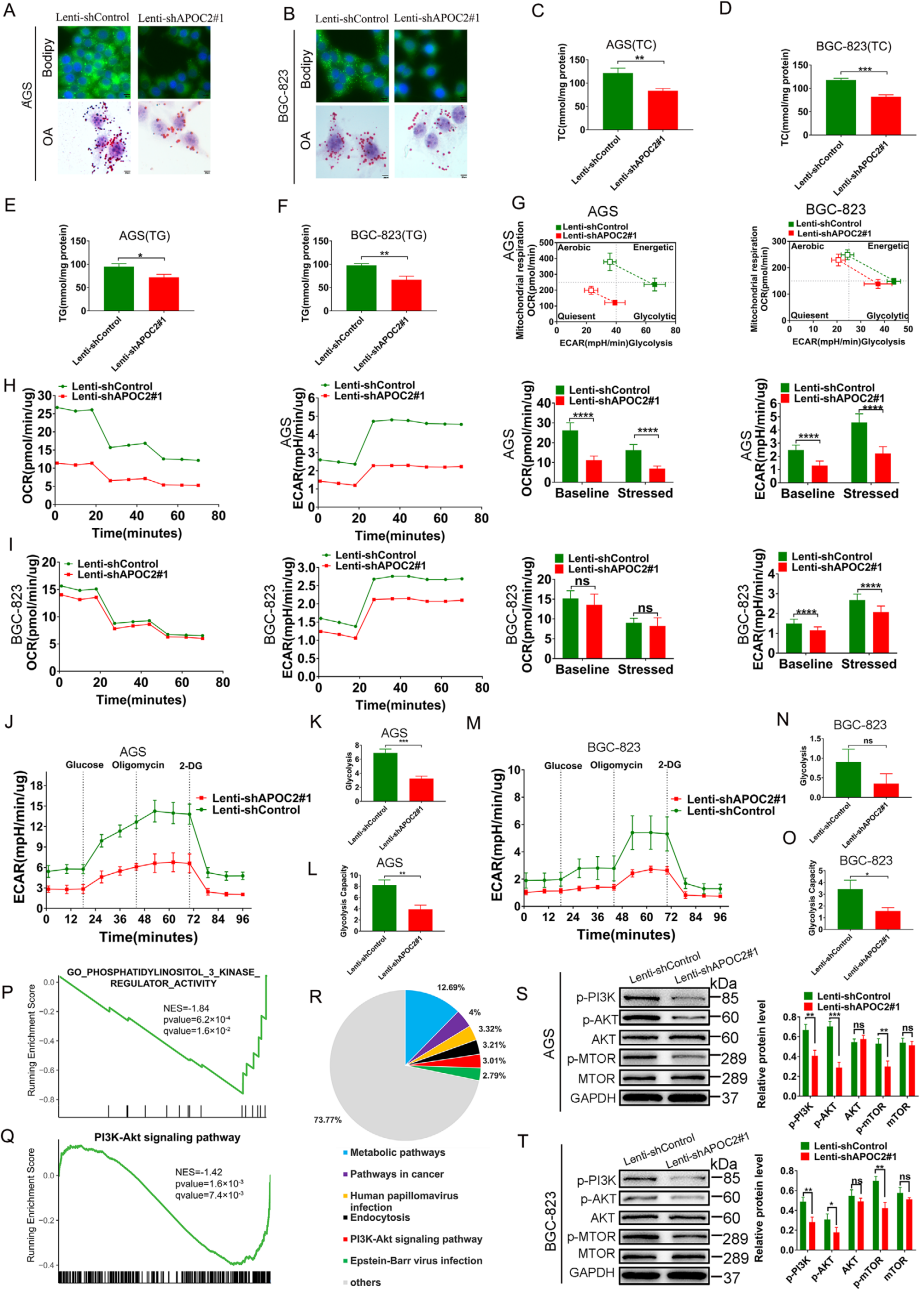
Knockdown of APOC2 suppresses metabolic activity and inhibits PI3K/AKT/mTOR signaling in GC. (A, B) Representative images of lipid droplets (LDs) in AGS and BGC‐823 cells transfected with Lenti‐shAPOC2#1 and Lenti‐shControl as evaluated using BODIPY 493/503 staining (green) and Oil Red O staining; cell nuclei were stained with 4′,6‐Diamidino‐2‐Phenylindole (blue). (C, D) Concentration of total cholesterols (TCs) in AGS and BGC‐823 cells transfected with Lenti‐shAPOC2#1 and Lenti‐shControl. (E, F) Concentration of triglyceride (TGs) in AGS and BGC‐823 cells transfected with Lenti‐shAPOC2#1 and Lenti‐shControl. (G) Cell energy phenotype of Lenti‐shAPOC2#1 and Lenti‐shControl AGS and BGC‐823 cells. Baseline phenotype is indicated by an open marker. Stressed phenotype is indicated by a filled marker. (H, I) The oxygen consumption rate (OCR) and extracellular acidification rate (ECAR) of AGS and BGC‐823 cells transfected with Lenti‐shAPOC2#1 and Lenti‐shControl tested by Seahorse XF cell analysis. (J–O) Glycolytic capacity of AGS and BGC‐823 cells transfected with Lenti‐shAPOC2#1 and Lenti‐shControl was measured by glycolytic stress test, the key parameters of glycolytic flux, including glycolysis and glycolytic capacity, were shown. (P, Q) GO (P) and KEGG pathway (Q) analysis of these differentially expressed genes revealed that the PI3K/AKT signaling was inhibited in *APOC2* knockdown cells using GSEA. (R) KEGG annotations of the identified proteins from GC and PM tissues. (S, T) After the cells were transfected with Lenti‐shAPOC2#1 and Lenti‐shControl, the proteins in the PI3K/AKT/mTOR pathway of AGS and BGC‐823 cells were evaluated using western blot. Data are shown as mean ± SD; ns, no significant difference; *p < 0.05, **p < 0.01, ***p < 0.001, ****p < 0.0001, based on Student's *t*‐test

To further explore the mechanism of APOC2 in regulating the lipid metabolism processes of GC, we first infected GC cells with lentivirus‐shAPOC2 (to stably silence *APOC2*) and the control nontargeting shControl. Western blot demonstrated the efficiency of knockdown in AGS and BGC‐823 cells, respectively (Figure S7A). Then, we performed Oil Red O and BODIPY 493/503 staining assays and found that *APOC2* knockdown significantly suppressed lipid droplet (LD) formation in AGS (Figure 4A) and BGC‐823 cells (Figure 4B). *APOC2* knockdown also markedly inhibited the total cholesterol (TC) (Figure 4C, D) and TG (Figure 4E, F) synthesis in GC cells. These data suggested a pivotal role of APOC2 in lipid metabolism and transportation. Furthermore, we used Seahorse XF to measure the live‐cell metabolic index, and found that Lenti‐shAPOC2#1 cells exhibited lower energetic metabolic phenotype compared with controls (Figure 4G). Under basal conditions, the mitochondrial respiration rate of shAPOC2 AGS cells (Figure 4H) but not shAPOC2 BGC‐823(Figure 4I) decreased, which was evaluated by OCR. However, compared with shCtrl cells, both shAPOC2 AGS and shAPOC2 BGC‐823 cells had a lower ECAR under stress conditions (Figure 4H, I). Furthermore, the glycolytic stress test showed that *APOC2* knockdown reduced glycolysis levels and glycolytic capacity of AGS cells (Figure 4J‐L), but unable to affect the glycolysis level of BGC‐823 cell (Figure 4M‐O). Then, we tested whether APOC2 can affect the expression of glycolysis‐related proteins (GLUT1, HK2, PKM2, and LDHA).^37^ We found that *APOC2* knockdown significantly inhibited the expression of HK2 in AGS and BGC‐823 cells, except for GLUT1, PKM2, and LDHA. Regarding whether APOC2 can regulate the expression of lipid metabolism‐related proteins, we verified the relationship between *APOC2* knockdown and its closely related lipid metabolism proteins (LIPL, LDLR, and LRP1).^38^, ^39^, ^40^ We found that *APOC2* knockdown significantly inhibited the expression of LIPL in AGS (Figure S7C) and BGC‐823 cells (Figure S7D), but did not affect the expression of LDLR and LRP1.

We then performed transcriptome sequencing of AGS and AGS‐shAPOC2 cells. The mRNA‐seq analysis identified abundant gene expression changes after *APOC2* knockdown. GO (Figure 4P) KEGG pathway (Figure 4Q) analysis of these differentially expressed genes revealed that PI3K/AKT signaling was inhibited in *APOC2* knockdown cells using GSEA. KEGG pathway analysis of all identified proteins in our samples showed that the highly enriched pathways included metabolic pathway and PI3K‐AKT signaling (Figure 4R). It has been described that PI3K/AKT/mTOR axis drives EMT to promote tumor metastasis.^41^, ^42^ The role of the mTOR complex in lipid metabolism is particularly important.^43^ Combined with the results of transcriptomics, we speculated that APOC2 may serve an irreplaceable role in regulating PI3K/AKT/mTOR pathway in GC. Then, we proved that *APOC2* knockdown inhibited the phosphorylation of PI3K, AKT, and mTOR, which was consistent with the RNA‐seq data (Figure 4S, T). Together, these data indicated that APOC2 was essential to regulate metabolism and PI3K/AKT/mTOR signaling in GC.

### 3.5 Effects of APOC2 in GC depend on PI3K/AKT/mTOR signaling

**FIGURE 5:**
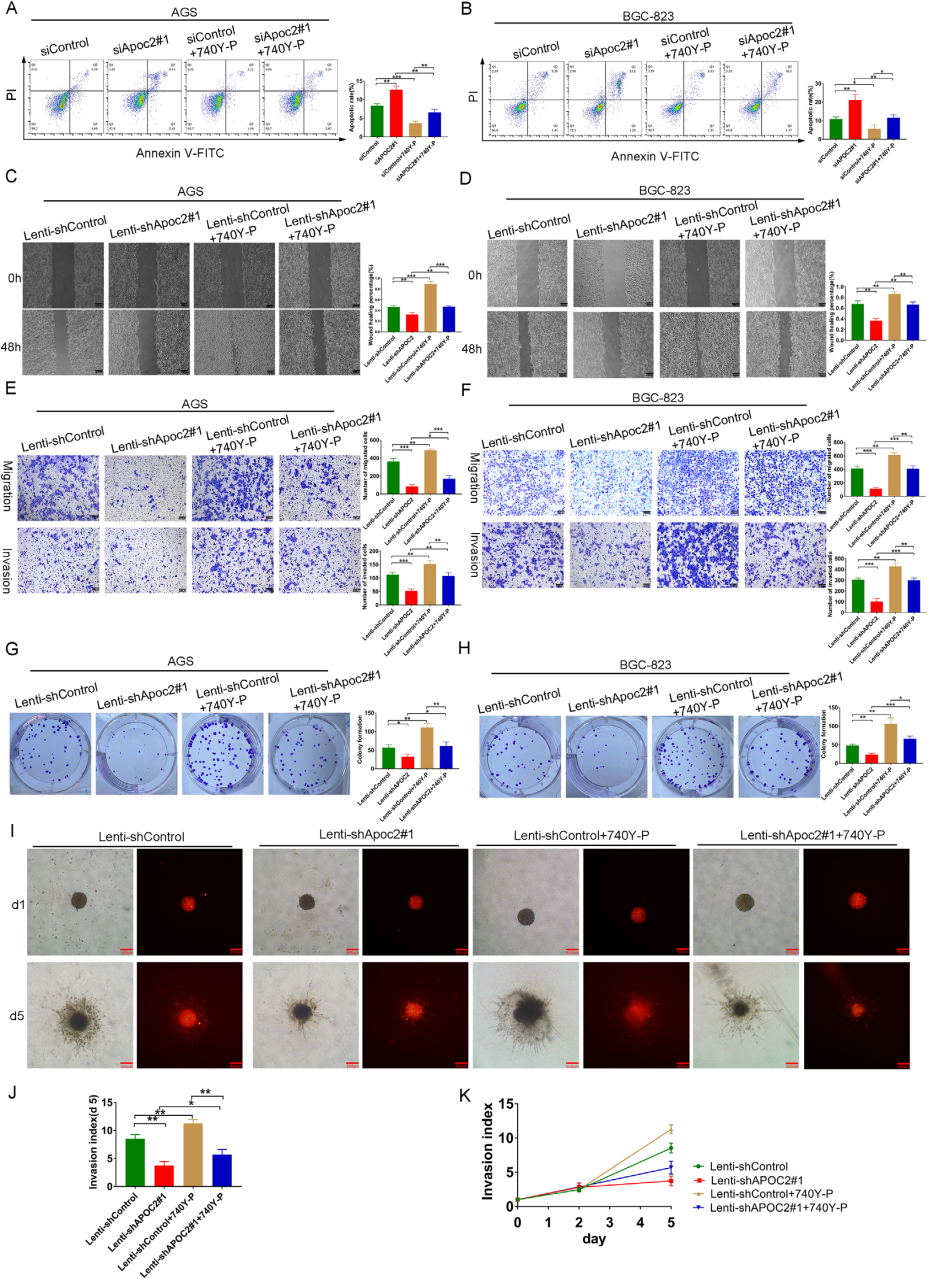
Inhibition of APOC2 suppresses cell migration, invasion, proliferation, and increases apoptosis in GC cells. (A, B) Flow cytometry was used to assess cellular apoptosis, with or without APOC2 inhibition, in GC cells, as well as with or without the PI3K pathway activator by 740Y‐P. (C–H) Wound healing (C), transwell (E), and colony formation (G) assays were performed to detect the migration, invasion, and proliferation ability of GC cells transfected with Lenti‐shAPOC2#1 and Lenti‐shControl, as well as those of cells treated with or without 740Y‐P. (I–K) 3D invasion assays revealed that *APOC2* silencing suppressed BGC‐823 invasion significantly, and 740Y‐P abolished the effect of Lenti‐shAPOC2 on GC cell Invasion. Data represent mean ± SD of three independent experiments. Data are shown as mean ± SD; ns, no significant difference; *p < 0.05, **p < 0.01, ***p < 0.001, based on Student's *t*‐test

Next, we sought to explore the indispensability of PI3K/AKT/mTOR signaling in APOC2‐regulated GC behaviors. Compared with control group, AGS and BGC‐823 apoptosis in the siAPOC2 group increased significantly, while that in 740Y‐P group (PI3K pathway activator) decreased significantly. Notably, 740Y‐P could partially compensate siAPOC2‐induced cell apoptosis (Figure 5A, B). Wound healing, migration, invasion, and the colony formation ability of GC cells were significantly reduced in shAPOC2 group and increased in 740Y‐P group, while 740Y‐P restored *APOC2* knockdown’ effects (Figure 5C–H). Similarly, 3D invasion assays showed that *APOC2* silencing suppressed cell invasion, and 740Y‐P abolished the effect of shAPOC2 on GC cell invasion (Figure 5I‐K).

**FIGURE 6:**
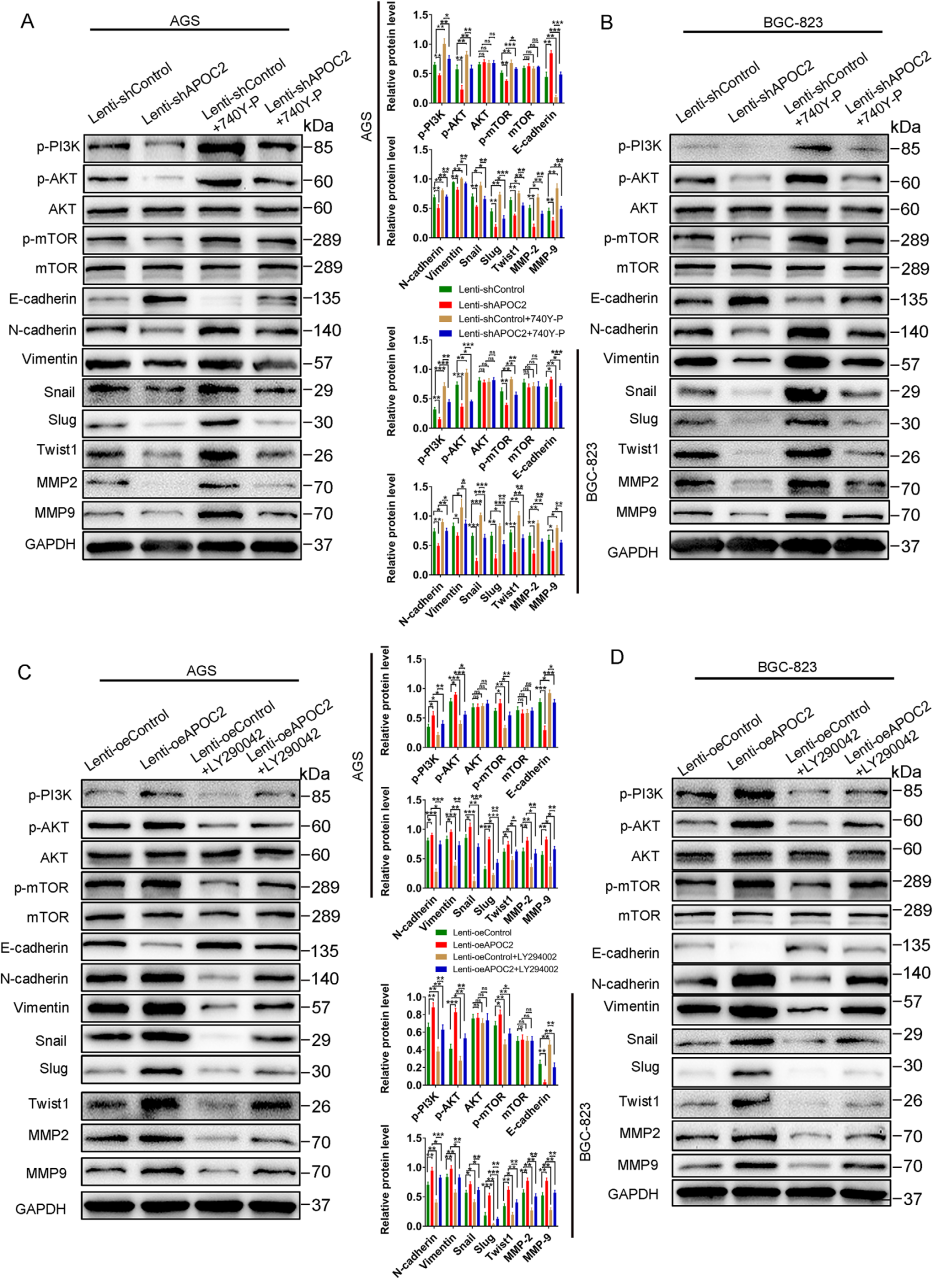
APOC2 mediates EMT via PI3K/AKT/mTOR signaling in GC. (A, B) Western blot analysis of the protein levels of p‐PI3K, p‐AKT, p‐mTOR, E cadherin, N‐cadherin, vimentin, Snail, Slug, Twist1, MMP‐2, and MMP‐9 in GC cells transfected with Lenti‐shAPOC2#1 and Lenti‐shControl, as well as cells treated with or without the PI3K pathway activator 740Y‐P. (C, D). Western blot analysis of the protein levels of p‐PI3K, p‐AKT, p‐mTOR, E‐cadherin, N‐cadherin, vimentin, Snail, Slug, Twist1, MMP‐2, and MMP‐9 in AGS and BGC‐823 cells transfected with Lenti‐oeAPOC2 and Lenti‐oeControl, as well as cells treated with or without PI3K pathway inhibitor by LY294002. Data represent mean ± SD of three independent experiments. Data are shown as mean ± SD; ns, no significant difference; *p < 0.05, **p < 0.01, ***p < 0.001, based on Student's *t*‐test

Next, we observed that p‐PI3K, p‐AKT, and p‐mTOR were significant in shAPOC2 group. After transfection with Lenti‐shAPOC2, E‐cadherin was upregulated, whereas N‐cadherin, vimentin, Snail, Slug, Twist1, MMP‐2, and MMP‐9 were downregulated in GC cells (Figure 6A, B). Treatment with 740Y‐P could compensate effects by Lenti‐shAPOC2 (Figure 6A, B). Lentiviral transfection was used to construct GC cells stably overexpressing *APOC2*. The efficiency of overexpression in GC cells was verified by western blotting (Figure S7B). Wound healing, migration, invasion, and colony formation ability of GC cells were greatly increased in the *APOC2*‐overexpressing group and reduced in the LY294002 (a selective inhibitor of PI3K‐dependent AKT phosphorylation and kinase activity) group, while LY294002 restored the effects of *APOC2* overexpression (Figure S8A‐C). Moreover, the phosphorylation of PI3K, AKT, and mTOR was enhanced in *APOC2*‐overexpressing GC cells. Overexpression of *APOC2* also increased N‐cadherin, Vimentin, Snail, Slug, Twist1, MMP‐2, and MMP‐9, while suppressed E‐cadherin expression. In addition, LY294002 inhibited PI3K‐AKT‐mTOR signaling and EMT of GC cells (Figure 6C, D).

### 3.6 APOC2 cooperates with CD36 to promote tumor progression and PM in GC

APOC2 has been found to interact with CD36 in atherosclerosis^24^ and AML.^11^ CD36 has also been reported in regulating lipid metabolism and EMT process of GC cells.^21^, ^22^, ^23^ Our proteomics data (Figure S4A) and western blot (FigureS4B) showed that CD36 was highly expressed in PM tissues.

**FIGURE 7:**
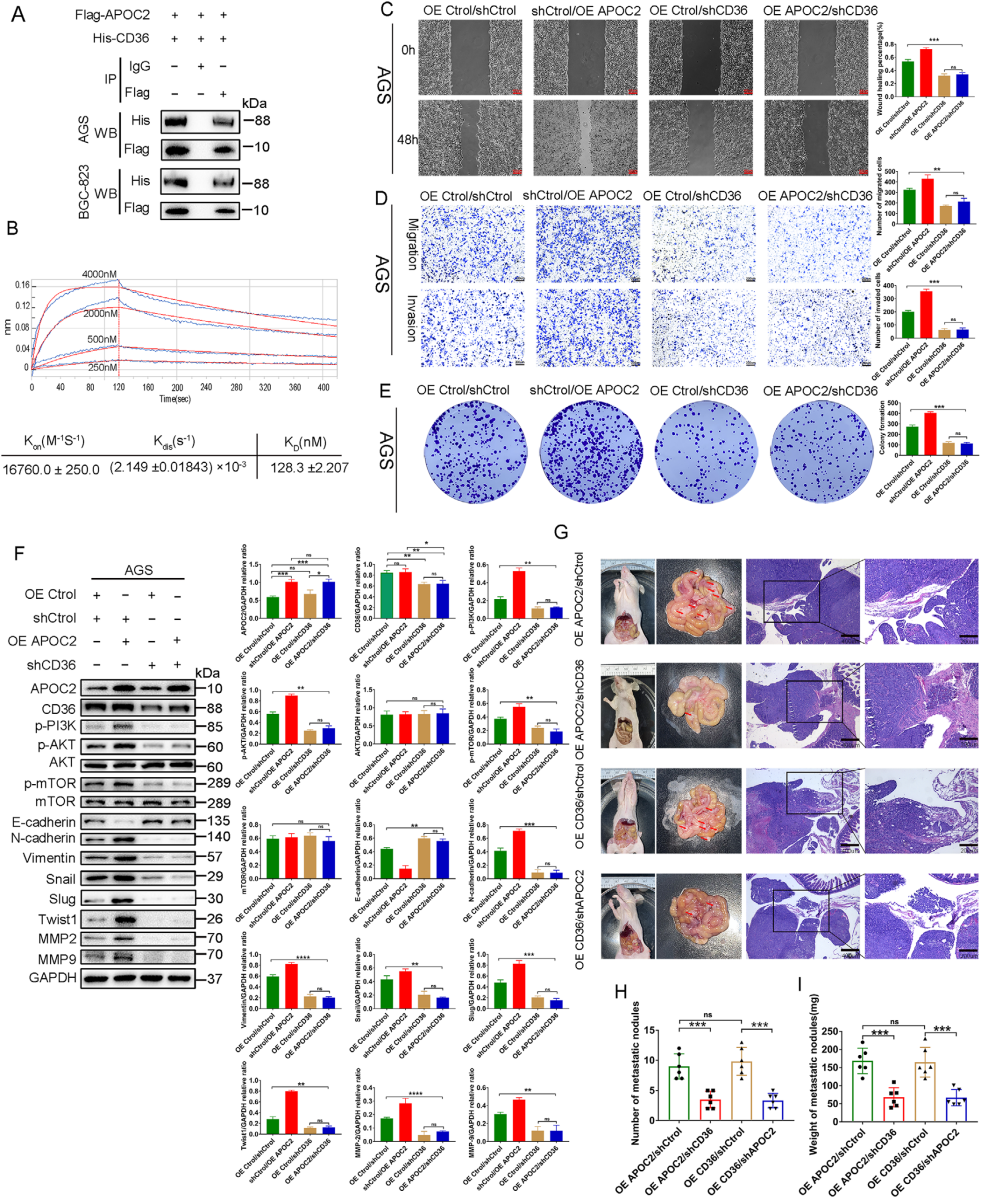
APOC2 cooperates with CD36 to promote tumor progression and PM in GC. (A) Flag‐APOC2 interacts with His‐CD36 in AGS and BGC‐823 cells. The cells were cotransfected with Flag‐APOC2 and His‐CD36. For the co‐IP assays, anti‐FLAG antibody was used for pulldown and anti‐His antibody was detected by western blot. (B) Measurement of the binding affinity of APOC2 peptide to CD36 by biolayer interferometry method. Various concentrations of APOC2 peptide were shown. (C–E) The effect of knocking down *CD36* on the migration, invasion, and proliferation of OE APOC2 stable cells. (F) The levels of p‐PI3K, p‐AKT, p‐mTOR, E cadherin, N‐cadherin, vimentin, Snail, Slug, Twist1, MMP‐2, and MMP‐9 proteins detected by western blot in OE APOC2 AGS cells transduced with shCD36 lentiviral particles. (G–I) Representative images of the macroscopic appearance and hematoxylin and eosin (H&E) staining of PM nodules in nude mice treated with intraperitoneal injection of AGS OE APOC2/shCtrol, AGS OE APOC2/shCD36, AGS OE CD36/shCtrol, and AGS OE CD36/shAPOC2 cells, respectively (*n* = 6 per group). The total number (H) and weight (I) of PM nodules in the respective groups. Data are shown as mean ± SD; ns, no significant difference; *p < 0.05, **p < 0.01, ***p < 0.001, ****p < 0.0001, based on Student's *t*‐test

The expression level of *CD36* gene was negatively correlated with the prognosis of GC patients (Figure S4F), and CD36 could regulate EMT of GC (Figure S5K, L). Then, we cotransfected AGS or BGC‐823 cells with Flag‐APOC2 and His‐CD36 for 48 h to co‐IP. Western blot showed that APOC2 was able to interact with CD36 in GC cells (Figure 7A). In addition, BLI analysis demonstrated the binding affinity of CD36 and APOC2 (Figure 7B). The calculation of the values was: *K*_on_ = 16,760.0 ± 250.0 M^−1^ S^−1^ for the association phase, *K*_dis_ = (2.149 ±0.01843) ×10^–3^ S^−1^ for the dissociation phase, and *K_D_* = 128.3 ± 2.207 nM for overall dissociation.

To explore the biological function of CD36 in GC cells, we first constructed CD36‐overexpressed (OE CD36) GC cell lines (Figure S9A), and then selected sequence #3 with the highest silencing efficiency to construct shCD36 lentiviral system (Figures S5J and S9B). Interestingly, compared with OE APOC2 or OE CD36 cells, OE APOC2 + OE CD36 cells exhibited improved migration, invasion, and proliferation (Figures S9C, D and S10A‐C). Likewise, simultaneous overexpression of CD36 and APOC2 triggered an induction in p‐PI3K, p‐AKT, p‐mTOR, N‐Cadherin, vimentin, snail, Slug, Twist1, MMP‐2, and MMP‐9 but a reduction in E‐cadherin (Figures S9F and S10D).

To further verify the functional relationship between APOC2 and CD36, we transduced shAPOC2 or shCD36 GC cells with lentiviral particles overexpressing CD36 or APOC2, respectively. We found that *CD36* knockdown in AGS (Figure 7C‐E) and BGC‐823 (Figure S11A‐C) cells abolished the APOC2‐induced cell migration, invasion, and proliferation. Knockdown of *APOC2* (Figure S12A‐C) in AGS and BGC823 (Figure S13A‐C) cells abolished the CD36‐induced cell migration, invasion, and proliferation. More importantly, the inhibition of E‐cadherin by OE APOC2 or OE CD36 could be recovered by *CD36* knockdown (Figure 7F and Figure S11D) or *APOC2* knockdown (Figures S12D and S13D), respectively. Similarly, knockdown of *CD36* (Figure 7F and Figure S11D) or *APOC2* (Figures S12D and S13D) abolished the effect of APOC2 or CD36‐induced p‐PI3K, p‐AKT, p‐mTOR, N‐Cadherin, vimentin, snail, Slug, Twist1, MMP‐2, and MMP‐9 expression. These data indicated that both APOC2 and CD36 were necessary for the migration, invasion, and proliferation.

Next, we established a peritoneal dissemination model and xenograft GC model in nude mice. We found that *CD36* or *APOC2* knockdown attenuated the effect of APOC2 or CD36‐induced GC PM (Figure 7G) and GC proliferation (Figure S14A). Treatment with OE APOC2/shCD36 or OE CD36/shAPOC2 led to significantly fewer PM nodules (Figure 7H), lighter PM nodules (Figure 7I), and less subcutaneous tumors (Figure S14B‐D). Taken together, these data suggested that APOC2 cooperated with CD36 to promote tumor progression and PM in GC.

### 3.7 In‐vivo validation

**FIGURE 8:**
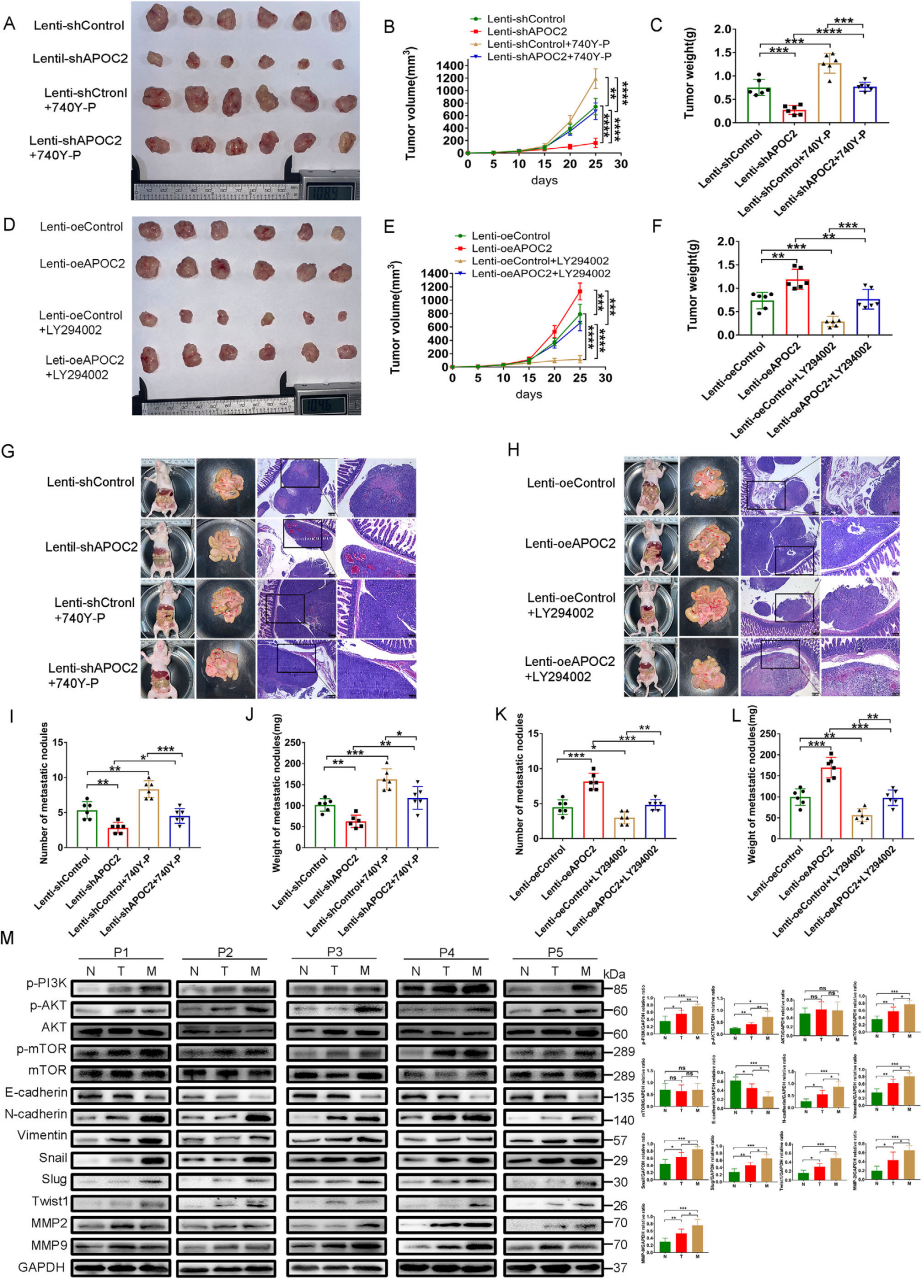
APOC2 promotes tumor progression and PM of GC cells in vivo via the PI3K/AKT/mTOR pathway. (A–C) Photograph of subcutaneous tumors excised from nude mice treated with 740Y‐P or intraperitoneal injection of AGS cells stably infected with Lenti‐shControl or Lenti‐shAPOC2 lentiviral particles (*N* = 6 per group). Tumors were observed and recorded by tumor volume (B) and tumor weight (C). (D–F) Photograph of subcutaneous tumors excised from nude mice treated with LY294002 or intraperitoneal injection of AGS cells stably infected with Lenti‐oeControl or Lenti‐oeAPOC2 lentiviral particles (*N* = 6 per group). Tumors were observed and recorded by tumor volume (E) and tumor weight (F). (G) Representative images of the macroscopic appearance and hematoxylin and eosin (H&E) staining of PM nodules in nude mice treated with the PI3K pathway activator by 740Y‐P or intraperitoneal injection of AGS cells stably infected with Lenti‐shControl or Lenti‐shAPOC2 lentiviral particles (*N* = 6 per group). (I, J) The total number (I) and weight (J) of PM nodules in the respective groups. (H) Representative images of the macroscopic appearance and H&E staining of PM nodules in nude mice treated with PI3K pathway inhibitor by LY294002 or intraperitoneal injection of AGS cells stably infected with Lenti‐oeControl or Lenti‐oeAPOC2 lentiviral particles (*N* = 6 per group). (K, L) The total number (K) and weight (L) of PM nodules in the respective group. (M) Western blot was used to detect proteins expression of p‐PI3K, p‐AKT, p‐mTOR, E‐cadherin, N‐cadherin, vimentin, Snail, Slug, Twist1, MMP‐2, and MMP‐9 from five patients with GC in tumor tissues, ANTs, and PM tissues. Data are shown as mean ± SD; ns, no significant difference; *p < 0.05, **p < 0.01, ***p < 0.001, ****p < 0.0001, based on Student's *t*‐test

To confirm the effects of APOC2 on the tumorigenicity of GC *in vivo*, we established a subcutaneous xenograft model. As shown in Figure 8A‐F, the tumor volume and weight were decreased by lenti‐shAPOC2‐transfected cells, while increased by lenti‐oeAPOC2‐transfected cells. Activation of PI3K signaling by 740Y‐P induced tumor proliferation in vivo, and could partially reverse the impact of *APOC2* knockdown (Figure 8A‐C). Inhibition of PI3K signaling by LY29004 was capable to suppress the tumor progression by *APOC2* overexpression (Figure 8D‐F).

To determine the effect of APOC2 on the PM of GC *in vivo*, we established a peritoneal dissemination model. AGS shAPOC2 cells produced fewer and lighter PM nodules compared with those by AGS‐shControl cells (Figure 8G,8I, J). In contrast, activation of PI3K signaling by 740Y‐P induced PM in vivo, and could partially reverse the impact of *APOC2* knockdown on PM. Furthermore, a PM model was established using *APOC2*‐overexpressing AGS cells (Figure 8H). As expected, *APOC2* overexpression increased the number (Figure 8K) and weight (Figure 8L) of the PM nodules. Inhibition of PI3K signaling by LY294002 inhibited PM in vivo, which was enhanced by Lenti‐oeAPOC2. In addition, we also found that the expression of EMT markers and the activation of PI3K/AKT/mTOR signaling were upregulated in PM (Figure 8M). These data indicated that APOC2 promoted proliferation and PM of GC through PI3K/AKT/mTOR signaling pathway *in vivo*.

## 4 DISCUSSION

PM occurs frequently in GC, with a median survival of 7 months.^44^ Treatment for patients with PM is obscure, and temporary palliative strategy is largely based on experience.^45^, ^46^ Therefore, molecular mechanism of PM in GC is urgently expected for potential diagnostic and therapeutic target exploration.

RNA‐seq and proteomics mass spectrometry studies could help to find transcriptional and proteomic markers that lead to PM of GC.^47^, ^48^, ^49^ However, current sequencing samples are mainly obtained from ascites or plasma of patients, which hamper our understanding toward molecular or genomic composition of PM. To date, PM nodule tissue has not been used for protein mass spectrometry sequencing. Proteomics can provide a comprehensive and quantitative description of protein levels and their changes under the influence of biological interference, such as diseases or drug treatment.^50^ Tandem mass tag (TMT), an LC‐MS/MS‐based analysis strategy, is a very effective method for quantitative proteomics. It can identify proteins in different samples based on their relative abundance and quantify peptides and proteins using isotopomer labels, thereby creating large‐scale, highly precise data sets with minimal missing values, and have been used widely quantitative proteomics.^51^, ^52^, ^53^ Therefore, detailed proteomic characterization of PM samples combined with transcriptomic analysis of GC cells could reveal drivers that promote PM development.

In the present study, TMT‐based proteomic analysis revealed that 1600 proteins (595 upregulated and 1005 downregulated) were differentially abundant between GC and PM tissues. The bioinformatics analysis of DEPs showed that the highly expressed proteins in PM tissue were mainly related to lipid metabolism tumor extracellular matrix components. Further R‐language‐based analysis demonstrated that APOC2 was highly expressed in PMs and played a central role in lipid metabolism. Paget's “seed and soil” theory is a widely accepted theory of PM.^54^, ^55^ Metastatic homing of malignant cells is guided by the interaction between metastatic cancer cells and the microenvironment of specific organs.^56^ Cancer cells show higher energy requirements than normal gastric mucosal cells to achieve their increased invasion potential.^57^, ^58^ The relationship between lipid metabolism and cancer has been proven, but there are relatively few studies on the role of APOC2 in tumor.

As an important component of LDL (low‐density lipoproteins), VLDL (very low‐density lipoproteins), and HDL (‐density lipoproteins), APOC2 helps hydrolyze TGs related to lipoproteins via its ability to activate LPL.^7^ In the field of tumor‐related research, it has been shown that APOC2 is significantly associated with survival of pancreatic cancer.^59^ Pancreatic cancer cells with high APOC2 expression show enhanced cell invasion capability. Elevated APOC2 levels have been considered to be of diagnostic importance^60^ and associated with pancreatic cancer‐associated cachexia.^61^ In addition, according to the serum APOC2 expression level, the radiation treatment outcome of patients with locally advanced cervical cancer can be predicted and evaluated.^62^ Inhibiting *APOC2* expression can inhibit the invasion of gastrointestinal stromal tumor cells.^63^ APOC2 also promotes the growth of leukemia through the CD36‐ERK signaling.^11^ However, detailed elucidation of underlying mechanism in these diseases is awaited. We hypothesized that high APOC2 could help maintain the metabolic requirements of GC cells to further promote their metastasis to peritoneal cavity. We verified that APOC2 level was not only higher in GC cells, but also higher in PM tissues. A large clinical cohort containing 111 patients showed that APOC2 correlated with GC progression and reduced OS period, suggesting that APOC2 could serve as a prognostic biomarker for GC patients.

EMT has been reported to be critical for the development of cancer.^64^ Our proteomics data implied high expression of extracellular matrix components in PM tissues. We demonstrated that *APOC2* knockdown could ameliorate EMT process and malignant behavior of GC cells. Mechanistically, *APOC2* knockdown caused changes in lipid metabolism pathways and affected PI3K/AKT/mTOR signaling. We observed that APOC2 was responsible for the formation of LDs, the synthesis of TCs and TGs, as well as energy metabolism phenotype of GC cells. PI3K/AKT/mTOR signaling plays a key role in the EMT process.^35^, ^65^ EMT involves a transition from the expression of the epithelial marker to a cell state that favors the expression of mesenchymal markers.^66^ These changes are reflected by regulating the activity of EMT transcription factors (Slug, Snail, and Twist1).^67^ Our data demonstrated that PI3K/AKT/mTOR pathway participated in APOC2‐mediated EMT of GC cells.

**FIGURE 9:**
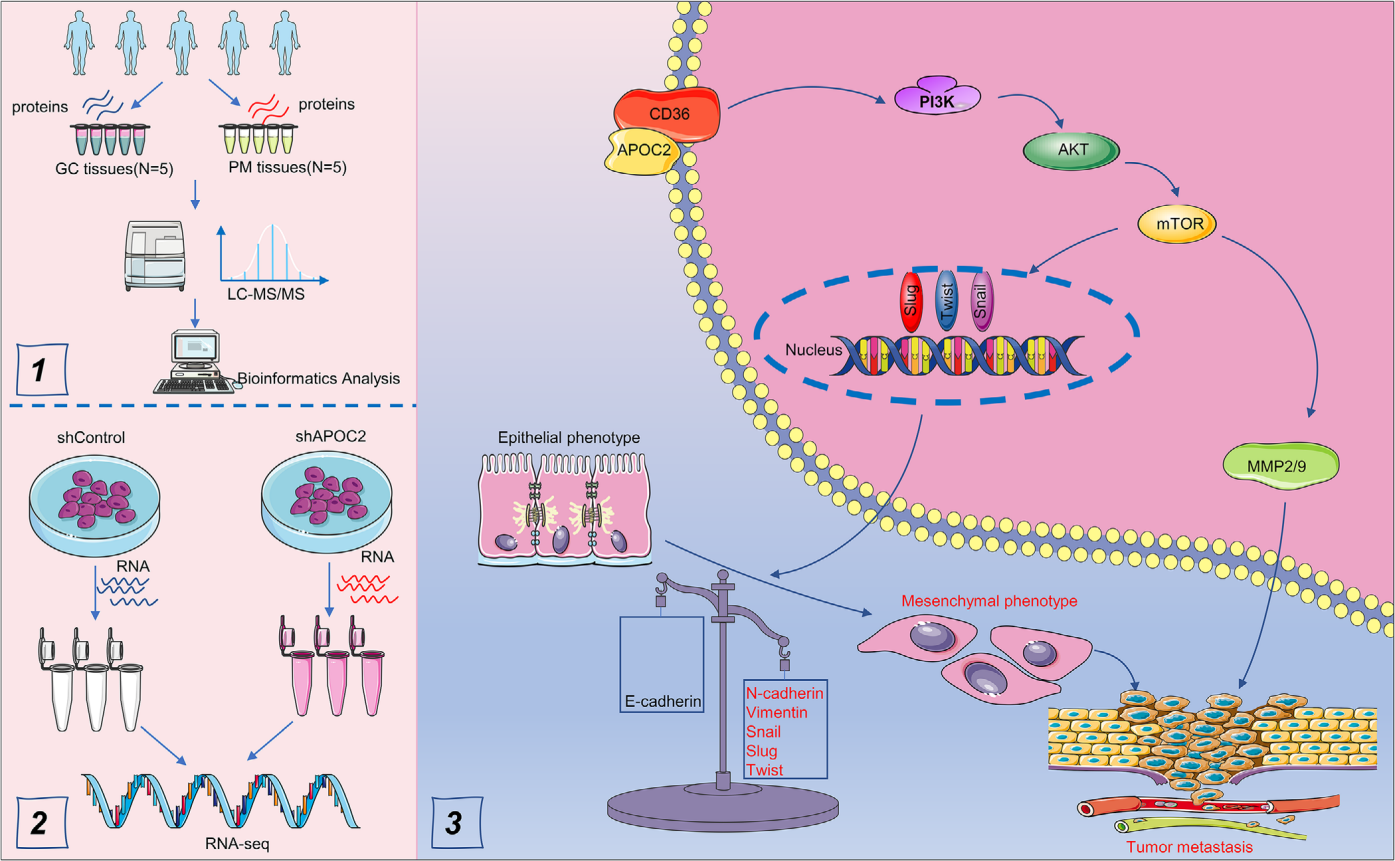
Schematic figure for the findings in the present study

CD36, a fatty acid translocase, is critical for the regulation of lipid metabolism in GC cells.^22^, ^23^, ^68^ Overexpression of CD36 results in elevated fatty acid uptake and could be a confirmatory marker for EMT to a more aggressive phenotype in various tumors,^69^, ^70^, ^71^ including GC.^21^ Although APOC2 has been reported to interact with CD36 and initiate downstream signaling in diseases,^11^, ^24^ the collaboration between APOC2 and CD36 needs to be further confirmed in GC. Our data demonstrated that both CD36 and APOC2 were indispensable for GC migration, invasion, and proliferation. It has also been indicated that CD36 promotes cancer progression by activating PI3K/AKT signaling.^31^, ^32^ Consistently, we proved that the interaction between CD36 and APOC2 was essential for the activation of PI3K/AKT/mTOR signaling to regulate EMT of GC cells (Figure 9). This finding provides a deeper understanding how APOC2 controls EMT to promote PM. Nevertheless, specific mechanism that governs APOC2 expression in GC needs to be further elucidated. We found that *APOC2* was hypomethylated and upregulated in GC. Relative mechanism of *APOC2* gene methylation in GC requires further exploration as well.

## 5 CONCLUSION

By integrating proteomic and transcriptomics profiles of GC and PM samples, we reported that APOC2 cooperated with CD36 to regulate EMT process via PI3K/AKT/mTOR signaling, which eventually promoted tumor progression and PM in GC.

## CONFLICT OF INTEREST

The authors declare that they have no known conflict of interest.

## ETHICS STATEMENT

The use of clinical samples and all animal procedures was approved by the Ethics Committee of Nanjing Drum Tower Hospital, the Affiliated Drum Tower Hospital of Nanjing University Medical School, and carried out in accordance with the principles of the Declaration of Helsinki.

## AUTHOR CONTRIBUTIONS

WG and SL conceived the study. CW, ZY, EX, and XS performed the experiments. CW and ZY collected the data. CW, XW, ZL, KC, HY, QY, and XX analyzed and interpreted the data. CW and SL wrote the manuscript. All authors read and approved the final manuscript.

## CONSENT FOR PUBLICATION

All contributing authors agree to the publication of this article.

## Supporting information

SUPPLEMENTAL MATERIAL

Figure S1 Analysis of all the identified proteins in GC tissues and PM tissues based on TMT‐labeled quantitative proteomics.

Figure S2 Identification of DEPs between GC tissue and PM tissue based on TMT‐labeled quantitative proteomics analysis.

Figure S3 Analysis of the biological functions of LIPL, SORC2, IBP6, CD36, and APOA in TCGA STAD data set.

Figure S4 LIPL, SORC2, IBP6, CD36, and APOA are upregulated in GC patients with PM.

Figure S5 Knockdown of LIPL and CD36 but not IBP, SOR, and APOA inhibits PI3K/AKT/mTOR signaling in GC.

Figure S6 *APOC2* was hypomethylated and upregulated in GC.

Figure S7 APOC2 induces potential proteins change in glycolysis and lipid metabolism pathway.

Figure S8 Overexpression of APOC2 promotes GC cell migration, invasion, and proliferation.

Figure S9 APOC2 cooperates with CD36 mediates EMT via PI3K/AKT/mTOR signaling to promote AGS cell migration, invasion, and proliferation.

Figure S10 APOC2 cooperates with CD36 mediates EMT via PI3K/AKT/mTOR signaling to promote BGC‐823 cell migration, invasion, and proliferation.

Figure S11 Knockdown *CD36* inhibits EMT via PI3K/AKT/mTOR signaling to suppress OE APOC2 stable BGC‐823 cell migration, invasion, and proliferation.

Figure S12 Knockdown *APOC2* inhibits EMT via PI3K/AKT/mTOR signaling to suppress OE CD36 stable AGS cell migration, invasion, and proliferation

Figure S13 Knockdown *APOC2* inhibits EMT via PI3K/AKT/mTOR signaling to suppress OE CD36 stable BGC‐823 cell migration, invasion, and proliferation.

Figure S14 APOC2 cooperates with CD36 to promote tumor progression in GC.

Table S1. A total of 7638 proteins were identified at 1% false discovery rate (FDR).

Table S2. Compared with the primary GC, 595 proteins in the PM tissues were upregulated among these DEPs.

Table S3. Compared with the primary GC, 1050 proteins in the PM tissues were downregulated among these DEPs.

Table S4. GO enrichment analysis of all identified protein‐specific information and visualization results.

Table S5. GO enrichment analysis for the DEPs.

Table S6. KOG analysis of all identified proteins.

Table S7. KOG analysis of the DEPs.

Table S8. KEGG enrichment analysis of all identified proteins.

Table S9. KEGG enrichment analysis of the DEPs.

Table S10. Subcellular localizations of the DEPs via bioinformatic tools (WoLF PSORT).

Table S11. GO_REGULATION_OF_LIPID_METABOLIC_PROCESS.

Table S12. Associations between serum APOC2 expression and clinical pathological characteristics in patients with GC

## Data Availability

The data generated during and/or analyzed during the current study are available from the corresponding author on reasonable request.
