# Supplementary material for: Apolipoprotein C‐II induces EMT to promote gastric cancer peritoneal metastasis via PI3K/AKT/mTOR pathway
Source: Clin Transl Med. 2021 Aug 9;11(8):e522. doi: 10.1002/ctm2.522 (PMC8351524; doi:10.1002/ctm2.522)
Supplement: Supplementary file 1 — SUPPLEMENTAL MATERIAL [file CTM2-11-e522-s005.docx]

**SUPPLEMENTAL MATERIAL:**

**TITLE**

Apolipoprotein C-II induces EMT to promote gastric cancer peritoneal metastasis via PI3K/AKT/mTOR pathway

**RUNNING TITLE**

APOC2 promotes peritoneal metastasis in GC

Chao Wang^1^^†^, Zhi Yang^2†^, En Xu^1†^, Xiaofei Shen^1†^, Xingzhou Wang^1^, Zijian Li^2^, Heng Yu^2^, Kai Chen^1^, Song Liu^1^*, Wenxian Guan^1^*

^1^Department of General Surgery, Nanjing Drum Tower Hospital, The Affiliated Hospital of Nanjing University Medical School, Nanjing 210008, China; ^2^ Department of General Surgery, Nanjing Drum Tower Hospital Clinical College of Nanjing Medical University, Nanjing 210008, China.

^†^These authors contributed equally to this work.

***Corresponding author:**

**Wenxian Guan,** Department of General Surgery, Nanjing Drum Tower Hospital, The Affiliated Hospital of Nanjing University Medical School, 321 Zhongshan Road, Nanjing, Jiangsu 210008, China; E-mail: guan_wenxian@sina.com.

**Song Liu,** Department of General Surgery, Nanjing Drum Tower Hospital, The Affiliated Hospital of Nanjing University Medical School, 321 Zhongshan Road, Nanjing, Jiangsu 210008, China; E-mail: medical.lis@gmail.com.

**SUPPLEMENTAL MATERIALS AND METHODS**

**1 Tandem Mass tag (TMT) quantification proteomic analysis**

GC tissues and corresponding PM tissues were sampled from five patients with GC and combined. The samples were prepared and the differentially expressed proteins (DEPs) were determined using Liquid chromatography–tandem mass spectrometry (LC-MS/MS)-based proteomic and bioinformatic analyses at the Beijing Genomics Institution (BGI). The main steps are as follows:

1.1 Protein Extraction

(1) Weigh the appropriate amount of sample, transfer it into a 2 mL centrifuge tube, add two steel beads, add 1XCocktail with appropriate amount of SDS L3 and EDTA, put on ice for 5 minutes, add DTT with the final concentration of 10 mM; (2) Use a grinder (frequency is 60HZ, time is 2 minutes) to crush the tissue, centrifuge at 25,000×g at 4°C for 15 minutes, and take the supernatant; (3) Add DTT with the final concentration of 10 mM, water bath at 56°C for 1 hour; (4) Add IAM at a final concentration of 55mM, and place in a dark room for 45 minutes; (5) Add cold acetone to the protein solution at a ratio of 1:5, place in a refrigerator at -20°C for 30 minutes, centrifuge at 25,000 × g at 4°C for 15 minutes, and discard the supernatant; (6) Air-dry precipitation, add lysis buffer without SDS L3, use a grinder (frequency is 60HZ, time is 2 minutes) to promote protein solubilization; (7) Centrifuge for 15 minutes at 25,000 × g at 4°C to take the supernatant, and the supernatant is the protein solution.

1.2 Quality Control of Protein Extraction

(1) Standard proteins (0.2 µg/µL BSA) 0, 2, 4, 6, 8, 10, 12, 14, 16, 18 µL were sequentially added to the 96-well microtiter plates A1 to A10, followed by the addition of pure water 20, 18, 16, 14, 12, 10, 8, 6, 4, 2 µL, and then 180 µL of Coomassie Brilliant Blue G-250 Quantitative Working Solution was added to each well. The OD595 was measured with a microplate reader, and a linear standard curve was drawn based on the OD595 and protein concentration. Diluted the protein solution to be tested several times, added 180 µL of the quantitative working solution to 20 µL of the protein solution, and read at OD595. The sample protein concentration was calculated from the standard curve and sample OD595. (2) Each 10 µg of protein solution was mixed with an appropriate amount of loading buffer, heated at 95°C for 5 minutes, centrifuged at 25,000 × g for 5 minutes, and the supernatant was loaded into a well of a 12% SDS polyacrylamide gel, 80V constant pressure electrophoresis for 30 minutes followed by 120V constant pressure electrophoresis for 120 minutes; After electrophoresis, the gel was stained and de-stained by a protein staining instrument for 10 minutes and the images were scanned.

1.3 Proteolysis

(1) Sampling: Took 100 µg (total amount of protein for digestion is adjusted according to theproject requirements) and added it to a 1.5 mL centrifuge tube; (2) Dilution of protein solution: If there is high concentration of urea or SDS in the protein solution, it should be diluted with 0.5M TEAB to make the final concentration of urea less than 2 M, and the final concentration of SDS is less than 0.1%; (3) According to Trypsin enzyme (µg): substrate protein (µg) = 1:20, enzyme solution was added, vortexed, centrifuged at low speed for 1 minute, and incubated at 37°C for 4 hours; (4) Took out the digested peptide liquid for desalting; (5) Freeze-dried the peptide liquid obtained after salt removal.

1.4 Peptide Labeling

(1) Each tube of TMT (0.8 mg) reagent was dissolved in 41 µL of acetonitrile and shaken for more than 1 minute to fully dissolve; (2) The peptide fragment after digestion and desalting was dissolved in 0.1 M TEAB solution to make the peptide concentration 3.74 µg/µL, and shook for more than 1 minute to fully dissolve; (3) Took 100μg of peptide (26.7 µL) and 41 µL of TMT reagent for rapid mixing, shook and centrifuged, and then checked the pH value between 7.0-8.0; (4) The same procedure was used for each channel of the TMT reagent and allowed to stand at room temperature for 2 hours to obtain sufficient labeling.

1.5 Peptide Fractionation

The Shimadzu LC-20AB liquid phase system was used, and the separation column was a 5um 4.6 × 250mm Gemini C18 column for liquid phase separation of the sample. The dried peptide samples were reconstituted with mobile phase A (5% ACN pH 9.8) and injected, eluting at a flow rate of 1mL/min by following gradients: 5% mobile phase B (95% ACN, pH 9.8) for 10 minutes. 5% to 35% mobile phase B for 40 minutes, 35% to 95% mobile phase B for 1 minute, mobile phase B for 3 minutes, and 5% mobile phase B for 10 minutes. The elution peak was monitored at a wavelength of 214 nm and one component was collected per minute, and the samples were combined according to the chromatographic elution peak map to obtain 20 components, which were then freeze-dried.

1.6 HPLC

The dried peptide samples were reconstituted with mobile phase A (2% ACN, 0.1% FA), centrifuged at 20,000 × g for 10 minutes, and the supernatant was taken for injection. Separation was performed by Thermo UltiMate 3000 UHPLC. The sample was first enriched in trap column and desalted, and then entered a self-packed C18 column (75 µm internal diameter, 3 µm column size, 25 cm column length) and separated at a flow rate of 300 nL/min by the following effective gradient: 0~5 minutes, 5% mobile phase B (98% ACN, 0.1% FA); 5~45 minutes, mobile phase B linearly increased from 5% to 25%; 45~50minutes, mobile phase B increased from 25% to 35%; 50~52 minutes, mobile phase B rose from 35% to 80%; 52~54 minutes, 80% mobile phase B; 54~60 minutes, 5% mobile phase B. The nanoliter liquid phase separation end was directly connected to the mass spectrometer.

1.7 Mass Spectrometry Detection

The peptides separated by liquid phase chromatography were ionized by a nanoESI source and then passed to a tandem mass spectrometer Q-Exactive HF X (Thermo Fisher Scientific, San Jose, CA) for DDA (Data Dependent Acquisition) mode detection. The main parameters were set: ion source voltage was set to 1.9kV, MS1 scanning range was 350~1,500 m/z; resolution was set to 60,000; MS2 starting m/z was fixed at 100; resolution was 15,000. The ion screening conditions for MS2 fragmentation: charge 2+ to 6+, and the top 20 parent ions with the peak intensity exceeding 10,000. The ion fragmentation mode was HCD, and the fragment ions were detected in Orbitrap. The dynamic exclusion time was set to 30 seconds. The AGC was set to: MS1 3E6, MS2 1E5.

**2 RNA sequencing analysis**

The mRNA of the AGS cells knocked down for APOC2 expression and untreated AGS cells was extracted and sent to Shanghai Genechem Co., Ltd for mRNA sequencing. The main steps are as follows:

2.1 RNA quantification and qualification

(1) RNA degradation and contamination were monitored on 1% agarose gels. (2) RNA purity was checked using the NanoPhotometer® spectrophotometer (IMPLEN, CA, USA). (3) RNA integrity was assessed using the RNA Nano 6000 Assay Kit of the Bioanalyzer 2100 system (Agilent Technologies, CA, USA).

2.2 Library preparation for Transcriptome sequencing

A total amount of 1 µg RNA per sample was used as input material for the RNA sample preparations. Sequencing libraries were generated using NEBNext® UltraTM RNA Library Prep Kit for Illumina® (NEB, USA) following manufacturer’s recommendations and index codes were added to attribute sequences to each sample. Briefly, mRNA was purified from total RNA using poly-T oligo-attached magnetic beads. Fragmentation was carried out using divalent cations under elevated temperature in NEBNext First Strand Synthesis Reaction Buffer (5×). First strand cDNA was synthesized using random hexamer primer and M-MuLV Reverse Transcriptase (RNase H-). Second strand cDNA synthesis was subsequently performed using DNA Polymerase I and RNase H. Remaining overhangs were converted into blunt ends via exonuclease/polymerase activities. After adenylation of 3’ ends of DNA fragments, NEBNext Adaptor with hairpin loop structure were ligated to prepare for hybridization. In order to select cDNA fragments of preferentially 250~300 bp in length, the library fragments were purified with AMPure XP system (Beckman Coulter, Beverly, USA). Then 3 µl USER Enzyme (NEB, USA) was used with size-selected, adaptor-ligated cDNA at 37°C for 15 m in followed by 5 min at 95 °Cbefore PCR. Then PCR was performed with Phusion High -Fidelity DNA polymerase, Universal PCR primers and Index (X) Primer. At last, PCR products were purified (AMPure XP system) and library quality was assessed on the Agilent Bioanalyzer 2100 system.

2.3 Clustering and sequencing (Genechem Experimental Department)

The clustering of the index-coded samples was performed on a cBot Cluster Generation System using TruSeq PE Cluster Kit v3-cBot-HS (Illumia) according to the manufacturer’s instructions. After cluster generation, the library preparations were sequenced on an Illumina Novaseq platform and 150 bp paired-end reads were generated.

**3 Western blot**

Proteins were extracted from cell samples and tissues using Radioimmunoprecipitation assay (RIPA) lysis buffer as previously described^1^. In short, proteins were separated by SDS-PAGE gel transferred onto a PVDF membrane. Primary antibody information is as follows: LIPL (Affinity, DF12534), SORC2( ProteinTech Group, #55063-1-AP), IBP6(Affinity, DF8959), APOA(Affinity, BF0429), GLUT1 (Affinity, AF0173), HK2 (Affinity, DF6176), PKM2(Affinity, AF5234), LDHA (Affinity,DF6280), LDLR(Affinity, DF7696), LRP1(Affinity,DF2935).

**4 Small interfering RNA (siRNA) assay**

Cells were treated with LIPL-siRNAs (siRNA#1, 5’-GCCGCCGCCGACCAAAGAATT-3’,5’-UUCUUUGGUCGGCGGCGGCTT-3’; siRNA#2, 5’-CCAUACCAAUCAGGCCUUUTT-3’, 5’-AAAGGCCUGAUUGGUAUGGTT-3’; siRNA#3, 5’-GGUGGAGCAGUCCCGGCUUTT-3’, 5’-AAGCCGGGACUGCUCCACCTT-3’); SORC2-siRNAs (siRNA#1,5’-CUGCCAACUUCUGGUUUAATT-3’, 5’-UUAAACCAGAAGUUGGCAGTT-3’; siRNA#2, 5’-GGUGGAAUAUAAAGAAGAATT-3’, 5’-UUCUUCUUUAUAUUCCACCTT’; siRNA#3, 5’-GAGCCUUCAUCCUCUACAATT-3’, 5’-UUGUAGAGGAUGAAGGCUCTT-3’); IBP6-siRNAs (siRNA#1,5’-GCUGUUGCAGAGGAGAAUCTT-3’, 5’-GAUUCUCCUCUGCAACAGCTT-3’; siRNA#2, 5’-GGUGUCCAAGACACUGAGATT-3’, 5’-UCUCAGUGUCUUGGACACCTT’; siRNA#3, 5’-GCCCAAUUGUGACCAUCGATT-3’, 5’-UCGAUGGUCACAAUUGGGCTT-3’); APOA-siRNAs (siRNA#1,5’-GCAGCUCCUUAUUGUUAUATT-3’, 5’-UAUAACAAUAAGGAGCUGCTT-3’; siRNA#2, 5’-GCAGCUCCUUAUUGUUAUATT-3’, 5’-UAUAACAAUAAGGAGCUGCTT’; siRNA#3, 5’-GCAGCUCCUUAUUGUUAUATT-3’, 5’-UAUAACAAUAAGGAGCUGCTT-3’) or control-siRNA. These siRNAs were provided by Shanghai GenePharma Company (Shanghai, China). 2×10^5^ GC cells per well plated into a six-well plate were treated with siRNA (1-2 µg) encapsulated by the interferin reagent (Polyplus, USA) based on the protocol. Western blot was used to evaluated the knockdown efficiency.

**5 Next generation sequencing- (NGS-) based DNA methylation profiling**

The normal gastric mucosal tissues (n=4), primary gastric cancer tissues(n=4), gastric cancer peritoneal metastasis tissues (n=4) were prepared and *APOC2* gene methylation were detected via NGS-based DNA methylation profiling at Tsingke Biotechnology Co., Ltd. (Nanjing, China). Referring to previous research on APOC2 gene methylation detection^2^, we performed NGS-based DNA methylation profiling for the *APOC2* gene region (hg19_dna range=chr19:45447831-45449574) including the CpG site (MAPINFO:45449297). In brief, HiPure Tissue DNA Mini kit (Magen, Guangzhou, China) was used to extract DNA from tissues. Then DNA was sheared into ~300*-*bp fragments using sonication. Next, DNA was purified with 2× magnetic beads and was modified using bisulfite conversion kit (EZ DNA Methylation-Gold Kit™; ZYMO Research Corp.). Then performing primer specific PCR with TaKaRa's (Kyoto, Japan) bisulfite specific enzyme and using TaKaRa's (Kyoto, Japan) common Taq enzyme for the second round of PCR amplification. Finally, after Gel electrophoresis detection, sequencing after purification with 1.2× magnetic beads. Primer sequence:

APOC2-1NF: AGTTCAGACGTGTGCTCTTCCGATCTGGTTGTTTTTGGGTTATCGGGAG,

APOC2-1NR: TCCCTACACGACGCTCTTCCGATCTACACCCTCACCAAAACCATTACCAC;

APOC2-1NF2: AGTTCAGACGTGTGCTCTTCCGATCTAGTATGTTAGTTAGAGGTTTAGGAAGG,

APOC2-1NR2: TCCCTACACGACGCTCTTCCGATCTCACCACCCAACCCAACACACCCT;

APOC2-3NF: AGTTCAGACGTGTGCTCTTCCGATCTTGGGGATAAGGATTAGGGTTAAAATG,

APOC2-3NR: TCCCTACACGACGCTCTTCCGATCTTCTTTTTATTTTACCCCCAAAAAATCTC;

APOC2-4NF: AGTTCAGACGTGTGCTCTTCCGATCTATGAGTAGAAGAGGTGATATTTGATG,

APOC2-4NR: TCCCTACACGACGCTCTTCCGATCTTCATAAAAACCTACTAACTCCAC;

APOC2-4NF2: AGTTCAGACGTGTGCTCTTCCGATCTGGTGGAGTTAGTAGGTTTTTATG,

APOC2-4NR2: TCCCTACACGACGCTCTTCCGATCTACCTTCTCTCAAATAACAAAAAACCC;

APOC2-5NF: AGTTCAGACGTGTGCTCTTCCGATCTAGGGTTTTTTGTTATTTGAGAGAAG,

APOC2-5NR: TCCCTACACGACGCTCTTCCGATCTACTCTAACAAACTATCCTCACCA.

**6 Public genomic data**

The transcriptome sequencing data of TCGA Stomach Adenocarcinoma cohort (TCGA-STAD, 375 cancerous tissues and 32 paracancerous tissues) was downloaded from The Cancer Genome Atlas (TCGA; https://portal.gdc.cancer.gov/). The DNA methylation data of 27 paracancerous tissues and 443 cancerous tissues of TCGA-STAD was downloaded from UCSC Xena (https://xenabrowser.net/datapages/).

**7 Collection of gene sets**

For Gene Ontology (GO) analysis, gene sets were downloaded from Gene Ontology Resource (http://geneontology.org/). For Kyoto Encyclopedia of Genes and Genomes (KEGG) pathway analysis, gene sets were downloaded from KEGG PATHWAY Database (https://www.genome.jp/kegg/pathway.html). For gene set enrichment analysis (GSEA) and gene set variation analysis (GSVA), gene sets were downloaded from The Molecular Signatures Database (mSigDB, https://www.gsea msigdb.org/gsea/index.jsp).

**8 GO and KEGG analysis**

The conversion of protein ID and gene symbol were completed by the R package org.Hs.eg.db (version 3.11.1). GO and KEGG analysis of specific proteins were performed using R package clusterProfiler (version 3.16.1). Gene sets with p value <0.05 and adjusted p value <0.05 were considered statistically significant.

**9 GSEA**

Gene sets were retrieved from mSigDB. The input data was pre-ranked using the log2-FoldChange. GSEA was carried out to determine the biological functions and pathways of genes. R package clusterProfiler (version 3.16.1) was used to perform the analysis and plot the results. The enrichment results that satisfied two conditions (p-value < 0.05 and q-value < 0.05 indicated statistical significance and were visualized by the R package enrichplot (version 1.8.1).

**10 GSVA**

All gene ontology of biology process gene sets (“c5.all.v7.0.symbols.gmt”) was downloaded from mSigDB. The R package GSVA (version 1.36.2) was used to score all the gene sets for each sample to represent the active level of each biological process. The normalized GSVA score was submitted to further analysis.

**SUPPLEMENTARY FIGURE LEGEND**

**Figure S1 Analysis of all the identified proteins in GC tissues and PM tissues based on TMT-labeled quantitative proteomics.**

(A) Functional GO enrichment analysis of all the identified proteins. (B) The potential functions of all the identified proteins annotated by KOG. (C) KEGG enrichment analysis to characterize the biological functions of all the identified proteins.

**Figure S2** **Identification of DEPs between GC tissue and PM tissue based on TMT-labeled quantitative proteomics analysis.**

(A) GO classification of up- and downregulated DEPs. (B) KEGG pathway classification of up- and downregulated DEPs. (G) KEGG enrichment analysis to characterize the biological functions of the DEPs.

**Figure S3 Analysis of the biological functions of** **LIPL, SORC2, IBP6, CD36, and APOA in TCGA STAD dataset.**

(A-E) GSVA was used to evaluate the functional mechanism of LIPL (A), SORC2 (B), IBP6(C), CD36(D), and APOA (E) in GC tissues (n=375) according to TCGA STAD dataset.

**Figure S4 LIPL, SORC2, IBP6, CD36, and APOA are upregulated in GC patients with PM.**

(A) Proteomics data showed that LIPL, SORC2, IBP6, CD36, and APOA highly expressed in PM tissues.

(B) western blot was used to detect protein expression of LIPL, SORC2, IBP6, CD36, and APOA from five patients with GC in tumor tissues and PM tissues. (C-G) Kaplan−Meier curves of *LIPL, SORC2, IBP6, CD36,* and *APOA* genes for overall survival were plotted. Latest RNA-sequencing data (HTSeq-FPKM) of TCGA-STAD cohort (n=375 for tumor samples) was downloaded through the R package TCGAbiolinks (version 2.16.4). The best cut off value of each gene was achieved utilizing the “surv_cutpoint” function of the R package survminer (version 0.4.8) according to their relationship with the patients’ survival probability with the maximum rank statistic, which was further used to group the TCGA-STAD cohort into high expression and low expression groups for each gene. Data represent mean ± SD, **P* < 0.05, based on Student’s t-test.

**Figure S5** **Knockdown of LIPL and CD36 but not IBP, SOR, and APOA inhibit PI3K/AKT/mTOR signaling in GC.**

(A, D, G, J and M) Western blot were used to assess the efficiency of knockdown of *LIPL*(A)*, SORC2*(D)*, IBP6*(G)*, CD36*(J) and *APOA*(M) in AGS and BGC-823 cells, respectively. (B, C, E, F, H, I, K, L, N and O) Western blot analysis of the protein levels of E-cadherin, N-cadherin, vimentin, Snail, Slug and Twist1 in AGS and BGC-823 cells transfected with control siRNA, siLIPL#3, siSORC2#3, siIBP#3, control shRNA, shCD36#3 and siAPOA#2. Data are shown as mean ± SD; ns: no significant difference; **P* < 0.05, ***P* < 0.01, ****P* < 0.001, based on Student’s t-test.

**Figure S6 *APOC2* was hypomethylated and upregulated in GC.**

(A) The relative expression of *APOC2* mRNA in 375 cases compared with 32 normal tissues in the TCGA STAD dataset. (B) Comparisons of *APOC2* methylation beta values between 27 paracancerous tissues and 443 GC tissues. The DNA methylation data was downloaded from UCSC Xena. (C) NGS-based DNA methylation profiling for the *APOC2* gene region (hg19_dna range=chr19:45447831-45449574) including the CpG site (MAPINFO:45449297). (D) RT-qPCR was used to detect mRNA levels of *APOC2* from eight patients with GC in tumor tissues, adjacent non-cancerous gastric tissues (ANTs), and PM tissues. (E) IHC staining analysis of APOC2 protein in GC tissues (n=111) and ANTs (n=111). Data are shown as mean ± SD; ns: no significant difference; **P* < 0.05, ***P* < 0.01, ****P* < 0.001, *****P* < 0.0001, based on Student’s t-test.

**Figure S7 APOC2 induces potential proteins change in glycolysis and lipid metabolism pathway.**

(A) Western blot was used to assess the efficiency of lentiviral knockdown of *APOC2* in AGS and BGC-823 cells, respectively. (B) Western blot was used to assess the efficiency of lentiviral overexpression of *APOC2* in AGS and BGC-823 cells, respectively. (C) Western blot analysis of the protein levels of GLUT1, HK2, PKM2, LDHA, LIPL, LDLR and LRP1 in AGS and BGC-823 cells transfected with Lenti-shAPOC2#1 and Lenti-shControl. Data are shown as mean ± SD; ns: no significant difference; **P* < 0.05, ***P* < 0.01, ****P* < 0.001, based on Student’s t-test.

**Figure S8 Overexpression of APOC2 promotes GC cell migration, invasion and proliferation.**

(A-C) Wound healing (A), Transwell (B), and colony formation (C) assays were performed to detect the migration, invasion, and proliferation ability of GC cells transfected with Lenti-oeAPOC2 and Lenti-oeControl, as well as those of cells treated with or without LY290042. Data represent mean ± SD, **P* < 0.05, ***P* < 0.01, ****P* < 0.001, based on Student’s t-test.

**Figure S9 APOC2 cooperates with CD36 mediates EMT via PI3K/AKT/mTOR signaling to promote AGS cell migration, invasion, and proliferation.**

(A) western blot was used to assess the efficiency of Lentiviral overexpression of CD36 in AGS and BGC-823 cells, respectively. (B) Western blot was used to assess the efficiency of lentiviral knockdown of CD36 in AGS and BGC-823 cells, respectively. (C-E) Effects of overexpression of *APOC2,* *CD36,* and both on cell migration, invasion and proliferation in AGS cells. (F) Western blot analysis of the protein levels of p-PI3K, p-AKT, p-mTOR, E-cadherin, N-cadherin, vimentin, Snail, Slug, Twist1, MMP-2 and MMP-9 in AGS OE Ctrl, OE APOC2, OE CD36 and OE APOC2+OECD36 cells. Data are shown as mean ± SD; ns: no significant difference; **P* < 0.05, ***P* < 0.01, ****P* < 0.001, *****P* < 0.0001, based on Student’s t-test.

**Figure S10 APOC2 cooperates with CD36 mediates EMT via PI3K/AKT/mTOR signaling to promote BGC-823 cell migration, invasion, and proliferation.**

(A-C) Effects of overexpression of *APOC2*, *CD36*, and both on cell migration, invasion and proliferation in BGC-823 cells. (D) Western blot analysis of the protein levels of p-PI3K, p-AKT, p-mTOR, E-cadherin, N-cadherin, vimentin, Snail, Slug, Twist1, MMP-2 and MMP-9 in BGC-823 OE Ctrl, OE APOC2, OE CD36, and OE APOC2+OECD36 cells. Data are shown as mean ± SD; ns: no significant difference; **P* < 0.05, ***P* < 0.01, ****P* < 0.001, *****P* < 0.0001, based on Student’s t-test.

**Figure S11 Knockdown *CD36* inhibits EMT via PI3K/AKT/mTOR signaling to suppress OE APOC2 stable BGC-823 cell migration, invasion, and proliferation.**

(A-C) The effect of knockdown *CD36* on the migration, invasion, proliferation of OE APOC2 BGC-823 stable cells. (D) The levels of p-PI3K, p-AKT, p-mTOR, E cadherin, N-cadherin, vimentin, Snail, Slug, Twist1, MMP-2 and MMP-9 proteins detected by western blot in OE APOC2 BGC-823 cells transduced with shCD36 lentiviral particles. Data are shown as mean ± SD; ns: no significant difference; **P* < 0.05, ***P* < 0.01, ****P* < 0.001, *****P* < 0.0001, based on Student’s t-test.

**Figure S12 Knockdown *APOC2* inhibits EMT via PI3K/AKT/mTOR signaling to suppress OE CD36 stable AGS cell migration, invasion, and proliferation**

(A-C) The effect of knockdown *APOC2* on the migration, invasion, proliferation of OE CD36 AGS stable cells. (D) The levels of p-PI3K, p-AKT, p-mTOR, E cadherin, N-cadherin, vimentin, Snail, Slug, Twist1, MMP-2 and MMP-9 proteins detected by western blot in OE CD36 BGC-823 cells transduced with shAPOC2 lentiviral particles. Data are shown as mean ± SD; ns: no significant difference; **P* < 0.05, ***P* < 0.01, ****P* < 0.001, based on Student’s t-test.

**Figure S13 Knockdown *APOC2* inhibits EMT via PI3K/AKT/mTOR signaling to suppress OE CD36 stable BGC-823 cell migration, invasion, and proliferation.**

(A-C) The effect of knockdown *APOC2* on the migration, invasion, proliferation of OE CD36 BGC-823 stable cells. (D) The levels of p-PI3K, p-AKT, p-mTOR, E cadherin, N-cadherin, vimentin, Snail, Slug, Twist1, MMP-2 and MMP-9 proteins detected by Western blot in OE CD36 BGC-823 cells transduced with shAPOC2 lentiviral particles. Data are shown as mean ± SD; ns: no significant difference; **P* < 0.05, ***P* < 0.01, ****P* < 0.001, based on Student’s t-test.

**Figure S14 APOC2 cooperates with CD36 to** **promote** **tumor progression in GC.**

(A) Photograph of subcutaneous tumors excised from nude mice injected with AGS OE APOC2/shCtrol, AGS OE APOC2/shCD36, AGS OE CD36/shCtrol and AGS OE CD36/shAPOC2 cells, respectively (n = 6 per group). (B-D) Tumors were observed and recorded by tumor volume (B and C) and tumor weight(D). Data are shown as mean ± SD; ns: no significant difference; *****P* < 0.0001, based on Student’s t-test.

**REFERENCES**

1. Wang C, Zhang C, Li X, et al. CPT1A-mediated succinylation of S100A10 increases human gastric cancer invasion. *J Cell Mol Med.* 2019;23(1):293-305.

2. Zhang T, Yang J, Vaikari VP, et al. Apolipoprotein C2 - CD36 Promotes Leukemia Growth and Presents a Targetable Axis in Acute Myeloid Leukemia. *Blood Cancer Discovery.* 2020;1(2):198-213.
