# Supplementary figures and images for: Apolipoprotein C‐II induces EMT to promote gastric cancer peritoneal metastasis via PI3K/AKT/mTOR pathway
Source: Clin Transl Med. 2021 Aug 9;11(8):e522. doi: 10.1002/ctm2.522 (PMC8351524; doi:10.1002/ctm2.522)

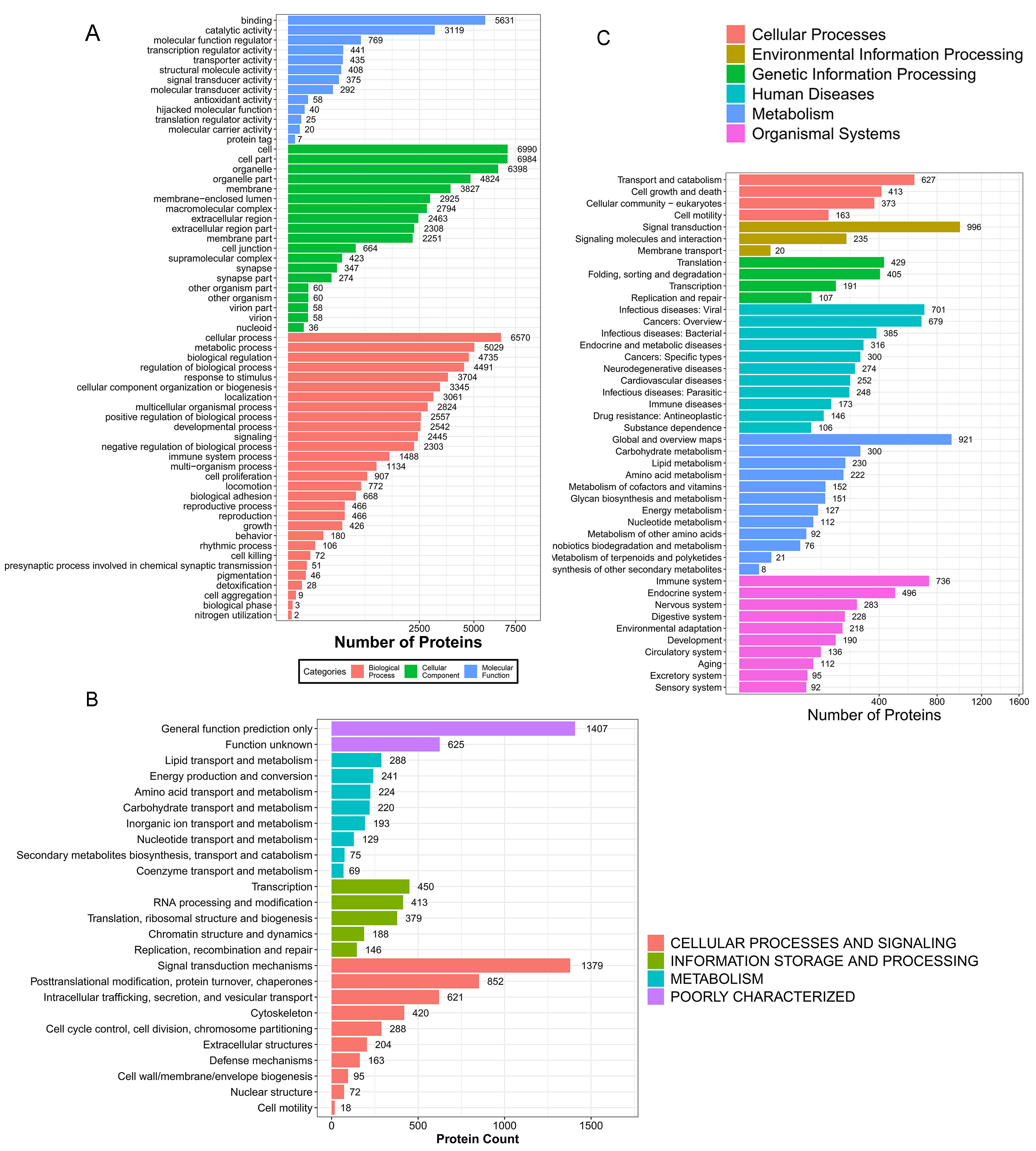

Supplement: Supplementary file 2 — Figure S1 Analysis of all the identified proteins in GC tissues and PM tissues based on TMT‐labeled quantitative proteomics. [file CTM2-11-e522-s008.tif]

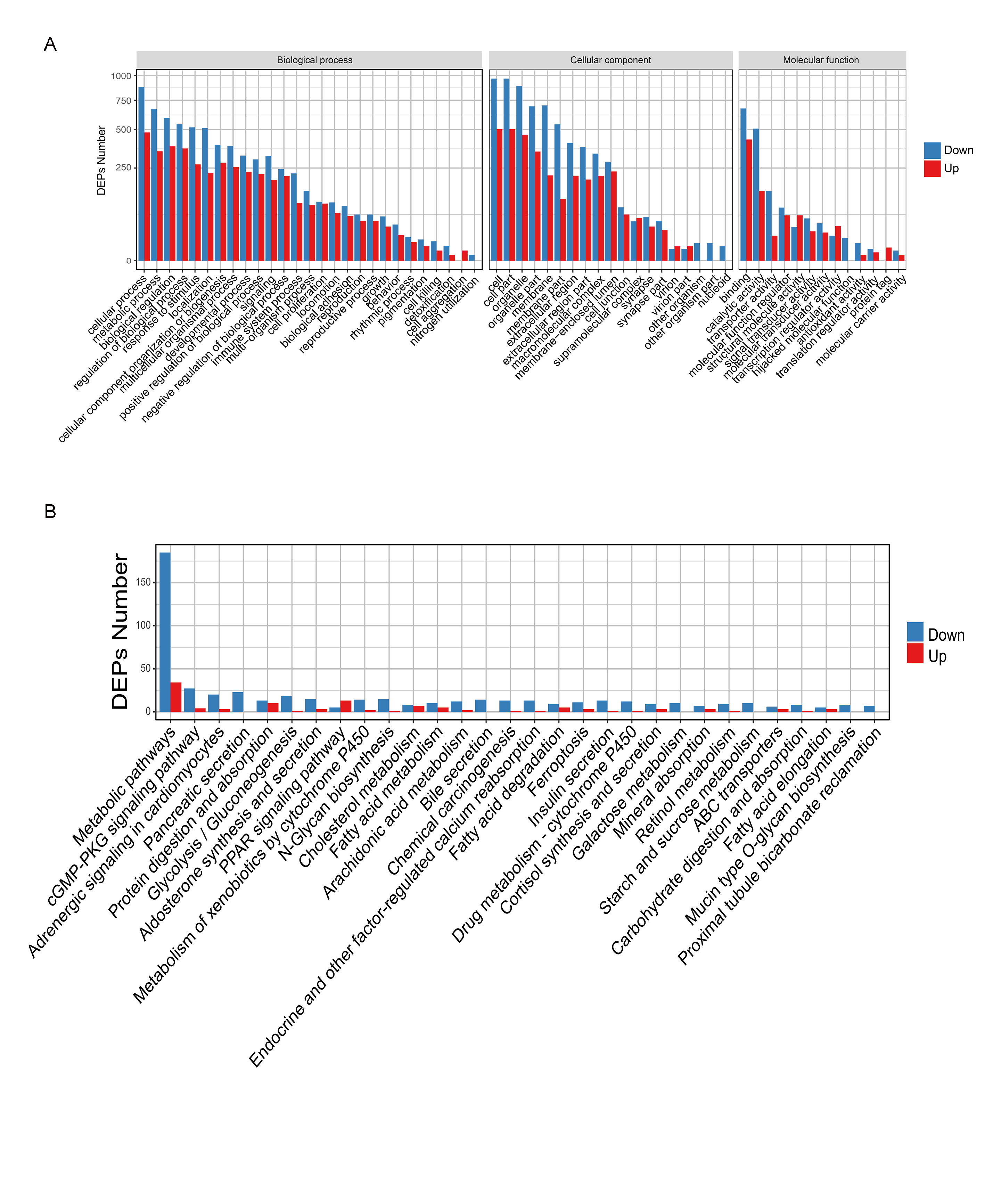

Supplement: Supplementary file 3 — Figure S2 Identification of DEPs between GC tissue and PM tissue based on TMT‐labeled quantitative proteomics analysis. [file CTM2-11-e522-s012.tif]

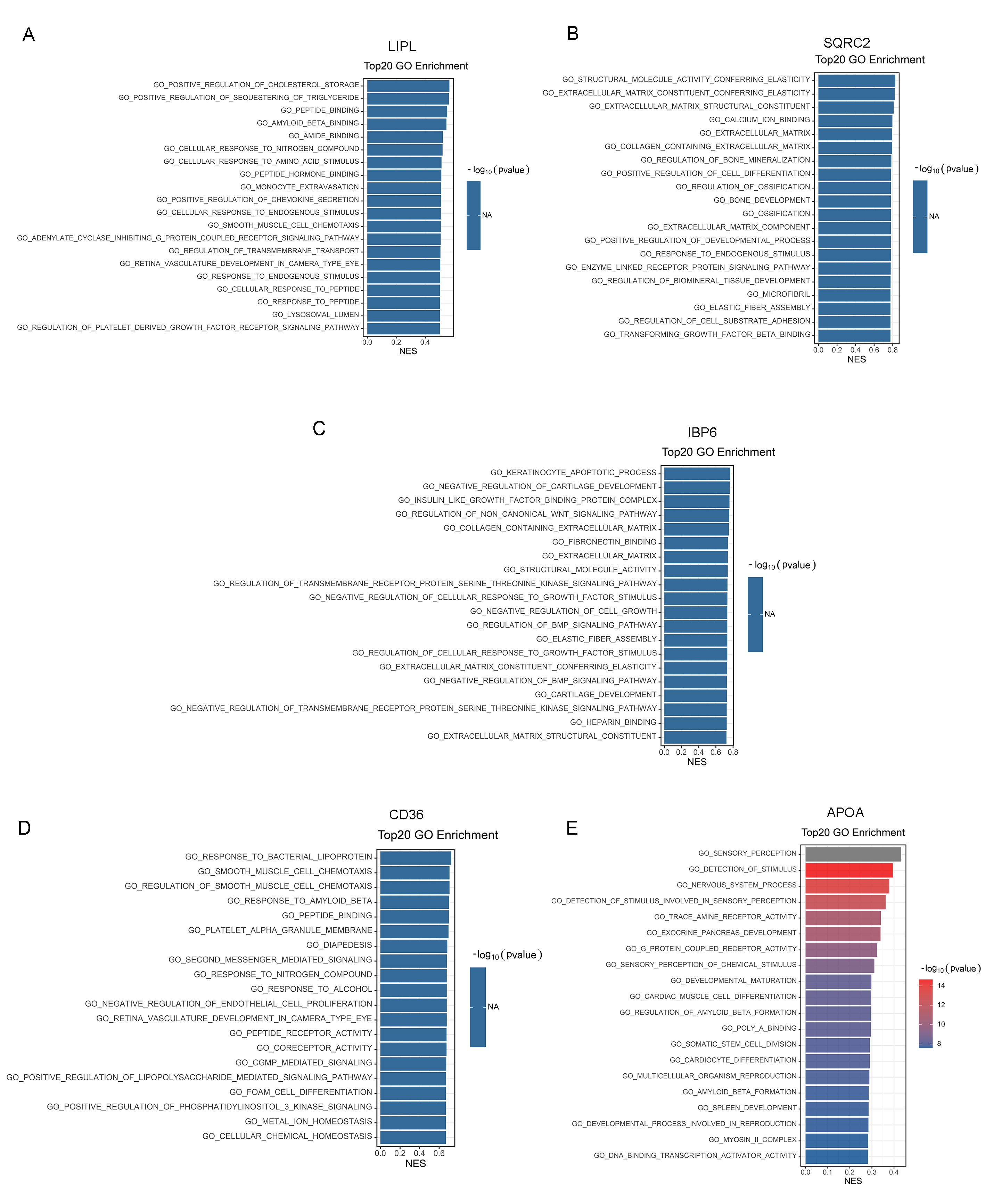

Supplement: Supplementary file 4 — Figure S3 Analysis of the biological functions of LIPL, SORC2, IBP6, CD36, and APOA in TCGA STAD data set. [file CTM2-11-e522-s023.tif]

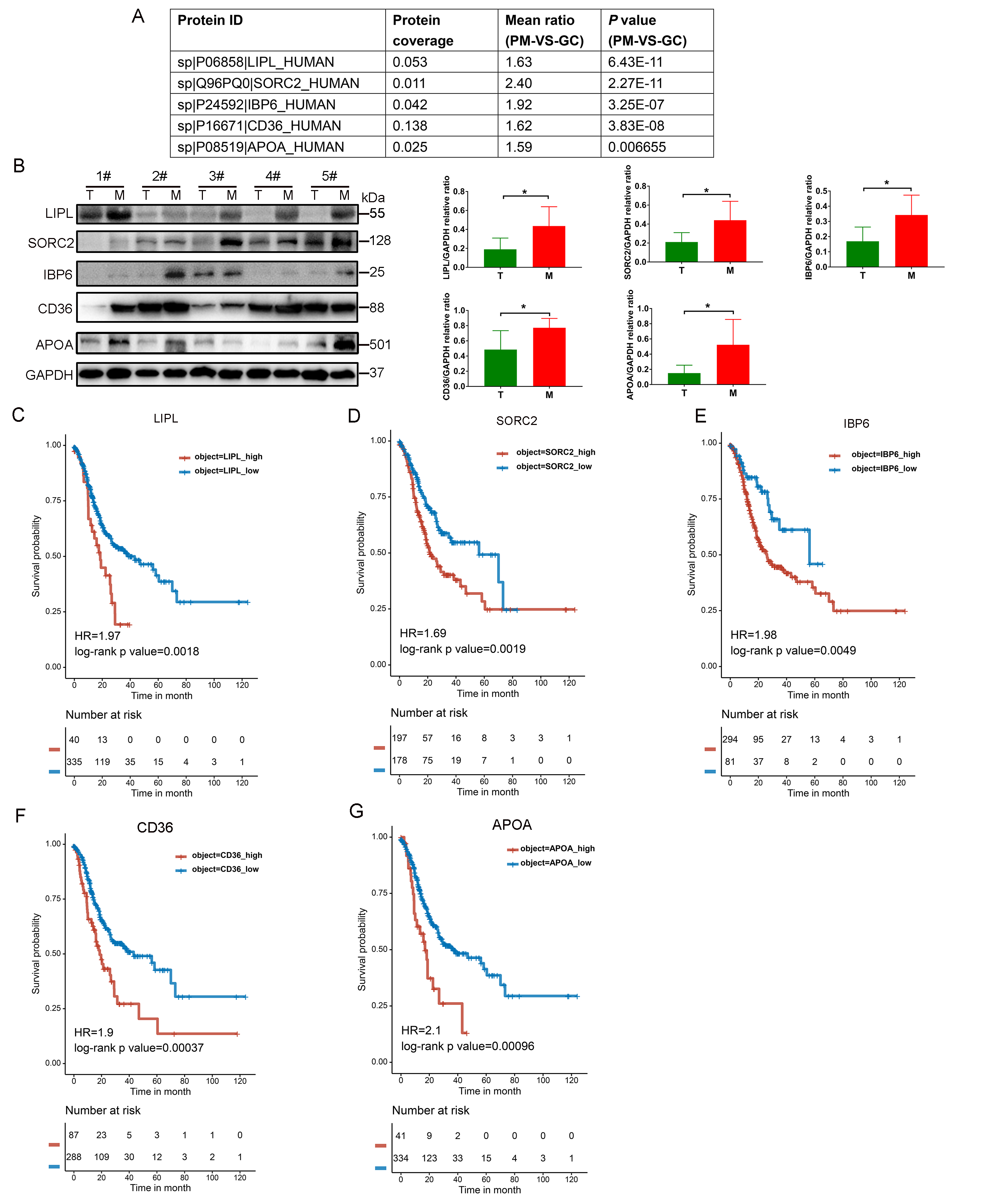

Supplement: Supplementary file 5 — Figure S4 LIPL, SORC2, IBP6, CD36, and APOA are upregulated in GC patients with PM. [file CTM2-11-e522-s002.tif]

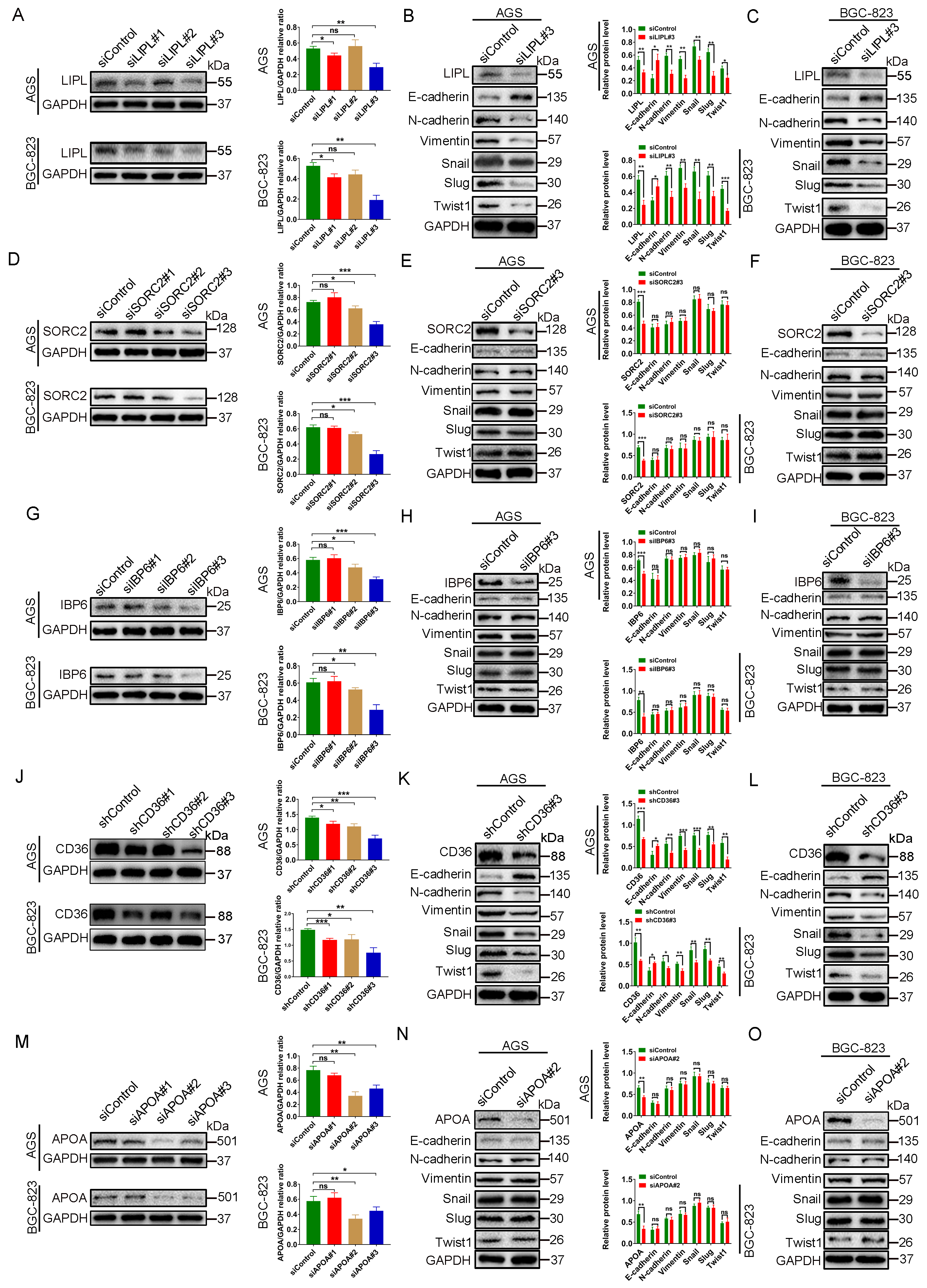

Supplement: Supplementary file 6 — Figure S5 Knockdown of LIPL and CD36 but not IBP, SOR, and APOA inhibits PI3K/AKT/mTOR signaling in GC. [file CTM2-11-e522-s019.tif]

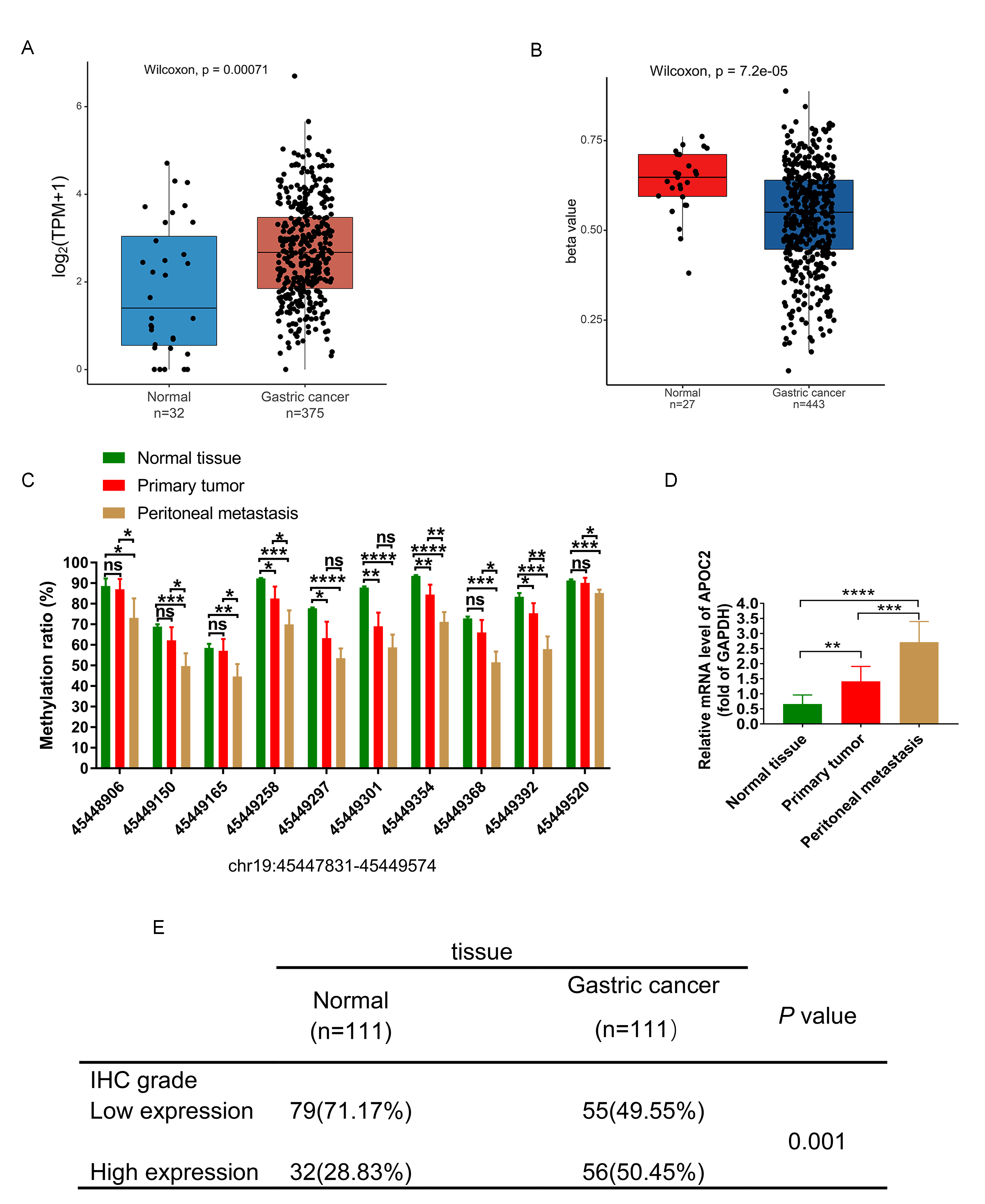

Supplement: Supplementary file 7 — Figure S6 APOC2 was hypomethylated and upregulated in GC. [file CTM2-11-e522-s009.tif]

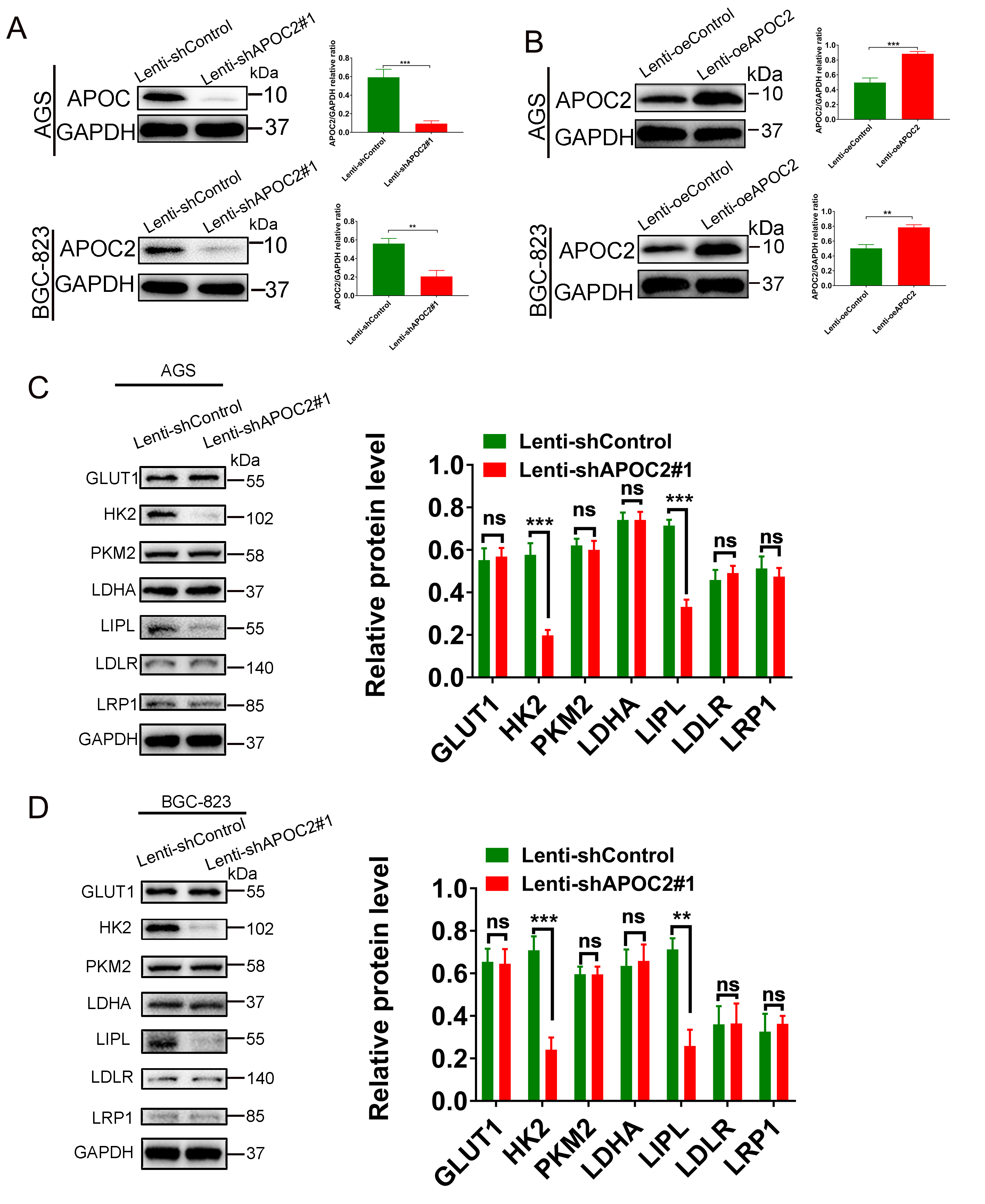

Supplement: Supplementary file 8 — Figure S7 APOC2 induces potential proteins change in glycolysis and lipid metabolism pathway. [file CTM2-11-e522-s003.tif]

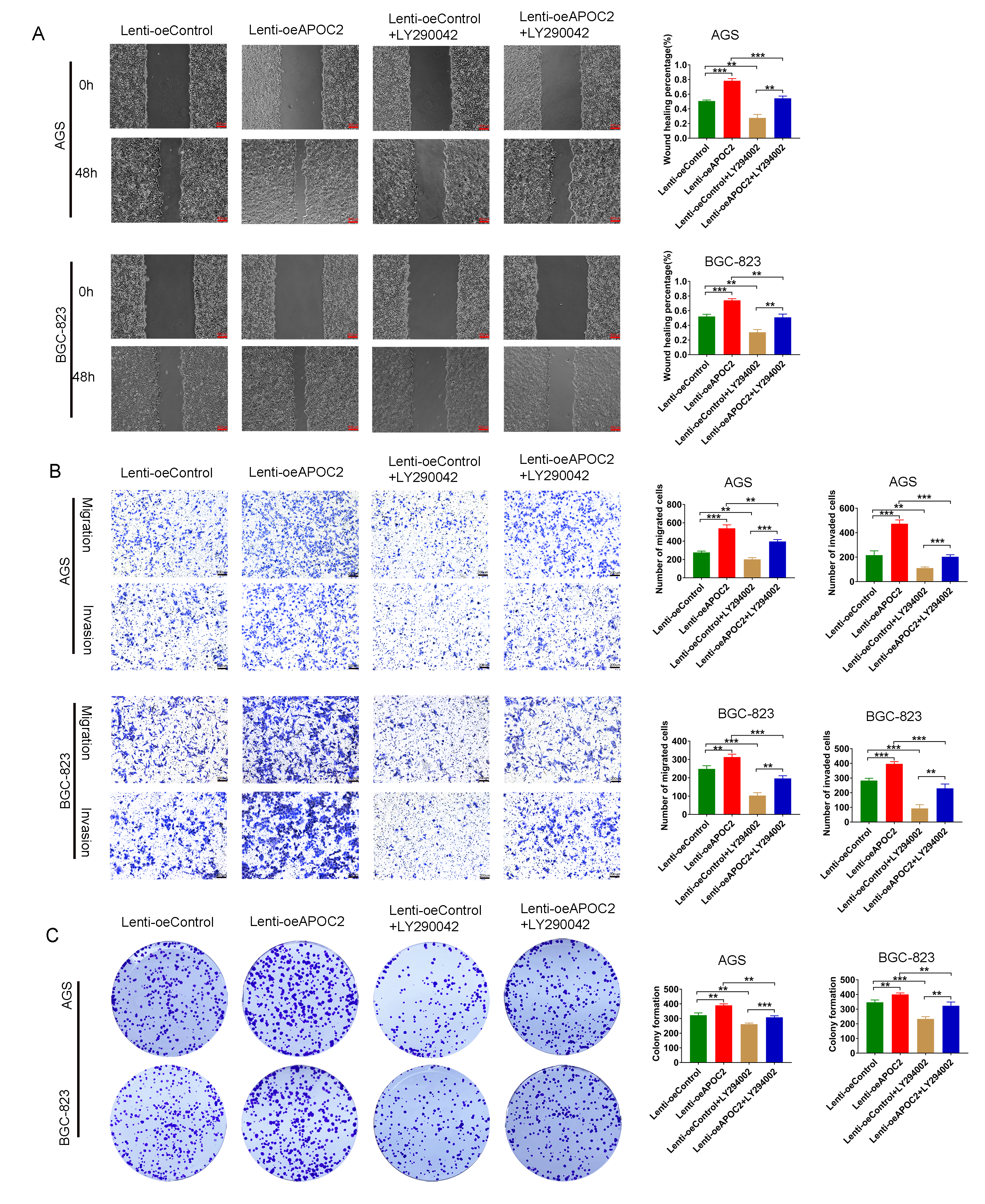

Supplement: Supplementary file 9 — Figure S8 Overexpression of APOC2 promotes GC cell migration, invasion, and proliferation. [file CTM2-11-e522-s004.tif]

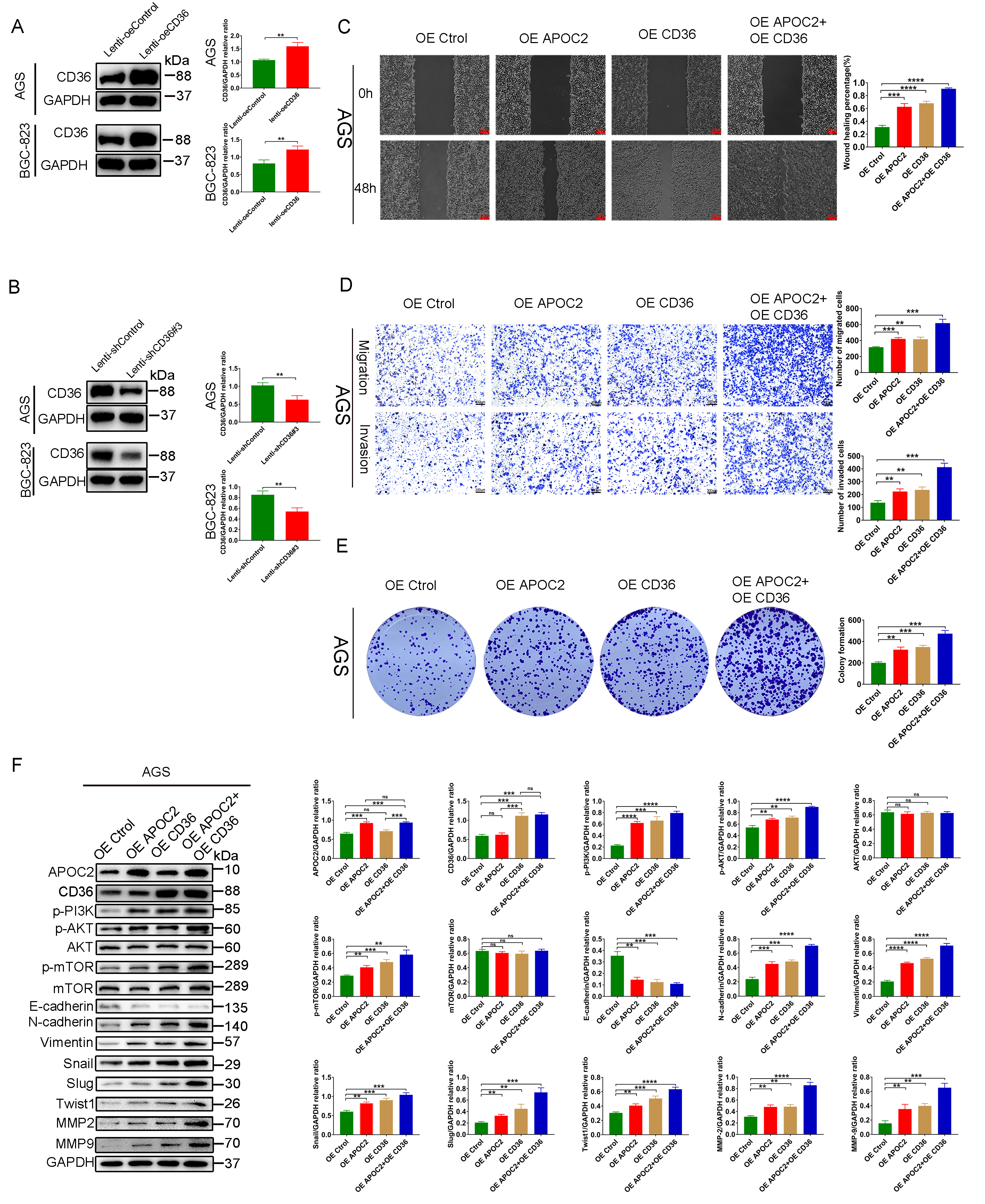

Supplement: Supplementary file 10 — Figure S9 APOC2 cooperates with CD36 mediates EMT via PI3K/AKT/mTOR signaling to promote AGS cell migration, invasion, and proliferation. [file CTM2-11-e522-s007.tif]

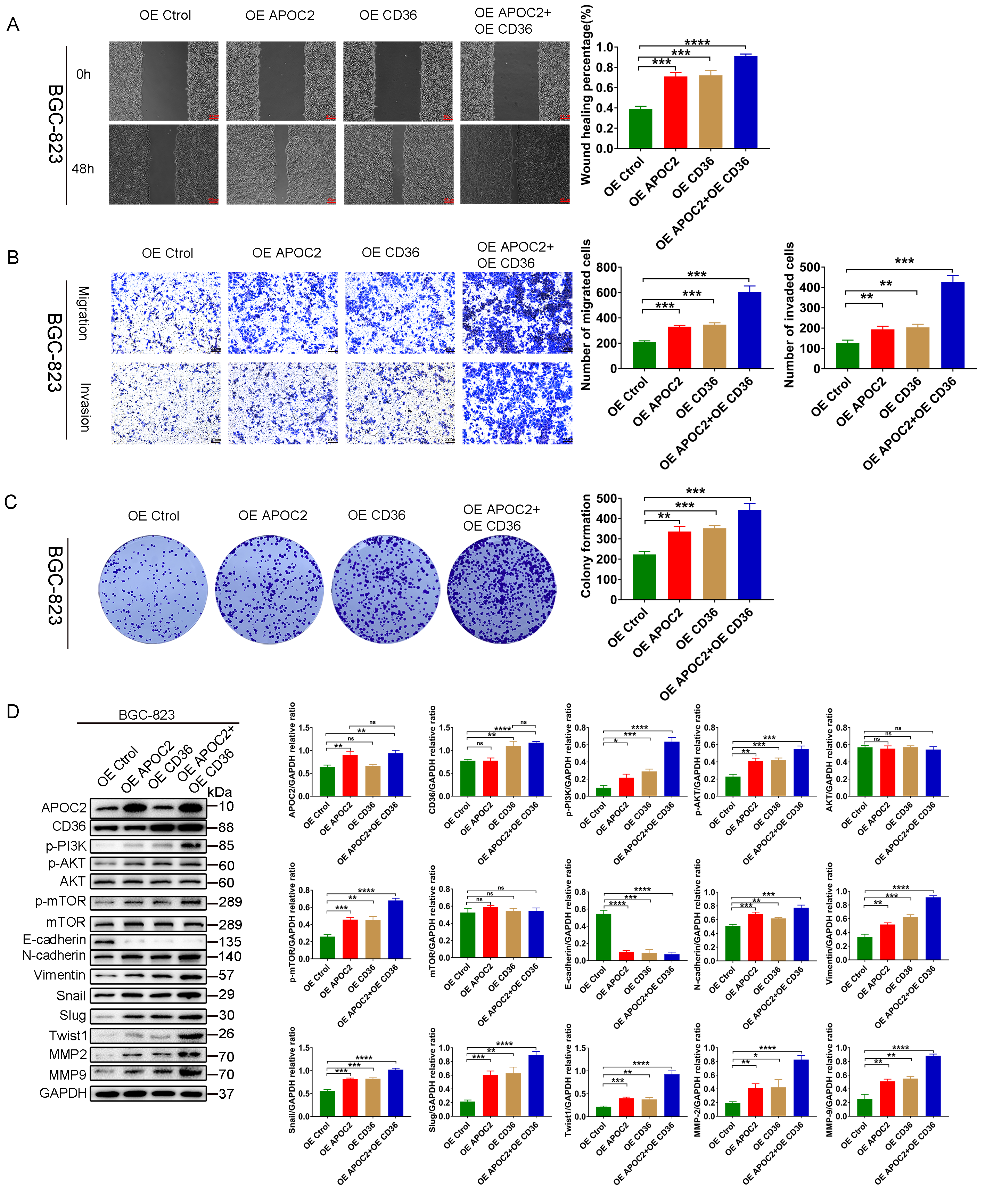

Supplement: Supplementary file 11 — Figure S10 APOC2 cooperates with CD36 mediates EMT via PI3K/AKT/mTOR signaling to promote BGC‐823 cell migration, invasion, and proliferation. [file CTM2-11-e522-s018.tif]

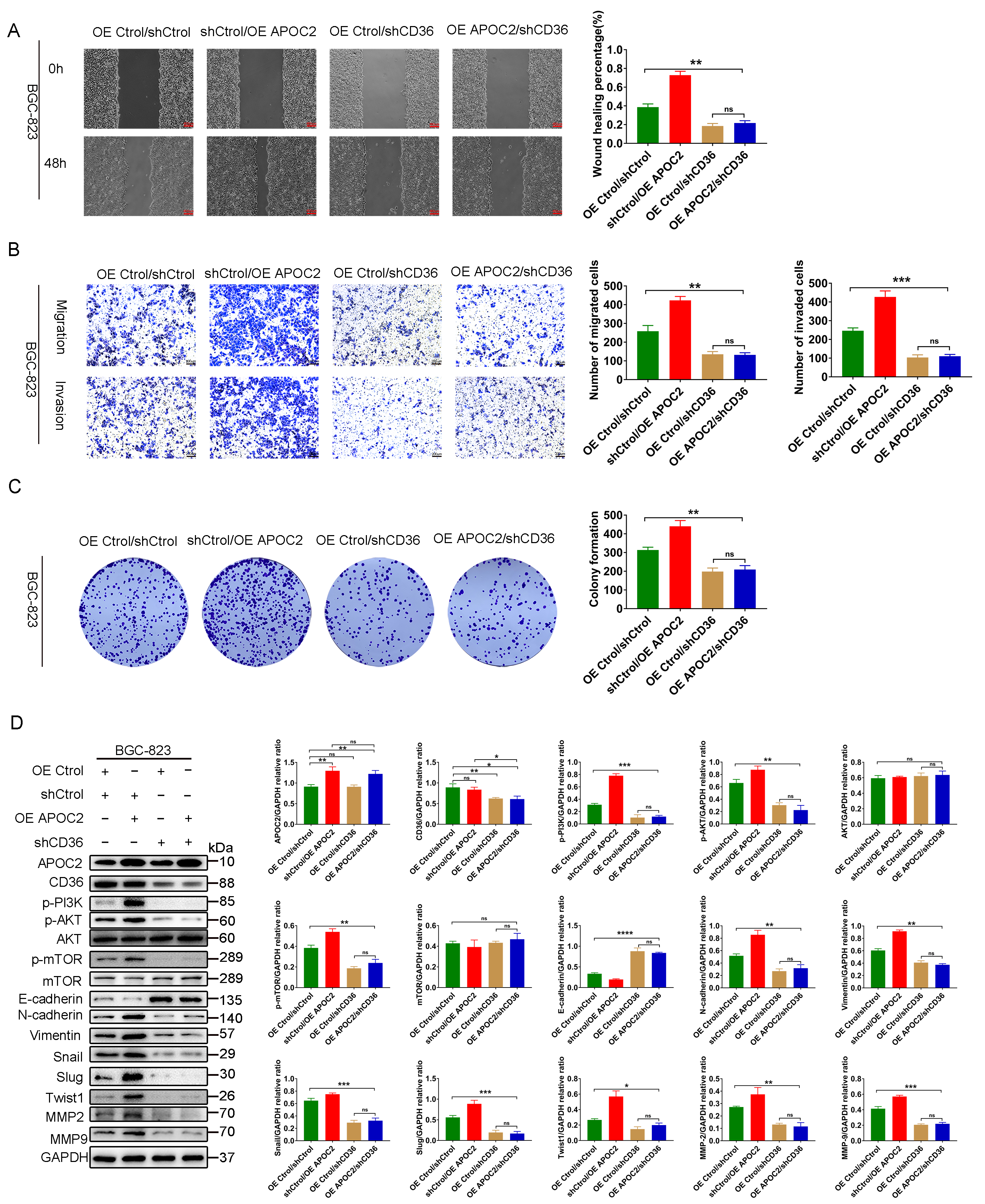

Supplement: Supplementary file 12 — Figure S11 Knockdown CD36 inhibits EMT via PI3K/AKT/mTOR signaling to suppress OE APOC2 stable BGC‐823 cell migration, invasion, and proliferation. [file CTM2-11-e522-s013.tif]

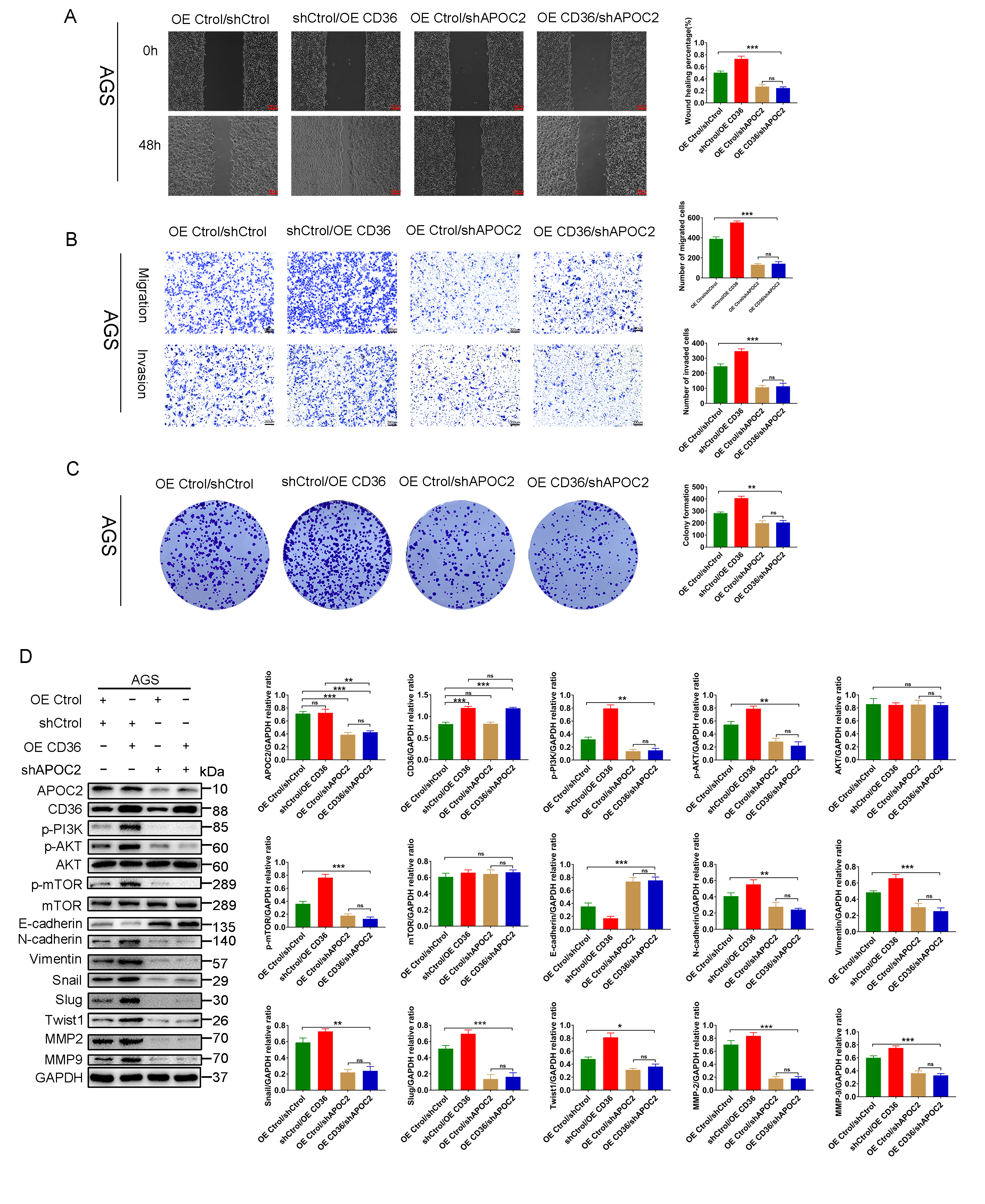

Supplement: Supplementary file 13 — Figure S12 Knockdown APOC2 inhibits EMT via PI3K/AKT/mTOR signaling to suppress OE CD36 stable AGS cell migration, invasion, and proliferation [file CTM2-11-e522-s011.tif]

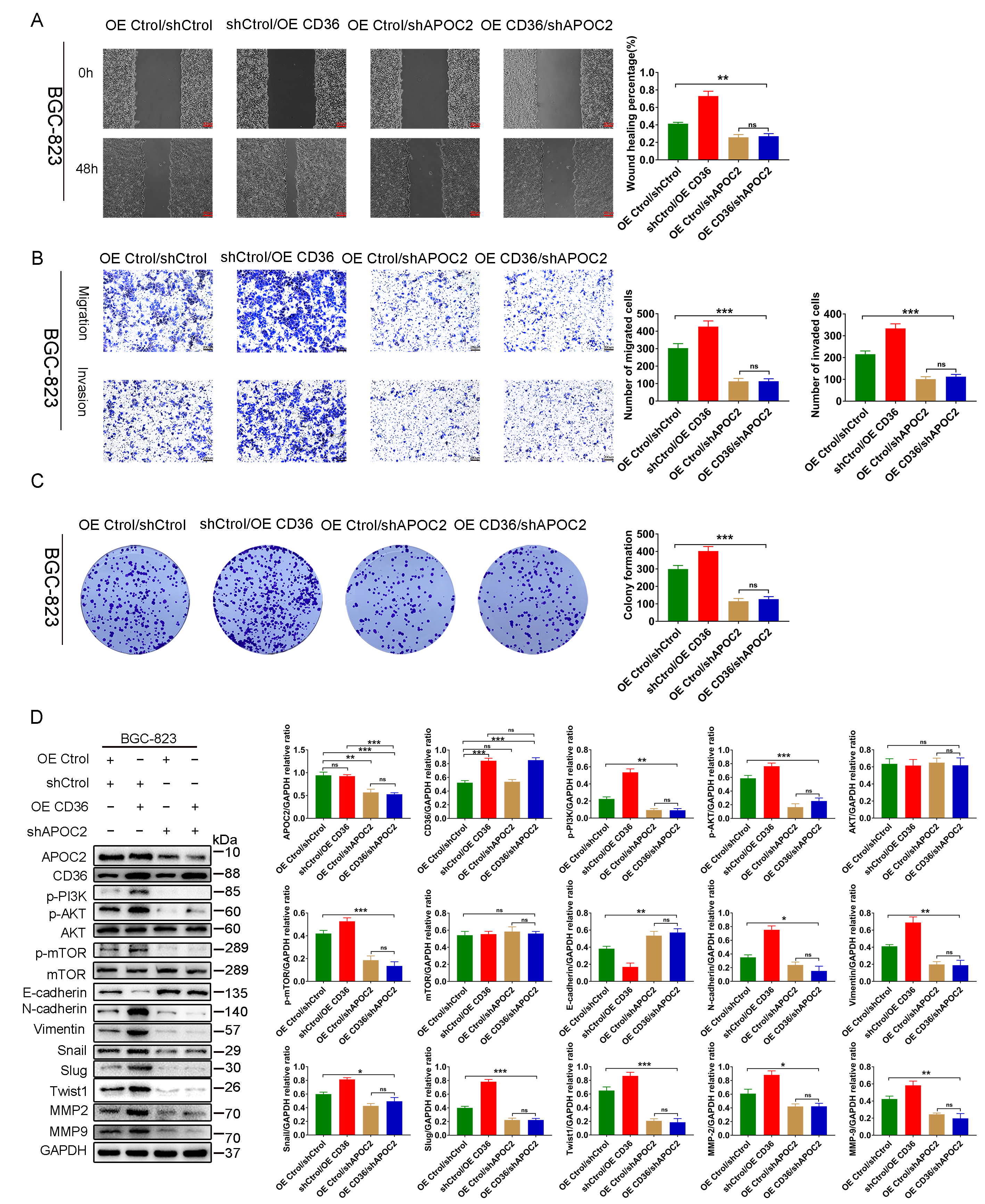

Supplement: Supplementary file 14 — Figure S13 Knockdown APOC2 inhibits EMT via PI3K/AKT/mTOR signaling to suppress OE CD36 stable BGC‐823 cell migration, invasion, and proliferation. [file CTM2-11-e522-s022.tif]

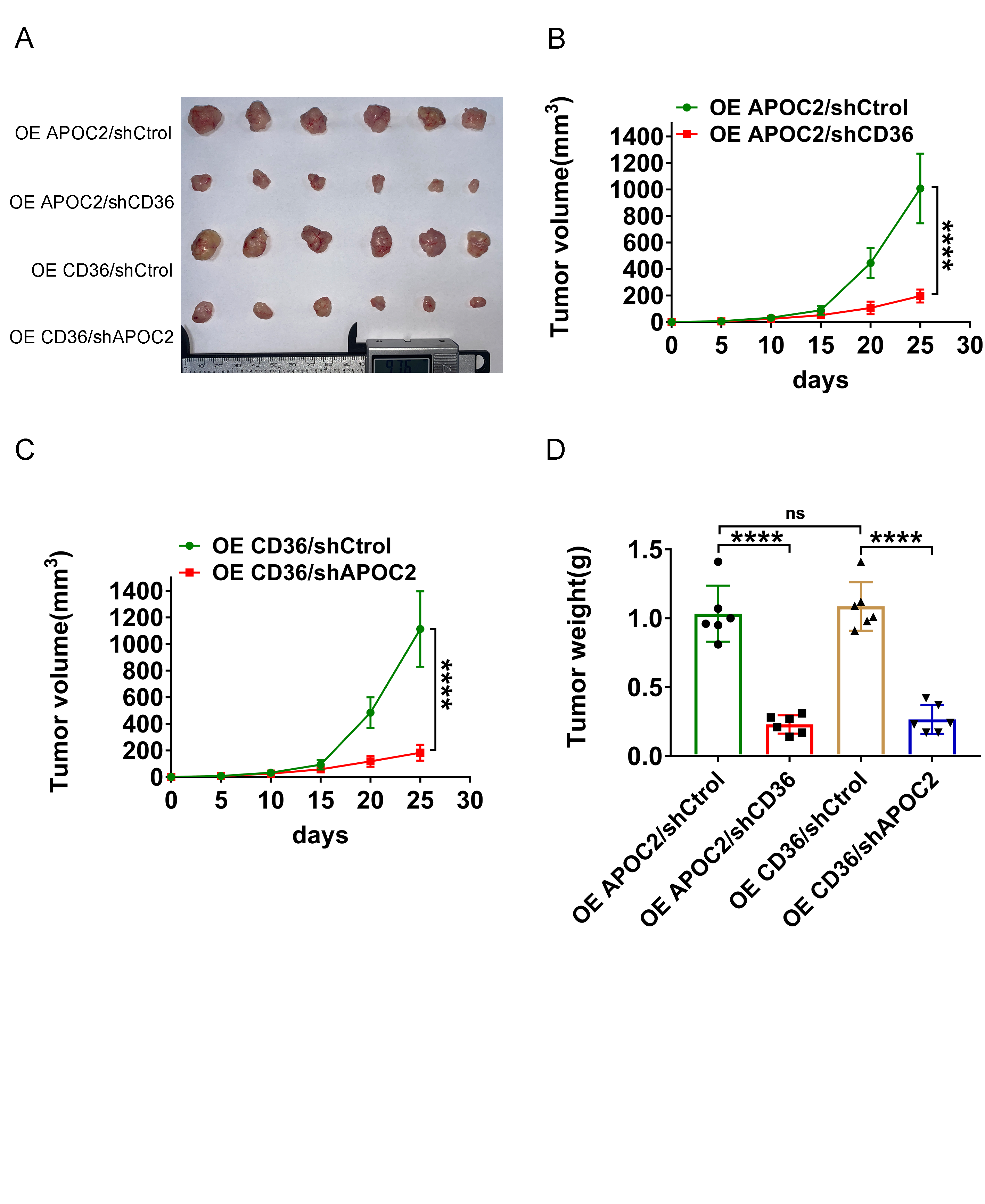

Supplement: Supplementary file 15 — Figure S14 APOC2 cooperates with CD36 to promote tumor progression in GC. [file CTM2-11-e522-s021.tif]
