# Supplementary material for: Apolipoprotein C‐II induces EMT to promote gastric cancer peritoneal metastasis via PI3K/AKT/mTOR pathway
Source: Clin Transl Med. 2021 Aug 9;11(8):e522. doi: 10.1002/ctm2.522 (PMC8351524; doi:10.1002/ctm2.522)
Supplement: Supplementary file 16 — Table S1. A total of 7638 proteins were identified at 1% false discovery rate (FDR). [file CTM2-11-e522-s017.docx]

**Table S1. A total of 7638 proteins were identified at 1% false discovery rate (FDR).**

| Protein_ID | Mass | Mean_Ratio_PM-VS-GC | SD_PM-VS-GC | Pvalue_PM-VS-GC |
| --- | --- | --- | --- | --- |
| sp|Q9BQ70|TCF25_HUMAN | 77227.9041 | 1.01 | 0.057 | 0.7498 |
| sp|Q6T310|RSLBA_HUMAN | 27142.22724 | 0.93 | 0.116 | 0.002856 |
| sp|P84085|ARF5_HUMAN | 20612.61223 | 0.69 | 0.081 | 2.70E-14 |
| sp|Q8NB46|ANR52_HUMAN | 116810.7143 | 0.89 | 0.093 | 5.33E-06 |
| sp|P19784|CSK22_HUMAN | 41340.17854 | 0.96 | 0.083 | 0.0215 |
| sp|P18428|LBP_HUMAN | 53502.96704 | 0.78 | 0.253 | 0.0002545 |
| sp|Q15545|TAF7_HUMAN | 40330.48204 | 1.19 | 0.127 | 2.71E-08 |
| sp|Q8WUF5|IASPP_HUMAN | 89359.86352 | 1.38 | 0.22 | 1.30E-09 |
| sp|O95273|CCDB1_HUMAN | 40617.59759 | 0.93 | 0.108 | 0.002639 |
| sp|A1IGU5|ARH37_HUMAN | 76612.06051 | 0.81 | 0.167 | 3.52E-05 |
| sp|Q658Y4|F91A1_HUMAN | 94630.01131 | 0.69 | 0.119 | 2.73E-11 |
| sp|P16104|H2AX_HUMAN | 15117.40454 | 1.74 | 0.563 | 2.20E-08 |
| sp|Q8N5I3|KCNRG_HUMAN | 31181.1404 | 0.54 | 0.092 | 3.69E-15 |
| sp|Q7L1T6|NB5R4_HUMAN | 59874.12246 | 0.68 | 0.152 | 1.48E-09 |
| sp|P62495|ERF1_HUMAN | 49210.22511 | 1.08 | 0.167 | 0.03752 |
| sp|Q01105|SET_HUMAN | 33450.69491 | 1.64 | 0.371 | 1.41E-10 |
| sp|Q12931|TRAP1_HUMAN | 80326.78803 | 0.75 | 0.156 | 5.17E-07 |
| sp|P10696|PPBN_HUMAN | 57608.07221 | 0.83 | 0.389 | 0.02188 |
| sp|P62333|PRS10_HUMAN | 44412.1958 | 1.21 | 0.102 | 7.06E-11 |
| sp|Q14240|IF4A2_HUMAN | 46582.85346 | 0.85 | 0.148 | 6.10E-05 |
| sp|Q8TBN0|R3GEF_HUMAN | 43161.78706 | 1.57 | 0.287 | 4.47E-11 |
| sp|P35240|MERL_HUMAN | 69856.03112 | 0.83 | 0.127 | 1.68E-06 |
| sp|Q00169|PIPNA_HUMAN | 31996.09224 | 1 | 0.091 | 0.6865 |
| sp|O75616|ERAL1_HUMAN | 48814.67067 | 0.84 | 0.154 | 1.92E-05 |
| sp|P00367|DHE3_HUMAN | 61683.3115 | 0.9 | 0.124 | 0.0005183 |
| sp|P35658|NU214_HUMAN | 214211.5207 | 1.17 | 0.091 | 4.98E-10 |
| sp|P14091|CATE_HUMAN | 43147.21571 | 0.82 | 0.334 | 0.008902 |
| sp|Q8TE73|DYH5_HUMAN | 532486.0075 | 1.03 | 0.117 | 0.3957 |
| sp|O95707|RPP29_HUMAN | 25561.87818 | 0.82 | 0.158 | 1.04E-05 |
| sp|Q9HBM6|TAF9B_HUMAN | 27700.45065 | 1.24 | 0.198 | 2.23E-06 |
| sp|O15091|MRPP3_HUMAN | 67995.72911 | 0.67 | 0.156 | 9.01E-09 |
| sp|P49006|MRP_HUMAN | 19556.24233 | 1.04 | 0.249 | 0.8049 |
| sp|Q96SB4|SRPK1_HUMAN | 74944.60486 | 1.01 | 0.225 | 0.7891 |
| sp|Q96KP4|CNDP2_HUMAN | 53169.03316 | 1.18 | 0.327 | 0.03438 |
| sp|Q2KHR3|QSER1_HUMAN | 190579.2651 | 1.18 | 0.2 | 0.0001633 |
| sp|Q8WUD4|CCD12_HUMAN | 19208.2501 | 1.2 | 0.247 | 0.0004638 |
| sp|Q6DKK2|TTC19_HUMAN | 42696.92451 | 0.77 | 0.117 | 5.85E-09 |
| sp|P17342|ANPRC_HUMAN | 60094.22867 | 1.32 | 0.509 | 0.004722 |
| sp|Q16595|FRDA_HUMAN | 23216.90104 | 1.11 | 0.193 | 0.0281 |
| sp|Q9UHR4|BI2L1_HUMAN | 57171.19436 | 0.88 | 0.195 | 0.003247 |
| sp|Q9HAB8|PPCS_HUMAN | 33965.78019 | 0.71 | 0.107 | 1.17E-11 |
| sp|Q9NP97|DLRB1_HUMAN | 10896.7123 | 1.29 | 0.16 | 7.68E-10 |
| sp|Q68DK2|ZFY26_HUMAN | 289109.9082 | 0.95 | 0.133 | 0.02973 |
| sp|Q9NRA2|S17A5_HUMAN | 55042.40962 | 0.73 | 0.244 | 2.18E-05 |
| sp|Q8WY91|THAP4_HUMAN | 63516.88186 | 0.93 | 0.175 | 0.02475 |
| sp|Q8NCH0|CHSTE_HUMAN | 43350.57042 | 0.85 | 0.208 | 0.0008263 |
| sp|Q9BXS5|AP1M1_HUMAN | 48709.11042 | 0.77 | 0.073 | 4.12E-13 |
| sp|P17010|ZFX_HUMAN | 92270.34112 | 1.17 | 0.191 | 0.0001763 |
| sp|Q5VWJ9|SNX30_HUMAN | 49969.29748 | 1.31 | 0.147 | 8.56E-12 |
| sp|O14556|G3PT_HUMAN | 44796.9082 | 1.11 | 0.25 | 0.05532 |
| sp|P04070|PROC_HUMAN | 53387.97058 | 0.98 | 0.212 | 0.3666 |
| sp|Q9P2P6|STAR9_HUMAN | 521821.6543 | 0.78 | 0.233 | 4.93E-05 |
| sp|Q92569|P55G_HUMAN | 54680.98611 | 1.77 | 0.635 | 5.82E-08 |
| sp|Q9NVU7|SDA1_HUMAN | 80258.64025 | 0.69 | 0.103 | 5.07E-12 |
| sp|P18859|ATP5J_HUMAN | 12561.593 | 1.55 | 0.51 | 1.97E-05 |
| sp|Q02575|HEN1_HUMAN | 14704.55822 | 1.03 | 0.228 | 0.8841 |
| sp|Q9BXR0|TGT_HUMAN | 44685.35888 | 0.82 | 0.107 | 2.84E-08 |
| sp|Q9HCM4|E41L5_HUMAN | 82357.11146 | 0.62 | 0.209 | 1.17E-08 |
| sp|Q14520|HABP2_HUMAN | 64722.22577 | 0.92 | 0.384 | 0.0653 |
| sp|P08218|CEL2B_HUMAN | 29400.259 | 0.5 | 0.296 | 7.15E-07 |
| sp|O95319|CELF2_HUMAN | 54688.2535 | 1.45 | 0.147 | 4.06E-15 |
| sp|Q3KRA9|ALKB6_HUMAN | 26562.99091 | 0.87 | 0.12 | 2.46E-05 |
| sp|Q9H0F6|SHRPN_HUMAN | 40476.43956 | 0.88 | 0.2 | 0.003834 |
| sp|Q6P3W7|SCYL2_HUMAN | 104308.661 | 0.78 | 0.067 | 2.00E-13 |
| sp|Q7Z6B0|CCD91_HUMAN | 50150.84341 | 1.23 | 0.231 | 1.87E-05 |
| sp|Q9H9P8|L2HDH_HUMAN | 51007.18913 | 0.67 | 0.165 | 9.38E-09 |
| sp|Q5BKZ1|ZN326_HUMAN | 65937.16796 | 1.44 | 0.34 | 1.63E-07 |
| sp|Q15738|NSDHL_HUMAN | 42140.66561 | 0.72 | 0.131 | 1.03E-09 |
| sp|P35579|MYH9_HUMAN | 227628.0531 | 0.8 | 0.093 | 7.92E-10 |
| sp|Q9BZL1|UBL5_HUMAN | 8637.458979 | 1.38 | 0.32 | 6.25E-06 |
| sp|Q9NZN8|CNOT2_HUMAN | 59852.98478 | 1.28 | 0.128 | 1.92E-11 |
| sp|Q14258|TRI25_HUMAN | 72563.33811 | 1.01 | 0.196 | 0.9021 |
| sp|P21860|ERBB3_HUMAN | 151405.5683 | 0.62 | 0.247 | 5.81E-07 |
| sp|Q9Y2H1|ST38L_HUMAN | 54178.48656 | 0.87 | 0.092 | 9.47E-07 |
| sp|Q9HCD5|NCOA5_HUMAN | 65706.55058 | 1.28 | 0.139 | 4.36E-11 |
| sp|P00519|ABL1_HUMAN | 123576.6259 | 1.54 | 0.49 | 3.10E-06 |
| sp|Q6RFH5|WDR74_HUMAN | 42966.8678 | 0.9 | 0.335 | 0.04576 |
| sp|Q92993|KAT5_HUMAN | 59268.35177 | 0.73 | 0.18 | 4.50E-07 |
| sp|Q9H1K1|ISCU_HUMAN | 18197.70917 | 1.25 | 0.17 | 4.80E-08 |
| sp|Q14956|GPNMB_HUMAN | 64605.17921 | 1.04 | 0.207 | 0.6681 |
| sp|Q9H9C1|SPE39_HUMAN | 57180.32294 | 0.6 | 0.055 | 2.20E-16 |
| sp|Q9NVU0|RPC5_HUMAN | 80513.65594 | 0.94 | 0.227 | 0.0779 |
| sp|O76041|NEBL_HUMAN | 116590.5732 | 0.82 | 0.152 | 3.14E-06 |
| sp|P46013|KI67_HUMAN | 360679.5273 | 1.27 | 0.601 | 0.108 |
| sp|Q05209|PTN12_HUMAN | 88888.74837 | 0.99 | 0.149 | 0.5549 |
| sp|O14874|BCKD_HUMAN | 46598.27291 | 0.64 | 0.205 | 3.24E-08 |
| sp|Q53HC9|EIPR1_HUMAN | 44014.23191 | 1.32 | 0.225 | 1.48E-07 |
| sp|Q9Y4G2|PKHM1_HUMAN | 119346.6985 | 1 | 0.263 | 0.5636 |
| sp|O95715|CXL14_HUMAN | 13279.12915 | 1.18 | 0.21 | 0.0002055 |
| sp|Q8N2M8|CLASR_HUMAN | 77267.93036 | 0.67 | 0.117 | 2.33E-11 |
| sp|O43169|CYB5B_HUMAN | 16418.00561 | 1.2 | 0.259 | 0.001024 |
| sp|Q8WXE1|ATRIP_HUMAN | 87076.94972 | 1.12 | 0.169 | 0.001777 |
| sp|Q8N4Z0|RAB42_HUMAN | 24499.3317 | 0.99 | 0.244 | 0.4676 |
| sp|Q9Y4K3|TRAF6_HUMAN | 61226.33621 | 0.78 | 0.058 | 1.76E-14 |
| sp|P52758|RIDA_HUMAN | 14523.62199 | 1.17 | 0.356 | 0.06564 |
| sp|P20073|ANXA7_HUMAN | 52972.84775 | 0.88 | 0.093 | 7.39E-07 |
| sp|Q04323|UBXN1_HUMAN | 33400.97271 | 1.45 | 0.253 | 5.65E-10 |
| sp|Q13561|DCTN2_HUMAN | 44299.88842 | 1.47 | 0.183 | 6.88E-14 |
| sp|O43824|GTPB6_HUMAN | 57186.22372 | 1.02 | 0.091 | 0.4683 |
| sp|Q9ULL8|SHRM4_HUMAN | 167017.3149 | 1.01 | 0.151 | 0.8624 |
| sp|P53985|MOT1_HUMAN | 54575.09799 | 0.69 | 0.194 | 9.22E-08 |
| sp|Q9Y3I1|FBX7_HUMAN | 58904.09374 | 0.82 | 0.146 | 7.03E-06 |
| sp|Q8NHQ9|DDX55_HUMAN | 69055.33692 | 0.86 | 0.206 | 0.001335 |
| sp|O95864|FADS2_HUMAN | 52321.97398 | 0.74 | 0.113 | 1.91E-10 |
| sp|Q96KG9|SCYL1_HUMAN | 90184.44683 | 0.69 | 0.07 | 2.71E-15 |
| sp|P27918|PROP_HUMAN | 53732.92488 | 1.01 | 0.459 | 0.3697 |
| sp|Q8WWI5|CTL1_HUMAN | 74774.83142 | 0.79 | 0.287 | 0.000651 |
| sp|O15382|BCAT2_HUMAN | 44640.19801 | 0.68 | 0.102 | 3.95E-12 |
| sp|P46977|STT3A_HUMAN | 81086.07121 | 0.51 | 0.129 | 9.16E-13 |
| sp|Q6NZI2|CAVN1_HUMAN | 43431.80874 | 1.86 | 0.384 | 4.03E-12 |
| sp|P54803|GALC_HUMAN | 77281.83621 | 0.57 | 0.207 | 1.69E-08 |
| sp|P24386|RAE1_HUMAN | 74722.08375 | 0.68 | 0.059 | 2.20E-16 |
| sp|A6NGU5|GGT3_HUMAN | 61900.94562 | 1.04 | 0.23 | 0.8167 |
| sp|Q15061|WDR43_HUMAN | 75794.79106 | 0.85 | 0.147 | 2.46E-05 |
| sp|Q9UPQ0|LIMC1_HUMAN | 122800.2793 | 1.45 | 0.345 | 2.33E-07 |
| sp|P19021|AMD_HUMAN | 109100.5888 | 0.66 | 0.225 | 3.92E-07 |
| sp|Q6P179|ERAP2_HUMAN | 111057.3434 | 0.82 | 0.461 | 0.005236 |
| sp|Q9UQ35|SRRM2_HUMAN | 300161.2786 | 1.41 | 0.109 | 2.20E-16 |
| sp|P62854|RS26_HUMAN | 13274.18105 | 1.13 | 0.238 | 0.03625 |
| sp|Q9Y2Z0|SGT1_HUMAN | 41265.58694 | 1.29 | 0.18 | 5.07E-09 |
| sp|P78560|CRADD_HUMAN | 22769.94213 | 1.25 | 0.277 | 0.0002114 |
| sp|Q9NQ29|LUC7L_HUMAN | 44082.45974 | 1.29 | 0.218 | 3.87E-07 |
| sp|Q7L523|RRAGA_HUMAN | 36923.69182 | 0.6 | 0.04 | 2.20E-16 |
| sp|Q9NWM0|SMOX_HUMAN | 62389.73358 | 1.17 | 0.148 | 4.59E-06 |
| sp|Q99501|GA2L1_HUMAN | 73338.09864 | 0.94 | 0.191 | 0.07731 |
| sp|P05165|PCCA_HUMAN | 80617.32325 | 0.7 | 0.201 | 1.52E-06 |
| sp|Q96GM5|SMRD1_HUMAN | 58462.94808 | 0.9 | 0.14 | 0.0005598 |
| sp|Q6DD88|ATLA3_HUMAN | 60941.58449 | 0.58 | 0.058 | 2.20E-16 |
| sp|Q96GQ5|RUS1_HUMAN | 51423.38651 | 0.75 | 0.099 | 6.58E-11 |
| sp|O43847|NRDC_HUMAN | 132625.9086 | 0.55 | 0.119 | 1.79E-13 |
| sp|P47755|CAZA2_HUMAN | 33138.68347 | 0.89 | 0.081 | 7.50E-07 |
| sp|Q8IZ07|AN13A_HUMAN | 67957.92661 | 0.81 | 0.206 | 0.00014 |
| sp|P49326|FMO5_HUMAN | 60620.07216 | 0.5 | 0.094 | 3.73E-15 |
| sp|Q9UKX7|NUP50_HUMAN | 50494.30329 | 1.16 | 0.146 | 1.72E-05 |
| sp|Q5T9L3|WLS_HUMAN | 62877.93437 | 0.49 | 0.098 | 9.57E-16 |
| sp|P04843|RPN1_HUMAN | 68622.84049 | 0.68 | 0.108 | 4.82E-12 |
| sp|P00387|NB5R3_HUMAN | 34422.79788 | 0.67 | 0.083 | 1.16E-14 |
| sp|Q02252|MMSA_HUMAN | 58240.81337 | 1.25 | 0.388 | 0.01645 |
| sp|P50502|F10A1_HUMAN | 41458.5297 | 1.34 | 0.236 | 5.20E-08 |
| sp|Q96CB9|NSUN4_HUMAN | 43613.26333 | 0.71 | 0.103 | 8.96E-12 |
| sp|Q9H7Z7|PGES2_HUMAN | 42069.7267 | 0.79 | 0.13 | 4.78E-08 |
| sp|P21964|COMT_HUMAN | 30455.792 | 0.92 | 0.18 | 0.01867 |
| sp|Q3KQV9|UAP1L_HUMAN | 57546.55802 | 1.1 | 0.142 | 0.001651 |
| sp|P10645|CMGA_HUMAN | 50810.7086 | 0.72 | 0.398 | 0.0006915 |
| sp|P61204|ARF3_HUMAN | 20626.74087 | 0.69 | 0.088 | 8.82E-14 |
| sp|Q96P16|RPR1A_HUMAN | 35907.64363 | 1.02 | 0.174 | 0.925 |
| sp|Q15011|HERP1_HUMAN | 43788.62609 | 1.16 | 0.18 | 0.0001331 |
| sp|Q9UNP9|PPIE_HUMAN | 33676.71103 | 1.36 | 0.259 | 1.51E-08 |
| sp|O00192|ARVC_HUMAN | 105243.6709 | 0.71 | 0.183 | 8.39E-08 |
| sp|Q9UKN8|TF3C4_HUMAN | 93216.94565 | 0.67 | 0.137 | 3.98E-10 |
| sp|P13284|GILT_HUMAN | 28554.23424 | 1.19 | 0.13 | 3.44E-08 |
| sp|Q7Z4Q2|HEAT3_HUMAN | 75771.44601 | 0.46 | 0.143 | 6.83E-12 |
| sp|O15254|ACOX3_HUMAN | 78303.11357 | 0.76 | 0.175 | 1.39E-06 |
| sp|Q9C040|TRIM2_HUMAN | 82487.5215 | 0.9 | 0.094 | 2.11E-05 |
| sp|Q9Y320|TMX2_HUMAN | 34339.75808 | 0.81 | 0.109 | 8.81E-08 |
| sp|O60826|CCD22_HUMAN | 71036.31547 | 1.02 | 0.068 | 0.2111 |
| sp|P55075|FGF8_HUMAN | 26718.94923 | 1.02 | 0.332 | 0.6043 |
| sp|Q9NWX6|THG1_HUMAN | 35132.55582 | 0.91 | 0.138 | 0.002817 |
| sp|O75570|RF1M_HUMAN | 52825.02369 | 1.07 | 0.175 | 0.1335 |
| sp|P41235|HNF4A_HUMAN | 53474.04882 | 0.6 | 0.238 | 7.07E-08 |
| sp|Q16774|KGUA_HUMAN | 21751.2126 | 1.72 | 0.195 | 2.20E-16 |
| sp|O43143|DHX15_HUMAN | 91655.45566 | 0.75 | 0.076 | 2.18E-13 |
| sp|Q9H4H8|FA83D_HUMAN | 64822.4994 | 0.55 | 0.143 | 1.21E-11 |
| sp|P13051|UNG_HUMAN | 34776.89599 | 0.72 | 0.169 | 8.22E-08 |
| sp|Q9C0D6|FHDC1_HUMAN | 125635.7815 | 1.63 | 0.473 | 2.27E-08 |
| sp|Q9H3S1|SEM4A_HUMAN | 84642.67135 | 0.92 | 0.268 | 0.0376 |
| sp|Q14692|BMS1_HUMAN | 146553.1532 | 0.77 | 0.143 | 5.49E-07 |
| sp|Q96GG9|DCNL1_HUMAN | 30315.12605 | 0.65 | 0.17 | 3.01E-08 |
| sp|P17213|BPI_HUMAN | 54075.14061 | 0.5 | 0.313 | 3.25E-06 |
| sp|O60437|PEPL_HUMAN | 205175.2314 | 1.36 | 0.416 | 0.0004804 |
| sp|P04003|C4BPA_HUMAN | 69024.15927 | 0.81 | 0.427 | 0.005869 |
| sp|Q7Z460|CLAP1_HUMAN | 170354.5433 | 0.62 | 0.069 | 2.20E-16 |
| sp|Q10713|MPPA_HUMAN | 58710.78444 | 1.05 | 0.157 | 0.2027 |
| sp|P02788|TRFL_HUMAN | 79995.61111 | 0.71 | 0.464 | 0.0003434 |
| sp|P57088|TMM33_HUMAN | 28283.93002 | 0.61 | 0.127 | 9.98E-11 |
| sp|P62913|RL11_HUMAN | 20449.71538 | 1.26 | 0.167 | 2.32E-08 |
| sp|Q8IVT5|KSR1_HUMAN | 103275.1422 | 1.03 | 0.149 | 0.5043 |
| sp|O00178|GTPB1_HUMAN | 73017.24602 | 0.93 | 0.117 | 0.005876 |
| sp|Q13347|EIF3I_HUMAN | 36859.75328 | 1.31 | 0.156 | 6.78E-11 |
| sp|Q9NRX1|PNO1_HUMAN | 28059.91395 | 0.64 | 0.192 | 2.61E-08 |
| sp|O95985|TOP3B_HUMAN | 98519.87347 | 1.02 | 0.315 | 0.7317 |
| sp|Q9P291|ARMX1_HUMAN | 49701.82282 | 0.94 | 0.209 | 0.06217 |
| sp|P16402|H13_HUMAN | 22318.30861 | 2.38 | 1.669 | 0.00414 |
| sp|P46734|MP2K3_HUMAN | 39617.04821 | 0.57 | 0.13 | 1.97E-12 |
| sp|O00625|PIR_HUMAN | 32189.28865 | 1.19 | 0.285 | 0.009321 |
| sp|O75884|RBBP9_HUMAN | 21196.5464 | 1.05 | 0.195 | 0.4402 |
| sp|Q676U5|A16L1_HUMAN | 68888.91124 | 1.01 | 0.118 | 0.8121 |
| sp|Q9UJ70|NAGK_HUMAN | 37676.04008 | 0.65 | 0.127 | 4.18E-11 |
| sp|Q99622|C10_HUMAN | 13265.70381 | 1.43 | 0.249 | 1.61E-09 |
| sp|O95163|ELP1_HUMAN | 151794.5724 | 0.82 | 0.142 | 1.58E-06 |
| sp|Q11206|SIA4C_HUMAN | 38231.12265 | 0.6 | 0.233 | 9.76E-07 |
| sp|Q9H0L4|CSTFT_HUMAN | 64605.58926 | 1.22 | 0.161 | 1.29E-07 |
| sp|Q86U86|PB1_HUMAN | 194061.6915 | 1.14 | 0.176 | 0.0003085 |
| sp|P22894|MMP8_HUMAN | 53531.55363 | 0.55 | 0.34 | 2.08E-06 |
| sp|Q8N2S1|LTBP4_HUMAN | 182425.2298 | 0.82 | 0.342 | 0.001172 |
| sp|Q9Y6D6|BIG1_HUMAN | 210840.3221 | 0.7 | 0.104 | 4.91E-12 |
| sp|Q9UBF6|RBX2_HUMAN | 13340.86671 | 0.74 | 0.108 | 2.01E-10 |
| sp|Q8N653|LZTR1_HUMAN | 95894.63444 | 0.92 | 0.206 | 0.03985 |
| sp|Q9P2D7|DYH1_HUMAN | 491139.8177 | 1.74 | 0.817 | 9.62E-06 |
| sp|P54646|AAPK2_HUMAN | 62831.77536 | 0.66 | 0.157 | 2.12E-09 |
| sp|Q5T3U5|MRP7_HUMAN | 163048.5367 | 1.72 | 1.43 | 0.04558 |
| sp|Q5BKT4|AG10A_HUMAN | 56292.26899 | 1 | 0.166 | 0.6743 |
| sp|Q14573|ITPR3_HUMAN | 306802.1989 | 0.69 | 0.154 | 2.09E-09 |
| sp|Q9NRG7|D39U1_HUMAN | 31096.39292 | 0.9 | 0.195 | 0.007807 |
| sp|Q13873|BMPR2_HUMAN | 116422.3104 | 0.97 | 0.113 | 0.1208 |
| sp|Q7Z6I6|RHG30_HUMAN | 119631.4072 | 1.07 | 0.167 | 0.08239 |
| sp|P52907|CAZA1_HUMAN | 33055.38176 | 1.04 | 0.124 | 0.1585 |
| sp|Q8WX93|PALLD_HUMAN | 151820.7409 | 0.93 | 0.204 | 0.04765 |
| sp|P14317|HCLS1_HUMAN | 54077.00705 | 1.56 | 0.48 | 3.75E-07 |
| sp|Q0VDD8|DYH14_HUMAN | 403898.4635 | 0.8 | 0.246 | 0.0002384 |
| sp|P27986|P85A_HUMAN | 83870.6584 | 1.04 | 0.119 | 0.1716 |
| sp|Q9Y6Q1|CAN6_HUMAN | 75365.94476 | 0.49 | 0.167 | 2.57E-10 |
| sp|P29536|LMOD1_HUMAN | 67141.99743 | 0.41 | 0.215 | 3.29E-10 |
| sp|Q96JJ3|ELMO2_HUMAN | 83000.42598 | 0.81 | 0.219 | 8.70E-05 |
| sp|Q8NBN7|RDH13_HUMAN | 36176.95139 | 0.82 | 0.137 | 5.28E-06 |
| sp|P27708|PYR1_HUMAN | 245149.3254 | 0.7 | 0.083 | 5.18E-14 |
| sp|I3L1E1|CS084_HUMAN | 19811.96825 | 2.22 | 1.013 | 6.92E-09 |
| sp|Q8N3R9|MPP5_HUMAN | 77512.64341 | 1.15 | 0.226 | 0.01279 |
| sp|Q5UIP0|RIF1_HUMAN | 276442.9559 | 1.12 | 0.212 | 0.01276 |
| sp|P02774|VTDB_HUMAN | 54461.53065 | 1.12 | 0.324 | 0.1492 |
| sp|P30679|GNA15_HUMAN | 43978.33015 | 0.65 | 0.132 | 8.96E-11 |
| sp|Q53EP0|FND3B_HUMAN | 134952.0433 | 1 | 0.172 | 0.6836 |
| sp|P43235|CATK_HUMAN | 37380.5715 | 1.49 | 0.596 | 0.001804 |
| sp|Q9NX46|ARHL2_HUMAN | 39246.28543 | 1.38 | 0.099 | 2.20E-16 |
| sp|A8K7I4|CLCA1_HUMAN | 100886.3383 | 0.8 | 0.445 | 0.004941 |
| sp|Q96IZ5|RBM41_HUMAN | 47337.20752 | 1.22 | 0.312 | 0.003908 |
| sp|Q96FV2|SCRN2_HUMAN | 47005.31168 | 1.39 | 0.199 | 1.49E-10 |
| sp|Q96FZ7|CHMP6_HUMAN | 23509.31319 | 1.49 | 0.283 | 4.49E-10 |
| sp|Q9Y2X7|GIT1_HUMAN | 85012.07817 | 1.21 | 0.173 | 1.90E-06 |
| sp|Q9NYF8|BCLF1_HUMAN | 106154.8233 | 1.5 | 0.302 | 4.16E-10 |
| sp|Q9UN86|G3BP2_HUMAN | 54126.81739 | 1.12 | 0.112 | 1.68E-05 |
| sp|O75717|WDHD1_HUMAN | 127352.7495 | 0.86 | 0.144 | 4.70E-05 |
| sp|O95352|ATG7_HUMAN | 79031.7429 | 0.6 | 0.076 | 2.20E-16 |
| sp|Q9Y6I3|EPN1_HUMAN | 60351.74911 | 1.33 | 0.218 | 4.33E-08 |
| sp|Q2TAY7|SMU1_HUMAN | 58116.45507 | 0.91 | 0.058 | 1.18E-07 |
| sp|Q53H82|LACB2_HUMAN | 33052.04002 | 1.03 | 0.276 | 0.8635 |
| sp|Q9UH99|SUN2_HUMAN | 80471.63563 | 0.74 | 0.081 | 4.04E-13 |
| sp|Q9BUR5|MIC26_HUMAN | 22366.56439 | 0.62 | 0.151 | 3.86E-10 |
| sp|Q8WVQ1|CANT1_HUMAN | 44907.72121 | 0.58 | 0.102 | 5.85E-14 |
| sp|Q13795|ARFRP_HUMAN | 23037.60619 | 0.67 | 0.144 | 1.88E-10 |
| sp|P10301|RRAS_HUMAN | 23618.944 | 0.64 | 0.334 | 4.37E-06 |
| sp|P60900|PSA6_HUMAN | 27819.97015 | 1.1 | 0.179 | 0.0165 |
| sp|Q99683|M3K5_HUMAN | 155733.0376 | 0.95 | 0.183 | 0.08228 |
| sp|Q9UBY9|HSPB7_HUMAN | 18638.045 | 1.2 | 0.257 | 0.001255 |
| sp|Q9HAN9|NMNA1_HUMAN | 32122.65238 | 1.3 | 0.221 | 1.25E-07 |
| sp|Q9BQ67|GRWD1_HUMAN | 49769.30269 | 1.09 | 0.101 | 0.000202 |
| sp|Q9BTW9|TBCD_HUMAN | 134264.9403 | 0.58 | 0.091 | 2.60E-15 |
| sp|P55083|MFAP4_HUMAN | 28954.00999 | 0.9 | 0.553 | 0.02964 |
| sp|Q9UBC2|EP15R_HUMAN | 94293.06589 | 1.47 | 0.189 | 2.53E-13 |
| sp|Q8TEH3|DEN1A_HUMAN | 111117.1229 | 1.53 | 0.413 | 1.33E-07 |
| sp|Q15643|TRIPB_HUMAN | 228112.8299 | 1.17 | 0.167 | 3.27E-05 |
| sp|O75431|MTX2_HUMAN | 30068.18783 | 0.7 | 0.15 | 5.44E-09 |
| sp|Q7Z4W1|DCXR_HUMAN | 26163.65183 | 0.72 | 0.148 | 4.69E-08 |
| sp|Q07960|RHG01_HUMAN | 50443.19728 | 0.77 | 0.081 | 2.83E-12 |
| sp|O60721|NCKX1_HUMAN | 121678.3393 | 0.83 | 0.204 | 0.0002145 |
| sp|O00541|PESC_HUMAN | 68341.1385 | 0.72 | 0.128 | 2.94E-09 |
| sp|P01591|IGJ_HUMAN | 18525.15585 | 1.1 | 0.39 | 0.5942 |
| sp|P17612|KAPCA_HUMAN | 40660.03019 | 0.81 | 0.097 | 2.98E-09 |
| sp|O75110|ATP9A_HUMAN | 119970.7235 | 0.6 | 0.135 | 1.81E-11 |
| sp|P55212|CASP6_HUMAN | 33840.70125 | 0.91 | 0.173 | 0.008851 |
| sp|Q9UBB9|TFP11_HUMAN | 97139.57671 | 0.88 | 0.113 | 2.47E-05 |
| sp|P55786|PSA_HUMAN | 103876.9761 | 0.61 | 0.069 | 2.20E-16 |
| sp|Q9P107|GMIP_HUMAN | 107568.1127 | 1.18 | 0.231 | 0.0008147 |
| sp|P54136|SYRC_HUMAN | 76111.15326 | 0.62 | 0.117 | 1.86E-12 |
| sp|P04275|VWF_HUMAN | 322382.929 | 0.99 | 0.314 | 0.3861 |
| sp|Q9BX40|LS14B_HUMAN | 42141.3396 | 1.22 | 0.461 | 0.05957 |
| sp|Q8N335|GPD1L_HUMAN | 39003.15043 | 0.66 | 0.154 | 1.06E-08 |
| sp|P60660|MYL6_HUMAN | 17072.18699 | 1.05 | 0.333 | 0.9739 |
| sp|P06493|CDK1_HUMAN | 34112.89441 | 0.72 | 0.217 | 2.94E-06 |
| sp|Q9BXY4|RSPO3_HUMAN | 32144.7364 | 0.97 | 0.907 | 0.04087 |
| sp|Q9Y217|MTMR6_HUMAN | 72759.48257 | 0.92 | 0.155 | 0.01219 |
| sp|Q9NXF1|TEX10_HUMAN | 106330.8796 | 0.82 | 0.203 | 0.0001619 |
| sp|Q9H239|MMP28_HUMAN | 59169.31485 | 0.87 | 0.146 | 0.0001304 |
| sp|Q12774|ARHG5_HUMAN | 177869.8944 | 1.12 | 0.304 | 0.1605 |
| sp|P15882|CHIN_HUMAN | 53747.18409 | 0.77 | 0.191 | 3.68E-06 |
| sp|P67870|CSK2B_HUMAN | 25250.12047 | 1.18 | 0.187 | 7.75E-05 |
| sp|P53350|PLK1_HUMAN | 68934.85644 | 0.83 | 0.249 | 0.0005408 |
| sp|P62166|NCS1_HUMAN | 21903.91701 | 1.24 | 0.332 | 0.002729 |
| sp|Q9NUL5|RYDEN_HUMAN | 33755.40268 | 1.24 | 0.377 | 0.005635 |
| sp|Q8NFL0|B3GN7_HUMAN | 46452.86299 | 0.55 | 0.216 | 7.73E-08 |
| sp|Q9Y490|TLN1_HUMAN | 271747.8544 | 0.91 | 0.135 | 0.001706 |
| sp|Q32MQ0|ZN750_HUMAN | 78149.78249 | 1.36 | 0.698 | 0.07644 |
| sp|Q8IY67|RAVR1_HUMAN | 64446.38648 | 1.31 | 0.136 | 3.24E-12 |
| sp|P52789|HXK2_HUMAN | 103720.994 | 0.7 | 0.095 | 6.31E-13 |
| sp|Q10472|GALT1_HUMAN | 65071.74558 | 0.55 | 0.088 | 7.66E-16 |
| sp|P0DOX8|IGL1_HUMAN | 23083.17961 | 1.59 | 0.399 | 6.96E-09 |
| sp|Q96I51|RCC1L_HUMAN | 50688.43757 | 0.97 | 0.097 | 0.08494 |
| sp|Q9ULD9|ZN608_HUMAN | 163116.7601 | 1.72 | 0.53 | 0.02022 |
| sp|Q96S90|LYSM1_HUMAN | 25026.94268 | 0.91 | 0.235 | 0.0196 |
| sp|O00154|BACH_HUMAN | 42435.5661 | 0.71 | 0.249 | 6.09E-06 |
| sp|Q8WXE0|CSKI2_HUMAN | 127257.64 | 0.47 | 0.073 | 2.20E-16 |
| sp|P22748|CAH4_HUMAN | 35276.99079 | 0.65 | 0.13 | 2.21E-11 |
| sp|P81133|SIM1_HUMAN | 86355.22934 | 1.03 | 0.341 | 0.7182 |
| sp|O15455|TLR3_HUMAN | 104714.1176 | 1.11 | 0.172 | 0.00958 |
| sp|P02647|APOA1_HUMAN | 30740.92196 | 1.27 | 0.515 | 0.0427 |
| sp|O15194|CTDSL_HUMAN | 31489.97612 | 0.73 | 0.104 | 6.99E-11 |
| sp|Q6ZNB6|NFXL1_HUMAN | 107695.3642 | 0.71 | 0.096 | 1.37E-12 |
| sp|O00151|PDLI1_HUMAN | 36487.20353 | 1.42 | 0.239 | 2.80E-10 |
| sp|P50395|GDIB_HUMAN | 51069.03878 | 0.84 | 0.114 | 8.39E-07 |
| sp|Q86VW0|SESD1_HUMAN | 80021.59419 | 1.2 | 0.162 | 7.16E-07 |
| sp|Q9Y5J9|TIM8B_HUMAN | 9547.611566 | 1.73 | 0.647 | 3.34E-06 |
| sp|O60603|TLR2_HUMAN | 90902.49332 | 0.73 | 0.209 | 2.50E-06 |
| sp|P35558|PCKGC_HUMAN | 69929.98694 | 1 | 0.333 | 0.3793 |
| sp|P48735|IDHP_HUMAN | 51315.02468 | 0.77 | 0.198 | 1.28E-05 |
| sp|Q9NSY0|NRBP2_HUMAN | 58261.56325 | 0.98 | 0.135 | 0.3831 |
| sp|Q96AC1|FERM2_HUMAN | 78419.83539 | 0.67 | 0.18 | 2.33E-08 |
| sp|Q9NRW7|VPS45_HUMAN | 65416.93648 | 0.74 | 0.061 | 9.76E-16 |
| sp|Q6ZMR5|TM11A_HUMAN | 47976.6245 | 2.35 | 1.44 | 3.91E-07 |
| sp|Q3MIR4|CC50B_HUMAN | 39354.51202 | 0.65 | 0.172 | 1.02E-08 |
| sp|Q9H944|MED20_HUMAN | 23644.58509 | 0.85 | 0.188 | 0.0002995 |
| sp|Q6YP21|KAT3_HUMAN | 51805.68624 | 0.78 | 0.239 | 6.12E-05 |
| sp|Q9BT78|CSN4_HUMAN | 46506.76761 | 1.22 | 0.098 | 9.79E-12 |
| sp|Q8IWE2|NXP20_HUMAN | 61028.24395 | 1.23 | 0.214 | 2.43E-05 |
| sp|P23526|SAHH_HUMAN | 48237.40789 | 0.62 | 0.111 | 4.41E-13 |
| sp|Q96LJ7|DHRS1_HUMAN | 34439.52247 | 1.05 | 0.323 | 0.9448 |
| sp|P14923|PLAK_HUMAN | 82415.99874 | 0.6 | 0.248 | 2.18E-07 |
| sp|Q8N5G0|SIM20_HUMAN | 7679.100651 | 0.81 | 0.176 | 8.65E-06 |
| sp|P42685|FRK_HUMAN | 58655.4223 | 0.88 | 0.195 | 0.002031 |
| sp|Q9H0R1|AP5M1_HUMAN | 55341.15099 | 0.78 | 0.082 | 1.85E-11 |
| sp|O15294|OGT1_HUMAN | 118086.3688 | 0.91 | 0.072 | 1.25E-06 |
| sp|Q92828|COR2A_HUMAN | 60220.5094 | 0.49 | 0.255 | 1.45E-08 |
| sp|Q9H9Q2|CSN7B_HUMAN | 29870.49615 | 1.2 | 0.182 | 1.24E-05 |
| sp|Q2M389|WASC4_HUMAN | 137324.5963 | 0.64 | 0.066 | 2.20E-16 |
| sp|Q03001|DYST_HUMAN | 865241.2438 | 0.96 | 0.123 | 0.07913 |
| sp|P31323|KAP3_HUMAN | 46654.07392 | 1.73 | 0.557 | 7.87E-08 |
| sp|P15531|NDKA_HUMAN | 17290.72507 | 0.96 | 0.245 | 0.1908 |
| sp|P31948|STIP1_HUMAN | 63208.63124 | 1.27 | 0.191 | 1.18E-07 |
| sp|O60331|PI51C_HUMAN | 73481.58785 | 1.53 | 0.486 | 2.11E-06 |
| sp|Q12929|EPS8_HUMAN | 92148.54189 | 1.08 | 0.18 | 0.06741 |
| sp|P01034|CYTC_HUMAN | 15999.15618 | 0.83 | 0.28 | 0.00512 |
| sp|Q8NEZ4|KMT2C_HUMAN | 548252.2121 | 1.09 | 0.225 | 0.08132 |
| sp|P19838|NFKB1_HUMAN | 105899.2558 | 1.23 | 0.112 | 5.28E-11 |
| sp|Q15386|UBE3C_HUMAN | 124909.0489 | 0.8 | 0.163 | 4.36E-06 |
| sp|P25445|TNR6_HUMAN | 38887.45114 | 1.12 | 0.141 | 0.0003562 |
| sp|P02743|SAMP_HUMAN | 25467.16322 | 0.89 | 0.464 | 0.02407 |
| sp|Q5T1B0|AXDN1_HUMAN | 118618.9275 | 1.26 | 0.528 | 0.1048 |
| sp|Q9NWV8|BABA1_HUMAN | 37031.80446 | 1.01 | 0.205 | 0.8803 |
| sp|Q92563|TICN2_HUMAN | 48156.42493 | 0.95 | 0.443 | 0.1156 |
| sp|Q8NAT1|PMGT2_HUMAN | 67067.74035 | 1.06 | 0.283 | 0.7229 |
| sp|Q9Y4X5|ARI1_HUMAN | 65882.45105 | 0.94 | 0.116 | 0.01098 |
| sp|O76038|SEGN_HUMAN | 32172.15893 | 0.48 | 0.258 | 7.87E-08 |
| sp|Q9NYH9|UTP6_HUMAN | 70758.0689 | 0.78 | 0.087 | 2.85E-11 |
| sp|Q9H0U4|RAB1B_HUMAN | 22310.28269 | 0.61 | 0.105 | 1.49E-13 |
| sp|P52888|THOP1_HUMAN | 79683.81091 | 1.14 | 0.126 | 1.12E-05 |
| sp|P50453|SPB9_HUMAN | 42985.53332 | 1.03 | 0.266 | 0.9984 |
| sp|Q99873|ANM1_HUMAN | 43043.16561 | 1.08 | 0.234 | 0.262 |
| sp|P08133|ANXA6_HUMAN | 76149.69496 | 1 | 0.143 | 0.757 |
| sp|Q9P2K5|MYEF2_HUMAN | 64290.59993 | 0.99 | 0.249 | 0.4679 |
| sp|Q53QZ3|RHG15_HUMAN | 54720.44368 | 0.68 | 0.178 | 3.20E-08 |
| sp|P11277|SPTB1_HUMAN | 247153.045 | 1.08 | 0.215 | 0.1356 |
| sp|Q9BYD1|RM13_HUMAN | 20717.69337 | 0.98 | 0.127 | 0.3554 |
| sp|Q13131|AAPK1_HUMAN | 64577.78345 | 0.87 | 0.13 | 4.00E-05 |
| sp|Q9UKK3|PARP4_HUMAN | 194564.1676 | 0.76 | 0.145 | 6.05E-08 |
| sp|Q86UT8|CCD84_HUMAN | 38445.11436 | 0.95 | 0.181 | 0.1073 |
| sp|Q9C0E2|XPO4_HUMAN | 131292.7114 | 0.77 | 0.089 | 3.39E-11 |
| sp|Q96HY7|DHTK1_HUMAN | 103734.4835 | 0.67 | 0.131 | 1.28E-10 |
| sp|P04179|SODM_HUMAN | 24887.63925 | 1.25 | 0.312 | 0.0009172 |
| sp|Q9NV06|DCA13_HUMAN | 51978.40203 | 1.08 | 0.142 | 0.02503 |
| sp|Q6IQ22|RAB12_HUMAN | 27555.20356 | 1.07 | 0.233 | 0.327 |
| sp|Q08211|DHX9_HUMAN | 142162.5919 | 0.68 | 0.085 | 5.78E-14 |
| sp|Q86VY4|TSYL5_HUMAN | 45326.03324 | 1.17 | 0.39 | 0.1953 |
| sp|O96019|ACL6A_HUMAN | 47925.52484 | 1.27 | 0.095 | 3.04E-14 |
| sp|Q9BPZ7|SIN1_HUMAN | 59524.14428 | 1.01 | 0.094 | 0.9453 |
| sp|Q13421|MSLN_HUMAN | 69608.4669 | 1.44 | 0.566 | 0.001665 |
| sp|Q03591|FHR1_HUMAN | 38748.38238 | 1.13 | 0.387 | 0.2814 |
| sp|Q96NB3|ZN830_HUMAN | 42126.3036 | 1.19 | 0.345 | 0.03192 |
| sp|Q01082|SPTB2_HUMAN | 275218.9747 | 1.03 | 0.251 | 0.9331 |
| sp|Q96AP7|ESAM_HUMAN | 41417.66956 | 1.16 | 0.142 | 5.86E-06 |
| sp|Q9H0A0|NAT10_HUMAN | 116550.9302 | 0.78 | 0.12 | 1.53E-08 |
| sp|P32969|RL9_HUMAN | 21945.82955 | 0.92 | 0.204 | 0.02136 |
| sp|Q9BSR8|YIPF4_HUMAN | 27104.32388 | 0.56 | 0.112 | 8.86E-14 |
| sp|Q5EBM0|CMPK2_HUMAN | 50139.87786 | 1.15 | 0.305 | 0.07832 |
| sp|P14406|CX7A2_HUMAN | 9372.095182 | 0.85 | 0.373 | 0.00933 |
| sp|O75376|NCOR1_HUMAN | 270938.7992 | 1.12 | 0.262 | 0.06173 |
| sp|Q96NB2|SFXN2_HUMAN | 36474.82699 | 0.7 | 0.205 | 6.22E-07 |
| sp|P43307|SSRA_HUMAN | 32197.33714 | 1.12 | 0.156 | 0.001663 |
| sp|Q13630|FCL_HUMAN | 36080.03523 | 0.76 | 0.11 | 4.45E-10 |
| sp|P14649|MYL6B_HUMAN | 22845.72614 | 1.68 | 0.351 | 2.38E-12 |
| sp|P08069|IGF1R_HUMAN | 157184.113 | 1.1 | 0.273 | 0.1677 |
| sp|Q12873|CHD3_HUMAN | 227971.1063 | 1.01 | 0.146 | 0.9359 |
| sp|Q13363|CTBP1_HUMAN | 47943.6562 | 1.05 | 0.094 | 0.01663 |
| sp|A0A0B4J1V0|HV315_HUMAN | 13070.53226 | 1.54 | 0.573 | 4.44E-05 |
| sp|Q8NDA8|MROH1_HUMAN | 183681.6897 | 0.86 | 0.167 | 0.0001145 |
| sp|Q9BRT9|SLD5_HUMAN | 26126.31265 | 1.28 | 0.249 | 8.76E-06 |
| sp|Q53GS7|GLE1_HUMAN | 80337.75412 | 0.8 | 0.102 | 1.51E-09 |
| sp|Q96C34|RUND1_HUMAN | 68096.0813 | 0.72 | 0.173 | 1.09E-07 |
| sp|Q6UN15|FIP1_HUMAN | 66582.56916 | 1.43 | 0.317 | 1.91E-08 |
| sp|Q8NGR3|OR1K1_HUMAN | 34740.97487 | 1.16 | 0.339 | 0.05439 |
| sp|Q3SXM5|HSDL1_HUMAN | 37359.26066 | 0.61 | 0.103 | 1.90E-13 |
| sp|Q4V328|GRAP1_HUMAN | 96270.98206 | 1.18 | 0.133 | 5.41E-07 |
| sp|Q6VMQ6|MCAF1_HUMAN | 137147.7049 | 1.11 | 0.295 | 0.193 |
| sp|O43490|PROM1_HUMAN | 98376.52577 | 0.46 | 0.246 | 7.22E-09 |
| sp|P57735|RAB25_HUMAN | 23577.02868 | 0.57 | 0.203 | 4.04E-09 |
| sp|P51178|PLCD1_HUMAN | 86334.26916 | 1 | 0.247 | 0.6095 |
| sp|O60307|MAST3_HUMAN | 143829.1026 | 1.82 | 0.474 | 5.57E-11 |
| sp|O15234|CASC3_HUMAN | 76327.57871 | 1.2 | 0.138 | 1.09E-07 |
| sp|A0A0B4J1Y9|HV372_HUMAN | 13347.55182 | 1.2 | 0.443 | 0.1241 |
| sp|O43570|CAH12_HUMAN | 39693.14355 | 1.11 | 0.423 | 0.5583 |
| sp|P50748|KNTC1_HUMAN | 253193.2594 | 1.23 | 0.173 | 2.14E-07 |
| sp|O00515|LAD1_HUMAN | 57135.85316 | 1.46 | 0.702 | 0.003632 |
| sp|O43815|STRN_HUMAN | 86516.94344 | 1.27 | 0.13 | 2.68E-11 |
| sp|Q96HP0|DOCK6_HUMAN | 231277.5785 | 0.86 | 0.091 | 9.99E-08 |
| sp|Q16706|MA2A1_HUMAN | 131723.5991 | 0.53 | 0.112 | 3.09E-14 |
| sp|P20023|CR2_HUMAN | 116302.0401 | 1.43 | 0.519 | 0.000382 |
| sp|Q9NYA4|MTMR4_HUMAN | 136271.7521 | 0.53 | 0.09 | 8.20E-16 |
| sp|Q7L5A8|FA2H_HUMAN | 42916.82289 | 0.71 | 0.274 | 3.22E-05 |
| sp|P78417|GSTO1_HUMAN | 27815.13487 | 1.05 | 0.172 | 0.2223 |
| sp|Q9NUW8|TYDP1_HUMAN | 68758.33143 | 0.7 | 0.14 | 5.62E-09 |
| sp|Q53RD9|FBLN7_HUMAN | 49037.35843 | 1.94 | 1.237 | 2.91E-05 |
| sp|Q969U6|FBXW5_HUMAN | 64491.28145 | 1.2 | 0.258 | 0.0009978 |
| sp|P46934|NEDD4_HUMAN | 150258.0098 | 1.06 | 0.137 | 0.05936 |
| sp|O60518|RNBP6_HUMAN | 126154.8953 | 0.62 | 0.146 | 9.65E-11 |
| sp|P42126|ECI1_HUMAN | 33062.33458 | 0.64 | 0.203 | 1.17E-07 |
| sp|P02538|K2C6A_HUMAN | 60275.37106 | 1.24 | 0.643 | 0.2101 |
| sp|P63244|RACK1_HUMAN | 35492.71913 | 0.75 | 0.12 | 2.90E-09 |
| sp|O14757|CHK1_HUMAN | 54951.10607 | 1.11 | 0.258 | 0.1153 |
| sp|P04156|PRIO_HUMAN | 27853.27156 | 1.08 | 0.331 | 0.6444 |
| sp|P25098|ARBK1_HUMAN | 80302.68957 | 0.84 | 0.189 | 0.0001752 |
| sp|A1A5B4|ANO9_HUMAN | 91168.96912 | 0.5 | 0.221 | 2.97E-09 |
| sp|P30838|AL3A1_HUMAN | 50743.98601 | 1.05 | 0.54 | 0.3311 |
| sp|Q14683|SMC1A_HUMAN | 143753.3428 | 1.14 | 0.126 | 7.83E-06 |
| sp|Q9UBC5|MYO1A_HUMAN | 119220.1224 | 0.46 | 0.177 | 6.42E-11 |
| sp|P56181|NDUV3_HUMAN | 11972.07411 | 1.29 | 0.501 | 0.0132 |
| sp|A3KN83|SBNO1_HUMAN | 154882.2964 | 0.81 | 0.183 | 4.27E-05 |
| sp|Q7L1V2|MON1B_HUMAN | 59618.41385 | 0.79 | 0.184 | 8.34E-06 |
| sp|Q8TDH9|BL1S5_HUMAN | 21748.88382 | 1.54 | 0.232 | 4.97E-13 |
| sp|O60749|SNX2_HUMAN | 58530.69069 | 1.19 | 0.121 | 2.47E-08 |
| sp|P11532|DMD_HUMAN | 428519.0409 | 0.78 | 0.291 | 0.0001733 |
| sp|Q9BQ15|SOSB1_HUMAN | 22477.24129 | 0.91 | 0.145 | 0.002449 |
| sp|P25786|PSA1_HUMAN | 29803.95744 | 1.14 | 0.17 | 0.0002746 |
| sp|Q92831|KAT2B_HUMAN | 94019.11278 | 1.1 | 0.258 | 0.1965 |
| sp|P05543|THBG_HUMAN | 46618.72571 | 1.24 | 0.315 | 0.0012 |
| sp|P38919|IF4A3_HUMAN | 47108.2789 | 0.88 | 0.138 | 0.0001978 |
| sp|Q9BXI6|TB10A_HUMAN | 57576.51669 | 1.02 | 0.11 | 0.415 |
| sp|Q9HAU8|RNPL1_HUMAN | 81071.51442 | 0.82 | 0.128 | 1.68E-06 |
| sp|P43250|GRK6_HUMAN | 67185.01157 | 0.75 | 0.092 | 1.57E-11 |
| sp|P04839|CY24B_HUMAN | 66187.92142 | 0.54 | 0.192 | 1.82E-09 |
| sp|Q8NFZ5|TNIP2_HUMAN | 49221.6702 | 1.17 | 0.16 | 5.07E-05 |
| sp|Q6NUK1|SCMC1_HUMAN | 53530.41815 | 0.59 | 0.16 | 2.66E-10 |
| sp|Q96HH4|TM169_HUMAN | 33970.67822 | 3.46 | 1.576 | 1.58E-10 |
| sp|Q9BW91|NUDT9_HUMAN | 39253.7445 | 1.34 | 0.159 | 2.59E-11 |
| sp|O95881|TXD12_HUMAN | 19346.63374 | 1.24 | 0.139 | 1.00E-09 |
| sp|O14756|H17B6_HUMAN | 36266.621 | 0.73 | 0.225 | 2.89E-06 |
| sp|Q9UBU8|MO4L1_HUMAN | 41543.65693 | 0.87 | 0.288 | 0.01077 |
| sp|P60891|PRPS1_HUMAN | 35307.1104 | 0.67 | 0.089 | 5.49E-14 |
| sp|P51531|SMCA2_HUMAN | 181775.522 | 0.9 | 0.176 | 0.003749 |
| sp|P62888|RL30_HUMAN | 12928.79543 | 1.38 | 0.176 | 8.02E-12 |
| sp|P40926|MDHM_HUMAN | 35918.8938 | 1.08 | 0.214 | 0.1579 |
| sp|Q8TDV5|GP119_HUMAN | 37416.55557 | 1.05 | 0.145 | 0.2207 |
| sp|Q96B45|BORC7_HUMAN | 11727.03836 | 0.91 | 0.394 | 0.04744 |
| sp|P21953|ODBB_HUMAN | 43761.14454 | 0.67 | 0.341 | 2.41E-05 |
| sp|P23497|SP100_HUMAN | 101476.5471 | 1.77 | 0.314 | 2.12E-13 |
| sp|Q96QI5|HS3S6_HUMAN | 37486.62124 | 0.57 | 0.179 | 2.15E-10 |
| sp|P22083|FUT4_HUMAN | 59485.17932 | 0.93 | 0.216 | 0.03569 |
| sp|O00425|IF2B3_HUMAN | 63989.68007 | 1.39 | 0.687 | 0.04418 |
| sp|Q05D32|CTSL2_HUMAN | 53119.19543 | 0.43 | 0.094 | 2.80E-16 |
| sp|O75955|FLOT1_HUMAN | 47535.69398 | 0.82 | 0.058 | 3.20E-13 |
| sp|Q5TGY1|TMCO4_HUMAN | 68647.13065 | 0.91 | 0.165 | 0.007417 |
| sp|Q9UI08|EVL_HUMAN | 44688.24969 | 1.88 | 0.504 | 7.53E-11 |
| sp|Q9Y6N7|ROBO1_HUMAN | 181940.0446 | 1.09 | 0.133 | 0.004328 |
| sp|O95104|SFR15_HUMAN | 126114.3557 | 0.84 | 0.086 | 2.76E-09 |
| sp|Q63ZY3|KANK2_HUMAN | 91898.16785 | 1.18 | 0.426 | 0.09919 |
| sp|P22760|AAAD_HUMAN | 45800.85452 | 0.8 | 0.398 | 0.003105 |
| sp|Q9UGC7|RF1ML_HUMAN | 43668.80978 | 0.59 | 0.164 | 1.32E-08 |
| sp|P02765|FETUA_HUMAN | 40095.97746 | 1.32 | 0.259 | 7.59E-07 |
| sp|O00295|TULP2_HUMAN | 59065.53375 | 0.69 | 0.272 | 9.86E-06 |
| sp|Q02978|M2OM_HUMAN | 34192.90513 | 0.47 | 0.132 | 2.19E-13 |
| sp|P49747|COMP_HUMAN | 85412.59322 | 2.19 | 1.013 | 1.16E-07 |
| sp|P00325|ADH1B_HUMAN | 40665.90789 | 1.65 | 0.66 | 6.67E-05 |
| sp|P00441|SODC_HUMAN | 16135.97913 | 1.2 | 0.155 | 4.56E-07 |
| sp|P60983|GMFB_HUMAN | 16855.5396 | 1.51 | 0.225 | 5.02E-13 |
| sp|Q9NXH8|TOR4A_HUMAN | 47323.09691 | 0.98 | 0.128 | 0.3361 |
| sp|Q96HR9|REEP6_HUMAN | 23556.3689 | 0.72 | 0.16 | 1.29E-08 |
| sp|P67812|SC11A_HUMAN | 20594.08275 | 0.72 | 0.072 | 7.11E-15 |
| sp|Q96JH7|VCIP1_HUMAN | 135586.3561 | 1.04 | 0.086 | 0.03514 |
| sp|Q14839|CHD4_HUMAN | 219388.6427 | 1.01 | 0.179 | 0.8256 |
| sp|P51649|SSDH_HUMAN | 58015.62233 | 0.78 | 0.211 | 3.25E-05 |
| sp|P15586|GNS_HUMAN | 62822.3275 | 1.26 | 0.191 | 1.88E-07 |
| sp|Q6IAA8|LTOR1_HUMAN | 17829.86327 | 1.17 | 0.081 | 5.80E-11 |
| sp|Q9Y678|COPG1_HUMAN | 98948.94958 | 0.76 | 0.139 | 2.20E-08 |
| sp|Q15311|RBP1_HUMAN | 76397.32627 | 0.92 | 0.179 | 0.01239 |
| sp|P55916|UCP3_HUMAN | 34631.6282 | 1.42 | 0.376 | 3.70E-06 |
| sp|P50148|GNAQ_HUMAN | 42382.41116 | 0.62 | 0.143 | 1.68E-10 |
| sp|Q6WCQ1|MPRIP_HUMAN | 117241.7661 | 1.51 | 0.195 | 1.61E-14 |
| sp|Q15906|VPS72_HUMAN | 40779.73122 | 1.1 | 0.138 | 0.001474 |
| sp|P46019|KPB2_HUMAN | 139385.84 | 0.81 | 0.134 | 9.46E-07 |
| sp|P20749|BCL3_HUMAN | 47934.68844 | 1.11 | 0.307 | 0.2325 |
| sp|O76024|WFS1_HUMAN | 101349.3699 | 0.6 | 0.177 | 3.82E-09 |
| sp|Q9H0V1|TM168_HUMAN | 80938.74448 | 0.65 | 0.075 | 1.01E-15 |
| sp|O60832|DKC1_HUMAN | 58075.94664 | 0.64 | 0.098 | 1.11E-13 |
| sp|O75995|SASH3_HUMAN | 41722.82768 | 1.16 | 0.162 | 3.02E-05 |
| sp|Q15834|CC85B_HUMAN | 22401.2518 | 1.23 | 0.191 | 1.29E-06 |
| sp|Q9UKU7|ACAD8_HUMAN | 45877.78259 | 0.97 | 0.311 | 0.2495 |
| sp|Q7Z739|YTHD3_HUMAN | 63917.79623 | 1.15 | 0.298 | 0.06546 |
| sp|Q96RY7|IF140_HUMAN | 166837.1337 | 0.94 | 0.161 | 0.02756 |
| sp|O15446|RPA34_HUMAN | 55218.46308 | 1.07 | 0.546 | 0.5865 |
| sp|O75151|PHF2_HUMAN | 121651.0814 | 0.88 | 0.127 | 9.39E-05 |
| sp|Q9HC62|SENP2_HUMAN | 68591.97419 | 0.6 | 0.24 | 3.88E-07 |
| sp|Q9H5V8|CDCP1_HUMAN | 94279.88948 | 0.54 | 0.188 | 1.91E-08 |
| sp|Q6ZNC4|ZN704_HUMAN | 45799.64344 | 0.52 | 0.112 | 1.15E-13 |
| sp|O15551|CLD3_HUMAN | 23798.31814 | 0.79 | 0.459 | 0.0065 |
| sp|Q14146|URB2_HUMAN | 172355.3775 | 1 | 0.169 | 0.6608 |
| sp|O00303|EIF3F_HUMAN | 37636.18384 | 0.72 | 0.101 | 1.31E-11 |
| sp|P51948|MAT1_HUMAN | 36238.46275 | 1.27 | 0.184 | 8.80E-08 |
| sp|Q8IWT0|ARCH_HUMAN | 19517.43822 | 1.12 | 0.214 | 0.0237 |
| sp|Q92797|SYMPK_HUMAN | 141896.5161 | 0.86 | 0.063 | 8.76E-11 |
| sp|Q9UBX1|CATF_HUMAN | 53941.02867 | 1.32 | 0.673 | 0.0636 |
| sp|P62993|GRB2_HUMAN | 25286.46623 | 1.45 | 0.2 | 3.52E-13 |
| sp|P16278|BGAL_HUMAN | 76464.73368 | 0.7 | 0.108 | 1.26E-11 |
| sp|P27658|CO8A1_HUMAN | 73413.15712 | 3.36 | 2.296 | 4.71E-07 |
| sp|Q9BT73|PSMG3_HUMAN | 13249.11639 | 0.52 | 0.05 | 2.20E-16 |
| sp|P36405|ARL3_HUMAN | 20595.81283 | 1.36 | 0.144 | 6.26E-13 |
| sp|O75531|BAF_HUMAN | 10262.09884 | 1.26 | 0.264 | 5.38E-05 |
| sp|P07199|CENPB_HUMAN | 65512.69563 | 0.78 | 0.258 | 0.0001339 |
| sp|Q9HD15|SRA1_HUMAN | 25809.98421 | 1.34 | 0.294 | 5.49E-06 |
| sp|O15397|IPO8_HUMAN | 120926.6576 | 0.57 | 0.092 | 3.47E-15 |
| sp|P40200|TACT_HUMAN | 66372.15371 | 1.04 | 0.161 | 0.4158 |
| sp|Q86VH2|KIF27_HUMAN | 161306.2333 | 1.43 | 0.393 | 1.25E-06 |
| sp|P54259|ATN1_HUMAN | 125490.6503 | 1.69 | 0.24 | 4.74E-15 |
| sp|Q9BXP5|SRRT_HUMAN | 101042.4697 | 1.2 | 0.164 | 5.01E-07 |
| sp|O14497|ARI1A_HUMAN | 242786.5596 | 0.88 | 0.131 | 0.0001245 |
| sp|O75170|PP6R2_HUMAN | 106340.2785 | 0.8 | 0.153 | 1.38E-06 |
| sp|Q4KMQ2|ANO6_HUMAN | 107161.647 | 0.71 | 0.123 | 1.98E-10 |
| sp|P61758|PFD3_HUMAN | 22796.49841 | 1.5 | 0.249 | 1.98E-11 |
| sp|Q8IX12|CCAR1_HUMAN | 133405.3266 | 1.12 | 0.148 | 0.0004812 |
| sp|Q7Z7A4|PXK_HUMAN | 65461.14142 | 0.78 | 0.269 | 4.47E-05 |
| sp|Q13227|GPS2_HUMAN | 36647.49558 | 1.22 | 0.085 | 4.92E-13 |
| sp|Q96CG8|CTHR1_HUMAN | 26759.33401 | 1.37 | 0.254 | 1.83E-08 |
| sp|Q9BZJ0|CRNL1_HUMAN | 100884.3117 | 0.65 | 0.145 | 7.16E-10 |
| sp|Q9Y5V0|ZN706_HUMAN | 8588.479495 | 1.36 | 0.237 | 3.05E-08 |
| sp|Q9NSD9|SYFB_HUMAN | 66682.77246 | 0.78 | 0.12 | 1.69E-08 |
| sp|O14531|DPYL4_HUMAN | 62447.63327 | 1.98 | 0.64 | 6.27E-10 |
| sp|Q9BZK7|TBL1R_HUMAN | 56169.30504 | 1.14 | 0.103 | 1.10E-07 |
| sp|Q6P1X6|CH082_HUMAN | 24255.23464 | 0.8 | 0.17 | 8.00E-06 |
| sp|P57105|SYJ2B_HUMAN | 15900.1489 | 0.62 | 0.112 | 1.47E-12 |
| sp|Q8TEM1|PO210_HUMAN | 205877.0461 | 0.65 | 0.102 | 2.74E-13 |
| sp|Q9Y3L5|RAP2C_HUMAN | 20941.47016 | 1.1 | 0.309 | 0.2698 |
| sp|Q9BR76|COR1B_HUMAN | 54866.49477 | 0.94 | 0.167 | 0.04483 |
| sp|Q8WUY3|PRUN2_HUMAN | 342516.7032 | 0.91 | 0.365 | 0.04196 |
| sp|O43665|RGS10_HUMAN | 20318.98908 | 1.92 | 0.332 | 3.82E-15 |
| sp|Q8WU39|MZB1_HUMAN | 21005.34518 | 1.36 | 0.506 | 0.002532 |
| sp|P17980|PRS6A_HUMAN | 49439.55317 | 1.36 | 0.089 | 2.20E-16 |
| sp|Q66K14|TBC9B_HUMAN | 141558.0569 | 0.88 | 0.101 | 2.90E-06 |
| sp|Q8N4Q1|MIA40_HUMAN | 16367.19999 | 2.62 | 0.914 | 9.77E-11 |
| sp|Q13526|PIN1_HUMAN | 18328.00262 | 1.77 | 0.458 | 1.37E-10 |
| sp|Q9HB21|PKHA1_HUMAN | 45906.03886 | 0.81 | 0.144 | 2.79E-06 |
| sp|P26640|SYVC_HUMAN | 141623.8358 | 0.75 | 0.086 | 5.47E-12 |
| sp|Q8NHU6|TDRD7_HUMAN | 125027.6733 | 0.98 | 0.198 | 0.3213 |
| sp|Q9BUI4|RPC3_HUMAN | 60954.37649 | 0.86 | 0.135 | 2.90E-05 |
| sp|P09132|SRP19_HUMAN | 16355.50685 | 1.6 | 0.47 | 1.07E-07 |
| sp|Q5T0N5|FBP1L_HUMAN | 70459.6567 | 1.26 | 0.233 | 2.41E-06 |
| sp|P62633|CNBP_HUMAN | 20686.02527 | 1.37 | 0.191 | 6.57E-11 |
| sp|Q6ZMI0|PPR21_HUMAN | 88754.71293 | 0.96 | 0.043 | 2.72E-05 |
| sp|P49755|TMEDA_HUMAN | 25113.07695 | 0.72 | 0.171 | 4.83E-08 |
| sp|P54105|ICLN_HUMAN | 26351.97232 | 1.25 | 0.186 | 1.56E-07 |
| sp|Q8IZA0|K319L_HUMAN | 116651.0121 | 0.43 | 0.173 | 7.49E-12 |
| sp|Q92503|S14L1_HUMAN | 82035.36213 | 0.95 | 0.178 | 0.09243 |
| sp|Q6GPI1|CTRB2_HUMAN | 28514.34184 | 0.61 | 0.238 | 3.67E-07 |
| sp|P55345|ANM2_HUMAN | 49562.5083 | 0.92 | 0.126 | 0.002671 |
| sp|P18084|ITB5_HUMAN | 91285.44562 | 0.93 | 0.181 | 0.02306 |
| sp|Q15013|MD2BP_HUMAN | 31526.75818 | 0.69 | 0.153 | 4.16E-09 |
| sp|Q8TCC3|RM30_HUMAN | 18630.11441 | 1.12 | 0.181 | 0.003983 |
| sp|Q9UQ16|DYN3_HUMAN | 98066.09315 | 0.77 | 0.149 | 4.38E-07 |
| sp|Q95IE3|2B1C_HUMAN | 30183.18606 | 1.59 | 0.632 | 4.60E-05 |
| sp|P19388|RPAB1_HUMAN | 24631.73445 | 1.05 | 0.076 | 0.005748 |
| sp|Q96CD0|FBXL8_HUMAN | 41384.57259 | 1.01 | 0.144 | 0.9332 |
| sp|P17927|CR1_HUMAN | 230455.4563 | 0.66 | 0.371 | 4.18E-05 |
| sp|Q5VTU8|AT5EL_HUMAN | 5842.201873 | 1.07 | 0.137 | 0.02164 |
| sp|P41970|ELK3_HUMAN | 44365.18133 | 1.08 | 0.137 | 0.01055 |
| sp|Q13206|DDX10_HUMAN | 101149.576 | 0.93 | 0.181 | 0.02829 |
| sp|Q13477|MADCA_HUMAN | 40511.62637 | 1.22 | 0.26 | 0.0003196 |
| sp|Q08499|PDE4D_HUMAN | 91610.12282 | 0.79 | 0.253 | 0.0002131 |
| sp|Q7Z434|MAVS_HUMAN | 57045.2937 | 1.09 | 0.208 | 0.06179 |
| sp|P23588|IF4B_HUMAN | 69149.29637 | 1.46 | 0.264 | 2.41E-10 |
| sp|Q96K19|RN170_HUMAN | 30233.34734 | 0.63 | 0.169 | 5.82E-09 |
| sp|Q8WW12|PCNP_HUMAN | 18895.38927 | 1.68 | 0.508 | 1.13E-07 |
| sp|Q08116|RGS1_HUMAN | 23996.11135 | 1.42 | 0.763 | 0.05373 |
| sp|P39748|FEN1_HUMAN | 42890.22055 | 0.93 | 0.21 | 0.05457 |
| sp|Q00613|HSF1_HUMAN | 57491.84593 | 1.15 | 0.19 | 0.001368 |
| sp|P23634|AT2B4_HUMAN | 139012.2255 | 0.64 | 0.214 | 6.45E-08 |
| sp|P26927|HGFL_HUMAN | 82815.48079 | 1.22 | 0.39 | 0.0454 |
| sp|P40939|ECHA_HUMAN | 83670.14266 | 1.15 | 0.191 | 0.001213 |
| sp|Q9UPV0|CE164_HUMAN | 164709.4367 | 1.2 | 0.336 | 0.02075 |
| sp|O60575|ISK4_HUMAN | 9771.876973 | 0.43 | 0.278 | 1.76E-07 |
| sp|O15120|PLCB_HUMAN | 31275.26322 | 0.92 | 0.119 | 0.002297 |
| sp|Q05519|SRS11_HUMAN | 53606.20984 | 1.22 | 0.134 | 9.87E-09 |
| sp|Q5JSP0|FGD3_HUMAN | 80188.39284 | 1.07 | 0.309 | 0.7272 |
| sp|Q96RU3|FNBP1_HUMAN | 71700.46886 | 1.43 | 0.27 | 2.67E-09 |
| sp|Q9BTZ2|DHRS4_HUMAN | 29785.4748 | 1.05 | 0.121 | 0.09307 |
| sp|O00764|PDXK_HUMAN | 35289.98723 | 1.26 | 0.13 | 1.88E-10 |
| sp|Q30201|HFE_HUMAN | 40349.47216 | 0.82 | 0.13 | 5.19E-07 |
| sp|Q9H814|PHAX_HUMAN | 44528.15433 | 1.3 | 0.327 | 0.0001623 |
| sp|Q96MW1|CCD43_HUMAN | 25271.97025 | 1.39 | 0.269 | 2.60E-08 |
| sp|Q9NX55|HYPK_HUMAN | 14638.37096 | 1.32 | 0.128 | 4.54E-13 |
| sp|Q9NWH9|SLTM_HUMAN | 117345.6142 | 1.41 | 0.199 | 1.58E-11 |
| sp|P50990|TCPQ_HUMAN | 60134.71018 | 0.89 | 0.116 | 2.47E-05 |
| sp|Q6ZSR9|YJ005_HUMAN | 38162.65201 | 1.31 | 0.139 | 9.74E-12 |
| sp|O15037|KHNYN_HUMAN | 75210.64698 | 0.89 | 0.136 | 0.001144 |
| sp|Q8IU85|KCC1D_HUMAN | 43267.83152 | 0.88 | 0.219 | 0.007117 |
| sp|O43772|MCAT_HUMAN | 33246.21673 | 0.53 | 0.103 | 2.92E-14 |
| sp|P05160|F13B_HUMAN | 77724.01434 | 0.85 | 0.312 | 0.003406 |
| sp|Q9Y383|LC7L2_HUMAN | 46923.7525 | 1.13 | 0.108 | 2.06E-06 |
| sp|Q9ULW0|TPX2_HUMAN | 86209.21462 | 1.18 | 0.527 | 0.4534 |
| sp|Q00587|BORG5_HUMAN | 40366.07222 | 1.21 | 0.094 | 1.53E-11 |
| sp|Q9GZM7|TINAL_HUMAN | 53703.45805 | 1.09 | 0.361 | 0.4978 |
| sp|Q96FK6|WDR89_HUMAN | 43853.43216 | 1.39 | 0.205 | 3.35E-11 |
| sp|P55061|BI1_HUMAN | 26786.99217 | 0.28 | 0.086 | 2.20E-16 |
| sp|Q9GZZ9|UBA5_HUMAN | 45272.49254 | 0.86 | 0.129 | 4.85E-05 |
| sp|Q6UWP7|LCLT1_HUMAN | 49326.41504 | 0.9 | 0.135 | 0.000537 |
| sp|Q9UBP9|GULP1_HUMAN | 34906.60236 | 1.29 | 0.358 | 0.0004302 |
| sp|Q9H6E4|CC134_HUMAN | 26582.96834 | 0.64 | 0.297 | 2.99E-06 |
| sp|Q96AM1|MRGRF_HUMAN | 39154.08287 | 0.4 | 0.228 | 8.72E-10 |
| sp|P04271|S100B_HUMAN | 10802.08211 | 2.28 | 0.968 | 6.13E-08 |
| sp|P14316|IRF2_HUMAN | 39482.87345 | 1.34 | 0.293 | 2.35E-06 |
| sp|Q9Y3B2|EXOS1_HUMAN | 21762.10225 | 0.94 | 0.206 | 0.05789 |
| sp|P55084|ECHB_HUMAN | 51528.6185 | 1.34 | 0.196 | 9.02E-10 |
| sp|Q92636|FAN_HUMAN | 105314.2946 | 1 | 0.212 | 0.5924 |
| sp|P13987|CD59_HUMAN | 14777.01854 | 0.55 | 0.12 | 2.23E-13 |
| sp|Q8WVC6|DCAKD_HUMAN | 26629.34645 | 0.8 | 0.156 | 4.12E-06 |
| sp|Q9NPJ3|ACO13_HUMAN | 15046.89048 | 1.26 | 0.383 | 0.005879 |
| sp|Q9UIW2|PLXA1_HUMAN | 214108.1281 | 0.79 | 0.065 | 1.54E-13 |
| sp|O14734|ACOT8_HUMAN | 36329.19211 | 0.66 | 0.2 | 4.17E-07 |
| sp|Q6ZV73|FGD6_HUMAN | 162578.1981 | 1.18 | 0.43 | 0.1777 |
| sp|Q13158|FADD_HUMAN | 23474.9209 | 1.17 | 0.198 | 0.0005315 |
| sp|Q8WUY1|THEM6_HUMAN | 24116.96606 | 0.68 | 0.16 | 4.70E-09 |
| sp|Q12962|TAF10_HUMAN | 21793.8898 | 0.97 | 0.381 | 0.1784 |
| sp|Q8IYB5|SMAP1_HUMAN | 50678.46609 | 0.79 | 0.144 | 3.41E-07 |
| sp|O60716|CTND1_HUMAN | 108655.5811 | 0.87 | 0.164 | 0.0004353 |
| sp|O75688|PPM1B_HUMAN | 53161.5678 | 1.2 | 0.117 | 6.47E-10 |
| sp|Q8TC12|RDH11_HUMAN | 35744.58477 | 0.73 | 0.127 | 1.09E-09 |
| sp|Q6P2C8|MED27_HUMAN | 35619.40039 | 0.69 | 0.131 | 2.41E-10 |
| sp|Q16254|E2F4_HUMAN | 44257.42294 | 1.13 | 0.169 | 0.0006164 |
| sp|Q9Y672|ALG6_HUMAN | 59261.00251 | 0.69 | 0.221 | 9.33E-07 |
| sp|P07741|APT_HUMAN | 19748.48607 | 1.06 | 0.199 | 0.3064 |
| sp|Q8TBA6|GOGA5_HUMAN | 83012.83324 | 0.88 | 0.166 | 0.0008392 |
| sp|Q9UPZ3|HPS5_HUMAN | 129289.0234 | 0.92 | 0.277 | 0.03249 |
| sp|Q9UDY2|ZO2_HUMAN | 134086.352 | 0.93 | 0.219 | 0.05832 |
| sp|P29353|SHC1_HUMAN | 63277.67744 | 1.03 | 0.091 | 0.2248 |
| sp|P55058|PLTP_HUMAN | 54914.61821 | 0.7 | 0.207 | 5.69E-07 |
| sp|Q8WWA0|ITLN1_HUMAN | 35491.50652 | 0.72 | 0.421 | 0.0004123 |
| sp|O14815|CAN9_HUMAN | 79712.95526 | 0.85 | 0.327 | 0.01857 |
| sp|Q99733|NP1L4_HUMAN | 42949.94471 | 1.5 | 0.305 | 2.75E-09 |
| sp|P07737|PROF1_HUMAN | 15197.60988 | 1.19 | 0.202 | 0.000155 |
| sp|P23508|CRCM_HUMAN | 93806.97821 | 1.12 | 0.204 | 0.03323 |
| sp|Q15596|NCOA2_HUMAN | 159892.9131 | 1.01 | 0.216 | 0.8061 |
| sp|Q9NWU2|GID8_HUMAN | 26770.66852 | 1.05 | 0.196 | 0.4206 |
| sp|Q9NP66|HM20A_HUMAN | 40101.05232 | 1.21 | 0.359 | 0.01508 |
| sp|O75489|NDUS3_HUMAN | 30318.7448 | 0.92 | 0.228 | 0.04183 |
| sp|Q9NV56|MRGBP_HUMAN | 22556.03592 | 1.4 | 0.435 | 5.29E-05 |
| sp|P01624|KV315_HUMAN | 12584.23996 | 1.32 | 0.323 | 1.79E-05 |
| sp|Q7Z5Q1|CPEB2_HUMAN | 65626.05048 | 1.12 | 0.218 | 0.01023 |
| sp|Q5TCQ9|MAGI3_HUMAN | 163742.5012 | 1.04 | 0.294 | 0.955 |
| sp|P31431|SDC4_HUMAN | 21610.02078 | 0.68 | 0.377 | 4.53E-05 |
| sp|Q7Z2K6|ERMP1_HUMAN | 101004.6805 | 0.52 | 0.184 | 9.29E-10 |
| sp|Q9H3H5|GPT_HUMAN | 46668.72399 | 0.76 | 0.106 | 2.27E-10 |
| sp|P04155|TFF1_HUMAN | 9581.362933 | 0.97 | 0.629 | 0.08728 |
| sp|P06280|AGAL_HUMAN | 49458.14032 | 0.89 | 0.167 | 0.002117 |
| sp|Q9HD26|GOPC_HUMAN | 50869.94097 | 1.43 | 0.279 | 3.20E-09 |
| sp|Q7L099|RUFY3_HUMAN | 53198.23577 | 1.03 | 0.15 | 0.4504 |
| sp|Q96HW7|INT4_HUMAN | 109053.4593 | 1.04 | 0.379 | 0.8084 |
| sp|Q8TDG4|HELQ_HUMAN | 125289.2348 | 0.73 | 0.089 | 1.60E-12 |
| sp|Q6ZSZ5|ARHGI_HUMAN | 152557.5023 | 0.94 | 0.163 | 0.02922 |
| sp|Q10469|MGAT2_HUMAN | 52183.57036 | 0.45 | 0.204 | 1.36E-07 |
| sp|Q00536|CDK16_HUMAN | 55891.05152 | 0.75 | 0.091 | 1.44E-11 |
| sp|P19793|RXRA_HUMAN | 51501.76372 | 0.71 | 0.049 | 2.20E-16 |
| sp|Q7L5L3|GDPD3_HUMAN | 36953.86582 | 0.55 | 0.305 | 1.73E-06 |
| sp|Q96P70|IPO9_HUMAN | 116840.092 | 0.64 | 0.082 | 2.15E-15 |
| sp|P19404|NDUV2_HUMAN | 27641.11224 | 1.26 | 0.251 | 2.73E-05 |
| sp|Q9BTC8|MTA3_HUMAN | 68184.26133 | 1.13 | 0.194 | 0.00675 |
| sp|O60884|DNJA2_HUMAN | 46325.81613 | 1.21 | 0.174 | 1.32E-06 |
| sp|Q92947|GCDH_HUMAN | 48591.62214 | 0.76 | 0.225 | 3.96E-05 |
| sp|Q96BY7|ATG2B_HUMAN | 234479.3644 | 0.88 | 0.111 | 2.42E-05 |
| sp|O75964|ATP5L_HUMAN | 11403.23246 | 0.45 | 0.146 | 1.95E-12 |
| sp|Q96EP5|DAZP1_HUMAN | 43566.13115 | 1.54 | 0.234 | 2.49E-13 |
| sp|Q8NI36|WDR36_HUMAN | 106263.539 | 0.74 | 0.096 | 2.49E-11 |
| sp|O95837|GNA14_HUMAN | 41982.34499 | 0.76 | 0.202 | 3.99E-06 |
| sp|A4D1E9|GTPBA_HUMAN | 43172.71804 | 0.77 | 0.127 | 3.87E-08 |
| sp|Q8WV41|SNX33_HUMAN | 65547.29244 | 0.91 | 0.161 | 0.008689 |
| sp|P78356|PI42B_HUMAN | 47728.90663 | 0.84 | 0.1 | 3.96E-08 |
| sp|O75147|OBSL1_HUMAN | 209878.3944 | 1.23 | 0.333 | 0.00504 |
| sp|Q15382|RHEB_HUMAN | 20523.52078 | 0.75 | 0.095 | 1.01E-11 |
| sp|O00712|NFIB_HUMAN | 47736.08741 | 1.26 | 0.504 | 0.01902 |
| sp|Q15041|AR6P1_HUMAN | 23500.48797 | 0.39 | 0.106 | 1.75E-15 |
| sp|Q7LBR1|CHM1B_HUMAN | 22134.18014 | 1.5 | 0.303 | 1.22E-09 |
| sp|O76021|RL1D1_HUMAN | 55149.00523 | 0.91 | 0.152 | 0.004716 |
| sp|P13612|ITA4_HUMAN | 116234.0764 | 1.08 | 0.134 | 0.008307 |
| sp|Q14241|ELOA1_HUMAN | 90291.43805 | 1.24 | 0.302 | 0.0006278 |
| sp|O00187|MASP2_HUMAN | 77175.19184 | 0.77 | 0.331 | 0.0005588 |
| sp|P24071|FCAR_HUMAN | 32682.3697 | 0.71 | 0.437 | 0.0003859 |
| sp|Q8NEF9|SRFB1_HUMAN | 48700.45152 | 1.78 | 0.49 | 1.99E-09 |
| sp|Q96C86|DCPS_HUMAN | 38738.0739 | 1.07 | 0.091 | 0.001559 |
| sp|Q96I34|PP16A_HUMAN | 58384.50846 | 1.15 | 0.217 | 0.002677 |
| sp|P53674|CRBB1_HUMAN | 28044.89907 | 1.95 | 0.466 | 1.61E-13 |
| sp|P56470|LEG4_HUMAN | 36014.2168 | 0.41 | 0.16 | 2.13E-11 |
| sp|Q99519|NEUR1_HUMAN | 45933.81645 | 0.96 | 0.222 | 0.1958 |
| sp|O60234|GMFG_HUMAN | 16943.48374 | 1.27 | 0.191 | 7.84E-08 |
| sp|P43034|LIS1_HUMAN | 47160.36176 | 1.1 | 0.119 | 0.000467 |
| sp|Q9Y3I0|RTCB_HUMAN | 55670.03872 | 1.26 | 0.1 | 2.77E-13 |
| sp|O94832|MYO1D_HUMAN | 116908.8399 | 0.59 | 0.14 | 6.45E-11 |
| sp|Q8TBZ2|MYBPP_HUMAN | 108580.3283 | 1.49 | 0.245 | 9.86E-12 |
| sp|P04732|MT1E_HUMAN | 7131.619888 | 1.29 | 0.9 | 0.9466 |
| sp|Q8TDY2|RBCC1_HUMAN | 185067.107 | 1.11 | 0.369 | 0.3785 |
| sp|Q9BYZ8|REG4_HUMAN | 18598.96494 | 0.56 | 0.121 | 3.69E-12 |
| sp|Q96SI9|STRBP_HUMAN | 74272.10807 | 0.88 | 0.299 | 0.007741 |
| sp|P16615|AT2A2_HUMAN | 116318.1713 | 0.48 | 0.077 | 2.20E-16 |
| sp|Q8N0Z6|TTC5_HUMAN | 49220.97321 | 0.94 | 0.078 | 0.0004632 |
| sp|P62841|RS15_HUMAN | 17011.16275 | 1.69 | 0.459 | 5.53E-09 |
| sp|Q5VZE5|NAA35_HUMAN | 84535.48601 | 0.75 | 0.148 | 1.34E-07 |
| sp|Q8N9N7|LRC57_HUMAN | 27061.42999 | 0.79 | 0.187 | 1.08E-05 |
| sp|P09525|ANXA4_HUMAN | 36070.19617 | 0.87 | 0.211 | 0.001125 |
| sp|Q9NRN7|ADPPT_HUMAN | 35963.14439 | 0.89 | 0.113 | 0.0001759 |
| sp|P56589|PEX3_HUMAN | 42494.08744 | 0.82 | 0.123 | 3.73E-07 |
| sp|Q5VWQ8|DAB2P_HUMAN | 132494.5026 | 0.94 | 0.067 | 7.08E-05 |
| sp|Q8N8S7|ENAH_HUMAN | 66565.92697 | 1.5 | 0.398 | 1.30E-07 |
| sp|P52292|IMA1_HUMAN | 58150.06541 | 0.64 | 0.34 | 2.21E-05 |
| sp|P16435|NCPR_HUMAN | 77079.4119 | 0.77 | 0.108 | 1.20E-09 |
| sp|Q13616|CUL1_HUMAN | 90288.22557 | 0.74 | 0.061 | 1.13E-15 |
| sp|Q13435|SF3B2_HUMAN | 100261.0095 | 1.31 | 0.164 | 2.03E-10 |
| sp|Q9NZP8|C1RL_HUMAN | 54187.6129 | 1.12 | 0.17 | 0.004382 |
| sp|Q8IY22|CMIP_HUMAN | 87682.64256 | 0.43 | 0.068 | 2.20E-16 |
| sp|A0A0C4DH34|HV428_HUMAN | 13211.68531 | 0.47 | 0.187 | 2.65E-10 |
| sp|Q7L8L6|FAKD5_HUMAN | 87469.97844 | 0.7 | 0.13 | 7.58E-10 |
| sp|P00995|ISK1_HUMAN | 8825.294101 | 0.87 | 0.528 | 0.01784 |
| sp|P02760|AMBP_HUMAN | 39868.31421 | 1.33 | 0.354 | 0.0001006 |
| sp|O94874|UFL1_HUMAN | 89977.91217 | 0.81 | 0.113 | 3.37E-08 |
| sp|Q9NQZ5|STAR7_HUMAN | 43238.75052 | 1.33 | 0.245 | 8.79E-08 |
| sp|O14545|TRAD1_HUMAN | 66207.67118 | 1.21 | 0.46 | 0.07722 |
| sp|O95758|PTBP3_HUMAN | 59919.14743 | 0.92 | 0.166 | 0.006549 |
| sp|Q9Y2K7|KDM2A_HUMAN | 135141.4493 | 0.62 | 0.16 | 2.47E-10 |
| sp|P23470|PTPRG_HUMAN | 163023.8046 | 0.96 | 0.208 | 0.1853 |
| sp|Q9H019|MFR1L_HUMAN | 32375.42758 | 1.07 | 0.241 | 0.3375 |
| sp|Q03111|ENL_HUMAN | 62399.09605 | 1.05 | 0.275 | 0.7516 |
| sp|Q9P265|DIP2B_HUMAN | 173587.8532 | 0.7 | 0.186 | 3.69E-07 |
| sp|Q9Y5J7|TIM9_HUMAN | 10581.20378 | 1.31 | 0.236 | 1.27E-07 |
| sp|O60664|PLIN3_HUMAN | 47198.99973 | 1.46 | 0.205 | 9.09E-13 |
| sp|Q8WUX9|CHMP7_HUMAN | 51146.43645 | 0.95 | 0.184 | 0.08736 |
| sp|P02792|FRIL_HUMAN | 20046.11312 | 2.17 | 0.99 | 2.20E-08 |
| sp|P04899|GNAI2_HUMAN | 40977.25842 | 0.75 | 0.102 | 1.30E-10 |
| sp|Q03518|TAP1_HUMAN | 87714.92437 | 0.77 | 0.249 | 3.99E-05 |
| sp|Q8WW22|DNJA4_HUMAN | 45435.23785 | 0.82 | 0.074 | 7.13E-11 |
| sp|Q12766|HMGX3_HUMAN | 170661.8576 | 1.16 | 0.712 | 0.9337 |
| sp|Q6VY07|PACS1_HUMAN | 105214.5238 | 1.18 | 0.166 | 1.11E-05 |
| sp|P61619|S61A1_HUMAN | 52668.67462 | 0.51 | 0.111 | 3.05E-14 |
| sp|Q96MF7|NSE2_HUMAN | 28239.10901 | 1.31 | 0.116 | 1.09E-13 |
| sp|O43896|KIF1C_HUMAN | 123707.2388 | 0.79 | 0.154 | 2.05E-06 |
| sp|P52788|SPSY_HUMAN | 41679.94539 | 0.63 | 0.129 | 2.13E-11 |
| sp|Q9H3H3|CK068_HUMAN | 31563.79807 | 1.27 | 0.187 | 5.07E-08 |
| sp|Q9P2Y5|UVRAG_HUMAN | 78882.50655 | 0.98 | 0.108 | 0.1764 |
| sp|Q8NGK1|O51G1_HUMAN | 36988.63567 | 1.39 | 0.958 | 0.1284 |
| sp|Q5RKV6|EXOS6_HUMAN | 28484.7933 | 1.26 | 0.259 | 2.86E-05 |
| sp|Q9BR61|ACBD6_HUMAN | 31569.52966 | 1.33 | 0.149 | 4.17E-12 |
| sp|Q9UJA5|TRM6_HUMAN | 56031.42367 | 0.93 | 0.229 | 0.06336 |
| sp|P28300|LYOX_HUMAN | 47580.76705 | 1.66 | 0.264 | 7.07E-14 |
| sp|Q9BRP4|PAAF1_HUMAN | 43114.39837 | 0.88 | 0.147 | 0.0003083 |
| sp|P30043|BLVRB_HUMAN | 22201.45882 | 1.2 | 0.076 | 4.74E-13 |
| sp|Q96DH6|MSI2H_HUMAN | 35327.17305 | 1.32 | 0.54 | 0.004187 |
| sp|O00478|BT3A3_HUMAN | 65456.05779 | 1.18 | 0.429 | 0.1241 |
| sp|Q9ULC4|MCTS1_HUMAN | 20751.89626 | 0.65 | 0.071 | 3.89E-16 |
| sp|P15336|ATF2_HUMAN | 54770.85668 | 1.26 | 0.15 | 1.38E-09 |
| sp|Q8N129|CNPY4_HUMAN | 28616.04687 | 1.43 | 0.237 | 4.14E-10 |
| sp|A6NDG6|PGP_HUMAN | 34422.66895 | 1.19 | 0.201 | 8.89E-05 |
| sp|Q9UHB6|LIMA1_HUMAN | 85611.51962 | 1.54 | 0.325 | 4.13E-09 |
| sp|P48444|COPD_HUMAN | 57612.28637 | 1.07 | 0.198 | 0.201 |
| sp|Q6PK18|OGFD3_HUMAN | 35834.02475 | 0.97 | 0.255 | 0.285 |
| sp|Q8N3P4|VPS8_HUMAN | 163684.7931 | 0.7 | 0.112 | 3.20E-11 |
| sp|P11308|ERG_HUMAN | 54840.77681 | 0.85 | 0.19 | 0.0005475 |
| sp|P53602|MVD1_HUMAN | 43872.33352 | 0.67 | 0.113 | 6.50E-12 |
| sp|O15204|ADEC1_HUMAN | 53692.89353 | 2.4 | 3.577 | 0.3076 |
| sp|Q92625|ANS1A_HUMAN | 123983.0739 | 0.84 | 0.152 | 0.0001171 |
| sp|P55769|NH2L1_HUMAN | 14374.63001 | 1.23 | 0.12 | 1.92E-10 |
| sp|Q6GPH4|XAF1_HUMAN | 35839.14572 | 1.17 | 0.286 | 0.01889 |
| sp|Q9HAJ7|SP30L_HUMAN | 21073.68838 | 1.34 | 0.183 | 9.88E-11 |
| sp|Q96C90|PP14B_HUMAN | 15997.07795 | 1.35 | 0.431 | 0.0008591 |
| sp|P03886|NU1M_HUMAN | 35618.99441 | 0.44 | 0.151 | 1.32E-11 |
| sp|P01130|LDLR_HUMAN | 98887.9767 | 0.57 | 0.117 | 2.01E-13 |
| sp|Q13568|IRF5_HUMAN | 56560.75273 | 1.28 | 0.178 | 1.01E-08 |
| sp|Q9NQC7|CYLD_HUMAN | 108996.386 | 0.71 | 0.25 | 1.61E-05 |
| sp|Q86TC9|MYPN_HUMAN | 146688.1493 | 1.25 | 0.309 | 0.0006322 |
| sp|Q04760|LGUL_HUMAN | 20974.3231 | 1.03 | 0.168 | 0.5495 |
| sp|P01008|ANT3_HUMAN | 53007.02548 | 1.29 | 0.289 | 1.91E-05 |
| sp|Q9H6T3|RPAP3_HUMAN | 75938.99964 | 1.23 | 0.152 | 3.09E-08 |
| sp|Q9Y315|DEOC_HUMAN | 35475.5704 | 0.96 | 0.104 | 0.08074 |
| sp|O75044|SRGP2_HUMAN | 121974.0521 | 1.08 | 0.169 | 0.04418 |
| sp|Q8IWJ2|GCC2_HUMAN | 196854.109 | 1.26 | 0.256 | 9.93E-06 |
| sp|P28370|SMCA1_HUMAN | 123193.3318 | 0.78 | 0.07 | 3.62E-13 |
| sp|O60336|MABP1_HUMAN | 165522.9022 | 1.02 | 0.246 | 0.8289 |
| sp|Q5TGL8|PXDC1_HUMAN | 26696.38083 | 0.73 | 0.113 | 4.04E-10 |
| sp|Q9H061|T126A_HUMAN | 21723.55828 | 0.76 | 0.16 | 3.36E-07 |
| sp|P35612|ADDB_HUMAN | 81241.59128 | 1.35 | 0.263 | 2.23E-07 |
| sp|P37059|DHB2_HUMAN | 43423.20214 | 0.67 | 0.357 | 0.0003346 |
| sp|P53004|BIEA_HUMAN | 33674.42696 | 0.81 | 0.13 | 4.21E-07 |
| sp|P35241|RADI_HUMAN | 68617.42285 | 1.34 | 0.101 | 4.13E-16 |
| sp|Q4J6C6|PPCEL_HUMAN | 84824.72473 | 1.04 | 0.101 | 0.09685 |
| sp|O95721|SNP29_HUMAN | 28934.58453 | 1.19 | 0.207 | 7.07E-05 |
| sp|Q8IUC6|TCAM1_HUMAN | 77325.21195 | 1.08 | 0.132 | 0.006885 |
| sp|Q86V88|MGDP1_HUMAN | 20135.32609 | 1.19 | 0.124 | 2.79E-08 |
| sp|Q86WB0|NIPA_HUMAN | 56348.48857 | 0.79 | 0.122 | 3.27E-08 |
| sp|P63218|GBG5_HUMAN | 7409.837378 | 0.62 | 0.168 | 2.16E-09 |
| sp|P41271|NBL1_HUMAN | 19947.38651 | 1.01 | 0.4 | 0.485 |
| sp|P25940|CO5A3_HUMAN | 172682.0294 | 1.28 | 0.555 | 0.06199 |
| sp|Q8NI27|THOC2_HUMAN | 184523.0436 | 0.75 | 0.086 | 4.69E-12 |
| sp|Q4G0J3|LARP7_HUMAN | 67124.51028 | 1.04 | 0.098 | 0.09919 |
| sp|P56537|IF6_HUMAN | 27077.37727 | 1.15 | 0.171 | 8.65E-05 |
| sp|P01619|KV320_HUMAN | 12645.29925 | 1.22 | 0.423 | 0.06505 |
| sp|Q04912|RON_HUMAN | 154692.0889 | 0.52 | 0.133 | 3.49E-12 |
| sp|O75521|ECI2_HUMAN | 43938.51565 | 1.17 | 0.264 | 0.009833 |
| sp|P52655|TF2AA_HUMAN | 41527.48057 | 1.21 | 0.225 | 4.93E-05 |
| sp|Q5VYY1|ANR22_HUMAN | 22215.50286 | 0.62 | 0.275 | 3.85E-06 |
| sp|Q96HD1|CREL1_HUMAN | 47614.6524 | 0.79 | 0.136 | 1.09E-07 |
| sp|P16109|LYAM3_HUMAN | 94575.66473 | 1.29 | 0.558 | 0.02109 |
| sp|Q8TAX9|GSDMB_HUMAN | 46967.10406 | 1.18 | 0.684 | 0.8705 |
| sp|Q07021|C1QBP_HUMAN | 31723.74809 | 1.69 | 0.467 | 5.16E-09 |
| sp|P18887|XRCC1_HUMAN | 69758.0512 | 1.18 | 0.216 | 0.0009537 |
| sp|P30041|PRDX6_HUMAN | 25115.22267 | 0.99 | 0.2 | 0.5497 |
| sp|Q8N5M1|ATPF2_HUMAN | 32961.9823 | 0.57 | 0.105 | 2.96E-14 |
| sp|Q9NX20|RM16_HUMAN | 28470.02135 | 0.95 | 0.209 | 0.09816 |
| sp|Q6P5R6|RL22L_HUMAN | 14579.56396 | 1.36 | 0.444 | 0.0004912 |
| sp|Q9NX62|IMPA3_HUMAN | 38810.00717 | 0.91 | 0.115 | 0.001025 |
| sp|Q9NRL2|BAZ1A_HUMAN | 180227.5991 | 1.08 | 0.467 | 0.9934 |
| sp|Q9HCN4|GPN1_HUMAN | 41809.72498 | 1.13 | 0.244 | 0.03412 |
| sp|Q9BT17|MTG1_HUMAN | 37593.93112 | 1.03 | 0.153 | 0.4085 |
| sp|P09012|SNRPA_HUMAN | 31241.18816 | 0.96 | 0.296 | 0.1653 |
| sp|O95487|SC24B_HUMAN | 138510.5822 | 0.7 | 0.083 | 5.18E-14 |
| sp|Q13330|MTA1_HUMAN | 81401.51844 | 1.13 | 0.105 | 1.48E-06 |
| sp|Q9UNS2|CSN3_HUMAN | 48394.21806 | 0.68 | 0.14 | 1.93E-09 |
| sp|P04075|ALDOA_HUMAN | 39833.4671 | 1.14 | 0.059 | 7.77E-12 |
| sp|P14616|INSRR_HUMAN | 146233.0065 | 1.35 | 0.566 | 0.004681 |
| sp|Q9UPN7|PP6R1_HUMAN | 97272.94493 | 0.7 | 0.128 | 3.55E-10 |
| sp|P49447|CY561_HUMAN | 27694.62579 | 1.37 | 0.683 | 0.06149 |
| sp|Q63HN8|RN213_HUMAN | 598539.0685 | 0.79 | 0.195 | 9.06E-06 |
| sp|O75448|MED24_HUMAN | 111698.3377 | 0.52 | 0.122 | 1.73E-13 |
| sp|P02654|APOC1_HUMAN | 9308.076456 | 0.83 | 0.439 | 0.005569 |
| sp|P35914|HMGCL_HUMAN | 34775.99558 | 0.72 | 0.172 | 1.61E-07 |
| sp|Q05823|RN5A_HUMAN | 84260.82939 | 0.92 | 0.113 | 0.001077 |
| sp|Q7Z4H7|HAUS6_HUMAN | 109618.1823 | 0.75 | 0.122 | 4.96E-09 |
| sp|Q9BZE4|NOG1_HUMAN | 74298.96219 | 0.88 | 0.137 | 0.00021 |
| sp|P20674|COX5A_HUMAN | 16904.74291 | 1.26 | 0.325 | 0.001116 |
| sp|Q15223|NECT1_HUMAN | 57446.59572 | 1.15 | 0.247 | 0.009523 |
| sp|Q7Z418|KCNKI_HUMAN | 44479.81882 | 1.14 | 0.387 | 0.1357 |
| sp|Q02153|GCYB1_HUMAN | 71249.8711 | 0.71 | 0.143 | 6.33E-09 |
| sp|P57740|NU107_HUMAN | 107030.1606 | 0.71 | 0.115 | 5.50E-11 |
| sp|Q9Y6A5|TACC3_HUMAN | 91141.0647 | 0.96 | 0.19 | 0.1455 |
| sp|Q9NTK5|OLA1_HUMAN | 44925.37394 | 0.91 | 0.138 | 0.002235 |
| sp|P07738|PMGE_HUMAN | 30139.56421 | 1.2 | 0.259 | 0.0006724 |
| sp|Q9Y6E0|STK24_HUMAN | 49544.41267 | 1.38 | 0.173 | 7.16E-12 |
| sp|Q9NY12|GAR1_HUMAN | 22487.20851 | 1.13 | 0.271 | 0.07154 |
| sp|Q13938|CAYP1_HUMAN | 30487.9794 | 0.74 | 0.433 | 0.0009576 |
| sp|P53370|NUDT6_HUMAN | 35980.17396 | 1.37 | 0.447 | 0.0003307 |
| sp|P20339|RAB5A_HUMAN | 23853.89303 | 0.97 | 0.312 | 0.1964 |
| sp|Q96AG3|S2546_HUMAN | 46411.91855 | 0.7 | 0.186 | 5.85E-07 |
| sp|Q9UBS0|KS6B2_HUMAN | 53802.68436 | 0.94 | 0.154 | 0.02339 |
| sp|Q13573|SNW1_HUMAN | 61495.49331 | 1.18 | 0.126 | 1.64E-07 |
| sp|Q96Q80|DERL3_HUMAN | 26757.04017 | 0.88 | 0.567 | 0.02425 |
| sp|Q96A33|CCD47_HUMAN | 56105.40803 | 0.83 | 0.143 | 5.14E-06 |
| sp|Q9HBA9|FOH1B_HUMAN | 50109.04792 | 0.8 | 0.325 | 0.009396 |
| sp|P10721|KIT_HUMAN | 111144.5964 | 0.47 | 0.258 | 5.71E-09 |
| sp|Q9Y3Q3|TMED3_HUMAN | 25028.39752 | 0.88 | 0.063 | 2.79E-09 |
| sp|O15514|RPB4_HUMAN | 16397.15737 | 1.54 | 0.223 | 1.62E-13 |
| sp|P29728|OAS2_HUMAN | 83329.86239 | 0.84 | 0.231 | 0.001509 |
| sp|Q96EK9|KTI12_HUMAN | 38801.92603 | 1.37 | 0.112 | 4.80E-16 |
| sp|Q9UBU9|NXF1_HUMAN | 70633.86725 | 0.9 | 0.13 | 0.0005641 |
| sp|Q16873|LTC4S_HUMAN | 16652.17602 | 0.56 | 0.119 | 1.14E-12 |
| sp|P08708|RS17_HUMAN | 15579.40076 | 1.37 | 0.192 | 5.72E-11 |
| sp|Q9UM47|NOTC3_HUMAN | 256622.0841 | 1.68 | 0.522 | 7.15E-08 |
| sp|Q04726|TLE3_HUMAN | 84144.40268 | 1.02 | 0.108 | 0.4957 |
| sp|O43181|NDUS4_HUMAN | 20077.42175 | 1.89 | 0.793 | 4.98E-06 |
| sp|Q9H875|PKRI1_HUMAN | 20965.92735 | 1.93 | 0.868 | 4.95E-06 |
| sp|Q6ZMG9|CERS6_HUMAN | 45355.80434 | 0.54 | 0.222 | 1.30E-07 |
| sp|Q9Y653|AGRG1_HUMAN | 78525.46541 | 0.85 | 0.16 | 0.0001036 |
| sp|P48729|KC1A_HUMAN | 39099.96366 | 0.53 | 0.117 | 2.86E-14 |
| sp|Q9Y6Z7|COL10_HUMAN | 31066.46228 | 1.09 | 0.571 | 0.6164 |
| sp|Q96SZ5|AEDO_HUMAN | 30056.03021 | 1.05 | 0.165 | 0.3108 |
| sp|Q9C000|NLRP1_HUMAN | 167794.7469 | 0.87 | 0.149 | 0.0001101 |
| sp|P00747|PLMN_HUMAN | 93229.17693 | 0.91 | 0.455 | 0.0469 |
| sp|P49903|SPS1_HUMAN | 43377.84219 | 1.13 | 0.147 | 0.0002403 |
| sp|Q9GZS3|WDR61_HUMAN | 33712.8229 | 1.07 | 0.116 | 0.01001 |
| sp|P52565|GDIR1_HUMAN | 23231.71628 | 1.24 | 0.183 | 1.73E-07 |
| sp|O75386|TULP3_HUMAN | 49992.03414 | 0.98 | 0.313 | 0.3229 |
| sp|Q96AE7|TTC17_HUMAN | 130656.1542 | 1.14 | 0.32 | 0.1187 |
| sp|O43617|TPPC3_HUMAN | 20414.20095 | 0.64 | 0.148 | 7.53E-10 |
| sp|P13688|CEAM1_HUMAN | 57962.87221 | 0.66 | 0.322 | 3.30E-05 |
| sp|Q5W0V3|F16B1_HUMAN | 87568.46164 | 0.66 | 0.128 | 1.00E-10 |
| sp|P49458|SRP09_HUMAN | 10201.14335 | 1.08 | 0.158 | 0.02035 |
| sp|Q01469|FABP5_HUMAN | 15478.66953 | 1.36 | 0.315 | 1.53E-06 |
| sp|Q15772|SPEG_HUMAN | 356846.614 | 1.78 | 0.485 | 1.36E-10 |
| sp|Q9UBL3|ASH2L_HUMAN | 69288.70511 | 1.2 | 0.141 | 2.61E-07 |
| sp|P05062|ALDOB_HUMAN | 39943.24287 | 0.75 | 0.306 | 0.00147 |
| sp|P09936|UCHL1_HUMAN | 25132.57604 | 2.48 | 0.765 | 7.51E-13 |
| sp|Q96GP6|SREC2_HUMAN | 96866.56512 | 1.58 | 0.545 | 1.57E-06 |
| sp|Q9Y2J8|PADI2_HUMAN | 76238.60919 | 0.46 | 0.099 | 7.26E-15 |
| sp|Q86VP1|TAXB1_HUMAN | 91885.2846 | 0.91 | 0.164 | 0.008354 |
| sp|Q9NP92|RT30_HUMAN | 50941.98778 | 0.7 | 0.073 | 5.74E-15 |
| sp|P25942|TNR5_HUMAN | 31892.28147 | 1.23 | 0.616 | 0.2284 |
| sp|P49902|5NTC_HUMAN | 65366.47559 | 0.83 | 0.092 | 5.85E-09 |
| sp|P51570|GALK1_HUMAN | 42683.73207 | 1.03 | 0.124 | 0.3724 |
| sp|P31939|PUR9_HUMAN | 65070.52468 | 0.94 | 0.137 | 0.02962 |
| sp|Q9H6S3|ES8L2_HUMAN | 81179.34752 | 0.93 | 0.184 | 0.02849 |
| sp|P30040|ERP29_HUMAN | 29014.16648 | 1.36 | 0.331 | 2.96E-06 |
| sp|Q8TB03|CX038_HUMAN | 37426.59636 | 0.79 | 0.163 | 2.34E-06 |
| sp|Q14409|GLPK3_HUMAN | 61396.05407 | 0.56 | 0.143 | 1.51E-11 |
| sp|P23946|CMA1_HUMAN | 27745.34889 | 0.93 | 0.34 | 0.06826 |
| sp|Q6UXN9|WDR82_HUMAN | 35437.6047 | 0.75 | 0.246 | 1.23E-05 |
| sp|Q9BW92|SYTM_HUMAN | 81822.74661 | 0.73 | 0.169 | 1.47E-07 |
| sp|P43155|CACP_HUMAN | 71250.48391 | 0.76 | 0.098 | 8.95E-11 |
| sp|Q6UX01|LMBRL_HUMAN | 55839.61762 | 0.66 | 0.173 | 3.43E-08 |
| sp|P33908|MA1A1_HUMAN | 73132.35405 | 0.73 | 0.203 | 1.04E-06 |
| sp|Q13838|DX39B_HUMAN | 49398.10259 | 0.54 | 0.064 | 2.20E-16 |
| sp|Q14587|ZN268_HUMAN | 111706.3502 | 1.32 | 0.581 | 0.04517 |
| sp|Q8IWE5|PKHM2_HUMAN | 114401.7226 | 1.1 | 0.14 | 0.001408 |
| sp|O15523|DDX3Y_HUMAN | 73546.28242 | 2.36 | 1.448 | 1.06E-06 |
| sp|Q3SYG4|PTHB1_HUMAN | 100396.7297 | 1.49 | 0.345 | 1.19E-07 |
| sp|P19623|SPEE_HUMAN | 34354.91973 | 1.42 | 0.129 | 3.40E-16 |
| sp|Q8ND24|RN214_HUMAN | 78341.91723 | 1.2 | 0.177 | 7.59E-06 |
| sp|O43242|PSMD3_HUMAN | 61035.52035 | 0.58 | 0.117 | 3.88E-13 |
| sp|O75052|CAPON_HUMAN | 56438.90879 | 0.62 | 0.211 | 2.49E-07 |
| sp|P04083|ANXA1_HUMAN | 38900.05444 | 1.5 | 0.369 | 8.80E-08 |
| sp|Q9BYX7|ACTBM_HUMAN | 42312.96062 | 0.89 | 0.173 | 0.001798 |
| sp|Q96M27|PRRC1_HUMAN | 46710.82645 | 1.31 | 0.329 | 4.72E-05 |
| sp|Q9UH03|SEPT3_HUMAN | 40945.14553 | 1.13 | 0.358 | 0.3065 |
| sp|Q8N1B4|VPS52_HUMAN | 82493.73746 | 0.6 | 0.082 | 7.47E-16 |
| sp|P33176|KINH_HUMAN | 110340.2576 | 0.99 | 0.114 | 0.6024 |
| sp|Q9UNW9|NOVA2_HUMAN | 49131.63373 | 1.32 | 0.249 | 1.60E-07 |
| sp|Q14376|GALE_HUMAN | 38638.39869 | 0.53 | 0.191 | 2.44E-08 |
| sp|Q9H7C9|AAMDC_HUMAN | 13419.80098 | 1.44 | 0.529 | 0.0009148 |
| sp|Q15436|SC23A_HUMAN | 86999.58802 | 0.83 | 0.088 | 6.70E-09 |
| sp|Q9BRT2|UQCC2_HUMAN | 14961.48099 | 1.29 | 0.25 | 5.40E-06 |
| sp|P61088|UBE2N_HUMAN | 17165.98537 | 0.91 | 0.157 | 0.003393 |
| sp|P62266|RS23_HUMAN | 15950.77102 | 1.33 | 0.177 | 1.37E-10 |
| sp|Q9UNN5|FAF1_HUMAN | 74345.7416 | 0.71 | 0.08 | 4.36E-14 |
| sp|Q9Y5M8|SRPRB_HUMAN | 29893.8367 | 0.38 | 0.113 | 3.79E-14 |
| sp|P35611|ADDA_HUMAN | 81285.87892 | 1 | 0.212 | 0.6313 |
| sp|Q9ULG6|CCPG1_HUMAN | 88009.34994 | 0.81 | 0.1 | 1.59E-09 |
| sp|Q9NSY2|STAR5_HUMAN | 24159.9199 | 1.2 | 0.222 | 0.0001952 |
| sp|Q9HC07|TM165_HUMAN | 35036.53724 | 0.84 | 0.152 | 3.38E-05 |
| sp|Q8N6M0|OTU6B_HUMAN | 34001.56634 | 1.02 | 0.283 | 0.6641 |
| sp|Q6DT37|MRCKG_HUMAN | 173759.7458 | 0.98 | 0.289 | 0.3383 |
| sp|Q01433|AMPD2_HUMAN | 101404.219 | 0.77 | 0.109 | 4.07E-09 |
| sp|Q5H8C1|FREM1_HUMAN | 245807.1698 | 0.56 | 0.094 | 1.77E-15 |
| sp|Q96HD9|ACY3_HUMAN | 35485.6216 | 0.98 | 0.136 | 0.2462 |
| sp|O43866|CD5L_HUMAN | 39584.51265 | 1.12 | 0.356 | 0.3157 |
| sp|O75153|CLU_HUMAN | 147984.6864 | 0.72 | 0.174 | 4.71E-07 |
| sp|P37837|TALDO_HUMAN | 37669.51548 | 0.56 | 0.092 | 1.38E-15 |
| sp|Q9P2W9|STX18_HUMAN | 38802.69108 | 0.86 | 0.126 | 7.33E-06 |
| sp|Q96N67|DOCK7_HUMAN | 244271.0432 | 0.85 | 0.084 | 4.37E-09 |
| sp|P10619|PPGB_HUMAN | 54926.30357 | 0.71 | 0.186 | 3.30E-07 |
| sp|Q8NBF6|AVL9_HUMAN | 72226.5565 | 1.09 | 0.183 | 0.03097 |
| sp|O96013|PAK4_HUMAN | 64299.28553 | 1 | 0.112 | 0.9338 |
| sp|Q9UM00|TMCO1_HUMAN | 21371.23444 | 0.57 | 0.145 | 8.70E-12 |
| sp|Q86YW9|MD12L_HUMAN | 242686.1145 | 1.22 | 0.179 | 1.41E-06 |
| sp|Q9Y3B6|EMC9_HUMAN | 23313.75219 | 0.9 | 0.295 | 0.02294 |
| sp|O75223|GGCT_HUMAN | 21204.40848 | 1.06 | 0.245 | 0.5353 |
| sp|Q9NVK5|FGOP2_HUMAN | 29504.04176 | 1.21 | 0.112 | 3.85E-10 |
| sp|P50452|SPB8_HUMAN | 43291.39788 | 1.43 | 0.421 | 2.59E-05 |
| sp|P48723|HSP13_HUMAN | 51934.10625 | 1.07 | 0.148 | 0.04182 |
| sp|P35250|RFC2_HUMAN | 39570.25689 | 0.8 | 0.174 | 8.96E-06 |
| sp|Q5TC12|ATPF1_HUMAN | 36737.75995 | 0.93 | 0.322 | 0.06367 |
| sp|Q99717|SMAD5_HUMAN | 52890.97624 | 1.16 | 0.092 | 2.11E-09 |
| sp|Q6ZVK8|NUD18_HUMAN | 35973.55321 | 0.85 | 0.093 | 1.31E-07 |
| sp|O15231|ZN185_HUMAN | 74374.53723 | 1.06 | 0.191 | 0.3143 |
| sp|Q5JTZ9|SYAM_HUMAN | 108281.2772 | 0.85 | 0.196 | 0.0007604 |
| sp|P33993|MCM7_HUMAN | 81865.79529 | 0.91 | 0.268 | 0.02256 |
| sp|Q6ULP2|AFTIN_HUMAN | 102830.118 | 1 | 0.189 | 0.7111 |
| sp|Q9Y2E8|SL9A8_HUMAN | 65589.34763 | 0.56 | 0.094 | 3.14E-15 |
| sp|Q7Z2Z2|EFL1_HUMAN | 126643.4611 | 0.86 | 0.098 | 2.38E-07 |
| sp|Q13683|ITA7_HUMAN | 130102.8794 | 1.07 | 0.191 | 0.1642 |
| sp|P27338|AOFB_HUMAN | 59220.33812 | 0.73 | 0.197 | 2.52E-06 |
| sp|P02549|SPTA1_HUMAN | 281021.3365 | 1.53 | 0.28 | 2.48E-11 |
| sp|Q05469|LIPS_HUMAN | 117305.3222 | 1.18 | 0.176 | 2.68E-05 |
| sp|P35555|FBN1_HUMAN | 332646.2411 | 1.05 | 0.483 | 0.6349 |
| sp|Q5RI15|COX20_HUMAN | 13492.87167 | 0.81 | 0.178 | 2.58E-05 |
| sp|Q8WYP5|ELYS_HUMAN | 254205.2988 | 1.21 | 0.067 | 1.01E-14 |
| sp|P30085|KCY_HUMAN | 22418.35549 | 1.02 | 0.25 | 0.7977 |
| sp|O14763|TR10B_HUMAN | 48856.07622 | 1.11 | 0.394 | 0.5444 |
| sp|Q07092|COGA1_HUMAN | 159459.2192 | 3.11 | 1.92 | 2.08E-09 |
| sp|Q6DN90|IQEC1_HUMAN | 109084.6035 | 0.72 | 0.044 | 2.20E-16 |
| sp|O60613|SEP15_HUMAN | 18519.25071 | 1.1 | 0.128 | 0.0008475 |
| sp|Q9H4M9|EHD1_HUMAN | 60627.67643 | 0.78 | 0.125 | 2.81E-08 |
| sp|P47985|UCRI_HUMAN | 29916.4627 | 1.06 | 0.168 | 0.1455 |
| sp|Q6P4E1|CASC4_HUMAN | 48930.72635 | 0.9 | 0.164 | 0.003627 |
| sp|P62699|YPEL5_HUMAN | 14042.91995 | 1.58 | 0.186 | 9.26E-16 |
| sp|Q9ULC3|RAB23_HUMAN | 26852.62351 | 1.08 | 0.243 | 0.2319 |
| sp|P14314|GLU2B_HUMAN | 60339.17546 | 1.49 | 0.194 | 6.78E-14 |
| sp|P80294|MT1H_HUMAN | 7156.687907 | 1.09 | 0.578 | 0.6638 |
| sp|O95376|ARI2_HUMAN | 59359.72173 | 0.86 | 0.117 | 5.74E-06 |
| sp|P30837|AL1B1_HUMAN | 57608.38724 | 0.91 | 0.139 | 0.002811 |
| sp|P56524|HDAC4_HUMAN | 119745.9936 | 1 | 0.173 | 0.792 |
| sp|Q9BU02|THTPA_HUMAN | 25703.07196 | 1.38 | 0.137 | 5.39E-14 |
| sp|Q8NCF5|NF2IP_HUMAN | 45941.80191 | 1.7 | 0.649 | 3.20E-06 |
| sp|Q6FI81|CPIN1_HUMAN | 34113.28565 | 1.32 | 0.339 | 0.0001129 |
| sp|Q8N3X1|FNBP4_HUMAN | 110636.4103 | 1.14 | 0.231 | 0.01006 |
| sp|Q8IZQ5|SELH_HUMAN | 13485.14687 | 1.19 | 0.214 | 0.00016 |
| sp|Q53QV2|LBH_HUMAN | 12418.7744 | 1.17 | 0.215 | 0.00058 |
| sp|O15156|ZBT7B_HUMAN | 58884.5766 | 0.87 | 0.194 | 0.0008999 |
| sp|Q06323|PSME1_HUMAN | 28858.06859 | 1.01 | 0.286 | 0.7015 |
| sp|O95497|VNN1_HUMAN | 57698.33295 | 1.02 | 0.482 | 0.3739 |
| sp|P26572|MGAT1_HUMAN | 51113.50426 | 0.63 | 0.088 | 6.80E-15 |
| sp|O95429|BAG4_HUMAN | 49773.49075 | 0.87 | 0.1 | 1.25E-06 |
| sp|Q8NHQ1|CEP70_HUMAN | 70260.13461 | 1.04 | 0.115 | 0.148 |
| sp|Q5NDL2|EOGT_HUMAN | 62808.07834 | 0.66 | 0.107 | 7.39E-13 |
| sp|Q15102|PA1B3_HUMAN | 25814.26476 | 0.91 | 0.132 | 0.0009936 |
| sp|Q9H773|DCTP1_HUMAN | 18765.39267 | 1.01 | 0.209 | 0.7495 |
| sp|A0M8Q6|IGLC7_HUMAN | 11399.66708 | 1.06 | 0.229 | 0.4859 |
| sp|A0A0C4DH72|KV106_HUMAN | 12842.40599 | 1.35 | 0.478 | 0.0003654 |
| sp|Q9H3S7|PTN23_HUMAN | 179812.3146 | 0.92 | 0.089 | 0.0003879 |
| sp|Q6UX15|LAYN_HUMAN | 43747.32087 | 1.44 | 0.321 | 2.22E-07 |
| sp|O75164|KDM4A_HUMAN | 122562.8318 | 0.47 | 0.069 | 2.20E-16 |
| sp|Q92851|CASPA_HUMAN | 59579.47296 | 1.31 | 0.744 | 0.1508 |
| sp|Q9UIJ7|KAD3_HUMAN | 25531.53744 | 0.98 | 0.139 | 0.2964 |
| sp|Q9UBB6|NCDN_HUMAN | 80221.16201 | 0.58 | 0.063 | 2.20E-16 |
| sp|Q8IUE6|H2A2B_HUMAN | 13968.83318 | 1.08 | 0.115 | 0.002687 |
| sp|Q9UMY4|SNX12_HUMAN | 19871.14637 | 1.28 | 0.109 | 1.85E-13 |
| sp|P78371|TCPB_HUMAN | 57776.24807 | 1.2 | 0.133 | 2.48E-08 |
| sp|Q14203|DCTN1_HUMAN | 142330.342 | 1.02 | 0.092 | 0.3537 |
| sp|P14778|IL1R1_HUMAN | 66254.95356 | 0.82 | 0.319 | 0.005228 |
| sp|Q9UJF2|NGAP_HUMAN | 129599.6284 | 1.31 | 0.171 | 6.61E-10 |
| sp|P49366|DHYS_HUMAN | 41154.66196 | 1.05 | 0.193 | 0.3915 |
| sp|Q15648|MED1_HUMAN | 169324.4821 | 0.55 | 0.056 | 2.20E-16 |
| sp|P22570|ADRO_HUMAN | 54241.17214 | 1.26 | 0.096 | 1.66E-13 |
| sp|P0DOX4|IGE_HUMAN | 61122.01032 | 0.8 | 0.268 | 0.002207 |
| sp|Q9H9Y6|RPA2_HUMAN | 129781.2836 | 0.53 | 0.116 | 1.28E-13 |
| sp|Q9Y5B9|SP16H_HUMAN | 120390.6236 | 0.74 | 0.187 | 8.67E-07 |
| sp|Q93088|BHMT1_HUMAN | 45408.00986 | 0.59 | 0.107 | 3.89E-13 |
| sp|Q8IUF8|RIOX2_HUMAN | 53091.14729 | 1.17 | 0.233 | 0.001274 |
| sp|Q13085|ACACA_HUMAN | 267077.4836 | 0.66 | 0.119 | 1.86E-10 |
| sp|Q96BQ1|FAM3D_HUMAN | 25213.8662 | 0.47 | 0.205 | 8.43E-10 |
| sp|Q6ZNE5|BAKOR_HUMAN | 55997.82814 | 0.97 | 0.102 | 0.07211 |
| sp|Q8WXG6|MADD_HUMAN | 184481.6413 | 0.7 | 0.073 | 4.08E-15 |
| sp|P22676|CALB2_HUMAN | 31615.60932 | 3.55 | 2.259 | 1.94E-09 |
| sp|Q99832|TCPH_HUMAN | 59824.10679 | 0.79 | 0.09 | 1.73E-10 |
| sp|Q9NZQ7|PD1L1_HUMAN | 33578.26117 | 1.07 | 0.344 | 0.6642 |
| sp|Q7LG56|RIR2B_HUMAN | 40920.51686 | 0.64 | 0.129 | 3.12E-11 |
| sp|P20231|TRYB2_HUMAN | 30933.62793 | 0.94 | 0.323 | 0.08129 |
| sp|O95782|AP2A1_HUMAN | 108543.1504 | 0.72 | 0.069 | 2.99E-15 |
| sp|P51965|UB2E1_HUMAN | 21657.76162 | 0.82 | 0.074 | 3.47E-11 |
| sp|P62140|PP1B_HUMAN | 37942.91595 | 0.66 | 0.069 | 2.20E-16 |
| sp|Q13643|FHL3_HUMAN | 33148.34371 | 1.43 | 0.195 | 3.68E-12 |
| sp|Q9NX63|MIC19_HUMAN | 26403.32431 | 1.4 | 0.19 | 1.68E-11 |
| sp|P57103|NAC3_HUMAN | 103951.5583 | 1.25 | 0.139 | 1.01E-09 |
| sp|Q5T7V8|GORAB_HUMAN | 45289.41349 | 1.1 | 0.233 | 0.1526 |
| sp|Q9Y5J5|PHLA3_HUMAN | 14035.47755 | 2.47 | 0.632 | 7.87E-15 |
| sp|Q9P258|RCC2_HUMAN | 56772.47151 | 0.72 | 0.142 | 4.49E-09 |
| sp|P08621|RU17_HUMAN | 51564.84434 | 1.3 | 0.148 | 1.10E-10 |
| sp|P61601|NCALD_HUMAN | 22326.94526 | 0.93 | 0.124 | 0.007611 |
| sp|Q9Y4G8|RPGF2_HUMAN | 168149.8441 | 1.1 | 0.211 | 0.05686 |
| sp|E7EU14|PP5D1_HUMAN | 19877.1847 | 1.13 | 0.428 | 0.4575 |
| sp|O15127|SCAM2_HUMAN | 37063.4951 | 0.55 | 0.089 | 3.33E-16 |
| sp|Q96G01|BICD1_HUMAN | 111233.8377 | 1.44 | 0.471 | 8.60E-05 |
| sp|Q02750|MP2K1_HUMAN | 43735.40193 | 0.92 | 0.206 | 0.02183 |
| sp|Q8WYA6|CTBL1_HUMAN | 65570.25346 | 0.69 | 0.157 | 6.32E-09 |
| sp|Q8WUM4|PDC6I_HUMAN | 96572.34615 | 0.78 | 0.113 | 1.11E-08 |
| sp|P99999|CYC_HUMAN | 11837.15881 | 1.11 | 0.385 | 0.4366 |
| sp|P33897|ABCD1_HUMAN | 83379.68705 | 0.8 | 0.105 | 7.35E-09 |
| sp|Q96EY7|PTCD3_HUMAN | 79165.98247 | 0.75 | 0.1 | 4.98E-11 |
| sp|P62701|RS4X_HUMAN | 29789.12914 | 0.9 | 0.208 | 0.009041 |
| sp|P52566|GDIR2_HUMAN | 23012.61569 | 1.51 | 0.309 | 1.98E-09 |
| sp|Q99729|ROAA_HUMAN | 36298.4403 | 1.31 | 0.281 | 1.33E-05 |
| sp|Q86YS6|RAB43_HUMAN | 23534.82245 | 0.87 | 0.104 | 1.99E-06 |
| sp|Q02817|MUC2_HUMAN | 552199.9512 | 0.79 | 0.585 | 0.004561 |
| sp|Q9BRJ2|RM45_HUMAN | 35595.21046 | 1.17 | 0.112 | 4.29E-08 |
| sp|Q6PIF6|MYO7B_HUMAN | 243594.291 | 0.73 | 0.192 | 4.11E-07 |
| sp|Q8TAE8|G45IP_HUMAN | 25407.23217 | 1.02 | 0.156 | 0.8816 |
| sp|Q9UMZ2|SYNRG_HUMAN | 141289.1884 | 1.46 | 0.498 | 0.0001031 |
| sp|P49916|DNLI3_HUMAN | 114299.3478 | 0.78 | 0.217 | 4.31E-05 |
| sp|P53367|ARFP1_HUMAN | 41751.61815 | 0.91 | 0.154 | 0.004423 |
| sp|Q9P2C4|TM181_HUMAN | 69945.90147 | 0.68 | 0.179 | 2.30E-07 |
| sp|Q9BTY2|FUCO2_HUMAN | 54356.24421 | 0.45 | 0.061 | 2.20E-16 |
| sp|Q13557|KCC2D_HUMAN | 56942.77992 | 1.14 | 0.24 | 0.01574 |
| sp|Q9NX00|TM160_HUMAN | 19741.04985 | 1.22 | 0.175 | 1.39E-06 |
| sp|O95425|SVIL_HUMAN | 249399.2758 | 1.05 | 0.273 | 0.7177 |
| sp|Q06830|PRDX1_HUMAN | 22306.35416 | 1.23 | 0.199 | 3.86E-06 |
| sp|Q9UPU7|TBD2B_HUMAN | 110478.2346 | 1.21 | 0.167 | 3.04E-07 |
| sp|Q9Y2P8|RCL1_HUMAN | 41254.72655 | 0.92 | 0.231 | 0.03573 |
| sp|Q9Y2H6|FND3A_HUMAN | 133745.2116 | 0.97 | 0.203 | 0.2271 |
| sp|Q13283|G3BP1_HUMAN | 52171.08739 | 1.15 | 0.104 | 1.33E-07 |
| sp|Q86SQ0|PHLB2_HUMAN | 142793.5449 | 1.28 | 0.308 | 5.59E-05 |
| sp|P07902|GALT_HUMAN | 43602.68101 | 0.96 | 0.115 | 0.0646 |
| sp|Q9NR28|DBLOH_HUMAN | 27323.79508 | 0.62 | 0.087 | 4.47E-15 |
| sp|Q10570|CPSF1_HUMAN | 162018.4841 | 0.63 | 0.098 | 9.36E-14 |
| sp|Q9Y4F1|FARP1_HUMAN | 119281.9921 | 0.68 | 0.158 | 4.48E-09 |
| sp|Q9Y446|PKP3_HUMAN | 87467.15378 | 0.8 | 0.274 | 0.0002136 |
| sp|P53621|COPA_HUMAN | 139779.4557 | 0.66 | 0.089 | 3.17E-14 |
| sp|Q9BYC8|RM32_HUMAN | 21715.20462 | 0.73 | 0.186 | 5.81E-07 |
| sp|Q16831|UPP1_HUMAN | 34635.46753 | 0.69 | 0.357 | 7.49E-05 |
| sp|Q9NNW7|TRXR2_HUMAN | 57137.8881 | 0.9 | 0.199 | 0.008212 |
| sp|P04278|SHBG_HUMAN | 43961.89084 | 1.26 | 0.303 | 0.0001261 |
| sp|Q9Y5W9|SNX11_HUMAN | 30852.40022 | 0.76 | 0.164 | 8.78E-07 |
| sp|Q9Y508|RN114_HUMAN | 26628.63079 | 0.93 | 0.304 | 0.0654 |
| sp|Q9H0M0|WWP1_HUMAN | 105973.2863 | 1.96 | 1.016 | 0.0002576 |
| sp|Q8N465|D2HDH_HUMAN | 57046.45962 | 0.56 | 0.148 | 8.18E-11 |
| sp|Q7Z478|DHX29_HUMAN | 156204.0383 | 0.9 | 0.162 | 0.003441 |
| sp|P01042|KNG1_HUMAN | 72977.54816 | 1.16 | 0.338 | 0.0885 |
| sp|P40925|MDHC_HUMAN | 36613.09544 | 0.79 | 0.125 | 3.69E-08 |
| sp|Q2TAM9|TUSC1_HUMAN | 23471.85645 | 1.07 | 0.206 | 0.1379 |
| sp|O60524|NEMF_HUMAN | 123316.2264 | 0.9 | 0.095 | 4.63E-05 |
| sp|Q8NEU8|DP13B_HUMAN | 74941.46432 | 1.28 | 0.246 | 3.68E-06 |
| sp|P49792|RBP2_HUMAN | 362346.6954 | 1.02 | 0.118 | 0.7002 |
| sp|P11233|RALA_HUMAN | 23705.02593 | 1.16 | 0.323 | 0.05536 |
| sp|Q9Y4K1|CRBG1_HUMAN | 190079.3601 | 1.14 | 0.126 | 2.24E-05 |
| sp|Q9BWW4|SSBP3_HUMAN | 40547.91755 | 1.15 | 0.291 | 0.03757 |
| sp|P18283|GPX2_HUMAN | 22151.18418 | 0.62 | 0.377 | 4.64E-06 |
| sp|Q96A29|FUCT1_HUMAN | 40277.9657 | 0.53 | 0.141 | 1.18E-12 |
| sp|Q9ULA0|DNPEP_HUMAN | 53004.14808 | 0.9 | 0.116 | 0.0001517 |
| sp|P20594|ANPRB_HUMAN | 117785.3129 | 0.53 | 0.188 | 6.94E-09 |
| sp|P13761|2B17_HUMAN | 30127.07603 | 2.25 | 1.807 | 0.01058 |
| sp|Q8IVU3|HERC6_HUMAN | 116516.8643 | 0.66 | 0.263 | 4.35E-05 |
| sp|Q9ULF5|S39AA_HUMAN | 94910.01901 | 1 | 0.411 | 0.361 |
| sp|Q9Y5Y7|LYVE1_HUMAN | 35743.16129 | 2.46 | 1.387 | 2.38E-06 |
| sp|P23246|SFPQ_HUMAN | 76197.65902 | 1.39 | 0.126 | 2.29E-15 |
| sp|Q13409|DC1I2_HUMAN | 71793.24748 | 1.16 | 0.115 | 1.75E-07 |
| sp|P62244|RS15A_HUMAN | 14925.9998 | 0.49 | 0.072 | 2.20E-16 |
| sp|Q9NSI8|SAMN1_HUMAN | 41949.48034 | 1.72 | 1.052 | 0.0004751 |
| sp|P21217|FUT3_HUMAN | 42471.37843 | 0.6 | 0.175 | 5.09E-10 |
| sp|Q13423|NNTM_HUMAN | 114545.8192 | 0.63 | 0.125 | 8.17E-12 |
| sp|Q9BRG2|SH23A_HUMAN | 63605.7111 | 0.75 | 0.115 | 1.33E-09 |
| sp|Q8TDB6|DTX3L_HUMAN | 84566.67012 | 1.2 | 0.231 | 0.0001864 |
| sp|Q969S9|RRF2M_HUMAN | 87383.40123 | 0.97 | 0.116 | 0.1178 |
| sp|Q9BWS9|CHID1_HUMAN | 45065.34716 | 1.05 | 0.172 | 0.2212 |
| sp|Q96IV0|NGLY1_HUMAN | 75465.82927 | 0.85 | 0.137 | 1.44E-05 |
| sp|Q8NFU3|TSTD1_HUMAN | 12561.41042 | 0.88 | 0.157 | 0.0004346 |
| sp|Q58WW2|DCAF6_HUMAN | 97240.70279 | 1.21 | 0.147 | 1.43E-07 |
| sp|P49591|SYSC_HUMAN | 59235.22992 | 1.06 | 0.155 | 0.1161 |
| sp|Q5F1R6|DJC21_HUMAN | 62427.43405 | 0.99 | 0.12 | 0.4984 |
| sp|Q9HC16|ABC3G_HUMAN | 47214.83355 | 0.77 | 0.155 | 7.23E-08 |
| sp|O15020|SPTN2_HUMAN | 272507.6203 | 1.04 | 0.206 | 0.5315 |
| sp|Q92484|ASM3A_HUMAN | 51722.86854 | 0.86 | 0.219 | 0.00153 |
| sp|P08476|INHBA_HUMAN | 48192.29717 | 1.03 | 0.34 | 0.7147 |
| sp|Q96QR8|PURB_HUMAN | 33373.532 | 1.55 | 0.212 | 4.13E-14 |
| sp|Q96JP5|ZFP91_HUMAN | 64243.05577 | 1.3 | 0.221 | 7.69E-08 |
| sp|Q92629|SGCD_HUMAN | 32374.97041 | 0.51 | 0.191 | 1.76E-10 |
| sp|Q9NR46|SHLB2_HUMAN | 44156.67523 | 1.08 | 0.106 | 0.0006764 |
| sp|Q8TF68|ZN384_HUMAN | 64301.27144 | 1.42 | 0.231 | 9.00E-11 |
| sp|P62995|TRA2B_HUMAN | 33741.59119 | 1.49 | 0.219 | 4.09E-12 |
| sp|Q9UQE7|SMC3_HUMAN | 141834.9757 | 1.02 | 0.11 | 0.5016 |
| sp|Q8N0U8|VKORL_HUMAN | 20089.77308 | 0.45 | 0.11 | 6.45E-15 |
| sp|Q96CT7|CC124_HUMAN | 25801.5507 | 1.49 | 0.456 | 1.76E-06 |
| sp|Q9ULG1|INO80_HUMAN | 177537.5198 | 0.71 | 0.089 | 2.55E-13 |
| sp|Q92845|KIFA3_HUMAN | 91926.73957 | 0.61 | 0.235 | 7.01E-08 |
| sp|P23284|PPIB_HUMAN | 23766.54646 | 0.89 | 0.141 | 0.0005903 |
| sp|Q8N3F8|MILK1_HUMAN | 94334.48902 | 1.1 | 0.151 | 0.006203 |
| sp|Q9NRD5|PICK1_HUMAN | 47008.78891 | 0.55 | 0.168 | 1.50E-10 |
| sp|Q8TBZ0|CC110_HUMAN | 97217.35972 | 1.79 | 1.166 | 0.0005742 |
| sp|P53794|SC5A3_HUMAN | 80478.61104 | 0.57 | 0.173 | 3.49E-09 |
| sp|P84101|SERF2_HUMAN | 6877.627475 | 1.29 | 0.659 | 0.09817 |
| sp|Q9UPQ9|TNR6B_HUMAN | 194720.5835 | 1.33 | 0.127 | 3.10E-13 |
| sp|P0DMV8|HS71A_HUMAN | 70276.13388 | 0.98 | 0.143 | 0.3751 |
| sp|P82675|RT05_HUMAN | 48471.28007 | 1.13 | 0.189 | 0.005422 |
| sp|P53803|RPAB4_HUMAN | 7209.570091 | 1.12 | 0.261 | 0.06447 |
| sp|Q6PRD1|GP179_HUMAN | 260605.3173 | 0.98 | 0.134 | 0.2544 |
| sp|Q9H190|SDCB2_HUMAN | 31726.80638 | 1.02 | 0.368 | 0.5876 |
| sp|P51164|ATP4B_HUMAN | 33840.59779 | 0.89 | 0.348 | 0.05385 |
| sp|Q969H8|MYDGF_HUMAN | 18879.34609 | 1.52 | 0.202 | 1.26E-13 |
| sp|Q15434|RBMS2_HUMAN | 44140.76491 | 2.41 | 1.061 | 1.04E-09 |
| sp|Q8NI35|INADL_HUMAN | 197027.6826 | 0.93 | 0.337 | 0.08645 |
| sp|O95831|AIFM1_HUMAN | 67126.00347 | 1.07 | 0.257 | 0.4677 |
| sp|Q13155|AIMP2_HUMAN | 35650.40371 | 0.66 | 0.113 | 3.29E-12 |
| sp|P43246|MSH2_HUMAN | 105399.9642 | 0.89 | 0.174 | 0.002199 |
| sp|P17706|PTN2_HUMAN | 48823.51974 | 0.93 | 0.165 | 0.01987 |
| sp|O75112|LDB3_HUMAN | 78208.33871 | 0.65 | 0.31 | 8.55E-07 |
| sp|Q86T65|DAAM2_HUMAN | 124257.6716 | 0.84 | 0.187 | 8.06E-05 |
| sp|P43121|MUC18_HUMAN | 72513.95055 | 1.01 | 0.289 | 0.6136 |
| sp|O95866|G6B_HUMAN | 26357.12616 | 0.97 | 0.386 | 0.2005 |
| sp|Q9Y285|SYFA_HUMAN | 57566.52067 | 0.85 | 0.132 | 1.60E-05 |
| sp|Q9Y5Y6|ST14_HUMAN | 96971.63755 | 0.91 | 0.174 | 0.006668 |
| sp|Q9NZT2|OGFR_HUMAN | 73832.34979 | 0.86 | 0.202 | 0.0009728 |
| sp|Q2TAL8|QRIC1_HUMAN | 86820.03546 | 1.22 | 0.18 | 1.29E-06 |
| sp|P15408|FOSL2_HUMAN | 35324.75816 | 1.45 | 0.22 | 4.37E-11 |
| sp|Q9Y6R4|M3K4_HUMAN | 183262.2267 | 0.57 | 0.07 | 2.20E-16 |
| sp|Q9H1A4|APC1_HUMAN | 218510.2076 | 1 | 0.13 | 0.7116 |
| sp|P19801|AOC1_HUMAN | 85704.84047 | 0.61 | 0.207 | 1.37E-08 |
| sp|Q96GQ7|DDX27_HUMAN | 90274.36007 | 1.13 | 0.365 | 0.2631 |
| sp|Q9P2M7|CING_HUMAN | 136514.2139 | 0.99 | 0.395 | 0.3266 |
| sp|Q86W42|THOC6_HUMAN | 38063.23077 | 0.95 | 0.121 | 0.04477 |
| sp|O60341|KDM1A_HUMAN | 93339.84837 | 0.77 | 0.08 | 4.15E-12 |
| sp|P61009|SPCS3_HUMAN | 20339.54912 | 0.54 | 0.203 | 2.76E-09 |
| sp|Q92954|PRG4_HUMAN | 152147.1032 | 1.29 | 0.569 | 0.1209 |
| sp|Q5T6V5|QSPP_HUMAN | 39441.92874 | 0.74 | 0.109 | 2.47E-10 |
| sp|Q7Z7K0|COXM1_HUMAN | 12805.41904 | 0.82 | 0.31 | 0.003323 |
| sp|Q9BQD3|KXDL1_HUMAN | 19751.57678 | 1.49 | 0.332 | 1.17E-08 |
| sp|Q99795|GPA33_HUMAN | 36218.71536 | 0.8 | 0.211 | 7.46E-05 |
| sp|Q13637|RAB32_HUMAN | 25191.76681 | 0.54 | 0.117 | 1.02E-13 |
| sp|A0A0B4J1U7|HV601_HUMAN | 13568.87108 | 1.09 | 0.406 | 0.6998 |
| sp|Q12899|TRI26_HUMAN | 62907.36603 | 0.92 | 0.165 | 0.00932 |
| sp|Q9P2B4|CT2NL_HUMAN | 70552.95552 | 1.73 | 0.227 | 2.20E-16 |
| sp|O75843|AP1G2_HUMAN | 87898.99118 | 0.73 | 0.193 | 2.98E-07 |
| sp|O95967|FBLN4_HUMAN | 51749.16001 | 1.06 | 0.195 | 0.3039 |
| sp|Q9UBT2|SAE2_HUMAN | 71731.45025 | 0.86 | 0.139 | 2.22E-05 |
| sp|P30153|2AAA_HUMAN | 66047.20322 | 0.67 | 0.047 | 2.20E-16 |
| sp|Q5T0W9|FA83B_HUMAN | 115166.7047 | 1.12 | 0.154 | 0.001271 |
| sp|Q9NPH2|INO1_HUMAN | 61524.28372 | 1.01 | 0.226 | 0.8072 |
| sp|P40426|PBX3_HUMAN | 47427.19171 | 0.98 | 0.16 | 0.2946 |
| sp|Q9NWZ5|UCKL1_HUMAN | 61483.65303 | 0.7 | 0.048 | 2.20E-16 |
| sp|Q04206|TF65_HUMAN | 60676.55951 | 1.14 | 0.06 | 8.78E-12 |
| sp|Q02833|RASF7_HUMAN | 40529.7522 | 0.88 | 0.177 | 0.001034 |
| sp|Q9Y6F6|MRVI1_HUMAN | 98478.65357 | 0.86 | 0.243 | 0.001747 |
| sp|Q96S15|WDR24_HUMAN | 103901.4832 | 0.95 | 0.108 | 0.0191 |
| sp|P12956|XRCC6_HUMAN | 70066.15106 | 1.08 | 0.186 | 0.1016 |
| sp|Q96G28|CFA36_HUMAN | 39574.92691 | 1.09 | 0.171 | 0.02466 |
| sp|P63172|DYLT1_HUMAN | 12654.07764 | 0.86 | 0.858 | 0.006103 |
| sp|P40306|PSB10_HUMAN | 29185.16271 | 1.42 | 0.451 | 1.04E-05 |
| sp|P20337|RAB3B_HUMAN | 24952.18585 | 0.58 | 0.216 | 4.04E-07 |
| sp|Q86TU7|SETD3_HUMAN | 67539.26922 | 0.77 | 0.122 | 4.71E-09 |
| sp|Q86WU2|LDHD_HUMAN | 55673.02338 | 1.61 | 1.094 | 0.03004 |
| sp|O95210|STBD1_HUMAN | 39193.1149 | 1.07 | 0.196 | 0.1958 |
| sp|Q93009|UBP7_HUMAN | 129285.7306 | 0.81 | 0.128 | 7.03E-07 |
| sp|P02787|TRFE_HUMAN | 79276.47801 | 0.99 | 0.216 | 0.4561 |
| sp|Q09013|DMPK_HUMAN | 69836.44978 | 0.8 | 0.225 | 0.0001347 |
| sp|Q9GZT3|SLIRP_HUMAN | 12380.43491 | 1.12 | 0.19 | 0.005245 |
| sp|Q9HAU0|PKHA5_HUMAN | 127993.98 | 1.1 | 0.18 | 0.01762 |
| sp|Q9H2U1|DHX36_HUMAN | 115582.1307 | 0.97 | 0.068 | 0.0373 |
| sp|Q8NFW8|NEUA_HUMAN | 49014.89505 | 0.77 | 0.123 | 4.36E-09 |
| sp|Q6ZVX7|FBX50_HUMAN | 30923.91392 | 0.92 | 0.248 | 0.02753 |
| sp|P07919|QCR6_HUMAN | 10999.0369 | 1.41 | 0.302 | 2.01E-07 |
| sp|Q9BRK5|CAB45_HUMAN | 41876.49516 | 1.37 | 0.294 | 4.58E-07 |
| sp|O14613|BORG1_HUMAN | 22508.46713 | 0.68 | 0.258 | 2.92E-05 |
| sp|Q13126|MTAP_HUMAN | 31711.02545 | 1.09 | 0.166 | 0.02582 |
| sp|Q9H0S4|DDX47_HUMAN | 50881.81818 | 0.9 | 0.147 | 0.001288 |
| sp|Q9UI14|PRAF1_HUMAN | 20787.80262 | 0.64 | 0.105 | 4.70E-13 |
| sp|P35442|TSP2_HUMAN | 133767.2214 | 3.11 | 1.24 | 6.59E-11 |
| sp|Q86X83|COMD2_HUMAN | 22770.08546 | 0.69 | 0.087 | 1.41E-13 |
| sp|P41223|BUD31_HUMAN | 17540.7935 | 0.95 | 0.16 | 0.07925 |
| sp|Q9H477|RBSK_HUMAN | 34559.44552 | 0.87 | 0.273 | 0.005081 |
| sp|P53597|SUCA_HUMAN | 36607.98475 | 1.07 | 0.166 | 0.1025 |
| sp|P10746|HEM4_HUMAN | 29047.75266 | 1.24 | 0.188 | 5.62E-07 |
| sp|Q6IQ23|PKHA7_HUMAN | 127608.4249 | 1.07 | 0.315 | 0.697 |
| sp|P13929|ENOB_HUMAN | 47281.4425 | 0.81 | 0.157 | 4.93E-06 |
| sp|Q8NE71|ABCF1_HUMAN | 96304.63675 | 1.07 | 0.079 | 0.0003869 |
| sp|P60842|IF4A1_HUMAN | 46334.63092 | 0.75 | 0.104 | 7.21E-11 |
| sp|Q12756|KIF1A_HUMAN | 192522.7422 | 0.86 | 0.122 | 8.06E-06 |
| sp|Q5U5X0|LYRM7_HUMAN | 11986.48847 | 1.15 | 0.264 | 0.02329 |
| sp|Q6P1X5|TAF2_HUMAN | 138347.636 | 0.49 | 0.066 | 2.20E-16 |
| sp|P08865|RSSA_HUMAN | 32929.46547 | 1.19 | 0.106 | 1.75E-09 |
| sp|P38159|RBMX_HUMAN | 42288.32312 | 1.48 | 0.27 | 3.86E-10 |
| sp|Q8WZA1|PMGT1_HUMAN | 75756.46127 | 0.54 | 0.099 | 2.40E-15 |
| sp|Q99627|CSN8_HUMAN | 23249.97987 | 1.35 | 0.086 | 2.20E-16 |
| sp|Q9UNT1|RBL2B_HUMAN | 26237.76175 | 1.44 | 0.488 | 0.0001183 |
| sp|P04440|DPB1_HUMAN | 29407.84995 | 0.94 | 0.444 | 0.092 |
| sp|Q9HBL8|NMRL1_HUMAN | 33476.36023 | 0.85 | 0.084 | 1.81E-08 |
| sp|Q9BQA9|CYBC1_HUMAN | 20856.81585 | 0.84 | 0.087 | 2.46E-08 |
| sp|Q16222|UAP1_HUMAN | 59112.98617 | 0.8 | 0.129 | 3.34E-07 |
| sp|Q07666|KHDR1_HUMAN | 48293.21527 | 1.31 | 0.209 | 1.51E-08 |
| sp|O75449|KTNA1_HUMAN | 56196.41721 | 1.27 | 0.275 | 1.43E-05 |
| sp|P56378|68MP_HUMAN | 6639.581593 | 0.95 | 0.31 | 0.1034 |
| sp|Q68DQ2|CRBG3_HUMAN | 333088.7605 | 1.14 | 0.18 | 0.001734 |
| sp|P62277|RS13_HUMAN | 17193.65601 | 1.16 | 0.231 | 0.003478 |
| sp|Q92817|EVPL_HUMAN | 232756.4768 | 1.4 | 0.534 | 0.001216 |
| sp|Q9NVQ4|FAIM1_HUMAN | 20298.18257 | 1.07 | 0.129 | 0.02721 |
| sp|Q8NBX0|SCPDL_HUMAN | 47445.56762 | 0.59 | 0.156 | 2.80E-10 |
| sp|P49441|INPP_HUMAN | 44522.45131 | 0.79 | 0.27 | 0.0009505 |
| sp|P07355|ANXA2_HUMAN | 38789.89006 | 1.54 | 0.314 | 8.05E-11 |
| sp|A6NFA0|F205C_HUMAN | 38198.12233 | 0.78 | 0.279 | 0.0003439 |
| sp|Q68CQ7|GL8D1_HUMAN | 42005.06041 | 0.94 | 0.135 | 0.02115 |
| sp|Q9NWU1|OXSM_HUMAN | 49420.99598 | 1.02 | 0.276 | 0.7563 |
| sp|Q8NAN2|MIGA1_HUMAN | 71911.80059 | 0.82 | 0.158 | 6.80E-06 |
| sp|O94916|NFAT5_HUMAN | 166611.2559 | 0.86 | 0.162 | 0.0002016 |
| sp|P22352|GPX3_HUMAN | 25746.9793 | 0.96 | 0.318 | 0.1578 |
| sp|P16444|DPEP1_HUMAN | 46083.15122 | 1.1 | 0.223 | 0.07603 |
| sp|P27448|MARK3_HUMAN | 84871.25209 | 0.72 | 0.095 | 1.33E-11 |
| sp|Q8N4N3|KLH36_HUMAN | 70803.00695 | 0.85 | 0.136 | 2.90E-05 |
| sp|O00422|SAP18_HUMAN | 17588.97887 | 1.53 | 0.348 | 4.43E-09 |
| sp|Q15326|ZMY11_HUMAN | 72324.64892 | 0.91 | 0.162 | 0.005193 |
| sp|Q9Y2G8|DJC16_HUMAN | 91029.67519 | 0.61 | 0.172 | 1.70E-09 |
| sp|P11310|ACADM_HUMAN | 46996.74366 | 1.49 | 0.378 | 2.62E-07 |
| sp|Q9UNY4|TTF2_HUMAN | 130744.1845 | 0.91 | 0.231 | 0.0245 |
| sp|Q02218|ODO1_HUMAN | 117040.8394 | 0.76 | 0.095 | 1.02E-10 |
| sp|P13498|CY24A_HUMAN | 21095.16609 | 0.64 | 0.32 | 3.02E-06 |
| sp|Q86UV5|UBP48_HUMAN | 121047.9366 | 0.88 | 0.313 | 0.01761 |
| sp|P48728|GCST_HUMAN | 44469.83957 | 0.78 | 0.286 | 0.0003572 |
| sp|O75306|NDUS2_HUMAN | 52892.86339 | 0.76 | 0.171 | 1.48E-06 |
| sp|Q641Q2|WAC2A_HUMAN | 147247.945 | 1.67 | 0.305 | 1.63E-12 |
| sp|Q12768|WASC5_HUMAN | 135095.1879 | 0.69 | 0.084 | 2.62E-14 |
| sp|Q8IUC4|RHPN2_HUMAN | 77553.62636 | 0.7 | 0.169 | 1.59E-08 |
| sp|Q9NWB6|ARGL1_HUMAN | 33178.81654 | 1.54 | 0.201 | 6.46E-14 |
| sp|Q9UIQ6|LCAP_HUMAN | 117769.4132 | 0.47 | 0.066 | 2.20E-16 |
| sp|Q9BSE5|SPEB_HUMAN | 38188.38653 | 0.91 | 0.281 | 0.04371 |
| sp|O95825|QORL1_HUMAN | 39053.16879 | 0.91 | 0.166 | 0.005648 |
| sp|O75312|ZPR1_HUMAN | 51445.20893 | 1.19 | 0.15 | 1.59E-06 |
| sp|O95260|ATE1_HUMAN | 59946.53414 | 0.82 | 0.138 | 6.13E-06 |
| sp|O95760|IL33_HUMAN | 31234.66015 | 1.15 | 0.57 | 0.5893 |
| sp|Q29RF7|PDS5A_HUMAN | 152255.8657 | 0.69 | 0.104 | 6.60E-12 |
| sp|P30825|CTR1_HUMAN | 68431.44896 | 0.71 | 0.166 | 9.86E-07 |
| sp|Q9BUP3|HTAI2_HUMAN | 27242.06269 | 0.79 | 0.129 | 4.34E-08 |
| sp|O94989|ARHGF_HUMAN | 92834.43475 | 1.23 | 0.237 | 5.80E-05 |
| sp|Q9UQ84|EXO1_HUMAN | 95167.01298 | 1.52 | 0.205 | 3.48E-14 |
| sp|P24390|ERD21_HUMAN | 24736.10401 | 0.73 | 0.27 | 3.34E-05 |
| sp|A5PL33|KRBA1_HUMAN | 108952.4846 | 1.04 | 0.268 | 0.8528 |
| sp|P20962|PTMS_HUMAN | 11505.17723 | 1.64 | 0.296 | 1.67E-12 |
| sp|Q9BY15|AGRE3_HUMAN | 74152.20305 | 0.71 | 0.473 | 0.0003137 |
| sp|O94876|TMCC1_HUMAN | 72419.60114 | 0.65 | 0.127 | 4.71E-11 |
| sp|Q96IZ0|PAWR_HUMAN | 36641.47502 | 1.49 | 0.388 | 2.51E-07 |
| sp|Q9BSG0|PADC1_HUMAN | 21295.67182 | 1.06 | 0.338 | 0.7546 |
| sp|Q9H410|DSN1_HUMAN | 40366.20632 | 1.16 | 0.114 | 1.14E-07 |
| sp|P23434|GCSH_HUMAN | 19082.69394 | 1.51 | 0.447 | 4.01E-06 |
| sp|Q9BZV1|UBXN6_HUMAN | 49989.98504 | 1.2 | 0.129 | 1.76E-08 |
| sp|Q07954|LRP1_HUMAN | 523131.9455 | 1.25 | 0.227 | 1.99E-06 |
| sp|Q6ZRQ5|MMS22_HUMAN | 144092.1258 | 1.98 | 0.778 | 8.62E-08 |
| sp|Q9BXX0|EMIL2_HUMAN | 116851.398 | 1.42 | 0.468 | 3.51E-05 |
| sp|Q04759|KPCT_HUMAN | 83389.26845 | 1.02 | 0.263 | 0.7987 |
| sp|O76039|CDKL5_HUMAN | 116132.6717 | 0.98 | 0.162 | 0.3541 |
| sp|P36980|FHR2_HUMAN | 31524.96686 | 0.75 | 0.469 | 0.001047 |
| sp|P17405|ASM_HUMAN | 70899.72658 | 0.57 | 0.119 | 1.51E-12 |
| sp|P40394|ADH7_HUMAN | 42234.55525 | 0.59 | 0.271 | 8.42E-05 |
| sp|P39656|OST48_HUMAN | 50922.02398 | 0.63 | 0.106 | 7.18E-13 |
| sp|Q9H5V9|CX056_HUMAN | 25989.13629 | 1.17 | 0.291 | 0.01913 |
| sp|Q96KA5|CLP1L_HUMAN | 62513.13323 | 0.91 | 0.477 | 0.0342 |
| sp|Q96K21|ANCHR_HUMAN | 52408.59237 | 0.99 | 0.236 | 0.4748 |
| sp|Q9NW08|RPC2_HUMAN | 129223.8985 | 0.63 | 0.076 | 4.12E-16 |
| sp|P49643|PRI2_HUMAN | 59207.19901 | 1.56 | 2.489 | 0.05602 |
| sp|P13224|GP1BB_HUMAN | 22255.96506 | 0.58 | 0.233 | 3.73E-07 |
| sp|P14625|ENPL_HUMAN | 92678.44091 | 1.23 | 0.138 | 4.82E-09 |
| sp|Q13472|TOP3A_HUMAN | 114221.1296 | 0.69 | 0.117 | 3.77E-10 |
| sp|Q9BZG8|DPH1_HUMAN | 49326.46151 | 0.72 | 0.183 | 6.28E-07 |
| sp|P18621|RL17_HUMAN | 21593.40796 | 1.41 | 0.331 | 1.40E-06 |
| sp|Q16526|CRY1_HUMAN | 67132.71805 | 1.16 | 0.434 | 0.2946 |
| sp|Q92522|H1X_HUMAN | 22455.52206 | 1.47 | 0.31 | 1.32E-08 |
| sp|Q6UXR4|SPA13_HUMAN | 35051.83852 | 0.81 | 0.269 | 0.0001825 |
| sp|Q9ULV4|COR1C_HUMAN | 53881.30903 | 0.9 | 0.161 | 0.005152 |
| sp|Q9BVA6|FICD_HUMAN | 51898.129 | 1.06 | 0.14 | 0.07252 |
| sp|O95670|VATG2_HUMAN | 13634.97007 | 2.77 | 2.022 | 4.81E-07 |
| sp|P17540|KCRS_HUMAN | 47969.54127 | 1.07 | 0.199 | 0.2232 |
| sp|Q9NPE3|NOP10_HUMAN | 7739.97957 | 1.34 | 0.309 | 1.14E-05 |
| sp|Q8IV38|ANKY2_HUMAN | 50275.04169 | 0.85 | 0.198 | 6.00E-04 |
| sp|Q00G26|PLIN5_HUMAN | 51425.9348 | 1.13 | 0.453 | 0.602 |
| sp|P08758|ANXA5_HUMAN | 35953.41188 | 1.03 | 0.113 | 0.213 |
| sp|O75152|ZC11A_HUMAN | 89913.32433 | 1.16 | 0.121 | 4.13E-07 |
| sp|P52701|MSH6_HUMAN | 154495.5339 | 0.8 | 0.14 | 4.26E-07 |
| sp|Q96LD8|SENP8_HUMAN | 24359.03918 | 1.06 | 0.246 | 0.4185 |
| sp|Q6NXT4|ZNT6_HUMAN | 51406.89599 | 0.63 | 0.147 | 1.21E-10 |
| sp|Q9Y303|NAGA_HUMAN | 44101.7152 | 1.47 | 0.208 | 7.88E-13 |
| sp|O43586|PPIP1_HUMAN | 47999.7912 | 1.18 | 0.181 | 3.95E-05 |
| sp|Q9Y2R5|RT17_HUMAN | 14588.96342 | 1.1 | 0.294 | 0.3455 |
| sp|P08174|DAF_HUMAN | 42381.95368 | 0.73 | 0.437 | 0.0006858 |
| sp|Q86YL5|TDRP_HUMAN | 20429.31752 | 0.64 | 0.25 | 3.82E-07 |
| sp|Q86X02|CDR2L_HUMAN | 53359.14673 | 0.99 | 0.204 | 0.415 |
| sp|P05107|ITB2_HUMAN | 87958.3398 | 0.72 | 0.327 | 9.97E-05 |
| sp|Q02040|AK17A_HUMAN | 81295.11385 | 0.92 | 0.28 | 0.04582 |
| sp|Q00577|PURA_HUMAN | 34985.42979 | 1.33 | 0.384 | 0.0001622 |
| sp|P61224|RAP1B_HUMAN | 21021.66804 | 0.92 | 0.105 | 0.0003705 |
| sp|P78316|NOP14_HUMAN | 98273.59704 | 1.12 | 0.179 | 0.003114 |
| sp|Q6UWZ7|ABRX1_HUMAN | 47014.54858 | 1.33 | 0.283 | 2.20E-06 |
| sp|Q5VTR2|BRE1A_HUMAN | 114201.5286 | 1.19 | 0.126 | 3.34E-08 |
| sp|P55036|PSMD4_HUMAN | 40921.27378 | 1.68 | 0.259 | 2.40E-14 |
| sp|Q9P206|K1522_HUMAN | 107411.0353 | 1.09 | 0.356 | 0.6289 |
| sp|P51153|RAB13_HUMAN | 22969.81632 | 0.58 | 0.125 | 1.22E-12 |
| sp|Q99943|PLCA_HUMAN | 32019.79023 | 0.69 | 0.105 | 5.75E-11 |
| sp|P14210|HGF_HUMAN | 85399.09833 | 1.26 | 0.159 | 5.71E-09 |
| sp|Q9HC35|EMAL4_HUMAN | 109913.6989 | 0.92 | 0.144 | 0.006818 |
| sp|Q96RR4|KKCC2_HUMAN | 65542.00735 | 1.26 | 0.199 | 4.37E-07 |
| sp|P01593|KVD33_HUMAN | 12993.32244 | 2.24 | 1.178 | 1.18E-07 |
| sp|Q16658|FSCN1_HUMAN | 55105.27773 | 1.39 | 0.231 | 1.94E-09 |
| sp|P42331|RHG25_HUMAN | 73883.97152 | 1.38 | 0.297 | 2.11E-07 |
| sp|A4FU01|MTMRB_HUMAN | 80161.73672 | 0.92 | 0.132 | 0.003281 |
| sp|Q9NR45|SIAS_HUMAN | 40719.61475 | 1.11 | 0.287 | 0.1441 |
| sp|Q9NRY6|PLS3_HUMAN | 32351.22255 | 0.72 | 0.105 | 1.48E-10 |
| sp|Q9NR48|ASH1L_HUMAN | 336156.2925 | 0.57 | 0.078 | 2.20E-16 |
| sp|O75396|SC22B_HUMAN | 24787.60771 | 0.86 | 0.121 | 7.29E-06 |
| sp|Q9UBP6|TRMB_HUMAN | 31604.16187 | 0.69 | 0.074 | 4.87E-15 |
| sp|Q9BW83|IFT27_HUMAN | 20734.42686 | 1.29 | 0.217 | 4.06E-07 |
| sp|Q969S3|ZN622_HUMAN | 54789.60591 | 1.09 | 0.101 | 0.0002552 |
| sp|Q9BZH6|WDR11_HUMAN | 138404.7365 | 0.38 | 0.13 | 8.48E-14 |
| sp|P52630|STAT2_HUMAN | 98577.84389 | 0.82 | 0.092 | 2.57E-09 |
| sp|P21462|FPR1_HUMAN | 38801.5337 | 1.16 | 0.269 | 0.01171 |
| sp|P63092|GNAS2_HUMAN | 46074.06198 | 0.66 | 0.082 | 5.25E-15 |
| sp|O75832|PSD10_HUMAN | 24679.47844 | 1.42 | 0.109 | 2.20E-16 |
| sp|Q9Y6X9|MORC2_HUMAN | 118530.0074 | 0.94 | 0.195 | 0.05606 |
| sp|P28827|PTPRM_HUMAN | 165328.0409 | 1.37 | 0.331 | 2.42E-06 |
| sp|Q8TAF3|WDR48_HUMAN | 76999.73332 | 0.95 | 0.148 | 0.0707 |
| sp|Q96S86|HPLN3_HUMAN | 41420.61062 | 1.34 | 0.237 | 2.26E-08 |
| sp|Q9BQG2|NUD12_HUMAN | 52765.95358 | 0.82 | 0.157 | 8.90E-06 |
| sp|P22061|PIMT_HUMAN | 24773.62511 | 1.45 | 0.253 | 3.17E-10 |
| sp|P28065|PSB9_HUMAN | 23345.56982 | 1.14 | 0.12 | 2.40E-06 |
| sp|O75298|RTN2_HUMAN | 59437.60358 | 0.57 | 0.201 | 2.29E-09 |
| sp|Q8NFD5|ARI1B_HUMAN | 237039.2663 | 1.08 | 0.126 | 0.006554 |
| sp|Q7L2J0|MEPCE_HUMAN | 74918.8667 | 1.1 | 0.278 | 0.218 |
| sp|O95248|MTMR5_HUMAN | 210404.1242 | 0.85 | 0.078 | 2.28E-09 |
| sp|P50895|BCAM_HUMAN | 68142.88167 | 1.17 | 0.629 | 0.5715 |
| sp|Q96RP9|EFGM_HUMAN | 84084.68985 | 0.98 | 0.226 | 0.3502 |
| sp|Q13043|STK4_HUMAN | 55804.94392 | 1.39 | 0.292 | 2.66E-07 |
| sp|Q15438|CYH1_HUMAN | 46821.52448 | 0.88 | 0.172 | 0.00112 |
| sp|P82979|SARNP_HUMAN | 23695.44041 | 1.72 | 0.71 | 5.93E-07 |
| sp|P21266|GSTM3_HUMAN | 26980.29888 | 0.47 | 0.179 | 4.10E-11 |
| sp|Q9UHV7|MED13_HUMAN | 242661.5891 | 0.96 | 0.238 | 0.1892 |
| sp|Q15814|TBCC_HUMAN | 39718.98132 | 1.09 | 0.101 | 0.0003062 |
| sp|Q13454|TUSC3_HUMAN | 39974.58822 | 0.83 | 0.159 | 2.57E-05 |
| sp|Q96CW6|S7A6O_HUMAN | 35216.78853 | 1.72 | 0.27 | 1.16E-14 |
| sp|Q9H1H9|KI13A_HUMAN | 203818.3675 | 0.96 | 0.127 | 0.07008 |
| sp|P13637|AT1A3_HUMAN | 113084.4374 | 0.62 | 0.236 | 1.85E-07 |
| sp|Q99719|SEPT5_HUMAN | 43187.84866 | 1.2 | 0.256 | 0.00102 |
| sp|Q86U42|PABP2_HUMAN | 32825.02457 | 1.12 | 0.115 | 3.39E-05 |
| sp|Q9NZJ6|COQ3_HUMAN | 41523.19333 | 0.94 | 0.281 | 0.08713 |
| sp|Q9Y2I7|FYV1_HUMAN | 239591.1351 | 0.95 | 0.227 | 0.09326 |
| sp|Q8TEW8|PAR3L_HUMAN | 133078.7672 | 1.15 | 0.27 | 0.02057 |
| sp|Q8N8V4|ANS4B_HUMAN | 46892.2458 | 0.99 | 0.517 | 0.223 |
| sp|Q6WKZ4|RFIP1_HUMAN | 137463.9962 | 1.28 | 0.28 | 4.82E-05 |
| sp|Q16719|KYNU_HUMAN | 52813.36843 | 0.86 | 0.183 | 0.0005004 |
| sp|Q96T23|RSF1_HUMAN | 165241.7244 | 1.27 | 0.178 | 1.51E-08 |
| sp|Q9BRA2|TXD17_HUMAN | 14198.89296 | 1.07 | 0.06 | 7.78E-06 |
| sp|P00390|GSHR_HUMAN | 56773.15488 | 0.93 | 0.158 | 0.0219 |
| sp|O75879|GATB_HUMAN | 62263.40194 | 0.75 | 0.203 | 8.23E-06 |
| sp|Q8TAQ2|SMRC2_HUMAN | 133177.891 | 1.15 | 0.126 | 4.53E-06 |
| sp|Q9UJ41|RABX5_HUMAN | 80557.20016 | 1.16 | 0.092 | 2.33E-09 |
| sp|P40261|NNMT_HUMAN | 29993.22119 | 1.1 | 0.389 | 0.6478 |
| sp|Q8WWP7|GIMA1_HUMAN | 34671.75808 | 0.93 | 0.245 | 0.05736 |
| sp|Q9UM13|APC10_HUMAN | 21391.78115 | 1.54 | 0.255 | 6.67E-12 |
| sp|Q96SW2|CRBN_HUMAN | 51407.4554 | 0.92 | 0.22 | 0.03356 |
| sp|P86790|CCZ1B_HUMAN | 56268.3974 | 0.98 | 0.118 | 0.2771 |
| sp|Q06828|FMOD_HUMAN | 43475.6386 | 1.19 | 0.272 | 0.001392 |
| sp|O60508|PRP17_HUMAN | 66145.73779 | 1.08 | 0.132 | 0.008352 |
| sp|P0DN76|U2AF5_HUMAN | 28349.65111 | 1.49 | 0.404 | 4.44E-07 |
| sp|Q13488|VPP3_HUMAN | 93631.62131 | 0.66 | 0.164 | 2.92E-09 |
| sp|Q03519|TAP2_HUMAN | 76168.01456 | 0.77 | 0.221 | 1.88E-05 |
| sp|Q96L58|B3GT6_HUMAN | 37495.4341 | 0.67 | 0.129 | 1.31E-10 |
| sp|Q6UVY6|MOXD1_HUMAN | 70502.01458 | 0.84 | 0.089 | 1.49E-08 |
| sp|Q16537|2A5E_HUMAN | 55045.6256 | 0.52 | 0.17 | 2.59E-11 |
| sp|Q14678|KANK1_HUMAN | 149003.2332 | 1.25 | 0.333 | 0.0004847 |
| sp|Q86VN1|VPS36_HUMAN | 44055.83614 | 1.12 | 0.127 | 0.0001145 |
| sp|Q02108|GCYA1_HUMAN | 78695.7948 | 0.73 | 0.144 | 1.49E-08 |
| sp|Q9C0B9|ZCHC2_HUMAN | 126980.1839 | 1.02 | 0.288 | 0.7078 |
| sp|Q08AF3|SLFN5_HUMAN | 102511.5126 | 1.06 | 0.184 | 0.307 |
| sp|A4D2B0|MBLC1_HUMAN | 27281.81223 | 0.79 | 0.17 | 3.98E-06 |
| sp|P23142|FBLN1_HUMAN | 81249.88644 | 0.96 | 0.241 | 0.1468 |
| sp|P09001|RM03_HUMAN | 38875.17015 | 1.13 | 0.17 | 0.001233 |
| sp|O43189|PHF1_HUMAN | 63359.65698 | 0.92 | 0.105 | 0.0004191 |
| sp|Q2UY09|COSA1_HUMAN | 117422.1763 | 0.62 | 0.292 | 8.92E-06 |
| sp|P46100|ATRX_HUMAN | 284844.5138 | 0.83 | 0.059 | 1.84E-12 |
| sp|Q86UL3|GPAT4_HUMAN | 52475.36629 | 0.94 | 0.092 | 0.004264 |
| sp|Q9H0U3|MAGT1_HUMAN | 38392.54753 | 0.53 | 0.084 | 2.20E-16 |
| sp|Q15813|TBCE_HUMAN | 59918.22209 | 1.08 | 0.176 | 0.08464 |
| sp|O94819|KBTBB_HUMAN | 66687.15636 | 0.85 | 0.191 | 0.000387 |
| sp|P13639|EF2_HUMAN | 96228.30347 | 0.77 | 0.088 | 1.58E-11 |
| sp|Q96FX7|TRM61_HUMAN | 31685.99855 | 0.61 | 0.104 | 7.93E-14 |
| sp|P30047|GFRP_HUMAN | 9730.902314 | 0.94 | 0.156 | 0.0297 |
| sp|Q9BSQ5|CCM2_HUMAN | 49244.75499 | 1.18 | 0.179 | 3.17E-05 |
| sp|Q58EX2|SDK2_HUMAN | 240142.0836 | 0.52 | 0.203 | 6.35E-09 |
| sp|Q04721|NOTC2_HUMAN | 279064.2561 | 1.45 | 0.368 | 1.71E-07 |
| sp|Q96BS2|CHP3_HUMAN | 24830.08169 | 0.83 | 0.291 | 0.003907 |
| sp|Q9NSA3|CNBP1_HUMAN | 9146.532171 | 1.47 | 0.374 | 1.16E-06 |
| sp|P13797|PLST_HUMAN | 71261.39138 | 1.33 | 0.263 | 2.30E-06 |
| sp|P04920|B3A2_HUMAN | 137475.3307 | 0.8 | 0.217 | 7.16E-05 |
| sp|P56159|GFRA1_HUMAN | 53171.84981 | 1.11 | 0.689 | 0.5778 |
| sp|Q9NVC6|MED17_HUMAN | 73339.72047 | 0.87 | 0.131 | 3.69E-05 |
| sp|Q13952|NFYC_HUMAN | 50538.36665 | 1.36 | 0.096 | 2.20E-16 |
| sp|Q15819|UB2V2_HUMAN | 16391.24379 | 1.32 | 0.222 | 6.19E-08 |
| sp|Q8WX92|NELFB_HUMAN | 66264.63704 | 0.74 | 0.089 | 2.75E-12 |
| sp|Q15036|SNX17_HUMAN | 53135.27631 | 0.84 | 0.139 | 1.57E-05 |
| sp|P46199|IF2M_HUMAN | 81818.68631 | 1.26 | 0.304 | 0.0001675 |
| sp|P09486|SPRC_HUMAN | 35447.02038 | 1.55 | 0.32 | 5.15E-10 |
| sp|Q9NRX4|PHP14_HUMAN | 13976.74875 | 1.48 | 0.175 | 2.21E-14 |
| sp|O94901|SUN1_HUMAN | 90787.6198 | 0.92 | 0.185 | 0.0133 |
| sp|O60610|DIAP1_HUMAN | 141923.7899 | 0.88 | 0.146 | 0.0002542 |
| sp|Q9H246|CA021_HUMAN | 13952.71739 | 0.99 | 0.105 | 0.4409 |
| sp|Q12846|STX4_HUMAN | 34254.88652 | 0.82 | 0.084 | 1.02E-09 |
| sp|P40855|PEX19_HUMAN | 33052.65706 | 1.27 | 0.186 | 3.38E-08 |
| sp|O00571|DDX3X_HUMAN | 73579.13837 | 0.91 | 0.106 | 0.0005363 |
| sp|Q12849|GRSF1_HUMAN | 53587.82335 | 1.43 | 0.344 | 1.05E-06 |
| sp|Q9BXN1|ASPN_HUMAN | 43770.45512 | 1.46 | 0.796 | 0.01283 |
| sp|Q03701|CEBPZ_HUMAN | 121507.5565 | 0.82 | 0.187 | 0.0001089 |
| sp|Q8WVD5|RN141_HUMAN | 26070.60203 | 0.64 | 0.118 | 8.83E-12 |
| sp|O60825|F262_HUMAN | 58934.91132 | 0.82 | 0.121 | 1.40E-07 |
| sp|Q16762|THTR_HUMAN | 33617.91261 | 0.74 | 0.293 | 0.0001536 |
| sp|Q92733|PRCC_HUMAN | 52367.52927 | 1.2 | 0.302 | 0.007291 |
| sp|Q14746|COG2_HUMAN | 83707.5086 | 0.69 | 0.176 | 3.76E-08 |
| sp|Q13424|SNTA1_HUMAN | 54186.02479 | 2.09 | 0.894 | 2.13E-06 |
| sp|P17252|KPCA_HUMAN | 77822.54751 | 0.76 | 0.151 | 9.13E-08 |
| sp|P26447|S10A4_HUMAN | 11930.80736 | 1.5 | 0.47 | 2.56E-06 |
| sp|P08123|CO1A2_HUMAN | 129730.6257 | 2.66 | 1.032 | 1.10E-11 |
| sp|P14927|QCR7_HUMAN | 13503.96626 | 1.26 | 0.237 | 8.28E-06 |
| sp|Q9BRT6|LLPH_HUMAN | 15311.43138 | 1.5 | 0.398 | 2.74E-07 |
| sp|Q13619|CUL4A_HUMAN | 88119.57863 | 0.72 | 0.072 | 2.28E-14 |
| sp|P06576|ATPB_HUMAN | 56506.59534 | 1.21 | 0.094 | 1.06E-11 |
| sp|Q13882|PTK6_HUMAN | 52353.38734 | 0.4 | 0.199 | 5.88E-12 |
| sp|O43924|PDE6D_HUMAN | 17504.88849 | 1.11 | 0.108 | 2.97E-05 |
| sp|Q9NPF4|OSGEP_HUMAN | 36898.45519 | 0.84 | 0.126 | 2.28E-06 |
| sp|Q5HYW2|NHSL2_HUMAN | 76635.29864 | 1.08 | 0.127 | 0.006582 |
| sp|Q96EK4|THA11_HUMAN | 34757.60361 | 1.18 | 0.169 | 9.81E-06 |
| sp|Q9NVI7|ATD3A_HUMAN | 71591.81893 | 0.83 | 0.157 | 2.96E-05 |
| sp|Q99952|PTN18_HUMAN | 51059.67761 | 1.4 | 0.367 | 1.92E-06 |
| sp|Q9BUE0|MED18_HUMAN | 23742.95471 | 0.65 | 0.245 | 9.23E-07 |
| sp|P27348|1433T_HUMAN | 28013.86353 | 0.78 | 0.138 | 6.19E-08 |
| sp|P22891|PROZ_HUMAN | 46008.19144 | 1.17 | 0.443 | 0.3374 |
| sp|Q9BRR6|ADPGK_HUMAN | 54378.76956 | 0.71 | 0.134 | 4.47E-10 |
| sp|Q9Y3E2|BOLA1_HUMAN | 14433.51284 | 1.15 | 0.2 | 0.002402 |
| sp|P48047|ATPO_HUMAN | 23358.69069 | 0.97 | 0.129 | 0.1713 |
| sp|Q7Z2T5|TRM1L_HUMAN | 82874.61676 | 0.92 | 0.128 | 0.002711 |
| sp|P35226|BMI1_HUMAN | 37477.56733 | 0.79 | 0.222 | 3.41E-05 |
| sp|Q9HBI0|PARVG_HUMAN | 37557.96212 | 0.36 | 0.097 | 2.32E-16 |
| sp|P20160|CAP7_HUMAN | 27306.81616 | 0.78 | 0.366 | 0.001001 |
| sp|Q8TER5|ARH40_HUMAN | 166247.9814 | 1.02 | 0.119 | 0.5478 |
| sp|Q8N766|EMC1_HUMAN | 112127.2569 | 0.66 | 0.1 | 1.20E-12 |
| sp|P30626|SORCN_HUMAN | 21929.45421 | 1.2 | 0.197 | 2.91E-05 |
| sp|O60292|SI1L3_HUMAN | 195783.0341 | 0.9 | 0.167 | 0.005402 |
| sp|Q13094|LCP2_HUMAN | 60247.11146 | 1.2 | 0.282 | 0.00472 |
| sp|Q96G23|CERS2_HUMAN | 44943.35546 | 0.57 | 0.099 | 4.56E-15 |
| sp|Q13214|SEM3B_HUMAN | 84191.68472 | 0.98 | 0.29 | 0.3043 |
| sp|Q8NBT2|SPC24_HUMAN | 22503.43551 | 0.9 | 0.192 | 0.008521 |
| sp|Q9C002|NMES1_HUMAN | 9593.164171 | 0.96 | 0.388 | 0.1414 |
| sp|P17480|UBF1_HUMAN | 89674.34458 | 1.36 | 0.164 | 5.90E-12 |
| sp|Q15008|PSMD6_HUMAN | 45769.37016 | 0.65 | 0.095 | 7.37E-14 |
| sp|Q96SY0|INT14_HUMAN | 57986.18468 | 0.71 | 0.081 | 8.04E-14 |
| sp|Q6NUQ1|RINT1_HUMAN | 91353.84694 | 0.79 | 0.133 | 1.73E-07 |
| sp|Q9H1Y0|ATG5_HUMAN | 32636.38614 | 0.69 | 0.163 | 5.94E-08 |
| sp|P43243|MATR3_HUMAN | 95059.98044 | 1.08 | 0.102 | 0.001136 |
| sp|Q9UN76|S6A14_HUMAN | 73227.58272 | 0.98 | 0.209 | 0.292 |
| sp|O75880|SCO1_HUMAN | 34002.58855 | 1.18 | 0.331 | 0.0453 |
| sp|Q96CF2|CHM4C_HUMAN | 26376.34125 | 1.33 | 0.402 | 0.0001151 |
| sp|P34947|GRK5_HUMAN | 68694.63531 | 1.09 | 0.153 | 0.0102 |
| sp|A0A0J9YXX1|HV5X1_HUMAN | 12917.34028 | 1.28 | 0.893 | 0.9744 |
| sp|P49005|DPOD2_HUMAN | 51866.20751 | 1.08 | 0.283 | 0.3721 |
| sp|P35556|FBN2_HUMAN | 335238.6077 | 0.92 | 0.299 | 0.05984 |
| sp|Q15833|STXB2_HUMAN | 66848.65656 | 0.92 | 0.16 | 0.008318 |
| sp|Q6P2Q9|PRP8_HUMAN | 274720.0437 | 0.7 | 0.058 | 2.20E-16 |
| sp|Q96SL4|GPX7_HUMAN | 21135.82541 | 2.06 | 0.616 | 3.63E-11 |
| sp|P48506|GSH1_HUMAN | 73499.63365 | 0.75 | 0.171 | 6.03E-07 |
| sp|P08294|SODE_HUMAN | 26272.83577 | 1.31 | 0.572 | 0.0123 |
| sp|Q96S42|NODAL_HUMAN | 40088.02769 | 2.12 | 0.969 | 2.37E-08 |
| sp|O60925|PFD1_HUMAN | 14183.49765 | 1.31 | 0.16 | 3.34E-10 |
| sp|O94906|PRP6_HUMAN | 107638.085 | 0.71 | 0.127 | 9.15E-10 |
| sp|P16930|FAAA_HUMAN | 46725.3497 | 1.12 | 0.106 | 9.16E-06 |
| sp|P43304|GPDM_HUMAN | 81296.79748 | 0.79 | 0.154 | 1.36E-06 |
| sp|P78386|KRT85_HUMAN | 57288.04181 | 0.78 | 0.198 | 1.47E-05 |
| sp|O43513|MED7_HUMAN | 27437.76967 | 1.17 | 0.182 | 8.54E-05 |
| sp|P47897|SYQ_HUMAN | 88637.2064 | 0.66 | 0.092 | 7.60E-14 |
| sp|Q16514|TAF12_HUMAN | 18009.24314 | 1.64 | 0.62 | 2.58E-05 |
| sp|P08195|4F2_HUMAN | 68161.80271 | 0.61 | 0.08 | 2.72E-16 |
| sp|Q92800|EZH1_HUMAN | 87307.37561 | 1.07 | 0.443 | 0.7433 |
| sp|Q9Y257|KCNK6_HUMAN | 33992.87681 | 0.61 | 0.116 | 4.31E-13 |
| sp|O43776|SYNC_HUMAN | 63739.82827 | 0.63 | 0.202 | 2.89E-08 |
| sp|Q53EL6|PDCD4_HUMAN | 52084.18592 | 0.83 | 0.23 | 0.0009876 |
| sp|O75436|VP26A_HUMAN | 38241.77777 | 0.58 | 0.059 | 2.20E-16 |
| sp|Q5TC63|GRTP1_HUMAN | 38910.18838 | 0.86 | 0.177 | 0.0009048 |
| sp|O00468|AGRIN_HUMAN | 225314.8014 | 1.03 | 0.225 | 0.8611 |
| sp|P01903|DRA_HUMAN | 28741.74907 | 1.29 | 0.431 | 0.004374 |
| sp|O14772|FPGT_HUMAN | 68916.97642 | 0.48 | 0.159 | 8.65E-12 |
| sp|Q8IWX8|CHERP_HUMAN | 104132.2979 | 1.04 | 0.192 | 0.5284 |
| sp|A0A087WW87|KV240_HUMAN | 13397.62633 | 1.79 | 1.086 | 8.59E-05 |
| sp|O14981|BTAF1_HUMAN | 209018.517 | 1 | 0.13 | 0.7057 |
| sp|O00267|SPT5H_HUMAN | 121305.6527 | 1.14 | 0.135 | 2.95E-05 |
| sp|P55072|TERA_HUMAN | 89931.96632 | 1.31 | 0.088 | 2.47E-16 |
| sp|P50548|ERF_HUMAN | 58990.53664 | 1.27 | 0.206 | 3.85E-07 |
| sp|P06241|FYN_HUMAN | 61218.5457 | 0.83 | 0.147 | 4.77E-06 |
| sp|Q9H3Q1|BORG4_HUMAN | 37995.57632 | 1.29 | 0.264 | 9.85E-06 |
| sp|Q9BZF1|OSBL8_HUMAN | 101741.4596 | 0.65 | 0.11 | 1.72E-12 |
| sp|O15226|NKRF_HUMAN | 78290.4639 | 1.12 | 0.229 | 0.0295 |
| sp|Q9Y5J1|UTP18_HUMAN | 62402.60761 | 1.11 | 0.248 | 0.05639 |
| sp|P01768|HV330_HUMAN | 13091.49754 | 1.14 | 0.33 | 0.1192 |
| sp|P42694|HELZ_HUMAN | 220582.6534 | 0.95 | 0.232 | 0.1017 |
| sp|Q9BX59|TPSNR_HUMAN | 50703.60243 | 0.78 | 0.216 | 2.10E-05 |
| sp|O60256|KPRB_HUMAN | 41280.49581 | 0.78 | 0.159 | 5.15E-07 |
| sp|Q69YN2|C19L1_HUMAN | 61304.1088 | 1.18 | 0.169 | 3.02E-05 |
| sp|Q9GZS1|RPA49_HUMAN | 47668.56178 | 0.88 | 0.263 | 0.009597 |
| sp|Q9UP95|S12A4_HUMAN | 121694.1962 | 0.91 | 0.168 | 0.005606 |
| sp|P55899|FCGRN_HUMAN | 40042.39332 | 1.1 | 0.116 | 0.0003667 |
| sp|Q9NYL2|M3K20_HUMAN | 91706.85417 | 0.79 | 0.14 | 6.48E-07 |
| sp|Q96F86|EDC3_HUMAN | 56765.63348 | 0.86 | 0.173 | 0.0003802 |
| sp|Q14728|MFS10_HUMAN | 48689.00115 | 0.49 | 0.101 | 3.41E-15 |
| sp|Q9NVH2|INT7_HUMAN | 108002.5277 | 0.39 | 0.056 | 2.20E-16 |
| sp|Q9NRA8|4ET_HUMAN | 108458.3755 | 1.15 | 0.286 | 0.04989 |
| sp|P62256|UBE2H_HUMAN | 20681.08136 | 1.35 | 0.369 | 8.93E-05 |
| sp|O14966|RAB7L_HUMAN | 23407.4983 | 0.78 | 0.152 | 2.03E-07 |
| sp|Q8N6S5|AR6P6_HUMAN | 24927.51686 | 0.85 | 0.193 | 0.0004991 |
| sp|Q03169|TNAP2_HUMAN | 72997.25618 | 0.88 | 0.254 | 0.005129 |
| sp|Q9NPI6|DCP1A_HUMAN | 63392.27911 | 1.05 | 0.091 | 0.009872 |
| sp|Q9H4A3|WNK1_HUMAN | 251534.3132 | 1.14 | 0.106 | 9.42E-07 |
| sp|O95793|STAU1_HUMAN | 63409.85635 | 1.31 | 0.319 | 4.62E-05 |
| sp|Q8NFH4|NUP37_HUMAN | 37122.2001 | 1.33 | 0.11 | 6.24E-15 |
| sp|Q9NPD8|UBE2T_HUMAN | 22602.648 | 1.31 | 0.313 | 2.00E-05 |
| sp|Q6ISB3|GRHL2_HUMAN | 71384.51422 | 0.86 | 0.292 | 0.007653 |
| sp|Q8NHL6|LIRB1_HUMAN | 71384.5857 | 1.18 | 0.234 | 0.002708 |
| sp|Q96C24|SYTL4_HUMAN | 76585.06334 | 0.85 | 0.231 | 0.0009732 |
| sp|P49368|TCPG_HUMAN | 61047.52182 | 1.07 | 0.141 | 0.02767 |
| sp|Q9BV57|MTND_HUMAN | 21523.65572 | 1.06 | 0.089 | 0.00376 |
| sp|Q86UX7|URP2_HUMAN | 76457.2134 | 0.76 | 0.169 | 8.00E-07 |
| sp|O95757|HS74L_HUMAN | 95460.89648 | 0.76 | 0.091 | 1.17E-11 |
| sp|Q9BT67|NFIP1_HUMAN | 24979.38411 | 0.81 | 0.156 | 1.63E-05 |
| sp|P02671|FIBA_HUMAN | 95637.68349 | 1.52 | 1.056 | 0.0343 |
| sp|Q9UM19|HPCL4_HUMAN | 22341.03974 | 1.32 | 0.309 | 0.0001395 |
| sp|Q9NV31|IMP3_HUMAN | 21932.60942 | 0.87 | 0.14 | 7.73E-05 |
| sp|Q96II8|LRCH3_HUMAN | 87095.3618 | 1.23 | 0.286 | 0.0003993 |
| sp|P32455|GBP1_HUMAN | 68382.89754 | 1.37 | 0.986 | 0.2415 |
| sp|P83916|CBX1_HUMAN | 21500.60466 | 1.43 | 0.257 | 9.93E-10 |
| sp|Q08209|PP2BA_HUMAN | 59316.55067 | 0.94 | 0.133 | 0.02723 |
| sp|O95299|NDUAA_HUMAN | 41049.06367 | 1.27 | 0.318 | 0.0005701 |
| sp|Q96PP4|TSG13_HUMAN | 31796.72378 | 2.01 | 0.888 | 4.02E-07 |
| sp|Q96RQ3|MCCA_HUMAN | 80917.11283 | 1.02 | 0.219 | 0.9748 |
| sp|Q8IY81|SPB1_HUMAN | 96880.33393 | 1.32 | 0.263 | 3.57E-07 |
| sp|P60033|CD81_HUMAN | 26458.3623 | 0.43 | 0.158 | 1.10E-11 |
| sp|P82673|RT35_HUMAN | 37088.18424 | 0.98 | 0.07 | 0.1184 |
| sp|Q9NUL3|STAU2_HUMAN | 62779.36722 | 1.16 | 0.179 | 0.0005933 |
| sp|Q9NXR1|NDE1_HUMAN | 37850.82667 | 1.3 | 0.082 | 2.20E-16 |
| sp|Q8IUK5|PLDX1_HUMAN | 56220.17764 | 0.7 | 0.087 | 3.59E-13 |
| sp|P16870|CBPE_HUMAN | 53498.2662 | 0.93 | 0.472 | 0.06982 |
| sp|O43157|PLXB1_HUMAN | 235382.8812 | 0.98 | 0.14 | 0.246 |
| sp|A6NI73|LIRA5_HUMAN | 33001.57331 | 0.88 | 0.25 | 0.009198 |
| sp|Q6UWY5|OLFL1_HUMAN | 46359.52404 | 2.01 | 1.323 | 8.84E-05 |
| sp|Q8NBQ5|DHB11_HUMAN | 33238.84922 | 0.52 | 0.105 | 1.05E-13 |
| sp|P27797|CALR_HUMAN | 48264.87845 | 1.45 | 0.173 | 6.31E-14 |
| sp|Q9P289|STK26_HUMAN | 46766.70762 | 1.27 | 0.218 | 1.60E-06 |
| sp|Q9Y3V2|RWDD3_HUMAN | 30790.99879 | 0.56 | 0.313 | 8.38E-08 |
| sp|Q01804|OTUD4_HUMAN | 124804.9224 | 1.26 | 0.116 | 3.26E-12 |
| sp|P25398|RS12_HUMAN | 14886.62125 | 1.02 | 0.124 | 0.5717 |
| sp|Q92896|GSLG1_HUMAN | 138323.0498 | 0.78 | 0.114 | 4.78E-09 |
| sp|P55160|NCKPL_HUMAN | 129535.5096 | 0.71 | 0.15 | 5.24E-09 |
| sp|Q86VR2|RETR3_HUMAN | 51744.87823 | 0.93 | 0.079 | 0.0001688 |
| sp|Q9Y6X8|ZHX2_HUMAN | 92916.01035 | 1.34 | 0.277 | 2.50E-06 |
| sp|Q8IXL7|MSRB3_HUMAN | 21298.17006 | 0.95 | 0.404 | 0.1291 |
| sp|P46778|RL21_HUMAN | 18592.07179 | 1.26 | 0.178 | 6.02E-08 |
| sp|Q13201|MMRN1_HUMAN | 139202.8546 | 0.82 | 0.253 | 0.0007363 |
| sp|Q63HR2|TNS2_HUMAN | 154348.2617 | 0.94 | 0.354 | 0.07235 |
| sp|O60762|DPM1_HUMAN | 29654.79385 | 0.48 | 0.146 | 2.07E-12 |
| sp|Q9UJU6|DBNL_HUMAN | 48444.66278 | 1.49 | 0.175 | 1.06E-14 |
| sp|P0DN80|OR5H8_HUMAN | 35186.57742 | 0.95 | 0.795 | 0.03296 |
| sp|Q9Y2H2|SAC2_HUMAN | 129334.3473 | 0.98 | 0.134 | 0.3188 |
| sp|P32321|DCTD_HUMAN | 20440.94515 | 1.22 | 0.218 | 0.0001047 |
| sp|O95396|MOCS3_HUMAN | 50532.23326 | 0.89 | 0.119 | 0.0001187 |
| sp|Q15018|ABRX2_HUMAN | 47081.98349 | 1.02 | 0.185 | 0.961 |
| sp|P23458|JAK1_HUMAN | 134997.9274 | 0.92 | 0.115 | 0.00104 |
| sp|Q15185|TEBP_HUMAN | 18952.51522 | 1.09 | 0.232 | 0.1118 |
| sp|Q8WXF7|ATLA1_HUMAN | 63827.40137 | 1.18 | 0.343 | 0.03635 |
| sp|Q9UQ03|COR2B_HUMAN | 55470.40276 | 1.67 | 0.443 | 3.28E-09 |
| sp|Q96JB5|CK5P3_HUMAN | 57209.13206 | 1.06 | 0.166 | 0.1598 |
| sp|P39060|COIA1_HUMAN | 179370.5947 | 0.97 | 0.185 | 0.2107 |
| sp|Q9BPZ3|PAIP2_HUMAN | 15013.88916 | 1.85 | 0.498 | 4.17E-10 |
| sp|Q86YJ7|AN13B_HUMAN | 70599.87825 | 4.27 | 3.557 | 0.0003462 |
| sp|Q6ZXV5|TMTC3_HUMAN | 104836.2258 | 1.28 | 0.594 | 0.03512 |
| sp|Q9H081|MIS12_HUMAN | 24448.44424 | 0.65 | 0.155 | 5.41E-10 |
| sp|Q9H0V9|LMA2L_HUMAN | 39895.30573 | 0.8 | 0.167 | 7.29E-06 |
| sp|Q9Y6N9|USH1C_HUMAN | 62382.29174 | 1.02 | 0.252 | 0.9014 |
| sp|Q9P2T1|GMPR2_HUMAN | 38345.18927 | 1.12 | 0.153 | 0.001743 |
| sp|Q6NUM9|RETST_HUMAN | 67442.90777 | 0.83 | 0.179 | 3.86E-05 |
| sp|Q14202|ZMYM3_HUMAN | 156082.9054 | 0.97 | 0.08 | 0.03623 |
| sp|Q9Y279|VSIG4_HUMAN | 44511.24324 | 2.33 | 0.726 | 5.20E-12 |
| sp|P36551|HEM6_HUMAN | 50900.26228 | 1.09 | 0.13 | 0.003933 |
| sp|O15247|CLIC2_HUMAN | 28605.54172 | 1.32 | 0.164 | 7.19E-11 |
| sp|Q9Y6M7|S4A7_HUMAN | 137079.8432 | 1.42 | 0.297 | 1.95E-08 |
| sp|Q16270|IBP7_HUMAN | 30119.8177 | 1.72 | 0.405 | 3.09E-11 |
| sp|Q99759|M3K3_HUMAN | 71234.49343 | 1.39 | 0.314 | 1.66E-07 |
| sp|Q9H8H3|MET7A_HUMAN | 28795.74176 | 0.53 | 0.242 | 7.67E-08 |
| sp|O75427|LRCH4_HUMAN | 74071.28285 | 1.09 | 0.175 | 0.03704 |
| sp|Q9ULV0|MYO5B_HUMAN | 215116.8825 | 0.74 | 0.192 | 4.33E-07 |
| sp|O95294|RASL1_HUMAN | 90853.50409 | 0.45 | 0.226 | 1.03E-08 |
| sp|P62249|RS16_HUMAN | 16531.07331 | 0.99 | 0.179 | 0.4317 |
| sp|Q4V9L6|TM119_HUMAN | 29394.72454 | 1.88 | 0.999 | 4.91E-05 |
| sp|Q92598|HS105_HUMAN | 97697.99019 | 0.91 | 0.291 | 0.03099 |
| sp|Q9NPL8|TIDC1_HUMAN | 32310.83063 | 0.78 | 0.096 | 5.65E-10 |
| sp|Q9NVM9|INT13_HUMAN | 81125.66269 | 1.03 | 0.079 | 0.073 |
| sp|P24666|PPAC_HUMAN | 18468.92878 | 1.09 | 0.119 | 0.002891 |
| sp|Q9Y6K5|OAS3_HUMAN | 122842.6747 | 0.65 | 0.189 | 3.47E-08 |
| sp|P62899|RL31_HUMAN | 14435.9193 | 1.46 | 0.243 | 1.60E-10 |
| sp|A8MW92|P20L1_HUMAN | 116231.7676 | 1.06 | 0.154 | 0.1774 |
| sp|Q6ZS81|WDFY4_HUMAN | 357870.7146 | 0.97 | 0.254 | 0.2352 |
| sp|Q8WUP2|FBLI1_HUMAN | 42164.29361 | 0.98 | 0.14 | 0.3619 |
| sp|P22695|QCR2_HUMAN | 48565.93694 | 0.77 | 0.143 | 1.99E-07 |
| sp|P09327|VILI_HUMAN | 93074.90205 | 0.69 | 0.307 | 2.50E-05 |
| sp|Q13232|NDK3_HUMAN | 19212.99719 | 0.63 | 0.163 | 6.49E-09 |
| sp|Q9NZM3|ITSN2_HUMAN | 194405.3035 | 1.25 | 0.158 | 3.59E-09 |
| sp|Q15257|PTPA_HUMAN | 41079.59789 | 0.78 | 0.095 | 1.24E-10 |
| sp|P49588|SYAC_HUMAN | 107466.493 | 0.68 | 0.132 | 3.33E-10 |
| sp|P50213|IDH3A_HUMAN | 40004.2342 | 1.11 | 0.137 | 0.001118 |
| sp|Q8IZH2|XRN1_HUMAN | 195506.1265 | 0.81 | 0.17 | 3.99E-06 |
| sp|P36969|GPX4_HUMAN | 22656.16015 | 0.76 | 0.073 | 2.17E-13 |
| sp|Q15642|CIP4_HUMAN | 68520.2756 | 1.57 | 0.148 | 2.20E-16 |
| sp|Q5QJE6|TDIF2_HUMAN | 84799.00424 | 1.82 | 0.68 | 1.29E-07 |
| sp|Q9NTJ5|SAC1_HUMAN | 67419.18735 | 0.55 | 0.109 | 7.06E-14 |
| sp|Q8WU76|SCFD2_HUMAN | 75859.41601 | 0.73 | 0.073 | 2.52E-14 |
| sp|Q8NBP0|TTC13_HUMAN | 97588.71863 | 0.79 | 0.135 | 3.99E-07 |
| sp|Q8WZA9|IRGQ_HUMAN | 63173.63925 | 0.96 | 0.166 | 0.1278 |
| sp|Q8IY33|MILK2_HUMAN | 98222.30352 | 0.91 | 0.143 | 0.001911 |
| sp|Q86UE4|LYRIC_HUMAN | 63837.82262 | 1.15 | 0.215 | 0.003322 |
| sp|Q9BV38|WDR18_HUMAN | 48041.25375 | 0.82 | 0.099 | 7.10E-09 |
| sp|Q9Y2Q9|RT28_HUMAN | 20982.85728 | 1.09 | 0.126 | 0.00207 |
| sp|Q02880|TOP2B_HUMAN | 184103.8053 | 0.71 | 0.089 | 8.75E-13 |
| sp|Q99856|ARI3A_HUMAN | 62889.01311 | 0.91 | 0.36 | 0.0448 |
| sp|Q08AM6|VAC14_HUMAN | 88925.08529 | 0.77 | 0.096 | 1.95E-10 |
| sp|Q2VPK5|CTU2_HUMAN | 57193.45167 | 0.81 | 0.094 | 1.06E-09 |
| sp|Q8NE86|MCU_HUMAN | 40108.86805 | 0.68 | 0.145 | 1.90E-09 |
| sp|Q99424|ACOX2_HUMAN | 77558.27276 | 0.69 | 0.152 | 5.50E-09 |
| sp|Q9P0P0|RN181_HUMAN | 18278.61913 | 1.12 | 0.363 | 0.5978 |
| sp|P29323|EPHB2_HUMAN | 119109.5327 | 0.91 | 0.28 | 0.0614 |
| sp|P26440|IVD_HUMAN | 47115.93055 | 0.99 | 0.212 | 0.4743 |
| sp|O94769|ECM2_HUMAN | 80633.53161 | 3.52 | 2.515 | 1.59E-09 |
| sp|O95865|DDAH2_HUMAN | 29892.5524 | 1.53 | 0.36 | 1.14E-09 |
| sp|Q9UG01|IF172_HUMAN | 199200.5527 | 1.32 | 0.423 | 0.0008221 |
| sp|Q8IYS2|K2013_HUMAN | 69665.69373 | 0.66 | 0.096 | 6.51E-13 |
| sp|Q15942|ZYX_HUMAN | 62417.60711 | 1.35 | 0.261 | 1.94E-07 |
| sp|Q14997|PSME4_HUMAN | 213518.4791 | 0.62 | 0.114 | 4.19E-12 |
| sp|O95169|NDUB8_HUMAN | 21847.40682 | 0.88 | 0.192 | 0.002264 |
| sp|Q9NW68|BSDC1_HUMAN | 47401.01921 | 1.52 | 0.189 | 1.37E-14 |
| sp|Q9H2I8|LRMDA_HUMAN | 22706.7181 | 0.92 | 0.25 | 0.03436 |
| sp|Q8IZD4|DCP1B_HUMAN | 68061.96128 | 0.97 | 0.122 | 0.1533 |
| sp|Q9BZ29|DOCK9_HUMAN | 238500.8455 | 1.08 | 0.237 | 0.2008 |
| sp|Q04828|AK1C1_HUMAN | 37203.17965 | 0.92 | 0.362 | 0.06488 |
| sp|P62195|PRS8_HUMAN | 45750.10692 | 1.37 | 0.086 | 2.20E-16 |
| sp|P29401|TKT_HUMAN | 68500.97173 | 0.78 | 0.083 | 8.95E-12 |
| sp|Q96AY4|TTC28_HUMAN | 272635.3178 | 0.84 | 0.138 | 8.12E-06 |
| sp|P05164|PERM_HUMAN | 84766.19309 | 0.65 | 0.319 | 1.69E-05 |
| sp|O60704|TPST2_HUMAN | 42265.99726 | 1 | 0.235 | 0.6208 |
| sp|Q86YV6|MYLK4_HUMAN | 44860.74096 | 0.73 | 0.136 | 4.34E-09 |
| sp|A0A0C4DH29|HV103_HUMAN | 13095.44215 | 1.31 | 0.525 | 0.01411 |
| sp|Q96AZ6|ISG20_HUMAN | 20503.66712 | 1.12 | 0.255 | 0.04289 |
| sp|P01111|RASN_HUMAN | 21482.61132 | 1.02 | 0.117 | 0.5761 |
| sp|P17026|ZNF22_HUMAN | 26565.19192 | 1.05 | 0.47 | 0.579 |
| sp|Q9BQ69|MACD1_HUMAN | 35977.899 | 0.82 | 0.147 | 3.65E-06 |
| sp|Q08380|LG3BP_HUMAN | 66183.61861 | 0.83 | 0.274 | 0.002198 |
| sp|Q9UK22|FBX2_HUMAN | 33687.97654 | 1.14 | 0.369 | 0.3624 |
| sp|P51665|PSMD7_HUMAN | 37041.50837 | 0.82 | 0.186 | 5.50E-05 |
| sp|P06213|INSR_HUMAN | 158894.3951 | 0.97 | 0.082 | 0.07832 |
| sp|P40937|RFC5_HUMAN | 38739.28226 | 0.74 | 0.278 | 5.99E-05 |
| sp|O60496|DOK2_HUMAN | 45731.85935 | 1.52 | 0.322 | 5.87E-09 |
| sp|P55809|SCOT1_HUMAN | 56560.09076 | 1.48 | 0.346 | 1.30E-07 |
| sp|Q8N8A6|DDX51_HUMAN | 72964.79301 | 0.99 | 0.172 | 0.4503 |
| sp|P61927|RL37_HUMAN | 11280.93969 | 1.36 | 0.361 | 0.0001384 |
| sp|Q8N4T8|CBR4_HUMAN | 25438.45939 | 0.89 | 0.339 | 0.01966 |
| sp|Q96D71|REPS1_HUMAN | 86762.3129 | 1.17 | 0.177 | 4.03E-05 |
| sp|Q12912|LRMP_HUMAN | 62749.34488 | 1.27 | 0.336 | 0.0001764 |
| sp|P01909|DQA1_HUMAN | 28054.32235 | 1.13 | 0.589 | 0.99 |
| sp|P32121|ARRB2_HUMAN | 46571.99246 | 0.94 | 0.121 | 0.009849 |
| sp|A0A0B4J241|TVAM1_HUMAN | 12668.36284 | 1.26 | 0.609 | 0.3176 |
| sp|Q9HC21|TPC_HUMAN | 35869.45719 | 0.57 | 0.093 | 5.23E-15 |
| sp|P51397|DAP1_HUMAN | 11139.6489 | 1.48 | 0.522 | 0.0001782 |
| sp|Q13555|KCC2G_HUMAN | 63290.97497 | 0.87 | 0.237 | 0.006609 |
| sp|Q8TDN6|BRX1_HUMAN | 41642.04225 | 0.96 | 0.188 | 0.1408 |
| sp|P61160|ARP2_HUMAN | 44999.33034 | 0.67 | 0.097 | 1.95E-13 |
| sp|O94817|ATG12_HUMAN | 15199.78996 | 0.81 | 0.209 | 0.0002224 |
| sp|Q96SK2|TM209_HUMAN | 63262.61104 | 0.72 | 0.146 | 3.03E-09 |
| sp|Q16204|CCDC6_HUMAN | 53410.88233 | 1.35 | 0.14 | 2.37E-13 |
| sp|Q93100|KPBB_HUMAN | 125927.7748 | 0.69 | 0.072 | 1.82E-15 |
| sp|Q8N3U4|STAG2_HUMAN | 142414.8684 | 0.57 | 0.059 | 2.20E-16 |
| sp|Q7Z4H3|HDDC2_HUMAN | 23528.70363 | 1.54 | 0.181 | 1.38E-15 |
| sp|Q14BN4|SLMAP_HUMAN | 95976.918 | 0.62 | 0.221 | 1.57E-07 |
| sp|Q9UJ78|ZMYM5_HUMAN | 76120.55072 | 0.96 | 0.191 | 0.1749 |
| sp|Q9Y2X0|MED16_HUMAN | 98423.26418 | 0.69 | 0.06 | 2.20E-16 |
| sp|Q96KQ4|ASPP1_HUMAN | 120043.8 | 0.68 | 0.089 | 2.07E-13 |
| sp|P10124|SRGN_HUMAN | 17793.69094 | 0.85 | 0.145 | 4.87E-05 |
| sp|Q5T013|HYI_HUMAN | 30482.59588 | 1.08 | 0.17 | 0.03417 |
| sp|Q9Y5R8|TPPC1_HUMAN | 16916.63697 | 0.8 | 0.077 | 2.61E-11 |
| sp|P02747|C1QC_HUMAN | 25967.21337 | 0.94 | 0.382 | 0.09039 |
| sp|Q76M96|CCD80_HUMAN | 108487.0664 | 1.67 | 0.325 | 6.49E-12 |
| sp|Q9BQE5|APOL2_HUMAN | 37108.40441 | 1.27 | 0.333 | 0.0003167 |
| sp|P62316|SMD2_HUMAN | 13614.25915 | 1.33 | 0.205 | 6.39E-09 |
| sp|O75177|CREST_HUMAN | 43059.60918 | 1.27 | 0.377 | 0.006657 |
| sp|Q9BXJ9|NAA15_HUMAN | 102444.172 | 0.65 | 0.103 | 1.92E-13 |
| sp|Q5TAQ9|DCAF8_HUMAN | 67477.35961 | 1.22 | 0.113 | 1.76E-10 |
| sp|A6NHL2|TBAL3_HUMAN | 50657.02019 | 0.24 | 0.052 | 2.20E-16 |
| sp|Q92538|GBF1_HUMAN | 208349.3736 | 0.63 | 0.131 | 2.82E-11 |
| sp|P20702|ITAX_HUMAN | 128928.0615 | 0.66 | 0.129 | 6.72E-11 |
| sp|Q969G6|RIFK_HUMAN | 17650.9556 | 1.06 | 0.201 | 0.3042 |
| sp|Q9BTM1|H2AJ_HUMAN | 13992.91585 | 2.16 | 1.552 | 0.0001509 |
| sp|Q9P2N5|RBM27_HUMAN | 119082.9115 | 1.39 | 0.311 | 6.85E-07 |
| sp|Q8NFA0|UBP32_HUMAN | 183803.2224 | 0.99 | 0.37 | 0.2908 |
| sp|Q9BUP0|EFHD1_HUMAN | 27006.85585 | 1.97 | 0.721 | 4.99E-10 |
| sp|Q8IYU8|MICU2_HUMAN | 49901.45705 | 0.67 | 0.199 | 9.30E-08 |
| sp|P78362|SRPK2_HUMAN | 78201.42196 | 0.72 | 0.097 | 3.64E-12 |
| sp|P51991|ROA3_HUMAN | 39780.65867 | 1.36 | 0.222 | 3.81E-09 |
| sp|Q5T619|ZN648_HUMAN | 63765.61688 | 1.77 | 0.829 | 1.71E-05 |
| sp|Q8IZV5|RDH10_HUMAN | 38728.66609 | 0.36 | 0.152 | 1.11E-12 |
| sp|Q92796|DLG3_HUMAN | 90809.82978 | 1.13 | 0.31 | 0.1092 |
| sp|Q14657|LAGE3_HUMAN | 14890.59847 | 1.16 | 0.223 | 0.001074 |
| sp|O15014|ZN609_HUMAN | 152334.134 | 1.68 | 0.415 | 8.40E-10 |
| sp|P08575|PTPRC_HUMAN | 148858.4466 | 0.75 | 0.212 | 3.84E-06 |
| sp|P17661|DESM_HUMAN | 53542.16078 | 0.52 | 0.245 | 4.41E-07 |
| sp|O95685|PPR3D_HUMAN | 33261.66112 | 0.95 | 0.09 | 0.01245 |
| sp|Q13614|MTMR2_HUMAN | 73887.23427 | 0.65 | 0.089 | 3.43E-14 |
| sp|O00584|RNT2_HUMAN | 30071.04665 | 1.1 | 0.281 | 0.1715 |
| sp|Q15306|IRF4_HUMAN | 52462.68943 | 1.13 | 0.211 | 0.01604 |
| sp|P63165|SUMO1_HUMAN | 11588.71641 | 1.59 | 0.466 | 2.95E-08 |
| sp|Q96PK6|RBM14_HUMAN | 69601.95946 | 1.22 | 0.177 | 5.14E-07 |
| sp|Q9BWH2|FUND2_HUMAN | 20701.71988 | 0.89 | 0.273 | 0.01077 |
| sp|Q6UXV4|MIC27_HUMAN | 29293.30658 | 0.64 | 0.103 | 5.84E-13 |
| sp|P34059|GALNS_HUMAN | 58427.24222 | 1.05 | 0.284 | 0.8377 |
| sp|Q6P4A7|SFXN4_HUMAN | 38240.91338 | 0.87 | 0.115 | 1.66E-05 |
| sp|Q13395|TARB1_HUMAN | 183821.6518 | 0.91 | 0.192 | 0.006895 |
| sp|Q9UIC8|LCMT1_HUMAN | 39134.27307 | 1.03 | 0.177 | 0.5948 |
| sp|Q9UKD2|MRT4_HUMAN | 27638.94433 | 0.58 | 0.235 | 7.87E-08 |
| sp|P18615|NELFE_HUMAN | 43309.77171 | 1.25 | 0.305 | 0.0002058 |
| sp|P60468|SC61B_HUMAN | 10007.10838 | 0.71 | 0.247 | 7.93E-06 |
| sp|Q9Y5A7|NUB1_HUMAN | 71217.39279 | 1.11 | 0.251 | 0.08244 |
| sp|P46976|GLYG_HUMAN | 39682.76404 | 1.01 | 0.156 | 0.9859 |
| sp|O43379|WDR62_HUMAN | 167998.2023 | 1.28 | 0.346 | 0.001203 |
| sp|Q9P1Z2|CACO1_HUMAN | 77726.91125 | 1.19 | 0.153 | 3.65E-07 |
| sp|Q15717|ELAV1_HUMAN | 36222.20388 | 1.5 | 0.189 | 1.27E-14 |
| sp|P0C870|JMJD7_HUMAN | 36290.05489 | 1.05 | 0.3 | 0.9212 |
| sp|O60942|MCE1_HUMAN | 69406.9016 | 1.3 | 0.405 | 0.003176 |
| sp|Q9UKF6|CPSF3_HUMAN | 78102.39577 | 0.73 | 0.082 | 2.87E-13 |
| sp|P05161|ISG15_HUMAN | 17915.354 | 0.95 | 0.236 | 0.1174 |
| sp|Q6NZY4|ZCHC8_HUMAN | 79137.7419 | 1.09 | 0.108 | 0.0002619 |
| sp|Q96K76|UBP47_HUMAN | 158562.958 | 1 | 0.09 | 0.6805 |
| sp|P13747|HLAE_HUMAN | 40242.82287 | 0.96 | 0.078 | 0.01923 |
| sp|O00182|LEG9_HUMAN | 39816.97889 | 0.69 | 0.076 | 5.78E-15 |
| sp|P63279|UBC9_HUMAN | 18205.27411 | 1.25 | 0.112 | 1.89E-11 |
| sp|Q9UFF9|CNOT8_HUMAN | 33785.62869 | 0.79 | 0.204 | 4.22E-05 |
| sp|Q8N9Z2|CC71L_HUMAN | 26455.03858 | 0.64 | 0.188 | 7.14E-08 |
| sp|Q03426|KIME_HUMAN | 42976.45952 | 0.56 | 0.142 | 1.71E-11 |
| sp|Q9BTV4|TMM43_HUMAN | 44886.20962 | 0.78 | 0.114 | 2.78E-09 |
| sp|O60936|NOL3_HUMAN | 22825.7389 | 0.99 | 0.164 | 0.5317 |
| sp|O60831|PRAF2_HUMAN | 19569.63914 | 0.53 | 0.103 | 5.73E-15 |
| sp|Q3MII6|TBC25_HUMAN | 76602.71429 | 0.98 | 0.102 | 0.3019 |
| sp|Q96AA3|RFT1_HUMAN | 60848.59511 | 0.58 | 0.087 | 1.96E-15 |
| sp|P17900|SAP3_HUMAN | 21262.8939 | 1.07 | 0.481 | 0.8568 |
| sp|A0A075B6I0|LV861_HUMAN | 12902.15602 | 2.05 | 1.5 | 0.000101 |
| sp|P04424|ARLY_HUMAN | 51892.39705 | 0.54 | 0.123 | 3.56E-13 |
| sp|Q96MY1|NOL4L_HUMAN | 47509.49395 | 1.04 | 0.266 | 0.8917 |
| sp|Q9UKI9|PO2F3_HUMAN | 47726.73619 | 1.39 | 0.246 | 3.04E-09 |
| sp|P62341|SELT_HUMAN | 22463.39057 | 0.58 | 0.093 | 5.63E-15 |
| sp|Q86UB9|TM135_HUMAN | 53036.37961 | 0.77 | 0.123 | 1.00E-08 |
| sp|P52272|HNRPM_HUMAN | 77731.41053 | 1.46 | 0.276 | 3.87E-09 |
| sp|P51993|FUT6_HUMAN | 42270.92806 | 0.47 | 0.091 | 2.20E-16 |
| sp|P49754|VPS41_HUMAN | 99682.52029 | 0.89 | 0.18 | 0.004255 |
| sp|Q9H3M7|TXNIP_HUMAN | 44242.34032 | 1.12 | 0.192 | 0.01481 |
| sp|Q63HM1|KFA_HUMAN | 34180.14165 | 0.76 | 0.319 | 0.0003134 |
| sp|Q5T0D9|TPRGL_HUMAN | 30345.73397 | 1.33 | 0.307 | 6.61E-06 |
| sp|P54821|PRRX1_HUMAN | 27318.7718 | 1.44 | 0.231 | 1.96E-10 |
| sp|Q9BPY3|F118B_HUMAN | 39741.35502 | 0.99 | 0.251 | 0.414 |
| sp|Q99969|RARR2_HUMAN | 18929.87367 | 1.39 | 0.188 | 3.94E-11 |
| sp|P61970|NTF2_HUMAN | 14622.10225 | 1.25 | 0.175 | 4.55E-08 |
| sp|Q99932|SPAG8_HUMAN | 51772.73079 | 1.68 | 0.66 | 7.34E-06 |
| sp|O15540|FABP7_HUMAN | 14975.50136 | 1.64 | 0.612 | 2.97E-05 |
| sp|Q969L2|MAL2_HUMAN | 19322.76605 | 0.58 | 0.155 | 1.16E-10 |
| sp|P07311|ACYP1_HUMAN | 11235.85127 | 1.12 | 0.194 | 0.005062 |
| sp|Q9BZR9|TRIM8_HUMAN | 62970.82932 | 1.14 | 0.096 | 6.44E-08 |
| sp|O60220|TIM8A_HUMAN | 11201.37119 | 1 | 0.183 | 0.7574 |
| sp|Q13263|TIF1B_HUMAN | 90243.07382 | 1.1 | 0.15 | 0.005829 |
| sp|Q99643|C560_HUMAN | 18807.8628 | 0.38 | 0.106 | 1.14E-15 |
| sp|P51116|FXR2_HUMAN | 74502.13127 | 1.15 | 0.109 | 2.67E-07 |
| sp|Q9HAT2|SIAE_HUMAN | 58943.3901 | 0.96 | 0.233 | 0.1406 |
| sp|Q6ZN84|CCD81_HUMAN | 76758.54085 | 0.63 | 0.174 | 5.02E-10 |
| sp|O95295|SNAPN_HUMAN | 14903.90372 | 2.01 | 0.382 | 1.25E-15 |
| sp|Q86TX2|ACOT1_HUMAN | 46629.19552 | 0.93 | 0.087 | 0.0007168 |
| sp|Q9BY67|CADM1_HUMAN | 48916.60895 | 1.16 | 0.528 | 0.6392 |
| sp|P20132|SDHL_HUMAN | 34927.35223 | 0.46 | 0.138 | 2.41E-13 |
| sp|P35221|CTNA1_HUMAN | 100674.7722 | 0.63 | 0.184 | 2.91E-08 |
| sp|P50897|PPT1_HUMAN | 34609.41223 | 1.29 | 0.132 | 1.68E-11 |
| sp|Q9UKV3|ACINU_HUMAN | 152151.9304 | 1.5 | 0.321 | 2.01E-09 |
| sp|Q96EB6|SIR1_HUMAN | 82695.35793 | 1 | 0.258 | 0.5885 |
| sp|Q15181|IPYR_HUMAN | 33077.31657 | 1.2 | 0.327 | 0.008107 |
| sp|Q9NR97|TLR8_HUMAN | 120874.8341 | 0.75 | 0.33 | 0.0003095 |
| sp|Q5JTD0|TJAP1_HUMAN | 62107.51975 | 1.01 | 0.114 | 0.9123 |
| sp|Q9UEU0|VTI1B_HUMAN | 26653.88209 | 1.01 | 0.105 | 0.7395 |
| sp|Q13308|PTK7_HUMAN | 119781.4005 | 1.21 | 0.266 | 0.0006683 |
| sp|P35869|AHR_HUMAN | 97094.61804 | 0.88 | 0.151 | 0.0004576 |
| sp|Q5XPI4|RN123_HUMAN | 149941.7375 | 0.67 | 0.083 | 2.49E-14 |
| sp|Q643R3|LPCT4_HUMAN | 57621.44918 | 0.38 | 0.197 | 7.12E-11 |
| sp|Q9H1C7|CYTM1_HUMAN | 10947.88319 | 0.59 | 0.257 | 1.99E-06 |
| sp|Q96SB3|NEB2_HUMAN | 89290.75267 | 1.52 | 0.191 | 3.33E-14 |
| sp|P41222|PTGDS_HUMAN | 21225.41487 | 1.24 | 0.475 | 0.03463 |
| sp|Q9UMX0|UBQL1_HUMAN | 62461.42567 | 1.21 | 0.195 | 9.95E-06 |
| sp|P41240|CSK_HUMAN | 51223.93592 | 0.94 | 0.221 | 0.08068 |
| sp|Q9Y251|HPSE_HUMAN | 61377.22007 | 1.18 | 0.551 | 0.4986 |
| sp|O95167|NDUA3_HUMAN | 9254.842501 | 0.76 | 0.149 | 1.28E-07 |
| sp|Q8IZ73|RUSD2_HUMAN | 61825.38333 | 1.1 | 0.113 | 0.0007441 |
| sp|Q9H115|SNAB_HUMAN | 33859.61031 | 0.7 | 0.271 | 2.29E-05 |
| sp|Q8WVM7|STAG1_HUMAN | 145287.0294 | 0.72 | 0.108 | 1.80E-11 |
| sp|Q9H0F7|ARL6_HUMAN | 21237.20378 | 0.9 | 0.377 | 0.02788 |
| sp|Q5VTQ0|TT39B_HUMAN | 77857.80738 | 0.33 | 0.097 | 2.20E-16 |
| sp|A6NGE7|URAD_HUMAN | 19270.97448 | 0.96 | 0.446 | 0.1242 |
| sp|Q13564|ULA1_HUMAN | 60646.72681 | 0.94 | 0.126 | 0.01073 |
| sp|Q96BX8|MOB3A_HUMAN | 25657.93954 | 0.47 | 0.115 | 7.30E-14 |
| sp|Q9H9H4|VP37B_HUMAN | 31326.50071 | 1.13 | 0.214 | 0.01373 |
| sp|O75882|ATRN_HUMAN | 163432.3408 | 0.53 | 0.098 | 1.65E-15 |
| sp|Q8N859|ZN713_HUMAN | 51091.49287 | 0.94 | 0.197 | 0.0694 |
| sp|Q7Z4L5|TT21B_HUMAN | 152361.7002 | 0.62 | 0.121 | 3.30E-12 |
| sp|Q13188|STK3_HUMAN | 56532.07759 | 1.53 | 0.285 | 4.08E-11 |
| sp|Q8N9N8|EIF1A_HUMAN | 19080.50182 | 0.79 | 0.423 | 0.002906 |
| sp|O43395|PRPF3_HUMAN | 77633.98848 | 1.27 | 0.205 | 3.75E-07 |
| sp|O95279|KCNK5_HUMAN | 55362.26927 | 0.93 | 0.375 | 0.06714 |
| sp|P13686|PPA5_HUMAN | 36728.76174 | 1.3 | 0.308 | 2.07E-05 |
| sp|Q8WW59|SPRY4_HUMAN | 23210.03576 | 1.11 | 0.153 | 0.002297 |
| sp|Q96IQ7|VSIG2_HUMAN | 34878.6715 | 0.79 | 0.31 | 0.003436 |
| sp|O14787|TNPO2_HUMAN | 102843.7991 | 0.71 | 0.09 | 1.85E-12 |
| sp|Q9NQ79|CRAC1_HUMAN | 72156.0987 | 1.16 | 0.274 | 0.01154 |
| sp|Q6QNY0|BL1S3_HUMAN | 21338.92573 | 1.09 | 0.12 | 0.000761 |
| sp|Q13641|TPBG_HUMAN | 46554.95422 | 0.73 | 0.231 | 2.81E-06 |
| sp|Q9NXV2|KCTD5_HUMAN | 26457.19054 | 1.19 | 0.259 | 0.001136 |
| sp|Q9H967|WDR76_HUMAN | 70448.4222 | 0.92 | 0.124 | 0.001557 |
| sp|Q9UHY1|NRBP_HUMAN | 60359.33328 | 1.09 | 0.102 | 0.0004955 |
| sp|P14902|I23O1_HUMAN | 45735.35229 | 1.71 | 1.248 | 0.004415 |
| sp|P51784|UBP11_HUMAN | 110926.6925 | 1.08 | 0.214 | 0.1554 |
| sp|Q70IA8|MOB3C_HUMAN | 25930.1437 | 0.73 | 0.2 | 5.72E-07 |
| sp|P43487|RANG_HUMAN | 23448.62422 | 1.68 | 0.483 | 1.29E-08 |
| sp|P35222|CTNB1_HUMAN | 86051.43796 | 0.6 | 0.174 | 1.53E-09 |
| sp|Q9ULE4|F184B_HUMAN | 121863.6152 | 0.65 | 0.209 | 1.64E-07 |
| sp|Q13610|PWP1_HUMAN | 56345.27944 | 1.03 | 0.153 | 0.4201 |
| sp|C4AMC7|WASH3_HUMAN | 50002.7816 | 1.24 | 0.177 | 7.59E-08 |
| sp|Q99497|PARK7_HUMAN | 20031.54208 | 1.38 | 0.185 | 7.75E-12 |
| sp|Q9UGJ1|GCP4_HUMAN | 76536.51563 | 0.83 | 0.098 | 2.40E-08 |
| sp|Q6P1N0|C2D1A_HUMAN | 104379.3885 | 1.04 | 0.164 | 0.4385 |
| sp|P10636|TAU_HUMAN | 79089.84363 | 2 | 0.566 | 9.85E-11 |
| sp|O75175|CNOT3_HUMAN | 82031.73015 | 0.98 | 0.116 | 0.3234 |
| sp|Q5BJF2|SGMR2_HUMAN | 20987.2236 | 0.72 | 0.367 | 0.0001815 |
| sp|Q9BYM8|HOIL1_HUMAN | 59340.54823 | 1.17 | 0.197 | 0.0002993 |
| sp|Q86XP3|DDX42_HUMAN | 103178.9664 | 1.11 | 0.084 | 6.05E-07 |
| sp|Q9UGU5|HMGX4_HUMAN | 65995.26862 | 0.97 | 0.211 | 0.2021 |
| sp|P01717|LV325_HUMAN | 12156.89628 | 1.13 | 0.236 | 0.01845 |
| sp|Q9BZI7|REN3B_HUMAN | 57823.13673 | 1.18 | 0.162 | 9.26E-06 |
| sp|Q5TEU4|NDUF5_HUMAN | 39103.67698 | 1.35 | 0.39 | 0.0003782 |
| sp|Q8TEX9|IPO4_HUMAN | 120161.1202 | 0.69 | 0.104 | 7.62E-12 |
| sp|Q05048|CSTF1_HUMAN | 49107.01284 | 1.15 | 0.154 | 4.13E-05 |
| sp|Q9Y2L9|LRCH1_HUMAN | 81832.41601 | 0.8 | 0.189 | 1.39E-05 |
| sp|P23025|XPA_HUMAN | 31728.89901 | 1.82 | 0.761 | 5.24E-06 |
| sp|Q13103|SPP24_HUMAN | 24588.85543 | 0.71 | 0.168 | 1.18E-08 |
| sp|Q6NSW5|FA45B_HUMAN | 40812.0477 | 0.87 | 0.133 | 5.22E-05 |
| sp|Q9H0B8|CRLD2_HUMAN | 57347.6178 | 1.13 | 0.335 | 0.1878 |
| sp|Q00610|CLH1_HUMAN | 193242.179 | 0.55 | 0.079 | 2.20E-16 |
| sp|Q15554|TERF2_HUMAN | 59709.63347 | 0.94 | 0.111 | 0.005716 |
| sp|Q9ULJ8|NEB1_HUMAN | 123533.8569 | 0.8 | 0.213 | 5.35E-05 |
| sp|P08047|SP1_HUMAN | 81252.82897 | 1.87 | 0.324 | 4.42E-15 |
| sp|Q16854|DGUOK_HUMAN | 32131.58832 | 0.69 | 0.126 | 1.48E-10 |
| sp|P15559|NQO1_HUMAN | 30887.01915 | 0.78 | 0.38 | 0.002485 |
| sp|P51608|MECP2_HUMAN | 52561.70853 | 1.71 | 0.406 | 1.79E-10 |
| sp|Q15904|VAS1_HUMAN | 52145.65064 | 0.82 | 0.152 | 3.58E-06 |
| sp|Q5ZPR3|CD276_HUMAN | 57922.74496 | 0.86 | 0.125 | 1.24E-05 |
| sp|Q9ULX3|NOB1_HUMAN | 47026.91943 | 1.31 | 0.339 | 6.69E-05 |
| sp|Q96PZ0|PUS7_HUMAN | 75312.27321 | 0.98 | 0.141 | 0.2676 |
| sp|Q7Z2W4|ZCCHV_HUMAN | 103116.6581 | 0.83 | 0.081 | 3.61E-10 |
| sp|Q9NZC3|GDE1_HUMAN | 38075.15717 | 0.84 | 0.138 | 5.54E-06 |
| sp|Q9NVJ2|ARL8B_HUMAN | 21735.2171 | 0.53 | 0.047 | 2.20E-16 |
| sp|P61812|TGFB2_HUMAN | 48554.49019 | 1.27 | 0.591 | 0.07601 |
| sp|O94822|LTN1_HUMAN | 203085.3694 | 0.75 | 0.117 | 1.02E-09 |
| sp|Q9GZN1|ARP6_HUMAN | 46218.92298 | 1.41 | 0.331 | 3.28E-07 |
| sp|O94804|STK10_HUMAN | 112731.3539 | 1.25 | 0.332 | 0.001119 |
| sp|Q9Y3R5|DOP2_HUMAN | 260330.3522 | 0.71 | 0.178 | 3.02E-07 |
| sp|P04217|A1BG_HUMAN | 54771.74356 | 1.19 | 0.202 | 8.54E-05 |
| sp|Q14139|UBE4A_HUMAN | 123547.3685 | 0.64 | 0.083 | 1.72E-15 |
| sp|P06753|TPM3_HUMAN | 32968.78844 | 1.51 | 0.107 | 2.20E-16 |
| sp|Q9BZR6|RTN4R_HUMAN | 51570.4113 | 1.29 | 0.778 | 0.3259 |
| sp|Q5J8M3|EMC4_HUMAN | 20226.21591 | 0.71 | 0.275 | 2.18E-05 |
| sp|Q9BV44|THUM3_HUMAN | 57747.12501 | 0.85 | 0.12 | 3.20E-06 |
| sp|P05026|AT1B1_HUMAN | 35420.00605 | 0.44 | 0.199 | 2.45E-11 |
| sp|P48059|LIMS1_HUMAN | 39089.54044 | 0.99 | 0.245 | 0.4279 |
| sp|P26368|U2AF2_HUMAN | 53791.30933 | 1.29 | 0.174 | 2.19E-09 |
| sp|Q9H8H0|NOL11_HUMAN | 82023.27053 | 0.82 | 0.188 | 9.00E-05 |
| sp|Q9BY32|ITPA_HUMAN | 21813.03448 | 1.47 | 0.384 | 2.86E-07 |
| sp|Q96CX2|KCD12_HUMAN | 35945.78814 | 1.31 | 0.315 | 9.48E-06 |
| sp|Q9UQN3|CHM2B_HUMAN | 23930.22293 | 2.03 | 0.783 | 2.47E-08 |
| sp|Q12830|BPTF_HUMAN | 340772.6507 | 0.93 | 0.097 | 0.001607 |
| sp|Q12979|ABR_HUMAN | 98088.37377 | 0.78 | 0.137 | 3.47E-08 |
| sp|Q86UU0|BCL9L_HUMAN | 157408.5969 | 1.12 | 0.175 | 0.006935 |
| sp|Q86X76|NIT1_HUMAN | 36710.47807 | 0.98 | 0.077 | 0.156 |
| sp|Q7L8A9|VASH1_HUMAN | 41027.12392 | 1.11 | 0.273 | 0.1124 |
| sp|P49768|PSN1_HUMAN | 52901.21865 | 0.91 | 0.174 | 0.007249 |
| sp|P62979|RS27A_HUMAN | 18277.60981 | 1.25 | 0.133 | 1.69E-10 |
| sp|Q9NRD1|FBX6_HUMAN | 34292.01634 | 0.86 | 0.745 | 0.008185 |
| sp|Q66LE6|2ABD_HUMAN | 52561.79436 | 1.18 | 0.178 | 1.01E-05 |
| sp|O14579|COPE_HUMAN | 34670.41578 | 1.15 | 0.108 | 8.36E-08 |
| sp|Q12874|SF3A3_HUMAN | 59136.37493 | 1.11 | 0.189 | 0.01114 |
| sp|Q7KZI7|MARK2_HUMAN | 88237.0746 | 0.85 | 0.145 | 3.21E-05 |
| sp|Q9H974|QTRT2_HUMAN | 47519.9288 | 0.79 | 0.198 | 6.26E-06 |
| sp|Q9Y5Y2|NUBP2_HUMAN | 29187.96438 | 1.36 | 0.21 | 2.03E-09 |
| sp|Q99715|COCA1_HUMAN | 334119.9854 | 1.3 | 0.495 | 0.1099 |
| sp|Q9BUF5|TBB6_HUMAN | 50263.16107 | 1.49 | 0.348 | 8.34E-08 |
| sp|Q96BY6|DOC10_HUMAN | 251806.6415 | 1.02 | 0.288 | 0.7787 |
| sp|Q99895|CTRC_HUMAN | 30074.07466 | 0.63 | 0.296 | 6.71E-06 |
| sp|Q9NSE4|SYIM_HUMAN | 114670.4355 | 0.64 | 0.109 | 5.97E-13 |
| sp|Q16822|PCKGM_HUMAN | 71433.92991 | 0.88 | 0.193 | 0.002089 |
| sp|P06746|DPOLB_HUMAN | 38306.84287 | 1.26 | 0.225 | 8.24E-06 |
| sp|Q8TCD5|NT5C_HUMAN | 23578.14717 | 1.15 | 0.088 | 4.47E-09 |
| sp|P15121|ALDR_HUMAN | 36211.63342 | 1.18 | 0.173 | 3.48E-05 |
| sp|Q9Y2W1|TR150_HUMAN | 108640.0278 | 1.71 | 0.451 | 1.39E-09 |
| sp|Q8WV99|ZFN2B_HUMAN | 29069.65147 | 1 | 0.183 | 0.7809 |
| sp|Q14738|2A5D_HUMAN | 70271.48399 | 0.8 | 0.101 | 2.79E-09 |
| sp|Q9UBV2|SE1L1_HUMAN | 89193.67946 | 0.89 | 0.085 | 7.35E-07 |
| sp|Q10567|AP1B1_HUMAN | 105464.4052 | 0.72 | 0.128 | 4.46E-10 |
| sp|Q13948|CASP_HUMAN | 77618.0402 | 1.06 | 0.097 | 0.002455 |
| sp|L0R819|ASURF_HUMAN | 11224.8933 | 1.52 | 0.268 | 6.28E-11 |
| sp|P09488|GSTM1_HUMAN | 25905.12932 | 0.61 | 0.371 | 3.05E-05 |
| sp|Q6P1J9|CDC73_HUMAN | 60635.11528 | 1.09 | 0.188 | 0.04876 |
| sp|O75937|DNJC8_HUMAN | 29805.41417 | 1.5 | 0.209 | 1.57E-13 |
| sp|Q5T8D3|ACBD5_HUMAN | 60492.14862 | 0.92 | 0.371 | 0.06688 |
| sp|Q15054|DPOD3_HUMAN | 51635.39964 | 1.08 | 0.353 | 0.7476 |
| sp|Q9UJX3|APC7_HUMAN | 67422.06827 | 1.18 | 0.239 | 0.001027 |
| sp|P49815|TSC2_HUMAN | 202573.1709 | 0.67 | 0.093 | 1.22E-13 |
| sp|P62750|RL23A_HUMAN | 17666.12362 | 1.54 | 0.254 | 4.55E-12 |
| sp|Q6PJT7|ZC3HE_HUMAN | 83774.68447 | 1.26 | 0.207 | 6.44E-07 |
| sp|Q32P28|P3H1_HUMAN | 84178.39806 | 1.8 | 0.481 | 4.67E-10 |
| sp|O15240|VGF_HUMAN | 67256.58994 | 0.96 | 0.311 | 0.2098 |
| sp|O75251|NDUS7_HUMAN | 23815.21622 | 0.78 | 0.109 | 3.30E-09 |
| sp|P09467|F16P1_HUMAN | 37199.9343 | 0.82 | 0.15 | 1.87E-05 |
| sp|Q9H147|TDIF1_HUMAN | 37256.97853 | 1.88 | 0.26 | 2.20E-16 |
| sp|O60513|B4GT4_HUMAN | 40339.79123 | 0.83 | 0.187 | 9.67E-05 |
| sp|O15344|TRI18_HUMAN | 76895.22436 | 0.76 | 0.098 | 9.13E-11 |
| sp|P42345|MTOR_HUMAN | 290741.4115 | 0.66 | 0.047 | 2.20E-16 |
| sp|Q4LE39|ARI4B_HUMAN | 148784.5565 | 0.97 | 0.13 | 0.152 |
| sp|Q9UQB8|BAIP2_HUMAN | 61096.71313 | 0.93 | 0.22 | 0.04142 |
| sp|P82664|RT10_HUMAN | 23081.07449 | 1.06 | 0.104 | 0.01693 |
| sp|P09471|GNAO_HUMAN | 40577.10103 | 0.58 | 0.222 | 2.30E-08 |
| sp|O75947|ATP5H_HUMAN | 18518.50642 | 1.47 | 0.294 | 3.62E-09 |
| sp|P17301|ITA2_HUMAN | 130450.3157 | 0.58 | 0.163 | 1.32E-09 |
| sp|O00399|DCTN6_HUMAN | 21057.75495 | 1.04 | 0.101 | 0.06358 |
| sp|P12724|ECP_HUMAN | 18868.52955 | 0.64 | 0.199 | 1.16E-06 |
| sp|O14682|ENC1_HUMAN | 67266.56822 | 1.84 | 0.452 | 1.63E-11 |
| sp|P16455|MGMT_HUMAN | 21899.25503 | 0.98 | 0.175 | 0.2874 |
| sp|Q5TDH0|DDI2_HUMAN | 44932.66186 | 1.32 | 0.15 | 1.31E-11 |
| sp|P07766|CD3E_HUMAN | 23456.59263 | 0.92 | 0.163 | 0.009788 |
| sp|O00330|ODPX_HUMAN | 54298.86106 | 1.35 | 0.193 | 4.04E-10 |
| sp|P61968|LMO4_HUMAN | 18818.82248 | 1.05 | 0.123 | 0.09856 |
| sp|Q9H6R3|ACSS3_HUMAN | 75339.71939 | 0.92 | 0.136 | 0.004146 |
| sp|P01876|IGHA1_HUMAN | 38467.9498 | 0.97 | 0.347 | 0.2229 |
| sp|Q08945|SSRP1_HUMAN | 81348.53851 | 0.71 | 0.166 | 1.05E-07 |
| sp|Q9Y6G3|RM42_HUMAN | 16917.43304 | 1.23 | 0.208 | 3.39E-06 |
| sp|Q9Y3A2|UTP11_HUMAN | 30466.71318 | 1.18 | 0.2 | 0.0002467 |
| sp|Q8TEA8|DTD1_HUMAN | 23562.01379 | 0.78 | 0.202 | 8.95E-06 |
| sp|P34932|HSP74_HUMAN | 95108.55161 | 1.08 | 0.147 | 0.02897 |
| sp|Q05397|FAK1_HUMAN | 119937.5855 | 0.88 | 0.095 | 1.96E-06 |
| sp|Q92506|DHB8_HUMAN | 27280.90298 | 1.37 | 0.473 | 0.002215 |
| sp|P07954|FUMH_HUMAN | 54755.2224 | 0.72 | 0.184 | 3.94E-07 |
| sp|Q96H20|SNF8_HUMAN | 28942.12944 | 0.58 | 0.126 | 9.86E-13 |
| sp|P13804|ETFA_HUMAN | 35381.70023 | 1.41 | 0.321 | 5.12E-07 |
| sp|Q9HB63|NET4_HUMAN | 72743.39582 | 0.95 | 0.104 | 0.01228 |
| sp|Q14722|KCAB1_HUMAN | 46972.04318 | 0.64 | 0.193 | 3.23E-09 |
| sp|Q5SRE7|PHYD1_HUMAN | 32600.32424 | 1.41 | 0.446 | 8.69E-05 |
| sp|Q9UK58|CCNL1_HUMAN | 59978.33132 | 0.87 | 0.074 | 6.99E-09 |
| sp|Q9Y3T6|R3HC1_HUMAN | 49442.44619 | 0.84 | 0.185 | 0.0001788 |
| sp|Q9NYJ1|COA4_HUMAN | 10337.73109 | 1.28 | 0.305 | 0.0001321 |
| sp|Q9Y2H5|PKHA6_HUMAN | 117380.3307 | 0.78 | 0.326 | 0.0008335 |
| sp|Q15459|SF3A1_HUMAN | 88869.52164 | 1.36 | 0.154 | 2.45E-12 |
| sp|P16422|EPCAM_HUMAN | 35575.94134 | 0.48 | 0.306 | 3.00E-07 |
| sp|A4D1U4|LCHN_HUMAN | 52078.87996 | 1.11 | 0.173 | 0.004044 |
| sp|O43660|PLRG1_HUMAN | 57482.2202 | 0.65 | 0.118 | 7.29E-12 |
| sp|O94886|CSCL1_HUMAN | 93530.5873 | 0.73 | 0.093 | 1.18E-11 |
| sp|O00629|IMA3_HUMAN | 58345.82494 | 0.32 | 0.305 | 1.25E-08 |
| sp|Q9Y484|WIPI4_HUMAN | 40622.23501 | 0.68 | 0.124 | 6.88E-11 |
| sp|O60888|CUTA_HUMAN | 19200.14482 | 1.18 | 0.28 | 0.005636 |
| sp|Q9UHX1|PUF60_HUMAN | 59990.67809 | 1.22 | 0.186 | 1.75E-06 |
| sp|Q96IU2|ZBED3_HUMAN | 25440.74238 | 1.08 | 0.368 | 0.6537 |
| sp|P29372|3MG_HUMAN | 33228.9326 | 0.71 | 0.175 | 7.98E-08 |
| sp|Q6MZP7|LIN54_HUMAN | 80794.64426 | 1.14 | 0.197 | 0.001958 |
| sp|Q02985|FHR3_HUMAN | 38478.18754 | 0.82 | 0.257 | 0.001964 |
| sp|P69892|HBG2_HUMAN | 16155.30669 | 1.11 | 0.651 | 0.7346 |
| sp|P48553|TPC10_HUMAN | 143791.0399 | 0.69 | 0.096 | 1.31E-12 |
| sp|P01137|TGFB1_HUMAN | 44979.04357 | 1.43 | 0.284 | 1.20E-08 |
| sp|P07098|LIPG_HUMAN | 45361.76001 | 0.3 | 0.143 | 2.88E-13 |
| sp|Q13617|CUL2_HUMAN | 87535.99259 | 0.63 | 0.068 | 2.20E-16 |
| sp|P08581|MET_HUMAN | 157761.3452 | 0.86 | 0.131 | 3.26E-05 |
| sp|P28330|ACADL_HUMAN | 48006.48637 | 2.12 | 0.882 | 3.94E-08 |
| sp|Q9BRJ7|TIRR_HUMAN | 23476.274 | 0.73 | 0.137 | 3.64E-09 |
| sp|Q14534|ERG1_HUMAN | 64263.50905 | 0.77 | 0.109 | 6.53E-10 |
| sp|Q6ZS30|NBEL1_HUMAN | 310216.602 | 0.62 | 0.184 | 2.49E-08 |
| sp|O75051|PLXA2_HUMAN | 214201.6501 | 0.52 | 0.157 | 3.34E-10 |
| sp|Q9UL12|SARDH_HUMAN | 101923.8957 | 0.68 | 0.093 | 4.91E-13 |
| sp|Q9HA77|SYCM_HUMAN | 62679.84489 | 1.04 | 0.211 | 0.5859 |
| sp|Q9Y5U2|TSSC4_HUMAN | 34457.68104 | 1.06 | 0.238 | 0.4135 |
| sp|O60343|TBCD4_HUMAN | 148049.5109 | 1.11 | 0.163 | 0.002126 |
| sp|Q5K4L6|S27A3_HUMAN | 74227.20964 | 0.62 | 0.115 | 5.60E-12 |
| sp|Q8WUB8|PHF10_HUMAN | 57023.4844 | 1.17 | 0.163 | 3.76E-05 |
| sp|Q9BVC4|LST8_HUMAN | 36519.46953 | 1.11 | 0.048 | 1.66E-11 |
| sp|Q8N983|RM43_HUMAN | 23683.26304 | 1.03 | 0.215 | 0.8759 |
| sp|Q9UPU5|UBP24_HUMAN | 297182.3523 | 0.56 | 0.113 | 1.03E-13 |
| sp|Q9NQV6|PRD10_HUMAN | 131632.965 | 0.71 | 0.139 | 7.85E-09 |
| sp|Q9H706|GARE1_HUMAN | 98532.3335 | 0.74 | 0.11 | 1.91E-10 |
| sp|P0DOX3|IGD_HUMAN | 56741.29158 | 0.79 | 0.205 | 8.09E-06 |
| sp|P49748|ACADV_HUMAN | 70726.56787 | 0.64 | 0.176 | 1.82E-08 |
| sp|Q66GS9|CP135_HUMAN | 133846.3416 | 1.38 | 0.287 | 2.84E-08 |
| sp|Q14195|DPYL3_HUMAN | 62305.23492 | 1.41 | 0.343 | 2.45E-06 |
| sp|Q8TAD4|ZNT5_HUMAN | 84885.9631 | 0.66 | 0.145 | 4.28E-10 |
| sp|P29120|NEC1_HUMAN | 84708.50612 | 0.69 | 0.316 | 9.00E-05 |
| sp|P46926|GNPI1_HUMAN | 32800.63503 | 0.82 | 0.238 | 0.0003161 |
| sp|O43556|SGCE_HUMAN | 50200.25659 | 0.48 | 0.175 | 2.06E-11 |
| sp|Q8NC51|PAIRB_HUMAN | 44977.47062 | 1.44 | 0.364 | 6.23E-07 |
| sp|O15327|INP4B_HUMAN | 105736.8629 | 0.69 | 0.11 | 7.08E-12 |
| sp|Q9UKJ3|GPTC8_HUMAN | 164992.4246 | 1.21 | 0.122 | 2.09E-09 |
| sp|A6NFY7|SDHF1_HUMAN | 12837.65336 | 1.37 | 0.557 | 0.004609 |
| sp|Q96DE0|NUD16_HUMAN | 21299.13187 | 0.68 | 0.054 | 2.20E-16 |
| sp|O75340|PDCD6_HUMAN | 21893.79631 | 0.69 | 0.128 | 1.95E-10 |
| sp|Q8N5G2|MACOI_HUMAN | 76681.73031 | 1.16 | 0.149 | 1.02E-05 |
| sp|Q9P2J5|SYLC_HUMAN | 135558.916 | 0.78 | 0.103 | 5.98E-10 |
| sp|P29373|RABP2_HUMAN | 15836.07423 | 3.03 | 1.467 | 1.47E-08 |
| sp|Q9BVS5|TR61B_HUMAN | 53597.96045 | 0.99 | 0.173 | 0.4486 |
| sp|Q13123|RED_HUMAN | 65657.79999 | 1.71 | 0.38 | 2.78E-11 |
| sp|Q6UXG2|K1324_HUMAN | 114427.0986 | 0.55 | 0.205 | 6.03E-09 |
| sp|O95816|BAG2_HUMAN | 23910.26482 | 1.06 | 0.346 | 0.9465 |
| sp|Q13332|PTPRS_HUMAN | 218141.2741 | 1.58 | 0.449 | 3.36E-08 |
| sp|Q92619|HMHA1_HUMAN | 125829.9413 | 1.15 | 0.156 | 9.67E-05 |
| sp|Q9P0R6|GSKIP_HUMAN | 15791.48063 | 1.14 | 0.363 | 0.1322 |
| sp|P14151|LYAM1_HUMAN | 43623.44331 | 1.32 | 0.495 | 0.02404 |
| sp|O76003|GLRX3_HUMAN | 37675.32988 | 1.11 | 0.169 | 0.006002 |
| sp|Q99417|MYCBP_HUMAN | 11941.26595 | 1.15 | 0.301 | 0.04314 |
| sp|P15311|EZRI_HUMAN | 69465.77019 | 1.22 | 0.295 | 0.003087 |
| sp|P41208|CETN2_HUMAN | 19707.86431 | 1.69 | 0.392 | 1.04E-10 |
| sp|Q92783|STAM1_HUMAN | 59523.17815 | 1.16 | 0.213 | 0.001315 |
| sp|Q8NBF2|NHLC2_HUMAN | 80230.76636 | 0.8 | 0.124 | 5.23E-08 |
| sp|Q6P5W5|S39A4_HUMAN | 69201.44285 | 0.95 | 0.193 | 0.1281 |
| sp|O95071|UBR5_HUMAN | 312333.5798 | 0.82 | 0.098 | 7.95E-09 |
| sp|Q9BTE3|MCMBP_HUMAN | 73771.09548 | 0.97 | 0.146 | 0.1742 |
| sp|Q9Y240|CLC11_HUMAN | 35996.62251 | 1.25 | 0.358 | 0.001376 |
| sp|Q9BXM9|FSD1L_HUMAN | 60206.92497 | 0.82 | 0.391 | 0.003963 |
| sp|P08567|PLEK_HUMAN | 40480.50441 | 1.23 | 0.282 | 0.0005131 |
| sp|Q92882|OSTF1_HUMAN | 23924.98762 | 1.32 | 0.156 | 2.68E-11 |
| sp|O43823|AKAP8_HUMAN | 76613.28473 | 1.05 | 0.203 | 0.415 |
| sp|A6NKD9|CC85C_HUMAN | 45449.06262 | 1.09 | 0.133 | 0.002798 |
| sp|Q8NFH8|REPS2_HUMAN | 71984.34268 | 0.96 | 0.34 | 0.1571 |
| sp|O75528|TADA3_HUMAN | 49082.33703 | 1.48 | 0.21 | 4.12E-13 |
| sp|P98194|AT2C1_HUMAN | 101634.6626 | 0.65 | 0.096 | 2.87E-13 |
| sp|Q96FW1|OTUB1_HUMAN | 31474.4927 | 1.09 | 0.161 | 0.03186 |
| sp|Q9GZQ3|COMD5_HUMAN | 24807.93972 | 0.59 | 0.085 | 1.62E-15 |
| sp|Q3KRA6|CB076_HUMAN | 14638.65157 | 1.35 | 0.354 | 7.15E-05 |
| sp|Q5JRA6|TGO1_HUMAN | 214236.5751 | 0.78 | 0.169 | 2.92E-06 |
| sp|O15031|PLXB2_HUMAN | 207716.199 | 0.84 | 0.133 | 9.44E-06 |
| sp|Q15477|SKIV2_HUMAN | 138619.3298 | 0.83 | 0.096 | 3.16E-08 |
| sp|Q9H0R6|GATA_HUMAN | 58090.30356 | 1 | 0.125 | 0.7632 |
| sp|P62273|RS29_HUMAN | 6882.378739 | 0.95 | 0.186 | 0.09143 |
| sp|P08697|A2AP_HUMAN | 54855.18349 | 1.01 | 0.371 | 0.4217 |
| sp|P07942|LAMB1_HUMAN | 205132.2924 | 1.09 | 0.186 | 0.04706 |
| sp|O00534|VMA5A_HUMAN | 87157.24268 | 0.89 | 0.21 | 0.006678 |
| sp|P46782|RS5_HUMAN | 23015.10621 | 0.89 | 0.201 | 0.00364 |
| sp|Q8IWE4|DCNL3_HUMAN | 34821.84745 | 0.73 | 0.25 | 1.75E-05 |
| sp|O60841|IF2P_HUMAN | 139179.8223 | 0.82 | 0.084 | 8.83E-10 |
| sp|Q8NHJ6|LIRB4_HUMAN | 49648.81078 | 1 | 0.231 | 0.62 |
| sp|Q15652|JHD2C_HUMAN | 286383.4091 | 1.32 | 0.194 | 9.46E-09 |
| sp|Q9HBR0|S38AA_HUMAN | 120353.4352 | 0.94 | 0.115 | 0.008978 |
| sp|P06276|CHLE_HUMAN | 68926.47432 | 1.11 | 0.341 | 0.4013 |
| sp|Q8WXW3|PIBF1_HUMAN | 90016.69805 | 1.28 | 0.16 | 6.27E-10 |
| sp|Q8IXQ6|PARP9_HUMAN | 97289.81506 | 1.04 | 0.182 | 0.6172 |
| sp|P67936|TPM4_HUMAN | 28600.5234 | 1.53 | 0.417 | 1.20E-07 |
| sp|Q96DI7|SNR40_HUMAN | 39723.86958 | 1.23 | 0.099 | 4.17E-12 |
| sp|Q13496|MTM1_HUMAN | 70382.74247 | 0.62 | 0.103 | 5.98E-14 |
| sp|O75190|DNJB6_HUMAN | 36103.88354 | 1.33 | 0.176 | 1.74E-10 |
| sp|P12429|ANXA3_HUMAN | 36505.71252 | 0.74 | 0.22 | 5.24E-06 |
| sp|P17676|CEBPB_HUMAN | 36464.29011 | 1.11 | 0.353 | 0.3192 |
| sp|Q7Z403|TMC6_HUMAN | 91337.72501 | 0.93 | 0.132 | 0.011 |
| sp|Q9UNW1|MINP1_HUMAN | 55625.3087 | 1.15 | 0.239 | 0.01078 |
| sp|O43708|MAAI_HUMAN | 24463.81613 | 0.69 | 0.112 | 3.59E-11 |
| sp|Q12884|SEPR_HUMAN | 88322.91455 | 0.91 | 0.456 | 0.04794 |
| sp|O43704|ST1B1_HUMAN | 35029.69236 | 1.1 | 0.633 | 0.4821 |
| sp|Q7Z333|SETX_HUMAN | 307004.3493 | 0.96 | 0.213 | 0.1893 |
| sp|P18031|PTN1_HUMAN | 50487.02312 | 1.12 | 0.302 | 0.1611 |
| sp|Q9HAF1|EAF6_HUMAN | 21603.86932 | 1.23 | 0.326 | 0.0008503 |
| sp|P02763|A1AG1_HUMAN | 23706.83463 | 1.26 | 0.361 | 0.003064 |
| sp|P01860|IGHG3_HUMAN | 42268.73682 | 1.16 | 0.375 | 0.1686 |
| sp|Q9UBY8|CLN8_HUMAN | 33146.79933 | 0.53 | 0.159 | 8.42E-12 |
| sp|Q5SW79|CE170_HUMAN | 175567.9111 | 1.33 | 0.264 | 2.74E-07 |
| sp|Q9H4F8|SMOC1_HUMAN | 49425.23041 | 1 | 0.68 | 0.1327 |
| sp|Q9NP81|SYSM_HUMAN | 58684.01619 | 0.93 | 0.164 | 0.01904 |
| sp|Q13428|TCOF_HUMAN | 152225.3983 | 1.4 | 0.254 | 1.48E-09 |
| sp|P02786|TFR1_HUMAN | 85256.10474 | 0.56 | 0.126 | 6.05E-13 |
| sp|P32322|P5CR1_HUMAN | 33549.63186 | 1.06 | 0.219 | 0.3399 |
| sp|Q13535|ATR_HUMAN | 304746.2405 | 0.84 | 0.193 | 0.0001255 |
| sp|Q9BYD3|RM04_HUMAN | 34936.34943 | 1.04 | 0.165 | 0.3367 |
| sp|Q8IUR0|TPPC5_HUMAN | 20866.1817 | 0.55 | 0.13 | 1.23E-12 |
| sp|Q9Y646|CBPQ_HUMAN | 52064.58866 | 0.67 | 0.19 | 9.21E-08 |
| sp|Q9NZN5|ARHGC_HUMAN | 174076.4647 | 1.09 | 0.116 | 0.001532 |
| sp|Q9NPD3|EXOS4_HUMAN | 26633.58949 | 1.11 | 0.342 | 0.4653 |
| sp|O75794|CD123_HUMAN | 39548.3122 | 0.75 | 0.146 | 5.73E-08 |
| sp|Q5T4S7|UBR4_HUMAN | 580529.1018 | 0.62 | 0.054 | 2.20E-16 |
| sp|O15553|MEFV_HUMAN | 87341.33483 | 1.31 | 0.385 | 0.0003211 |
| sp|Q14019|COTL1_HUMAN | 16031.06697 | 1.22 | 0.293 | 0.003249 |
| sp|P07996|TSP1_HUMAN | 133273.0472 | 1.32 | 0.761 | 0.5627 |
| sp|P17516|AK1C4_HUMAN | 37424.08488 | 0.89 | 0.34 | 0.02469 |
| sp|P02679|FIBG_HUMAN | 52088.09243 | 1.64 | 1.215 | 0.01557 |
| sp|O00233|PSMD9_HUMAN | 24819.56045 | 1.17 | 0.083 | 5.66E-11 |
| sp|P36957|ODO2_HUMAN | 49048.57934 | 1.27 | 0.121 | 6.52E-12 |
| sp|P31689|DNJA1_HUMAN | 45562.72099 | 1.3 | 0.263 | 2.88E-06 |
| sp|Q9UET6|TRM7_HUMAN | 36665.28619 | 0.86 | 0.157 | 8.57E-05 |
| sp|P45973|CBX5_HUMAN | 22364.10797 | 1.25 | 0.245 | 8.18E-06 |
| sp|P46783|RS10_HUMAN | 18867.85598 | 1.1 | 0.154 | 0.006729 |
| sp|P07205|PGK2_HUMAN | 45148.46949 | 0.97 | 0.123 | 0.1719 |
| sp|Q9NVA2|SEP11_HUMAN | 49634.29069 | 1.86 | 0.321 | 1.48E-14 |
| sp|P80723|BASP1_HUMAN | 22662.00121 | 1.88 | 0.431 | 1.04E-12 |
| sp|P08637|FCG3A_HUMAN | 29337.78485 | 1.25 | 0.661 | 0.4282 |
| sp|Q5R372|RBG1L_HUMAN | 93348.20131 | 0.72 | 0.194 | 5.31E-07 |
| sp|P28838|AMPL_HUMAN | 56511.95105 | 1.13 | 0.476 | 0.5079 |
| sp|Q5W0Z9|ZDH20_HUMAN | 43030.19172 | 0.75 | 0.148 | 1.27E-07 |
| sp|Q05682|CALD1_HUMAN | 93214.43751 | 1.06 | 0.379 | 0.8818 |
| sp|Q96A26|F162A_HUMAN | 17541.25491 | 0.73 | 0.278 | 3.21E-05 |
| sp|Q9HCH0|NCK5L_HUMAN | 140220.7914 | 1.4 | 0.342 | 2.07E-06 |
| sp|Q8N3Y7|RDHE2_HUMAN | 34567.96627 | 0.41 | 0.365 | 3.74E-07 |
| sp|Q9UHN6|CEIP2_HUMAN | 155683.9669 | 0.72 | 0.058 | 2.20E-16 |
| sp|P02751|FINC_HUMAN | 266034.4438 | 1.28 | 0.552 | 0.09551 |
| sp|Q14191|WRN_HUMAN | 164391.7126 | 0.8 | 0.237 | 0.0004828 |
| sp|Q08J23|NSUN2_HUMAN | 87196.09239 | 1.12 | 0.165 | 0.001897 |
| sp|Q5M9Q1|NKAPL_HUMAN | 46551.2296 | 1.2 | 0.258 | 0.0009491 |
| sp|P35613|BASI_HUMAN | 42555.20022 | 0.58 | 0.104 | 3.99E-14 |
| sp|O43264|ZW10_HUMAN | 89610.18091 | 0.58 | 0.124 | 1.56E-12 |
| sp|Q9UBW8|CSN7A_HUMAN | 30411.08278 | 1.06 | 0.132 | 0.04675 |
| sp|Q16853|AOC3_HUMAN | 85120.49321 | 0.84 | 0.248 | 0.001249 |
| sp|Q9P086|MED11_HUMAN | 13216.71943 | 1.04 | 0.136 | 0.2415 |
| sp|Q9BST9|RTKN_HUMAN | 63408.02702 | 0.93 | 0.179 | 0.0286 |
| sp|Q15751|HERC1_HUMAN | 538772.1949 | 0.87 | 0.131 | 0.0001035 |
| sp|P11387|TOP1_HUMAN | 91107.06912 | 1.14 | 0.422 | 0.3187 |
| sp|O00585|CCL21_HUMAN | 14960.90783 | 1.08 | 0.575 | 0.6333 |
| sp|A8MXQ7|YH010_HUMAN | 68748.18905 | 0.7 | 0.215 | 3.18E-06 |
| sp|O95841|ANGL1_HUMAN | 57121.53152 | 1.37 | 0.639 | 0.009503 |
| sp|Q9NZI8|IF2B1_HUMAN | 63765.29423 | 1.13 | 0.706 | 0.2726 |
| sp|Q6P1R4|DUS1L_HUMAN | 54318.53418 | 0.66 | 0.163 | 3.47E-09 |
| sp|P51580|TPMT_HUMAN | 28600.29993 | 0.77 | 0.159 | 2.64E-07 |
| sp|O15417|TNC18_HUMAN | 315961.6213 | 0.81 | 0.147 | 1.21E-06 |
| sp|P0DPB6|RPAC2_HUMAN | 15380.50292 | 1.07 | 0.197 | 0.1676 |
| sp|Q03135|CAV1_HUMAN | 20611.53511 | 0.8 | 0.251 | 0.0001988 |
| sp|Q15911|ZFHX3_HUMAN | 408822.5616 | 1.5 | 0.172 | 3.17E-15 |
| sp|P16112|PGCA_HUMAN | 263089.293 | 1.15 | 0.166 | 0.0001468 |
| sp|P35232|PHB_HUMAN | 29824.91341 | 1.11 | 0.179 | 0.01124 |
| sp|P61024|CKS1_HUMAN | 9635.925353 | 1.22 | 0.3 | 0.0009075 |
| sp|Q9HD89|RETN_HUMAN | 12077.70871 | 0.61 | 0.448 | 2.19E-05 |
| sp|O95466|FMNL1_HUMAN | 122499.9809 | 0.88 | 0.174 | 0.001206 |
| sp|Q495W5|FUT11_HUMAN | 56275.83247 | 1.14 | 0.178 | 0.0007715 |
| sp|P43652|AFAM_HUMAN | 70944.73268 | 0.63 | 0.157 | 1.48E-09 |
| sp|P48449|ERG7_HUMAN | 84434.55603 | 0.68 | 0.072 | 1.26E-15 |
| sp|Q5GLZ8|HERC4_HUMAN | 119894.8658 | 0.69 | 0.111 | 3.06E-11 |
| sp|P19105|ML12A_HUMAN | 19820.50134 | 1.49 | 0.191 | 9.16E-14 |
| sp|P04004|VTNC_HUMAN | 55051.45765 | 0.85 | 0.296 | 0.006865 |
| sp|P78536|ADA17_HUMAN | 94939.04133 | 1.14 | 0.111 | 1.53E-06 |
| sp|Q96GC5|RM48_HUMAN | 24015.7886 | 1.28 | 0.236 | 1.24E-06 |
| sp|Q96I99|SUCB2_HUMAN | 46805.55393 | 1.19 | 0.406 | 0.1375 |
| sp|P04233|HG2A_HUMAN | 33931.8351 | 1.16 | 0.29 | 0.02605 |
| sp|P35080|PROF2_HUMAN | 15360.38387 | 1.38 | 0.485 | 0.001653 |
| sp|Q15154|PCM1_HUMAN | 230038.9453 | 1.23 | 0.153 | 6.98E-08 |
| sp|Q9GZT9|EGLN1_HUMAN | 46829.146 | 1.26 | 0.258 | 1.01E-05 |
| sp|Q7KZF4|SND1_HUMAN | 102599.7566 | 0.99 | 0.174 | 0.4868 |
| sp|P01871|IGHM_HUMAN | 50074.82728 | 1.21 | 0.258 | 0.0003917 |
| sp|Q9BYD6|RM01_HUMAN | 37095.48114 | 1.58 | 0.426 | 7.21E-08 |
| sp|P15880|RS2_HUMAN | 31571.69196 | 0.81 | 0.17 | 1.10E-05 |
| sp|Q9TQE0|2B19_HUMAN | 30131.08938 | 1.05 | 0.528 | 0.4125 |
| sp|Q86W25|NAL13_HUMAN | 121126.4642 | 1.52 | 0.679 | 0.0005786 |
| sp|Q8NC44|RETR2_HUMAN | 58118.25928 | 0.62 | 0.126 | 8.27E-12 |
| sp|Q9NQT8|KI13B_HUMAN | 203956.0641 | 1 | 0.233 | 0.5471 |
| sp|O60506|HNRPQ_HUMAN | 69769.67278 | 1.3 | 0.135 | 1.00E-11 |
| sp|P06730|IF4E_HUMAN | 25291.58719 | 1.15 | 0.145 | 3.07E-05 |
| sp|P26022|PTX3_HUMAN | 42501.04223 | 0.83 | 0.161 | 2.81E-05 |
| sp|P06239|LCK_HUMAN | 58459.0307 | 1.14 | 0.311 | 0.1137 |
| sp|P84022|SMAD3_HUMAN | 48887.06267 | 1 | 0.168 | 0.7657 |
| sp|Q12805|FBLN3_HUMAN | 56867.08191 | 0.79 | 0.352 | 0.001134 |
| sp|Q9UK23|NAGPA_HUMAN | 57615.65322 | 0.86 | 0.053 | 3.02E-12 |
| sp|P22223|CADH3_HUMAN | 91856.77446 | 0.74 | 0.25 | 2.50E-05 |
| sp|Q9H9T3|ELP3_HUMAN | 62771.25947 | 1.08 | 0.091 | 0.0002816 |
| sp|Q15286|RAB35_HUMAN | 23277.86349 | 0.54 | 0.067 | 2.20E-16 |
| sp|Q08623|HDHD1_HUMAN | 25385.96434 | 0.88 | 0.241 | 0.004921 |
| sp|P08962|CD63_HUMAN | 26456.40325 | 0.42 | 0.183 | 5.05E-11 |
| sp|O95622|ADCY5_HUMAN | 140739.0035 | 0.6 | 0.131 | 1.74E-11 |
| sp|P16401|H15_HUMAN | 22548.45774 | 1.65 | 0.714 | 0.0006315 |
| sp|Q08495|DEMA_HUMAN | 45582.25677 | 1.8 | 0.334 | 8.92E-14 |
| sp|Q9NS15|LTBP3_HUMAN | 146433.4769 | 1.31 | 0.477 | 0.04408 |
| sp|Q99611|SPS2_HUMAN | 47713.8901 | 0.95 | 0.123 | 0.03006 |
| sp|Q9BSH4|TACO1_HUMAN | 32894.86604 | 1.24 | 0.158 | 1.71E-08 |
| sp|A0A075B6J9|LV218_HUMAN | 12499.98392 | 1.71 | 0.665 | 8.77E-06 |
| sp|O14602|IF1AY_HUMAN | 16528.30078 | 1.87 | 0.77 | 6.52E-06 |
| sp|P20774|MIME_HUMAN | 34225.00284 | 1.87 | 1.01 | 4.65E-06 |
| sp|P13645|K1C10_HUMAN | 59001.76894 | 1.53 | 0.538 | 5.93E-05 |
| sp|P36222|CH3L1_HUMAN | 42979.53957 | 0.63 | 0.383 | 2.44E-05 |
| sp|A6NHA9|O4C46_HUMAN | 35233.10531 | 0.7 | 0.504 | 0.0003419 |
| sp|Q15366|PCBP2_HUMAN | 38936.73122 | 1.06 | 0.215 | 0.3197 |
| sp|Q9H9S4|CB39L_HUMAN | 39215.82621 | 0.3 | 0.179 | 2.74E-12 |
| sp|Q8IXK0|PHC2_HUMAN | 91323.00078 | 1.6 | 0.431 | 7.89E-09 |
| sp|Q5T1M5|FKB15_HUMAN | 134042.0686 | 1.42 | 0.093 | 2.20E-16 |
| sp|P62837|UB2D2_HUMAN | 16934.49797 | 0.56 | 0.073 | 2.20E-16 |
| sp|Q9BYG0|B3GN5_HUMAN | 44462.22364 | 0.76 | 0.097 | 7.67E-11 |
| sp|Q5QGZ9|CL12A_HUMAN | 31236.58095 | 0.64 | 0.365 | 2.57E-05 |
| sp|Q9UHI6|DDX20_HUMAN | 92962.79521 | 0.74 | 0.077 | 1.01E-13 |
| sp|Q9Y2B0|CNPY2_HUMAN | 20963.28881 | 1.3 | 0.224 | 7.36E-08 |
| sp|O75475|PSIP1_HUMAN | 60162.71206 | 1.59 | 0.246 | 3.87E-13 |
| sp|Q96HB5|CC120_HUMAN | 68135.02218 | 0.72 | 0.193 | 1.39E-07 |
| sp|Q8NFX7|STXB6_HUMAN | 23806.03204 | 0.89 | 0.086 | 1.34E-06 |
| sp|Q9UBF2|COPG2_HUMAN | 98682.00169 | 0.58 | 0.065 | 2.20E-16 |
| sp|Q13753|LAMC2_HUMAN | 134751.1275 | 1.3 | 0.386 | 0.001352 |
| sp|O43414|ERI3_HUMAN | 37708.86096 | 0.42 | 0.182 | 5.41E-12 |
| sp|Q13671|RIN1_HUMAN | 84883.82839 | 0.94 | 0.457 | 0.09127 |
| sp|P25092|GUC2C_HUMAN | 124332.0953 | 0.62 | 0.269 | 3.09E-06 |
| sp|Q9BYK8|HELZ2_HUMAN | 298267.6383 | 0.83 | 0.174 | 7.08E-05 |
| sp|O00186|STXB3_HUMAN | 68615.17672 | 0.84 | 0.077 | 7.38E-10 |
| sp|Q92995|UBP13_HUMAN | 97988.31847 | 1.01 | 0.116 | 0.965 |
| sp|Q13242|SRSF9_HUMAN | 25622.4617 | 1.34 | 0.146 | 7.69E-13 |
| sp|Q9NTI5|PDS5B_HUMAN | 165799.7527 | 0.7 | 0.064 | 2.20E-16 |
| sp|Q9UBI9|HDC_HUMAN | 60263.18368 | 1.01 | 0.088 | 0.8799 |
| sp|Q9Y250|LZTS1_HUMAN | 66953.04227 | 1.38 | 0.359 | 3.61E-05 |
| sp|Q9Y6X5|ENPP4_HUMAN | 51989.08142 | 0.94 | 0.137 | 0.01903 |
| sp|Q9NS71|GKN1_HUMAN | 22251.71825 | 0.69 | 0.333 | 0.001621 |
| sp|Q8NBJ4|GOLM1_HUMAN | 45458.82382 | 0.93 | 0.439 | 0.07605 |
| sp|P35268|RL22_HUMAN | 14816.81502 | 1.28 | 0.156 | 5.80E-10 |
| sp|Q9UBK9|UXT_HUMAN | 18273.62138 | 1.13 | 0.657 | 0.994 |
| sp|P30305|MPIP2_HUMAN | 65612.69633 | 0.9 | 0.354 | 0.03655 |
| sp|Q16773|KAT1_HUMAN | 48168.25551 | 0.72 | 0.122 | 7.10E-10 |
| sp|Q9NW61|PKHJ1_HUMAN | 17692.97743 | 1.4 | 0.295 | 4.82E-07 |
| sp|Q12981|SEC20_HUMAN | 26268.97793 | 0.76 | 0.133 | 2.13E-08 |
| sp|O14735|CDIPT_HUMAN | 23847.24813 | 0.47 | 0.178 | 5.88E-11 |
| sp|Q9Y676|RT18B_HUMAN | 29701.13148 | 1.04 | 0.123 | 0.1325 |
| sp|Q93015|NAA80_HUMAN | 31692.55018 | 0.71 | 0.115 | 1.35E-10 |
| sp|Q13542|4EBP2_HUMAN | 13027.35875 | 1.09 | 0.2 | 0.07467 |
| sp|P34982|OR1D2_HUMAN | 35825.63876 | 3.32 | 2.785 | 0.001055 |
| sp|Q8N2G8|GHDC_HUMAN | 58039.49829 | 0.65 | 0.108 | 1.54E-12 |
| sp|Q96E52|OMA1_HUMAN | 60747.68544 | 0.46 | 0.223 | 1.89E-08 |
| sp|O75339|CILP1_HUMAN | 134742.8455 | 1.87 | 0.753 | 2.26E-08 |
| sp|Q9NQC1|JADE2_HUMAN | 88874.65518 | 1.36 | 0.295 | 9.32E-07 |
| sp|Q6UX72|B3GN9_HUMAN | 44047.9212 | 1.06 | 0.153 | 0.1547 |
| sp|P04196|HRG_HUMAN | 60492.22205 | 1.27 | 0.343 | 0.002632 |
| sp|P39210|MPV17_HUMAN | 19930.46051 | 1.16 | 0.163 | 0.0001305 |
| sp|Q96G46|DUS3L_HUMAN | 73670.28765 | 1.22 | 0.279 | 0.0007813 |
| sp|O75140|DEPD5_HUMAN | 182784.1291 | 0.94 | 0.122 | 0.01763 |
| sp|Q96S44|PRPK_HUMAN | 28238.87829 | 1.26 | 0.109 | 1.60E-12 |
| sp|P29590|PML_HUMAN | 99124.49762 | 1.36 | 0.162 | 2.78E-12 |
| sp|Q9UHG2|PCSK1_HUMAN | 27394.79121 | 1.02 | 0.295 | 0.7247 |
| sp|P29279|CTGF_HUMAN | 40270.67491 | 1.16 | 0.327 | 0.0546 |
| sp|Q13976|KGP1_HUMAN | 76925.19256 | 0.67 | 0.183 | 3.56E-08 |
| sp|P68402|PA1B2_HUMAN | 25706.17645 | 1.1 | 0.186 | 0.02255 |
| sp|Q9NQX3|GEPH_HUMAN | 80364.22524 | 1.04 | 0.149 | 0.4202 |
| sp|P49427|UB2R1_HUMAN | 26873.0921 | 1.36 | 0.245 | 2.81E-08 |
| sp|Q96EE3|SEH1_HUMAN | 40118.58262 | 0.69 | 0.129 | 1.98E-10 |
| sp|Q53GS9|SNUT2_HUMAN | 65720.83552 | 0.68 | 0.087 | 7.06E-14 |
| sp|Q05084|ICA69_HUMAN | 55162.42964 | 0.89 | 0.297 | 0.01564 |
| sp|Q9H0J9|PAR12_HUMAN | 80477.64646 | 0.82 | 0.079 | 2.42E-10 |
| sp|A0A0B4J1Y8|LV949_HUMAN | 13111.55391 | 1.44 | 0.346 | 8.75E-07 |
| sp|Q96GA7|SDSL_HUMAN | 35204.29638 | 0.89 | 0.173 | 0.003029 |
| sp|O60885|BRD4_HUMAN | 152562.1086 | 1.21 | 0.124 | 4.23E-09 |
| sp|O15230|LAMA5_HUMAN | 412005.2499 | 0.69 | 0.236 | 7.83E-07 |
| sp|Q8IZR5|CKLF4_HUMAN | 26078.36763 | 0.58 | 0.237 | 4.63E-07 |
| sp|Q9NUJ1|ABHDA_HUMAN | 34234.86537 | 0.98 | 0.212 | 0.3398 |
| sp|P54920|SNAA_HUMAN | 33649.44065 | 0.85 | 0.153 | 9.99E-05 |
| sp|Q9NWZ3|IRAK4_HUMAN | 51935.09422 | 1.31 | 0.176 | 8.35E-10 |
| sp|Q9UPR3|SMG5_HUMAN | 115433.9054 | 0.44 | 0.06 | 2.20E-16 |
| sp|Q9H501|ESF1_HUMAN | 99116.12446 | 1.38 | 0.413 | 0.0001271 |
| sp|P29350|PTN6_HUMAN | 67900.19609 | 1.09 | 0.277 | 0.2861 |
| sp|Q14669|TRIPC_HUMAN | 222216.4853 | 0.77 | 0.113 | 1.60E-09 |
| sp|O75348|VATG1_HUMAN | 13845.11542 | 1.86 | 0.633 | 2.17E-09 |
| sp|P08236|BGLR_HUMAN | 75008.8104 | 0.63 | 0.122 | 3.08E-12 |
| sp|Q9UHA4|LTOR3_HUMAN | 13653.26846 | 1.28 | 0.19 | 5.56E-08 |
| sp|O95684|FR1OP_HUMAN | 43134.96038 | 1.52 | 0.378 | 3.34E-08 |
| sp|Q5H9U9|DDX6L_HUMAN | 199809.7481 | 0.77 | 0.161 | 6.38E-07 |
| sp|Q9P2A4|ABI3_HUMAN | 39334.91644 | 1.48 | 0.247 | 2.17E-11 |
| sp|P13647|K2C5_HUMAN | 62550.05227 | 1.78 | 0.492 | 1.07E-09 |
| sp|Q8TB96|TIP_HUMAN | 68445.60851 | 0.58 | 0.124 | 2.03E-12 |
| sp|Q15646|OASL_HUMAN | 59683.84296 | 0.83 | 0.259 | 0.001338 |
| sp|Q92542|NICA_HUMAN | 79084.80764 | 0.72 | 0.15 | 2.67E-08 |
| sp|Q9BWF3|RBM4_HUMAN | 40669.78839 | 1.43 | 0.229 | 2.27E-10 |
| sp|Q13057|COASY_HUMAN | 62614.06939 | 0.77 | 0.174 | 2.86E-06 |
| sp|P08910|ABHD2_HUMAN | 49063.40974 | 0.5 | 0.095 | 1.45E-15 |
| sp|Q15788|NCOA1_HUMAN | 157951.4977 | 1.07 | 0.255 | 0.3692 |
| sp|O94979|SC31A_HUMAN | 133882.3626 | 0.79 | 0.115 | 1.64E-08 |
| sp|Q9GZU7|CTDS1_HUMAN | 29509.00051 | 0.89 | 0.163 | 0.001737 |
| sp|O15530|PDPK1_HUMAN | 63607.16432 | 0.86 | 0.072 | 4.28E-09 |
| sp|Q5VW38|GP107_HUMAN | 67272.01609 | 1.21 | 0.206 | 3.42E-05 |
| sp|Q9NQS1|AVEN_HUMAN | 38692.9359 | 2.99 | 0.604 | 2.20E-16 |
| sp|Q6UXB8|PI16_HUMAN | 50105.78522 | 1.1 | 0.306 | 0.3125 |
| sp|O95359|TACC2_HUMAN | 311614.3562 | 1.05 | 0.195 | 0.4689 |
| sp|Q7L311|ARMX2_HUMAN | 65909.80429 | 1.18 | 0.261 | 0.004232 |
| sp|Q7Z304|MAMC2_HUMAN | 78571.21146 | 0.51 | 0.129 | 8.34E-14 |
| sp|Q9Y399|RT02_HUMAN | 33495.17373 | 0.46 | 0.12 | 6.41E-14 |
| sp|P29474|NOS3_HUMAN | 134826.1946 | 0.89 | 0.073 | 1.77E-07 |
| sp|Q92968|PEX13_HUMAN | 44141.27744 | 0.97 | 0.177 | 0.276 |
| sp|Q14790|CASP8_HUMAN | 56078.8829 | 1.11 | 0.169 | 0.009131 |
| sp|O75167|PHAR2_HUMAN | 69753.69228 | 1.36 | 0.369 | 4.69E-05 |
| sp|Q5T0F9|C2D1B_HUMAN | 94376.45564 | 1.29 | 0.118 | 1.31E-12 |
| sp|Q9BVM2|DPCD_HUMAN | 23378.11712 | 1.08 | 0.174 | 0.06338 |
| sp|Q16558|KCMB1_HUMAN | 22107.41546 | 0.79 | 0.119 | 2.26E-08 |
| sp|P25024|CXCR1_HUMAN | 40317.05895 | 0.66 | 0.372 | 4.81E-05 |
| sp|Q96JM3|CHAP1_HUMAN | 89994.29032 | 1.33 | 0.156 | 1.87E-11 |
| sp|P61026|RAB10_HUMAN | 22736.66435 | 0.24 | 0.047 | 2.20E-16 |
| sp|O14786|NRP1_HUMAN | 104305.0584 | 1.11 | 0.16 | 0.004158 |
| sp|Q99569|PKP4_HUMAN | 132567.3384 | 1.38 | 0.2 | 1.50E-10 |
| sp|Q16643|DREB_HUMAN | 71823.59969 | 1.89 | 0.445 | 8.83E-12 |
| sp|P24468|COT2_HUMAN | 46435.97541 | 0.92 | 0.145 | 0.004756 |
| sp|Q68CZ2|TENS3_HUMAN | 156348.0886 | 1.24 | 0.204 | 3.70E-06 |
| sp|P60484|PTEN_HUMAN | 47688.35166 | 0.85 | 0.09 | 2.16E-08 |
| sp|P62875|RPAB5_HUMAN | 7850.062162 | 1.07 | 0.153 | 0.05589 |
| sp|Q8N1S5|S39AB_HUMAN | 35355.37906 | 0.59 | 0.156 | 1.15E-08 |
| sp|Q9BYC5|FUT8_HUMAN | 66912.18618 | 1.29 | 0.325 | 8.49E-05 |
| sp|P54687|BCAT1_HUMAN | 43490.72943 | 1.54 | 0.481 | 6.85E-06 |
| sp|Q9Y6M9|NDUB9_HUMAN | 22026.91502 | 1.06 | 0.083 | 0.00269 |
| sp|Q9NY64|GTR8_HUMAN | 51280.88838 | 0.72 | 0.225 | 2.46E-06 |
| sp|P24592|IBP6_HUMAN | 26200.49621 | 1.92 | 0.821 | 3.25E-07 |
| sp|Q9NQ48|LZTL1_HUMAN | 34610.11946 | 1.64 | 0.379 | 4.68E-09 |
| sp|Q9BTY7|HGH1_HUMAN | 42540.81869 | 1.3 | 0.179 | 1.49E-09 |
| sp|Q99661|KIF2C_HUMAN | 82155.53992 | 0.98 | 0.37 | 0.253 |
| sp|Q96S66|CLCC1_HUMAN | 62649.33539 | 0.81 | 0.399 | 0.002263 |
| sp|Q96ER9|CCD51_HUMAN | 45992.8518 | 1.05 | 0.071 | 0.0008532 |
| sp|Q86U44|MTA70_HUMAN | 65156.76234 | 1.13 | 0.108 | 2.54E-06 |
| sp|P07332|FES_HUMAN | 94104.45829 | 1.08 | 0.244 | 0.2842 |
| sp|P01709|LV208_HUMAN | 12469.92707 | 1.67 | 0.29 | 2.85E-13 |
| sp|O94766|B3GA3_HUMAN | 37251.71228 | 1.14 | 0.193 | 0.001877 |
| sp|Q99698|LYST_HUMAN | 434151.2721 | 0.55 | 0.064 | 2.20E-16 |
| sp|Q96BY9|SARAF_HUMAN | 37503.87254 | 0.8 | 0.109 | 3.81E-08 |
| sp|P05090|APOD_HUMAN | 21528.85971 | 1.12 | 0.543 | 0.9762 |
| sp|O75909|CCNK_HUMAN | 64579.81748 | 0.81 | 0.166 | 3.09E-05 |
| sp|Q6UB35|C1TM_HUMAN | 106618.0694 | 0.93 | 0.172 | 0.02109 |
| sp|Q96AX2|RAB37_HUMAN | 25009.54718 | 0.58 | 0.102 | 3.67E-14 |
| sp|Q9BVJ7|DUS23_HUMAN | 16787.68926 | 0.82 | 0.14 | 3.90E-06 |
| sp|Q9NY93|DDX56_HUMAN | 61989.11557 | 0.81 | 0.147 | 2.13E-06 |
| sp|Q9Y5Q5|CORIN_HUMAN | 121408.4639 | 0.95 | 0.396 | 0.1348 |
| sp|Q5T3F8|CSCL2_HUMAN | 96474.72279 | 0.66 | 0.075 | 1.74E-15 |
| sp|P38935|SMBP2_HUMAN | 109976.2771 | 0.98 | 0.171 | 0.341 |
| sp|P23610|F8I2_HUMAN | 39459.64743 | 1.04 | 0.339 | 0.8463 |
| sp|Q9UI26|IPO11_HUMAN | 113471.1341 | 1.02 | 0.22 | 0.9751 |
| sp|Q92997|DVL3_HUMAN | 78330.04275 | 1.21 | 0.295 | 0.001418 |
| sp|A6NHC0|CAN8_HUMAN | 80102.77863 | 0.86 | 0.658 | 0.01623 |
| sp|P21333|FLNA_HUMAN | 283282.8932 | 0.7 | 0.204 | 8.99E-07 |
| sp|Q15063|POSTN_HUMAN | 93864.50068 | 1.55 | 0.445 | 4.39E-06 |
| sp|Q9UGK3|STAP2_HUMAN | 45189.44523 | 0.81 | 0.463 | 0.003057 |
| sp|Q09327|MGAT3_HUMAN | 61826.05363 | 0.86 | 0.463 | 0.01876 |
| sp|P62253|UB2G1_HUMAN | 19592.91995 | 1.57 | 0.384 | 2.44E-09 |
| sp|Q86UD3|MARH3_HUMAN | 29208.68188 | 0.46 | 0.163 | 8.86E-11 |
| sp|Q99700|ATX2_HUMAN | 140805.214 | 1.44 | 0.107 | 2.20E-16 |
| sp|Q9BUT1|BDH2_HUMAN | 27030.841 | 0.81 | 0.243 | 0.0004788 |
| sp|Q53GL7|PAR10_HUMAN | 110881.2068 | 1.09 | 0.172 | 0.0446 |
| sp|P55347|PKNX1_HUMAN | 47957.81726 | 1.49 | 0.31 | 2.19E-09 |
| sp|Q96F15|GIMA5_HUMAN | 35262.04305 | 1 | 0.301 | 0.5196 |
| sp|P0DMM9|ST1A3_HUMAN | 34270.11891 | 1.03 | 0.272 | 0.921 |
| sp|P61923|COPZ1_HUMAN | 20224.4771 | 0.69 | 0.27 | 1.24E-05 |
| sp|Q5T1C6|THEM4_HUMAN | 27549.78377 | 1.2 | 0.577 | 0.5174 |
| sp|P17028|ZNF24_HUMAN | 42738.29968 | 1.27 | 0.213 | 6.16E-07 |
| sp|P26641|EF1G_HUMAN | 50411.26059 | 0.59 | 0.112 | 3.25E-13 |
| sp|Q12792|TWF1_HUMAN | 40410.7324 | 1.08 | 0.178 | 0.06688 |
| sp|A0A0B4J2D5|GAL3B_HUMAN | 28448.81881 | 1.21 | 0.136 | 7.63E-09 |
| sp|Q9BV19|CA050_HUMAN | 22016.03735 | 1.12 | 0.218 | 0.03395 |
| sp|P83110|HTRA3_HUMAN | 49471.3837 | 1.28 | 0.463 | 0.01821 |
| sp|Q8N5W9|RFLB_HUMAN | 23191.63689 | 1.31 | 0.447 | 0.00928 |
| sp|P20700|LMNB1_HUMAN | 66634.71936 | 1.35 | 0.076 | 2.20E-16 |
| sp|Q14789|GOGB1_HUMAN | 377197.1751 | 1.24 | 0.189 | 4.70E-07 |
| sp|P02749|APOH_HUMAN | 39566.14247 | 1.25 | 0.367 | 0.004381 |
| sp|Q9Y4L1|HYOU1_HUMAN | 111476.2853 | 1.01 | 0.194 | 0.8605 |
| sp|P14854|CX6B1_HUMAN | 10395.79173 | 1.1 | 0.374 | 0.6342 |
| sp|P12544|GRAA_HUMAN | 29532.15145 | 1.45 | 0.638 | 0.0004188 |
| sp|O14727|APAF_HUMAN | 144068.8808 | 0.64 | 0.168 | 1.47E-09 |
| sp|Q13200|PSMD2_HUMAN | 100859.058 | 0.71 | 0.101 | 5.74E-12 |
| sp|Q15283|RASA2_HUMAN | 97903.42268 | 0.63 | 0.116 | 2.01E-12 |
| sp|O75354|ENTP6_HUMAN | 53536.6531 | 0.67 | 0.255 | 7.76E-05 |
| sp|Q9H492|MLP3A_HUMAN | 14302.4006 | 1.53 | 0.629 | 0.0001651 |
| sp|O60687|SRPX2_HUMAN | 54174.17567 | 1.53 | 0.35 | 1.14E-08 |
| sp|A6NHR9|SMHD1_HUMAN | 227923.7157 | 0.87 | 0.132 | 2.85E-05 |
| sp|Q86VW2|ARHGP_HUMAN | 64582.57428 | 1.09 | 0.119 | 0.0009419 |
| sp|O00418|EF2K_HUMAN | 82644.65631 | 1.25 | 0.177 | 2.37E-07 |
| sp|P57737|CORO7_HUMAN | 101607.7421 | 1.18 | 0.177 | 6.78E-05 |
| sp|P01780|HV307_HUMAN | 13087.4649 | 1.24 | 0.342 | 0.004175 |
| sp|P26583|HMGB2_HUMAN | 24171.77968 | 1.54 | 0.471 | 2.42E-06 |
| sp|Q9UL40|ZN346_HUMAN | 33520.72588 | 1.4 | 0.225 | 3.87E-10 |
| sp|Q13190|STX5_HUMAN | 39743.86111 | 0.92 | 0.114 | 0.00147 |
| sp|Q14814|MEF2D_HUMAN | 56113.4443 | 1.23 | 0.281 | 0.0004408 |
| sp|Q9UII2|ATIF1_HUMAN | 12223.29749 | 1.6 | 1.1 | 0.05699 |
| sp|Q9BW62|KATL1_HUMAN | 55681.3452 | 2.02 | 0.326 | 2.20E-16 |
| sp|Q3YBM2|T176B_HUMAN | 29475.2634 | 0.86 | 0.231 | 0.002961 |
| sp|P49789|FHIT_HUMAN | 16886.6193 | 0.86 | 0.273 | 0.00872 |
| sp|Q9BYB4|GNB1L_HUMAN | 36261.36276 | 1.1 | 0.173 | 0.01078 |
| sp|Q7Z7F0|KHDC4_HUMAN | 65014.96567 | 1.3 | 0.123 | 3.45E-13 |
| sp|Q6AI14|SL9A4_HUMAN | 90365.80913 | 1.01 | 0.338 | 0.5107 |
| sp|Q9NUI1|DECR2_HUMAN | 31082.14163 | 0.69 | 0.172 | 1.01E-07 |
| sp|P53992|SC24C_HUMAN | 119770.7201 | 0.59 | 0.103 | 1.27E-14 |
| sp|P06748|NPM_HUMAN | 32707.89553 | 1.38 | 0.179 | 1.95E-11 |
| sp|Q9NVF7|FBX28_HUMAN | 41390.50249 | 0.86 | 0.215 | 0.001256 |
| sp|Q96KN1|FA84B_HUMAN | 34663.15935 | 0.88 | 0.169 | 0.00125 |
| sp|Q5VTE0|EF1A3_HUMAN | 50477.27137 | 1.06 | 0.101 | 0.01086 |
| sp|Q13432|U119A_HUMAN | 27098.47476 | 1.29 | 0.124 | 1.47E-12 |
| sp|Q99961|SH3G1_HUMAN | 41674.11851 | 1.7 | 0.19 | 2.20E-16 |
| sp|Q02790|FKBP4_HUMAN | 52039.16812 | 1.2 | 0.204 | 1.87E-05 |
| sp|Q8NBL1|PGLT1_HUMAN | 46597.47814 | 0.83 | 0.093 | 1.60E-08 |
| sp|O75355|ENTP3_HUMAN | 59790.5223 | 0.56 | 0.239 | 5.78E-07 |
| sp|Q6YN16|HSDL2_HUMAN | 45632.61247 | 0.89 | 0.127 | 9.59E-05 |
| sp|Q9NWS0|PIHD1_HUMAN | 32495.42574 | 1.14 | 0.167 | 0.0004235 |
| sp|Q9BQA1|MEP50_HUMAN | 37424.34285 | 1.21 | 0.138 | 2.25E-08 |
| sp|Q9Y3C8|UFC1_HUMAN | 19599.07152 | 0.92 | 0.115 | 0.001918 |
| sp|Q8NEB9|PK3C3_HUMAN | 102151.0335 | 0.73 | 0.124 | 4.97E-10 |
| sp|O60814|H2B1K_HUMAN | 13863.55203 | 1.45 | 0.277 | 1.14E-09 |
| sp|P21709|EPHA1_HUMAN | 109693.5311 | 0.9 | 0.112 | 0.000138 |
| sp|Q9UG56|PISD_HUMAN | 47137.14417 | 0.7 | 0.221 | 1.23E-06 |
| sp|O75881|CP7B1_HUMAN | 58713.69919 | 0.94 | 0.239 | 0.08086 |
| sp|Q8N6R0|EFNMT_HUMAN | 79554.84064 | 0.87 | 0.112 | 4.41E-06 |
| sp|P56962|STX17_HUMAN | 33592.71475 | 1.27 | 0.223 | 1.56E-06 |
| sp|Q9BXK5|B2L13_HUMAN | 52786.64696 | 1.37 | 0.227 | 3.49E-09 |
| sp|Q9UL18|AGO1_HUMAN | 98274.53672 | 0.59 | 0.142 | 5.68E-11 |
| sp|Q92541|RTF1_HUMAN | 80474.68472 | 1.42 | 0.293 | 9.67E-08 |
| sp|O00469|PLOD2_HUMAN | 85354.8751 | 1.44 | 0.728 | 0.03436 |
| sp|O95822|DCMC_HUMAN | 55349.8943 | 0.59 | 0.187 | 5.47E-09 |
| sp|O14653|GOSR2_HUMAN | 24855.79758 | 0.85 | 0.102 | 6.19E-07 |
| sp|P61604|CH10_HUMAN | 10906.86061 | 1.2 | 0.355 | 0.01883 |
| sp|Q96A65|EXOC4_HUMAN | 111151.8388 | 0.57 | 0.101 | 2.24E-14 |
| sp|Q16637|SMN_HUMAN | 32266.64671 | 1.16 | 0.357 | 0.1058 |
| sp|O15371|EIF3D_HUMAN | 64541.65728 | 1 | 0.114 | 0.9167 |
| sp|P48960|CD97_HUMAN | 94585.16138 | 1.15 | 0.277 | 0.01765 |
| sp|Q08431|MFGM_HUMAN | 43857.78968 | 0.69 | 0.289 | 0.0001036 |
| sp|O15235|RT12_HUMAN | 15544.16033 | 1.24 | 0.333 | 0.004868 |
| sp|P06731|CEAM5_HUMAN | 77470.49552 | 0.7 | 0.368 | 0.0002151 |
| sp|Q9GZT6|CC90B_HUMAN | 29526.16123 | 1.39 | 0.229 | 1.76E-09 |
| sp|Q14432|PDE3A_HUMAN | 126422.1698 | 0.82 | 0.135 | 1.48E-06 |
| sp|Q9NRG9|AAAS_HUMAN | 60373.63727 | 0.6 | 0.113 | 4.53E-13 |
| sp|Q9HAC8|UBTD1_HUMAN | 26132.41602 | 1.21 | 0.345 | 0.006662 |
| sp|Q13077|TRAF1_HUMAN | 47085.29408 | 0.77 | 0.106 | 3.85E-10 |
| sp|Q14160|SCRIB_HUMAN | 175729.5443 | 1.05 | 0.137 | 0.1278 |
| sp|O15554|KCNN4_HUMAN | 48159.73168 | 0.69 | 0.267 | 9.92E-06 |
| sp|P13762|DRB4_HUMAN | 30303.085 | 1.07 | 0.724 | 0.2823 |
| sp|A0A075B6I1|LV460_HUMAN | 13132.32086 | 2.63 | 2.824 | 0.004446 |
| sp|Q96MW5|COG8_HUMAN | 68989.51637 | 0.76 | 0.125 | 6.74E-09 |
| sp|Q7Z3T8|ZFY16_HUMAN | 171174.0427 | 1.31 | 0.224 | 8.19E-08 |
| sp|O75665|OFD1_HUMAN | 117037.0944 | 0.92 | 0.138 | 0.002835 |
| sp|Q96TC7|RMD3_HUMAN | 52524.19919 | 1.26 | 0.183 | 6.51E-08 |
| sp|Q9ULU8|CAPS1_HUMAN | 153754.9601 | 0.67 | 0.124 | 2.56E-11 |
| sp|Q9H8Y8|GORS2_HUMAN | 47269.05982 | 1.12 | 0.093 | 3.40E-07 |
| sp|Q6UW78|UQCC3_HUMAN | 10056.21079 | 0.92 | 0.222 | 0.04103 |
| sp|Q8TDD1|DDX54_HUMAN | 98801.29501 | 0.84 | 0.152 | 1.66E-05 |
| sp|Q9HB40|RISC_HUMAN | 51064.83816 | 0.88 | 0.157 | 0.0005518 |
| sp|Q9UKY3|CES1P_HUMAN | 30755.27298 | 0.88 | 0.23 | 0.003883 |
| sp|P35790|CHKA_HUMAN | 52653.65149 | 0.72 | 0.197 | 1.57E-07 |
| sp|P13521|SCG2_HUMAN | 70879.02989 | 0.69 | 0.295 | 3.63E-05 |
| sp|Q8NAV1|PR38A_HUMAN | 37663.46126 | 0.62 | 0.105 | 2.52E-13 |
| sp|A7XYQ1|SOBP_HUMAN | 93834.88252 | 0.88 | 0.153 | 0.0005542 |
| sp|Q92854|SEM4D_HUMAN | 97439.17681 | 1.11 | 0.274 | 0.09346 |
| sp|Q9UN79|SOX13_HUMAN | 69679.69436 | 0.97 | 0.285 | 0.2226 |
| sp|O75157|T22D2_HUMAN | 79560.49303 | 1.08 | 0.19 | 0.1618 |
| sp|P04264|K2C1_HUMAN | 66152.05474 | 1.53 | 0.648 | 6.73E-05 |
| sp|Q8TD55|PKHO2_HUMAN | 53641.21833 | 1.58 | 0.187 | 1.22E-15 |
| sp|P08134|RHOC_HUMAN | 22316.37362 | 0.42 | 0.083 | 2.20E-16 |
| sp|P04180|LCAT_HUMAN | 49870.2067 | 0.93 | 0.207 | 0.03343 |
| sp|O15270|SPTC2_HUMAN | 63550.10532 | 0.55 | 0.146 | 1.08E-11 |
| sp|P38571|LICH_HUMAN | 45770.80563 | 0.73 | 0.227 | 5.31E-06 |
| sp|O43741|AAKB2_HUMAN | 30379.26527 | 1.34 | 0.218 | 1.08E-08 |
| sp|Q6ZRP7|QSOX2_HUMAN | 78202.88054 | 0.61 | 0.136 | 2.30E-11 |
| sp|Q14117|DPYS_HUMAN | 57088.87828 | 2.54 | 1.635 | 5.27E-05 |
| sp|Q96PC5|MIA2_HUMAN | 160345.7779 | 0.74 | 0.148 | 7.53E-08 |
| sp|Q99685|MGLL_HUMAN | 33450.25557 | 0.92 | 0.195 | 0.01905 |
| sp|Q16630|CPSF6_HUMAN | 59326.47306 | 1.28 | 0.109 | 3.38E-13 |
| sp|Q93096|TP4A1_HUMAN | 20126.34616 | 0.77 | 0.073 | 2.67E-13 |
| sp|Q9HBB8|CDHR5_HUMAN | 88549.8127 | 0.72 | 0.342 | 0.0002165 |
| sp|Q5JTH9|RRP12_HUMAN | 145018.6195 | 0.69 | 0.084 | 1.08E-13 |
| sp|Q7Z4N2|TRPM1_HUMAN | 183412.7449 | 8.12 | 2.406 | 2.20E-16 |
| sp|Q8WV24|PHLA1_HUMAN | 45483.23115 | 0.79 | 0.15 | 6.58E-07 |
| sp|Q9NQX4|MYO5C_HUMAN | 203975.6867 | 0.82 | 0.19 | 0.0001439 |
| sp|Q9ULH7|MRTFB_HUMAN | 118264.4338 | 1.48 | 0.257 | 1.42E-10 |
| sp|Q9HBW9|AGRL4_HUMAN | 79623.48978 | 1.2 | 0.262 | 0.0008867 |
| sp|Q96FQ6|S10AG_HUMAN | 11832.97976 | 0.96 | 0.15 | 0.09765 |
| sp|Q8IWV8|UBR2_HUMAN | 203811.038 | 0.68 | 0.096 | 1.11E-12 |
| sp|P31513|FMO3_HUMAN | 60603.60444 | 0.56 | 0.121 | 7.28E-13 |
| sp|Q15633|TRBP2_HUMAN | 39623.88983 | 1.1 | 0.138 | 0.00328 |
| sp|Q9Y376|CAB39_HUMAN | 39996.79533 | 0.37 | 0.06 | 2.20E-16 |
| sp|Q9NXX6|NSE4A_HUMAN | 44370.20465 | 1.11 | 0.209 | 0.04642 |
| sp|Q16836|HCDH_HUMAN | 34310.9471 | 1.81 | 0.642 | 2.40E-07 |
| sp|Q9H1Z4|WDR13_HUMAN | 54271.41546 | 1.1 | 0.129 | 0.001263 |
| sp|Q9BXL7|CAR11_HUMAN | 134493.778 | 1.58 | 0.618 | 0.0004619 |
| sp|Q9H8W4|PKHF2_HUMAN | 28446.08276 | 0.9 | 0.103 | 4.71E-05 |
| sp|Q9UN36|NDRG2_HUMAN | 41096.30206 | 0.69 | 0.245 | 5.08E-06 |
| sp|P06396|GELS_HUMAN | 86025.32904 | 1.3 | 0.319 | 3.98E-05 |
| sp|Q31612|1B73_HUMAN | 40791.06867 | 1.35 | 1.583 | 0.4386 |
| sp|P61981|1433G_HUMAN | 28437.96658 | 1.55 | 0.201 | 1.48E-14 |
| sp|O95218|ZRAB2_HUMAN | 37820.18281 | 1.68 | 0.832 | 3.36E-05 |
| sp|Q7Z5L9|I2BP2_HUMAN | 61709.92621 | 1 | 0.137 | 0.7033 |
| sp|Q96B97|SH3K1_HUMAN | 73234.75638 | 1.49 | 0.247 | 7.25E-11 |
| sp|O43674|NDUB5_HUMAN | 21718.54741 | 0.77 | 0.219 | 4.24E-05 |
| sp|P14618|KPYM_HUMAN | 58452.22747 | 1.14 | 0.156 | 0.0001549 |
| sp|Q5W111|SPRY7_HUMAN | 22089.81888 | 0.99 | 0.075 | 0.4876 |
| sp|P63241|IF5A1_HUMAN | 17031.47986 | 1.44 | 0.227 | 2.31E-11 |
| sp|P11166|GTR1_HUMAN | 54372.65406 | 0.66 | 0.202 | 8.97E-08 |
| sp|Q9BXF6|RFIP5_HUMAN | 70582.11273 | 1.19 | 0.335 | 0.01073 |
| sp|Q5VUA4|ZN318_HUMAN | 252593.6474 | 1.11 | 0.213 | 0.04255 |
| sp|O94759|TRPM2_HUMAN | 172724.7748 | 1.33 | 0.399 | 0.0001554 |
| sp|Q9NZJ5|E2AK3_HUMAN | 126145.8655 | 0.64 | 0.1 | 9.40E-14 |
| sp|O60315|ZEB2_HUMAN | 137825.6353 | 0.81 | 0.117 | 9.94E-08 |
| sp|Q13510|ASAH1_HUMAN | 45069.00752 | 0.72 | 0.203 | 3.57E-07 |
| sp|Q86XZ4|SPAS2_HUMAN | 59831.68019 | 1.37 | 0.169 | 4.91E-12 |
| sp|Q14687|GSE1_HUMAN | 136347.7583 | 1 | 0.269 | 0.5442 |
| sp|Q8IXJ6|SIR2_HUMAN | 43763.66475 | 0.89 | 0.149 | 0.001531 |
| sp|Q9Y3P9|RBGP1_HUMAN | 122896.882 | 0.68 | 0.072 | 1.40E-15 |
| sp|Q00403|TF2B_HUMAN | 35305.82476 | 1.01 | 0.138 | 0.9899 |
| sp|Q9H0U6|RM18_HUMAN | 20773.61411 | 0.79 | 0.166 | 3.62E-06 |
| sp|P46821|MAP1B_HUMAN | 271647.4507 | 1.72 | 0.355 | 1.48E-11 |
| sp|Q9UFW8|CGBP1_HUMAN | 19075.72098 | 0.75 | 0.215 | 4.19E-06 |
| sp|P08319|ADH4_HUMAN | 41090.04799 | 0.98 | 0.392 | 0.2558 |
| sp|Q92890|UFD1_HUMAN | 34745.40391 | 1.26 | 0.155 | 3.43E-09 |
| sp|Q9UDY8|MALT1_HUMAN | 93734.69477 | 0.75 | 0.125 | 5.09E-09 |
| sp|O60264|SMCA5_HUMAN | 122494.6079 | 0.74 | 0.148 | 2.63E-08 |
| sp|Q9UJC5|SH3L2_HUMAN | 12357.23737 | 1.26 | 0.674 | 0.5734 |
| sp|P10909|CLUS_HUMAN | 53013.20993 | 0.92 | 0.231 | 0.02565 |
| sp|Q92932|PTPR2_HUMAN | 111982.3643 | 1.09 | 0.27 | 0.2467 |
| sp|Q9Y608|LRRF2_HUMAN | 82331.36643 | 1.44 | 0.251 | 4.39E-10 |
| sp|P20226|TBP_HUMAN | 37827.51843 | 0.78 | 0.117 | 8.11E-09 |
| sp|Q92508|PIEZ1_HUMAN | 289837.1535 | 0.92 | 0.15 | 0.009798 |
| sp|P17858|PFKAL_HUMAN | 85744.4497 | 0.61 | 0.15 | 4.90E-10 |
| sp|O75663|TIPRL_HUMAN | 31633.78924 | 0.86 | 0.137 | 1.40E-05 |
| sp|P61960|UFM1_HUMAN | 9150.883514 | 1.25 | 0.344 | 0.003866 |
| sp|Q86Y39|NDUAB_HUMAN | 15052.61141 | 0.36 | 0.153 | 2.18E-11 |
| sp|P01706|LV211_HUMAN | 12789.07376 | 1.88 | 0.6 | 8.09E-09 |
| sp|P46736|BRCC3_HUMAN | 36430.21932 | 1.35 | 0.195 | 2.25E-10 |
| sp|P47974|TISD_HUMAN | 51982.15433 | 0.9 | 0.113 | 0.0002417 |
| sp|P16035|TIMP2_HUMAN | 25049.37354 | 1 | 0.264 | 0.5653 |
| sp|P23219|PGH1_HUMAN | 69364.96643 | 0.74 | 0.158 | 1.54E-07 |
| sp|O95477|ABCA1_HUMAN | 256344.9254 | 0.67 | 0.129 | 1.79E-10 |
| sp|Q14677|EPN4_HUMAN | 68254.80548 | 0.99 | 0.216 | 0.4176 |
| sp|P18124|RL7_HUMAN | 29246.2086 | 0.65 | 0.107 | 1.37E-12 |
| sp|Q6P996|PDXD1_HUMAN | 87546.6357 | 0.64 | 0.2 | 4.16E-08 |
| sp|O15264|MK13_HUMAN | 42215.70771 | 0.76 | 0.216 | 6.68E-06 |
| sp|Q7Z2E3|APTX_HUMAN | 41095.26056 | 0.91 | 0.111 | 0.000602 |
| sp|Q8TF42|UBS3B_HUMAN | 73771.83391 | 0.78 | 0.187 | 4.66E-06 |
| sp|P12931|SRC_HUMAN | 60292.17503 | 0.52 | 0.17 | 7.51E-11 |
| sp|P01700|LV147_HUMAN | 12428.95937 | 1.53 | 0.306 | 1.60E-10 |
| sp|P07333|CSF1R_HUMAN | 109094.5703 | 1.96 | 0.947 | 2.26E-07 |
| sp|Q9BY42|RTF2_HUMAN | 34531.43989 | 1.47 | 0.508 | 2.00E-05 |
| sp|Q14940|SL9A5_HUMAN | 99729.13244 | 0.67 | 0.245 | 1.63E-06 |
| sp|Q9NSK0|KLC4_HUMAN | 69035.78694 | 1 | 0.211 | 0.6315 |
| sp|P05413|FABPH_HUMAN | 14887.73781 | 1.48 | 0.395 | 8.91E-07 |
| sp|O75533|SF3B1_HUMAN | 146461.3619 | 0.7 | 0.137 | 1.04E-09 |
| sp|P09601|HMOX1_HUMAN | 32780.01709 | 1.18 | 0.305 | 0.02192 |
| sp|O43716|GATC_HUMAN | 15172.69957 | 1.24 | 0.166 | 9.77E-08 |
| sp|Q9BSU1|CP070_HUMAN | 48046.08695 | 0.94 | 0.165 | 0.05239 |
| sp|Q495B1|AKD1A_HUMAN | 57895.58765 | 1.97 | 1.215 | 0.001169 |
| sp|P61244|MAX_HUMAN | 18245.9027 | 1.5 | 0.267 | 4.06E-11 |
| sp|Q9UBN7|HDAC6_HUMAN | 132914.196 | 0.95 | 0.142 | 0.05039 |
| sp|Q8N8A2|ANR44_HUMAN | 109341.9502 | 1.1 | 0.253 | 0.1021 |
| sp|Q9UHY7|ENOPH_HUMAN | 29067.52483 | 1.2 | 0.126 | 8.03E-09 |
| sp|Q8WU79|SMAP2_HUMAN | 47078.90264 | 0.85 | 0.195 | 0.0003575 |
| sp|P27169|PON1_HUMAN | 39859.28197 | 1.22 | 0.444 | 0.0924 |
| sp|P28715|ERCC5_HUMAN | 133749.9513 | 0.95 | 0.198 | 0.07706 |
| sp|P10319|1B58_HUMAN | 40579.13766 | 0.62 | 0.376 | 5.11E-05 |
| sp|P83876|TXN4A_HUMAN | 16871.27971 | 1.04 | 0.168 | 0.4776 |
| sp|Q13231|CHIT1_HUMAN | 52200.55902 | 1.16 | 0.901 | 0.4908 |
| sp|P0C2W1|FBSP1_HUMAN | 31165.4478 | 0.79 | 0.162 | 2.49E-06 |
| sp|P82914|RT15_HUMAN | 29919.46911 | 1.21 | 0.228 | 0.0001427 |
| sp|Q6MZZ7|CAN13_HUMAN | 77313.72331 | 0.8 | 0.271 | 0.001163 |
| sp|P01011|AACT_HUMAN | 47773.59027 | 1.65 | 0.79 | 0.001254 |
| sp|P07203|GPX1_HUMAN | 22342.20542 | 1.33 | 0.119 | 3.20E-14 |
| sp|Q9BYX2|TBD2A_HUMAN | 106242.3665 | 0.54 | 0.206 | 5.43E-09 |
| sp|Q9HD34|LYRM4_HUMAN | 10733.77319 | 1.14 | 0.228 | 0.01474 |
| sp|P11766|ADHX_HUMAN | 40535.69081 | 0.57 | 0.134 | 2.77E-12 |
| sp|O60934|NBN_HUMAN | 85572.24031 | 1.42 | 0.24 | 1.05E-09 |
| sp|P51798|CLCN7_HUMAN | 89117.62211 | 0.58 | 0.143 | 2.78E-11 |
| sp|Q16787|LAMA3_HUMAN | 375633.5055 | 1.2 | 0.346 | 0.0227 |
| sp|Q92575|UBXN4_HUMAN | 57009.78141 | 0.67 | 0.238 | 4.36E-07 |
| sp|O00562|PITM1_HUMAN | 135885.4944 | 1.02 | 0.135 | 0.7148 |
| sp|Q8NFT2|STEA2_HUMAN | 56286.7486 | 0.65 | 0.296 | 1.09E-05 |
| sp|Q96M96|FGD4_HUMAN | 87579.80759 | 0.82 | 0.11 | 1.18E-07 |
| sp|P00734|THRB_HUMAN | 71456.67158 | 1.23 | 0.464 | 0.1022 |
| sp|P19823|ITIH2_HUMAN | 106834.7705 | 1.26 | 0.351 | 0.001676 |
| sp|Q9HCY8|S10AE_HUMAN | 11807.8145 | 0.78 | 0.335 | 0.0007117 |
| sp|P55011|S12A2_HUMAN | 132029.9575 | 0.57 | 0.148 | 3.43E-12 |
| sp|A6NMZ7|CO6A6_HUMAN | 248654.3659 | 1.74 | 0.632 | 5.00E-08 |
| sp|O14818|PSA7_HUMAN | 28022.6685 | 0.9 | 0.126 | 0.0002835 |
| sp|P07951|TPM2_HUMAN | 32926.59932 | 0.88 | 0.453 | 0.01937 |
| sp|Q96MH6|TMM68_HUMAN | 37667.64098 | 0.66 | 0.168 | 6.91E-09 |
| sp|O60239|3BP5_HUMAN | 50660.93356 | 1.4 | 0.554 | 0.007894 |
| sp|O95926|SYF2_HUMAN | 28743.81866 | 1.54 | 0.519 | 1.17E-05 |
| sp|Q9NZR1|TMOD2_HUMAN | 39552.67487 | 1.36 | 0.614 | 0.008294 |
| sp|Q7L8J4|3BP5L_HUMAN | 43682.96203 | 1.39 | 0.177 | 3.06E-12 |
| sp|O75607|NPM3_HUMAN | 19598.49799 | 1.82 | 0.762 | 4.21E-05 |
| sp|Q16621|NFE2_HUMAN | 41656.9154 | 1.17 | 0.426 | 0.1959 |
| sp|O15061|SYNEM_HUMAN | 172987.3987 | 0.66 | 0.282 | 2.53E-05 |
| sp|P48739|PIPNB_HUMAN | 31787.0122 | 0.95 | 0.075 | 0.001332 |
| sp|P02452|CO1A1_HUMAN | 139864.9463 | 3.11 | 1.707 | 1.97E-10 |
| sp|Q08257|QOR_HUMAN | 35337.59736 | 0.86 | 0.209 | 0.001842 |
| sp|Q9Y3D8|KAD6_HUMAN | 20201.9602 | 1.25 | 0.315 | 0.0005082 |
| sp|Q9H8S9|MOB1A_HUMAN | 25216.66219 | 0.82 | 0.272 | 0.00107 |
| sp|P01857|IGHG1_HUMAN | 36578.34964 | 1.24 | 0.368 | 0.01044 |
| sp|Q9UHJ9|PGAP2_HUMAN | 29990.11996 | 0.69 | 0.097 | 1.81E-12 |
| sp|P32856|STX2_HUMAN | 33359.22862 | 0.89 | 0.104 | 2.54E-05 |
| sp|Q9NUP1|BL1S4_HUMAN | 23489.51867 | 1.62 | 0.255 | 2.34E-13 |
| sp|O14647|CHD2_HUMAN | 212165.0412 | 1.01 | 0.104 | 0.7034 |
| sp|P09455|RET1_HUMAN | 15992.9513 | 1.29 | 0.559 | 0.07539 |
| sp|Q7Z7H8|RM10_HUMAN | 29473.75765 | 0.98 | 0.134 | 0.3234 |
| sp|Q96IP4|TET5A_HUMAN | 50471.76022 | 0.94 | 0.344 | 0.1075 |
| sp|P51170|SCNNG_HUMAN | 75458.53174 | 1.12 | 0.095 | 1.61E-06 |
| sp|Q9Y5K6|CD2AP_HUMAN | 71617.40133 | 1.27 | 0.347 | 0.001328 |
| sp|Q9UK99|FBX3_HUMAN | 55362.73734 | 0.47 | 0.095 | 9.38E-16 |
| sp|Q9Y4D8|HECD4_HUMAN | 444692.1115 | 0.59 | 0.121 | 1.74E-12 |
| sp|Q93099|HGD_HUMAN | 50597.83915 | 0.65 | 0.122 | 8.10E-11 |
| sp|Q9NZB2|F120A_HUMAN | 122989.9871 | 0.84 | 0.122 | 1.97E-06 |
| sp|Q9UBQ6|EXTL2_HUMAN | 37708.3739 | 1.1 | 0.099 | 3.88E-05 |
| sp|Q96D96|HVCN1_HUMAN | 31758.98377 | 0.94 | 0.232 | 0.07345 |
| sp|Q13503|MED21_HUMAN | 15707.74081 | 1.22 | 0.168 | 7.05E-07 |
| sp|O14965|AURKA_HUMAN | 46161.98262 | 0.55 | 0.182 | 8.06E-10 |
| sp|Q9NZU5|LMCD1_HUMAN | 41985.51465 | 1.11 | 0.201 | 0.02243 |
| sp|P25815|S100P_HUMAN | 10432.23337 | 0.79 | 0.623 | 0.003046 |
| sp|O75363|BCAS1_HUMAN | 61938.56693 | 0.57 | 0.28 | 1.98E-06 |
| sp|Q9H4A5|GLP3L_HUMAN | 32785.9878 | 0.87 | 0.346 | 0.01555 |
| sp|Q9H5K3|SG196_HUMAN | 40462.11554 | 0.94 | 0.097 | 0.004406 |
| sp|Q9BXJ4|C1QT3_HUMAN | 27243.90114 | 1.3 | 0.408 | 0.005802 |
| sp|O00566|MPP10_HUMAN | 78911.71623 | 1.1 | 0.204 | 0.03486 |
| sp|P26885|FKBP2_HUMAN | 15792.37077 | 1.08 | 0.242 | 0.2469 |
| sp|O60232|SSA27_HUMAN | 21898.84748 | 1.53 | 0.361 | 1.39E-08 |
| sp|O43251|RFOX2_HUMAN | 41330.34126 | 1.3 | 0.192 | 9.66E-09 |
| sp|Q96AB3|ISOC2_HUMAN | 22589.9387 | 0.76 | 0.133 | 1.36E-08 |
| sp|Q16513|PKN2_HUMAN | 112801.5549 | 0.85 | 0.081 | 2.90E-09 |
| sp|Q13033|STRN3_HUMAN | 87536.45242 | 1.28 | 0.139 | 1.53E-10 |
| sp|Q9C0J8|WDR33_HUMAN | 146180.4258 | 0.83 | 0.142 | 5.22E-06 |
| sp|P35542|SAA4_HUMAN | 14833.30085 | 0.98 | 0.369 | 0.2556 |
| sp|Q8N8R7|AL14E_HUMAN | 30212.77979 | 1.11 | 0.143 | 0.0008637 |
| sp|Q9NZZ3|CHMP5_HUMAN | 24594.36531 | 1.49 | 0.342 | 1.98E-08 |
| sp|Q9NQC3|RTN4_HUMAN | 130232.3277 | 0.74 | 0.148 | 3.50E-08 |
| sp|P62072|TIM10_HUMAN | 10536.01858 | 1.45 | 0.321 | 5.62E-08 |
| sp|P54922|ADPRH_HUMAN | 39691.42519 | 1.2 | 0.186 | 1.69E-05 |
| sp|Q92917|GPKOW_HUMAN | 52406.6394 | 1.14 | 0.359 | 0.2215 |
| sp|Q70E73|RAPH1_HUMAN | 135780.5034 | 1.09 | 0.141 | 0.004724 |
| sp|O43865|SAHH2_HUMAN | 59978.83945 | 0.93 | 0.129 | 0.005304 |
| sp|P05534|1A24_HUMAN | 40930.12816 | 1.78 | 0.917 | 0.000289 |
| sp|Q96KB5|TOPK_HUMAN | 36386.10542 | 1.22 | 0.242 | 0.0001155 |
| sp|Q8IUX7|AEBP1_HUMAN | 131570.2643 | 1.85 | 0.536 | 6.07E-10 |
| sp|Q92945|FUBP2_HUMAN | 73337.12089 | 1.42 | 0.073 | 2.20E-16 |
| sp|Q7KYR7|BT2A1_HUMAN | 60374.78219 | 0.89 | 0.149 | 0.000595 |
| sp|P00915|CAH1_HUMAN | 28891.39907 | 1.09 | 0.352 | 0.6273 |
| sp|Q8WXH0|SYNE2_HUMAN | 801798.7293 | 0.95 | 0.225 | 0.1242 |
| sp|P18085|ARF4_HUMAN | 20593.72547 | 0.66 | 0.089 | 3.02E-14 |
| sp|Q8TAL6|FIBIN_HUMAN | 24525.02053 | 1.09 | 0.374 | 0.725 |
| sp|P09496|CLCA_HUMAN | 27156.06438 | 1.08 | 0.164 | 0.06106 |
| sp|Q9Y6W3|CAN7_HUMAN | 93317.10578 | 0.82 | 0.065 | 4.16E-12 |
| sp|Q9C0B7|TNG6_HUMAN | 122192.5485 | 0.82 | 0.108 | 3.73E-08 |
| sp|Q9HAV7|GRPE1_HUMAN | 24473.99375 | 1.11 | 0.198 | 0.02715 |
| sp|P50914|RL14_HUMAN | 23513.05012 | 1.36 | 0.266 | 1.83E-07 |
| sp|Q9Y4F9|RIPR2_HUMAN | 119396.1231 | 1.03 | 0.284 | 0.9899 |
| sp|Q07820|MCL1_HUMAN | 37410.35567 | 1.47 | 0.392 | 6.05E-07 |
| sp|P98175|RBM10_HUMAN | 103793.4778 | 1.42 | 0.346 | 6.60E-07 |
| sp|Q99613|EIF3C_HUMAN | 105943.9671 | 0.77 | 0.13 | 2.97E-08 |
| sp|Q9P270|SLAI2_HUMAN | 62715.35236 | 1.68 | 0.495 | 5.47E-08 |
| sp|P07478|TRY2_HUMAN | 26909.19545 | 0.5 | 0.257 | 5.30E-07 |
| sp|Q96AE4|FUBP1_HUMAN | 67671.52541 | 1.41 | 0.192 | 2.49E-11 |
| sp|P49407|ARRB1_HUMAN | 47417.42782 | 0.95 | 0.177 | 0.08771 |
| sp|P15170|ERF3A_HUMAN | 56329.52248 | 0.84 | 0.175 | 0.0001451 |
| sp|P23193|TCEA1_HUMAN | 34386.18748 | 1.23 | 0.273 | 0.0004369 |
| sp|O15232|MATN3_HUMAN | 54361.33733 | 1.54 | 0.391 | 3.61E-08 |
| sp|O60507|TPST1_HUMAN | 42542.11987 | 1.06 | 0.167 | 0.1796 |
| sp|Q6AWC2|WWC2_HUMAN | 134817.2831 | 1.09 | 0.264 | 0.2146 |
| sp|Q15019|SEPT2_HUMAN | 41671.32563 | 1.34 | 0.173 | 7.07E-11 |
| sp|Q8IWB1|IPRI_HUMAN | 62913.76393 | 0.82 | 0.12 | 3.80E-07 |
| sp|Q13087|PDIA2_HUMAN | 58493.81494 | 0.62 | 0.337 | 7.48E-05 |
| sp|Q8WXF1|PSPC1_HUMAN | 58802.22701 | 1.27 | 0.151 | 9.18E-10 |
| sp|Q9NP80|PLPL8_HUMAN | 89201.31812 | 0.61 | 0.092 | 1.94E-14 |
| sp|P41091|IF2G_HUMAN | 51629.4222 | 0.62 | 0.11 | 6.47E-13 |
| sp|A8K855|EFCB7_HUMAN | 73000.422 | 0.95 | 0.287 | 0.1641 |
| sp|Q9NRK6|ABCBA_HUMAN | 79479.07916 | 0.62 | 0.112 | 4.74E-13 |
| sp|P00736|C1R_HUMAN | 81588.3833 | 1.14 | 0.328 | 0.1438 |
| sp|Q9NVM4|ANM7_HUMAN | 79644.99452 | 1.05 | 0.123 | 0.0797 |
| sp|O00560|SDCB1_HUMAN | 32576.97284 | 0.95 | 0.175 | 0.07455 |
| sp|P28161|GSTM2_HUMAN | 25881.03792 | 1.01 | 0.404 | 0.4469 |
| sp|Q8TF76|HASP_HUMAN | 89619.32159 | 3.17 | 1.6 | 1.58E-10 |
| sp|Q9Y2L1|RRP44_HUMAN | 109999.3229 | 0.93 | 0.132 | 0.007828 |
| sp|O00339|MATN2_HUMAN | 110626.3253 | 1.03 | 0.562 | 0.3904 |
| sp|Q16625|OCLN_HUMAN | 59487.19045 | 0.67 | 0.391 | 7.49E-05 |
| sp|Q9UGM5|FETUB_HUMAN | 42865.27461 | 1.01 | 0.39 | 0.4808 |
| sp|Q96L93|KI16B_HUMAN | 152469.7519 | 1.01 | 0.092 | 0.644 |
| sp|Q9NRP0|OSTC_HUMAN | 16913.86675 | 0.64 | 0.114 | 1.89E-12 |
| sp|P02656|APOC3_HUMAN | 10827.48843 | 1.38 | 0.539 | 0.007801 |
| sp|Q9H078|CLPB_HUMAN | 79175.18575 | 1.01 | 0.087 | 0.8031 |
| sp|Q8NA72|POC5_HUMAN | 63692.99879 | 0.92 | 0.154 | 0.004685 |
| sp|Q9H1K0|RBNS5_HUMAN | 89594.8108 | 1.18 | 0.222 | 0.000879 |
| sp|Q01432|AMPD3_HUMAN | 89250.81663 | 0.72 | 0.179 | 3.64E-07 |
| sp|Q86YR5|GPSM1_HUMAN | 75073.37912 | 0.91 | 0.102 | 0.0001076 |
| sp|O14908|GIPC1_HUMAN | 36122.7227 | 1.09 | 0.248 | 0.1761 |
| sp|Q9NRZ7|PLCC_HUMAN | 43904.92966 | 0.47 | 0.159 | 8.10E-12 |
| sp|Q96MP8|KCTD7_HUMAN | 33548.65415 | 1.23 | 0.141 | 4.86E-09 |
| sp|Q9BSJ2|GCP2_HUMAN | 103078.1293 | 0.92 | 0.081 | 2.22E-05 |
| sp|P01701|LV151_HUMAN | 12451.02976 | 1.79 | 0.444 | 8.50E-11 |
| sp|Q96DV4|RM38_HUMAN | 44949.51136 | 1.13 | 0.195 | 0.006296 |
| sp|P07360|CO8G_HUMAN | 22416.59631 | 0.72 | 0.186 | 2.12E-07 |
| sp|Q9H8G2|CAAP1_HUMAN | 38953.04499 | 1.41 | 0.268 | 3.28E-09 |
| sp|Q4L180|FIL1L_HUMAN | 130910.3356 | 1.18 | 0.26 | 0.001967 |
| sp|Q9Y6M1|IF2B2_HUMAN | 66176.6523 | 1.23 | 0.221 | 4.57E-05 |
| sp|O43745|CHP2_HUMAN | 22420.4581 | 1.04 | 0.274 | 0.927 |
| sp|Q5SWX8|ODR4_HUMAN | 51680.00181 | 0.35 | 0.126 | 4.21E-14 |
| sp|Q96RT1|ERBIN_HUMAN | 158923.3966 | 1.32 | 0.2 | 6.58E-09 |
| sp|Q9NX58|LYAR_HUMAN | 44026.02476 | 1.28 | 0.185 | 1.22E-08 |
| sp|Q92879|CELF1_HUMAN | 52411.00469 | 1.19 | 0.237 | 0.0006992 |
| sp|A2RRP1|NBAS_HUMAN | 271689.9399 | 0.79 | 0.098 | 1.73E-09 |
| sp|Q5VWQ0|RSBN1_HUMAN | 90909.824 | 0.87 | 0.265 | 0.008867 |
| sp|P40818|UBP8_HUMAN | 128395.3715 | 0.84 | 0.089 | 1.09E-08 |
| sp|Q9ULT0|TTC7A_HUMAN | 96960.42291 | 1 | 0.121 | 0.7418 |
| sp|I1YAP6|TRI77_HUMAN | 53936.66298 | 7.02 | 3.221 | 2.73E-13 |
| sp|P49207|RL34_HUMAN | 13494.54755 | 1.59 | 0.335 | 3.48E-10 |
| sp|Q9Y2U9|KLDC2_HUMAN | 46678.51295 | 1.11 | 0.257 | 0.09858 |
| sp|O95347|SMC2_HUMAN | 136067.0093 | 0.81 | 0.331 | 0.001201 |
| sp|P16989|YBOX3_HUMAN | 40048.01526 | 1.8 | 0.234 | 2.20E-16 |
| sp|P62829|RL23_HUMAN | 14952.09391 | 1.21 | 0.2 | 2.04E-05 |
| sp|Q9GZX9|TWSG1_HUMAN | 26349.80075 | 0.95 | 0.16 | 0.07265 |
| sp|Q9BPX3|CND3_HUMAN | 115327.4585 | 0.91 | 0.234 | 0.01535 |
| sp|Q14137|BOP1_HUMAN | 84243.48272 | 1 | 0.145 | 0.7915 |
| sp|Q8NI77|KI18A_HUMAN | 103395.6122 | 1.18 | 0.341 | 0.02671 |
| sp|P25685|DNJB1_HUMAN | 38173.42305 | 1.33 | 0.265 | 3.70E-07 |
| sp|P52951|GBX2_HUMAN | 37420.94209 | 0.73 | 0.155 | 2.98E-08 |
| sp|Q12872|SFSWA_HUMAN | 105138.8283 | 1.07 | 0.105 | 0.004727 |
| sp|P37108|SRP14_HUMAN | 14656.84932 | 1.29 | 0.235 | 8.06E-07 |
| sp|Q8NBJ7|SUMF2_HUMAN | 33917.84233 | 1.07 | 0.184 | 0.1407 |
| sp|Q2TB90|HKDC1_HUMAN | 103771.9407 | 0.96 | 0.297 | 0.1917 |
| sp|P09693|CD3G_HUMAN | 20666.55724 | 1.25 | 0.314 | 0.0005405 |
| sp|O00743|PPP6_HUMAN | 35787.73021 | 0.81 | 0.176 | 3.82E-05 |
| sp|Q9H9L4|KANL2_HUMAN | 56072.82691 | 1.16 | 0.195 | 0.0005518 |
| sp|P10643|CO7_HUMAN | 96632.48061 | 0.84 | 0.294 | 0.001946 |
| sp|Q8IWR0|Z3H7A_HUMAN | 112330.1624 | 0.98 | 0.09 | 0.2497 |
| sp|Q15120|PDK3_HUMAN | 47062.11934 | 0.62 | 0.123 | 3.33E-11 |
| sp|P61163|ACTZ_HUMAN | 42682.93791 | 0.64 | 0.152 | 1.60E-09 |
| sp|Q9HC84|MUC5B_HUMAN | 611565.9896 | 1.57 | 1.484 | 0.2404 |
| sp|Q96NY8|NECT4_HUMAN | 55914.96264 | 0.89 | 0.203 | 0.003953 |
| sp|P52790|HXK3_HUMAN | 100597.7357 | 0.66 | 0.272 | 3.44E-06 |
| sp|P55198|AF17_HUMAN | 113442.3141 | 1.5 | 0.277 | 1.12E-10 |
| sp|P42785|PCP_HUMAN | 56259.10141 | 0.98 | 0.185 | 0.298 |
| sp|Q96EL3|RM53_HUMAN | 12252.24294 | 1.2 | 0.297 | 0.003628 |
| sp|P11216|PYGB_HUMAN | 97300.77151 | 0.5 | 0.124 | 4.71E-13 |
| sp|Q9BX70|BTBD2_HUMAN | 56562.34203 | 1.36 | 0.335 | 1.07E-05 |
| sp|Q9H5N1|RABE2_HUMAN | 63942.29655 | 1.35 | 0.114 | 5.28E-15 |
| sp|Q7Z404|TMC4_HUMAN | 80051.43153 | 0.46 | 0.152 | 5.02E-12 |
| sp|P78330|SERB_HUMAN | 25144.95744 | 1.06 | 0.134 | 0.06373 |
| sp|P55010|IF5_HUMAN | 49629.96631 | 1.11 | 0.08 | 1.36E-07 |
| sp|Q4G176|ACSF3_HUMAN | 64698.50777 | 0.8 | 0.105 | 4.17E-09 |
| sp|P11047|LAMC1_HUMAN | 183172.6856 | 0.96 | 0.357 | 0.1363 |
| sp|Q9H7C4|SYNCI_HUMAN | 55475.70946 | 1.5 | 0.425 | 1.74E-06 |
| sp|O75144|ICOSL_HUMAN | 33822.86352 | 1.02 | 0.289 | 0.6555 |
| sp|O60701|UGDH_HUMAN | 55655.49482 | 0.99 | 0.26 | 0.3885 |
| sp|P12236|ADT3_HUMAN | 33055.26437 | 0.4 | 0.122 | 1.22E-13 |
| sp|Q8TCB0|IFI44_HUMAN | 51125.18837 | 0.86 | 0.436 | 0.0234 |
| sp|Q8IY37|DHX37_HUMAN | 130528.9115 | 0.89 | 0.234 | 0.009697 |
| sp|Q12789|TF3C1_HUMAN | 241044.3983 | 0.93 | 0.088 | 0.0004579 |
| sp|P35573|GDE_HUMAN | 176800.6676 | 0.73 | 0.074 | 4.60E-14 |
| sp|P31946|1433B_HUMAN | 28160.86301 | 1.17 | 0.166 | 1.62E-05 |
| sp|P78537|BL1S1_HUMAN | 17290.86941 | 1.75 | 0.308 | 2.07E-13 |
| sp|Q92973|TNPO1_HUMAN | 103753.3291 | 0.5 | 0.069 | 2.20E-16 |
| sp|O43396|TXNL1_HUMAN | 32611.86791 | 1.33 | 0.164 | 8.59E-11 |
| sp|O43390|HNRPR_HUMAN | 71166.34497 | 1.29 | 0.122 | 2.15E-12 |
| sp|Q99959|PKP2_HUMAN | 97849.99207 | 0.86 | 0.149 | 0.0001385 |
| sp|Q9HA64|KT3K_HUMAN | 34600.3129 | 0.89 | 0.183 | 0.002531 |
| sp|Q6UXD5|SE6L2_HUMAN | 99019.80968 | 0.87 | 0.238 | 0.00174 |
| sp|A0A0C4DH68|KV224_HUMAN | 13166.66975 | 1.86 | 0.874 | 6.25E-05 |
| sp|Q6P1A2|MBOA5_HUMAN | 56493.2779 | 0.36 | 0.115 | 9.19E-13 |
| sp|Q96IY4|CBPB2_HUMAN | 48945.64579 | 0.7 | 0.198 | 8.28E-07 |
| sp|Q9UNK4|PA2GD_HUMAN | 17372.08786 | 1.15 | 0.206 | 0.002387 |
| sp|Q9C0B0|UNK_HUMAN | 89778.37959 | 1.32 | 0.242 | 1.50E-07 |
| sp|P50454|SERPH_HUMAN | 46507.20959 | 2.05 | 0.626 | 1.02E-10 |
| sp|Q53GQ0|DHB12_HUMAN | 34398.2243 | 0.51 | 0.14 | 5.55E-13 |
| sp|Q9BRZ2|TRI56_HUMAN | 83129.13721 | 0.91 | 0.168 | 0.008861 |
| sp|O00602|FCN1_HUMAN | 35494.2581 | 0.88 | 0.544 | 0.022 |
| sp|Q12800|TFCP2_HUMAN | 57657.88276 | 1.05 | 0.125 | 0.07435 |
| sp|Q15847|ADIRF_HUMAN | 7831.963687 | 3.84 | 2.722 | 4.03E-08 |
| sp|Q5TFE4|NT5D1_HUMAN | 52421.0876 | 0.72 | 0.188 | 8.60E-07 |
| sp|O00754|MA2B1_HUMAN | 114338.6143 | 0.98 | 0.066 | 0.0565 |
| sp|Q9BV94|EDEM2_HUMAN | 65206.79823 | 0.87 | 0.084 | 6.66E-08 |
| sp|Q8N1A6|CD033_HUMAN | 23605.66302 | 1.12 | 0.18 | 0.002943 |
| sp|Q5VZ89|DEN4C_HUMAN | 214895.5626 | 0.79 | 0.088 | 8.44E-11 |
| sp|Q32P41|TRM5_HUMAN | 58533.36198 | 0.99 | 0.257 | 0.484 |
| sp|O60662|KLH41_HUMAN | 68774.19256 | 0.79 | 0.12 | 1.83E-08 |
| sp|P23469|PTPRE_HUMAN | 81313.25886 | 0.76 | 0.166 | 5.28E-07 |
| sp|O96033|MOC2A_HUMAN | 9788.21581 | 1.1 | 0.155 | 0.00479 |
| sp|Q9H4A6|GOLP3_HUMAN | 34056.5998 | 0.66 | 0.171 | 2.13E-09 |
| sp|P00749|UROK_HUMAN | 49883.25515 | 0.72 | 0.41 | 0.002836 |
| sp|Q9UPT5|EXOC7_HUMAN | 83711.24302 | 1.15 | 0.109 | 1.03E-07 |
| sp|O95613|PCNT_HUMAN | 380581.5475 | 1.23 | 0.169 | 4.49E-08 |
| sp|P25090|FPR2_HUMAN | 39376.72042 | 0.89 | 0.229 | 0.007806 |
| sp|Q06033|ITIH3_HUMAN | 100053.6301 | 1.37 | 0.295 | 2.25E-07 |
| sp|Q9Y547|IFT25_HUMAN | 16383.15042 | 1.02 | 0.265 | 0.8233 |
| sp|Q8TB72|PUM2_HUMAN | 114697.0584 | 1.08 | 0.1 | 0.001416 |
| sp|Q8WWM7|ATX2L_HUMAN | 113570.6383 | 1.39 | 0.203 | 1.60E-10 |
| sp|Q86TM6|SYVN1_HUMAN | 68135.50051 | 0.63 | 0.153 | 9.56E-10 |
| sp|Q7RTV0|PHF5A_HUMAN | 13120.23029 | 1.01 | 0.14 | 0.91 |
| sp|P33992|MCM5_HUMAN | 83013.44659 | 1.01 | 0.393 | 0.4147 |
| sp|Q8TDJ6|DMXL2_HUMAN | 342944.2663 | 0.81 | 0.159 | 6.55E-06 |
| sp|P05452|TETN_HUMAN | 22903.41329 | 1.32 | 0.344 | 0.000149 |
| sp|Q8IVL6|P3H3_HUMAN | 82565.92131 | 1.55 | 0.459 | 1.42E-06 |
| sp|Q05932|FOLC_HUMAN | 65348.54858 | 1.25 | 0.21 | 5.37E-06 |
| sp|O00116|ADAS_HUMAN | 73646.04023 | 0.64 | 0.146 | 2.79E-10 |
| sp|Q15067|ACOX1_HUMAN | 74871.36963 | 0.76 | 0.169 | 2.09E-06 |
| sp|P00813|ADA_HUMAN | 41005.75309 | 1.76 | 0.25 | 3.07E-16 |
| sp|Q15784|NDF2_HUMAN | 41716.45698 | 0.63 | 0.189 | 7.18E-09 |
| sp|Q9P2D3|HTR5B_HUMAN | 226821.3476 | 0.71 | 0.082 | 1.23E-13 |
| sp|O95336|6PGL_HUMAN | 27796.64828 | 1.19 | 0.138 | 2.06E-07 |
| sp|Q12959|DLG1_HUMAN | 100660.3292 | 1.14 | 0.172 | 0.0007448 |
| sp|Q01740|FMO1_HUMAN | 60880.95982 | 1.1 | 0.396 | 0.5662 |
| sp|A0A0B4J1X5|HV374_HUMAN | 12984.42404 | 1.04 | 0.323 | 0.9749 |
| sp|P11177|ODPB_HUMAN | 39532.15085 | 1.18 | 0.215 | 0.0007921 |
| sp|Q9Y5S2|MRCKB_HUMAN | 196170.5497 | 1.08 | 0.156 | 0.02407 |
| sp|Q9Y2C4|EXOG_HUMAN | 41326.00847 | 1.44 | 0.358 | 2.36E-07 |
| sp|Q9ULU4|PKCB1_HUMAN | 133188.2952 | 1.1 | 0.089 | 1.33E-05 |
| sp|O60783|RT14_HUMAN | 15225.04933 | 1.17 | 0.288 | 0.017 |
| sp|Q96I15|SCLY_HUMAN | 48385.70579 | 1.52 | 0.207 | 4.69E-14 |
| sp|Q9H1P3|OSBL2_HUMAN | 55661.46504 | 0.52 | 0.093 | 9.12E-16 |
| sp|Q99541|PLIN2_HUMAN | 48255.51624 | 2.04 | 1.334 | 0.0003639 |
| sp|Q14165|MLEC_HUMAN | 32366.63825 | 0.53 | 0.091 | 2.20E-16 |
| sp|Q8TDB8|GTR14_HUMAN | 56835.67722 | 0.81 | 0.254 | 0.0004599 |
| sp|O94913|PCF11_HUMAN | 173610.4918 | 1.31 | 0.339 | 0.0001048 |
| sp|Q8TF72|SHRM3_HUMAN | 218302.9136 | 0.97 | 0.151 | 0.1491 |
| sp|Q9UHB7|AFF4_HUMAN | 127762.8979 | 1.6 | 0.242 | 1.19E-13 |
| sp|P20718|GRAH_HUMAN | 27678.52751 | 1.37 | 0.456 | 8.29E-05 |
| sp|Q96EQ0|SGTB_HUMAN | 33675.36121 | 1.45 | 0.256 | 2.52E-10 |
| sp|P05387|RLA2_HUMAN | 11639.83676 | 1.45 | 0.245 | 1.63E-10 |
| sp|Q9UHW9|S12A6_HUMAN | 128599.647 | 0.94 | 0.219 | 0.08143 |
| sp|Q9UI10|EI2BD_HUMAN | 58016.51159 | 0.87 | 0.188 | 0.0009837 |
| sp|Q9BYV8|CEP41_HUMAN | 41552.94966 | 1.61 | 0.243 | 4.58E-14 |
| sp|Q5JS54|PSMG4_HUMAN | 13804.98 | 0.64 | 0.079 | 6.89E-16 |
| sp|Q07507|DERM_HUMAN | 24541.00394 | 1.64 | 0.891 | 0.000406 |
| sp|Q8WUZ0|BCL7C_HUMAN | 23492.87019 | 1.5 | 0.261 | 1.81E-11 |
| sp|Q9BZE9|ASPC1_HUMAN | 60641.00595 | 1.18 | 0.141 | 7.59E-07 |
| sp|Q5JPH6|SYEM_HUMAN | 59089.77896 | 0.72 | 0.228 | 7.85E-06 |
| sp|Q5HYI8|RABL3_HUMAN | 26559.59083 | 1.33 | 0.153 | 1.25E-11 |
| sp|O94925|GLSK_HUMAN | 74251.21501 | 0.79 | 0.147 | 3.34E-07 |
| sp|Q9Y639|NPTN_HUMAN | 44683.61831 | 0.82 | 0.08 | 4.19E-10 |
| sp|Q75QN2|INT8_HUMAN | 114480.2044 | 1.21 | 0.267 | 0.000641 |
| sp|O00268|TAF4_HUMAN | 110313.9836 | 1.1 | 0.163 | 0.01139 |
| sp|O00400|ACATN_HUMAN | 61364.94652 | 0.72 | 0.114 | 1.00E-10 |
| sp|P35251|RFC1_HUMAN | 128670.2081 | 1.39 | 0.241 | 1.75E-09 |
| sp|Q92889|XPF_HUMAN | 105314.6271 | 0.91 | 0.132 | 0.001481 |
| sp|Q8NBM4|UBAC2_HUMAN | 39319.30891 | 1.08 | 0.25 | 0.3203 |
| sp|Q14005|IL16_HUMAN | 142958.3327 | 1.57 | 0.525 | 1.05E-06 |
| sp|Q13620|CUL4B_HUMAN | 104468.4132 | 0.68 | 0.08 | 1.15E-14 |
| sp|Q9BWJ5|SF3B5_HUMAN | 10224.81787 | 1.31 | 0.173 | 3.75E-10 |
| sp|Q8WV22|NSE1_HUMAN | 31273.81104 | 0.89 | 0.073 | 3.03E-07 |
| sp|Q02878|RL6_HUMAN | 32746.63975 | 0.9 | 0.138 | 0.0008906 |
| sp|A0A0C4DH38|HV551_HUMAN | 12819.35381 | 1.06 | 0.351 | 0.8987 |
| sp|Q00796|DHSO_HUMAN | 38909.03912 | 0.69 | 0.139 | 8.57E-09 |
| sp|Q9Y4B6|DCAF1_HUMAN | 170649.7641 | 1.33 | 0.186 | 1.23E-09 |
| sp|Q9UID3|VPS51_HUMAN | 86882.56495 | 0.65 | 0.094 | 1.24E-13 |
| sp|P62263|RS14_HUMAN | 16415.58665 | 1.2 | 0.061 | 4.55E-15 |
| sp|O14558|HSPB6_HUMAN | 17163.98928 | 1.15 | 0.288 | 0.05031 |
| sp|Q53GA4|PHLA2_HUMAN | 17234.90201 | 0.96 | 0.175 | 0.1129 |
| sp|Q7Z3C6|ATG9A_HUMAN | 95338.37882 | 0.45 | 0.147 | 1.52E-12 |
| sp|Q15628|TRADD_HUMAN | 34549.93002 | 1.04 | 0.199 | 0.5928 |
| sp|P07093|GDN_HUMAN | 44184.27958 | 1.14 | 0.451 | 0.5137 |
| sp|Q68DL7|CR063_HUMAN | 78018.61087 | 1.28 | 0.404 | 0.005877 |
| sp|Q29940|1B59_HUMAN | 40826.25513 | 1.4 | 0.539 | 0.001714 |
| sp|Q9UBB5|MBD2_HUMAN | 43552.02207 | 1.4 | 0.164 | 1.98E-13 |
| sp|Q9NW81|DMAC2_HUMAN | 29686.9289 | 0.89 | 0.187 | 0.003447 |
| sp|Q8TDR0|MIPT3_HUMAN | 78908.21555 | 2.83 | 1.309 | 2.60E-10 |
| sp|P00966|ASSY_HUMAN | 46768.06033 | 1.26 | 0.519 | 0.04005 |
| sp|Q16881|TRXR1_HUMAN | 71813.57114 | 0.73 | 0.063 | 1.04E-15 |
| sp|Q9UKA9|PTBP2_HUMAN | 57550.82777 | 0.78 | 0.242 | 0.0001258 |
| sp|Q76MJ5|ERN2_HUMAN | 103081.8339 | 0.85 | 0.171 | 0.0001673 |
| sp|Q8NI37|PPTC7_HUMAN | 33006.28298 | 1.07 | 0.141 | 0.02727 |
| sp|Q9ULC5|ACSL5_HUMAN | 76836.49718 | 0.7 | 0.174 | 2.63E-08 |
| sp|Q5T0Z8|CF132_HUMAN | 124396.1192 | 0.91 | 0.225 | 0.02398 |
| sp|Q96BR5|COA7_HUMAN | 26415.32427 | 0.86 | 0.167 | 0.0002808 |
| sp|Q5TAH2|SL9C2_HUMAN | 130547.1475 | 1.16 | 0.648 | 0.8819 |
| sp|O00567|NOP56_HUMAN | 66389.85665 | 0.69 | 0.127 | 1.60E-10 |
| sp|Q7Z5L2|R3HCL_HUMAN | 88836.40081 | 1.06 | 0.128 | 0.05908 |
| sp|Q9Y4E6|WDR7_HUMAN | 166139.1812 | 0.83 | 0.094 | 5.99E-09 |
| sp|P49321|NASP_HUMAN | 85453.16911 | 1.47 | 0.411 | 5.08E-06 |
| sp|Q6SZW1|SARM1_HUMAN | 80346.59148 | 0.9 | 0.389 | 0.04205 |
| sp|Q5JTJ3|COA6_HUMAN | 14430.97192 | 1.18 | 0.256 | 0.00417 |
| sp|O75351|VPS4B_HUMAN | 49424.54294 | 0.97 | 0.099 | 0.06951 |
| sp|O14672|ADA10_HUMAN | 86122.44805 | 0.63 | 0.13 | 1.58E-10 |
| sp|Q9UKT5|FBX4_HUMAN | 44489.10739 | 1.04 | 0.223 | 0.6377 |
| sp|Q8WVJ2|NUDC2_HUMAN | 17817.79738 | 1.51 | 0.389 | 5.80E-08 |
| sp|P49760|CLK2_HUMAN | 60491.1455 | 0.72 | 0.13 | 1.57E-09 |
| sp|O75787|RENR_HUMAN | 38965.31349 | 0.64 | 0.11 | 1.07E-12 |
| sp|P36954|RPB9_HUMAN | 15008.80554 | 1.47 | 0.129 | 2.20E-16 |
| sp|P14324|FPPS_HUMAN | 48739.81161 | 0.52 | 0.119 | 1.69E-13 |
| sp|P68400|CSK21_HUMAN | 45210.83137 | 1.04 | 0.1 | 0.08126 |
| sp|P39687|AN32A_HUMAN | 28664.3148 | 1.42 | 0.325 | 8.24E-08 |
| sp|Q8IXM2|BAP18_HUMAN | 17928.32794 | 1.67 | 0.309 | 1.75E-12 |
| sp|P43686|PRS6B_HUMAN | 47432.57122 | 1.35 | 0.078 | 2.20E-16 |
| sp|Q16629|SRSF7_HUMAN | 27560.1007 | 1.3 | 0.101 | 1.43E-14 |
| sp|Q92530|PSMF1_HUMAN | 29951.068 | 1.09 | 0.139 | 0.009512 |
| sp|P39900|MMP12_HUMAN | 54120.19875 | 0.64 | 0.32 | 7.26E-06 |
| sp|Q15124|PGM5_HUMAN | 62737.90359 | 0.49 | 0.183 | 2.01E-10 |
| sp|Q96H55|MYO19_HUMAN | 110758.1689 | 0.59 | 0.099 | 7.54E-14 |
| sp|O75888|TNF13_HUMAN | 27569.14756 | 1.3 | 0.18 | 2.61E-09 |
| sp|P41252|SYIC_HUMAN | 145699.5184 | 0.58 | 0.127 | 3.07E-12 |
| sp|Q9HBY0|NOX3_HUMAN | 65730.33422 | 0.37 | 0.153 | 8.05E-12 |
| sp|Q96C36|P5CR2_HUMAN | 33939.80261 | 0.84 | 0.158 | 3.97E-05 |
| sp|O14879|IFIT3_HUMAN | 56672.78202 | 1.45 | 0.36 | 2.06E-06 |
| sp|P62847|RS24_HUMAN | 15395.42128 | 1.25 | 0.253 | 0.0001157 |
| sp|Q53FZ2|ACSM3_HUMAN | 66662.92988 | 0.76 | 0.436 | 0.001787 |
| sp|P19075|TSN8_HUMAN | 26692.73117 | 0.61 | 0.417 | 2.67E-05 |
| sp|O43768|ENSA_HUMAN | 13362.74027 | 1.44 | 0.275 | 4.47E-09 |
| sp|Q99536|VAT1_HUMAN | 42103.49254 | 0.67 | 0.109 | 2.21E-12 |
| sp|Q7Z392|TPC11_HUMAN | 130433.6363 | 0.65 | 0.09 | 2.98E-14 |
| sp|Q12955|ANK3_HUMAN | 482376.3142 | 0.96 | 0.065 | 0.005303 |
| sp|P60953|CDC42_HUMAN | 21569.13547 | 0.66 | 0.097 | 2.56E-13 |
| sp|P27707|DCK_HUMAN | 30823.18352 | 1.21 | 0.344 | 0.02416 |
| sp|P33241|LSP1_HUMAN | 37379.07935 | 1.39 | 0.292 | 1.64E-07 |
| sp|Q14802|FXYD3_HUMAN | 9466.743873 | 0.96 | 0.14 | 0.07102 |
| sp|Q14244|MAP7_HUMAN | 84097.54892 | 1.08 | 0.362 | 0.715 |
| sp|O95229|ZWINT_HUMAN | 31313.24352 | 1.35 | 0.374 | 0.0001572 |
| sp|Q9NVS9|PNPO_HUMAN | 30292.91552 | 1.17 | 0.221 | 0.001862 |
| sp|P00492|HPRT_HUMAN | 24773.67473 | 0.73 | 0.18 | 2.14E-07 |
| sp|P30520|PURA2_HUMAN | 50446.91398 | 0.62 | 0.136 | 1.37E-11 |
| sp|P04433|KV311_HUMAN | 12663.3363 | 1.51 | 0.464 | 6.62E-06 |
| sp|Q8IXH7|NELFD_HUMAN | 66813.56091 | 0.55 | 0.099 | 1.08E-14 |
| sp|P23368|MAOM_HUMAN | 66011.00631 | 0.71 | 0.199 | 1.78E-06 |
| sp|O94880|PHF14_HUMAN | 101797.1978 | 1.2 | 0.241 | 0.0005029 |
| sp|Q8TEQ8|PIGO_HUMAN | 119516.8957 | 0.69 | 0.113 | 2.59E-11 |
| sp|P08397|HEM3_HUMAN | 39515.68722 | 1.27 | 0.218 | 2.80E-06 |
| sp|Q9NZ63|TLS1_HUMAN | 33706.15556 | 1.61 | 0.403 | 3.93E-09 |
| sp|Q96K49|TM87B_HUMAN | 64160.95606 | 0.64 | 0.173 | 1.25E-09 |
| sp|P22681|CBL_HUMAN | 100862.7399 | 0.85 | 0.119 | 2.96E-06 |
| sp|Q96S19|MTL26_HUMAN | 22830.79492 | 0.95 | 0.165 | 0.07535 |
| sp|Q9H0R3|TM222_HUMAN | 23595.57688 | 0.63 | 0.247 | 4.28E-07 |
| sp|P19532|TFE3_HUMAN | 61522.68136 | 1.21 | 0.15 | 6.28E-08 |
| sp|O60245|PCDH7_HUMAN | 116494.6855 | 0.97 | 0.273 | 0.2064 |
| sp|Q7Z7E8|UB2Q1_HUMAN | 46479.21408 | 0.99 | 0.248 | 0.4629 |
| sp|Q9Y6A9|SPCS1_HUMAN | 11836.12598 | 0.56 | 0.154 | 7.14E-11 |
| sp|Q9P0U4|CXXC1_HUMAN | 77526.87392 | 1.13 | 0.097 | 2.61E-07 |
| sp|P50053|KHK_HUMAN | 32997.6346 | 1.1 | 0.167 | 0.008664 |
| sp|P01112|RASH_HUMAN | 21608.66932 | 0.97 | 0.338 | 0.2046 |
| sp|Q3KQU3|MA7D1_HUMAN | 93088.21739 | 1.54 | 0.135 | 2.20E-16 |
| sp|P31941|ABC3A_HUMAN | 23321.29498 | 0.83 | 0.088 | 4.90E-09 |
| sp|Q9Y4D1|DAAM1_HUMAN | 123947.8082 | 0.76 | 0.13 | 7.92E-09 |
| sp|Q9BVG4|PBDC1_HUMAN | 26136.71824 | 1.47 | 0.28 | 8.00E-10 |
| sp|P09669|COX6C_HUMAN | 8757.679314 | 0.69 | 0.278 | 1.15E-05 |
| sp|Q92990|GLMN_HUMAN | 68887.83182 | 0.71 | 0.094 | 8.75E-13 |
| sp|Q8IV08|PLD3_HUMAN | 55108.66999 | 0.81 | 0.118 | 9.50E-08 |
| sp|Q8IYB8|SUV3_HUMAN | 88772.71246 | 0.68 | 0.171 | 5.92E-08 |
| sp|Q15274|NADC_HUMAN | 31150.22265 | 1.14 | 0.364 | 0.2287 |
| sp|O60869|EDF1_HUMAN | 16340.86825 | 1.41 | 0.431 | 0.000115 |
| sp|Q15393|SF3B3_HUMAN | 136557.138 | 0.63 | 0.128 | 1.36E-11 |
| sp|O15075|DCLK1_HUMAN | 82725.1614 | 1.45 | 0.546 | 4.38E-05 |
| sp|O60635|TSN1_HUMAN | 26892.37596 | 0.45 | 0.187 | 1.43E-10 |
| sp|O00423|EMAL1_HUMAN | 90756.06984 | 0.63 | 0.167 | 9.46E-10 |
| sp|P14550|AK1A1_HUMAN | 36873.97401 | 1.17 | 0.274 | 0.01534 |
| sp|P12107|COBA1_HUMAN | 181562.9094 | 1.58 | 0.539 | 1.61E-05 |
| sp|Q96G25|MED8_HUMAN | 29157.87042 | 1.26 | 0.157 | 3.50E-09 |
| sp|P0C7P0|CISD3_HUMAN | 14530.53026 | 0.74 | 0.298 | 0.0001442 |
| sp|Q6IPR1|ETFR1_HUMAN | 10838.85185 | 1.18 | 0.255 | 0.002972 |
| sp|Q9HCC0|MCCB_HUMAN | 61789.59032 | 0.79 | 0.202 | 3.40E-05 |
| sp|P35237|SPB6_HUMAN | 42918.17816 | 1.3 | 0.237 | 5.96E-07 |
| sp|Q96BH1|RNF25_HUMAN | 51795.90053 | 1.15 | 0.245 | 0.0115 |
| sp|O00139|KIF2A_HUMAN | 80570.88407 | 1.26 | 0.226 | 1.68E-06 |
| sp|Q9H089|LSG1_HUMAN | 75844.60322 | 1.15 | 0.109 | 1.18E-07 |
| sp|P49593|PPM1F_HUMAN | 49953.16143 | 1.2 | 0.101 | 8.40E-11 |
| sp|Q9H9L3|I20L2_HUMAN | 39396.65813 | 1.03 | 0.097 | 0.1524 |
| sp|Q8IUD2|RB6I2_HUMAN | 128217.6593 | 1.66 | 0.333 | 3.34E-12 |
| sp|Q9UJ14|GGT7_HUMAN | 70803.94785 | 0.86 | 0.102 | 3.92E-07 |
| sp|Q6NUK4|REEP3_HUMAN | 29227.22788 | 0.52 | 0.081 | 2.20E-16 |
| sp|P02766|TTHY_HUMAN | 15973.07943 | 1.15 | 0.297 | 0.05334 |
| sp|Q9BT43|RPC7L_HUMAN | 25300.42772 | 1.72 | 0.416 | 1.36E-09 |
| sp|Q8N1G0|ZN687_HUMAN | 131880.2143 | 0.81 | 0.177 | 1.00E-05 |
| sp|Q53H96|P5CR3_HUMAN | 29140.08934 | 1.42 | 0.243 | 1.09E-09 |
| sp|P05556|ITB1_HUMAN | 91646.24493 | 0.65 | 0.092 | 1.17E-13 |
| sp|P45985|MP2K4_HUMAN | 44697.54823 | 0.97 | 0.124 | 0.1508 |
| sp|Q9UK45|LSM7_HUMAN | 11691.03919 | 1.54 | 0.253 | 6.72E-13 |
| sp|Q9P2E5|CHPF2_HUMAN | 86617.51287 | 0.75 | 0.168 | 4.56E-07 |
| sp|Q8NFF5|FAD1_HUMAN | 65947.52801 | 0.73 | 0.158 | 1.83E-08 |
| sp|P47914|RL29_HUMAN | 17780.07202 | 1.41 | 0.35 | 8.32E-07 |
| sp|Q8TAP9|MPLKI_HUMAN | 19174.07168 | 1.18 | 0.234 | 0.001277 |
| sp|Q8IWB9|TEX2_HUMAN | 125833.7102 | 1.02 | 0.154 | 0.8713 |
| sp|P06744|G6PI_HUMAN | 63317.31885 | 0.52 | 0.053 | 2.20E-16 |
| sp|Q96EY4|TMA16_HUMAN | 24059.50178 | 1.24 | 0.327 | 0.004795 |
| sp|Q8IX04|UEVLD_HUMAN | 52498.13031 | 0.63 | 0.095 | 3.69E-14 |
| sp|O75165|DJC13_HUMAN | 256515.2867 | 0.74 | 0.068 | 1.43E-14 |
| sp|Q96C10|DHX58_HUMAN | 77458.53496 | 0.98 | 0.32 | 0.2997 |
| sp|Q69YN4|VIR_HUMAN | 203533.9876 | 0.9 | 0.143 | 0.001169 |
| sp|Q16352|AINX_HUMAN | 55510.44704 | 1.04 | 0.196 | 0.6223 |
| sp|P61956|SUMO2_HUMAN | 10903.37423 | 1.86 | 0.32 | 2.32E-15 |
| sp|Q5JSH3|WDR44_HUMAN | 101855.9042 | 1.02 | 0.094 | 0.5253 |
| sp|Q9NT62|ATG3_HUMAN | 36279.61253 | 1.16 | 0.203 | 0.0004934 |
| sp|Q8N0X4|CLYBL_HUMAN | 37545.96237 | 1.09 | 0.279 | 0.3931 |
| sp|Q8N163|CCAR2_HUMAN | 103447.0005 | 1.1 | 0.152 | 0.00277 |
| sp|P22105|TENX_HUMAN | 465095.8405 | 1.47 | 0.636 | 0.0001286 |
| sp|Q9HD67|MYO10_HUMAN | 239175.7288 | 0.8 | 0.218 | 0.0005107 |
| sp|P30039|PBLD_HUMAN | 32032.32008 | 0.99 | 0.574 | 0.1685 |
| sp|P37198|NUP62_HUMAN | 53375.51981 | 1.26 | 0.145 | 2.86E-09 |
| sp|Q96KP1|EXOC2_HUMAN | 105065.8363 | 0.67 | 0.071 | 2.88E-16 |
| sp|Q12926|ELAV2_HUMAN | 39746.15474 | 1.07 | 0.617 | 0.5551 |
| sp|Q96BT7|ALKB8_HUMAN | 75998.25484 | 1.11 | 0.21 | 0.01976 |
| sp|P06729|CD2_HUMAN | 39747.74566 | 0.97 | 0.229 | 0.2422 |
| sp|Q9NZW5|MPP6_HUMAN | 61345.8466 | 1.42 | 0.302 | 2.06E-07 |
| sp|Q9H1A3|METL9_HUMAN | 36836.82839 | 1.37 | 0.254 | 2.31E-08 |
| sp|Q86V48|LUZP1_HUMAN | 120754.3442 | 1.28 | 0.102 | 5.48E-14 |
| sp|P23786|CPT2_HUMAN | 74225.5801 | 0.68 | 0.226 | 2.32E-06 |
| sp|O43709|BUD23_HUMAN | 32184.19897 | 1.21 | 0.315 | 0.008133 |
| sp|Q99985|SEM3C_HUMAN | 86446.08566 | 1.09 | 0.127 | 0.002879 |
| sp|Q14689|DIP2A_HUMAN | 172352.9448 | 0.61 | 0.109 | 9.83E-13 |
| sp|P43403|ZAP70_HUMAN | 70778.34354 | 1.2 | 0.346 | 0.01335 |
| sp|P05019|IGF1_HUMAN | 22436.16947 | 0.93 | 0.192 | 0.03509 |
| sp|P50851|LRBA_HUMAN | 321568.0289 | 0.71 | 0.183 | 5.54E-07 |
| sp|Q8N954|GPT11_HUMAN | 33523.64816 | 1.29 | 0.208 | 9.37E-08 |
| sp|Q9BZ67|FRMD8_HUMAN | 51681.37869 | 1.29 | 0.104 | 6.75E-14 |
| sp|P22415|USF1_HUMAN | 33613.66604 | 1.06 | 0.086 | 0.003602 |
| sp|Q5EBL4|RIPL1_HUMAN | 47118.0099 | 1.61 | 0.268 | 2.34E-12 |
| sp|P84090|ERH_HUMAN | 12404.05639 | 1.47 | 0.274 | 9.24E-10 |
| sp|P42858|HD_HUMAN | 351356.2741 | 0.77 | 0.09 | 4.19E-11 |
| sp|Q9H8Y5|ANKZ1_HUMAN | 81543.48568 | 1.03 | 0.375 | 0.6592 |
| sp|Q9Y624|JAM1_HUMAN | 32943.63007 | 0.63 | 0.135 | 1.96E-11 |
| sp|Q96RK0|CIC_HUMAN | 164271.3105 | 1.61 | 0.714 | 7.20E-06 |
| sp|P04222|1C03_HUMAN | 41216.41715 | 0.93 | 0.17 | 0.01728 |
| sp|O60941|DTNB_HUMAN | 72146.93051 | 0.97 | 0.323 | 0.1954 |
| sp|Q96IW7|SC22A_HUMAN | 35533.72637 | 0.79 | 0.148 | 8.76E-07 |
| sp|P51854|TKTL1_HUMAN | 66185.81702 | 0.91 | 0.629 | 0.03541 |
| sp|Q13287|NMI_HUMAN | 35187.99788 | 1.44 | 0.427 | 3.13E-06 |
| sp|P53007|TXTP_HUMAN | 34315.11962 | 0.5 | 0.14 | 2.57E-12 |
| sp|Q9UBB4|ATX10_HUMAN | 54178.08355 | 0.68 | 0.133 | 1.15E-10 |
| sp|P10316|1A69_HUMAN | 41218.27352 | 0.83 | 0.483 | 0.004807 |
| sp|P35637|FUS_HUMAN | 53603.86653 | 1.24 | 0.334 | 0.001597 |
| sp|P08254|MMP3_HUMAN | 54210.38803 | 0.83 | 0.42 | 0.01904 |
| sp|P14621|ACYP2_HUMAN | 11171.58593 | 1.36 | 0.088 | 2.20E-16 |
| sp|Q9NPQ8|RIC8A_HUMAN | 60166.65625 | 0.9 | 0.07 | 4.51E-07 |
| sp|P30622|CLIP1_HUMAN | 162869.8983 | 1.36 | 0.125 | 1.38E-14 |
| sp|Q16543|CDC37_HUMAN | 44935.18235 | 1.31 | 0.222 | 5.32E-08 |
| sp|O95870|ABHGA_HUMAN | 63812.31271 | 0.58 | 0.09 | 2.40E-15 |
| sp|Q86VP3|PACS2_HUMAN | 98421.69147 | 0.95 | 0.098 | 0.01434 |
| sp|Q9NXE4|NSMA3_HUMAN | 98470.71018 | 0.86 | 0.122 | 5.98E-06 |
| sp|Q9BZZ5|API5_HUMAN | 59292.02981 | 0.91 | 0.241 | 0.01965 |
| sp|Q8TDM6|DLG5_HUMAN | 215427.8291 | 1.28 | 0.298 | 1.75E-05 |
| sp|Q9UK59|DBR1_HUMAN | 62125.4878 | 0.79 | 0.13 | 1.36E-07 |
| sp|P84095|RHOG_HUMAN | 21733.09278 | 0.78 | 0.116 | 8.96E-09 |
| sp|O43291|SPIT2_HUMAN | 28933.08593 | 0.69 | 0.335 | 0.0005457 |
| sp|O43324|MCA3_HUMAN | 19837.35294 | 0.78 | 0.129 | 6.13E-08 |
| sp|P56385|ATP5I_HUMAN | 7910.314936 | 1.11 | 0.157 | 0.002011 |
| sp|Q9NZD4|AHSP_HUMAN | 11814.96985 | 2.23 | 0.697 | 1.01E-10 |
| sp|Q9Y266|NUDC_HUMAN | 38258.13835 | 1.34 | 0.17 | 5.90E-11 |
| sp|A6NEE1|PLHD1_HUMAN | 59433.18568 | 0.85 | 0.221 | 0.001447 |
| sp|Q96B26|EXOS8_HUMAN | 30572.51688 | 1.07 | 0.201 | 0.2207 |
| sp|P03897|NU3M_HUMAN | 13216.06126 | 0.52 | 0.163 | 4.60E-11 |
| sp|P46527|CDN1B_HUMAN | 22269.80451 | 1.33 | 0.282 | 2.09E-06 |
| sp|Q6IA86|ELP2_HUMAN | 94247.53519 | 0.73 | 0.104 | 4.00E-11 |
| sp|P05388|RLA0_HUMAN | 34404.85151 | 0.49 | 0.114 | 3.75E-14 |
| sp|O43818|U3IP2_HUMAN | 52417.74021 | 0.98 | 0.223 | 0.363 |
| sp|Q8TDL5|BPIB1_HUMAN | 52561.52623 | 1.18 | 0.317 | 0.01859 |
| sp|Q96P44|COLA1_HUMAN | 100086.9823 | 1.56 | 0.587 | 8.19E-06 |
| sp|Q8IUH3|RBM45_HUMAN | 53849.92903 | 0.95 | 0.114 | 0.02854 |
| sp|Q5T5C0|STXB5_HUMAN | 129070.4812 | 0.65 | 0.148 | 1.92E-10 |
| sp|Q9H2U2|IPYR2_HUMAN | 38391.16549 | 1.26 | 0.34 | 0.003333 |
| sp|P19367|HXK1_HUMAN | 103542.5574 | 0.55 | 0.113 | 9.79E-14 |
| sp|O43236|SEPT4_HUMAN | 55501.78383 | 1.72 | 0.448 | 2.49E-10 |
| sp|Q6UW68|TM205_HUMAN | 21450.98631 | 0.6 | 0.069 | 2.20E-16 |
| sp|Q9NVR2|INT10_HUMAN | 83191.29094 | 0.7 | 0.02 | 2.20E-16 |
| sp|Q9NUU6|OTULL_HUMAN | 42549.70165 | 0.51 | 0.132 | 2.11E-12 |
| sp|A0A0C4DH25|KVD20_HUMAN | 12603.26621 | 1.39 | 0.39 | 1.25E-05 |
| sp|P98088|MUC5A_HUMAN | 601944.9974 | 0.73 | 0.32 | 0.002948 |
| sp|Q9NV88|INT9_HUMAN | 74775.11454 | 0.54 | 0.155 | 1.02E-10 |
| sp|Q9UN37|VPS4A_HUMAN | 49134.09816 | 1.21 | 0.111 | 2.89E-10 |
| sp|Q7LDG7|GRP2_HUMAN | 70041.65954 | 0.83 | 0.155 | 2.61E-05 |
| sp|P01599|KV117_HUMAN | 12923.41354 | 1.1 | 0.233 | 0.1239 |
| sp|Q15417|CNN3_HUMAN | 36543.83819 | 1.57 | 0.586 | 1.27E-05 |
| sp|O15498|YKT6_HUMAN | 22556.4606 | 0.79 | 0.127 | 4.29E-08 |
| sp|P63313|TYB10_HUMAN | 5004.542395 | 2.64 | 1.006 | 4.88E-12 |
| sp|P46087|NOP2_HUMAN | 89571.23474 | 1.2 | 0.225 | 0.0002834 |
| sp|Q14112|NID2_HUMAN | 153934.3637 | 1.37 | 0.309 | 7.14E-07 |
| sp|Q96FH0|BORC8_HUMAN | 13433.65985 | 1.15 | 0.161 | 6.11E-05 |
| sp|P13796|PLSL_HUMAN | 70796.09561 | 0.93 | 0.304 | 0.07716 |
| sp|Q86WW8|COA5_HUMAN | 8694.190915 | 1.05 | 0.243 | 0.7254 |
| sp|Q96T37|RBM15_HUMAN | 107334.0987 | 1.22 | 0.172 | 9.08E-07 |
| sp|P28676|GRAN_HUMAN | 24204.55121 | 0.74 | 0.223 | 6.04E-06 |
| sp|Q9BV73|CP250_HUMAN | 281861.5699 | 1.3 | 0.091 | 7.45E-16 |
| sp|P37235|HPCL1_HUMAN | 22395.01823 | 0.77 | 0.158 | 7.97E-07 |
| sp|Q9UNH6|SNX7_HUMAN | 45540.66347 | 1.39 | 0.237 | 6.84E-09 |
| sp|Q9Y3D2|MSRB2_HUMAN | 19961.8548 | 1.19 | 0.173 | 2.68E-05 |
| sp|Q86TN4|TRPT1_HUMAN | 27877.63855 | 1.25 | 0.563 | 0.1921 |
| sp|P30530|UFO_HUMAN | 99510.48878 | 1.28 | 0.245 | 1.34E-06 |
| sp|Q9NWS6|F118A_HUMAN | 40738.52094 | 1.08 | 0.362 | 0.8277 |
| sp|Q9BQ13|KCD14_HUMAN | 29782.10991 | 0.59 | 0.31 | 2.53E-05 |
| sp|P68133|ACTS_HUMAN | 42347.97103 | 0.75 | 0.245 | 9.60E-05 |
| sp|Q92922|SMRC1_HUMAN | 123285.3289 | 1.2 | 0.18 | 9.13E-06 |
| sp|Q53S33|BOLA3_HUMAN | 12145.40972 | 0.98 | 0.185 | 0.342 |
| sp|Q9HAC7|SUCHY_HUMAN | 48755.13508 | 1.77 | 2.119 | 0.4495 |
| sp|Q86TP1|PRUN1_HUMAN | 50777.12616 | 0.68 | 0.06 | 2.20E-16 |
| sp|Q8IZ81|ELMD2_HUMAN | 35204.80233 | 0.59 | 0.083 | 1.32E-15 |
| sp|A7E2V4|ZSWM8_HUMAN | 199321.8313 | 0.55 | 0.143 | 8.78E-12 |
| sp|Q8NFQ8|TOIP2_HUMAN | 51442.22491 | 1.1 | 0.206 | 0.05828 |
| sp|P29083|T2EA_HUMAN | 49745.13476 | 1.34 | 0.211 | 2.75E-08 |
| sp|A0PJY2|FEZF1_HUMAN | 53126.73655 | 0.71 | 0.245 | 4.96E-06 |
| sp|Q15276|RABE1_HUMAN | 99610.52383 | 1.49 | 0.225 | 1.48E-12 |
| sp|O94830|DDHD2_HUMAN | 81646.71645 | 1.5 | 0.306 | 3.95E-10 |
| sp|Q96EK5|KBP_HUMAN | 72434.61041 | 0.8 | 0.108 | 6.86E-09 |
| sp|Q15714|T22D1_HUMAN | 109648.9297 | 1.99 | 1.188 | 1.39E-05 |
| sp|Q9Y2I8|WDR37_HUMAN | 55297.78332 | 1.26 | 0.165 | 7.35E-09 |
| sp|P25774|CATS_HUMAN | 38080.5283 | 0.93 | 0.417 | 0.07769 |
| sp|Q9BZE2|PUS3_HUMAN | 56164.12655 | 0.74 | 0.159 | 9.89E-08 |
| sp|P32004|L1CAM_HUMAN | 140866.6497 | 0.85 | 0.13 | 7.60E-06 |
| sp|P17568|NDUB7_HUMAN | 16601.34241 | 1.47 | 0.475 | 1.38E-05 |
| sp|Q330K2|NDUF6_HUMAN | 38533.07509 | 0.49 | 0.095 | 1.35E-15 |
| sp|Q9NXJ5|PGPI_HUMAN | 23561.62418 | 0.6 | 0.113 | 3.66E-13 |
| sp|O60645|EXOC3_HUMAN | 87284.96478 | 0.85 | 0.078 | 1.80E-09 |
| sp|Q86UU1|PHLB1_HUMAN | 152019.6603 | 1.44 | 0.212 | 3.18E-11 |
| sp|Q86XK7|VSIG1_HUMAN | 42223.20357 | 0.58 | 0.36 | 5.94E-05 |
| sp|O94813|SLIT2_HUMAN | 175785.2847 | 1.53 | 0.44 | 9.83E-07 |
| sp|P50579|MAP2_HUMAN | 53695.45335 | 1.12 | 0.231 | 0.03836 |
| sp|P53999|TCP4_HUMAN | 14368.3996 | 1.43 | 0.476 | 0.000196 |
| sp|Q01813|PFKAP_HUMAN | 86435.8645 | 0.66 | 0.149 | 4.36E-10 |
| sp|Q96SZ6|CK5P1_HUMAN | 68482.90105 | 0.9 | 0.117 | 0.0006529 |
| sp|P00558|PGK1_HUMAN | 44967.26647 | 0.96 | 0.127 | 0.08288 |
| sp|Q96P47|AGAP3_HUMAN | 95879.61565 | 0.89 | 0.218 | 0.003608 |
| sp|P16150|LEUK_HUMAN | 40279.25489 | 1.47 | 0.584 | 0.0004482 |
| sp|Q9C0A0|CNTP4_HUMAN | 147160.2259 | 1.54 | 0.347 | 1.49E-09 |
| sp|Q9H3F6|BACD3_HUMAN | 35790.98504 | 0.95 | 0.367 | 0.1206 |
| sp|Q14C86|GAPD1_HUMAN | 166341.0446 | 0.59 | 0.102 | 2.87E-14 |
| sp|P14598|NCF1_HUMAN | 44863.96035 | 0.84 | 0.368 | 0.006498 |
| sp|O95050|INMT_HUMAN | 29481.60591 | 0.56 | 0.246 | 1.33E-08 |
| sp|P16144|ITB4_HUMAN | 205727.0187 | 0.79 | 0.216 | 2.23E-05 |
| sp|A0A075B6K4|LV310_HUMAN | 12586.00696 | 2.06 | 0.86 | 5.70E-09 |
| sp|O60313|OPA1_HUMAN | 112113.0171 | 1.09 | 0.124 | 0.003428 |
| sp|Q8IXL6|FA20C_HUMAN | 66801.74509 | 1.02 | 0.169 | 0.7524 |
| sp|P24001|IL32_HUMAN | 27154.04674 | 1.39 | 0.792 | 0.05138 |
| sp|P05166|PCCB_HUMAN | 58787.97523 | 0.99 | 0.349 | 0.3519 |
| sp|Q14766|LTBP1_HUMAN | 195093.717 | 0.91 | 0.231 | 0.02109 |
| sp|Q9NUQ8|ABCF3_HUMAN | 80076.28851 | 1.1 | 0.106 | 4.64E-05 |
| sp|A6NI79|CCD69_HUMAN | 34928.02811 | 1.27 | 0.212 | 2.90E-07 |
| sp|Q96BQ5|CC127_HUMAN | 30968.06259 | 1.55 | 0.407 | 8.91E-08 |
| sp|Q13541|4EBP1_HUMAN | 12668.13268 | 0.58 | 0.281 | 5.89E-07 |
| sp|Q9H1C3|GL8D2_HUMAN | 40210.74865 | 0.99 | 0.115 | 0.5887 |
| sp|Q6PIU2|NCEH1_HUMAN | 46045.82499 | 0.59 | 0.198 | 5.47E-09 |
| sp|P36402|TCF7_HUMAN | 41711.72333 | 1.26 | 0.192 | 1.20E-07 |
| sp|Q9BTE6|AASD1_HUMAN | 45775.4075 | 1.43 | 0.253 | 9.89E-09 |
| sp|Q14103|HNRPD_HUMAN | 38563.35645 | 1.58 | 0.216 | 1.00E-14 |
| sp|Q3SY17|S2552_HUMAN | 34023.98593 | 0.36 | 0.074 | 2.20E-16 |
| sp|Q9BZ23|PANK2_HUMAN | 63250.40663 | 1 | 0.143 | 0.6969 |
| sp|Q9NYY8|FAKD2_HUMAN | 82361.31706 | 1.03 | 0.279 | 0.9371 |
| sp|Q6P4A8|PLBL1_HUMAN | 63481.28377 | 0.56 | 0.153 | 2.02E-11 |
| sp|Q5VZK9|CARL1_HUMAN | 152698.5335 | 0.76 | 0.126 | 2.88E-09 |
| sp|P18077|RL35A_HUMAN | 12568.78142 | 1.23 | 0.307 | 0.002412 |
| sp|Q16181|SEPT7_HUMAN | 50915.09096 | 1.33 | 0.187 | 3.52E-10 |
| sp|Q13601|KRR1_HUMAN | 43847.73817 | 0.79 | 0.194 | 2.30E-05 |
| sp|Q12851|M4K2_HUMAN | 92449.44511 | 0.86 | 0.243 | 0.001657 |
| sp|Q9H2J4|PDCL3_HUMAN | 27693.11128 | 0.89 | 0.163 | 0.001268 |
| sp|P27987|IP3KB_HUMAN | 103149.4697 | 1.23 | 0.385 | 0.01499 |
| sp|Q16649|NFIL3_HUMAN | 51592.87024 | 0.78 | 0.324 | 0.0002842 |
| sp|P62879|GBB2_HUMAN | 38030.32772 | 0.97 | 0.247 | 0.2615 |
| sp|Q86V81|THOC4_HUMAN | 26853.60949 | 1.15 | 0.103 | 9.91E-08 |
| sp|Q9HBL0|TENS1_HUMAN | 186480.632 | 0.95 | 0.224 | 0.09223 |
| sp|P49336|CDK8_HUMAN | 53574.02075 | 0.85 | 0.141 | 1.26E-05 |
| sp|Q6ZRY4|RBPS2_HUMAN | 22521.43132 | 1 | 0.28 | 0.4707 |
| sp|Q96P11|NSUN5_HUMAN | 47271.53964 | 0.94 | 0.16 | 0.03499 |
| sp|P48637|GSHB_HUMAN | 52505.31279 | 1.14 | 0.144 | 5.80E-05 |
| sp|Q6IN84|MRM1_HUMAN | 39052.1238 | 0.74 | 0.275 | 0.0001506 |
| sp|P05106|ITB3_HUMAN | 90175.60404 | 0.59 | 0.194 | 2.10E-08 |
| sp|Q9Y3F4|STRAP_HUMAN | 38738.09242 | 1.27 | 0.121 | 7.33E-12 |
| sp|O94829|IPO13_HUMAN | 109305.101 | 0.95 | 0.116 | 0.03177 |
| sp|Q92539|LPIN2_HUMAN | 100174.5162 | 0.93 | 0.261 | 0.06429 |
| sp|Q16799|RTN1_HUMAN | 83833.14156 | 1.97 | 0.538 | 2.94E-11 |
| sp|P15151|PVR_HUMAN | 45769.0115 | 0.54 | 0.18 | 9.46E-10 |
| sp|P00846|ATP6_HUMAN | 24782.84971 | 0.62 | 0.164 | 1.99E-09 |
| sp|Q3B8N2|LEG9B_HUMAN | 39958.97719 | 0.67 | 0.056 | 2.20E-16 |
| sp|P12532|KCRU_HUMAN | 47388.34597 | 0.69 | 0.318 | 2.61E-05 |
| sp|P54652|HSP72_HUMAN | 70244.9626 | 0.92 | 0.247 | 0.04528 |
| sp|P49863|GRAK_HUMAN | 29358.90069 | 1.88 | 0.601 | 8.95E-09 |
| sp|Q9BQS8|FYCO1_HUMAN | 168572.0077 | 0.93 | 0.244 | 0.04749 |
| sp|O14975|S27A2_HUMAN | 71047.62079 | 0.5 | 0.202 | 3.25E-10 |
| sp|O60271|JIP4_HUMAN | 146895.2804 | 1.24 | 0.194 | 1.34E-06 |
| sp|O15391|TYY2_HUMAN | 41816.27643 | 1.24 | 0.29 | 0.0005337 |
| sp|Q0ZGT2|NEXN_HUMAN | 80818.76701 | 1.39 | 0.403 | 1.35E-05 |
| sp|P49848|TAF6_HUMAN | 73232.10819 | 0.77 | 0.111 | 3.88E-09 |
| sp|Q8WUY8|NAT14_HUMAN | 21789.70498 | 0.9 | 0.148 | 0.001407 |
| sp|P11217|PYGM_HUMAN | 97468.78505 | 0.58 | 0.124 | 1.95E-12 |
| sp|P55145|MANF_HUMAN | 21124.97302 | 0.94 | 0.134 | 0.02717 |
| sp|Q9H9B4|SFXN1_HUMAN | 35863.44217 | 0.65 | 0.088 | 1.61E-14 |
| sp|Q5SQ64|LY66F_HUMAN | 32938.87251 | 0.77 | 0.311 | 0.000439 |
| sp|Q9H6X4|TM134_HUMAN | 21725.96363 | 1.02 | 0.173 | 0.9659 |
| sp|Q9BXS9|S26A6_HUMAN | 83352.14889 | 0.52 | 0.288 | 3.92E-07 |
| sp|Q9BXB4|OSB11_HUMAN | 84313.89452 | 0.81 | 0.263 | 0.000502 |
| sp|Q8NF50|DOCK8_HUMAN | 240868.3016 | 1 | 0.133 | 0.6729 |
| sp|Q9HB09|B2L12_HUMAN | 37236.4397 | 1.03 | 0.166 | 0.5753 |
| sp|Q14999|CUL7_HUMAN | 192789.9057 | 0.58 | 0.132 | 5.11E-12 |
| sp|Q9BYC9|RM20_HUMAN | 17584.62029 | 0.89 | 0.149 | 0.0008983 |
| sp|Q8NBJ5|GT251_HUMAN | 71914.60137 | 1 | 0.154 | 0.7266 |
| sp|Q9UM73|ALK_HUMAN | 178535.0897 | 1.42 | 0.37 | 7.03E-06 |
| sp|Q9UBQ7|GRHPR_HUMAN | 36026.88237 | 0.72 | 0.105 | 3.89E-11 |
| sp|P27216|ANX13_HUMAN | 35603.25655 | 0.7 | 0.3 | 5.77E-05 |
| sp|Q96CP2|FWCH2_HUMAN | 14650.57666 | 1.65 | 0.652 | 3.20E-06 |
| sp|Q96FJ0|STALP_HUMAN | 50417.66487 | 1.02 | 0.227 | 0.9103 |
| sp|Q01201|RELB_HUMAN | 62818.63882 | 1.12 | 0.245 | 0.04242 |
| sp|P82663|RT25_HUMAN | 20313.33154 | 1.1 | 0.09 | 1.26E-05 |
| sp|P11171|41_HUMAN | 97509.48706 | 0.99 | 0.132 | 0.5047 |
| sp|Q05193|DYN1_HUMAN | 97728.1156 | 1.22 | 0.233 | 2.13E-05 |
| sp|Q5MNZ9|WIPI1_HUMAN | 49251.4107 | 0.81 | 0.256 | 0.0004728 |
| sp|Q9NRR5|UBQL4_HUMAN | 63851.36758 | 1.12 | 0.153 | 0.0005043 |
| sp|Q14232|EI2BA_HUMAN | 33957.84949 | 0.59 | 0.096 | 4.13E-15 |
| sp|Q9BXJ8|T120A_HUMAN | 41021.96666 | 0.42 | 0.122 | 3.62E-14 |
| sp|Q9NQ55|SSF1_HUMAN | 53427.86819 | 1.06 | 0.268 | 0.6827 |
| sp|Q9Y262|EIF3L_HUMAN | 66894.48196 | 0.64 | 0.11 | 1.50E-12 |
| sp|Q7L8W6|DPH6_HUMAN | 30611.44709 | 1.48 | 0.276 | 4.51E-10 |
| sp|P04908|H2A1B_HUMAN | 14108.93938 | 1.7 | 0.576 | 6.89E-08 |
| sp|Q92621|NU205_HUMAN | 230153.1605 | 0.59 | 0.126 | 2.67E-12 |
| sp|P20338|RAB4A_HUMAN | 24641.31203 | 0.55 | 0.094 | 3.54E-15 |
| sp|P22102|PUR2_HUMAN | 108935.4614 | 0.94 | 0.168 | 0.03505 |
| sp|O75390|CISY_HUMAN | 51889.58885 | 0.67 | 0.11 | 7.58E-12 |
| sp|Q96CM8|ACSF2_HUMAN | 68975.10038 | 0.95 | 0.25 | 0.1208 |
| sp|Q9UHE8|STEA1_HUMAN | 39978.48265 | 0.71 | 0.182 | 4.70E-08 |
| sp|Q70CQ2|UBP34_HUMAN | 409714.0881 | 0.66 | 0.237 | 4.73E-07 |
| sp|P27540|ARNT_HUMAN | 87362.49212 | 0.92 | 0.105 | 0.0006618 |
| sp|Q96AX1|VP33A_HUMAN | 67949.33833 | 0.88 | 0.056 | 3.38E-10 |
| sp|Q13438|OS9_HUMAN | 75953.01531 | 1.26 | 0.15 | 2.18E-09 |
| sp|P13611|CSPG2_HUMAN | 374567.2778 | 1.98 | 0.446 | 1.36E-12 |
| sp|Q9NV70|EXOC1_HUMAN | 102754.215 | 0.92 | 0.064 | 3.62E-06 |
| sp|Q00537|CDK17_HUMAN | 59811.73879 | 1.02 | 0.195 | 0.9164 |
| sp|Q8WXA3|RUFY2_HUMAN | 70689.43044 | 0.89 | 0.085 | 1.89E-06 |
| sp|Q8WUJ3|CEMIP_HUMAN | 154422.0217 | 0.79 | 0.289 | 0.001892 |
| sp|Q9Y4P1|ATG4B_HUMAN | 44988.9238 | 1.23 | 0.491 | 0.06055 |
| sp|Q64LD2|WDR25_HUMAN | 60960.20518 | 1.26 | 0.295 | 0.0004283 |
| sp|Q8NEY1|NAV1_HUMAN | 203069.966 | 1.2 | 0.274 | 0.001957 |
| sp|A0FGR9|ESYT3_HUMAN | 101151.598 | 1.26 | 0.324 | 0.0005706 |
| sp|Q13509|TBB3_HUMAN | 50838.41617 | 1.39 | 0.335 | 1.55E-06 |
| sp|Q8TAK5|GABP2_HUMAN | 48601.82646 | 1.02 | 0.162 | 0.7558 |
| sp|O75367|H2AY_HUMAN | 39745.52097 | 0.81 | 0.096 | 1.98E-09 |
| sp|P08F94|PKHD1_HUMAN | 452102.152 | 1.22 | 0.214 | 2.88E-05 |
| sp|Q96AX9|MIB2_HUMAN | 111619.7559 | 1.21 | 0.578 | 0.4914 |
| sp|O95049|ZO3_HUMAN | 101431.3666 | 0.98 | 0.185 | 0.3178 |
| sp|Q92729|PTPRU_HUMAN | 164240.762 | 2.24 | 1.17 | 1.91E-06 |
| sp|Q13618|CUL3_HUMAN | 89425.65971 | 0.61 | 0.056 | 2.20E-16 |
| sp|A4D1P6|WDR91_HUMAN | 84186.06837 | 1.14 | 0.175 | 0.000845 |
| sp|Q96JA1|LRIG1_HUMAN | 120673.8041 | 0.62 | 0.16 | 5.56E-10 |
| sp|Q9NXG6|P4HTM_HUMAN | 57063.37793 | 1.19 | 0.179 | 1.52E-05 |
| sp|Q8NDH3|PEPL1_HUMAN | 56719.80045 | 0.62 | 0.073 | 2.20E-16 |
| sp|Q99707|METH_HUMAN | 141730.9282 | 1.02 | 0.19 | 0.9324 |
| sp|O15144|ARPC2_HUMAN | 34407.5176 | 0.94 | 0.138 | 0.02362 |
| sp|Q5SZK8|FREM2_HUMAN | 352572.8454 | 0.66 | 0.205 | 2.13E-07 |
| sp|Q96TA0|PCDBI_HUMAN | 80837.23333 | 0.89 | 0.327 | 0.02714 |
| sp|Q8WXD2|SCG3_HUMAN | 52954.87076 | 0.36 | 0.192 | 4.96E-09 |
| sp|Q92841|DDX17_HUMAN | 80888.26471 | 1.08 | 0.103 | 0.00141 |
| sp|O14656|TOR1A_HUMAN | 38108.59775 | 0.43 | 0.123 | 8.46E-14 |
| sp|Q14061|COX17_HUMAN | 7234.498624 | 1.19 | 0.221 | 0.0008094 |
| sp|Q10471|GALT2_HUMAN | 65414.73472 | 0.51 | 0.077 | 2.20E-16 |
| sp|Q03013|GSTM4_HUMAN | 25754.89707 | 0.84 | 0.237 | 0.001611 |
| sp|O15321|TM9S1_HUMAN | 69311.5169 | 0.6 | 0.1 | 2.47E-14 |
| sp|O43615|TIM44_HUMAN | 51647.57268 | 0.92 | 0.184 | 0.01643 |
| sp|Q7Z7A1|CNTRL_HUMAN | 269842.066 | 1.16 | 0.329 | 0.1317 |
| sp|P35244|RFA3_HUMAN | 13655.88442 | 1 | 0.089 | 0.9715 |
| sp|O15084|ANR28_HUMAN | 114587.031 | 0.9 | 0.163 | 0.002569 |
| sp|Q8ND30|LIPB2_HUMAN | 99034.52714 | 1.2 | 0.205 | 3.36E-05 |
| sp|Q9GZU8|PIP30_HUMAN | 29104.62247 | 1 | 0.116 | 0.8436 |
| sp|P52732|KIF11_HUMAN | 120093.4099 | 0.74 | 0.208 | 2.17E-06 |
| sp|Q92730|RND1_HUMAN | 26648.4583 | 1.35 | 0.636 | 0.0285 |
| sp|Q9UFN0|NPS3A_HUMAN | 28544.56975 | 0.63 | 0.127 | 6.64E-11 |
| sp|P09497|CLCB_HUMAN | 25271.16223 | 1.34 | 0.345 | 4.56E-05 |
| sp|P08574|CY1_HUMAN | 35723.08801 | 1 | 0.147 | 0.6525 |
| sp|P31751|AKT2_HUMAN | 56114.24352 | 1.05 | 0.111 | 0.0444 |
| sp|Q14807|KIF22_HUMAN | 73597.79082 | 1.46 | 0.329 | 1.03E-07 |
| sp|P56856|CLD18_HUMAN | 28275.92 | 1.76 | 2.329 | 0.5292 |
| sp|Q13098|CSN1_HUMAN | 56053.3704 | 0.63 | 0.073 | 2.20E-16 |
| sp|Q8TEW0|PARD3_HUMAN | 151825.6161 | 1 | 0.117 | 0.8971 |
| sp|Q6IAN0|DRS7B_HUMAN | 35363.72325 | 0.68 | 0.124 | 5.32E-11 |
| sp|P53611|PGTB2_HUMAN | 37566.55349 | 0.65 | 0.172 | 5.70E-09 |
| sp|P55196|AFAD_HUMAN | 207684.3002 | 0.96 | 0.12 | 0.03854 |
| sp|Q13867|BLMH_HUMAN | 53137.24529 | 0.76 | 0.133 | 3.36E-08 |
| sp|Q6R327|RICTR_HUMAN | 194188.5992 | 0.58 | 0.095 | 8.27E-15 |
| sp|Q9H4E7|DEFI6_HUMAN | 74416.52355 | 0.92 | 0.2 | 0.01957 |
| sp|Q8WUW1|BRK1_HUMAN | 8778.474897 | 1.23 | 0.123 | 2.83E-10 |
| sp|O00244|ATOX1_HUMAN | 7549.789017 | 1.24 | 0.243 | 1.92E-05 |
| sp|P55060|XPO2_HUMAN | 111126.5717 | 0.57 | 0.094 | 3.26E-15 |
| sp|P55211|CASP9_HUMAN | 46974.67377 | 0.97 | 0.09 | 0.0592 |
| sp|P11802|CDK4_HUMAN | 33918.47287 | 1.11 | 0.201 | 0.02095 |
| sp|P60709|ACTB_HUMAN | 42033.84839 | 0.94 | 0.128 | 0.01143 |
| sp|P02649|APOE_HUMAN | 36227.78743 | 0.96 | 0.33 | 0.1739 |
| sp|P42771|CDN2A_HUMAN | 16561.38062 | 1.19 | 0.364 | 0.02049 |
| sp|P12235|ADT1_HUMAN | 33253.29153 | 0.59 | 0.236 | 1.04E-07 |
| sp|Q96AJ9|VTI1A_HUMAN | 25184.08238 | 0.98 | 0.123 | 0.2734 |
| sp|Q14318|FKBP8_HUMAN | 44971.82883 | 1.45 | 0.184 | 3.69E-13 |
| sp|O14569|C56D2_HUMAN | 24225.15642 | 1.01 | 0.171 | 0.9234 |
| sp|Q7Z3E5|ARMC9_HUMAN | 92484.23641 | 0.56 | 0.067 | 2.20E-16 |
| sp|Q9BZG1|RAB34_HUMAN | 29464.09111 | 0.53 | 0.093 | 5.56E-16 |
| sp|O43488|ARK72_HUMAN | 40001.86632 | 1.15 | 0.18 | 0.0009565 |
| sp|Q7Z4G1|COMD6_HUMAN | 9727.810096 | 1.21 | 0.18 | 3.93E-06 |
| sp|Q9Y5N5|N6MT1_HUMAN | 23152.87301 | 1.22 | 0.21 | 2.40E-05 |
| sp|Q02809|PLOD1_HUMAN | 84049.59497 | 1.08 | 0.204 | 0.1203 |
| sp|P42566|EPS15_HUMAN | 99204.0168 | 1.55 | 0.103 | 2.20E-16 |
| sp|Q9P003|CNIH4_HUMAN | 16406.36743 | 1 | 0.268 | 0.4906 |
| sp|Q05315|LEG10_HUMAN | 16538.24813 | 0.36 | 0.124 | 2.22E-12 |
| sp|Q8WVM8|SCFD1_HUMAN | 72658.16553 | 1.02 | 0.14 | 0.6047 |
| sp|Q93091|RNAS6_HUMAN | 17736.8627 | 0.84 | 0.188 | 0.0001436 |
| sp|Q8TCU6|PREX1_HUMAN | 188746.8435 | 0.91 | 0.171 | 0.005628 |
| sp|Q8IYJ3|SYTL1_HUMAN | 62086.27813 | 0.89 | 0.351 | 0.0356 |
| sp|Q969V3|NCLN_HUMAN | 63087.63003 | 0.71 | 0.091 | 8.95E-13 |
| sp|Q12841|FSTL1_HUMAN | 36085.27905 | 1.44 | 0.405 | 3.69E-06 |
| sp|Q9NZ43|USE1_HUMAN | 29448.51473 | 0.89 | 0.118 | 0.0001458 |
| sp|Q3SY69|AL1L2_HUMAN | 102347.4665 | 1.1 | 0.229 | 0.07971 |
| sp|P51398|RT29_HUMAN | 45861.75847 | 0.78 | 0.142 | 1.86E-07 |
| sp|Q9UHD9|UBQL2_HUMAN | 65636.60639 | 1.48 | 0.206 | 2.72E-12 |
| sp|P61457|PHS_HUMAN | 12031.0267 | 1.04 | 0.083 | 0.03729 |
| sp|Q9Y2X3|NOP58_HUMAN | 60035.70027 | 0.71 | 0.121 | 6.23E-10 |
| sp|Q86UW9|DTX2_HUMAN | 68154.7233 | 1.01 | 0.361 | 0.4682 |
| sp|P61966|AP1S1_HUMAN | 18930.67903 | 0.7 | 0.144 | 5.33E-09 |
| sp|P49821|NDUV1_HUMAN | 51451.04191 | 0.82 | 0.224 | 0.000492 |
| sp|Q8IV36|HID1_HUMAN | 89297.74535 | 0.48 | 0.204 | 8.72E-10 |
| sp|P27449|VATL_HUMAN | 15707.45 | 0.36 | 0.085 | 2.20E-16 |
| sp|Q53EZ4|CEP55_HUMAN | 54469.33473 | 0.98 | 0.223 | 0.2763 |
| sp|Q9P0J1|PDP1_HUMAN | 61567.76744 | 0.94 | 0.12 | 0.01002 |
| sp|O00635|TRI38_HUMAN | 54617.66791 | 1.1 | 0.152 | 0.002938 |
| sp|Q96A49|SYAP1_HUMAN | 39947.87441 | 1.18 | 0.208 | 0.0005989 |
| sp|Q9BVP2|GNL3_HUMAN | 62449.63179 | 1.37 | 0.256 | 8.94E-08 |
| sp|O43715|TRIA1_HUMAN | 8990.185277 | 1.79 | 0.778 | 1.80E-06 |
| sp|O94911|ABCA8_HUMAN | 180822.2326 | 1.02 | 0.392 | 0.5577 |
| sp|Q92620|PRP16_HUMAN | 141252.3963 | 1.06 | 0.111 | 0.02316 |
| sp|Q6P4Q7|CNNM4_HUMAN | 87104.12254 | 0.94 | 0.433 | 0.09498 |
| sp|Q9Y3Y2|CHTOP_HUMAN | 26362.26885 | 2.6 | 1.257 | 1.00E-08 |
| sp|O60449|LY75_HUMAN | 201814.9338 | 0.87 | 0.137 | 3.76E-05 |
| sp|Q05707|COEA1_HUMAN | 194459.876 | 1.31 | 0.702 | 0.1379 |
| sp|Q9NZA1|CLIC5_HUMAN | 46797.81024 | 1.28 | 0.464 | 0.01872 |
| sp|Q9NX14|NDUBB_HUMAN | 17344.74262 | 0.77 | 0.191 | 9.65E-06 |
| sp|Q92609|TBCD5_HUMAN | 89500.78503 | 0.71 | 0.091 | 9.73E-13 |
| sp|O94768|ST17B_HUMAN | 42869.35917 | 1.42 | 0.437 | 2.35E-05 |
| sp|Q9UN70|PCDGK_HUMAN | 101282.5981 | 1.26 | 0.363 | 0.002562 |
| sp|P62736|ACTA_HUMAN | 42362.94554 | 0.58 | 0.237 | 7.48E-07 |
| sp|O76076|WISP2_HUMAN | 28442.35931 | 0.96 | 0.26 | 0.1892 |
| sp|P61962|DCAF7_HUMAN | 39510.28798 | 1.32 | 0.149 | 1.00E-11 |
| sp|O43657|TSN6_HUMAN | 28154.29491 | 0.53 | 0.33 | 6.25E-07 |
| sp|P31040|SDHA_HUMAN | 73653.66599 | 1.19 | 0.243 | 0.001597 |
| sp|P28845|DHI1_HUMAN | 32589.70597 | 0.53 | 0.276 | 7.18E-08 |
| sp|A8TX70|CO6A5_HUMAN | 291778.4392 | 1.19 | 0.159 | 1.59E-06 |
| sp|Q9UER7|DAXX_HUMAN | 82046.18884 | 1.41 | 0.136 | 5.51E-15 |
| sp|Q13162|PRDX4_HUMAN | 30730.88781 | 1.35 | 0.269 | 1.58E-07 |
| sp|P40938|RFC3_HUMAN | 41310.15549 | 0.95 | 0.382 | 0.1108 |
| sp|Q9NP77|SSU72_HUMAN | 22998.12092 | 1.14 | 0.353 | 0.2369 |
| sp|Q53GT1|KLH22_HUMAN | 72629.59192 | 0.7 | 0.16 | 7.73E-09 |
| sp|Q15059|BRD3_HUMAN | 79759.1708 | 1.29 | 0.136 | 1.61E-11 |
| sp|Q8IYI6|EXOC8_HUMAN | 82413.68699 | 0.77 | 0.094 | 7.14E-11 |
| sp|Q9UKR5|ERG28_HUMAN | 15949.5674 | 0.48 | 0.119 | 3.79E-14 |
| sp|Q13356|PPIL2_HUMAN | 59224.76894 | 0.96 | 0.135 | 0.08048 |
| sp|Q9H2K8|TAOK3_HUMAN | 105777.8877 | 1.16 | 0.166 | 9.49E-05 |
| sp|O75638|CTAG2_HUMAN | 21229.51573 | 0.92 | 0.373 | 0.06319 |
| sp|Q6ZN55|ZN574_HUMAN | 101157.0148 | 1.03 | 0.193 | 0.7036 |
| sp|P10398|ARAF_HUMAN | 68322.55801 | 0.72 | 0.071 | 3.55E-15 |
| sp|A0A0A0MS15|HV349_HUMAN | 13200.58536 | 1.21 | 0.371 | 0.03666 |
| sp|Q8IWL3|HSC20_HUMAN | 27728.83986 | 1.12 | 0.178 | 0.001575 |
| sp|Q03188|CENPC_HUMAN | 107377.7457 | 0.98 | 0.207 | 0.3842 |
| sp|O14832|PAHX_HUMAN | 38894.70917 | 0.81 | 0.237 | 0.0001863 |
| sp|O14925|TIM23_HUMAN | 22082.04516 | 0.61 | 0.122 | 5.84E-12 |
| sp|P46063|RECQ1_HUMAN | 74418.36992 | 0.81 | 0.118 | 5.27E-08 |
| sp|Q9NXC5|MIO_HUMAN | 100213.5526 | 0.77 | 0.126 | 2.07E-08 |
| sp|Q02318|CP27A_HUMAN | 60577.39895 | 0.73 | 0.201 | 8.48E-06 |
| sp|Q13625|ASPP2_HUMAN | 126204.485 | 0.94 | 0.184 | 0.06205 |
| sp|Q03692|COAA1_HUMAN | 66155.77617 | 1.24 | 0.309 | 0.0007016 |
| sp|Q99547|MPH6_HUMAN | 19050.59179 | 1.29 | 0.173 | 3.56E-09 |
| sp|Q5JU69|TOR2A_HUMAN | 36129.64184 | 1.24 | 0.201 | 2.71E-06 |
| sp|Q5MNZ6|WIPI3_HUMAN | 38877.36343 | 0.82 | 0.105 | 1.21E-08 |
| sp|P18074|ERCC2_HUMAN | 87634.36284 | 0.82 | 0.159 | 6.76E-06 |
| sp|B7ZAQ6|GPHRA_HUMAN | 53148.98062 | 0.57 | 0.129 | 6.55E-13 |
| sp|Q9BQP7|MGME1_HUMAN | 39777.12902 | 0.89 | 0.142 | 0.0003691 |
| sp|P35858|ALS_HUMAN | 66717.06465 | 0.73 | 0.195 | 3.04E-06 |
| sp|O95139|NDUB6_HUMAN | 15461.12398 | 0.56 | 0.135 | 8.61E-12 |
| sp|Q8N556|AFAP1_HUMAN | 81512.07077 | 1.05 | 0.342 | 0.928 |
| sp|Q92551|IP6K1_HUMAN | 50869.76352 | 1 | 0.233 | 0.5897 |
| sp|P02652|APOA2_HUMAN | 11263.92973 | 1.76 | 0.639 | 2.80E-07 |
| sp|Q15464|SHB_HUMAN | 55731.32867 | 1.02 | 0.281 | 0.761 |
| sp|P51884|LUM_HUMAN | 38728.91284 | 2.31 | 1.362 | 1.30E-07 |
| sp|Q9BUJ2|HNRL1_HUMAN | 96231.4952 | 0.93 | 0.109 | 0.002822 |
| sp|O43583|DENR_HUMAN | 22459.21001 | 1.31 | 0.359 | 0.0001635 |
| sp|Q86VP6|CAND1_HUMAN | 137981.2931 | 0.48 | 0.083 | 2.20E-16 |
| sp|O75529|TAF5L_HUMAN | 66779.92684 | 0.98 | 0.148 | 0.3149 |
| sp|P15884|ITF2_HUMAN | 71474.16839 | 1.11 | 0.323 | 0.3548 |
| sp|Q96T58|MINT_HUMAN | 403012.205 | 1.28 | 0.107 | 1.50E-13 |
| sp|Q96GW9|SYMM_HUMAN | 67443.12519 | 0.62 | 0.128 | 3.15E-11 |
| sp|Q9NUP9|LIN7C_HUMAN | 21916.49666 | 1.15 | 0.176 | 0.0002104 |
| sp|Q9NVE5|UBP40_HUMAN | 141619.7606 | 0.99 | 0.103 | 0.5449 |
| sp|O94903|PLPHP_HUMAN | 30591.67339 | 1.14 | 0.103 | 3.66E-07 |
| sp|Q9NVP1|DDX18_HUMAN | 75683.56271 | 0.67 | 0.161 | 3.77E-09 |
| sp|P17947|SPI1_HUMAN | 31159.34196 | 1.19 | 0.279 | 0.004344 |
| sp|P51911|CNN1_HUMAN | 33302.61404 | 0.56 | 0.281 | 5.53E-07 |
| sp|Q92743|HTRA1_HUMAN | 52148.94652 | 1.15 | 0.438 | 0.4565 |
| sp|Q8TDQ1|CLM1_HUMAN | 32638.56483 | 1.08 | 0.564 | 0.6531 |
| sp|Q53GG5|PDLI3_HUMAN | 39816.64198 | 0.97 | 0.181 | 0.2612 |
| sp|P53396|ACLY_HUMAN | 121656.2139 | 0.65 | 0.067 | 2.20E-16 |
| sp|P31942|HNRH3_HUMAN | 36942.09257 | 1.37 | 0.105 | 2.20E-16 |
| sp|Q53H47|SETMR_HUMAN | 79391.34684 | 1.05 | 0.264 | 0.6511 |
| sp|Q96RF0|SNX18_HUMAN | 69346.35337 | 1.03 | 0.169 | 0.498 |
| sp|Q9C0C9|UBE2O_HUMAN | 142612.5463 | 1.04 | 0.102 | 0.1255 |
| sp|Q9UJS0|CMC2_HUMAN | 74509.78307 | 0.51 | 0.116 | 4.67E-14 |
| sp|Q9H2G2|SLK_HUMAN | 143216.4585 | 1.25 | 0.106 | 3.07E-12 |
| sp|Q96F44|TRI11_HUMAN | 53748.9115 | 0.68 | 0.187 | 1.06E-07 |
| sp|P46937|YAP1_HUMAN | 54466.48738 | 1.56 | 0.272 | 1.26E-11 |
| sp|P02144|MYG_HUMAN | 17211.96569 | 1.6 | 0.752 | 0.0003446 |
| sp|P29622|KAIN_HUMAN | 48664.21199 | 0.66 | 0.17 | 2.15E-09 |
| sp|O43278|SPIT1_HUMAN | 60109.86998 | 0.83 | 0.255 | 0.0005323 |
| sp|Q9BRR9|RHG09_HUMAN | 83874.13162 | 1.11 | 0.176 | 0.008179 |
| sp|Q3KP66|INAVA_HUMAN | 73421.68591 | 1.21 | 0.26 | 0.001042 |
| sp|Q2M2I8|AAK1_HUMAN | 104543.8823 | 0.92 | 0.127 | 0.001624 |
| sp|P48061|SDF1_HUMAN | 10925.91779 | 1.07 | 0.235 | 0.3024 |
| sp|P54868|HMCS2_HUMAN | 57094.59841 | 0.63 | 0.366 | 5.30E-06 |
| sp|Q9GZU5|NYX_HUMAN | 52804.61558 | 2.73 | 0.897 | 7.98E-14 |
| sp|Q5VTE6|ANGE2_HUMAN | 63421.46718 | 1.28 | 0.252 | 7.82E-06 |
| sp|A4D263|SPT48_HUMAN | 49965.17201 | 1 | 0.435 | 0.2528 |
| sp|Q9Y3C1|NOP16_HUMAN | 21214.11686 | 1.62 | 0.443 | 9.13E-08 |
| sp|Q9HDC9|APMAP_HUMAN | 46603.90404 | 0.79 | 0.137 | 6.28E-07 |
| sp|P62910|RL32_HUMAN | 15945.83655 | 1.23 | 0.219 | 2.45E-05 |
| sp|P53384|NUBP1_HUMAN | 35121.57798 | 1.22 | 0.219 | 3.32E-05 |
| sp|Q16851|UGPA_HUMAN | 57057.77376 | 0.85 | 0.079 | 4.23E-09 |
| sp|Q9NRQ5|SMCO4_HUMAN | 6715.849297 | 1.12 | 0.438 | 0.6017 |
| sp|O95476|CNEP1_HUMAN | 28455.21644 | 0.68 | 0.132 | 1.81E-10 |
| sp|Q9NZD8|SPG21_HUMAN | 35204.73438 | 0.77 | 0.067 | 6.31E-14 |
| sp|Q8WVT3|TPC12_HUMAN | 79763.28273 | 0.53 | 0.13 | 1.14E-12 |
| sp|Q9UJ83|HACL1_HUMAN | 64410.61902 | 0.63 | 0.325 | 7.34E-06 |
| sp|Q92546|RGP1_HUMAN | 42923.89513 | 1.28 | 0.212 | 2.75E-07 |
| sp|Q9Y291|RT33_HUMAN | 12602.65009 | 1.05 | 0.186 | 0.4433 |
| sp|P46459|NSF_HUMAN | 83037.25857 | 0.81 | 0.125 | 1.55E-07 |
| sp|O95208|EPN2_HUMAN | 68649.35482 | 0.77 | 0.179 | 1.25E-06 |
| sp|O75874|IDHC_HUMAN | 46896.61122 | 1.3 | 0.349 | 0.0002461 |
| sp|P17948|VGFR1_HUMAN | 152536.2046 | 0.88 | 0.144 | 0.0002656 |
| sp|Q13501|SQSTM_HUMAN | 48437.15866 | 1.57 | 0.45 | 2.83E-07 |
| sp|P18564|ITB6_HUMAN | 89225.50484 | 0.99 | 0.186 | 0.5132 |
| sp|Q9NXG2|THUM1_HUMAN | 39671.9431 | 1.15 | 0.258 | 0.01376 |
| sp|P61165|TM258_HUMAN | 9054.780921 | 0.72 | 0.226 | 6.68E-06 |
| sp|P10153|RNAS2_HUMAN | 18837.25869 | 0.6 | 0.16 | 6.58E-09 |
| sp|Q92871|PMM1_HUMAN | 30052.15279 | 1.51 | 0.303 | 1.42E-09 |
| sp|Q8TBE9|NANP_HUMAN | 28347.26319 | 1.02 | 0.138 | 0.594 |
| sp|P24928|RPB1_HUMAN | 218389.5616 | 0.92 | 0.08 | 2.78E-05 |
| sp|P55265|DSRAD_HUMAN | 137160.3339 | 0.95 | 0.198 | 0.08869 |
| sp|Q9H8U3|ZFAN3_HUMAN | 26005.12903 | 1.13 | 0.278 | 0.09233 |
| sp|Q86X52|CHSS1_HUMAN | 92334.9586 | 0.52 | 0.278 | 1.78E-07 |
| sp|Q687X5|STEA4_HUMAN | 52499.74515 | 0.78 | 0.348 | 0.0004019 |
| sp|Q9Y2Y8|PRG3_HUMAN | 26169.30086 | 0.53 | 0.191 | 1.44E-08 |
| sp|Q9H0C8|ILKAP_HUMAN | 43431.97295 | 1.1 | 0.216 | 0.05661 |
| sp|Q9Y248|PSF2_HUMAN | 21453.03129 | 1.27 | 0.434 | 0.01771 |
| sp|Q3L8U1|CHD9_HUMAN | 327967.7723 | 0.9 | 0.12 | 0.0003147 |
| sp|Q9HC38|GLOD4_HUMAN | 35152.39392 | 1.39 | 0.311 | 4.00E-07 |
| sp|P55290|CAD13_HUMAN | 78676.08719 | 1.12 | 0.215 | 0.01634 |
| sp|Q9Y530|OARD1_HUMAN | 17280.77447 | 1.31 | 0.231 | 4.81E-08 |
| sp|Q30154|DRB5_HUMAN | 30304.11373 | 3.22 | 2.908 | 2.62E-06 |
| sp|Q96CB8|INT12_HUMAN | 49215.287 | 1.46 | 0.315 | 1.40E-08 |
| sp|O95182|NDUA7_HUMAN | 12582.64012 | 1.2 | 0.272 | 0.002771 |
| sp|P62714|PP2AB_HUMAN | 36104.5334 | 1.27 | 0.207 | 2.83E-07 |
| sp|Q16610|ECM1_HUMAN | 62213.86563 | 1.11 | 0.269 | 0.137 |
| sp|P98095|FBLN2_HUMAN | 132230.2379 | 1.41 | 0.38 | 8.41E-06 |
| sp|Q08752|PPID_HUMAN | 41119.04002 | 1.22 | 0.169 | 3.39E-07 |
| sp|Q12905|ILF2_HUMAN | 43245.26236 | 0.59 | 0.091 | 3.26E-15 |
| sp|Q9UNZ2|NSF1C_HUMAN | 40530.29824 | 1.32 | 0.247 | 1.85E-06 |
| sp|P51159|RB27A_HUMAN | 25119.34788 | 0.56 | 0.17 | 3.23E-10 |
| sp|Q9NUY8|TBC23_HUMAN | 79109.43429 | 0.76 | 0.122 | 4.29E-09 |
| sp|Q16540|RM23_HUMAN | 17752.11474 | 1.17 | 0.161 | 2.82E-05 |
| sp|P13984|T2FB_HUMAN | 28401.84866 | 1.44 | 0.347 | 6.67E-08 |
| sp|Q8N5B7|CERS5_HUMAN | 46331.35334 | 0.64 | 0.121 | 3.54E-12 |
| sp|Q9UNF0|PACN2_HUMAN | 56028.05519 | 1.08 | 0.092 | 0.0004385 |
| sp|Q5TBA9|FRY_HUMAN | 342062.2589 | 0.59 | 0.134 | 1.03E-11 |
| sp|Q8N1I0|DOCK4_HUMAN | 226869.588 | 0.82 | 0.175 | 2.69E-05 |
| sp|P62937|PPIA_HUMAN | 18210.96058 | 1.21 | 0.098 | 4.09E-11 |
| sp|O00214|LEG8_HUMAN | 35938.59646 | 0.69 | 0.123 | 4.51E-11 |
| sp|Q9Y316|MEMO1_HUMAN | 34092.63097 | 0.97 | 0.163 | 0.2143 |
| sp|Q13425|SNTB2_HUMAN | 58351.18474 | 0.84 | 0.116 | 9.14E-07 |
| sp|Q68DU8|KCD16_HUMAN | 49943.73551 | 1.4 | 0.306 | 1.67E-07 |
| sp|O00763|ACACB_HUMAN | 278342.7525 | 1.68 | 0.467 | 3.83E-09 |
| sp|Q9Y618|NCOR2_HUMAN | 274211.8776 | 1.22 | 0.213 | 1.94E-05 |
| sp|Q03112|MECOM_HUMAN | 139797.6511 | 1.17 | 0.49 | 0.2637 |
| sp|Q00534|CDK6_HUMAN | 37239.14512 | 1.13 | 0.251 | 0.02931 |
| sp|Q86SX6|GLRX5_HUMAN | 16713.57214 | 1.16 | 0.205 | 0.002107 |
| sp|Q9NSK7|CS012_HUMAN | 16314.52312 | 0.87 | 0.311 | 0.02809 |
| sp|P09884|DPOLA_HUMAN | 167556.8846 | 1.44 | 0.387 | 2.33E-06 |
| sp|P09543|CN37_HUMAN | 47929.8305 | 0.78 | 0.085 | 2.73E-11 |
| sp|Q16539|MK14_HUMAN | 41477.08544 | 0.73 | 0.1 | 2.07E-11 |
| sp|Q9NYZ1|TV23B_HUMAN | 23828.14381 | 0.8 | 0.187 | 8.80E-06 |
| sp|Q9NSB2|KRT84_HUMAN | 65923.8814 | 0.46 | 0.22 | 5.30E-09 |
| sp|Q8NFI3|ENASE_HUMAN | 85112.68613 | 0.95 | 0.187 | 0.09619 |
| sp|O60240|PLIN1_HUMAN | 56222.89197 | 2.06 | 0.415 | 7.71E-15 |
| sp|Q9Y6Y0|NS1BP_HUMAN | 72919.31824 | 0.68 | 0.134 | 6.65E-10 |
| sp|P61011|SRP54_HUMAN | 55935.38592 | 0.95 | 0.142 | 0.03814 |
| sp|P11234|RALB_HUMAN | 23489.97353 | 1.12 | 0.188 | 0.008189 |
| sp|O95159|ZFPL1_HUMAN | 34815.44445 | 0.77 | 0.144 | 2.29E-07 |
| sp|Q8N122|RPTOR_HUMAN | 150635.0976 | 0.82 | 0.074 | 4.24E-11 |
| sp|P19971|TYPH_HUMAN | 50305.31234 | 1.11 | 0.323 | 0.207 |
| sp|P51692|STA5B_HUMAN | 90304.75771 | 0.74 | 0.117 | 1.66E-10 |
| sp|P33240|CSTF2_HUMAN | 61016.50427 | 1.08 | 0.144 | 0.01895 |
| sp|Q9H0Q0|FA49A_HUMAN | 37669.87387 | 0.92 | 0.199 | 0.01668 |
| sp|P49354|FNTA_HUMAN | 44477.13106 | 1.58 | 0.189 | 3.34E-16 |
| sp|Q8N884|CGAS_HUMAN | 59443.68087 | 1.11 | 0.236 | 0.07303 |
| sp|P11117|PPAL_HUMAN | 48694.5923 | 0.77 | 0.115 | 3.06E-09 |
| sp|Q53FA7|QORX_HUMAN | 35666.63805 | 0.88 | 0.178 | 0.001485 |
| sp|Q9NPF2|CHSTB_HUMAN | 42023.16453 | 0.76 | 0.197 | 4.21E-06 |
| sp|Q13685|AAMP_HUMAN | 47387.65149 | 1.13 | 0.301 | 0.1195 |
| sp|Q9UG22|GIMA2_HUMAN | 38943.56226 | 0.67 | 0.309 | 1.54E-05 |
| sp|O00506|STK25_HUMAN | 48291.88916 | 1.12 | 0.122 | 4.14E-05 |
| sp|Q8WXA9|SREK1_HUMAN | 59384.02139 | 1.25 | 0.151 | 2.84E-09 |
| sp|Q92520|FAM3C_HUMAN | 24931.67581 | 0.78 | 0.276 | 0.000341 |
| sp|Q14934|NFAC4_HUMAN | 96112.61744 | 1.64 | 0.323 | 1.25E-11 |
| sp|P09668|CATH_HUMAN | 38035.28367 | 1.62 | 0.577 | 1.65E-05 |
| sp|Q5T870|PRR9_HUMAN | 13903.12075 | 4.58 | 3.021 | 1.23E-10 |
| sp|Q9UIA9|XPO7_HUMAN | 125178.481 | 0.67 | 0.106 | 4.69E-12 |
| sp|Q9BX68|HINT2_HUMAN | 17190.25478 | 1.08 | 0.102 | 0.001361 |
| sp|Q14558|KPRA_HUMAN | 39635.72891 | 0.7 | 0.107 | 4.67E-12 |
| sp|Q96EI5|TCAL4_HUMAN | 24727.91583 | 1.44 | 0.5 | 0.0001263 |
| sp|Q8N554|ZN276_HUMAN | 68697.69405 | 0.96 | 0.151 | 0.09533 |
| sp|O95628|CNOT4_HUMAN | 64136.55022 | 1.4 | 0.19 | 7.51E-12 |
| sp|O00161|SNP23_HUMAN | 23663.65319 | 0.82 | 0.164 | 1.49E-05 |
| sp|P32320|CDD_HUMAN | 16669.04088 | 0.82 | 0.391 | 0.003642 |
| sp|Q9Y2D5|AKAP2_HUMAN | 94983.91253 | 1.68 | 0.179 | 2.20E-16 |
| sp|Q14289|FAK2_HUMAN | 117093.663 | 0.91 | 0.116 | 0.0009018 |
| sp|Q8NEY8|PPHLN_HUMAN | 52801.11954 | 1.56 | 0.182 | 2.49E-16 |
| sp|O00483|NDUA4_HUMAN | 9402.921237 | 0.76 | 0.244 | 9.52E-05 |
| sp|Q9BZZ2|SN_HUMAN | 184943.5511 | 1.12 | 0.186 | 0.006296 |
| sp|P80188|NGAL_HUMAN | 22726.76998 | 0.74 | 0.43 | 0.0004565 |
| sp|Q9Y606|TRUA_HUMAN | 47992.11613 | 0.74 | 0.137 | 1.44E-08 |
| sp|P13598|ICAM2_HUMAN | 31129.75368 | 0.68 | 0.16 | 5.12E-09 |
| sp|Q9BUL8|PDC10_HUMAN | 24667.82718 | 0.85 | 0.138 | 8.25E-06 |
| sp|Q13769|THOC5_HUMAN | 79181.203 | 0.64 | 0.43 | 0.00013 |
| sp|Q96ED9|HOOK2_HUMAN | 83707.55215 | 1.37 | 0.251 | 3.03E-08 |
| sp|P09326|CD48_HUMAN | 28103.37149 | 0.94 | 0.261 | 0.07859 |
| sp|P98082|DAB2_HUMAN | 82493.03253 | 1.8 | 0.412 | 7.68E-12 |
| sp|P17812|PYRG1_HUMAN | 67314.23682 | 0.8 | 0.242 | 0.0001121 |
| sp|P01116|RASK_HUMAN | 21909.12556 | 1.11 | 0.146 | 0.0008949 |
| sp|Q02818|NUCB1_HUMAN | 53828.31916 | 1.41 | 0.177 | 6.09E-13 |
| sp|Q9NWB7|IFT57_HUMAN | 49230.68826 | 1.38 | 0.176 | 1.65E-11 |
| sp|Q9P0T7|TMEM9_HUMAN | 21055.58944 | 0.7 | 0.206 | 7.44E-07 |
| sp|Q9H7E9|CH033_HUMAN | 25301.01465 | 1.87 | 0.509 | 2.59E-10 |
| sp|P59666|DEF3_HUMAN | 10562.28668 | 0.72 | 0.48 | 0.0008935 |
| sp|O15438|MRP3_HUMAN | 170641.9088 | 0.66 | 0.213 | 5.08E-07 |
| sp|Q9UKE5|TNIK_HUMAN | 155342.7172 | 1.1 | 0.252 | 0.153 |
| sp|Q9H7B2|RPF2_HUMAN | 35713.23157 | 1.04 | 0.286 | 0.9365 |
| sp|Q5VIR6|VPS53_HUMAN | 80382.30767 | 0.71 | 0.125 | 5.33E-10 |
| sp|Q92665|RT31_HUMAN | 45386.51851 | 1.13 | 0.217 | 0.01583 |
| sp|P01031|CO5_HUMAN | 189878.7675 | 0.62 | 0.248 | 4.01E-07 |
| sp|P42765|THIM_HUMAN | 42335.80702 | 1.56 | 0.283 | 2.77E-11 |
| sp|Q8NDT2|RB15B_HUMAN | 97413.60795 | 1.34 | 0.199 | 7.82E-10 |
| sp|Q5JSL3|DOC11_HUMAN | 240124.2039 | 0.72 | 0.143 | 7.25E-09 |
| sp|Q9BXC9|BBS2_HUMAN | 80429.68517 | 1.02 | 0.318 | 0.7317 |
| sp|Q6ZUM4|RHG27_HUMAN | 99001.324 | 1.24 | 0.249 | 4.83E-05 |
| sp|P50991|TCPD_HUMAN | 58382.94367 | 0.8 | 0.095 | 4.54E-10 |
| sp|P33121|ACSL1_HUMAN | 78901.2444 | 1.01 | 0.122 | 0.7557 |
| sp|P00167|CYB5_HUMAN | 15302.49613 | 2.07 | 0.717 | 1.60E-09 |
| sp|Q2I0M4|LRC26_HUMAN | 35500.82957 | 1.26 | 0.452 | 0.05729 |
| sp|Q9HB20|PKHA3_HUMAN | 34334.67152 | 1.09 | 0.334 | 0.4119 |
| sp|Q8IYJ1|CPNE9_HUMAN | 62263.25596 | 0.56 | 0.173 | 3.18E-10 |
| sp|O43684|BUB3_HUMAN | 37569.34078 | 1.12 | 0.081 | 1.00E-07 |
| sp|Q9UBR2|CATZ_HUMAN | 34512.46869 | 1.61 | 0.402 | 2.22E-09 |
| sp|Q9UBV7|B4GT7_HUMAN | 37820.15523 | 0.62 | 0.055 | 2.20E-16 |
| sp|P33151|CADH5_HUMAN | 87798.08857 | 1.33 | 0.364 | 2.67E-05 |
| sp|O60522|TDRD6_HUMAN | 239827.4539 | 2 | 0.713 | 6.20E-09 |
| sp|Q9P000|COMD9_HUMAN | 22015.51699 | 0.86 | 0.121 | 1.06E-05 |
| sp|Q9GZR7|DDX24_HUMAN | 96880.68325 | 1.05 | 0.126 | 0.1021 |
| sp|Q5VWZ2|LYPL1_HUMAN | 26566.52619 | 0.65 | 0.094 | 5.74E-14 |
| sp|Q15155|NOMO1_HUMAN | 135190.7624 | 0.84 | 0.183 | 0.0001762 |
| sp|P05162|LEG2_HUMAN | 14731.2036 | 0.77 | 0.191 | 6.24E-06 |
| sp|Q15650|TRIP4_HUMAN | 66942.21158 | 1.15 | 0.127 | 2.48E-06 |
| sp|P50281|MMP14_HUMAN | 66176.0752 | 1.12 | 0.358 | 0.3865 |
| sp|Q5SRE5|NU188_HUMAN | 198350.9547 | 0.68 | 0.175 | 6.39E-09 |
| sp|P00746|CFAD_HUMAN | 27511.04756 | 1.28 | 0.268 | 9.43E-06 |
| sp|Q96SU4|OSBL9_HUMAN | 83798.65779 | 0.71 | 0.08 | 3.95E-14 |
| sp|Q7L2E3|DHX30_HUMAN | 134919.8989 | 0.72 | 0.073 | 2.47E-14 |
| sp|Q9GZR2|REXO4_HUMAN | 46852.8355 | 1.09 | 0.187 | 0.05478 |
| sp|Q86WJ1|CHD1L_HUMAN | 101489.2764 | 0.86 | 0.165 | 0.000208 |
| sp|Q8WV93|AFG1L_HUMAN | 55419.12928 | 1.21 | 0.303 | 0.007019 |
| sp|P10768|ESTD_HUMAN | 31937.64061 | 1.07 | 0.144 | 0.03869 |
| sp|Q96A35|RM24_HUMAN | 24995.01982 | 1.27 | 0.179 | 8.20E-08 |
| sp|Q01581|HMCS1_HUMAN | 57809.55611 | 0.75 | 0.326 | 0.0001677 |
| sp|P56279|TCL1A_HUMAN | 13489.94294 | 1.62 | 0.933 | 0.07194 |
| sp|Q9NTZ6|RBM12_HUMAN | 97543.47637 | 1.23 | 0.153 | 3.82E-08 |
| sp|P52815|RM12_HUMAN | 21544.72339 | 1.25 | 0.296 | 0.0003176 |
| sp|O94964|SOGA1_HUMAN | 161182.506 | 1.28 | 0.258 | 4.85E-06 |
| sp|P49757|NUMB_HUMAN | 71425.62861 | 1.19 | 0.159 | 2.63E-06 |
| sp|Q7Z4H8|PLGT3_HUMAN | 58916.35617 | 1.41 | 0.241 | 4.88E-09 |
| sp|P43308|SSRB_HUMAN | 20104.47503 | 1.28 | 0.206 | 8.69E-08 |
| sp|Q14566|MCM6_HUMAN | 93782.59163 | 1.05 | 0.317 | 0.9219 |
| sp|Q13835|PKP1_HUMAN | 84101.31273 | 1.01 | 0.818 | 0.05076 |
| sp|C9J798|RAS4B_HUMAN | 91358.00787 | 0.71 | 0.143 | 7.51E-09 |
| sp|Q15397|PUM3_HUMAN | 73919.41994 | 0.79 | 0.126 | 8.71E-08 |
| sp|Q5JVF3|PCID2_HUMAN | 46609.02696 | 0.9 | 0.139 | 0.0009495 |
| sp|O95140|MFN2_HUMAN | 87070.09751 | 0.76 | 0.077 | 7.10E-13 |
| sp|Q8N6G6|ATL1_HUMAN | 199253.4287 | 0.8 | 0.319 | 0.006446 |
| sp|Q08554|DSC1_HUMAN | 101388.2967 | 0.86 | 0.139 | 2.39E-05 |
| sp|Q14527|HLTF_HUMAN | 114865.0429 | 0.9 | 0.479 | 0.02513 |
| sp|Q9BY44|EIF2A_HUMAN | 65501.38334 | 0.89 | 0.121 | 7.85E-05 |
| sp|P35659|DEK_HUMAN | 42915.01724 | 1.23 | 0.271 | 0.000362 |
| sp|P19387|RPB3_HUMAN | 31745.85982 | 1.35 | 0.153 | 1.77E-12 |
| sp|Q9BU61|NDUF3_HUMAN | 20547.49094 | 1.04 | 0.092 | 0.08641 |
| sp|Q86VS8|HOOK3_HUMAN | 83455.57631 | 1.6 | 0.255 | 6.78E-13 |
| sp|P23528|COF1_HUMAN | 18700.73307 | 1.25 | 0.23 | 1.73E-05 |
| sp|Q86XP6|GKN2_HUMAN | 20740.64649 | 0.7 | 0.333 | 0.002281 |
| sp|Q96EX1|SIM12_HUMAN | 10774.66489 | 1.03 | 0.243 | 0.9188 |
| sp|O95197|RTN3_HUMAN | 113150.6516 | 0.71 | 0.072 | 7.67E-15 |
| sp|Q9UNL2|SSRG_HUMAN | 21049.18623 | 0.43 | 0.248 | 2.22E-09 |
| sp|Q14134|TRI29_HUMAN | 66459.5494 | 1.3 | 0.691 | 0.07093 |
| sp|Q9Y4G6|TLN2_HUMAN | 273763.2669 | 1.33 | 0.226 | 4.26E-08 |
| sp|Q86SF2|GALT7_HUMAN | 76007.2672 | 0.66 | 0.253 | 6.81E-06 |
| sp|Q96BN2|TADA1_HUMAN | 37625.26554 | 1.2 | 0.186 | 3.92E-05 |
| sp|P18627|LAG3_HUMAN | 57850.82768 | 1.1 | 0.667 | 0.7138 |
| sp|Q9Y385|UB2J1_HUMAN | 35386.74177 | 1 | 0.133 | 0.8743 |
| sp|P36507|MP2K2_HUMAN | 44663.14956 | 0.88 | 0.094 | 1.82E-06 |
| sp|O43681|ASNA_HUMAN | 39205.57809 | 1 | 0.11 | 0.9451 |
| sp|Q9BVS4|RIOK2_HUMAN | 63681.40701 | 1.49 | 0.283 | 1.28E-10 |
| sp|Q6N069|NAA16_HUMAN | 102405.5242 | 0.86 | 0.103 | 1.03E-06 |
| sp|Q04695|K1C17_HUMAN | 48343.1409 | 2.71 | 2.854 | 0.0009677 |
| sp|Q6P158|DHX57_HUMAN | 157085.3941 | 0.9 | 0.157 | 0.003433 |
| sp|P15169|CBPN_HUMAN | 52520.4088 | 1.15 | 0.277 | 0.03613 |
| sp|O95394|AGM1_HUMAN | 60252.42932 | 0.69 | 0.111 | 1.55E-11 |
| sp|Q13572|ITPK1_HUMAN | 46144.31185 | 0.61 | 0.195 | 1.12E-08 |
| sp|Q6UWP8|SBSN_HUMAN | 60543.60703 | 1.03 | 0.319 | 0.8345 |
| sp|Q12860|CNTN1_HUMAN | 114086.2644 | 1.01 | 0.416 | 0.3882 |
| sp|Q9UDY4|DNJB4_HUMAN | 37993.20894 | 1.05 | 0.27 | 0.8273 |
| sp|Q6ICG6|K0930_HUMAN | 46145.84515 | 0.91 | 0.168 | 0.005147 |
| sp|Q9Y6C9|MTCH2_HUMAN | 33918.08956 | 0.57 | 0.131 | 4.60E-12 |
| sp|Q14508|WFDC2_HUMAN | 13935.23096 | 0.58 | 0.417 | 3.14E-06 |
| sp|Q9BTT0|AN32E_HUMAN | 30883.92331 | 1.22 | 0.188 | 2.24E-06 |
| sp|Q9BUQ8|DDX23_HUMAN | 95848.01216 | 1.17 | 0.171 | 1.98E-05 |
| sp|P41218|MNDA_HUMAN | 46074.31947 | 0.88 | 0.44 | 0.02317 |
| sp|P05156|CFAI_HUMAN | 68083.49883 | 0.8 | 0.176 | 1.49E-05 |
| sp|Q12802|AKP13_HUMAN | 310363.2073 | 1.23 | 0.094 | 3.85E-13 |
| sp|Q9P2R6|RERE_HUMAN | 173439.2192 | 2.09 | 0.783 | 4.01E-09 |
| sp|Q0VF96|CGNL1_HUMAN | 149540.7993 | 1.65 | 0.558 | 1.30E-06 |
| sp|O15541|R113A_HUMAN | 39485.87199 | 1.46 | 0.273 | 7.45E-10 |
| sp|Q6PCB7|S27A1_HUMAN | 71900.17114 | 0.8 | 0.214 | 3.88E-05 |
| sp|Q8TC07|TBC15_HUMAN | 80334.00718 | 0.87 | 0.047 | 2.48E-12 |
| sp|P18577|RHCE_HUMAN | 45854.40962 | 0.89 | 0.223 | 0.009776 |
| sp|Q8WZ42|TITIN_HUMAN | 3842885.76 | 1.79 | 0.867 | 0.00012 |
| 100 | NP_001243779.1 | Titin OS=Homo sapiens GN=TTN PE=1 SV=4 | 7.01E+04 | KOG0613 |
| sp|Q14123|PDE1C_HUMAN | 80863.20416 | 0.88 | 0.12 | 1.66E-05 |
| sp|Q9H4G0|E41L1_HUMAN | 98994.37917 | 0.83 | 0.285 | 0.002398 |
| sp|P06702|S10A9_HUMAN | 13272.51914 | 1 | 0.867 | 0.06902 |
| sp|Q86YP4|P66A_HUMAN | 68344.66461 | 1.43 | 0.309 | 1.72E-07 |
| sp|Q9Y666|S12A7_HUMAN | 120265.0408 | 0.92 | 0.155 | 0.008271 |
| sp|Q9NZU0|FLRT3_HUMAN | 73623.73448 | 1.35 | 0.281 | 1.57E-07 |
| sp|P54725|RD23A_HUMAN | 39623.65917 | 1.86 | 0.277 | 2.20E-16 |
| sp|A9UHW6|MI4GD_HUMAN | 25730.96771 | 1.01 | 0.187 | 0.9473 |
| sp|Q8N4C8|MINK1_HUMAN | 150395.0088 | 1.13 | 0.246 | 0.05164 |
| sp|P52735|VAV2_HUMAN | 102460.2177 | 0.73 | 0.074 | 4.00E-14 |
| sp|Q9BVL4|SELO_HUMAN | 74281.00863 | 0.97 | 0.191 | 0.2295 |
| sp|P16403|H12_HUMAN | 21333.73698 | 1.68 | 0.654 | 1.05E-05 |
| sp|O75525|KHDR3_HUMAN | 38757.6107 | 1.49 | 0.689 | 0.003711 |
| sp|Q6P5S2|LEG1H_HUMAN | 38225.67735 | 0.65 | 0.312 | 0.0006861 |
| sp|Q969N2|PIGT_HUMAN | 66210.14476 | 0.59 | 0.124 | 1.99E-12 |
| sp|Q9Y6R9|CCD61_HUMAN | 57543.4364 | 1.06 | 0.161 | 0.135 |
| sp|Q5JWR5|DOP1_HUMAN | 279384.4236 | 1.11 | 0.513 | 0.8209 |
| sp|Q9Y6X0|SETBP_HUMAN | 175622.3412 | 1.46 | 0.815 | 0.0103 |
| sp|Q58A45|PAN3_HUMAN | 96275.44302 | 1 | 0.338 | 0.4204 |
| sp|Q9NQR4|NIT2_HUMAN | 30969.69564 | 1.14 | 0.093 | 4.42E-08 |
| sp|A0A0C4DH67|KV108_HUMAN | 12739.3103 | 1.19 | 0.295 | 0.006691 |
| sp|Q8WVC0|LEO1_HUMAN | 75455.32596 | 1.34 | 0.15 | 2.04E-12 |
| sp|Q13045|FLII_HUMAN | 146124.0426 | 0.67 | 0.103 | 8.60E-13 |
| sp|Q9Y4D7|PLXD1_HUMAN | 215274.6576 | 1.2 | 0.318 | 0.01878 |
| sp|Q76N32|CEP68_HUMAN | 81946.4686 | 1.88 | 0.857 | 1.34E-06 |
| sp|O95834|EMAL2_HUMAN | 71585.42685 | 0.65 | 0.137 | 3.83E-10 |
| sp|Q5VT66|MARC1_HUMAN | 37970.71853 | 1.24 | 0.285 | 0.0001866 |
| sp|Q06546|GABPA_HUMAN | 51758.293 | 0.8 | 0.158 | 1.70E-06 |
| sp|Q9BYN8|RT26_HUMAN | 24235.86766 | 1.21 | 0.239 | 0.0005056 |
| sp|Q13574|DGKZ_HUMAN | 125685.9165 | 1.53 | 0.641 | 0.0001544 |
| sp|Q9P2B2|FPRP_HUMAN | 99445.95244 | 0.82 | 0.213 | 7.05E-05 |
| sp|P22413|ENPP1_HUMAN | 107005.5127 | 0.73 | 0.17 | 1.05E-07 |
| sp|Q96CS2|HAUS1_HUMAN | 31882.59941 | 1.36 | 0.173 | 1.24E-11 |
| sp|Q99447|PCY2_HUMAN | 44246.08759 | 1.02 | 0.126 | 0.5696 |
| sp|Q5T1J5|CHCH9_HUMAN | 15689.78815 | 1.11 | 0.32 | 0.2954 |
| sp|P78545|ELF3_HUMAN | 41809.30474 | 0.93 | 0.236 | 0.05686 |
| sp|P62330|ARF6_HUMAN | 20165.46716 | 0.54 | 0.11 | 1.85E-13 |
| sp|P17844|DDX5_HUMAN | 69599.91649 | 1.31 | 0.244 | 1.05E-06 |
| sp|O00193|SMAP_HUMAN | 20359.22652 | 1.9 | 0.988 | 1.93E-06 |
| sp|Q9Y2S7|PDIP2_HUMAN | 42217.23695 | 1.21 | 0.24 | 0.0003183 |
| sp|Q9UBS8|RNF14_HUMAN | 55608.29731 | 1.13 | 0.464 | 0.5383 |
| sp|Q9BVL2|NUP58_HUMAN | 60955.71386 | 1.17 | 0.275 | 0.01197 |
| sp|P82980|RET5_HUMAN | 16074.11609 | 0.91 | 0.358 | 0.04737 |
| sp|P51956|NEK3_HUMAN | 58163.38732 | 0.91 | 0.162 | 0.004904 |
| sp|Q8ND90|PNMA1_HUMAN | 39889.35599 | 1.1 | 0.338 | 0.3403 |
| sp|Q6UX53|MET7B_HUMAN | 28251.45704 | 0.49 | 0.156 | 1.35E-11 |
| sp|O14976|GAK_HUMAN | 144564.8083 | 0.88 | 0.157 | 0.0003477 |
| sp|P09972|ALDOC_HUMAN | 39812.3767 | 1.18 | 0.136 | 2.23E-07 |
| sp|P35625|TIMP3_HUMAN | 24795.07651 | 0.9 | 0.763 | 0.01742 |
| sp|Q9ULX6|AKP8L_HUMAN | 72032.84825 | 1.3 | 0.167 | 3.77E-10 |
| sp|P52333|JAK3_HUMAN | 126825.6479 | 0.88 | 0.308 | 0.01166 |
| sp|Q9UH62|ARMX3_HUMAN | 42797.98923 | 0.71 | 0.111 | 2.88E-11 |
| sp|P38117|ETFB_HUMAN | 28036.22821 | 1.31 | 0.176 | 1.15E-09 |
| sp|Q4U2R6|RM51_HUMAN | 15181.18522 | 0.8 | 0.193 | 1.34E-05 |
| sp|O60669|MOT2_HUMAN | 52547.29835 | 1.06 | 0.259 | 0.6344 |
| sp|P38606|VATA_HUMAN | 68641.63192 | 1.09 | 0.113 | 0.0003741 |
| sp|P42768|WASP_HUMAN | 53089.93836 | 1.24 | 0.338 | 0.006244 |
| sp|Q99973|TEP1_HUMAN | 293482.18 | 0.66 | 0.105 | 7.60E-13 |
| sp|Q9UGI8|TES_HUMAN | 49770.56849 | 0.76 | 0.121 | 1.08E-08 |
| sp|Q8IWA0|WDR75_HUMAN | 95902.99561 | 0.7 | 0.084 | 1.64E-13 |
| sp|Q96QA5|GSDMA_HUMAN | 49600.91684 | 1.12 | 0.386 | 0.4229 |
| sp|P09104|ENOG_HUMAN | 47563.1214 | 1.02 | 0.188 | 0.9848 |
| sp|P67809|YBOX1_HUMAN | 35884.66505 | 1.75 | 0.466 | 7.26E-10 |
| sp|O95999|BCL10_HUMAN | 26502.42256 | 1.14 | 0.179 | 0.0006166 |
| sp|Q96FV9|THOC1_HUMAN | 76341.80941 | 0.78 | 0.13 | 9.80E-08 |
| sp|Q53T59|H1BP3_HUMAN | 42849.95438 | 1.11 | 0.119 | 0.0001813 |
| sp|Q9UKN7|MYO15_HUMAN | 397591.5668 | 1.21 | 0.632 | 0.5636 |
| sp|Q04656|ATP7A_HUMAN | 164733.2296 | 0.64 | 0.116 | 8.16E-12 |
| sp|Q8IZL8|PELP1_HUMAN | 120860.9542 | 0.75 | 0.099 | 3.63E-11 |
| sp|Q9P0M6|H2AW_HUMAN | 40243.44137 | 1.05 | 0.21 | 0.5439 |
| sp|Q9Y5L0|TNPO3_HUMAN | 105942.7503 | 0.97 | 0.095 | 0.1032 |
| sp|Q06187|BTK_HUMAN | 76898.60603 | 0.88 | 0.173 | 0.001058 |
| sp|O95786|DDX58_HUMAN | 107995.8606 | 0.82 | 0.179 | 7.72E-05 |
| sp|P41567|EIF1_HUMAN | 12820.59665 | 1.33 | 0.238 | 1.24E-07 |
| sp|Q9Y6I9|TX264_HUMAN | 34434.06174 | 1.07 | 0.125 | 0.02695 |
| sp|Q12778|FOXO1_HUMAN | 69998.52224 | 1.4 | 0.63 | 0.008346 |
| sp|O14972|DSCR3_HUMAN | 33427.46512 | 0.63 | 0.109 | 1.44E-12 |
| sp|Q8N5N7|RM50_HUMAN | 18466.42415 | 1 | 0.155 | 0.6249 |
| sp|Q9UBV8|PEF1_HUMAN | 30628.40299 | 0.63 | 0.065 | 2.20E-16 |
| sp|P50749|RASF2_HUMAN | 37919.58484 | 1.05 | 0.245 | 0.6632 |
| sp|P09417|DHPR_HUMAN | 25983.10369 | 1.11 | 0.192 | 0.01798 |
| sp|O95562|SFT2B_HUMAN | 18148.62024 | 0.83 | 0.134 | 2.05E-06 |
| sp|Q8NCJ5|SPRY3_HUMAN | 50157.65564 | 1.16 | 0.077 | 4.89E-11 |
| sp|Q8NC56|LEMD2_HUMAN | 57320.66671 | 0.95 | 0.135 | 0.06304 |
| sp|Q6V1P9|PCD23_HUMAN | 323726.2581 | 0.53 | 0.154 | 5.18E-10 |
| sp|Q12769|NU160_HUMAN | 164336.8209 | 0.64 | 0.118 | 3.36E-12 |
| sp|Q8TD30|ALAT2_HUMAN | 58646.73604 | 0.97 | 0.315 | 0.1916 |
| sp|Q9C099|LRCC1_HUMAN | 120701.0268 | 0.7 | 0.16 | 5.64E-08 |
| sp|O75487|GPC4_HUMAN | 63437.06841 | 0.86 | 0.134 | 6.18E-05 |
| sp|O43182|RHG06_HUMAN | 106605.5808 | 0.99 | 0.11 | 0.3726 |
| sp|Q96PY6|NEK1_HUMAN | 143577.6273 | 1.4 | 0.421 | 1.89E-05 |
| sp|Q9UL25|RAB21_HUMAN | 24713.3952 | 0.84 | 0.088 | 6.57E-09 |
| sp|Q9Y3Q8|T22D4_HUMAN | 41039.90569 | 1.35 | 0.232 | 3.18E-08 |
| sp|O14791|APOL1_HUMAN | 43985.90509 | 0.5 | 0.13 | 3.37E-12 |
| sp|P28062|PSB8_HUMAN | 30658.68878 | 0.88 | 0.129 | 5.65E-05 |
| sp|P08842|STS_HUMAN | 66287.36842 | 0.65 | 0.195 | 2.18E-07 |
| sp|Q96CS7|PKHB2_HUMAN | 25043.9802 | 0.71 | 0.308 | 2.88E-05 |
| sp|Q6FHJ7|SFRP4_HUMAN | 40865.72294 | 2.58 | 1.43 | 0.0001319 |
| sp|Q9H0G5|NSRP1_HUMAN | 66560.14068 | 1.21 | 0.232 | 0.0002013 |
| sp|P30038|AL4A1_HUMAN | 62118.75679 | 0.84 | 0.151 | 2.14E-05 |
| sp|P07686|HEXB_HUMAN | 63509.34097 | 1.05 | 0.342 | 0.9446 |
| sp|Q01415|GALK2_HUMAN | 51239.94167 | 0.75 | 0.196 | 2.90E-06 |
| sp|Q9Y276|BCS1_HUMAN | 47657.5034 | 0.92 | 0.115 | 0.0008781 |
| sp|P12074|CX6A1_HUMAN | 12129.13759 | 1.06 | 0.361 | 0.9785 |
| sp|Q9BTT6|LRRC1_HUMAN | 59928.13001 | 0.82 | 0.533 | 0.005234 |
| sp|P40429|RL13A_HUMAN | 23601.38822 | 0.91 | 0.119 | 0.0005921 |
| sp|Q9HCJ6|VAT1L_HUMAN | 46194.25894 | 0.53 | 0.205 | 1.84E-08 |
| sp|Q6PML9|ZNT9_HUMAN | 64084.0173 | 0.76 | 0.092 | 1.39E-11 |
| sp|Q8NDI1|EHBP1_HUMAN | 140540.4997 | 1.18 | 0.164 | 9.76E-06 |
| sp|P12111|CO6A3_HUMAN | 345149.4054 | 0.87 | 0.222 | 0.002239 |
| sp|Q0VDF9|HSP7E_HUMAN | 55426.30474 | 1.06 | 0.168 | 0.1929 |
| sp|O43301|HS12A_HUMAN | 75198.59497 | 1.3 | 0.276 | 2.77E-06 |
| sp|O75394|RM33_HUMAN | 7653.277507 | 1.15 | 0.243 | 0.01159 |
| sp|Q8NI22|MCFD2_HUMAN | 16475.83025 | 1.56 | 0.37 | 2.01E-08 |
| sp|Q96QD9|UIF_HUMAN | 35835.45302 | 1.43 | 0.425 | 7.34E-05 |
| sp|Q13371|PHLP_HUMAN | 34640.83265 | 1.3 | 0.128 | 1.51E-12 |
| sp|Q96EZ8|MCRS1_HUMAN | 51981.27869 | 0.96 | 0.076 | 0.01487 |
| sp|Q9HAV0|GBB4_HUMAN | 38266.2904 | 1.29 | 0.339 | 0.000162 |
| sp|Q9NZR2|LRP1B_HUMAN | 534813.7694 | 1.14 | 0.245 | 0.01647 |
| sp|P15735|PHKG2_HUMAN | 46793.80104 | 1.04 | 0.578 | 0.3396 |
| sp|Q9P0W2|HM20B_HUMAN | 35886.57927 | 1.42 | 0.275 | 6.84E-09 |
| sp|P46776|RL27A_HUMAN | 16647.0757 | 1.21 | 0.241 | 0.000759 |
| sp|P13760|2B14_HUMAN | 30417.12654 | 0.78 | 0.385 | 0.007893 |
| sp|O94811|TPPP_HUMAN | 23832.2713 | 1.09 | 0.182 | 0.03974 |
| sp|P13727|PRG2_HUMAN | 25855.50417 | 0.59 | 0.184 | 9.07E-08 |
| sp|Q9HB90|RRAGC_HUMAN | 44576.97071 | 0.62 | 0.13 | 6.40E-11 |
| sp|P12109|CO6A1_HUMAN | 109584.43 | 1.12 | 0.27 | 0.05343 |
| sp|Q66PJ3|AR6P4_HUMAN | 45269.34285 | 1.3 | 0.414 | 0.0008899 |
| sp|Q6UX04|CWC27_HUMAN | 53967.22103 | 1.23 | 0.137 | 5.16E-09 |
| sp|Q9UKL0|RCOR1_HUMAN | 53390.67324 | 1.17 | 0.191 | 0.0001263 |
| sp|Q8IY21|DDX60_HUMAN | 199704.4035 | 0.67 | 0.205 | 3.35E-07 |
| sp|P25054|APC_HUMAN | 313603.7104 | 1.24 | 0.305 | 0.0006251 |
| sp|P0DP23|CALM1_HUMAN | 16808.82375 | 1.6 | 0.31 | 9.10E-12 |
| sp|Q9Y282|ERGI3_HUMAN | 43746.69992 | 0.81 | 0.124 | 1.38E-07 |
| sp|O14733|MP2K7_HUMAN | 47892.66654 | 0.73 | 0.163 | 7.24E-08 |
| sp|Q15208|STK38_HUMAN | 54479.49126 | 0.99 | 0.098 | 0.6108 |
| sp|P27487|DPP4_HUMAN | 88888.73139 | 1.18 | 0.298 | 0.009808 |
| sp|Q9UL26|RB22A_HUMAN | 22051.29363 | 0.71 | 0.115 | 4.50E-11 |
| sp|Q8TD43|TRPM4_HUMAN | 135679.8085 | 0.68 | 0.144 | 2.00E-09 |
| sp|Q01974|ROR2_HUMAN | 106325.2514 | 0.76 | 0.205 | 4.51E-06 |
| sp|Q9Y2D0|CAH5B_HUMAN | 36734.61255 | 1.29 | 0.233 | 5.33E-07 |
| sp|P48643|TCPE_HUMAN | 60070.9687 | 1.35 | 0.205 | 5.24E-10 |
| sp|O14841|OPLA_HUMAN | 138721.1127 | 0.77 | 0.13 | 4.44E-08 |
| sp|Q9Y5P6|GMPPB_HUMAN | 40360.7921 | 0.64 | 0.184 | 1.58E-08 |
| sp|Q9BY76|ANGL4_HUMAN | 45566.98566 | 1.21 | 0.287 | 0.003493 |
| sp|Q9Y3S2|ZN330_HUMAN | 37812.91912 | 0.94 | 0.157 | 0.02877 |
| sp|Q5M775|CYTSB_HUMAN | 119178.6295 | 1.28 | 0.167 | 3.01E-09 |
| sp|Q06136|KDSR_HUMAN | 36544.77156 | 0.69 | 0.06 | 2.20E-16 |
| sp|P31930|QCR1_HUMAN | 53278.67697 | 1.06 | 0.107 | 0.01598 |
| sp|P04216|THY1_HUMAN | 18133.42267 | 0.8 | 0.177 | 2.61E-06 |
| sp|Q8TCS8|PNPT1_HUMAN | 86505.85262 | 1.01 | 0.18 | 0.8898 |
| sp|Q4LDE5|SVEP1_HUMAN | 404663.0243 | 1.02 | 0.373 | 0.6158 |
| sp|P35052|GPC1_HUMAN | 62706.41884 | 0.88 | 0.191 | 0.001209 |
| sp|P09960|LKHA4_HUMAN | 69850.46304 | 0.56 | 0.071 | 2.20E-16 |
| sp|P08582|TRFM_HUMAN | 81741.89738 | 1.06 | 0.254 | 0.5088 |
| sp|Q9NU22|MDN1_HUMAN | 637990.2493 | 0.99 | 0.227 | 0.393 |
| sp|Q13546|RIPK1_HUMAN | 76434.75131 | 0.8 | 0.153 | 7.90E-06 |
| sp|O95395|GCNT3_HUMAN | 51553.98756 | 0.81 | 0.13 | 7.18E-07 |
| sp|O75330|HMMR_HUMAN | 84429.86511 | 1 | 0.388 | 0.359 |
| sp|Q99460|PSMD1_HUMAN | 106777.0703 | 0.65 | 0.094 | 7.62E-14 |
| sp|B2RTY4|MYO9A_HUMAN | 294899.203 | 0.59 | 0.164 | 6.35E-10 |
| sp|Q96L92|SNX27_HUMAN | 61835.68426 | 0.77 | 0.075 | 9.51E-13 |
| sp|P46940|IQGA1_HUMAN | 189743.0235 | 0.87 | 0.112 | 5.81E-06 |
| sp|Q15024|EXOS7_HUMAN | 32410.32541 | 0.88 | 0.084 | 1.02E-06 |
| sp|P20036|DPA1_HUMAN | 29571.95021 | 1.28 | 0.615 | 0.0948 |
| sp|Q13506|NAB1_HUMAN | 54805.6879 | 1.34 | 0.443 | 0.001613 |
| sp|P07585|PGS2_HUMAN | 40045.95757 | 2.4 | 1.63 | 3.41E-07 |
| sp|Q9UJY4|GGA2_HUMAN | 67602.7434 | 1.01 | 0.144 | 0.8758 |
| sp|Q13177|PAK2_HUMAN | 58272.97985 | 1.33 | 0.169 | 1.64E-10 |
| sp|Q9Y5Z0|BACE2_HUMAN | 56582.88329 | 0.65 | 0.139 | 1.22E-10 |
| sp|Q9UNH7|SNX6_HUMAN | 46886.85277 | 1.59 | 0.203 | 2.16E-15 |
| sp|L0R6Q1|S35U4_HUMAN | 11164.89549 | 0.63 | 0.058 | 2.20E-16 |
| sp|Q9UL45|BL1S6_HUMAN | 19827.28937 | 2.03 | 0.487 | 4.35E-13 |
| sp|Q99571|P2RX4_HUMAN | 44121.20906 | 0.6 | 0.105 | 5.73E-14 |
| sp|P10451|OSTP_HUMAN | 35554.29298 | 1.37 | 0.763 | 0.09022 |
| sp|P54098|DPOG1_HUMAN | 140595.8388 | 0.76 | 0.125 | 1.04E-08 |
| sp|Q9HAU5|RENT2_HUMAN | 148953.7739 | 0.69 | 0.095 | 1.59E-12 |
| sp|O95571|ETHE1_HUMAN | 28350.29231 | 0.81 | 0.149 | 3.19E-06 |
| sp|Q9UKK6|NXT1_HUMAN | 16046.70379 | 0.52 | 0.156 | 2.62E-11 |
| sp|Q8N3J5|PPM1K_HUMAN | 41466.81688 | 1.28 | 0.344 | 0.0003742 |
| sp|P49758|RGS6_HUMAN | 54712.79806 | 1.29 | 0.71 | 0.1291 |
| sp|Q9NRS6|SNX15_HUMAN | 38306.60684 | 1.25 | 0.109 | 6.88E-12 |
| sp|Q7Z6Z7|HUWE1_HUMAN | 485505.1256 | 0.66 | 0.093 | 9.88E-14 |
| sp|P61421|VA0D1_HUMAN | 40741.378 | 0.82 | 0.168 | 2.84E-05 |
| sp|Q96F85|CNRP1_HUMAN | 18732.58518 | 1.47 | 0.341 | 2.22E-07 |
| sp|O14686|KMT2D_HUMAN | 599556.6582 | 1.16 | 0.136 | 2.15E-06 |
| sp|O96008|TOM40_HUMAN | 38193.25852 | 0.51 | 0.164 | 5.33E-11 |
| sp|Q9C086|IN80B_HUMAN | 38994.09414 | 1.34 | 0.346 | 2.94E-05 |
| sp|Q9UIV1|CNOT7_HUMAN | 33048.05886 | 0.72 | 0.17 | 1.38E-07 |
| sp|P38432|COIL_HUMAN | 63236.16807 | 1.12 | 0.32 | 0.1647 |
| sp|Q9BPY8|HOP_HUMAN | 8351.027419 | 1.27 | 0.328 | 0.0003669 |
| sp|O14880|MGST3_HUMAN | 16715.64265 | 0.51 | 0.141 | 2.82E-12 |
| sp|P02741|CRP_HUMAN | 25175.7273 | 0.97 | 0.59 | 0.1154 |
| sp|Q00839|HNRPU_HUMAN | 91251.27327 | 1.29 | 0.195 | 7.74E-08 |
| sp|Q92905|CSN5_HUMAN | 37764.79372 | 0.99 | 0.156 | 0.5253 |
| sp|Q6NWY9|PR40B_HUMAN | 99620.21169 | 1.21 | 0.189 | 8.74E-06 |
| sp|P78344|IF4G2_HUMAN | 102791.9956 | 0.79 | 0.108 | 4.15E-09 |
| sp|P32942|ICAM3_HUMAN | 60454.18524 | 0.96 | 0.213 | 0.1683 |
| sp|Q86YS7|C2CD5_HUMAN | 111898.0773 | 0.64 | 0.151 | 2.17E-09 |
| sp|Q8TEB1|DCA11_HUMAN | 62240.48583 | 0.77 | 0.18 | 1.13E-05 |
| sp|O43747|AP1G1_HUMAN | 92130.44003 | 0.55 | 0.075 | 2.20E-16 |
| sp|Q8NBJ9|SIDT2_HUMAN | 95572.63872 | 0.73 | 0.113 | 1.80E-10 |
| sp|Q8N4P3|MESH1_HUMAN | 20355.6783 | 1.33 | 0.258 | 1.16E-06 |
| sp|Q8IWT6|LRC8A_HUMAN | 95033.01939 | 0.59 | 0.058 | 2.20E-16 |
| sp|Q9H2M9|RBGPR_HUMAN | 157464.3524 | 0.71 | 0.078 | 4.18E-14 |
| sp|P50993|AT1A2_HUMAN | 113486.9148 | 0.62 | 0.224 | 4.18E-08 |
| sp|O00622|CYR61_HUMAN | 44147.0833 | 0.97 | 0.411 | 0.1708 |
| sp|Q02083|NAAA_HUMAN | 40193.91555 | 0.52 | 0.156 | 7.05E-11 |
| sp|Q8N126|CADM3_HUMAN | 43710.86496 | 0.89 | 0.241 | 0.008929 |
| sp|P56945|BCAR1_HUMAN | 93524.43748 | 0.99 | 0.191 | 0.5573 |
| sp|Q9Y4C8|RBM19_HUMAN | 107703.6186 | 1.28 | 0.212 | 3.67E-07 |
| sp|Q9NUB1|ACS2L_HUMAN | 75646.30442 | 0.55 | 0.17 | 4.29E-10 |
| sp|Q9P246|STIM2_HUMAN | 84755.54348 | 0.92 | 0.128 | 0.003158 |
| sp|Q03468|ERCC6_HUMAN | 169433.8526 | 1.16 | 0.208 | 0.0008074 |
| sp|P45983|MK08_HUMAN | 48816.56399 | 0.9 | 0.14 | 0.001095 |
| sp|P61353|RL27_HUMAN | 15769.73703 | 1.19 | 0.114 | 5.80E-09 |
| sp|Q6Y1H2|HACD2_HUMAN | 28389.15024 | 0.66 | 0.148 | 1.02E-09 |
| sp|Q9UEW3|MARCO_HUMAN | 52949.49876 | 1.38 | 0.189 | 6.07E-11 |
| sp|Q96CS3|FAF2_HUMAN | 52914.66381 | 0.82 | 0.086 | 1.66E-09 |
| sp|P10586|PTPRF_HUMAN | 213923.9295 | 0.99 | 0.186 | 0.5468 |
| sp|Q92552|RT27_HUMAN | 47905.48995 | 0.72 | 0.15 | 3.02E-08 |
| sp|A0FGR8|ESYT2_HUMAN | 102789.1159 | 0.93 | 0.107 | 0.003179 |
| sp|P60228|EIF3E_HUMAN | 52568.5278 | 0.71 | 0.092 | 1.16E-12 |
| sp|Q13137|CACO2_HUMAN | 52943.88262 | 1.14 | 0.194 | 0.002199 |
| sp|Q2WGJ9|FR1L6_HUMAN | 210412.6112 | 0.65 | 0.229 | 3.25E-05 |
| sp|O60224|SSX4_HUMAN | 21883.03037 | 1.1 | 0.159 | 0.007543 |
| sp|P02748|CO9_HUMAN | 64597.24656 | 0.88 | 0.343 | 0.02332 |
| sp|Q96C23|GALM_HUMAN | 37952.20661 | 0.97 | 0.17 | 0.2625 |
| sp|P02768|ALBU_HUMAN | 71299.23638 | 1.1 | 0.297 | 0.2181 |
| sp|Q6PL24|TMED8_HUMAN | 35928.43551 | 1.46 | 0.243 | 2.82E-11 |
| sp|P51659|DHB4_HUMAN | 80074.46673 | 0.72 | 0.075 | 1.81E-14 |
| sp|P05204|HMGN2_HUMAN | 9369.046877 | 2.76 | 1.602 | 2.12E-08 |
| sp|Q9H4M7|PKHA4_HUMAN | 85559.01613 | 1.43 | 0.399 | 6.58E-06 |
| sp|Q96N21|AP4AT_HUMAN | 55825.98573 | 0.78 | 0.238 | 6.85E-05 |
| sp|O00443|P3C2A_HUMAN | 192137.8507 | 1.13 | 0.239 | 0.01518 |
| sp|O94868|FCSD2_HUMAN | 84719.27742 | 1.27 | 0.146 | 3.34E-10 |
| sp|O00194|RB27B_HUMAN | 24802.30488 | 0.59 | 0.232 | 6.49E-06 |
| sp|O60462|NRP2_HUMAN | 106114.4751 | 0.61 | 0.068 | 2.20E-16 |
| sp|Q9H6S1|AZI2_HUMAN | 45401.89868 | 1.52 | 0.271 | 2.46E-11 |
| sp|Q86UK7|ZN598_HUMAN | 100381.8758 | 1.1 | 0.137 | 0.001563 |
| sp|O75319|DUS11_HUMAN | 44121.12584 | 1.13 | 0.268 | 0.06652 |
| sp|O14562|UBFD1_HUMAN | 33456.88145 | 1.51 | 0.288 | 5.11E-10 |
| sp|P17174|AATC_HUMAN | 46428.60416 | 0.79 | 0.124 | 7.17E-08 |
| sp|Q92791|SC65_HUMAN | 50844.38955 | 1.63 | 0.394 | 3.54E-09 |
| sp|Q07817|B2CL1_HUMAN | 26071.71313 | 0.92 | 0.352 | 0.05547 |
| sp|Q68CQ4|DIEXF_HUMAN | 87324.90299 | 0.64 | 0.166 | 1.29E-09 |
| sp|Q8IVD9|NUDC3_HUMAN | 40835.67549 | 1.24 | 0.262 | 0.0001884 |
| sp|P29218|IMPA1_HUMAN | 30550.30605 | 0.87 | 0.165 | 0.0003171 |
| sp|Q9H6W3|RIOX1_HUMAN | 71365.53719 | 1.22 | 0.134 | 1.06E-08 |
| sp|P05121|PAI1_HUMAN | 45070.04303 | 0.77 | 0.445 | 0.001421 |
| sp|A5PLL7|TM189_HUMAN | 31553.06548 | 0.88 | 0.241 | 0.008345 |
| sp|Q9UHD1|CHRD1_HUMAN | 38245.92436 | 0.83 | 0.212 | 0.0001308 |
| sp|O15117|FYB1_HUMAN | 85545.20731 | 1.71 | 0.458 | 2.52E-09 |
| sp|Q8NBU5|ATAD1_HUMAN | 41042.11183 | 0.63 | 0.116 | 5.25E-12 |
| sp|Q9BY50|SC11C_HUMAN | 21510.44106 | 0.82 | 0.16 | 2.04E-05 |
| sp|Q13418|ILK_HUMAN | 51881.10753 | 0.79 | 0.123 | 6.63E-08 |
| sp|Q14CN4|K2C72_HUMAN | 56451.66333 | 1.41 | 0.456 | 0.0001335 |
| sp|P42167|LAP2B_HUMAN | 50678.46499 | 0.84 | 0.282 | 0.002006 |
| sp|P21695|GPDA_HUMAN | 38152.54346 | 3.41 | 1.291 | 2.65E-13 |
| sp|Q6GMV3|PTRD1_HUMAN | 15891.33673 | 1.44 | 0.184 | 7.24E-13 |
| sp|Q96J88|ESIP1_HUMAN | 36923.99295 | 1.87 | 0.545 | 1.67E-10 |
| sp|Q9Y5B0|CTDP1_HUMAN | 105286.0015 | 1.25 | 0.161 | 1.33E-08 |
| sp|Q92556|ELMO1_HUMAN | 84498.87555 | 0.68 | 0.127 | 5.13E-11 |
| sp|P09913|IFIT2_HUMAN | 55263.73532 | 1.23 | 0.254 | 0.0003015 |
| sp|Q96BI1|S22AI_HUMAN | 45254.31711 | 0.53 | 0.131 | 5.60E-12 |
| sp|Q641Q3|METRL_HUMAN | 34928.52428 | 1 | 0.159 | 0.7462 |
| sp|Q63HM2|PCX4_HUMAN | 133738.2354 | 2.12 | 1.078 | 7.88E-07 |
| sp|Q86YR7|MF2L2_HUMAN | 128149.1365 | 1.63 | 0.732 | 9.56E-05 |
| sp|P03973|SLPI_HUMAN | 15210.24182 | 0.67 | 0.472 | 9.47E-05 |
| sp|P13861|KAP2_HUMAN | 45814.07402 | 1.22 | 0.186 | 1.98E-06 |
| sp|P51688|SPHM_HUMAN | 57154.63467 | 1.14 | 0.144 | 4.27E-05 |
| sp|Q8WUQ7|CATIN_HUMAN | 88743.57339 | 1.39 | 0.283 | 2.56E-08 |
| sp|O15269|SPTC1_HUMAN | 53262.671 | 0.81 | 0.038 | 2.20E-16 |
| sp|O75689|ADAP1_HUMAN | 43691.92699 | 0.9 | 0.167 | 0.003936 |
| sp|Q9NW15|ANO10_HUMAN | 76775.00636 | 0.54 | 0.052 | 2.20E-16 |
| sp|Q96MX6|WDR92_HUMAN | 40153.31445 | 1.08 | 0.228 | 0.2367 |
| sp|Q9C0B1|FTO_HUMAN | 59025.15812 | 1.11 | 0.119 | 9.65E-05 |
| sp|Q16626|MEA1_HUMAN | 19874.40083 | 1.94 | 1.091 | 0.0001034 |
| sp|Q9BU89|DOHH_HUMAN | 33264.5892 | 1.12 | 0.153 | 0.0005466 |
| sp|P56199|ITA1_HUMAN | 132286.2786 | 0.63 | 0.154 | 2.51E-10 |
| sp|Q9Y496|KIF3A_HUMAN | 80315.0954 | 0.99 | 0.115 | 0.4126 |
| sp|Q6DKI1|RL7L_HUMAN | 29803.62949 | 0.93 | 0.334 | 0.07026 |
| sp|P21757|MSRE_HUMAN | 50168.98081 | 1.36 | 0.258 | 8.79E-08 |
| sp|P33260|CP2CI_HUMAN | 56511.6289 | 0.83 | 0.393 | 0.01237 |
| sp|Q0JRZ9|FCHO2_HUMAN | 89364.18198 | 1.34 | 0.193 | 2.90E-10 |
| sp|O75746|CMC1_HUMAN | 75095.98907 | 0.51 | 0.107 | 1.55E-15 |
| sp|Q9BXW6|OSBL1_HUMAN | 109751.5912 | 1.28 | 0.356 | 0.00147 |
| sp|O00501|CLD5_HUMAN | 23798.13925 | 1.14 | 0.619 | 0.8449 |
| sp|Q14161|GIT2_HUMAN | 85099.46939 | 1.35 | 0.132 | 1.02E-13 |
| sp|Q9Y5K5|UCHL5_HUMAN | 37850.08957 | 1.32 | 0.214 | 1.89E-08 |
| sp|Q969X5|ERGI1_HUMAN | 32952.62536 | 0.65 | 0.171 | 4.79E-09 |
| sp|Q96EP0|RNF31_HUMAN | 122636.1946 | 0.93 | 0.089 | 0.0005635 |
| sp|Q8WUA4|TF3C2_HUMAN | 101681.1611 | 0.71 | 0.131 | 4.83E-10 |
| sp|Q9UNA3|A4GCT_HUMAN | 39909.79834 | 1.17 | 0.149 | 3.63E-06 |
| sp|Q13362|2A5G_HUMAN | 61402.70534 | 0.63 | 0.064 | 2.20E-16 |
| sp|Q8N474|SFRP1_HUMAN | 36256.38681 | 1.62 | 0.46 | 6.12E-08 |
| sp|Q8WWX9|SELM_HUMAN | 16261.45315 | 0.88 | 0.164 | 0.0007513 |
| sp|O94760|DDAH1_HUMAN | 31426.05004 | 0.98 | 0.161 | 0.3435 |
| sp|P17813|EGLN_HUMAN | 71541.47845 | 0.92 | 0.167 | 0.01184 |
| sp|Q96NT0|CC115_HUMAN | 19787.367 | 0.95 | 0.137 | 0.04427 |
| sp|Q92696|PGTA_HUMAN | 66095.87477 | 0.95 | 0.122 | 0.02393 |
| sp|P61964|WDR5_HUMAN | 37117.65638 | 0.99 | 0.124 | 0.536 |
| sp|Q96T88|UHRF1_HUMAN | 91278.59603 | 1 | 0.201 | 0.6729 |
| sp|P49959|MRE11_HUMAN | 80867.43678 | 1.08 | 0.254 | 0.3112 |
| sp|Q4KMP7|TB10B_HUMAN | 87639.91672 | 1.02 | 0.086 | 0.2449 |
| sp|P52943|CRIP2_HUMAN | 23258.29222 | 1.26 | 0.465 | 0.02782 |
| sp|Q92185|SIA8A_HUMAN | 41044.49082 | 0.93 | 0.185 | 0.02024 |
| sp|Q7L7V1|DHX32_HUMAN | 85771.34176 | 0.62 | 0.261 | 8.36E-07 |
| sp|P15309|PPAP_HUMAN | 44861.59116 | 0.48 | 0.33 | 4.02E-08 |
| sp|P30419|NMT1_HUMAN | 57093.97052 | 0.93 | 0.211 | 0.04799 |
| sp|Q16527|CSRP2_HUMAN | 21834.21247 | 1.08 | 0.319 | 0.4727 |
| sp|O95714|HERC2_HUMAN | 533491.6772 | 0.88 | 0.116 | 8.32E-05 |
| sp|Q9H257|CARD9_HUMAN | 62697.10139 | 0.8 | 0.237 | 7.41E-05 |
| sp|Q9Y3C0|WASC3_HUMAN | 21198.61033 | 1.3 | 0.125 | 1.20E-12 |
| sp|Q16718|NDUA5_HUMAN | 13489.18561 | 1.45 | 0.378 | 3.03E-06 |
| sp|Q96IY1|NSL1_HUMAN | 32522.62579 | 0.85 | 0.159 | 0.000107 |
| sp|Q86U38|NOP9_HUMAN | 70117.71559 | 0.66 | 0.157 | 1.40E-09 |
| sp|P28068|DMB_HUMAN | 29362.58738 | 0.95 | 0.381 | 0.1288 |
| sp|Q96ST2|IWS1_HUMAN | 91938.16544 | 1.43 | 0.202 | 8.02E-12 |
| sp|Q7Z7A3|CTU1_HUMAN | 37207.14147 | 0.85 | 0.161 | 0.000111 |
| sp|P48163|MAOX_HUMAN | 64661.27274 | 1.13 | 0.296 | 0.1364 |
| sp|Q9UHV9|PFD2_HUMAN | 16676.62034 | 1.41 | 0.199 | 1.16E-11 |
| sp|Q9Y2V7|COG6_HUMAN | 73727.73906 | 0.73 | 0.171 | 1.32E-07 |
| sp|Q9ULR0|ISY1_HUMAN | 33010.92351 | 1.28 | 0.167 | 2.95E-09 |
| sp|P62308|RUXG_HUMAN | 8529.438063 | 1.11 | 0.189 | 0.01728 |
| sp|Q969P0|IGSF8_HUMAN | 65602.73693 | 0.66 | 0.182 | 5.44E-08 |
| sp|Q96CU9|FXRD1_HUMAN | 54101.78754 | 0.73 | 0.16 | 8.73E-08 |
| sp|O94929|ABLM3_HUMAN | 79444.2542 | 1.89 | 0.589 | 6.54E-10 |
| sp|P14866|HNRPL_HUMAN | 64701.58931 | 1.37 | 0.131 | 1.10E-14 |
| sp|Q15293|RCN1_HUMAN | 38848.15404 | 1.53 | 0.226 | 1.91E-13 |
| sp|O75695|XRP2_HUMAN | 40452.76842 | 0.74 | 0.104 | 7.38E-11 |
| sp|P78539|SRPX_HUMAN | 52774.58126 | 1.3 | 0.694 | 0.08047 |
| sp|O75891|AL1L1_HUMAN | 99603.8764 | 1.99 | 0.446 | 3.24E-13 |
| sp|Q5W0U4|BSPRY_HUMAN | 45019.5483 | 0.76 | 0.157 | 1.05E-06 |
| sp|Q13310|PABP4_HUMAN | 71062.20904 | 1.43 | 0.162 | 6.53E-14 |
| sp|P09914|IFIT1_HUMAN | 55763.38181 | 1.15 | 0.257 | 0.04617 |
| sp|P04259|K2C6B_HUMAN | 60297.38777 | 1.94 | 1.075 | 5.83E-05 |
| sp|P80365|DHI2_HUMAN | 44593.56915 | 0.72 | 0.266 | 0.0002976 |
| sp|Q96AG4|LRC59_HUMAN | 35290.0162 | 0.95 | 0.207 | 0.1149 |
| sp|Q9UFG5|CS025_HUMAN | 12908.60149 | 1.36 | 0.199 | 2.45E-10 |
| sp|P05997|CO5A2_HUMAN | 145772.3292 | 2.35 | 0.968 | 2.49E-10 |
| sp|Q14156|EFR3A_HUMAN | 93759.22044 | 0.76 | 0.083 | 2.81E-12 |
| sp|Q8IZP0|ABI1_HUMAN | 55142.74967 | 1.14 | 0.115 | 2.70E-06 |
| sp|Q9Y281|COF2_HUMAN | 18820.88158 | 0.95 | 0.25 | 0.1313 |
| sp|P49840|GSK3A_HUMAN | 51387.1594 | 0.87 | 0.196 | 0.0009992 |
| sp|Q08397|LOXL1_HUMAN | 63736.80446 | 1.44 | 0.612 | 0.0005719 |
| sp|Q969U7|PSMG2_HUMAN | 29758.16611 | 0.59 | 0.294 | 2.44E-07 |
| sp|Q9UJY1|HSPB8_HUMAN | 21743.68217 | 1.13 | 0.322 | 0.1336 |
| sp|P19174|PLCG1_HUMAN | 149731.3587 | 0.88 | 0.158 | 0.0009375 |
| sp|P80303|NUCB2_HUMAN | 50287.39082 | 1.11 | 0.202 | 0.03368 |
| sp|P19256|LFA3_HUMAN | 28738.10052 | 0.88 | 0.168 | 0.0006731 |
| sp|Q9UG63|ABCF2_HUMAN | 71796.82626 | 1 | 0.146 | 0.6384 |
| sp|Q9NVA1|UQCC1_HUMAN | 34958.74538 | 0.48 | 0.118 | 1.25E-13 |
| sp|Q96GM8|TOE1_HUMAN | 57349.29879 | 1.68 | 0.334 | 1.23E-12 |
| sp|P49908|SEPP1_HUMAN | 44107.68796 | 1.21 | 0.36 | 0.02421 |
| sp|P30101|PDIA3_HUMAN | 57127.88717 | 1.29 | 0.233 | 8.65E-07 |
| sp|Q9H270|VPS11_HUMAN | 109061.9043 | 0.86 | 0.033 | 6.69E-16 |
| sp|Q13751|LAMB3_HUMAN | 133348.0527 | 1.17 | 0.393 | 0.1372 |
| sp|Q9NY59|NSMA2_HUMAN | 72329.43765 | 0.7 | 0.357 | 0.0001303 |
| sp|Q01844|EWS_HUMAN | 68702.79996 | 1.39 | 0.155 | 1.53E-13 |
| sp|Q96JC1|VPS39_HUMAN | 102866.5836 | 0.72 | 0.075 | 2.47E-14 |
| sp|Q9BXV9|GON7_HUMAN | 10891.08833 | 0.98 | 0.237 | 0.3123 |
| sp|Q9NVT9|ARMC1_HUMAN | 31470.8604 | 0.92 | 0.108 | 0.000473 |
| sp|Q5XXA6|ANO1_HUMAN | 115012.911 | 0.62 | 0.229 | 2.20E-06 |
| sp|P63151|2ABA_HUMAN | 52154.66936 | 1.32 | 0.121 | 1.03E-13 |
| sp|Q86WR0|CCD25_HUMAN | 24616.1676 | 1.21 | 0.229 | 0.0001359 |
| sp|Q14204|DYHC1_HUMAN | 534790.8596 | 0.56 | 0.062 | 2.20E-16 |
| sp|Q16875|F263_HUMAN | 60351.51405 | 1.04 | 0.199 | 0.535 |
| sp|Q7Z6M1|RABEK_HUMAN | 41147.81243 | 1 | 0.063 | 0.6691 |
| sp|Q9Y2H0|DLGP4_HUMAN | 108896.8069 | 0.99 | 0.217 | 0.4063 |
| sp|P63096|GNAI1_HUMAN | 40887.4338 | 0.49 | 0.18 | 7.05E-11 |
| sp|Q12913|PTPRJ_HUMAN | 147030.1188 | 0.81 | 0.274 | 0.0005957 |
| sp|Q9UPT9|UBP22_HUMAN | 61385.97282 | 0.77 | 0.216 | 2.60E-05 |
| sp|Q9H0W5|CCDC8_HUMAN | 59434.90159 | 1.28 | 0.315 | 0.0004955 |
| sp|Q9NV96|CC50A_HUMAN | 41038.76834 | 0.55 | 0.15 | 3.02E-11 |
| sp|O95544|NADK_HUMAN | 49862.80586 | 0.96 | 0.187 | 0.1594 |
| sp|P16949|STMN1_HUMAN | 17273.9431 | 1.54 | 0.393 | 8.79E-08 |
| sp|O15400|STX7_HUMAN | 29893.35546 | 1.12 | 0.27 | 0.1112 |
| sp|O15111|IKKA_HUMAN | 85707.3804 | 0.42 | 0.06 | 2.20E-16 |
| sp|Q99996|AKAP9_HUMAN | 454970.5627 | 1.14 | 0.167 | 0.0003349 |
| sp|P61081|UBC12_HUMAN | 21153.74548 | 0.94 | 0.11 | 0.008196 |
| sp|P09382|LEG1_HUMAN | 15030.31755 | 1.51 | 0.382 | 1.32E-07 |
| sp|O43520|AT8B1_HUMAN | 144783.5691 | 0.47 | 0.169 | 2.15E-11 |
| sp|P60866|RS20_HUMAN | 13460.34084 | 1.34 | 0.12 | 2.11E-14 |
| sp|Q96EM0|T3HPD_HUMAN | 38551.51289 | 1.06 | 0.287 | 0.5704 |
| sp|P49720|PSB3_HUMAN | 23200.5479 | 0.6 | 0.184 | 1.05E-09 |
| sp|Q8WTS6|SETD7_HUMAN | 41019.23479 | 1.65 | 0.411 | 5.24E-09 |
| sp|P05109|S10A8_HUMAN | 10866.66013 | 0.56 | 0.361 | 5.59E-06 |
| sp|P78347|GTF2I_HUMAN | 112841.2593 | 1.15 | 0.192 | 0.0007126 |
| sp|Q9NR12|PDLI7_HUMAN | 50878.00049 | 0.72 | 0.24 | 1.40E-05 |
| sp|Q9ULZ3|ASC_HUMAN | 21652.27281 | 1.03 | 0.255 | 0.975 |
| sp|Q9Y2T3|GUAD_HUMAN | 51465.74996 | 0.9 | 0.433 | 0.02647 |
| sp|Q14974|IMB1_HUMAN | 98401.49787 | 0.68 | 0.07 | 5.29E-16 |
| sp|Q01968|OCRL_HUMAN | 105374.4188 | 0.78 | 0.08 | 5.81E-12 |
| sp|Q9UBI1|COMD3_HUMAN | 22347.23233 | 1.1 | 0.156 | 0.00815 |
| sp|Q9UPN4|CP131_HUMAN | 122513.2991 | 1.56 | 0.386 | 4.18E-09 |
| sp|Q5T8P6|RBM26_HUMAN | 113908.629 | 1.38 | 0.215 | 8.72E-10 |
| sp|P36639|8ODP_HUMAN | 22601.14909 | 1.48 | 0.307 | 4.19E-09 |
| sp|O60573|IF4E2_HUMAN | 28440.22045 | 0.93 | 0.159 | 0.0106 |
| sp|P63104|1433Z_HUMAN | 27880.78224 | 1.04 | 0.173 | 0.4723 |
| sp|P18754|RCC1_HUMAN | 45378.71057 | 1.09 | 0.234 | 0.1722 |
| sp|Q9BUB7|TMM70_HUMAN | 29103.93125 | 0.72 | 0.188 | 8.77E-07 |
| sp|O15460|P4HA2_HUMAN | 61244.764 | 1.66 | 0.565 | 7.91E-07 |
| sp|Q96LT7|CI072_HUMAN | 54788.18109 | 2.54 | 2.263 | 0.006608 |
| sp|P08311|CATG_HUMAN | 29143.18783 | 0.89 | 0.43 | 0.02735 |
| sp|Q9P278|FNIP2_HUMAN | 123616.3891 | 0.88 | 0.235 | 0.004966 |
| sp|Q6NUJ1|SAPL1_HUMAN | 58452.83167 | 0.62 | 0.286 | 0.0002721 |
| sp|Q96PZ7|CSMD1_HUMAN | 396566.6002 | 0.89 | 0.254 | 0.01466 |
| sp|Q15233|NONO_HUMAN | 54293.31336 | 1.3 | 0.146 | 3.88E-11 |
| sp|Q14764|MVP_HUMAN | 99533.03069 | 1.15 | 0.17 | 0.0001568 |
| sp|Q14914|PTGR1_HUMAN | 36056.80428 | 0.6 | 0.242 | 5.30E-07 |
| sp|Q9NP79|VTA1_HUMAN | 34124.98932 | 1.03 | 0.165 | 0.6649 |
| sp|Q15165|PON2_HUMAN | 39509.34223 | 1.39 | 0.356 | 5.85E-06 |
| sp|Q96FN4|CPNE2_HUMAN | 61816.85307 | 0.89 | 0.16 | 0.001278 |
| sp|Q7L592|NDUF7_HUMAN | 49416.55083 | 0.75 | 0.226 | 8.44E-06 |
| sp|Q9Y4R8|TELO2_HUMAN | 92412.41676 | 0.75 | 0.109 | 3.65E-10 |
| sp|P28074|PSB5_HUMAN | 28615.26354 | 0.66 | 0.069 | 2.20E-16 |
| sp|P30479|1B41_HUMAN | 40838.16779 | 0.86 | 0.379 | 0.01309 |
| sp|Q9P2R7|SUCB1_HUMAN | 50609.32961 | 0.87 | 0.18 | 0.001357 |
| sp|O14498|ISLR_HUMAN | 46577.59239 | 1.36 | 0.29 | 3.89E-07 |
| sp|P11831|SRF_HUMAN | 51599.78946 | 1.34 | 0.376 | 4.44E-05 |
| sp|Q6P9B9|INT5_HUMAN | 109220.7595 | 0.74 | 0.078 | 7.01E-14 |
| sp|P17655|CAN2_HUMAN | 80782.10488 | 0.78 | 0.159 | 9.84E-07 |
| sp|P48556|PSMD8_HUMAN | 39853.92423 | 0.85 | 0.133 | 9.59E-06 |
| sp|P01782|HV309_HUMAN | 13089.49178 | 1.18 | 0.36 | 0.04882 |
| sp|Q9NZU7|CABP1_HUMAN | 40080.07056 | 0.9 | 0.152 | 0.002292 |
| sp|P12277|KCRB_HUMAN | 42884.41497 | 0.73 | 0.352 | 0.0001013 |
| sp|Q5EB52|MEST_HUMAN | 38844.52472 | 0.59 | 0.155 | 2.43E-10 |
| sp|P55957|BID_HUMAN | 22134.09953 | 0.95 | 0.178 | 0.1064 |
| sp|Q9BWU0|NADAP_HUMAN | 89482.28152 | 1.19 | 0.207 | 4.85E-05 |
| sp|O95372|LYPA2_HUMAN | 25044.80923 | 1.05 | 0.181 | 0.2717 |
| sp|P50440|GATM_HUMAN | 48919.52728 | 1.07 | 0.425 | 0.8395 |
| sp|O43166|SI1L1_HUMAN | 201083.6678 | 0.93 | 0.108 | 0.001257 |
| sp|Q8IVN8|SBSPO_HUMAN | 30998.04217 | 0.48 | 0.452 | 5.10E-07 |
| sp|P12694|ODBA_HUMAN | 50706.28929 | 1.17 | 0.172 | 0.0001176 |
| sp|Q9HB07|MYG1_HUMAN | 42746.52022 | 1.13 | 0.098 | 2.49E-07 |
| sp|P14384|CBPM_HUMAN | 50919.65266 | 1.04 | 0.128 | 0.2816 |
| sp|Q6ZUT6|CCD9B_HUMAN | 57785.00763 | 0.5 | 0.459 | 1.35E-06 |
| sp|Q8IX19|MCEM1_HUMAN | 21425.03035 | 0.45 | 0.272 | 7.66E-08 |
| sp|P43353|AL3B1_HUMAN | 52415.78539 | 0.72 | 0.166 | 1.68E-07 |
| sp|Q86V15|CASZ1_HUMAN | 193237.1748 | 0.98 | 0.108 | 0.1908 |
| sp|C9JLW8|MCRI1_HUMAN | 10895.6023 | 1.77 | 0.185 | 2.20E-16 |
| sp|P48147|PPCE_HUMAN | 81542.36209 | 0.67 | 0.131 | 2.28E-10 |
| sp|P01825|HV459_HUMAN | 13023.64449 | 0.82 | 0.257 | 0.001023 |
| sp|Q12904|AIMP1_HUMAN | 34598.2088 | 1.26 | 0.177 | 3.73E-08 |
| sp|Q9NZ01|TECR_HUMAN | 36391.91595 | 0.4 | 0.138 | 7.79E-14 |
| sp|Q14451|GRB7_HUMAN | 60480.66353 | 0.88 | 0.387 | 0.02117 |
| sp|Q96I24|FUBP3_HUMAN | 61926.33143 | 1.28 | 0.144 | 1.63E-10 |
| sp|P07602|SAP_HUMAN | 59880.52578 | 0.87 | 0.255 | 0.006176 |
| sp|Q99836|MYD88_HUMAN | 33707.22719 | 0.82 | 0.146 | 2.49E-06 |
| sp|Q5ST30|SYVM_HUMAN | 119537.1959 | 0.64 | 0.112 | 9.77E-12 |
| sp|O95817|BAG3_HUMAN | 61709.92894 | 1.33 | 0.307 | 1.70E-05 |
| sp|Q16775|GLO2_HUMAN | 34222.47892 | 1.17 | 0.097 | 1.76E-09 |
| sp|Q9UKN1|MUC12_HUMAN | 558725.5669 | 0.7 | 0.205 | 1.37E-06 |
| sp|O95155|UBE4B_HUMAN | 147442.3608 | 0.67 | 0.087 | 5.28E-14 |
| sp|Q96PE7|MCEE_HUMAN | 18833.10057 | 1.24 | 0.264 | 6.70E-05 |
| sp|Q96QP1|ALPK1_HUMAN | 140181.7416 | 0.92 | 0.255 | 0.03602 |
| sp|Q96ST3|SIN3A_HUMAN | 145864.8791 | 0.96 | 0.109 | 0.06086 |
| sp|Q5SYB0|FRPD1_HUMAN | 175704.7259 | 0.67 | 0.118 | 1.97E-11 |
| sp|Q15057|ACAP2_HUMAN | 88924.55791 | 0.74 | 0.1 | 2.27E-11 |
| sp|Q70UQ0|IKIP_HUMAN | 39380.99198 | 1.82 | 0.644 | 2.47E-07 |
| sp|P30405|PPIF_HUMAN | 22350.31553 | 1.34 | 0.438 | 0.002482 |
| sp|P14555|PA2GA_HUMAN | 16852.07631 | 1.17 | 0.817 | 0.7488 |
| sp|Q8NHG8|ZNRF2_HUMAN | 24594.69005 | 1.01 | 0.285 | 0.6198 |
| sp|Q96C01|F136A_HUMAN | 16125.84308 | 1.12 | 0.339 | 0.2038 |
| sp|Q86UT6|NLRX1_HUMAN | 108556.3974 | 0.83 | 0.123 | 1.22E-06 |
| sp|Q07889|SOS1_HUMAN | 153319.4805 | 0.76 | 0.131 | 1.26E-08 |
| sp|Q8IXB3|TARG1_HUMAN | 19338.19162 | 6.33 | 2.515 | 2.20E-16 |
| sp|Q9UKA4|AKA11_HUMAN | 212928.3613 | 0.81 | 0.128 | 4.11E-07 |
| sp|Q8N5M9|JAGN1_HUMAN | 21093.10539 | 0.31 | 0.147 | 1.03E-12 |
| sp|Q9H981|ARP8_HUMAN | 71275.95101 | 0.9 | 0.149 | 0.0007379 |
| sp|Q96F63|CCD97_HUMAN | 39246.78845 | 1.02 | 0.098 | 0.2839 |
| sp|O96000|NDUBA_HUMAN | 21030.31028 | 1.25 | 0.272 | 0.0001865 |
| sp|Q969S8|HDA10_HUMAN | 72179.77742 | 1.1 | 0.199 | 0.02898 |
| sp|Q6ZUX7|LHPL2_HUMAN | 25135.56877 | 1.1 | 0.292 | 0.2833 |
| sp|Q86W92|LIPB1_HUMAN | 114504.6092 | 1.65 | 0.386 | 8.87E-10 |
| sp|P22792|CPN2_HUMAN | 61355.44083 | 0.68 | 0.246 | 3.44E-06 |
| sp|P35219|CAH8_HUMAN | 33219.62176 | 0.48 | 0.359 | 1.80E-07 |
| sp|Q9BSB4|ATGA1_HUMAN | 25253.80201 | 0.99 | 0.12 | 0.4296 |
| sp|Q96RL7|VP13A_HUMAN | 361854.1221 | 0.54 | 0.089 | 1.37E-15 |
| sp|P67775|PP2AA_HUMAN | 36123.60075 | 0.63 | 0.204 | 1.65E-08 |
| sp|Q9UHB9|SRP68_HUMAN | 71180.74407 | 0.94 | 0.118 | 0.01108 |
| sp|P10176|COX8A_HUMAN | 7613.223744 | 0.84 | 0.121 | 1.36E-06 |
| sp|P02790|HEMO_HUMAN | 52366.54149 | 1.16 | 0.284 | 0.0232 |
| sp|Q8N108|MIER1_HUMAN | 58271.78562 | 1.37 | 0.229 | 3.37E-09 |
| sp|P02750|A2GL_HUMAN | 38364.1864 | 1.33 | 0.392 | 0.0001725 |
| sp|Q07002|CDK18_HUMAN | 54714.13135 | 0.98 | 0.27 | 0.3534 |
| sp|P78310|CXAR_HUMAN | 40556.57871 | 0.48 | 0.199 | 2.40E-10 |
| sp|Q6PJ69|TRI65_HUMAN | 58724.37183 | 1.1 | 0.15 | 0.005059 |
| sp|Q9ULI1|NWD2_HUMAN | 200173.2929 | 1.51 | 0.352 | 4.66E-08 |
| sp|Q99418|CYH2_HUMAN | 46840.72096 | 0.89 | 0.128 | 0.0003888 |
| sp|Q99496|RING2_HUMAN | 38069.78978 | 1.11 | 0.119 | 7.90E-05 |
| sp|Q07866|KLC1_HUMAN | 65764.05453 | 1.39 | 0.149 | 5.91E-14 |
| sp|Q8TDN4|CABL1_HUMAN | 68222.96471 | 0.99 | 0.159 | 0.4491 |
| sp|Q86WC4|OSTM1_HUMAN | 38012.90265 | 1.08 | 0.156 | 0.02462 |
| sp|P61803|DAD1_HUMAN | 12641.60835 | 0.53 | 0.122 | 2.40E-13 |
| sp|O00160|MYO1F_HUMAN | 125489.0997 | 0.82 | 0.189 | 8.40E-05 |
| sp|Q9NXR7|BABA2_HUMAN | 43961.98941 | 0.7 | 0.067 | 1.03E-15 |
| sp|Q8IYB3|SRRM1_HUMAN | 102313.1396 | 1.59 | 0.407 | 9.73E-09 |
| sp|P01920|DQB1_HUMAN | 30182.27348 | 1.06 | 0.277 | 0.6943 |
| sp|P78318|IGBP1_HUMAN | 39293.53507 | 1.12 | 0.123 | 2.93E-05 |
| sp|Q13361|MFAP5_HUMAN | 20037.00407 | 1.14 | 0.245 | 0.01333 |
| sp|P00973|OAS1_HUMAN | 46608.57475 | 0.61 | 0.498 | 1.03E-05 |
| sp|Q9UBQ5|EIF3K_HUMAN | 25310.53678 | 0.89 | 0.134 | 0.0004354 |
| sp|P19634|SL9A1_HUMAN | 91200.21659 | 0.91 | 0.216 | 0.03862 |
| sp|O43310|CTIF_HUMAN | 68096.60106 | 0.91 | 0.611 | 0.03311 |
| sp|Q92623|TTC9A_HUMAN | 24687.63222 | 1.36 | 0.338 | 8.40E-06 |
| sp|P43251|BTD_HUMAN | 61987.59136 | 0.71 | 0.103 | 1.16E-11 |
| sp|P63272|SPT4H_HUMAN | 13451.60275 | 1.49 | 0.37 | 1.14E-07 |
| sp|P13671|CO6_HUMAN | 108349.368 | 0.89 | 0.277 | 0.01867 |
| sp|O00159|MYO1C_HUMAN | 122443.2276 | 0.83 | 0.174 | 2.05E-05 |
| sp|Q01196|RUNX1_HUMAN | 48859.28157 | 1.37 | 0.315 | 1.04E-06 |
| sp|Q12888|TP53B_HUMAN | 215477.4503 | 1.62 | 0.118 | 2.20E-16 |
| sp|Q96I59|SYNM_HUMAN | 54550.89563 | 0.75 | 0.101 | 1.43E-10 |
| sp|O43914|TYOBP_HUMAN | 12324.46913 | 0.85 | 0.246 | 0.00125 |
| sp|P07948|LYN_HUMAN | 58974.95939 | 0.76 | 0.225 | 4.64E-06 |
| sp|Q9Y6K0|CEPT1_HUMAN | 47417.07624 | 0.83 | 0.146 | 1.04E-05 |
| sp|O00273|DFFA_HUMAN | 36880.6428 | 1.48 | 0.233 | 2.81E-11 |
| sp|Q9NVD7|PARVA_HUMAN | 42256.43911 | 0.63 | 0.117 | 2.57E-12 |
| sp|O14896|IRF6_HUMAN | 53647.81666 | 0.59 | 0.156 | 1.62E-10 |
| sp|Q15532|SSXT_HUMAN | 45995.24378 | 1.48 | 0.244 | 1.82E-11 |
| sp|Q9NY27|PP4R2_HUMAN | 47250.00657 | 0.95 | 0.214 | 0.08328 |
| sp|Q9NRF2|SH2B1_HUMAN | 79755.66862 | 1.29 | 0.151 | 5.50E-11 |
| sp|P20742|PZP_HUMAN | 165224.1304 | 0.61 | 0.214 | 1.70E-08 |
| sp|P51617|IRAK1_HUMAN | 77439.9114 | 0.89 | 0.12 | 8.87E-05 |
| sp|Q15014|MO4L2_HUMAN | 32326.77596 | 1.01 | 0.382 | 0.4054 |
| sp|P20815|CP3A5_HUMAN | 57339.20282 | 0.79 | 0.274 | 0.002127 |
| sp|Q9NPA8|ENY2_HUMAN | 11617.34564 | 1.52 | 0.543 | 1.30E-05 |
| sp|Q5JTB6|PLAC9_HUMAN | 10398.27754 | 1.58 | 0.99 | 0.007865 |
| sp|Q9NQG5|RPR1B_HUMAN | 36973.2179 | 1.16 | 0.232 | 0.007578 |
| sp|Q96ME7|ZN512_HUMAN | 65706.49115 | 1.27 | 0.247 | 3.61E-06 |
| sp|Q9H694|BICC1_HUMAN | 105387.1159 | 1.99 | 0.601 | 2.56E-10 |
| sp|O75569|PRKRA_HUMAN | 34820.70738 | 1.14 | 0.154 | 0.0001478 |
| sp|Q8TCT9|HM13_HUMAN | 41728.64551 | 0.56 | 0.148 | 3.33E-11 |
| sp|O60502|OGA_HUMAN | 103914.2393 | 1.14 | 0.19 | 0.002864 |
| sp|Q7Z4I7|LIMS2_HUMAN | 40753.26245 | 0.61 | 0.059 | 2.20E-16 |
| sp|P07384|CAN1_HUMAN | 82447.41368 | 0.56 | 0.09 | 6.82E-16 |
| sp|O15427|MOT4_HUMAN | 50046.11677 | 0.39 | 0.146 | 3.97E-12 |
| sp|Q9H4L4|SENP3_HUMAN | 65577.91401 | 0.65 | 0.11 | 2.66E-12 |
| sp|Q96S82|UBL7_HUMAN | 40524.32484 | 1.18 | 0.223 | 0.0006872 |
| sp|Q9Y5U9|IR3IP_HUMAN | 9001.949884 | 0.39 | 0.055 | 2.20E-16 |
| sp|Q9H6Z4|RANB3_HUMAN | 60497.3469 | 1.48 | 0.17 | 9.82E-15 |
| sp|Q8IUH4|ZDH13_HUMAN | 71936.5545 | 0.46 | 0.108 | 3.05E-15 |
| sp|P84157|MXRA7_HUMAN | 21491.41205 | 1.39 | 0.518 | 0.001043 |
| sp|Q9NX70|MED29_HUMAN | 21440.66596 | 1.14 | 0.116 | 2.33E-06 |
| sp|O14828|SCAM3_HUMAN | 38643.46593 | 0.65 | 0.101 | 2.84E-13 |
| sp|Q9GZV4|IF5A2_HUMAN | 17106.35598 | 1.74 | 0.241 | 2.20E-16 |
| sp|Q9NY25|CLC5A_HUMAN | 21831.76982 | 0.76 | 0.323 | 0.0003262 |
| sp|Q9UKF2|ADA30_HUMAN | 91372.6714 | 1 | 0.366 | 0.3538 |
| sp|Q96P48|ARAP1_HUMAN | 163724.9692 | 0.98 | 0.105 | 0.3431 |
| sp|Q7L3T8|SYPM_HUMAN | 54236.50259 | 0.63 | 0.166 | 2.39E-09 |
| sp|Q9HCG8|CWC22_HUMAN | 105953.7121 | 0.32 | 0.088 | 2.20E-16 |
| sp|Q52LW3|RHG29_HUMAN | 143495.7754 | 0.86 | 0.106 | 1.06E-06 |
| sp|P08603|CFAH_HUMAN | 143662.4478 | 0.97 | 0.295 | 0.2251 |
| sp|P11413|G6PD_HUMAN | 59657.15384 | 0.64 | 0.146 | 5.31E-10 |
| sp|Q8TEQ0|SNX29_HUMAN | 91578.27009 | 1.18 | 0.132 | 3.93E-07 |
| sp|Q92979|NEP1_HUMAN | 26913.22698 | 0.57 | 0.066 | 2.20E-16 |
| sp|P61254|RL26_HUMAN | 17229.52174 | 1.5 | 0.253 | 1.97E-11 |
| sp|Q7L0Y3|TM10C_HUMAN | 47583.67569 | 0.91 | 0.181 | 0.008322 |
| sp|Q8N684|CPSF7_HUMAN | 52171.3363 | 1.3 | 0.203 | 3.95E-08 |
| sp|Q9BSE4|HERP2_HUMAN | 45214.71408 | 0.77 | 0.144 | 1.05E-07 |
| sp|Q96QS6|KPSH2_HUMAN | 43152.39218 | 4.92 | 2.626 | 2.70E-13 |
| sp|Q16698|DECR_HUMAN | 36311.89797 | 0.76 | 0.183 | 3.38E-06 |
| sp|O60671|RAD1_HUMAN | 32415.83249 | 0.7 | 0.179 | 5.09E-08 |
| sp|A2A288|ZC12D_HUMAN | 58479.8333 | 1.19 | 0.251 | 0.003147 |
| sp|P24752|THIL_HUMAN | 45437.74025 | 1.36 | 0.406 | 0.0002575 |
| sp|P11215|ITAM_HUMAN | 128392.0041 | 0.67 | 0.283 | 1.35E-05 |
| sp|Q8IU81|I2BP1_HUMAN | 62543.30898 | 1 | 0.187 | 0.651 |
| sp|P01705|LV223_HUMAN | 12038.74996 | 2.47 | 2.224 | 0.01226 |
| sp|Q9UFC0|LRWD1_HUMAN | 72052.65979 | 0.88 | 0.163 | 0.0005318 |
| sp|P54840|GYS2_HUMAN | 81546.94196 | 1.08 | 0.259 | 0.3385 |
| sp|Q15435|PP1R7_HUMAN | 41634.56304 | 1.28 | 0.111 | 3.08E-13 |
| sp|Q8N1G2|CMTR1_HUMAN | 96154.29853 | 0.83 | 0.109 | 1.37E-07 |
| sp|Q00325|MPCP_HUMAN | 40506.89857 | 0.45 | 0.114 | 2.13E-14 |
| sp|Q9NVH6|TMLH_HUMAN | 50095.2766 | 0.95 | 0.195 | 0.08429 |
| sp|Q8TD16|BICD2_HUMAN | 93686.10466 | 1.45 | 0.222 | 9.67E-12 |
| sp|P63167|DYL1_HUMAN | 10512.16184 | 0.92 | 0.22 | 0.02649 |
| sp|Q9BV29|CCD32_HUMAN | 20853.07863 | 1.26 | 0.545 | 0.07646 |
| sp|P0DJ07|PT100_HUMAN | 9089.756812 | 0.81 | 0.372 | 0.002399 |
| sp|P63000|RAC1_HUMAN | 21817.38137 | 0.8 | 0.056 | 2.52E-14 |
| sp|Q9H1B7|I2BPL_HUMAN | 83558.33298 | 1.44 | 0.234 | 2.88E-11 |
| sp|Q5JPI3|CC038_HUMAN | 37898.11939 | 1 | 0.165 | 0.6039 |
| sp|Q9H665|IGFR1_HUMAN | 38764.49481 | 1.06 | 0.233 | 0.3383 |
| sp|Q8N130|NPT2C_HUMAN | 64289.36355 | 5.55 | 3.145 | 2.61E-11 |
| sp|Q9P2X0|DPM3_HUMAN | 10240.28968 | 0.97 | 0.313 | 0.2064 |
| sp|Q86V21|AACS_HUMAN | 75648.19412 | 0.71 | 0.173 | 1.08E-07 |
| sp|O43150|ASAP2_HUMAN | 112817.0522 | 0.76 | 0.107 | 1.74E-09 |
| sp|Q6DKJ4|NXN_HUMAN | 48742.773 | 0.77 | 0.137 | 1.25E-07 |
| sp|Q6ICL3|TNG2_HUMAN | 31184.58656 | 1.08 | 0.133 | 0.01418 |
| sp|P42166|LAP2A_HUMAN | 75997.83475 | 1.12 | 0.232 | 0.02769 |
| sp|Q14894|CRYM_HUMAN | 33907.43338 | 0.97 | 0.167 | 0.2473 |
| sp|P41227|NAA10_HUMAN | 26594.90534 | 1.44 | 0.17 | 2.25E-13 |
| sp|Q9NX08|COMD8_HUMAN | 21173.03365 | 0.91 | 0.122 | 0.001065 |
| sp|O00533|NCHL1_HUMAN | 136052.3812 | 1.07 | 0.247 | 0.3794 |
| sp|Q00688|FKBP3_HUMAN | 25200.32787 | 1.63 | 0.275 | 2.36E-13 |
| sp|Q16790|CAH9_HUMAN | 49934.16835 | 0.78 | 0.291 | 0.001994 |
| sp|O43432|IF4G3_HUMAN | 177664.4798 | 0.99 | 0.197 | 0.5196 |
| sp|Q9Y2W2|WBP11_HUMAN | 69935.90968 | 1.5 | 0.331 | 1.10E-08 |
| sp|P62487|RPB7_HUMAN | 19434.92982 | 0.63 | 0.105 | 7.92E-13 |
| sp|P08572|CO4A2_HUMAN | 168628.0699 | 1.1 | 0.232 | 0.09362 |
| sp|P10620|MGST1_HUMAN | 17626.22984 | 0.61 | 0.14 | 4.43E-11 |
| sp|Q8NB14|UBP38_HUMAN | 117822.5694 | 1.14 | 0.348 | 0.1963 |
| sp|Q9NYL9|TMOD3_HUMAN | 39723.35381 | 1.51 | 0.193 | 1.71E-14 |
| sp|P35580|MYH10_HUMAN | 229809.308 | 1.14 | 0.459 | 0.4547 |
| sp|Q8TDP1|RNH2C_HUMAN | 17925.06386 | 1.07 | 0.117 | 0.01421 |
| sp|Q9Y2A9|B3GN3_HUMAN | 43002.12738 | 0.74 | 0.073 | 7.17E-14 |
| sp|Q8IXM3|RM41_HUMAN | 15411.99943 | 1.19 | 0.277 | 0.005053 |
| sp|Q9H4Z3|CAPAM_HUMAN | 81512.70637 | 1.09 | 0.326 | 0.5656 |
| sp|Q9H7H0|MET17_HUMAN | 51253.5137 | 1.13 | 0.174 | 0.002041 |
| sp|Q4KWH8|PLCH1_HUMAN | 191196.0797 | 1.07 | 0.093 | 0.001167 |
| sp|Q6P1M3|L2GL2_HUMAN | 114328.5238 | 0.76 | 0.189 | 1.47E-06 |
| sp|O43175|SERA_HUMAN | 57337.67285 | 1.39 | 0.334 | 3.11E-06 |
| sp|Q9BSD7|NTPCR_HUMAN | 20910.18817 | 1.12 | 0.168 | 0.003285 |
| sp|Q96IX5|USMG5_HUMAN | 6492.38406 | 0.48 | 0.091 | 2.20E-16 |
| sp|Q99543|DNJC2_HUMAN | 72446.8075 | 1.11 | 0.227 | 0.07724 |
| sp|Q9NZM5|NOP53_HUMAN | 54452.45871 | 1.41 | 0.327 | 5.46E-07 |
| sp|P30414|NKTR_HUMAN | 166585.245 | 1.38 | 0.303 | 1.52E-06 |
| sp|O75116|ROCK2_HUMAN | 161921.2435 | 0.91 | 0.115 | 0.0004459 |
| sp|Q15853|USF2_HUMAN | 37028.23345 | 1.39 | 0.196 | 5.37E-11 |
| sp|Q9H6F5|CCD86_HUMAN | 40307.35537 | 1 | 0.136 | 0.7892 |
| sp|P83731|RL24_HUMAN | 17863.92916 | 1.29 | 0.302 | 7.98E-05 |
| sp|Q9P2E9|RRBP1_HUMAN | 152745.8206 | 1.52 | 0.331 | 5.41E-09 |
| sp|O95373|IPO7_HUMAN | 120733.3535 | 0.72 | 0.072 | 1.34E-14 |
| sp|Q06787|FMR1_HUMAN | 71454.98516 | 1.16 | 0.173 | 0.0001441 |
| sp|Q9H3U7|SMOC2_HUMAN | 50936.0246 | 1.43 | 0.543 | 0.01466 |
| sp|Q8IXM6|NRM_HUMAN | 29513.13966 | 0.61 | 0.116 | 9.44E-13 |
| sp|P30519|HMOX2_HUMAN | 36162.88346 | 1.19 | 0.092 | 8.37E-11 |
| sp|P46779|RL28_HUMAN | 15776.68184 | 1.44 | 0.222 | 2.94E-11 |
| sp|O00148|DX39A_HUMAN | 49593.13799 | 0.63 | 0.148 | 5.48E-10 |
| sp|P24821|TENA_HUMAN | 246327.264 | 0.88 | 0.496 | 0.02121 |
| sp|Q32P44|EMAL3_HUMAN | 96202.90693 | 1.02 | 0.088 | 0.453 |
| sp|Q9UJV3|TRIM1_HUMAN | 85019.95519 | 0.85 | 0.091 | 3.42E-08 |
| sp|Q16651|PRSS8_HUMAN | 37074.50207 | 0.71 | 0.292 | 6.31E-06 |
| sp|Q6NXE6|ARMC6_HUMAN | 55172.75868 | 1.15 | 0.105 | 8.39E-08 |
| sp|Q14151|SAFB2_HUMAN | 107903.2609 | 1.29 | 0.125 | 2.56E-12 |
| sp|O60476|MA1A2_HUMAN | 73167.57018 | 0.7 | 0.076 | 1.04E-14 |
| sp|P31949|S10AB_HUMAN | 11828.85746 | 1.61 | 0.279 | 2.24E-12 |
| sp|P28290|ITPI2_HUMAN | 139536.0822 | 1.17 | 0.099 | 5.20E-09 |
| sp|P62136|PP1A_HUMAN | 38211.07265 | 0.84 | 0.191 | 0.0002043 |
| sp|Q9BU23|LMF2_HUMAN | 79914.56927 | 0.73 | 0.078 | 8.54E-14 |
| sp|Q13588|GRAP_HUMAN | 25416.80552 | 0.91 | 0.225 | 0.01699 |
| sp|Q14141|SEPT6_HUMAN | 50066.48104 | 1.19 | 0.371 | 0.03611 |
| sp|Q9UQC2|GAB2_HUMAN | 74906.54904 | 1.24 | 0.31 | 0.0008223 |
| sp|Q9GZP9|DERL2_HUMAN | 27645.32154 | 0.55 | 0.3 | 1.36E-06 |
| sp|P08514|ITA2B_HUMAN | 114427.9335 | 0.81 | 0.417 | 0.005501 |
| sp|Q15080|NCF4_HUMAN | 39103.29989 | 0.78 | 0.338 | 0.0006832 |
| sp|Q6PG37|ZN790_HUMAN | 76671.74905 | 1.29 | 0.393 | 0.001672 |
| sp|Q9BVI4|NOC4L_HUMAN | 58811.84119 | 0.68 | 0.111 | 1.43E-11 |
| sp|O43707|ACTN4_HUMAN | 105226.6349 | 0.76 | 0.114 | 3.14E-09 |
| sp|Q6VN20|RBP10_HUMAN | 67994.58475 | 1.23 | 0.219 | 5.80E-06 |
| sp|Q13439|GOGA4_HUMAN | 261874.0721 | 1.17 | 0.165 | 3.02E-05 |
| sp|Q5T447|HECD3_HUMAN | 98116.84819 | 0.82 | 0.087 | 6.53E-10 |
| sp|Q9UDW1|QCR9_HUMAN | 7285.815428 | 0.35 | 0.144 | 4.88E-12 |
| sp|P23229|ITA6_HUMAN | 127705.6226 | 0.77 | 0.155 | 2.63E-07 |
| sp|O60493|SNX3_HUMAN | 18789.76841 | 1.38 | 0.221 | 5.81E-10 |
| sp|P03950|ANGI_HUMAN | 16863.55728 | 1.28 | 0.214 | 1.19E-07 |
| sp|P33316|DUT_HUMAN | 26813.69929 | 1.36 | 0.233 | 7.30E-09 |
| sp|Q9HAW8|UD110_HUMAN | 60550.48646 | 1.17 | 1.208 | 0.2379 |
| sp|Q9P1F3|ABRAL_HUMAN | 9089.874345 | 1.11 | 0.352 | 0.46 |
| sp|Q92581|SL9A6_HUMAN | 74609.09771 | 1.13 | 0.265 | 0.07261 |
| sp|Q9NYU2|UGGG1_HUMAN | 177801.2798 | 0.68 | 0.105 | 1.44E-12 |
| sp|Q5JVS0|HABP4_HUMAN | 45853.59835 | 1.21 | 0.148 | 1.01E-07 |
| sp|Q9BQG0|MBB1A_HUMAN | 149712.9818 | 0.72 | 0.137 | 2.35E-09 |
| sp|P00739|HPTR_HUMAN | 39499.8623 | 1.2 | 0.494 | 0.2338 |
| sp|Q9NPF8|ADAP2_HUMAN | 44759.03244 | 1.08 | 0.145 | 0.02167 |
| sp|Q8IYD1|ERF3B_HUMAN | 69448.80999 | 1.16 | 0.285 | 0.02444 |
| sp|Q13148|TADBP_HUMAN | 45035.42453 | 1.69 | 0.499 | 8.90E-09 |
| sp|O95983|MBD3_HUMAN | 33033.4051 | 1.35 | 0.14 | 2.92E-13 |
| sp|P42704|LPPRC_HUMAN | 158984.5565 | 0.57 | 0.148 | 8.25E-11 |
| sp|P23381|SYWC_HUMAN | 53455.72504 | 1.82 | 1.582 | 0.0135 |
| sp|Q8TB22|SPT20_HUMAN | 88566.39891 | 0.95 | 0.2 | 0.08777 |
| sp|Q969M3|YIPF5_HUMAN | 28351.75739 | 0.63 | 0.229 | 2.41E-07 |
| sp|Q96CW5|GCP3_HUMAN | 104286.381 | 0.5 | 0.069 | 2.20E-16 |
| sp|Q5T2T1|MPP7_HUMAN | 65636.17392 | 1.18 | 0.23 | 0.0005853 |
| sp|Q9BQC6|RT63_HUMAN | 12240.50295 | 1.09 | 0.18 | 0.03436 |
| sp|Q9P2E3|ZNFX1_HUMAN | 225084.0875 | 0.7 | 0.159 | 9.73E-09 |
| sp|Q15084|PDIA6_HUMAN | 48472.40173 | 1.19 | 0.241 | 0.0006929 |
| sp|Q86TI0|TBCD1_HUMAN | 134293.2972 | 0.94 | 0.138 | 0.01703 |
| sp|Q16695|H31T_HUMAN | 15594.54531 | 1.56 | 0.786 | 0.001616 |
| sp|Q9Y3D3|RT16_HUMAN | 15545.07875 | 0.87 | 0.387 | 0.01953 |
| sp|Q9H6U8|ALG9_HUMAN | 70598.12742 | 0.81 | 0.133 | 4.89E-07 |
| sp|O14646|CHD1_HUMAN | 197689.1308 | 0.87 | 0.132 | 6.22E-05 |
| sp|Q9HB58|SP110_HUMAN | 79581.83218 | 0.78 | 0.169 | 2.77E-06 |
| sp|P98172|EFNB1_HUMAN | 38363.63066 | 0.99 | 0.204 | 0.4573 |
| sp|Q5T0U0|CC122_HUMAN | 32396.54658 | 0.95 | 0.13 | 0.04472 |
| sp|Q9UJX2|CDC23_HUMAN | 69570.28025 | 0.85 | 0.165 | 6.93E-05 |
| sp|Q6NXG1|ESRP1_HUMAN | 76431.44383 | 0.87 | 0.26 | 0.005947 |
| sp|Q9H0R4|HDHD2_HUMAN | 28728.01817 | 0.92 | 0.145 | 0.00623 |
| sp|Q8TCT8|SPP2A_HUMAN | 58998.64565 | 0.74 | 0.183 | 1.81E-07 |
| sp|Q9P260|RELCH_HUMAN | 135439.4173 | 0.87 | 0.252 | 0.003133 |
| sp|P04844|RPN2_HUMAN | 69337.0035 | 0.65 | 0.102 | 2.81E-13 |
| sp|P23141|EST1_HUMAN | 62748.24941 | 0.86 | 0.226 | 0.001633 |
| sp|Q15287|RNPS1_HUMAN | 34169.69083 | 1.37 | 0.147 | 2.47E-13 |
| sp|P53582|MAP11_HUMAN | 44081.58282 | 1.16 | 0.161 | 2.41E-05 |
| sp|Q99487|PAFA2_HUMAN | 44559.21407 | 0.57 | 0.135 | 1.23E-11 |
| sp|Q96CV9|OPTN_HUMAN | 66262.31332 | 1.64 | 0.245 | 1.83E-14 |
| sp|P40424|PBX1_HUMAN | 46863.86971 | 1.62 | 0.392 | 1.82E-09 |
| sp|Q9Y478|AAKB1_HUMAN | 30516.32553 | 1.1 | 0.219 | 0.04159 |
| sp|Q969V5|MUL1_HUMAN | 40554.77619 | 0.93 | 0.504 | 0.04574 |
| sp|Q9Y5J6|T10B_HUMAN | 11788.82282 | 1.29 | 0.277 | 2.49E-05 |
| sp|Q07020|RL18_HUMAN | 21717.10021 | 1.04 | 0.184 | 0.4785 |
| sp|P06132|DCUP_HUMAN | 41084.94974 | 0.69 | 0.158 | 1.68E-08 |
| sp|Q99633|PRP18_HUMAN | 39988.0054 | 0.74 | 0.215 | 4.81E-06 |
| sp|Q7RTW8|OTOAN_HUMAN | 129516.4708 | 0.88 | 0.497 | 0.02334 |
| sp|P13497|BMP1_HUMAN | 113497.5787 | 0.9 | 0.413 | 0.04206 |
| sp|P02675|FIBB_HUMAN | 56558.50782 | 1.54 | 1.137 | 0.04943 |
| sp|Q96HE7|ERO1A_HUMAN | 55195.36396 | 0.66 | 0.128 | 3.16E-10 |
| sp|Q9BZF9|UACA_HUMAN | 163526.8675 | 1.46 | 0.313 | 2.89E-09 |
| sp|P61587|RND3_HUMAN | 27845.9241 | 1.35 | 0.499 | 0.001022 |
| sp|Q9H9F9|ARP5_HUMAN | 68806.54566 | 1.92 | 0.682 | 3.90E-09 |
| sp|P19447|ERCC3_HUMAN | 90001.30265 | 0.89 | 0.245 | 0.008527 |
| sp|P56134|ATPK_HUMAN | 11006.72896 | 0.58 | 0.215 | 6.29E-08 |
| sp|P47902|CDX1_HUMAN | 28159.32362 | 1.01 | 0.397 | 0.3957 |
| sp|P0CG29|GST2_HUMAN | 27641.63196 | 0.72 | 0.248 | 2.13E-05 |
| sp|Q13480|GAB1_HUMAN | 77177.73396 | 1.76 | 0.435 | 8.70E-11 |
| sp|Q15645|PCH2_HUMAN | 48844.59815 | 0.89 | 0.175 | 0.002168 |
| sp|P49590|SYHM_HUMAN | 57574.93092 | 0.91 | 0.206 | 0.02946 |
| sp|O14561|ACPM_HUMAN | 17559.03812 | 1.47 | 0.395 | 2.34E-06 |
| sp|Q99757|THIOM_HUMAN | 18524.68235 | 1.64 | 0.749 | 0.001497 |
| sp|Q9BQB6|VKOR1_HUMAN | 18603.67478 | 0.68 | 0.066 | 3.09E-16 |
| sp|P60174|TPIS_HUMAN | 31038.79288 | 1.37 | 0.136 | 3.71E-14 |
| sp|Q7L1Q6|BZW1_HUMAN | 48165.80008 | 0.63 | 0.084 | 2.42E-15 |
| sp|Q99623|PHB2_HUMAN | 33257.90558 | 0.7 | 0.1 | 3.19E-12 |
| sp|Q9Y6K9|NEMO_HUMAN | 48776.58716 | 1.43 | 0.158 | 3.72E-14 |
| sp|Q7Z3D6|GLUCM_HUMAN | 67345.32056 | 0.92 | 0.24 | 0.04322 |
| sp|O75592|MYCB2_HUMAN | 521444.7407 | 0.83 | 0.104 | 3.28E-08 |
| sp|P11021|BIP_HUMAN | 72384.46874 | 1.29 | 0.192 | 3.03E-08 |
| sp|A3KMH1|VWA8_HUMAN | 215697.6722 | 0.9 | 0.149 | 0.001502 |
| sp|Q8WVE0|EFMT1_HUMAN | 24871.00576 | 1.75 | 0.159 | 2.20E-16 |
| sp|Q15437|SC23B_HUMAN | 87374.87055 | 0.65 | 0.163 | 1.85E-09 |
| sp|Q9BTU6|P4K2A_HUMAN | 54369.747 | 0.74 | 0.138 | 7.41E-09 |
| sp|Q9P1Y5|CAMP3_HUMAN | 135446.4494 | 0.91 | 0.296 | 0.04978 |
| sp|Q9H3U1|UN45A_HUMAN | 104247.9507 | 0.57 | 0.088 | 8.54E-16 |
| sp|Q9P0U3|SENP1_HUMAN | 73986.94952 | 0.82 | 0.069 | 8.39E-12 |
| sp|O15355|PPM1G_HUMAN | 59900.93414 | 1.43 | 0.165 | 6.40E-14 |
| sp|Q15363|TMED2_HUMAN | 22842.41234 | 0.66 | 0.134 | 1.43E-10 |
| sp|O43933|PEX1_HUMAN | 143785.999 | 0.76 | 0.118 | 1.01E-09 |
| sp|E9PRG8|CK098_HUMAN | 13771.6388 | 2 | 0.787 | 1.59E-07 |
| sp|P06865|HEXA_HUMAN | 61102.43635 | 0.89 | 0.271 | 0.01281 |
| sp|Q9BYT8|NEUL_HUMAN | 81322.96383 | 1 | 0.186 | 0.7527 |
| sp|Q7L5N7|PCAT2_HUMAN | 60778.62132 | 0.59 | 0.053 | 2.20E-16 |
| sp|P31629|ZEP2_HUMAN | 271090.443 | 1.17 | 0.25 | 0.004519 |
| sp|Q9Y3C6|PPIL1_HUMAN | 18321.15077 | 1.06 | 0.152 | 0.1353 |
| sp|P22626|ROA2_HUMAN | 37445.73769 | 1.47 | 0.219 | 1.91E-12 |
| sp|P09871|C1S_HUMAN | 78156.37066 | 1.26 | 0.29 | 0.0003181 |
| sp|Q8NCA5|FA98A_HUMAN | 55804.50314 | 0.95 | 0.105 | 0.02346 |
| sp|O95833|CLIC3_HUMAN | 26898.80838 | 1.41 | 0.364 | 2.49E-06 |
| sp|P35249|RFC4_HUMAN | 40152.07009 | 1.46 | 0.427 | 2.53E-06 |
| sp|O60637|TSN3_HUMAN | 28779.44933 | 0.75 | 0.23 | 5.07E-06 |
| sp|O00462|MANBA_HUMAN | 101782.1524 | 0.61 | 0.187 | 1.71E-08 |
| sp|Q66K74|MAP1S_HUMAN | 113093.3356 | 0.87 | 0.08 | 1.43E-07 |
| sp|Q8IWT3|CUL9_HUMAN | 285422.1073 | 1.15 | 0.157 | 4.61E-05 |
| sp|Q9C005|DPY30_HUMAN | 11224.7922 | 1.08 | 0.198 | 0.1388 |
| sp|P25788|PSA3_HUMAN | 28625.15671 | 0.66 | 0.096 | 1.88E-13 |
| sp|Q6UW02|CP20A_HUMAN | 52723.13152 | 0.61 | 0.147 | 5.76E-11 |
| sp|O43402|EMC8_HUMAN | 24195.82267 | 1.04 | 0.149 | 0.3414 |
| sp|Q9NZN3|EHD3_HUMAN | 60887.72312 | 0.93 | 0.223 | 0.04168 |
| sp|Q16739|CEGT_HUMAN | 45433.07553 | 1 | 0.169 | 0.6897 |
| sp|Q96JD6|AKCL2_HUMAN | 37117.90379 | 0.96 | 0.316 | 0.1752 |
| sp|P49641|MA2A2_HUMAN | 131122.7877 | 0.71 | 0.277 | 2.16E-05 |
| sp|Q01995|TAGL_HUMAN | 22635.44416 | 0.8 | 0.345 | 0.002813 |
| sp|P54819|KAD2_HUMAN | 26670.84719 | 1.2 | 0.155 | 6.80E-07 |
| sp|P07108|ACBP_HUMAN | 10020.01039 | 1.53 | 0.369 | 1.74E-07 |
| sp|Q6UXH1|CREL2_HUMAN | 40314.63838 | 1.75 | 0.468 | 1.01E-10 |
| sp|Q9BQS7|HEPH_HUMAN | 131202.5976 | 0.71 | 0.179 | 6.96E-07 |
| sp|Q9BRJ6|CG050_HUMAN | 22108.82779 | 1.12 | 0.153 | 0.0006889 |
| sp|P55268|LAMB2_HUMAN | 202964.1123 | 0.73 | 0.225 | 3.63E-06 |
| sp|Q8IWW6|RHG12_HUMAN | 96519.62213 | 0.69 | 0.167 | 2.10E-08 |
| sp|Q8NEZ2|VP37A_HUMAN | 44439.57228 | 1.33 | 0.126 | 6.78E-14 |
| sp|Q8N511|TM199_HUMAN | 23211.57059 | 1.56 | 0.575 | 1.06E-05 |
| sp|P60903|S10AA_HUMAN | 11291.54361 | 1.82 | 0.399 | 1.03E-12 |
| sp|O14949|QCR8_HUMAN | 9882.110268 | 0.72 | 0.173 | 1.53E-07 |
| sp|P02753|RET4_HUMAN | 23319.37643 | 0.8 | 0.336 | 0.0009429 |
| sp|Q8IYT2|CMTR2_HUMAN | 89925.7304 | 0.68 | 0.188 | 3.66E-07 |
| sp|Q96PQ0|SORC2_HUMAN | 129079.4578 | 2.4 | 0.862 | 2.27E-11 |
| sp|Q11201|SIA4A_HUMAN | 39374.18454 | 0.77 | 0.162 | 3.00E-07 |
| sp|Q96MG8|PCMD1_HUMAN | 40916.62795 | 0.82 | 0.082 | 3.57E-10 |
| sp|Q8TE02|ELP5_HUMAN | 35200.54812 | 1.12 | 0.276 | 0.1226 |
| sp|P17081|RHOQ_HUMAN | 23196.71857 | 0.72 | 0.18 | 5.69E-07 |
| sp|P04080|CYTB_HUMAN | 11171.60184 | 1.32 | 0.187 | 2.24E-09 |
| sp|Q9Y4K0|LOXL2_HUMAN | 88760.11705 | 1.12 | 0.316 | 0.1815 |
| sp|Q14012|KCC1A_HUMAN | 41862.91117 | 0.95 | 0.239 | 0.1347 |
| sp|Q9UIA0|CYH4_HUMAN | 46252.23519 | 0.85 | 0.293 | 0.003909 |
| sp|P32929|CGL_HUMAN | 45031.69121 | 0.75 | 0.236 | 2.70E-05 |
| sp|Q6NYC8|PPR18_HUMAN | 68168.60203 | 1.43 | 0.351 | 2.35E-07 |
| sp|Q7L4I2|RSRC2_HUMAN | 50568.31324 | 0.97 | 0.246 | 0.2367 |
| sp|Q9NWF9|RN216_HUMAN | 101148.7645 | 1.12 | 0.115 | 1.53E-05 |
| sp|P46952|3HAO_HUMAN | 32688.59415 | 1.16 | 0.257 | 0.01218 |
| sp|Q92900|RENT1_HUMAN | 125560.1967 | 0.94 | 0.087 | 0.001817 |
| sp|Q86T13|CLC14_HUMAN | 52838.97056 | 0.83 | 0.489 | 0.003421 |
| sp|Q9BWN1|PRR14_HUMAN | 64669.73648 | 1.24 | 0.282 | 0.0005635 |
| sp|Q86Y07|VRK2_HUMAN | 58541.9481 | 1.17 | 0.325 | 0.02064 |
| sp|Q09028|RBBP4_HUMAN | 47893.16921 | 1.23 | 0.226 | 1.95E-05 |
| sp|Q96DC8|ECHD3_HUMAN | 32937.0688 | 2.09 | 0.694 | 2.31E-09 |
| sp|Q9Y2V2|CHSP1_HUMAN | 16092.104 | 1.37 | 0.265 | 6.16E-08 |
| sp|O75864|PPR37_HUMAN | 75387.10131 | 0.74 | 0.123 | 7.14E-10 |
| sp|Q14155|ARHG7_HUMAN | 90849.66522 | 1.11 | 0.175 | 0.009846 |
| sp|Q9UEE5|ST17A_HUMAN | 46909.77549 | 1.2 | 0.173 | 3.47E-06 |
| sp|O95861|BPNT1_HUMAN | 33695.31482 | 0.77 | 0.117 | 2.07E-09 |
| sp|Q9H0E2|TOLIP_HUMAN | 30472.24852 | 1.24 | 0.181 | 2.20E-07 |
| sp|Q9Y5B6|PAXB1_HUMAN | 105176.887 | 0.81 | 0.218 | 0.0001029 |
| sp|Q9Y4P3|TBL2_HUMAN | 50375.4214 | 0.66 | 0.128 | 4.09E-11 |
| sp|Q9UKD1|GMEB2_HUMAN | 56823.9697 | 1.15 | 0.093 | 1.08E-08 |
| sp|Q8TEY7|UBP33_HUMAN | 108522.4121 | 1.05 | 0.29 | 0.9116 |
| sp|Q8TAV3|CP2W1_HUMAN | 54304.84755 | 0.94 | 0.249 | 0.08302 |
| sp|Q8IY63|AMOL1_HUMAN | 106832.4365 | 1.12 | 0.247 | 0.03061 |
| sp|P62857|RS28_HUMAN | 7875.212297 | 1.94 | 0.784 | 9.05E-08 |
| sp|Q96FJ2|DYL2_HUMAN | 10439.09784 | 0.89 | 0.217 | 0.006083 |
| sp|Q9Y2T2|AP3M1_HUMAN | 47233.46544 | 0.57 | 0.098 | 1.43E-14 |
| sp|O00142|KITM_HUMAN | 31308.9561 | 0.9 | 0.142 | 0.0009568 |
| sp|O95602|RPA1_HUMAN | 196664.8004 | 0.84 | 0.115 | 6.17E-07 |
| sp|Q9H0P0|5NT3A_HUMAN | 38247.91032 | 1.05 | 0.236 | 0.5636 |
| sp|Q9NNX1|TUFT1_HUMAN | 44503.64702 | 1.02 | 0.146 | 0.6248 |
| sp|Q6ZNJ1|NBEL2_HUMAN | 305386.7873 | 0.7 | 0.138 | 1.08E-09 |
| sp|Q99805|TM9S2_HUMAN | 76790.86618 | 0.7 | 0.117 | 1.30E-10 |
| sp|Q52LJ0|FA98B_HUMAN | 37548.16555 | 1.09 | 0.134 | 0.006851 |
| sp|Q9GZR1|SENP6_HUMAN | 127532.1119 | 1.42 | 0.521 | 0.002171 |
| sp|A5PLN9|TPC13_HUMAN | 47159.71738 | 1.12 | 0.119 | 5.40E-05 |
| sp|Q96HS1|PGAM5_HUMAN | 32194.68271 | 0.97 | 0.19 | 0.2104 |
| sp|Q8N2F6|ARM10_HUMAN | 37840.72102 | 0.8 | 0.219 | 6.80E-05 |
| sp|P16219|ACADS_HUMAN | 44592.87943 | 0.95 | 0.273 | 0.1456 |
| sp|Q9Y6D5|BIG2_HUMAN | 204400.073 | 0.47 | 0.136 | 9.82E-14 |
| sp|P09110|THIK_HUMAN | 44816.05931 | 0.99 | 0.145 | 0.4279 |
| sp|Q6UWJ1|TMCO3_HUMAN | 76044.55793 | 1.21 | 0.307 | 0.004157 |
| sp|Q13530|SERC3_HUMAN | 53382.97946 | 0.85 | 0.241 | 0.00124 |
| sp|P28331|NDUS1_HUMAN | 80424.91712 | 1.07 | 0.192 | 0.1869 |
| sp|P36873|PP1G_HUMAN | 37682.99234 | 0.86 | 0.138 | 1.45E-05 |
| sp|Q9H074|PAIP1_HUMAN | 53929.26713 | 0.57 | 0.099 | 3.70E-15 |
| sp|O75503|CLN5_HUMAN | 41850.76775 | 0.66 | 0.129 | 5.77E-11 |
| sp|Q02388|CO7A1_HUMAN | 295992.2973 | 0.85 | 0.283 | 0.008841 |
| sp|Q9H2L5|RASF4_HUMAN | 37105.95829 | 1.45 | 0.736 | 0.01667 |
| sp|Q8IUX4|ABC3F_HUMAN | 45827.88913 | 0.77 | 0.248 | 9.34E-05 |
| sp|P16152|CBR1_HUMAN | 30622.95866 | 0.53 | 0.158 | 3.98E-10 |
| sp|O95630|STABP_HUMAN | 48598.76115 | 1.14 | 0.159 | 0.0002945 |
| sp|Q96ME1|FXL18_HUMAN | 90091.14948 | 0.82 | 0.123 | 7.72E-07 |
| sp|Q9NWS8|RMND1_HUMAN | 51952.01534 | 0.71 | 0.051 | 2.20E-16 |
| sp|Q96K37|S35E1_HUMAN | 45067.8622 | 0.61 | 0.539 | 1.10E-05 |
| sp|P52757|CHIO_HUMAN | 54498.492 | 0.93 | 0.182 | 0.03442 |
| sp|Q9HCE6|ARGAL_HUMAN | 141869.1307 | 0.88 | 0.167 | 0.0007607 |
| sp|Q3KQZ1|S2535_HUMAN | 32626.65473 | 0.41 | 0.178 | 3.10E-08 |
| sp|Q96DM3|RMC1_HUMAN | 75649.87848 | 0.6 | 0.171 | 1.38E-09 |
| sp|P50995|ANX11_HUMAN | 54679.18833 | 1.15 | 0.141 | 8.23E-06 |
| sp|P13073|COX41_HUMAN | 19603.05944 | 0.73 | 0.193 | 1.90E-06 |
| sp|Q13889|TF2H3_HUMAN | 34964.83728 | 1.3 | 0.509 | 0.05363 |
| sp|Q96PE2|ARHGH_HUMAN | 223626.551 | 1.11 | 0.217 | 0.03182 |
| sp|P58546|MTPN_HUMAN | 13039.65923 | 1.32 | 0.163 | 1.80E-10 |
| sp|Q9Y4H4|GPSM3_HUMAN | 17950.89675 | 1.43 | 0.318 | 5.51E-08 |
| sp|Q6IA69|NADE_HUMAN | 80527.03976 | 0.68 | 0.073 | 2.44E-15 |
| sp|Q15172|2A5A_HUMAN | 56482.27389 | 0.66 | 0.098 | 2.27E-13 |
| sp|O75113|N4BP1_HUMAN | 101210.0457 | 1.04 | 0.12 | 0.2491 |
| sp|Q9Y2B2|PIGL_HUMAN | 28779.9105 | 1.56 | 0.199 | 1.13E-14 |
| sp|Q96E11|RRFM_HUMAN | 29354.66182 | 1.02 | 0.136 | 0.6967 |
| sp|O75369|FLNB_HUMAN | 280138.8633 | 1.02 | 0.29 | 0.7885 |
| sp|P09211|GSTP1_HUMAN | 23551.09806 | 1.29 | 0.463 | 0.006564 |
| sp|P36776|LONM_HUMAN | 106917.5848 | 0.91 | 0.139 | 0.001758 |
| sp|Q7Z5G4|GOGA7_HUMAN | 16024.07261 | 0.97 | 0.232 | 0.2311 |
| sp|O95183|VAMP5_HUMAN | 12949.75448 | 0.9 | 0.391 | 0.0322 |
| sp|O75439|MPPB_HUMAN | 55054.76384 | 0.98 | 0.223 | 0.3911 |
| sp|P51148|RAB5C_HUMAN | 23677.86094 | 0.69 | 0.07 | 2.65E-15 |
| sp|Q8TAC1|RFESD_HUMAN | 18074.74735 | 0.63 | 0.269 | 5.01E-06 |
| sp|Q8N831|TSYL6_HUMAN | 46111.97565 | 1.21 | 0.563 | 0.2339 |
| sp|Q6NZY7|BORG3_HUMAN | 15236.84858 | 1.31 | 0.19 | 5.51E-09 |
| sp|O94887|FARP2_HUMAN | 120707.0822 | 1.06 | 0.246 | 0.4615 |
| sp|O75828|CBR3_HUMAN | 31211.85925 | 1.62 | 0.293 | 2.69E-12 |
| sp|Q96AY3|FKB10_HUMAN | 64699.36852 | 1.63 | 0.453 | 4.64E-08 |
| sp|Q16666|IF16_HUMAN | 88637.59586 | 1.38 | 0.117 | 4.22E-16 |
| sp|P05451|REG1A_HUMAN | 19099.92452 | 0.51 | 0.269 | 2.96E-06 |
| sp|Q96H35|RBM18_HUMAN | 21731.36327 | 0.99 | 0.094 | 0.5305 |
| sp|Q00341|VIGLN_HUMAN | 141977.3666 | 1.34 | 0.153 | 2.44E-12 |
| sp|Q8N4X5|AF1L2_HUMAN | 92137.44415 | 1.18 | 0.19 | 7.06E-05 |
| sp|O94923|GLCE_HUMAN | 70380.78715 | 0.75 | 0.096 | 5.86E-12 |
| sp|Q14344|GNA13_HUMAN | 44345.78328 | 0.77 | 0.116 | 2.48E-09 |
| sp|Q08ET2|SIG14_HUMAN | 44551.44365 | 0.71 | 0.237 | 2.09E-06 |
| sp|Q14980|NUMA1_HUMAN | 239180.6212 | 1.46 | 0.315 | 9.56E-09 |
| sp|P29317|EPHA2_HUMAN | 109661.3617 | 0.88 | 0.239 | 0.005222 |
| sp|Q96JQ2|CLMN_HUMAN | 112020.2437 | 0.7 | 0.14 | 4.31E-10 |
| sp|O94851|MICA2_HUMAN | 128073.6553 | 0.7 | 0.137 | 2.46E-09 |
| sp|P61278|SMS_HUMAN | 12994.45337 | 0.64 | 0.292 | 0.0002875 |
| sp|Q08117|AES_HUMAN | 22109.04964 | 1.19 | 0.281 | 0.00389 |
| sp|Q15003|CND2_HUMAN | 83291.59329 | 1.34 | 0.352 | 9.63E-05 |
| sp|P49913|CAMP_HUMAN | 19499.23393 | 0.71 | 0.579 | 0.0005639 |
| sp|P15927|RFA2_HUMAN | 29324.48316 | 1.6 | 0.244 | 1.79E-13 |
| sp|Q70IA6|MOB2_HUMAN | 27233.44631 | 0.84 | 0.182 | 0.0002851 |
| sp|Q12972|PP1R8_HUMAN | 38607.88422 | 1.16 | 0.118 | 2.49E-07 |
| sp|Q10588|BST1_HUMAN | 36310.29712 | 0.85 | 0.288 | 0.005541 |
| sp|Q9Y3Z3|SAMH1_HUMAN | 72878.04337 | 1.37 | 0.261 | 1.41E-08 |
| sp|P45954|ACDSB_HUMAN | 47779.41355 | 0.97 | 0.254 | 0.242 |
| sp|Q6P587|FAHD1_HUMAN | 25093.83145 | 0.84 | 0.176 | 4.22E-05 |
| sp|Q15029|U5S1_HUMAN | 110317.6354 | 0.77 | 0.113 | 2.76E-09 |
| sp|P51970|NDUA8_HUMAN | 20530.22531 | 1.47 | 0.506 | 0.0002961 |
| sp|Q7L7X3|TAOK1_HUMAN | 116435.5952 | 1.17 | 0.094 | 1.15E-09 |
| sp|Q9Y3A4|RRP7A_HUMAN | 32467.06992 | 1.1 | 0.126 | 0.0009364 |
| sp|Q6UWE0|LRSM1_HUMAN | 84549.33121 | 1.19 | 0.124 | 2.77E-08 |
| sp|Q9UNI6|DUS12_HUMAN | 38272.14927 | 0.7 | 0.215 | 1.19E-06 |
| sp|P62312|LSM6_HUMAN | 9160.778021 | 0.9 | 0.15 | 0.001478 |
| sp|P83111|LACTB_HUMAN | 61093.15169 | 1.07 | 0.207 | 0.172 |
| sp|Q9H6X2|ANTR1_HUMAN | 63415.28844 | 1.13 | 0.246 | 0.02927 |
| sp|P63162|RSMN_HUMAN | 24751.09091 | 1.12 | 0.131 | 6.84E-05 |
| sp|Q9C035|TRIM5_HUMAN | 57481.49841 | 0.91 | 0.132 | 0.001459 |
| sp|Q9NX57|RAB20_HUMAN | 26584.29852 | 0.66 | 0.186 | 7.59E-08 |
| sp|Q9Y2J2|E41L3_HUMAN | 121440.4495 | 1.42 | 0.156 | 4.54E-14 |
| sp|P01833|PIGR_HUMAN | 84411.09277 | 1.09 | 0.709 | 0.4084 |
| sp|P12004|PCNA_HUMAN | 29074.41137 | 1 | 0.51 | 0.2696 |
| sp|Q15910|EZH2_HUMAN | 87229.29314 | 1.05 | 0.173 | 0.3288 |
| sp|Q6P1N9|TATD1_HUMAN | 34132.1374 | 0.84 | 0.126 | 4.74E-06 |
| sp|Q9Y4F3|MARF1_HUMAN | 195798.2304 | 0.78 | 0.124 | 2.66E-08 |
| sp|Q9NRB3|CHSTC_HUMAN | 48651.03794 | 1.72 | 0.894 | 0.0001745 |
| sp|P36578|RL4_HUMAN | 47934.5245 | 1.1 | 0.13 | 0.0005542 |
| sp|Q9UK55|ZPI_HUMAN | 50770.25555 | 0.76 | 0.232 | 2.28E-05 |
| sp|P03951|FA11_HUMAN | 72098.32051 | 1.12 | 0.407 | 0.6698 |
| sp|O75934|SPF27_HUMAN | 26211.03055 | 1.29 | 0.148 | 1.18E-10 |
| sp|Q2T9J0|TYSD1_HUMAN | 60279.69392 | 1.62 | 0.464 | 2.91E-08 |
| sp|P69891|HBG1_HUMAN | 16169.32234 | 0.74 | 0.205 | 1.45E-05 |
| sp|O75718|CRTAP_HUMAN | 47141.16308 | 1.51 | 0.409 | 5.53E-07 |
| sp|Q5T440|CAF17_HUMAN | 38455.19308 | 0.78 | 0.117 | 7.12E-09 |
| sp|O15145|ARPC3_HUMAN | 20743.44984 | 0.97 | 0.138 | 0.1442 |
| sp|Q12996|CSTF3_HUMAN | 83306.88929 | 0.6 | 0.097 | 4.63E-14 |
| sp|Q8TEQ6|GEMI5_HUMAN | 170802.7198 | 0.72 | 0.116 | 4.15E-10 |
| sp|Q9UK61|TASOR_HUMAN | 190435.1565 | 1.1 | 0.096 | 1.46E-05 |
| sp|Q14257|RCN2_HUMAN | 36892.65357 | 1.79 | 0.271 | 3.57E-16 |
| sp|Q8NEZ5|FBX22_HUMAN | 45260.06549 | 0.55 | 0.109 | 1.36E-14 |
| sp|P07437|TBB5_HUMAN | 50077.13253 | 1.23 | 0.135 | 4.75E-09 |
| sp|O60216|RAD21_HUMAN | 71911.75256 | 1.03 | 0.153 | 0.5267 |
| sp|Q5VT25|MRCKA_HUMAN | 199331.2636 | 0.88 | 0.208 | 0.002608 |
| sp|Q6AZZ1|TRI68_HUMAN | 57174.55625 | 0.84 | 0.2 | 0.0008557 |
| sp|O14641|DVL2_HUMAN | 79165.67902 | 1.02 | 0.133 | 0.5517 |
| sp|Q15334|L2GL1_HUMAN | 116639.0016 | 1.16 | 0.148 | 1.11E-05 |
| sp|O94927|HAUS5_HUMAN | 72247.22653 | 0.77 | 0.254 | 9.46E-05 |
| sp|Q13136|LIPA1_HUMAN | 136247.2059 | 1.39 | 0.281 | 7.90E-08 |
| sp|Q9HC36|MRM3_HUMAN | 47257.52062 | 1.27 | 0.292 | 0.0001201 |
| sp|Q9BT25|HAUS8_HUMAN | 44925.99363 | 1.06 | 0.12 | 0.04553 |
| sp|Q05086|UBE3A_HUMAN | 101574.7268 | 0.91 | 0.196 | 0.009774 |
| sp|A2RTX5|SYTC2_HUMAN | 93594.82954 | 0.68 | 0.337 | 1.11E-05 |
| sp|P12830|CADH1_HUMAN | 97834.02051 | 0.73 | 0.277 | 2.06E-05 |
| sp|Q7Z5K2|WAPL_HUMAN | 134498.3269 | 0.75 | 0.095 | 2.91E-11 |
| sp|Q9H910|JUPI2_HUMAN | 20089.99411 | 1.17 | 0.72 | 0.7018 |
| sp|P55263|ADK_HUMAN | 40900.80055 | 1.05 | 0.193 | 0.3863 |
| sp|Q9BW27|NUP85_HUMAN | 75808.3823 | 0.65 | 0.095 | 3.86E-14 |
| sp|P23396|RS3_HUMAN | 26824.48413 | 0.59 | 0.09 | 5.85E-15 |
| sp|O14980|XPO1_HUMAN | 124428.5148 | 0.56 | 0.063 | 2.20E-16 |
| sp|Q96D53|COQ8B_HUMAN | 60754.71332 | 0.66 | 0.185 | 3.34E-08 |
| sp|P49790|NU153_HUMAN | 155421.7702 | 1.34 | 0.153 | 6.28E-12 |
| sp|Q8WU67|ABHD3_HUMAN | 46474.16318 | 0.47 | 0.19 | 6.55E-10 |
| sp|O00487|PSDE_HUMAN | 34707.6097 | 1.31 | 0.126 | 8.30E-13 |
| sp|P0C0L4|CO4A_HUMAN | 194243.0614 | 1.01 | 0.505 | 0.2609 |
| sp|P21580|TNAP3_HUMAN | 91818.59965 | 0.94 | 0.195 | 0.06157 |
| sp|Q9H2P0|ADNP_HUMAN | 124835.5163 | 1.05 | 0.141 | 0.1599 |
| sp|Q6ZT12|UBR3_HUMAN | 215870.1907 | 1.36 | 0.353 | 4.25E-06 |
| sp|Q13247|SRSF6_HUMAN | 39659.42566 | 1.43 | 0.458 | 0.0001064 |
| sp|Q86Y56|DAAF5_HUMAN | 94755.54723 | 0.84 | 0.118 | 1.04E-06 |
| sp|P40227|TCPZ_HUMAN | 58425.75845 | 1.3 | 0.163 | 1.52E-10 |
| sp|Q15836|VAMP3_HUMAN | 11340.96214 | 0.78 | 0.174 | 1.98E-06 |
| sp|Q8WXX5|DNJC9_HUMAN | 30044.14738 | 1.42 | 0.335 | 4.34E-07 |
| sp|O75629|CREG1_HUMAN | 24155.34734 | 1.17 | 0.319 | 0.03905 |
| sp|P00414|COX3_HUMAN | 29970.04537 | 0.7 | 0.258 | 5.10E-06 |
| sp|Q9BRX8|PXL2A_HUMAN | 25843.46611 | 0.99 | 0.239 | 0.3831 |
| sp|Q04446|GLGB_HUMAN | 80860.79523 | 0.76 | 0.137 | 2.04E-08 |
| sp|P55008|AIF1_HUMAN | 16674.59802 | 2.05 | 0.571 | 4.75E-12 |
| sp|P46531|NOTC1_HUMAN | 286332.3735 | 1.28 | 0.209 | 1.92E-07 |
| sp|Q9BWH6|RPAP1_HUMAN | 154180.1341 | 0.77 | 0.152 | 2.85E-07 |
| sp|P31943|HNRH1_HUMAN | 49465.50575 | 1.19 | 0.139 | 5.14E-07 |
| sp|Q86V97|KBTB6_HUMAN | 77382.24192 | 0.96 | 0.153 | 0.1273 |
| sp|P41743|KPCI_HUMAN | 69169.70087 | 0.71 | 0.133 | 3.31E-09 |
| sp|Q8TBF4|ZCRB1_HUMAN | 24786.54298 | 1.17 | 0.177 | 5.05E-05 |
| sp|Q9H5X1|CIA2A_HUMAN | 18610.56036 | 1.14 | 0.269 | 0.02735 |
| sp|P15153|RAC2_HUMAN | 21796.21686 | 0.83 | 0.266 | 0.001676 |
| sp|P48634|PRC2A_HUMAN | 229161.7515 | 1.5 | 0.257 | 6.69E-11 |
| sp|Q13042|CDC16_HUMAN | 72389.76419 | 0.74 | 0.144 | 4.93E-08 |
| sp|Q9UJC3|HOOK1_HUMAN | 85204.05961 | 0.94 | 0.357 | 0.07576 |
| sp|Q02127|PYRD_HUMAN | 42822.9316 | 0.88 | 0.154 | 0.0007824 |
| sp|Q09666|AHNK_HUMAN | 629194.5684 | 1.65 | 0.346 | 2.77E-11 |
| sp|Q8ND56|LS14A_HUMAN | 50709.19226 | 1.3 | 0.178 | 1.09E-09 |
| sp|Q8N9V3|WSDU1_HUMAN | 54076.40321 | 0.65 | 0.151 | 9.47E-10 |
| sp|P61326|MGN_HUMAN | 17191.79727 | 0.65 | 0.159 | 1.45E-09 |
| sp|Q01831|XPC_HUMAN | 106724.5318 | 1.2 | 0.202 | 1.88E-05 |
| sp|Q6PCE3|PGM2L_HUMAN | 71347.83293 | 0.51 | 0.059 | 2.20E-16 |
| sp|O95274|LYPD3_HUMAN | 37013.01888 | 1.16 | 0.201 | 0.0009575 |
| sp|P32780|TF2H1_HUMAN | 62316.74129 | 1.35 | 0.282 | 8.45E-07 |
| sp|Q8N573|OXR1_HUMAN | 98461.63227 | 1.26 | 0.202 | 4.66E-07 |
| sp|Q504Q3|PAN2_HUMAN | 136745.6666 | 0.78 | 0.11 | 1.75E-09 |
| sp|Q14185|DOCK1_HUMAN | 216160.3423 | 0.74 | 0.075 | 6.62E-14 |
| sp|Q92599|SEPT8_HUMAN | 56216.23747 | 1.43 | 0.283 | 5.37E-09 |
| sp|Q13011|ECH1_HUMAN | 36117.50142 | 1.06 | 0.34 | 0.9725 |
| sp|P05089|ARGI1_HUMAN | 34866.351 | 0.84 | 0.338 | 0.004689 |
| sp|Q8WWM9|CYGB_HUMAN | 21487.01092 | 0.66 | 0.123 | 7.20E-11 |
| sp|P41212|ETV6_HUMAN | 53233.71007 | 1.13 | 0.2 | 0.005851 |
| sp|Q6PIJ6|FBX38_HUMAN | 135722.162 | 0.8 | 0.175 | 1.22E-05 |
| sp|P52434|RPAB3_HUMAN | 17171.45889 | 0.89 | 0.281 | 0.01359 |
| sp|Q8IY17|PLPL6_HUMAN | 152323.1643 | 0.75 | 0.076 | 3.95E-13 |
| sp|Q14694|UBP10_HUMAN | 87688.98132 | 0.86 | 0.187 | 0.0006613 |
| sp|Q9BRV8|SIKE1_HUMAN | 23858.99511 | 1.26 | 0.164 | 5.75E-09 |
| sp|Q14562|DHX8_HUMAN | 140063.8732 | 0.86 | 0.176 | 0.0003735 |
| sp|Q9UH65|SWP70_HUMAN | 69335.62291 | 1.11 | 0.147 | 0.002301 |
| sp|O14730|RIOK3_HUMAN | 59493.47541 | 1.14 | 0.141 | 1.82E-05 |
| sp|Q13153|PAK1_HUMAN | 60875.83384 | 1.14 | 0.25 | 0.03091 |
| sp|Q8IZQ1|WDFY3_HUMAN | 399493.1117 | 0.95 | 0.179 | 0.09743 |
| sp|Q9NR16|C163B_HUMAN | 165047.1649 | 0.83 | 0.206 | 0.0003508 |
| sp|Q8WYJ6|SEPT1_HUMAN | 42382.40365 | 1.35 | 0.284 | 8.75E-07 |
| sp|Q9NZV5|SELN_HUMAN | 66211.02441 | 1.09 | 0.304 | 0.4168 |
| sp|Q92485|ASM3B_HUMAN | 51219.33061 | 0.78 | 0.133 | 1.74E-08 |
| sp|P30273|FCERG_HUMAN | 9757.315316 | 1.05 | 0.683 | 0.344 |
| sp|P78332|RBM6_HUMAN | 129174.4198 | 1.37 | 0.179 | 3.18E-11 |
| sp|P01743|HV146_HUMAN | 13077.31073 | 1.39 | 0.432 | 0.0001452 |
| sp|Q9BTE1|DCTN5_HUMAN | 20665.51947 | 1.37 | 0.106 | 2.20E-16 |
| sp|P30876|RPB2_HUMAN | 135218.2122 | 0.77 | 0.12 | 8.59E-09 |
| sp|Q2TAA2|IAH1_HUMAN | 28019.20662 | 1.55 | 0.305 | 2.06E-10 |
| sp|Q8WUD6|CHPT1_HUMAN | 45676.38115 | 0.55 | 0.107 | 7.14E-14 |
| sp|Q5K651|SAMD9_HUMAN | 185686.1399 | 1.01 | 0.207 | 0.8046 |
| sp|Q8IUF1|CBWD2_HUMAN | 44330.7519 | 1.48 | 0.292 | 2.92E-09 |
| sp|P08138|TNR16_HUMAN | 46732.87921 | 4.07 | 2.008 | 4.73E-13 |
| sp|P81605|DCD_HUMAN | 11372.85992 | 1.12 | 0.193 | 0.004345 |
| sp|Q5VSL9|STRP1_HUMAN | 96238.02294 | 0.83 | 0.091 | 2.16E-09 |
| sp|Q9Y5X2|SNX8_HUMAN | 52917.25666 | 0.96 | 0.134 | 0.08454 |
| sp|P82921|RT21_HUMAN | 10891.46333 | 0.9 | 0.419 | 0.03039 |
| sp|Q9NWT1|PK1IP_HUMAN | 44488.05474 | 1.04 | 0.202 | 0.6331 |
| sp|Q15569|TESK1_HUMAN | 68535.86344 | 1.03 | 0.211 | 0.7723 |
| sp|Q16585|SGCB_HUMAN | 35078.771 | 0.66 | 0.132 | 5.46E-11 |
| sp|P49257|LMAN1_HUMAN | 57780.07811 | 0.85 | 0.156 | 8.50E-05 |
| sp|P61225|RAP2B_HUMAN | 20701.28301 | 0.96 | 0.166 | 0.1489 |
| sp|P08238|HS90B_HUMAN | 83536.22031 | 0.94 | 0.18 | 0.05615 |
| sp|Q15050|RRS1_HUMAN | 41207.1776 | 1.56 | 0.539 | 2.23E-05 |
| sp|Q13144|EI2BE_HUMAN | 81052.57795 | 0.64 | 0.273 | 2.82E-06 |
| sp|O60763|USO1_HUMAN | 108722.3881 | 0.81 | 0.156 | 6.00E-06 |
| sp|P29992|GNA11_HUMAN | 42363.61109 | 0.57 | 0.141 | 1.15E-11 |
| sp|Q15020|SART3_HUMAN | 110702.5894 | 0.77 | 0.065 | 1.84E-14 |
| sp|P18858|DNLI1_HUMAN | 102282.6546 | 0.87 | 0.36 | 0.01401 |
| sp|O43809|CPSF5_HUMAN | 26249.75068 | 1.28 | 0.278 | 5.39E-05 |
| sp|Q9H4A9|DPEP2_HUMAN | 53769.49497 | 0.91 | 0.251 | 0.0274 |
| sp|Q16891|MIC60_HUMAN | 84007.48875 | 1.05 | 0.096 | 0.02179 |
| sp|P36959|GMPR1_HUMAN | 37832.92579 | 1.15 | 0.1 | 2.27E-08 |
| sp|O75530|EED_HUMAN | 50888.89118 | 0.75 | 0.242 | 2.07E-05 |
| sp|Q9Y2G3|AT11B_HUMAN | 135397.3764 | 0.62 | 0.102 | 1.16E-13 |
| sp|P50120|RET2_HUMAN | 15849.77564 | 1.03 | 0.952 | 0.08056 |
| sp|P61626|LYSC_HUMAN | 16964.44504 | 0.55 | 0.222 | 2.37E-07 |
| sp|A7KAX9|RHG32_HUMAN | 232077.5578 | 0.97 | 0.196 | 0.198 |
| sp|Q9UHX3|AGRE2_HUMAN | 93017.62769 | 0.86 | 0.218 | 0.002343 |
| sp|Q13393|PLD1_HUMAN | 124772.0122 | 1.05 | 0.166 | 0.2037 |
| sp|Q9NVV4|PAPD1_HUMAN | 66967.30713 | 0.63 | 0.168 | 8.08E-09 |
| sp|Q9NUM4|T106B_HUMAN | 31374.69587 | 1.01 | 0.136 | 0.9672 |
| sp|Q8IYM9|TRI22_HUMAN | 58033.01357 | 0.93 | 0.119 | 0.005585 |
| sp|Q15027|ACAP1_HUMAN | 82379.16978 | 0.89 | 0.127 | 0.0002065 |
| sp|P20701|ITAL_HUMAN | 129924.3276 | 0.81 | 0.315 | 0.00176 |
| sp|Q9H8H2|DDX31_HUMAN | 94751.83566 | 0.79 | 0.097 | 7.05E-10 |
| sp|Q15629|TRAM1_HUMAN | 43196.82125 | 0.77 | 0.228 | 2.55E-05 |
| sp|Q7Z5R6|AB1IP_HUMAN | 73574.98745 | 1.4 | 0.358 | 4.38E-06 |
| sp|Q8NFC6|BD1L1_HUMAN | 332415.0093 | 1.45 | 0.179 | 1.13E-13 |
| sp|A1A4S6|RHG10_HUMAN | 89927.56755 | 1.22 | 0.128 | 9.06E-10 |
| sp|Q9NY97|B3GN2_HUMAN | 46373.69101 | 0.93 | 0.251 | 0.04258 |
| sp|Q96KR1|ZFR_HUMAN | 118061.3378 | 1.01 | 0.237 | 0.7646 |
| sp|Q92785|REQU_HUMAN | 45249.59473 | 1.1 | 0.199 | 0.02984 |
| sp|Q8WWQ0|PHIP_HUMAN | 208309.4197 | 0.92 | 0.137 | 0.003355 |
| sp|P40337|VHL_HUMAN | 24233.96831 | 1.06 | 0.113 | 0.01526 |
| sp|Q01543|FLI1_HUMAN | 51159.64727 | 0.88 | 0.068 | 7.81E-09 |
| sp|P78559|MAP1A_HUMAN | 306762.6911 | 1.51 | 0.3 | 2.40E-10 |
| sp|Q86WH2|RASF3_HUMAN | 27755.05415 | 0.83 | 0.161 | 2.76E-05 |
| sp|O00505|IMA4_HUMAN | 58270.02518 | 0.74 | 0.169 | 5.94E-07 |
| sp|Q8NG11|TSN14_HUMAN | 31336.81941 | 0.52 | 0.118 | 3.42E-13 |
| sp|Q13084|RM28_HUMAN | 30233.91978 | 1.09 | 0.179 | 0.04148 |
| sp|Q96JB2|COG3_HUMAN | 94987.68762 | 0.68 | 0.124 | 5.31E-11 |
| sp|P53816|PA216_HUMAN | 18021.2817 | 1.43 | 0.388 | 6.13E-06 |
| sp|P19878|NCF2_HUMAN | 60218.67502 | 0.77 | 0.257 | 0.0001573 |
| sp|Q13444|ADA15_HUMAN | 95618.21218 | 0.79 | 0.127 | 5.91E-08 |
| sp|Q86YT6|MIB1_HUMAN | 112442.6396 | 1.15 | 0.075 | 2.49E-10 |
| sp|Q9BY49|PECR_HUMAN | 32790.9511 | 1.03 | 0.12 | 0.3249 |
| sp|Q9NQG6|MID51_HUMAN | 51698.84315 | 0.65 | 0.132 | 1.12E-10 |
| sp|Q92934|BAD_HUMAN | 18362.69294 | 1.27 | 0.277 | 1.39E-05 |
| sp|Q96S99|PKHF1_HUMAN | 31840.95574 | 1.82 | 0.271 | 2.59E-16 |
| sp|O94842|TOX4_HUMAN | 66534.21871 | 1.3 | 0.267 | 7.63E-06 |
| sp|Q27J81|INF2_HUMAN | 136833.2179 | 0.9 | 0.144 | 0.002527 |
| sp|O94808|GFPT2_HUMAN | 77662.04623 | 0.77 | 0.275 | 0.0002111 |
| sp|Q6UXY8|TMC5_HUMAN | 115617.8801 | 0.61 | 0.207 | 6.21E-08 |
| sp|Q9BV79|MECR_HUMAN | 40760.14884 | 0.92 | 0.15 | 0.01162 |
| sp|P55039|DRG2_HUMAN | 41101.55502 | 0.9 | 0.106 | 6.32E-05 |
| sp|Q9Y616|IRAK3_HUMAN | 68845.49664 | 1.1 | 0.175 | 0.01578 |
| sp|Q86Y13|DZIP3_HUMAN | 140037.4572 | 0.8 | 0.145 | 1.19E-06 |
| sp|P35606|COPB2_HUMAN | 103259.7957 | 0.7 | 0.1 | 2.53E-12 |
| sp|P54886|P5CS_HUMAN | 87970.81156 | 0.87 | 0.167 | 0.0005209 |
| sp|P11678|PERE_HUMAN | 81940.48774 | 0.47 | 0.154 | 4.69E-10 |
| sp|Q9H497|TOR3A_HUMAN | 46721.53627 | 0.93 | 0.135 | 0.005098 |
| sp|Q8IU68|TMC8_HUMAN | 82597.40023 | 0.81 | 0.099 | 5.61E-09 |
| sp|P62304|RUXE_HUMAN | 10835.64782 | 0.92 | 0.322 | 0.08405 |
| sp|O00748|EST2_HUMAN | 62091.34529 | 0.71 | 0.36 | 0.0003109 |
| sp|Q8NBZ7|UXS1_HUMAN | 47642.72096 | 1.05 | 0.234 | 0.6694 |
| sp|O15504|NUPL2_HUMAN | 44997.0765 | 1.3 | 0.25 | 7.98E-07 |
| sp|Q8IY18|SMC5_HUMAN | 129962.0281 | 1.18 | 0.149 | 2.47E-06 |
| sp|Q03393|PTPS_HUMAN | 16471.19788 | 0.73 | 0.246 | 4.48E-05 |
| sp|Q8TDB4|HUMMR_HUMAN | 25413.25474 | 3.59 | 1.655 | 1.96E-10 |
| sp|Q15582|BGH3_HUMAN | 75243.20469 | 0.85 | 0.286 | 0.002941 |
| sp|O00291|HIP1_HUMAN | 117213.891 | 1.01 | 0.175 | 0.7993 |
| sp|Q13951|PEBB_HUMAN | 21704.76885 | 1.28 | 0.15 | 2.74E-10 |
| sp|P09619|PGFRB_HUMAN | 124954.8453 | 1.01 | 0.197 | 0.9178 |
| sp|Q9HBI1|PARVB_HUMAN | 41727.08456 | 0.86 | 0.269 | 0.004387 |
| sp|P45381|ACY2_HUMAN | 36207.269 | 2.02 | 0.697 | 3.24E-09 |
| sp|Q9NP58|ABCB6_HUMAN | 94378.3644 | 0.64 | 0.068 | 2.20E-16 |
| sp|Q5VTL8|PR38B_HUMAN | 64639.31936 | 0.98 | 0.167 | 0.2718 |
| sp|O95340|PAPS2_HUMAN | 70008.79866 | 1.04 | 0.154 | 0.393 |
| sp|Q9H7S9|ZN703_HUMAN | 58738.13742 | 1.01 | 0.147 | 0.8561 |
| sp|Q658P3|STEA3_HUMAN | 55060.9408 | 0.79 | 0.238 | 8.78E-05 |
| sp|Q9Y263|PLAP_HUMAN | 88622.92292 | 0.79 | 0.13 | 5.20E-08 |
| sp|A0A0J9YX35|HV64D_HUMAN | 12967.38492 | 1.13 | 0.456 | 0.5884 |
| sp|Q9NRX2|RM17_HUMAN | 20076.56755 | 0.71 | 0.144 | 4.96E-09 |
| sp|A2RUS2|DEND3_HUMAN | 137722.7591 | 0.89 | 0.114 | 3.07E-05 |
| sp|P13501|CCL5_HUMAN | 10250.15385 | 1.78 | 0.462 | 6.64E-11 |
| sp|Q96S55|WRIP1_HUMAN | 72811.0704 | 0.9 | 0.094 | 1.87E-05 |
| sp|P15144|AMPN_HUMAN | 109852.0206 | 0.93 | 0.285 | 0.05339 |
| sp|Q9UKM9|RALY_HUMAN | 32482.6322 | 1.38 | 0.388 | 3.98E-05 |
| sp|Q96QF0|RAB3I_HUMAN | 53653.95868 | 0.74 | 0.109 | 9.08E-11 |
| sp|Q9NXN4|GDAP2_HUMAN | 56570.60155 | 0.64 | 0.081 | 6.11E-16 |
| sp|P03905|NU4M_HUMAN | 51699.61263 | 0.52 | 0.158 | 3.75E-10 |
| sp|Q0VD83|APOBR_HUMAN | 115243.3179 | 1.34 | 0.398 | 0.0006676 |
| sp|P41219|PERI_HUMAN | 53714.45426 | 0.83 | 0.362 | 0.00343 |
| sp|Q07352|TISB_HUMAN | 36728.98089 | 1.27 | 0.336 | 0.001175 |
| sp|O43837|IDH3B_HUMAN | 42423.71513 | 1.16 | 0.138 | 2.34E-06 |
| sp|P20336|RAB3A_HUMAN | 25178.166 | 0.31 | 0.139 | 4.40E-12 |
| sp|Q9H583|HEAT1_HUMAN | 244364.1198 | 0.57 | 0.117 | 1.13E-12 |
| sp|P21589|5NTD_HUMAN | 63879.68943 | 0.81 | 0.206 | 2.73E-05 |
| sp|Q9P0K7|RAI14_HUMAN | 110582.5031 | 1.31 | 0.186 | 3.25E-09 |
| sp|Q9UBI6|GBG12_HUMAN | 8097.202426 | 0.82 | 0.178 | 1.52E-05 |
| sp|Q9P0V9|SEP10_HUMAN | 52997.70945 | 1.09 | 0.305 | 0.3491 |
| sp|Q9GZM3|RPB1B_HUMAN | 13176.05754 | 1.32 | 0.214 | 1.52E-08 |
| sp|Q9NPJ6|MED4_HUMAN | 29765.93472 | 1.45 | 0.236 | 8.00E-11 |
| sp|Q9NZ08|ERAP1_HUMAN | 107718.4619 | 0.53 | 0.168 | 2.11E-10 |
| sp|Q96KN4|FA84A_HUMAN | 32852.35606 | 0.75 | 0.206 | 4.26E-06 |
| sp|Q9ULB4|CADH9_HUMAN | 88958.14239 | 1.38 | 0.44 | 0.0001115 |
| sp|Q9H553|ALG2_HUMAN | 47727.54607 | 0.77 | 0.11 | 3.39E-09 |
| sp|Q7L5Y9|MAEA_HUMAN | 45924.25284 | 0.86 | 0.13 | 2.18E-05 |
| sp|O60711|LPXN_HUMAN | 44596.98399 | 1.22 | 0.391 | 0.02148 |
| sp|Q9H479|FN3K_HUMAN | 35358.93986 | 0.62 | 0.182 | 2.06E-08 |
| sp|Q96FS4|SIPA1_HUMAN | 112803.278 | 1.06 | 0.179 | 0.1733 |
| sp|P14060|3BHS1_HUMAN | 42492.10918 | 1.66 | 0.874 | 0.0006481 |
| sp|P49137|MAPK2_HUMAN | 45919.59792 | 0.92 | 0.1 | 0.0003419 |
| sp|Q9BT23|LIMD2_HUMAN | 14441.99646 | 1.25 | 0.739 | 0.5882 |
| sp|P62805|H4_HUMAN | 11342.37118 | 1.25 | 0.18 | 1.27E-07 |
| sp|P57772|SELB_HUMAN | 65872.32967 | 0.82 | 0.13 | 7.01E-07 |
| sp|Q9H9A5|CNO10_HUMAN | 83379.9163 | 1.06 | 0.34 | 0.9566 |
| sp|Q9BVK2|ALG8_HUMAN | 60771.23108 | 0.61 | 0.146 | 1.38E-10 |
| sp|P53990|IST1_HUMAN | 39878.51389 | 1.03 | 0.113 | 0.2654 |
| sp|Q14392|LRC32_HUMAN | 72942.01425 | 0.82 | 0.134 | 7.66E-07 |
| sp|O15160|RPAC1_HUMAN | 39435.10384 | 1.23 | 0.154 | 2.38E-08 |
| sp|Q9BX66|SRBS1_HUMAN | 142863.8866 | 1.04 | 0.312 | 0.9693 |
| sp|Q9H3N1|TMX1_HUMAN | 32151.94151 | 0.77 | 0.113 | 3.72E-09 |
| sp|Q96BJ8|ELMO3_HUMAN | 82195.36798 | 1.02 | 0.318 | 0.6631 |
| sp|Q9Y375|CIA30_HUMAN | 37779.05484 | 1.09 | 0.176 | 0.03984 |
| sp|P62280|RS11_HUMAN | 18572.04693 | 1.36 | 0.269 | 1.98E-07 |
| sp|Q9UJX5|APC4_HUMAN | 92837.99789 | 0.75 | 0.276 | 2.82E-05 |
| sp|Q9UN81|LORF1_HUMAN | 40240.77638 | 0.86 | 0.423 | 0.01292 |
| sp|P08263|GSTA1_HUMAN | 25653.67271 | 0.62 | 0.36 | 0.0001066 |
| sp|Q9NR33|DPOE4_HUMAN | 12297.32305 | 1.43 | 0.633 | 0.01316 |
| sp|Q9NP72|RAB18_HUMAN | 23229.73455 | 0.81 | 0.068 | 3.28E-12 |
| sp|Q96LB3|IFT74_HUMAN | 69292.12747 | 1.43 | 0.351 | 1.37E-06 |
| sp|P25789|PSA4_HUMAN | 29732.27684 | 0.81 | 0.181 | 4.34E-05 |
| sp|Q9HCU8|DPOD4_HUMAN | 12578.34191 | 1.53 | 0.304 | 1.98E-10 |
| sp|A4D1S5|RAB19_HUMAN | 24651.39111 | 0.84 | 0.271 | 0.0007654 |
| sp|P35270|SPRE_HUMAN | 28297.90102 | 0.71 | 0.153 | 8.03E-09 |
| sp|Q14642|I5P1_HUMAN | 48340.92073 | 0.63 | 0.123 | 3.16E-12 |
| sp|Q9UBP0|SPAST_HUMAN | 67479.33356 | 1.03 | 0.126 | 0.4413 |
| sp|A0A0G2JS06|LV539_HUMAN | 13538.72825 | 1.65 | 0.481 | 3.85E-08 |
| sp|Q15113|PCOC1_HUMAN | 48779.29167 | 1.28 | 0.404 | 0.004441 |
| sp|P36952|SPB5_HUMAN | 42511.55708 | 1.14 | 0.512 | 0.5837 |
| sp|Q96EK7|F120B_HUMAN | 105750.7893 | 0.47 | 0.137 | 1.21E-12 |
| sp|Q6UY14|ATL4_HUMAN | 119816.8673 | 0.91 | 0.234 | 0.01977 |
| sp|O14495|PLPP3_HUMAN | 35702.26975 | 0.72 | 0.211 | 3.03E-07 |
| sp|P15104|GLNA_HUMAN | 42646.53242 | 1.05 | 0.374 | 0.9477 |
| sp|P63173|RL38_HUMAN | 8251.742923 | 1.34 | 0.237 | 1.05E-07 |
| sp|Q9NQ92|COPRS_HUMAN | 20206.99981 | 1.73 | 0.696 | 5.60E-05 |
| sp|P02042|HBD_HUMAN | 16141.32224 | 1.31 | 0.516 | 0.01553 |
| sp|Q9H0U9|TSYL1_HUMAN | 49371.91844 | 0.96 | 0.12 | 0.05002 |
| sp|P54760|EPHB4_HUMAN | 109722.9379 | 0.53 | 0.088 | 6.17E-16 |
| sp|Q6ZMP0|THSD4_HUMAN | 115780.4905 | 1.09 | 0.352 | 0.544 |
| sp|Q9H204|MED28_HUMAN | 19661.08214 | 1.23 | 0.221 | 8.45E-06 |
| sp|Q13595|TRA2A_HUMAN | 32708.07462 | 1.36 | 0.276 | 1.14E-07 |
| sp|Q6KC79|NIPBL_HUMAN | 317888.6695 | 0.93 | 0.112 | 0.003128 |
| sp|P50479|PDLI4_HUMAN | 35870.84392 | 1.05 | 0.255 | 0.6994 |
| sp|Q8ND76|CCNY_HUMAN | 39807.12279 | 0.83 | 0.12 | 3.11E-07 |
| sp|Q86SZ2|TPC6B_HUMAN | 18181.29412 | 0.52 | 0.057 | 2.20E-16 |
| sp|O00217|NDUS8_HUMAN | 24184.80154 | 1.37 | 0.294 | 3.54E-07 |
| sp|Q9Y3B8|ORN_HUMAN | 27025.67427 | 1.48 | 0.266 | 2.90E-10 |
| sp|O75912|DGKI_HUMAN | 118672.5038 | 1.08 | 0.088 | 0.0001454 |
| sp|P05771|KPCB_HUMAN | 77941.52175 | 0.91 | 0.211 | 0.01552 |
| sp|O95236|APOL3_HUMAN | 44631.86332 | 2.03 | 0.532 | 1.46E-12 |
| sp|Q8N7H5|PAF1_HUMAN | 60092.16018 | 1.38 | 0.203 | 1.50E-10 |
| sp|Q6P4I2|WDR73_HUMAN | 42211.11681 | 1.06 | 0.148 | 0.06837 |
| sp|Q96AQ6|PBIP1_HUMAN | 81032.23432 | 1.09 | 0.103 | 0.0002063 |
| sp|Q4G0F5|VP26B_HUMAN | 39226.19761 | 0.95 | 0.131 | 0.02673 |
| sp|Q5VV41|ARHGG_HUMAN | 80322.31821 | 0.85 | 0.284 | 0.003422 |
| sp|O43293|DAPK3_HUMAN | 52656.43255 | 0.9 | 0.156 | 0.001813 |
| sp|Q7L775|EPMIP_HUMAN | 70934.29791 | 0.85 | 0.097 | 2.04E-07 |
| sp|Q8IVT2|MISP_HUMAN | 75464.00467 | 1.28 | 0.325 | 0.000489 |
| sp|Q96GD0|PLPP_HUMAN | 32059.43462 | 1.05 | 0.075 | 0.004074 |
| sp|Q16555|DPYL2_HUMAN | 62692.72934 | 1.26 | 0.145 | 5.37E-10 |
| sp|Q9UNX4|WDR3_HUMAN | 107097.1732 | 0.62 | 0.111 | 1.69E-12 |
| sp|O75356|ENTP5_HUMAN | 47868.1136 | 0.67 | 0.308 | 0.0001567 |
| sp|Q9HAD4|WDR41_HUMAN | 52361.64193 | 0.71 | 0.213 | 4.85E-06 |
| sp|Q99674|CGRE1_HUMAN | 31868.07398 | 1.06 | 0.147 | 0.08539 |
| sp|Q92643|GPI8_HUMAN | 45489.77829 | 0.69 | 0.128 | 5.70E-10 |
| sp|Q92878|RAD50_HUMAN | 154804.9526 | 0.96 | 0.131 | 0.08875 |
| sp|Q9Y6Q5|AP1M2_HUMAN | 48174.19977 | 0.9 | 0.202 | 0.006027 |
| sp|Q9NY61|AATF_HUMAN | 63190.37006 | 1.34 | 0.362 | 0.0001246 |
| sp|P48050|KCNJ4_HUMAN | 50077.03806 | 1.11 | 0.36 | 0.4739 |
| sp|Q9NPY3|C1QR1_HUMAN | 70949.31832 | 1.16 | 0.324 | 0.03016 |
| sp|O75594|PGRP1_HUMAN | 22097.83182 | 0.87 | 0.265 | 0.003737 |
| sp|Q9C037|TRIM4_HUMAN | 58261.53578 | 0.93 | 0.162 | 0.0206 |
| sp|P19338|NUCL_HUMAN | 76607.36959 | 1.44 | 0.26 | 2.56E-09 |
| sp|Q9H939|PPIP2_HUMAN | 39271.80716 | 1.2 | 0.513 | 0.1301 |
| sp|Q6XZF7|DNMBP_HUMAN | 178700.5733 | 0.94 | 0.122 | 0.01719 |
| sp|P10412|H14_HUMAN | 21833.99376 | 2.03 | 0.747 | 2.89E-08 |
| sp|Q99808|S29A1_HUMAN | 50738.56352 | 0.65 | 0.194 | 9.22E-08 |
| sp|P60520|GBRL2_HUMAN | 13697.07876 | 1.5 | 0.37 | 3.03E-08 |
| sp|Q8N0W3|FUK_HUMAN | 119126.8365 | 0.71 | 0.063 | 2.74E-16 |
| sp|O95081|AGFG2_HUMAN | 49313.44317 | 1.06 | 0.264 | 0.5089 |
| sp|Q5VV42|CDKAL_HUMAN | 65736.37556 | 0.83 | 0.159 | 2.29E-05 |
| sp|O60551|NMT2_HUMAN | 57268.26825 | 1.04 | 0.224 | 0.7171 |
| sp|P04066|FUCO_HUMAN | 53921.82175 | 0.51 | 0.146 | 5.09E-11 |
| sp|P22059|OSBP1_HUMAN | 90201.86276 | 0.84 | 0.19 | 0.0002266 |
| sp|Q12999|TSN31_HUMAN | 23647.06601 | 0.41 | 0.178 | 3.64E-10 |
| sp|Q13797|ITA9_HUMAN | 115708.8686 | 0.72 | 0.219 | 1.31E-06 |
| sp|P10599|THIO_HUMAN | 11996.83837 | 1.03 | 0.113 | 0.3889 |
| sp|Q9H223|EHD4_HUMAN | 61346.59825 | 0.93 | 0.151 | 0.01421 |
| sp|Q969G3|SMCE1_HUMAN | 46659.59835 | 1.33 | 0.136 | 4.91E-13 |
| sp|P20020|AT2B1_HUMAN | 135551.2432 | 0.55 | 0.105 | 3.14E-14 |
| sp|Q8NCS4|TM35B_HUMAN | 16969.44227 | 0.23 | 0.058 | 2.20E-16 |
| sp|Q9P0V3|SH3B4_HUMAN | 108379.408 | 0.55 | 0.191 | 5.06E-10 |
| sp|P17096|HMGA1_HUMAN | 11651.13157 | 1.86 | 0.922 | 4.44E-05 |
| sp|P10600|TGFB3_HUMAN | 48078.25438 | 1.48 | 0.741 | 0.006337 |
| sp|Q96J02|ITCH_HUMAN | 103575.4087 | 0.81 | 0.094 | 2.25E-09 |
| sp|Q9HCJ3|RAVR2_HUMAN | 74957.8383 | 0.85 | 0.227 | 0.001018 |
| sp|Q9Y2L5|TPPC8_HUMAN | 162531.4656 | 1.04 | 0.104 | 0.07151 |
| sp|P36871|PGM1_HUMAN | 61677.62552 | 0.55 | 0.091 | 6.31E-16 |
| sp|Q5VST9|OBSCN_HUMAN | 879611.655 | 2.92 | 2.344 | 6.54E-06 |
| sp|Q9BYG3|MK67I_HUMAN | 34354.088 | 1.37 | 0.29 | 3.84E-07 |
| sp|Q9UPY8|MARE3_HUMAN | 32229.06026 | 1.45 | 0.507 | 0.0001033 |
| sp|P61289|PSME3_HUMAN | 29583.59145 | 0.91 | 0.174 | 0.00658 |
| sp|P62068|UBP46_HUMAN | 43138.39264 | 0.39 | 0.076 | 2.20E-16 |
| sp|P20618|PSB1_HUMAN | 26682.44511 | 1.04 | 0.079 | 0.02273 |
| sp|Q9Y237|PIN4_HUMAN | 13839.98566 | 1.03 | 0.096 | 0.2002 |
| sp|Q92793|CBP_HUMAN | 268014.7611 | 1.13 | 0.157 | 0.0007003 |
| sp|Q9NQE9|HINT3_HUMAN | 20786.25984 | 0.83 | 0.191 | 0.0001729 |
| sp|P37275|ZEB1_HUMAN | 125803.6 | 0.96 | 0.389 | 0.1491 |
| sp|Q9NRP2|COXM2_HUMAN | 9663.795885 | 0.92 | 0.196 | 0.02793 |
| sp|Q9Y572|RIPK3_HUMAN | 57346.48489 | 0.96 | 0.124 | 0.05842 |
| sp|Q15005|SPCS2_HUMAN | 25253.83923 | 0.69 | 0.072 | 1.71E-15 |
| sp|Q9BSL1|UBAC1_HUMAN | 45520.08813 | 1.27 | 0.154 | 1.63E-09 |
| sp|Q9ULG3|K1257_HUMAN | 46977.91697 | 1.35 | 0.31 | 3.34E-06 |
| sp|Q96HF1|SFRP2_HUMAN | 34476.1688 | 1.93 | 0.66 | 3.07E-08 |
| sp|Q9UGM6|SYWM_HUMAN | 40274.41853 | 0.91 | 0.363 | 0.04714 |
| sp|P17275|JUNB_HUMAN | 36010.21484 | 1.1 | 0.318 | 0.4099 |
| sp|P06127|CD5_HUMAN | 55893.30692 | 0.76 | 0.149 | 3.73E-08 |
| sp|Q9NSY1|BMP2K_HUMAN | 129928.7524 | 1.21 | 0.344 | 0.006733 |
| sp|P21246|PTN_HUMAN | 19481.87111 | 1.07 | 0.193 | 0.1513 |
| sp|Q8WWW0|RASF5_HUMAN | 47669.86566 | 1.02 | 0.237 | 0.9385 |
| sp|Q9HCE5|MET14_HUMAN | 52670.19254 | 1.2 | 0.218 | 5.69E-05 |
| sp|P61201|CSN2_HUMAN | 51831.15179 | 0.75 | 0.079 | 1.38E-12 |
| sp|Q99470|SDF2_HUMAN | 23221.49135 | 1.36 | 0.147 | 2.67E-13 |
| sp|Q86Y38|XYLT1_HUMAN | 108338.7704 | 0.76 | 0.185 | 1.71E-06 |
| sp|P27105|STOM_HUMAN | 31863.79452 | 0.62 | 0.133 | 3.11E-11 |
| sp|P13591|NCAM1_HUMAN | 95352.46148 | 0.68 | 0.351 | 1.83E-05 |
| sp|P40123|CAP2_HUMAN | 53057.9186 | 1.14 | 0.346 | 0.1751 |
| sp|Q9UGT4|SUSD2_HUMAN | 91728.27898 | 1.36 | 0.364 | 4.04E-06 |
| sp|Q9Y243|AKT3_HUMAN | 56176.96805 | 1.4 | 0.207 | 4.32E-11 |
| sp|Q6NUQ4|TM214_HUMAN | 77938.75606 | 0.61 | 0.117 | 1.38E-12 |
| sp|Q96TA2|YMEL1_HUMAN | 86668.00881 | 0.75 | 0.134 | 1.53E-08 |
| sp|Q9BXP2|S12A9_HUMAN | 96714.86564 | 0.27 | 0.132 | 2.09E-14 |
| sp|P98171|RHG04_HUMAN | 105797.7523 | 0.91 | 0.154 | 0.004887 |
| sp|P28288|ABCD3_HUMAN | 75922.75783 | 0.5 | 0.142 | 1.12E-12 |
| sp|P62745|RHOB_HUMAN | 22547.28729 | 0.71 | 0.196 | 4.56E-07 |
| sp|O95994|AGR2_HUMAN | 20005.62631 | 0.63 | 0.324 | 6.91E-05 |
| sp|O95551|TYDP2_HUMAN | 41569.43852 | 1.25 | 0.25 | 1.53E-05 |
| sp|Q8NEL9|DDHD1_HUMAN | 101095.5766 | 0.96 | 0.197 | 0.1202 |
| sp|P21796|VDAC1_HUMAN | 30849.60251 | 0.54 | 0.189 | 7.14E-10 |
| sp|Q86SQ7|SDCG8_HUMAN | 83410.95764 | 1.69 | 0.205 | 2.20E-16 |
| sp|P62424|RL7A_HUMAN | 30130.0779 | 1.4 | 0.218 | 2.82E-10 |
| sp|Q9Y6R7|FCGBP_HUMAN | 596425.1691 | 0.54 | 0.221 | 6.48E-07 |
| sp|P16452|EPB42_HUMAN | 77798.0095 | 0.77 | 0.151 | 9.82E-08 |
| sp|Q5SRI9|MANEA_HUMAN | 53733.15106 | 1.13 | 0.241 | 0.03287 |
| sp|Q13950|RUNX2_HUMAN | 56879.68589 | 0.96 | 0.092 | 0.03367 |
| sp|P49914|MTHFS_HUMAN | 23393.95932 | 1.21 | 0.104 | 5.69E-11 |
| sp|P01033|TIMP1_HUMAN | 23821.79499 | 0.9 | 0.315 | 0.03217 |
| sp|P79483|DRB3_HUMAN | 30210.25896 | 0.51 | 0.236 | 3.03E-08 |
| sp|P17936|IBP3_HUMAN | 32662.11856 | 1.2 | 0.438 | 0.2137 |
| sp|Q02241|KIF23_HUMAN | 110998.1252 | 0.94 | 0.468 | 0.09452 |
| sp|O15357|SHIP2_HUMAN | 139293.2231 | 1.03 | 0.122 | 0.2821 |
| sp|Q15072|OZF_HUMAN | 34408.64375 | 1.16 | 0.387 | 0.09534 |
| sp|A8MXV4|NUD19_HUMAN | 42530.71482 | 0.79 | 0.13 | 9.56E-08 |
| sp|Q96NA2|RILP_HUMAN | 44326.65091 | 1.05 | 0.1 | 0.02332 |
| sp|Q04864|REL_HUMAN | 69256.48852 | 1.03 | 0.165 | 0.5501 |
| sp|P46109|CRKL_HUMAN | 33851.95518 | 1.33 | 0.08 | 2.20E-16 |
| sp|Q96B36|AKTS1_HUMAN | 27576.549 | 1.26 | 0.267 | 0.0001311 |
| sp|Q3ZCQ8|TIM50_HUMAN | 39831.61535 | 0.79 | 0.15 | 4.54E-07 |
| sp|Q9UIL8|PHF11_HUMAN | 38338.17656 | 1.1 | 0.21 | 0.06336 |
| sp|Q5SXH7|PKHS1_HUMAN | 52282.69911 | 1.22 | 1.311 | 0.1934 |
| sp|P05423|RPC4_HUMAN | 44521.36102 | 0.95 | 0.131 | 0.0458 |
| sp|Q9Y2L6|FRM4B_HUMAN | 118810.8416 | 1.27 | 0.19 | 6.11E-08 |
| sp|Q6NXS1|IPP2B_HUMAN | 23131.05729 | 1.51 | 0.162 | 6.21E-16 |
| sp|Q9P1U1|ARP3B_HUMAN | 48072.34387 | 1.02 | 0.161 | 0.7865 |
| sp|Q8TDX7|NEK7_HUMAN | 34966.59235 | 0.83 | 0.064 | 5.87E-12 |
| sp|Q99829|CPNE1_HUMAN | 59630.76928 | 0.67 | 0.112 | 1.07E-11 |
| sp|Q15046|SYK_HUMAN | 68442.75453 | 0.82 | 0.179 | 1.40E-05 |
| sp|Q9P1Z0|ZBTB4_HUMAN | 105999.9664 | 1.1 | 0.271 | 0.1739 |
| sp|Q14847|LASP1_HUMAN | 30079.35955 | 1.14 | 0.177 | 0.00102 |
| sp|P27695|APEX1_HUMAN | 35913.30612 | 0.76 | 0.137 | 3.01E-08 |
| sp|Q15118|PDK1_HUMAN | 49423.08162 | 0.89 | 0.171 | 0.001924 |
| sp|Q8WUK0|PTPM1_HUMAN | 22982.20261 | 0.73 | 0.144 | 8.86E-09 |
| sp|Q8TE68|ES8L1_HUMAN | 80469.06222 | 1.13 | 0.295 | 0.05632 |
| sp|O94875|SRBS2_HUMAN | 125152.5451 | 1.53 | 0.835 | 0.001769 |
| sp|Q02543|RL18A_HUMAN | 21015.99652 | 0.98 | 0.174 | 0.323 |
| sp|Q9NRY4|RHG35_HUMAN | 171928.2762 | 0.92 | 0.07 | 8.78E-06 |
| sp|P05091|ALDH2_HUMAN | 56840.80822 | 1.32 | 0.479 | 0.0086 |
| sp|O43516|WIPF1_HUMAN | 51266.38009 | 1.46 | 0.43 | 1.38E-05 |
| sp|P07305|H10_HUMAN | 20832.18466 | 1.17 | 0.305 | 0.05876 |
| sp|P68871|HBB_HUMAN | 16084.32256 | 1.49 | 0.601 | 0.0003315 |
| sp|Q7Z401|MYCPP_HUMAN | 211772.9003 | 1.74 | 0.598 | 9.65E-08 |
| sp|P01861|IGHG4_HUMAN | 36413.04851 | 2.27 | 1.473 | 8.65E-06 |
| sp|O43719|HTSF1_HUMAN | 86352.76544 | 1.21 | 0.212 | 2.23E-05 |
| sp|Q9Y3A6|TMED5_HUMAN | 26084.22226 | 0.59 | 0.141 | 1.62E-11 |
| sp|Q8TB52|FBX30_HUMAN | 84228.58088 | 1.27 | 0.208 | 7.40E-07 |
| sp|Q9UBZ9|REV1_HUMAN | 139340.6607 | 0.98 | 0.422 | 0.2063 |
| sp|P25787|PSA2_HUMAN | 25978.30242 | 0.94 | 0.094 | 0.003557 |
| sp|Q9Y6M5|ZNT1_HUMAN | 56272.42413 | 0.7 | 0.111 | 2.00E-11 |
| sp|Q6GQQ9|OTU7B_HUMAN | 93134.28168 | 1.14 | 0.292 | 0.06139 |
| sp|Q8N3E9|PLCD3_HUMAN | 90096.53822 | 1.01 | 0.133 | 0.8841 |
| sp|P11498|PYC_HUMAN | 130274.6798 | 1.19 | 0.155 | 2.35E-06 |
| sp|Q15542|TAF5_HUMAN | 87271.46152 | 1.04 | 0.109 | 0.09299 |
| sp|Q96D46|NMD3_HUMAN | 58745.29885 | 0.74 | 0.148 | 6.38E-08 |
| sp|Q9NSC5|HOME3_HUMAN | 39794.28705 | 1.35 | 0.201 | 7.46E-10 |
| sp|P35749|MYH11_HUMAN | 228036.2186 | 0.49 | 0.214 | 1.70E-08 |
| sp|Q9Y6A4|CFA20_HUMAN | 22913.04633 | 0.69 | 0.25 | 3.69E-06 |
| sp|Q16186|ADRM1_HUMAN | 42393.90801 | 1.29 | 0.216 | 2.83E-07 |
| sp|Q9P2K6|KLH42_HUMAN | 57440.6378 | 0.54 | 0.158 | 1.76E-10 |
| sp|P29692|EF1D_HUMAN | 31198.81559 | 1.48 | 0.177 | 5.58E-14 |
| sp|Q8NBK3|SUMF1_HUMAN | 41139.76486 | 1.63 | 0.443 | 2.29E-09 |
| sp|Q8N3Y1|FBXW8_HUMAN | 67846.78961 | 1.82 | 0.451 | 1.58E-10 |
| sp|Q02094|RHAG_HUMAN | 44435.77623 | 0.36 | 0.108 | 1.43E-15 |
| sp|Q96PP8|GBP5_HUMAN | 67127.17702 | 1.88 | 1.627 | 0.004592 |
| sp|P48740|MASP1_HUMAN | 80830.80929 | 0.92 | 0.326 | 0.06176 |
| sp|O94973|AP2A2_HUMAN | 104789.3196 | 0.89 | 0.086 | 9.19E-07 |
| sp|P37802|TAGL2_HUMAN | 22530.22686 | 1.26 | 0.193 | 1.67E-07 |
| sp|Q8NAF0|ZN579_HUMAN | 61707.78851 | 1.22 | 0.277 | 0.0002721 |
| sp|O14802|RPC1_HUMAN | 157519.4243 | 1.1 | 0.238 | 0.09224 |
| sp|P41226|UBA7_HUMAN | 112575.4472 | 0.8 | 0.124 | 1.08E-07 |
| sp|Q9NUU7|DD19A_HUMAN | 54378.9555 | 0.95 | 0.114 | 0.03456 |
| sp|P49023|PAXI_HUMAN | 65928.3982 | 1.08 | 0.113 | 0.001024 |
| sp|Q6Y7W6|GGYF2_HUMAN | 150302.3883 | 1.3 | 0.225 | 2.37E-07 |
| sp|P28906|CD34_HUMAN | 41072.53372 | 1.6 | 0.67 | 4.37E-06 |
| sp|P49327|FAS_HUMAN | 275859.2645 | 0.82 | 0.144 | 2.35E-06 |
| sp|Q8N5V2|NGEF_HUMAN | 83110.92619 | 0.8 | 0.144 | 4.57E-07 |
| sp|O43813|LANC1_HUMAN | 45976.84199 | 0.61 | 0.145 | 9.47E-12 |
| sp|Q15121|PEA15_HUMAN | 15069.79821 | 1.65 | 0.242 | 5.64E-15 |
| sp|Q92615|LAR4B_HUMAN | 80883.7743 | 1.37 | 0.452 | 0.0004517 |
| sp|Q8WWC4|MAIP1_HUMAN | 32905.13254 | 0.5 | 0.111 | 2.14E-14 |
| sp|Q8IV48|ERI1_HUMAN | 40476.37024 | 1.25 | 0.27 | 0.0001065 |
| sp|Q5FWF4|ZRAB3_HUMAN | 124805.3712 | 1.09 | 0.366 | 0.5571 |
| sp|Q6IBS0|TWF2_HUMAN | 39733.34855 | 1.22 | 0.14 | 1.22E-08 |
| sp|Q16647|PTGIS_HUMAN | 57163.8475 | 0.92 | 0.153 | 0.005933 |
| sp|Q7Z2W9|RM21_HUMAN | 22953.39553 | 1.34 | 0.264 | 6.64E-07 |
| sp|O15381|NVL_HUMAN | 95999.36296 | 1.17 | 0.254 | 0.004639 |
| sp|Q9UNE7|CHIP_HUMAN | 35272.40935 | 1.37 | 0.151 | 4.77E-13 |
| sp|Q8IYB7|DI3L2_HUMAN | 100224.8516 | 0.76 | 0.128 | 1.51E-08 |
| sp|P32519|ELF1_HUMAN | 67666.17469 | 1.4 | 0.433 | 2.34E-05 |
| sp|P36269|GGT5_HUMAN | 62717.49571 | 0.88 | 0.151 | 0.0003165 |
| sp|Q16512|PKN1_HUMAN | 104647.8487 | 0.97 | 0.159 | 0.1879 |
| sp|P54802|ANAG_HUMAN | 82651.80258 | 0.69 | 0.086 | 2.43E-13 |
| sp|Q15006|EMC2_HUMAN | 34964.42521 | 0.99 | 0.092 | 0.4749 |
| sp|Q7RTS9|DYM_HUMAN | 76553.29214 | 0.75 | 0.136 | 1.99E-08 |
| sp|P14174|MIF_HUMAN | 12621.27314 | 1.4 | 0.337 | 8.09E-07 |
| sp|Q9NZC7|WWOX_HUMAN | 47085.39757 | 0.79 | 0.179 | 3.15E-06 |
| sp|Q8N8N7|PTGR2_HUMAN | 38912.49275 | 1.05 | 0.269 | 0.7713 |
| sp|Q7LBC6|KDM3B_HUMAN | 193154.0745 | 1.17 | 0.146 | 2.33E-06 |
| sp|Q7Z7H5|TMED4_HUMAN | 26079.3939 | 0.61 | 0.19 | 7.62E-09 |
| sp|Q6UVK1|CSPG4_HUMAN | 251048.6417 | 0.94 | 0.098 | 0.003775 |
| sp|Q9UGP8|SEC63_HUMAN | 88322.74299 | 0.7 | 0.077 | 1.55E-14 |
| sp|Q14966|ZN638_HUMAN | 221895.5784 | 1.21 | 0.073 | 3.12E-14 |
| sp|Q92572|AP3S1_HUMAN | 21928.20497 | 0.72 | 0.165 | 5.24E-08 |
| sp|P61978|HNRPK_HUMAN | 51211.49955 | 1.25 | 0.178 | 5.66E-08 |
| sp|Q8N436|CPXM2_HUMAN | 86425.14666 | 0.88 | 0.128 | 9.23E-05 |
| sp|P00480|OTC_HUMAN | 40062.72077 | 0.69 | 0.286 | 4.92E-05 |
| sp|Q96K17|BT3L4_HUMAN | 17242.01145 | 1.29 | 0.228 | 4.65E-07 |
| sp|Q8NI60|COQ8A_HUMAN | 72399.57734 | 0.76 | 0.188 | 4.65E-06 |
| sp|Q9BWG4|SSBP4_HUMAN | 39515.52605 | 1.01 | 0.253 | 0.6792 |
| sp|O15484|CAN5_HUMAN | 74074.01141 | 0.55 | 0.091 | 2.27E-15 |
| sp|P51610|HCFC1_HUMAN | 210579.9129 | 1.17 | 0.126 | 4.10E-07 |
| sp|Q15637|SF01_HUMAN | 68495.97784 | 1.29 | 0.233 | 3.15E-06 |
| sp|Q9NR56|MBNL1_HUMAN | 42513.25918 | 1.24 | 0.124 | 1.51E-10 |
| sp|P55285|CADH6_HUMAN | 88521.26525 | 1.5 | 0.469 | 6.54E-06 |
| sp|P11836|CD20_HUMAN | 33322.8409 | 0.95 | 0.338 | 0.1582 |
| sp|Q8NC42|RN149_HUMAN | 43689.19404 | 0.75 | 0.193 | 1.10E-06 |
| sp|P04062|GLCM_HUMAN | 60116.40666 | 0.68 | 0.13 | 1.06E-10 |
| sp|Q8NHV1|GIMA7_HUMAN | 34981.91387 | 1.11 | 0.198 | 0.02428 |
| sp|O15439|MRP4_HUMAN | 150325.995 | 0.58 | 0.131 | 5.16E-12 |
| sp|O95747|OXSR1_HUMAN | 58253.14737 | 1.04 | 0.092 | 0.05529 |
| sp|Q9UQ90|SPG7_HUMAN | 88388.96447 | 0.94 | 0.201 | 0.05711 |
| sp|Q8WY22|BRI3B_HUMAN | 27913.86908 | 0.35 | 0.12 | 3.98E-14 |
| sp|O43852|CALU_HUMAN | 37179.57522 | 1.55 | 0.211 | 8.46E-14 |
| sp|Q9BQ75|CMS1_HUMAN | 32131.07004 | 0.82 | 0.22 | 0.0004141 |
| sp|Q9Y2Q0|AT8A1_HUMAN | 132578.9655 | 0.62 | 0.154 | 8.24E-10 |
| sp|Q86WQ0|NR2CA_HUMAN | 16018.99688 | 0.98 | 0.151 | 0.3968 |
| sp|P35527|K1C9_HUMAN | 62236.89002 | 1.6 | 0.598 | 8.95E-06 |
| sp|Q14CB8|RHG19_HUMAN | 55988.26732 | 0.99 | 0.257 | 0.3999 |
| sp|P22090|RS4Y1_HUMAN | 29647.11376 | 1.34 | 1.007 | 0.8994 |
| sp|P0DJD8|PEPA3_HUMAN | 42330.74346 | 0.64 | 0.209 | 1.66E-06 |
| sp|Q14008|CKAP5_HUMAN | 227044.4071 | 0.69 | 0.113 | 1.71E-11 |
| sp|Q9BY43|CHM4A_HUMAN | 25064.80966 | 1.46 | 0.372 | 2.73E-07 |
| sp|P00403|COX2_HUMAN | 25701.26517 | 0.62 | 0.182 | 1.01E-08 |
| sp|Q86YZ3|HORN_HUMAN | 283121.9613 | 2.13 | 1.782 | 0.0003369 |
| sp|O75438|NDUB1_HUMAN | 6995.649229 | 0.75 | 0.202 | 3.86E-06 |
| sp|O43765|SGTA_HUMAN | 34251.77015 | 1.44 | 0.152 | 3.85E-15 |
| sp|Q15042|RB3GP_HUMAN | 111576.1697 | 0.86 | 0.185 | 0.0002173 |
| sp|Q96ES7|SGF29_HUMAN | 33427.317 | 1.07 | 0.157 | 0.09757 |
| sp|Q15428|SF3A2_HUMAN | 49320.37555 | 1.37 | 0.286 | 1.72E-07 |
| sp|Q9Y5L4|TIM13_HUMAN | 10703.07077 | 1.36 | 0.264 | 8.79E-08 |
| sp|Q7Z3E2|CC186_HUMAN | 104289.3195 | 0.97 | 0.114 | 0.09055 |
| sp|Q969E2|SCAM4_HUMAN | 26034.85161 | 0.63 | 0.133 | 1.14E-10 |
| sp|P51606|RENBP_HUMAN | 49465.28964 | 0.62 | 0.113 | 1.52E-12 |
| sp|O14939|PLD2_HUMAN | 106700.5252 | 0.85 | 0.18 | 0.0003013 |
| sp|Q06587|RING1_HUMAN | 42954.97075 | 0.94 | 0.146 | 0.01889 |
| sp|P49736|MCM2_HUMAN | 102498.4051 | 0.97 | 0.257 | 0.2276 |
| sp|P52594|AGFG1_HUMAN | 58491.26064 | 1.86 | 0.825 | 7.16E-06 |
| sp|Q14697|GANAB_HUMAN | 107244.8087 | 0.83 | 0.113 | 2.15E-07 |
| sp|Q9Y2R4|DDX52_HUMAN | 67780.00808 | 1.37 | 0.288 | 6.78E-07 |
| sp|Q9HD20|AT131_HUMAN | 134505.2438 | 0.79 | 0.075 | 5.97E-12 |
| sp|Q15493|RGN_HUMAN | 33783.64725 | 1.23 | 0.827 | 0.6012 |
| sp|Q9Y692|GMEB1_HUMAN | 62875.65482 | 1.18 | 0.102 | 1.10E-09 |
| sp|Q9BPX7|CG025_HUMAN | 46689.25486 | 1.04 | 0.188 | 0.553 |
| sp|O00391|QSOX1_HUMAN | 83305.79568 | 0.58 | 0.165 | 5.25E-10 |
| sp|Q92574|TSC1_HUMAN | 130636.6858 | 1.29 | 0.17 | 3.44E-09 |
| sp|Q8TB37|NUBPL_HUMAN | 34270.96657 | 0.83 | 0.096 | 2.58E-08 |
| sp|Q9UBX3|DIC_HUMAN | 31700.48761 | 0.63 | 0.169 | 1.41E-09 |
| sp|P18510|IL1RA_HUMAN | 20366.0769 | 1.04 | 0.276 | 0.8746 |
| sp|Q8WTT2|NOC3L_HUMAN | 92927.73004 | 0.61 | 0.112 | 1.27E-12 |
| sp|Q8TEY5|CR3L4_HUMAN | 43900.24545 | 1.2 | 0.353 | 0.01889 |
| sp|P68366|TBA4A_HUMAN | 50615.63785 | 0.88 | 0.131 | 0.0001002 |
| sp|Q3ZCW2|LEGL_HUMAN | 19183.72719 | 0.78 | 0.123 | 3.63E-08 |
| sp|Q9NWV4|CZIB_HUMAN | 18360.92741 | 1.42 | 0.182 | 6.88E-13 |
| sp|Q9H7Z6|KAT8_HUMAN | 52864.87602 | 0.99 | 0.151 | 0.5135 |
| sp|Q13422|IKZF1_HUMAN | 58613.82326 | 1.3 | 0.362 | 0.0006232 |
| sp|P15924|DESP_HUMAN | 334002.6168 | 0.79 | 0.332 | 0.00104 |
| sp|P28070|PSB4_HUMAN | 29224.48879 | 0.93 | 0.125 | 0.006667 |
| sp|P0C7T5|ATX1L_HUMAN | 73755.95887 | 1.83 | 0.325 | 4.65E-15 |
| sp|Q9NUS5|AP5S1_HUMAN | 22660.95358 | 0.66 | 0.084 | 1.05E-14 |
| sp|Q9NUP7|TRM13_HUMAN | 55050.08345 | 1.13 | 0.142 | 6.91E-05 |
| sp|Q9UNA1|RHG26_HUMAN | 92899.83968 | 0.63 | 0.104 | 6.25E-13 |
| sp|O14530|TXND9_HUMAN | 26670.54042 | 1.43 | 0.154 | 2.11E-14 |
| sp|A0AV96|RBM47_HUMAN | 64496.39582 | 0.89 | 0.25 | 0.007084 |
| sp|Q8IZJ1|UNC5B_HUMAN | 105549.4718 | 1.08 | 0.449 | 0.9624 |
| sp|O15379|HDAC3_HUMAN | 49482.68439 | 0.86 | 0.206 | 0.001559 |
| sp|O75347|TBCA_HUMAN | 12885.72184 | 1.37 | 0.159 | 6.45E-13 |
| sp|P0CK96|S352B_HUMAN | 44073.14618 | 0.48 | 0.152 | 6.39E-12 |
| sp|Q9Y6Q2|STON1_HUMAN | 84267.67969 | 0.88 | 0.154 | 0.0007044 |
| sp|O43451|MGA_HUMAN | 211013.0519 | 0.61 | 0.311 | 5.36E-06 |
| sp|Q93008|USP9X_HUMAN | 295496.9049 | 0.62 | 0.057 | 2.20E-16 |
| sp|Q7L266|ASGL1_HUMAN | 32358.34289 | 0.75 | 0.189 | 1.91E-06 |
| sp|P01023|A2MG_HUMAN | 164595.4176 | 0.86 | 0.227 | 0.001421 |
| sp|P26038|MOES_HUMAN | 67873.82208 | 1.12 | 0.182 | 0.004939 |
| sp|P11172|UMPS_HUMAN | 52626.75634 | 0.83 | 0.07 | 2.62E-11 |
| sp|Q9UMX1|SUFU_HUMAN | 54237.12013 | 2.24 | 0.515 | 5.28E-14 |
| sp|Q7Z5L7|PODN_HUMAN | 69143.54092 | 1.2 | 0.381 | 0.01374 |
| sp|O00175|CCL24_HUMAN | 13448.94313 | 0.74 | 0.329 | 0.0006773 |
| sp|P20340|RAB6A_HUMAN | 23673.95004 | 0.61 | 0.075 | 2.20E-16 |
| sp|P20042|IF2B_HUMAN | 38688.48777 | 1.02 | 0.193 | 0.9918 |
| sp|Q9Y2S6|TMA7_HUMAN | 7043.922018 | 1.97 | 0.876 | 1.71E-06 |
| sp|Q13613|MTMR1_HUMAN | 75353.78003 | 0.74 | 0.13 | 4.05E-09 |
| sp|P51689|ARSD_HUMAN | 65826.43958 | 0.83 | 0.155 | 1.62E-05 |
| sp|Q96BP3|PPWD1_HUMAN | 74080.10394 | 0.96 | 0.118 | 0.06478 |
| sp|Q9ULD2|MTUS1_HUMAN | 142659.3718 | 1.05 | 0.258 | 0.7413 |
| sp|Q07075|AMPE_HUMAN | 109670.7798 | 1.06 | 0.235 | 0.3922 |
| sp|O75368|SH3L1_HUMAN | 12748.36775 | 1.39 | 0.3 | 2.44E-07 |
| sp|Q9Y2A7|NCKP1_HUMAN | 130000.004 | 0.49 | 0.08 | 2.20E-16 |
| sp|P16157|ANK1_HUMAN | 207316.3648 | 1.38 | 0.258 | 6.79E-09 |
| sp|Q15007|FL2D_HUMAN | 44369.75653 | 1.37 | 0.15 | 4.53E-13 |
| sp|A1A5D9|BICL2_HUMAN | 56839.18723 | 1.03 | 0.19 | 0.7974 |
| sp|Q7Z3U7|MON2_HUMAN | 192329.0847 | 0.49 | 0.07 | 2.20E-16 |
| sp|Q96EB1|ELP4_HUMAN | 46996.40142 | 1.23 | 0.306 | 0.002204 |
| sp|P80404|GABT_HUMAN | 57068.83464 | 0.81 | 0.277 | 0.002092 |
| sp|Q86XL3|ANKL2_HUMAN | 104886.9743 | 1.11 | 0.179 | 0.005139 |
| sp|P04632|CPNS1_HUMAN | 28450.78631 | 1.2 | 0.165 | 3.24E-06 |
| sp|O95239|KIF4A_HUMAN | 141372.3064 | 0.92 | 0.312 | 0.05475 |
| sp|P78314|3BP2_HUMAN | 62642.29164 | 0.9 | 0.241 | 0.01842 |
| sp|Q9NP74|PALMD_HUMAN | 62758.73859 | 2.77 | 0.931 | 3.49E-12 |
| sp|Q6UXB4|CLC4G_HUMAN | 33150.2203 | 1.13 | 0.291 | 0.07689 |
| sp|Q14690|RRP5_HUMAN | 209920.7243 | 0.86 | 0.161 | 0.0001089 |
| sp|Q8NCE2|MTMRE_HUMAN | 72995.10283 | 0.85 | 0.14 | 2.31E-05 |
| sp|P68036|UB2L3_HUMAN | 18003.29292 | 1.35 | 0.186 | 2.53E-10 |
| sp|Q00653|NFKB2_HUMAN | 97355.17185 | 1.18 | 0.205 | 0.0001094 |
| sp|Q03252|LMNB2_HUMAN | 70001.73037 | 1.51 | 0.392 | 2.34E-08 |
| sp|O75558|STX11_HUMAN | 33612.96801 | 0.98 | 0.308 | 0.3011 |
| sp|Q9H7N4|SFR19_HUMAN | 139795.5208 | 1.38 | 0.218 | 5.23E-10 |
| sp|Q7Z406|MYH14_HUMAN | 228683.1243 | 0.69 | 0.223 | 4.09E-07 |
| sp|Q9HAV4|XPO5_HUMAN | 138313.9307 | 0.68 | 0.16 | 8.36E-09 |
| sp|P16233|LIPP_HUMAN | 51904.56714 | 0.5 | 0.215 | 1.93E-09 |
| sp|Q9P2R3|ANFY1_HUMAN | 129896.6892 | 0.64 | 0.104 | 2.38E-13 |
| sp|Q86Y82|STX12_HUMAN | 31718.314 | 1.24 | 0.224 | 6.97E-06 |
| sp|Q96QZ7|MAGI1_HUMAN | 165432.1648 | 0.92 | 0.18 | 0.01856 |
| sp|Q9BZE1|RM37_HUMAN | 48582.27775 | 0.72 | 0.099 | 1.10E-11 |
| sp|P11908|PRPS2_HUMAN | 35128.11685 | 0.81 | 0.124 | 1.31E-07 |
| sp|P46060|RAGP1_HUMAN | 63940.46538 | 1.05 | 0.126 | 0.09126 |
| sp|O60231|DHX16_HUMAN | 119855.507 | 0.74 | 0.157 | 7.54E-08 |
| sp|Q9H2C0|GAN_HUMAN | 68660.07563 | 1.12 | 0.281 | 0.0963 |
| sp|P55201|BRPF1_HUMAN | 139047.9767 | 1.17 | 0.09 | 5.12E-10 |
| sp|Q9Y6I4|UBP3_HUMAN | 60437.51836 | 1.28 | 0.271 | 1.76E-05 |
| sp|Q16134|ETFD_HUMAN | 69232.14259 | 0.83 | 0.161 | 4.11E-05 |
| sp|Q9UPN9|TRI33_HUMAN | 124604.5196 | 1.44 | 0.413 | 8.03E-06 |
| sp|Q96QG7|MTMR9_HUMAN | 64144.39311 | 0.74 | 0.089 | 3.42E-12 |
| sp|Q16082|HSPB2_HUMAN | 20259.16386 | 2.31 | 0.754 | 6.46E-11 |
| sp|Q9Y2D2|S35A3_HUMAN | 36114.33909 | 0.47 | 0.189 | 5.18E-11 |
| sp|P30049|ATPD_HUMAN | 17461.18315 | 1.39 | 0.231 | 1.12E-09 |
| sp|O15372|EIF3H_HUMAN | 40058.1085 | 1.35 | 0.177 | 1.01E-10 |
| sp|P28066|PSA5_HUMAN | 26547.25881 | 1.35 | 0.161 | 2.10E-12 |
| sp|P20648|ATP4A_HUMAN | 115737.849 | 0.84 | 0.325 | 0.01691 |
| sp|Q9NXR8|ING3_HUMAN | 47208.40265 | 1.41 | 0.158 | 8.14E-14 |
| sp|Q9BWP8|COL11_HUMAN | 29028.12806 | 1.01 | 0.487 | 0.2699 |
| sp|P46939|UTRO_HUMAN | 396426.0816 | 1.32 | 0.425 | 0.0003075 |
| sp|P37268|FDFT_HUMAN | 48579.37532 | 0.64 | 0.192 | 6.09E-07 |
| sp|Q04917|1433F_HUMAN | 28354.07254 | 1.38 | 0.227 | 3.89E-09 |
| sp|O75400|PR40A_HUMAN | 109003.6724 | 1.43 | 0.297 | 1.13E-08 |
| sp|Q6P2E9|EDC4_HUMAN | 152974.2089 | 0.68 | 0.082 | 1.46E-14 |
| sp|Q8NHV4|NEDD1_HUMAN | 72530.38436 | 1.28 | 0.144 | 1.50E-10 |
| sp|P51648|AL3A2_HUMAN | 55251.0486 | 0.61 | 0.165 | 1.34E-08 |
| sp|Q8TCJ2|STT3B_HUMAN | 94222.89841 | 0.61 | 0.093 | 2.93E-14 |
| sp|Q8TB45|DPTOR_HUMAN | 46759.47875 | 1.34 | 0.342 | 9.93E-05 |
| sp|P07949|RET_HUMAN | 126160.0069 | 1 | 0.339 | 0.3881 |
| sp|Q8IYK4|GT252_HUMAN | 73202.06012 | 1.23 | 0.232 | 9.12E-05 |
| sp|P08670|VIME_HUMAN | 53658.08957 | 2.15 | 0.574 | 3.14E-13 |
| sp|O60675|MAFK_HUMAN | 17551.42898 | 1.07 | 0.163 | 0.1086 |
| sp|P55081|MFAP1_HUMAN | 51908.61666 | 1.31 | 0.328 | 5.20E-05 |
| sp|Q16890|TPD53_HUMAN | 22474.07721 | 1.67 | 0.629 | 3.06E-05 |
| sp|Q9H8M7|MINY3_HUMAN | 50358.78616 | 1.12 | 0.283 | 0.08665 |
| sp|O15212|PFD6_HUMAN | 14555.79338 | 1.16 | 0.186 | 0.0003466 |
| sp|Q86XR8|CEP57_HUMAN | 57377.62891 | 1.21 | 0.193 | 8.74E-06 |
| sp|Q7Z2X4|PCLI1_HUMAN | 28748.2502 | 1.02 | 0.21 | 0.9436 |
| sp|Q9H0E9|BRD8_HUMAN | 136031.4056 | 1.1 | 0.121 | 0.0001917 |
| sp|P83436|COG7_HUMAN | 86955.20916 | 0.69 | 0.141 | 1.09E-09 |
| sp|P54709|AT1B3_HUMAN | 31816.21249 | 0.65 | 0.104 | 7.12E-13 |
| sp|Q9BQ24|ZFY21_HUMAN | 27211.47435 | 0.88 | 0.203 | 0.002053 |
| sp|Q9Y4W2|LAS1L_HUMAN | 83964.15176 | 0.96 | 0.136 | 0.07539 |
| sp|Q9HCU0|CD248_HUMAN | 82785.00788 | 2.18 | 0.611 | 7.57E-12 |
| sp|Q2NL82|TSR1_HUMAN | 92132.98561 | 0.75 | 0.145 | 3.66E-08 |
| sp|P50542|PEX5_HUMAN | 71144.77093 | 1.05 | 0.164 | 0.2143 |
| sp|Q96GX2|A7L3B_HUMAN | 11031.269 | 1.36 | 0.218 | 5.10E-09 |
| sp|Q01459|DIAC_HUMAN | 44397.8369 | 1.37 | 0.304 | 3.61E-07 |
| sp|Q9NVZ3|NECP2_HUMAN | 28417.33386 | 1.54 | 0.256 | 1.80E-11 |
| sp|Q9Y259|CHKB_HUMAN | 45566.62001 | 0.86 | 0.169 | 0.0002777 |
| sp|P06681|CO2_HUMAN | 84564.81783 | 0.81 | 0.168 | 6.50E-06 |
| sp|Q9BSC4|NOL10_HUMAN | 80803.61444 | 1.23 | 0.143 | 2.77E-08 |
| sp|P52756|RBM5_HUMAN | 92592.14483 | 0.97 | 0.16 | 0.1937 |
| sp|Q13442|HAP28_HUMAN | 20599.61995 | 1.55 | 0.521 | 6.67E-06 |
| sp|P06307|CCKN_HUMAN | 12757.3809 | 0.76 | 0.359 | 0.001199 |
| sp|Q6PI78|TMM65_HUMAN | 25748.54522 | 0.43 | 0.099 | 2.33E-15 |
| sp|P53618|COPB_HUMAN | 108196.1676 | 0.62 | 0.08 | 1.42E-15 |
| sp|Q9BWD1|THIC_HUMAN | 41819.57015 | 0.84 | 0.137 | 7.02E-06 |
| sp|Q6Y288|B3GLT_HUMAN | 57194.20912 | 0.99 | 0.136 | 0.5237 |
| sp|Q14192|FHL2_HUMAN | 34148.35518 | 0.68 | 0.108 | 1.39E-11 |
| sp|Q9BXY0|MAK16_HUMAN | 35670.82383 | 1.06 | 0.156 | 0.1324 |
| sp|P61313|RL15_HUMAN | 24227.11629 | 1.11 | 0.099 | 1.10E-05 |
| sp|P78324|SHPS1_HUMAN | 55428.0064 | 1.18 | 0.255 | 0.002827 |
| sp|Q8NF91|SYNE1_HUMAN | 1017109.048 | 0.85 | 0.155 | 4.56E-05 |
| sp|O95168|NDUB4_HUMAN | 15238.08073 | 0.8 | 0.213 | 0.0001181 |
| sp|O14737|PDCD5_HUMAN | 14258.33028 | 1.35 | 0.303 | 4.59E-06 |
| sp|P82930|RT34_HUMAN | 25673.49933 | 0.93 | 0.211 | 0.05289 |
| sp|Q15746|MYLK_HUMAN | 213301.7877 | 0.61 | 0.256 | 3.36E-07 |
| sp|Q16602|CALRL_HUMAN | 53846.00737 | 0.74 | 0.11 | 3.39E-10 |
| sp|Q147X3|NAA30_HUMAN | 39846.82823 | 0.79 | 0.174 | 1.88E-06 |
| sp|P13667|PDIA4_HUMAN | 73211.09169 | 1.08 | 0.253 | 0.2808 |
| sp|Q9BXW7|HDHD5_HUMAN | 46729.95324 | 0.8 | 0.157 | 5.52E-06 |
| sp|Q8N2U0|TM256_HUMAN | 11830.1734 | 0.52 | 0.121 | 1.60E-13 |
| sp|Q96S16|JMJD8_HUMAN | 29643.84934 | 1.47 | 0.347 | 5.86E-07 |
| sp|Q9P016|THYN1_HUMAN | 25834.02479 | 1.32 | 0.195 | 2.38E-09 |
| sp|P26006|ITA3_HUMAN | 117717.4536 | 0.72 | 0.199 | 6.81E-07 |
| sp|Q9BQL6|FERM1_HUMAN | 77996.64968 | 0.76 | 0.249 | 1.93E-05 |
| sp|Q9HC52|CBX8_HUMAN | 43465.47566 | 0.7 | 0.185 | 1.04E-07 |
| sp|P04114|APOB_HUMAN | 516633.4158 | 0.52 | 0.222 | 4.69E-08 |
| sp|P50552|VASP_HUMAN | 39958.14268 | 1.03 | 0.183 | 0.664 |
| sp|Q6NSJ0|MYORG_HUMAN | 81416.48019 | 0.75 | 0.171 | 3.96E-07 |
| sp|O43399|TPD54_HUMAN | 22263.28191 | 1.57 | 0.289 | 5.02E-12 |
| sp|P30048|PRDX3_HUMAN | 27999.29387 | 1.26 | 0.177 | 4.32E-08 |
| sp|Q15631|TSN_HUMAN | 26262.81029 | 0.55 | 0.072 | 2.20E-16 |
| sp|Q9NWD8|TM248_HUMAN | 35467.66576 | 0.56 | 0.407 | 4.21E-05 |
| sp|Q32MZ4|LRRF1_HUMAN | 89807.95541 | 1.35 | 0.195 | 2.87E-10 |
| sp|Q6W4X9|MUC6_HUMAN | 263141.4446 | 0.69 | 0.379 | 0.001384 |
| sp|Q92805|GOGA1_HUMAN | 88226.21037 | 1.07 | 0.112 | 0.01081 |
| sp|P08729|K2C7_HUMAN | 51393.3185 | 1.79 | 1.125 | 0.01546 |
| sp|Q96D15|RCN3_HUMAN | 37451.98567 | 1.52 | 0.361 | 1.75E-08 |
| sp|Q9H0W9|CK054_HUMAN | 35589.79709 | 1.04 | 0.176 | 0.56 |
| sp|Q8WXD5|GEMI6_HUMAN | 18965.33598 | 1.04 | 0.128 | 0.1809 |
| sp|P23919|KTHY_HUMAN | 23957.51449 | 0.88 | 0.137 | 0.0002419 |
| sp|P31947|1433S_HUMAN | 27852.72578 | 1.56 | 0.759 | 0.007427 |
| sp|Q9H008|LHPP_HUMAN | 29413.97939 | 1.16 | 0.25 | 0.006512 |
| sp|P80217|IN35_HUMAN | 31679.72864 | 1.22 | 0.391 | 0.009301 |
| sp|Q7Z3Y7|K1C28_HUMAN | 51145.1448 | 1.01 | 0.137 | 0.9398 |
| sp|Q9Y224|RTRAF_HUMAN | 28146.73951 | 1.11 | 0.131 | 0.0004487 |
| sp|P13798|ACPH_HUMAN | 82123.96587 | 0.9 | 0.166 | 0.004304 |
| sp|Q7L576|CYFP1_HUMAN | 146724.3429 | 0.6 | 0.099 | 3.72E-14 |
| sp|Q9H3K6|BOLA2_HUMAN | 10263.23205 | 1.09 | 0.206 | 0.06823 |
| sp|Q96EK6|GNA1_HUMAN | 21059.74694 | 0.79 | 0.224 | 5.43E-05 |
| sp|Q9BU76|MMTA2_HUMAN | 29661.03709 | 1.4 | 0.304 | 1.37E-07 |
| sp|Q96I25|SPF45_HUMAN | 45143.99148 | 1.34 | 0.141 | 1.89E-12 |
| sp|Q9Y231|FUT9_HUMAN | 42539.14764 | 0.7 | 0.306 | 0.0005948 |
| sp|Q9NVS2|RT18A_HUMAN | 22493.90696 | 1.07 | 0.227 | 0.2828 |
| sp|Q53TQ3|IN80D_HUMAN | 99348.88246 | 1.29 | 0.262 | 5.90E-06 |
| sp|Q13151|ROA0_HUMAN | 30974.83891 | 1.91 | 1.063 | 8.24E-05 |
| sp|O76074|PDE5A_HUMAN | 101328.6377 | 0.77 | 0.097 | 6.92E-11 |
| sp|Q9ULP9|TBC24_HUMAN | 64001.32876 | 0.67 | 0.165 | 1.86E-08 |
| sp|P01889|1B07_HUMAN | 40759.06674 | 1.17 | 0.62 | 0.5716 |
| sp|O00442|RTCA_HUMAN | 39806.58663 | 0.45 | 0.075 | 2.20E-16 |
| sp|Q13813|SPTN1_HUMAN | 285144.4999 | 1.65 | 0.388 | 1.83E-10 |
| sp|Q96A19|C102A_HUMAN | 62768.12344 | 1.51 | 0.374 | 1.13E-08 |
| sp|O14681|EI24_HUMAN | 39206.75105 | 0.86 | 0.38 | 0.01159 |
| sp|O94992|HEXI1_HUMAN | 40865.57368 | 1.06 | 0.337 | 0.8519 |
| sp|Q9BSY4|CHCH5_HUMAN | 12825.05893 | 0.91 | 0.172 | 0.01334 |
| sp|P55287|CAD11_HUMAN | 88348.96319 | 1.44 | 0.362 | 6.19E-07 |
| sp|Q5TCZ1|SPD2A_HUMAN | 125878.7881 | 1.74 | 0.317 | 6.81E-13 |
| sp|Q53SF7|COBL1_HUMAN | 132428.411 | 1.12 | 0.259 | 0.08783 |
| sp|Q9UKL6|PPCT_HUMAN | 25037.3989 | 1.05 | 0.213 | 0.5665 |
| sp|Q15025|TNIP1_HUMAN | 72086.68263 | 1.01 | 0.101 | 0.8321 |
| sp|P21912|SDHB_HUMAN | 32389.12951 | 1.04 | 0.165 | 0.4505 |
| sp|Q6P3X3|TTC27_HUMAN | 97864.71139 | 0.5 | 0.072 | 2.20E-16 |
| sp|P84098|RL19_HUMAN | 23547.27896 | 1.35 | 0.279 | 3.73E-07 |
| sp|P07358|CO8B_HUMAN | 68696.10645 | 0.88 | 0.28 | 0.0119 |
| sp|Q99933|BAG1_HUMAN | 38965.10997 | 1.08 | 0.146 | 0.01178 |
| sp|Q5TEJ8|THMS2_HUMAN | 72783.34147 | 0.92 | 0.254 | 0.04484 |
| sp|Q9NX40|OCAD1_HUMAN | 27761.82791 | 1.07 | 0.207 | 0.2464 |
| sp|Q96NY7|CLIC6_HUMAN | 73177.78518 | 0.64 | 0.152 | 1.77E-09 |
| sp|P28340|DPOD1_HUMAN | 125017.1483 | 0.77 | 0.178 | 1.97E-06 |
| sp|O15056|SYNJ2_HUMAN | 166557.0915 | 0.81 | 0.333 | 0.0008674 |
| sp|P40616|ARL1_HUMAN | 20500.47062 | 0.41 | 0.129 | 1.47E-13 |
| sp|Q8TCT0|CERK1_HUMAN | 61175.53294 | 1.33 | 0.276 | 6.11E-06 |
| sp|Q969T7|5NT3B_HUMAN | 34634.49967 | 0.69 | 0.136 | 1.69E-09 |
| sp|Q9NW82|WDR70_HUMAN | 73878.30849 | 1.01 | 0.138 | 0.8785 |
| sp|Q8N5M4|TTC9C_HUMAN | 20096.21125 | 1.14 | 0.148 | 0.0001498 |
| sp|Q9BRR3|TM246_HUMAN | 46995.92692 | 1.1 | 0.154 | 0.004589 |
| sp|Q9NRY5|F1142_HUMAN | 55701.29059 | 1.2 | 0.157 | 6.56E-07 |
| sp|O60218|AK1BA_HUMAN | 36206.91212 | 0.7 | 0.396 | 0.00125 |
| sp|Q9UHQ9|NB5R1_HUMAN | 34226.23521 | 0.6 | 0.131 | 3.85E-11 |
| sp|P43897|EFTS_HUMAN | 35692.36449 | 1.01 | 0.166 | 0.9143 |
| sp|Q14108|SCRB2_HUMAN | 54693.61686 | 0.49 | 0.16 | 6.71E-11 |
| sp|O43172|PRP4_HUMAN | 59078.64437 | 1.15 | 0.083 | 6.63E-10 |
| sp|Q96KC8|DNJC1_HUMAN | 64167.4652 | 1.14 | 0.25 | 0.01655 |
| sp|P61586|RHOA_HUMAN | 22078.19042 | 0.66 | 0.086 | 9.09E-15 |
| sp|P62191|PRS4_HUMAN | 49306.74749 | 1.27 | 0.124 | 1.74E-11 |
| sp|Q76FK4|NOL8_HUMAN | 132429.7884 | 1.31 | 0.244 | 2.55E-07 |
| sp|Q9NYB9|ABI2_HUMAN | 55667.73525 | 1.26 | 0.272 | 4.92E-05 |
| sp|P24941|CDK2_HUMAN | 34061.07353 | 0.67 | 0.097 | 1.04E-12 |
| sp|O15067|PUR4_HUMAN | 146278.5021 | 0.81 | 0.108 | 3.78E-08 |
| sp|Q12906|ILF3_HUMAN | 95660.21226 | 0.99 | 0.069 | 0.2348 |
| sp|Q92876|KLK6_HUMAN | 27504.60295 | 2.33 | 1.97 | 0.001586 |
| sp|P58335|ANTR2_HUMAN | 54241.32999 | 0.91 | 0.177 | 0.00818 |
| sp|P05067|A4_HUMAN | 87896.43057 | 1.03 | 0.144 | 0.4656 |
| sp|Q9H330|TM245_HUMAN | 97790.41515 | 0.69 | 0.213 | 6.04E-07 |
| sp|Q86UD1|OAF_HUMAN | 31220.84466 | 0.92 | 0.252 | 0.05175 |
| sp|P11717|MPRI_HUMAN | 281137.3575 | 0.75 | 0.117 | 1.36E-09 |
| sp|Q8N4B1|SESQ1_HUMAN | 27408.1101 | 1.09 | 0.199 | 0.03843 |
| sp|Q9BSF4|TIM29_HUMAN | 29482.18457 | 1.09 | 0.368 | 0.5739 |
| sp|P80108|PHLD_HUMAN | 92887.18645 | 0.98 | 0.254 | 0.3075 |
| sp|Q9GZZ1|NAA50_HUMAN | 19596.11346 | 0.88 | 0.132 | 0.0001594 |
| sp|P37173|TGFR2_HUMAN | 65933.46159 | 0.88 | 0.087 | 1.31E-06 |
| sp|Q99986|VRK1_HUMAN | 45771.55393 | 0.79 | 0.221 | 1.43E-05 |
| sp|Q9Y487|VPP2_HUMAN | 99083.74244 | 0.6 | 0.103 | 4.24E-14 |
| sp|Q13233|M3K1_HUMAN | 166401.0987 | 0.95 | 0.416 | 0.1237 |
| sp|O94919|ENDD1_HUMAN | 55704.53372 | 0.83 | 0.165 | 1.93E-05 |
| sp|Q6ZVF9|GRIN3_HUMAN | 83339.09739 | 1.34 | 0.252 | 2.67E-07 |
| sp|Q99598|TSNAX_HUMAN | 33187.74569 | 1.34 | 0.166 | 1.40E-11 |
| sp|Q9UHF1|EGFL7_HUMAN | 30720.80895 | 0.97 | 0.224 | 0.2814 |
| sp|Q6ICB0|DESI1_HUMAN | 18347.26394 | 1.19 | 0.577 | 0.3098 |
| sp|Q8NGA1|OR1M1_HUMAN | 35539.5176 | 1.45 | 0.302 | 3.51E-08 |
| sp|P02730|B3AT_HUMAN | 101994.509 | 1 | 0.241 | 0.6172 |
| sp|P58107|EPIPL_HUMAN | 557749.8399 | 0.86 | 0.254 | 0.005239 |
| sp|O75391|SPAG7_HUMAN | 26114.26919 | 1.21 | 0.199 | 1.66E-05 |
| sp|Q6ECI4|ZN470_HUMAN | 85030.87755 | 1.25 | 0.396 | 0.01621 |
| sp|Q01518|CAP1_HUMAN | 52306.85379 | 1.13 | 0.107 | 2.17E-06 |
| sp|Q9Y3T9|NOC2L_HUMAN | 85703.46166 | 0.94 | 0.15 | 0.03668 |
| sp|P50402|EMD_HUMAN | 29014.93761 | 1 | 0.182 | 0.5792 |
| sp|Q92499|DDX1_HUMAN | 83331.1098 | 1.09 | 0.101 | 0.0002497 |
| sp|Q9ULZ2|STAP1_HUMAN | 34479.84378 | 1.07 | 0.144 | 0.02474 |
| sp|Q8TAG9|EXOC6_HUMAN | 94385.85453 | 0.65 | 0.111 | 2.41E-12 |
| sp|Q9HCH5|SYTL2_HUMAN | 105132.3333 | 0.98 | 0.298 | 0.316 |
| sp|P42229|STA5A_HUMAN | 91142.53135 | 0.83 | 0.166 | 2.27E-05 |
| sp|Q8TBH0|ARRD2_HUMAN | 44675.31597 | 0.48 | 0.217 | 3.08E-08 |
| sp|P04818|TYSY_HUMAN | 35960.25643 | 0.83 | 0.232 | 0.0003391 |
| sp|O43252|PAPS1_HUMAN | 71567.9218 | 1.23 | 0.159 | 4.79E-08 |
| sp|Q9UHG3|PCYOX_HUMAN | 56984.8849 | 0.7 | 0.098 | 2.66E-12 |
| sp|Q9UEW8|STK39_HUMAN | 59931.66994 | 0.74 | 0.132 | 3.50E-09 |
| sp|Q9H0N5|PHS2_HUMAN | 14395.40412 | 1.11 | 0.092 | 1.68E-06 |
| sp|Q9BTE7|DCNL5_HUMAN | 27814.71821 | 1.16 | 0.222 | 0.003075 |
| sp|Q99615|DNJC7_HUMAN | 57184.96229 | 1.11 | 0.283 | 0.1608 |
| sp|Q9HBH0|RHOF_HUMAN | 23991.36313 | 0.58 | 0.081 | 4.13E-16 |
| sp|Q9H9J2|RM44_HUMAN | 37835.82979 | 0.77 | 0.109 | 1.62E-09 |
| sp|P08571|CD14_HUMAN | 40659.97066 | 1.01 | 0.23 | 0.7944 |
| sp|Q969J3|BORC5_HUMAN | 22304.3892 | 1.41 | 0.222 | 1.68E-09 |
| sp|P31937|3HIDH_HUMAN | 35686.90164 | 1.21 | 0.249 | 0.000493 |
| sp|Q3LXA3|TKFC_HUMAN | 59234.15314 | 0.63 | 0.158 | 1.74E-09 |
| sp|Q96A57|TM230_HUMAN | 13162.07171 | 0.59 | 0.341 | 1.78E-06 |
| sp|Q5HYJ3|FA76B_HUMAN | 39634.69759 | 1.03 | 0.239 | 0.9876 |
| sp|Q9P2K3|RCOR3_HUMAN | 55643.12345 | 1.25 | 0.339 | 0.001075 |
| sp|Q9NUV9|GIMA4_HUMAN | 37720.45396 | 1.29 | 0.216 | 1.74E-07 |
| sp|P49915|GUAA_HUMAN | 77390.2255 | 1.11 | 0.19 | 0.01153 |
| sp|Q86WN1|FCSD1_HUMAN | 77162.20842 | 1.24 | 0.088 | 1.49E-13 |
| sp|P00748|FA12_HUMAN | 70010.74255 | 0.88 | 0.281 | 0.01573 |
| sp|Q9BYN0|SRXN1_HUMAN | 14289.41554 | 1.11 | 0.24 | 0.06929 |
| sp|P61218|RPAB2_HUMAN | 14508.00196 | 1.62 | 0.259 | 1.89E-13 |
| sp|Q8TBM8|DJB14_HUMAN | 42927.22862 | 0.77 | 0.17 | 4.30E-07 |
| sp|P62917|RL8_HUMAN | 28217.36407 | 1.57 | 0.243 | 4.00E-13 |
| sp|O75648|MTU1_HUMAN | 48266.4326 | 0.85 | 0.094 | 8.68E-08 |
| sp|Q9BV20|MTNA_HUMAN | 39449.46912 | 0.82 | 0.078 | 2.48E-10 |
| sp|P10321|1C07_HUMAN | 41118.12727 | 2.49 | 2.469 | 0.0009277 |
| sp|O00299|CLIC1_HUMAN | 27229.86636 | 0.76 | 0.166 | 2.72E-06 |
| sp|Q14331|FRG1_HUMAN | 29421.21906 | 1.4 | 0.24 | 8.83E-10 |
| sp|P06727|APOA4_HUMAN | 45353.45611 | 1.26 | 0.447 | 0.03833 |
| sp|Q96S52|PIGS_HUMAN | 61713.18493 | 0.68 | 0.06 | 2.20E-16 |
| sp|Q8NB49|AT11C_HUMAN | 130858.6125 | 0.72 | 0.21 | 6.47E-06 |
| sp|Q9UNK0|STX8_HUMAN | 26986.0418 | 0.84 | 0.093 | 1.91E-08 |
| sp|P24844|MYL9_HUMAN | 19853.45876 | 0.75 | 0.276 | 0.0001988 |
| sp|Q7Z569|BRAP_HUMAN | 68554.70652 | 1 | 0.087 | 0.9952 |
| sp|Q9P2P1|NYNRI_HUMAN | 209699.3012 | 0.86 | 0.217 | 0.001676 |
| sp|O75380|NDUS6_HUMAN | 14026.94099 | 1.11 | 0.272 | 0.1811 |
| sp|Q15404|RSU1_HUMAN | 31502.68129 | 1.29 | 0.212 | 2.02E-07 |
| sp|Q6PKC3|TXD11_HUMAN | 111809.5257 | 0.78 | 0.161 | 1.78E-06 |
| sp|O75410|TACC1_HUMAN | 88577.52849 | 1.41 | 0.188 | 3.09E-12 |
| sp|P27824|CALX_HUMAN | 67964.01139 | 0.71 | 0.114 | 5.98E-11 |
| sp|Q5JS37|NHLC3_HUMAN | 38525.58878 | 1.14 | 0.275 | 0.02873 |
| sp|Q5TBC7|B2L15_HUMAN | 17980.78099 | 0.69 | 0.474 | 0.0002748 |
| sp|P34913|HYES_HUMAN | 63297.94246 | 0.84 | 0.248 | 0.001622 |
| sp|Q8IZ69|TRM2A_HUMAN | 69690.56693 | 1.04 | 0.188 | 0.6089 |
| sp|O00231|PSD11_HUMAN | 47701.1374 | 0.81 | 0.115 | 6.67E-08 |
| sp|O75844|FACE1_HUMAN | 55044.62894 | 0.53 | 0.099 | 1.23E-14 |
| sp|P04406|G3P_HUMAN | 36183.45172 | 1.13 | 0.147 | 0.0002087 |
| sp|Q15125|EBP_HUMAN | 26545.83158 | 0.63 | 0.101 | 2.06E-13 |
| sp|Q9NR30|DDX21_HUMAN | 87785.56322 | 0.97 | 0.221 | 0.215 |
| sp|P08648|ITA5_HUMAN | 115587.1554 | 0.63 | 0.243 | 2.15E-06 |
| sp|P49888|ST1E1_HUMAN | 35313.66106 | 1.01 | 0.366 | 0.4783 |
| sp|Q9NTJ3|SMC4_HUMAN | 147756.7641 | 0.98 | 0.281 | 0.3175 |
| sp|P12081|SYHC_HUMAN | 57926.42361 | 1.07 | 0.157 | 0.06226 |
| sp|Q9UEY8|ADDG_HUMAN | 79429.28694 | 0.84 | 0.286 | 0.003284 |
| sp|O00170|AIP_HUMAN | 38050.2315 | 1.39 | 0.245 | 1.63E-09 |
| sp|P22692|IBP4_HUMAN | 29094.90765 | 0.96 | 0.126 | 0.08094 |
| sp|Q6JBY9|CPZIP_HUMAN | 44744.10714 | 1.37 | 0.216 | 4.29E-10 |
| sp|Q9Y2Q3|GSTK1_HUMAN | 25576.33032 | 0.9 | 0.303 | 0.02205 |
| sp|P14543|NID1_HUMAN | 139123.7744 | 0.97 | 0.25 | 0.23 |
| sp|Q99426|TBCB_HUMAN | 27575.59434 | 1.2 | 0.241 | 0.0007027 |
| sp|Q96PD5|PGRP2_HUMAN | 62730.08724 | 0.7 | 0.112 | 2.48E-11 |
| sp|Q9NXA8|SIR5_HUMAN | 34525.39728 | 0.91 | 0.087 | 2.63E-05 |
| sp|Q56VL3|OCAD2_HUMAN | 17266.69071 | 0.91 | 0.242 | 0.03038 |
| sp|O75150|BRE1B_HUMAN | 114331.8962 | 1.16 | 0.072 | 1.44E-11 |
| sp|Q8NF37|PCAT1_HUMAN | 59722.61124 | 0.74 | 0.098 | 2.71E-11 |
| sp|Q96IZ7|RSRC1_HUMAN | 38636.38788 | 1.25 | 0.233 | 2.94E-05 |
| sp|Q9GZM5|YIPF3_HUMAN | 38432.66094 | 0.53 | 0.142 | 1.46E-12 |
| sp|Q13459|MYO9B_HUMAN | 244827.8634 | 1.1 | 0.129 | 0.001015 |
| sp|P61513|RL37A_HUMAN | 10478.54993 | 1.33 | 0.23 | 1.04E-07 |
| sp|Q12933|TRAF2_HUMAN | 57572.349 | 0.62 | 0.091 | 9.98E-15 |
| sp|P01350|GAST_HUMAN | 11425.60676 | 0.92 | 0.454 | 0.06566 |
| sp|Q9NWT8|AKIP_HUMAN | 22550.20412 | 1.16 | 0.251 | 0.01829 |
| sp|O43156|TTI1_HUMAN | 123456.2418 | 1.21 | 0.255 | 0.0004285 |
| sp|O60244|MED14_HUMAN | 161968.5681 | 0.68 | 0.102 | 9.88E-12 |
| sp|P09769|FGR_HUMAN | 60049.76861 | 0.69 | 0.279 | 1.48E-05 |
| sp|P19397|CD53_HUMAN | 24990.7774 | 0.89 | 0.129 | 0.0001618 |
| sp|P15374|UCHL3_HUMAN | 26319.02737 | 1.37 | 0.223 | 1.60E-09 |
| sp|Q6P582|MZT2A_HUMAN | 16249.49917 | 1.45 | 0.386 | 1.75E-06 |
| sp|P11586|C1TC_HUMAN | 102161.5528 | 1.12 | 0.174 | 0.002945 |
| sp|P62070|RRAS2_HUMAN | 23594.79886 | 1.53 | 0.417 | 1.37E-07 |
| sp|Q15149|PLEC_HUMAN | 533443.7276 | 1.04 | 0.181 | 0.4199 |
| sp|O43504|LTOR5_HUMAN | 9760.771221 | 1.24 | 0.174 | 1.54E-07 |
| sp|Q6ZVM7|TM1L2_HUMAN | 55845.8579 | 0.85 | 0.136 | 1.31E-05 |
| sp|P51151|RAB9A_HUMAN | 23090.26325 | 1.16 | 0.163 | 2.72E-05 |
| sp|O00264|PGRC1_HUMAN | 21753.78971 | 1.32 | 0.249 | 7.50E-07 |
| sp|Q06265|EXOS9_HUMAN | 49527.32777 | 0.7 | 0.237 | 3.37E-06 |
| sp|Q68E01|INT3_HUMAN | 119515.4249 | 0.84 | 0.097 | 5.03E-08 |
| sp|Q9NYB0|TE2IP_HUMAN | 44385.87347 | 1.11 | 0.188 | 0.01747 |
| sp|Q7Z3K3|POGZ_HUMAN | 157336.697 | 0.93 | 0.108 | 0.003175 |
| sp|Q9BYX4|IFIH1_HUMAN | 117908.1963 | 1.08 | 0.251 | 0.3215 |
| sp|Q9NR31|SAR1A_HUMAN | 22448.56204 | 0.95 | 0.046 | 4.93E-05 |
| sp|Q3ZAQ7|VMA21_HUMAN | 11328.85447 | 0.75 | 0.126 | 9.63E-09 |
| sp|Q8NB90|AFG2H_HUMAN | 98452.16175 | 1.28 | 0.108 | 3.30E-13 |
| sp|Q9H0N0|RAB6C_HUMAN | 28547.48043 | 0.73 | 0.198 | 6.34E-07 |
| sp|P62820|RAB1A_HUMAN | 22873.45466 | 0.71 | 0.171 | 4.98E-08 |
| sp|Q6L8Q7|PDE12_HUMAN | 68203.26055 | 1.01 | 0.073 | 0.5507 |
| sp|O14807|RASM_HUMAN | 23870.44493 | 1.08 | 0.187 | 0.07121 |
| sp|Q9BRX5|PSF3_HUMAN | 24558.20344 | 1.2 | 0.216 | 6.74E-05 |
| sp|Q15599|NHRF2_HUMAN | 37601.04249 | 1.16 | 0.244 | 0.004198 |
| sp|Q9P035|HACD3_HUMAN | 43341.63142 | 0.69 | 0.178 | 1.83E-07 |
| sp|P25311|ZA2G_HUMAN | 34447.18001 | 1.22 | 0.378 | 0.01026 |
| sp|Q8NBS9|TXND5_HUMAN | 48264.90801 | 1.4 | 0.352 | 3.50E-06 |
| sp|Q9BQI0|AIF1L_HUMAN | 17095.63241 | 1.67 | 0.644 | 7.64E-06 |
| sp|P49750|YLPM1_HUMAN | 241704.8392 | 1.34 | 0.066 | 2.20E-16 |
| sp|Q9UHL4|DPP2_HUMAN | 54745.36187 | 0.86 | 0.191 | 0.0006306 |
| sp|Q6BCY4|NB5R2_HUMAN | 31477.38293 | 1.21 | 0.249 | 0.0008052 |
| sp|Q9P2I0|CPSF2_HUMAN | 89268.02837 | 0.78 | 0.115 | 5.44E-09 |
| sp|Q9Y3X0|CCDC9_HUMAN | 59763.09098 | 1.35 | 0.381 | 0.000105 |
| sp|Q9BVM4|GGACT_HUMAN | 17413.79308 | 1.25 | 0.588 | 0.2362 |
| sp|P49406|RM19_HUMAN | 33780.65027 | 0.68 | 0.183 | 3.31E-08 |
| sp|Q6PD74|AAGAB_HUMAN | 34896.02823 | 1.17 | 0.188 | 8.86E-05 |
| sp|Q13107|UBP4_HUMAN | 109960.4856 | 1.07 | 0.082 | 0.000246 |
| sp|Q9BZF2|OSBL7_HUMAN | 96380.04267 | 1.09 | 0.263 | 0.2985 |
| sp|Q08477|CP4F3_HUMAN | 60645.17524 | 0.75 | 0.258 | 2.55E-05 |
| sp|Q9H9G7|AGO3_HUMAN | 98477.43285 | 0.98 | 0.104 | 0.1884 |
| sp|O95619|YETS4_HUMAN | 26521.72062 | 1.07 | 0.084 | 0.0005456 |
| sp|Q9NR09|BIRC6_HUMAN | 536173.68 | 0.95 | 0.117 | 0.02204 |
| sp|P31327|CPSM_HUMAN | 165957.2261 | 0.54 | 0.276 | 1.62E-06 |
| sp|Q9H7D0|DOCK5_HUMAN | 216180.4312 | 0.7 | 0.118 | 7.12E-11 |
| sp|Q13049|TRI32_HUMAN | 73520.98926 | 1.04 | 0.138 | 0.2332 |
| sp|Q8TB36|GDAP1_HUMAN | 41529.70372 | 0.51 | 0.128 | 1.96E-13 |
| sp|Q9Y3L3|3BP1_HUMAN | 75989.93596 | 1.19 | 0.181 | 6.98E-06 |
| sp|O75534|CSDE1_HUMAN | 89666.18345 | 1.22 | 0.163 | 1.78E-07 |
| sp|O95428|PPN_HUMAN | 141585.6772 | 1.22 | 0.251 | 0.0002783 |
| sp|P22392|NDKB_HUMAN | 17382.97344 | 0.91 | 0.13 | 0.001029 |
| sp|O15173|PGRC2_HUMAN | 23842.74118 | 0.79 | 0.19 | 2.40E-05 |
| sp|Q9UBD5|ORC3_HUMAN | 83266.85422 | 0.66 | 0.19 | 2.56E-07 |
| sp|Q9Y6D9|MD1L1_HUMAN | 83282.70641 | 1.4 | 0.13 | 1.86E-15 |
| sp|P14672|GLUT4_HUMAN | 54905.41246 | 0.72 | 0.096 | 6.21E-12 |
| sp|Q96QH2|PRAM_HUMAN | 74019.02511 | 1.2 | 0.311 | 0.01427 |
| sp|P22897|MRC1_HUMAN | 168851.8654 | 1 | 0.283 | 0.4812 |
| sp|Q9Y6J9|TAF6L_HUMAN | 68324.18587 | 0.94 | 0.202 | 0.06145 |
| sp|O14979|HNRDL_HUMAN | 46562.14074 | 1.53 | 0.255 | 3.59E-11 |
| sp|Q96F07|CYFP2_HUMAN | 150280.0884 | 0.65 | 0.139 | 1.77E-10 |
| sp|O15042|SR140_HUMAN | 118656.7638 | 0.65 | 0.071 | 2.20E-16 |
| sp|Q9HCU5|PREB_HUMAN | 45991.87009 | 0.71 | 0.109 | 2.59E-11 |
| sp|Q9Y394|DHRS7_HUMAN | 38655.25764 | 0.46 | 0.125 | 2.54E-12 |
| sp|Q9Y4P8|WIPI2_HUMAN | 50157.08067 | 0.95 | 0.071 | 0.001439 |
| sp|Q99439|CNN2_HUMAN | 34056.43994 | 1.73 | 0.541 | 1.91E-07 |
| sp|Q5SXM8|DNLZ_HUMAN | 19459.01848 | 1.01 | 0.132 | 0.9462 |
| sp|Q5T5U3|RHG21_HUMAN | 218679.7606 | 1.24 | 0.54 | 0.06763 |
| sp|Q9H845|ACAD9_HUMAN | 69325.95742 | 0.68 | 0.098 | 1.26E-12 |
| sp|Q9H9A6|LRC40_HUMAN | 68702.30692 | 0.64 | 0.086 | 1.61E-14 |
| sp|Q8IWA5|CTL2_HUMAN | 81592.09341 | 0.67 | 0.206 | 5.94E-07 |
| sp|Q9BU40|CRDL1_HUMAN | 53054.4315 | 1.67 | 0.532 | 1.63E-07 |
| sp|O14618|CCS_HUMAN | 29517.54437 | 1.3 | 0.177 | 1.04E-09 |
| sp|P12955|PEPD_HUMAN | 55293.19704 | 1.1 | 0.119 | 0.0007633 |
| sp|Q9NW13|RBM28_HUMAN | 86179.92115 | 1.18 | 0.218 | 0.001227 |
| sp|E7EW31|PROB1_HUMAN | 107289.9355 | 0.48 | 0.273 | 6.20E-07 |
| sp|Q8IXK2|GLT12_HUMAN | 67733.11245 | 0.67 | 0.191 | 4.59E-07 |
| sp|P16050|LOX15_HUMAN | 75479.78419 | 0.58 | 0.186 | 3.91E-08 |
| sp|Q969E8|TSR2_HUMAN | 21091.01725 | 0.87 | 0.21 | 0.002184 |
| sp|Q5T2E6|ARMD3_HUMAN | 79496.71372 | 0.67 | 0.069 | 2.27E-16 |
| sp|P51149|RAB7A_HUMAN | 23741.93802 | 0.98 | 0.119 | 0.3699 |
| sp|Q9NUQ2|PLCE_HUMAN | 42369.04175 | 0.73 | 0.148 | 3.39E-08 |
| sp|P21741|MK_HUMAN | 16127.22353 | 1.66 | 1.061 | 0.008968 |
| sp|P03952|KLKB1_HUMAN | 73414.62655 | 0.68 | 0.188 | 3.10E-07 |
| sp|Q9BQ61|TRIR_HUMAN | 18447.24512 | 1.72 | 0.797 | 0.0001358 |
| sp|O00232|PSD12_HUMAN | 53251.71282 | 0.55 | 0.087 | 2.49E-16 |
| sp|Q01484|ANK2_HUMAN | 435939.1969 | 1.37 | 0.283 | 2.12E-07 |
| sp|Q9ULE0|WWC3_HUMAN | 123722.5648 | 1.11 | 0.164 | 0.00243 |
| sp|Q96JI7|SPTCS_HUMAN | 282663.0199 | 0.78 | 0.096 | 5.95E-10 |
| sp|Q8TD22|SFXN5_HUMAN | 37253.78988 | 0.82 | 0.167 | 1.95E-05 |
| sp|Q9UKK9|NUDT5_HUMAN | 24579.33343 | 1.13 | 0.153 | 0.0004665 |
| sp|Q14242|SELPL_HUMAN | 43327.49913 | 0.97 | 0.206 | 0.19 |
| sp|Q9UHD2|TBK1_HUMAN | 84198.04332 | 0.73 | 0.091 | 2.44E-12 |
| sp|O75954|TSN9_HUMAN | 27540.76441 | 0.71 | 0.136 | 4.81E-10 |
| sp|Q96T17|MA7D2_HUMAN | 82353.3293 | 0.77 | 0.179 | 3.04E-06 |
| sp|O75970|MPDZ_HUMAN | 222774.1539 | 1.06 | 0.164 | 0.1269 |
| sp|O75131|CPNE3_HUMAN | 60929.43851 | 0.59 | 0.105 | 2.40E-13 |
| sp|Q9NZK5|ADA2_HUMAN | 59162.28642 | 0.77 | 0.179 | 2.90E-06 |
| sp|Q8TBK6|ZCH10_HUMAN | 21107.18932 | 1.13 | 0.214 | 0.009123 |
| sp|Q8WW01|SEN15_HUMAN | 18782.23539 | 1.31 | 0.146 | 2.04E-11 |
| sp|P52306|GDS1_HUMAN | 67054.81859 | 0.7 | 0.092 | 3.12E-13 |
| sp|Q9Y3D6|FIS1_HUMAN | 16966.02924 | 0.85 | 0.25 | 0.00239 |
| sp|Q8N3F0|MTURN_HUMAN | 15182.8588 | 1.16 | 0.285 | 0.02059 |
| sp|P62081|RS7_HUMAN | 22095.24738 | 1.4 | 0.153 | 1.17E-13 |
| sp|O14639|ABLM1_HUMAN | 89494.78193 | 1.09 | 0.099 | 0.000218 |
| sp|P06703|S10A6_HUMAN | 10212.35344 | 1.26 | 0.243 | 2.72E-05 |
| sp|P30084|ECHM_HUMAN | 31805.29215 | 1.36 | 0.277 | 2.67E-07 |
| sp|Q96GY0|ZC21A_HUMAN | 35736.29461 | 1.32 | 0.287 | 6.72E-06 |
| sp|Q9Y287|ITM2B_HUMAN | 30813.55535 | 0.82 | 0.348 | 0.003702 |
| sp|Q8IV50|LYSM2_HUMAN | 23487.97056 | 1.35 | 0.416 | 0.0002124 |
| sp|Q4G0N4|NAKD2_HUMAN | 49954.37587 | 1.03 | 0.134 | 0.5245 |
| sp|Q9BRP8|PYM1_HUMAN | 22623.97672 | 2 | 0.649 | 2.25E-10 |
| sp|Q96LW7|CAR19_HUMAN | 26011.15762 | 1.06 | 0.157 | 0.08227 |
| sp|P24534|EF1B_HUMAN | 24901.31608 | 1.5 | 0.216 | 1.33E-12 |
| sp|Q99798|ACON_HUMAN | 86095.19281 | 1.06 | 0.262 | 0.5466 |
| sp|Q9Y6G9|DC1L1_HUMAN | 56811.09703 | 1.12 | 0.117 | 2.76E-05 |
| sp|Q9NZD2|GLTP_HUMAN | 23987.4847 | 0.52 | 0.098 | 6.82E-15 |
| sp|O75122|CLAP2_HUMAN | 141756.7522 | 0.83 | 0.121 | 5.29E-07 |
| sp|Q9Y619|ORNT1_HUMAN | 33209.89401 | 0.68 | 0.103 | 2.16E-12 |
| sp|O15066|KIF3B_HUMAN | 85168.86539 | 1.12 | 0.117 | 2.94E-05 |
| sp|P30086|PEBP1_HUMAN | 21139.69841 | 1.27 | 0.203 | 1.89E-07 |
| sp|Q9H2H8|PPIL3_HUMAN | 18353.00495 | 1.42 | 0.161 | 2.60E-14 |
| sp|Q96N96|SPT13_HUMAN | 75723.62084 | 1.09 | 0.173 | 0.03266 |
| sp|P62861|RS30_HUMAN | 6625.806172 | 1.15 | 0.284 | 0.05753 |
| sp|Q9Y2W6|TDRKH_HUMAN | 62388.55431 | 0.89 | 0.197 | 0.005679 |
| sp|P49788|TIG1_HUMAN | 33531.31167 | 0.88 | 0.335 | 0.01718 |
| sp|Q14166|TTL12_HUMAN | 75136.32856 | 0.75 | 0.107 | 3.08E-10 |
| sp|P80511|S10AC_HUMAN | 10550.51695 | 1.04 | 0.94 | 0.09244 |
| sp|Q96FN9|DTD2_HUMAN | 18858.771 | 1.3 | 0.286 | 6.68E-06 |
| sp|O60927|PP1RB_HUMAN | 14210.80798 | 0.81 | 0.601 | 0.007095 |
| sp|Q3LI76|KR151_HUMAN | 15805.71765 | 0.6 | 0.065 | 2.20E-16 |
| sp|Q9BZL6|KPCD2_HUMAN | 97954.17325 | 0.77 | 0.088 | 1.51E-11 |
| sp|Q15654|TRIP6_HUMAN | 51720.0092 | 1.52 | 0.274 | 2.22E-11 |
| sp|Q9NXV6|CARF_HUMAN | 61526.01335 | 1.23 | 0.179 | 6.26E-07 |
| sp|P16189|1A31_HUMAN | 41245.30892 | 1.57 | 0.35 | 9.39E-10 |
| sp|P57076|CF298_HUMAN | 33356.2511 | 1.09 | 0.355 | 0.4797 |
| sp|O60504|VINEX_HUMAN | 75504.98177 | 1.29 | 0.24 | 3.09E-07 |
| sp|Q9NWY4|HPF1_HUMAN | 39792.17312 | 1.13 | 0.161 | 0.0006176 |
| sp|O15116|LSM1_HUMAN | 15152.10716 | 1.24 | 0.127 | 5.01E-10 |
| sp|P0DJI8|SAA1_HUMAN | 13562.5292 | 1.38 | 0.627 | 0.008478 |
| sp|P59190|RAB15_HUMAN | 24642.28481 | 0.87 | 0.146 | 7.56E-05 |
| sp|Q96E17|RAB3C_HUMAN | 26145.70071 | 0.46 | 0.183 | 6.26E-10 |
| sp|Q96T76|MMS19_HUMAN | 114909.6325 | 0.71 | 0.073 | 5.55E-15 |
| sp|Q9H7F0|AT133_HUMAN | 139759.6511 | 0.61 | 0.245 | 6.83E-08 |
| sp|P30050|RL12_HUMAN | 17960.59428 | 0.98 | 0.087 | 0.227 |
| sp|P85037|FOXK1_HUMAN | 75848.80081 | 1.08 | 0.123 | 0.008952 |
| sp|Q8IVI9|NOSTN_HUMAN | 58118.66832 | 0.93 | 0.192 | 0.03549 |
| sp|Q9Y6G5|COMDA_HUMAN | 22991.12797 | 1.02 | 0.162 | 0.8538 |
| sp|P25686|DNJB2_HUMAN | 35654.43632 | 1.26 | 0.268 | 6.48E-05 |
| sp|Q9H6E5|STPAP_HUMAN | 94967.82945 | 1.58 | 0.397 | 1.55E-08 |
| sp|Q14957|NMDE3_HUMAN | 135587.8476 | 1.38 | 0.401 | 1.77E-05 |
| sp|O00255|MEN1_HUMAN | 68362.0236 | 1.1 | 0.253 | 0.1635 |
| sp|Q8TED0|UTP15_HUMAN | 58702.67743 | 0.85 | 0.107 | 4.18E-07 |
| sp|Q5H9R7|PP6R3_HUMAN | 98558.98951 | 0.49 | 0.103 | 9.05E-15 |
| sp|Q7Z449|CP2U1_HUMAN | 62385.47441 | 0.57 | 0.138 | 1.84E-11 |
| sp|Q96S94|CCNL2_HUMAN | 58606.65679 | 0.8 | 0.07 | 3.71E-12 |
| sp|Q9NZQ3|SPN90_HUMAN | 79633.39091 | 0.78 | 0.098 | 2.43E-10 |
| sp|Q9UHR5|S30BP_HUMAN | 33945.70216 | 1.41 | 0.248 | 2.51E-09 |
| sp|P46777|RL5_HUMAN | 34550.76943 | 1.34 | 0.165 | 4.73E-11 |
| sp|Q8NCL4|GALT6_HUMAN | 72007.44765 | 0.49 | 0.277 | 1.79E-06 |
| sp|Q96B54|ZN428_HUMAN | 20849.09123 | 1.67 | 0.366 | 1.29E-10 |
| sp|Q7Z3B4|NUP54_HUMAN | 55497.38547 | 1.4 | 0.118 | 2.69E-16 |
| sp|Q9NXL9|MCM9_HUMAN | 128697.1078 | 1.01 | 0.213 | 0.8689 |
| sp|Q8IVB5|LIX1L_HUMAN | 36806.6024 | 1.29 | 0.217 | 1.28E-07 |
| sp|Q6ZS17|RIPR1_HUMAN | 133404.8987 | 0.91 | 0.178 | 0.005083 |
| sp|P08651|NFIC_HUMAN | 56078.20756 | 0.93 | 0.21 | 0.04272 |
| sp|P0C0S5|H2AZ_HUMAN | 13526.54148 | 1.16 | 0.131 | 1.51E-06 |
| sp|Q9BRG1|VPS25_HUMAN | 20830.54184 | 0.76 | 0.085 | 4.13E-12 |
| sp|Q7Z7L8|CK096_HUMAN | 46751.59965 | 1.42 | 0.656 | 0.02202 |
| sp|Q8NFH5|NUP35_HUMAN | 34847.49132 | 1.44 | 0.262 | 5.94E-10 |
| sp|P04637|P53_HUMAN | 44177.58227 | 1.01 | 0.269 | 0.6993 |
| sp|Q49AR2|CE022_HUMAN | 50487.67393 | 1.07 | 0.165 | 0.09064 |
| sp|P13807|GYS1_HUMAN | 84512.75928 | 0.67 | 0.09 | 8.70E-14 |
| sp|O14929|HAT1_HUMAN | 49861.92906 | 0.73 | 0.173 | 2.69E-07 |
| sp|P35610|SOAT1_HUMAN | 65187.45725 | 0.83 | 0.275 | 0.0007731 |
| sp|Q92974|ARHG2_HUMAN | 112367.6844 | 0.93 | 0.107 | 0.001344 |
| sp|Q9NWQ9|CN119_HUMAN | 16094.91938 | 1.4 | 0.316 | 1.95E-07 |
| sp|Q96QC0|PP1RA_HUMAN | 99320.08896 | 1.19 | 0.258 | 0.002096 |
| sp|Q9BPX5|ARP5L_HUMAN | 16912.80061 | 1.45 | 0.198 | 9.51E-13 |
| sp|Q96EU7|C1GLC_HUMAN | 36739.10307 | 0.68 | 0.254 | 4.63E-06 |
| sp|Q6NY19|KANK3_HUMAN | 88980.63946 | 1.63 | 0.247 | 1.35E-14 |
| sp|Q13884|SNTB1_HUMAN | 58349.17232 | 0.7 | 0.204 | 1.93E-06 |
| sp|O75676|KS6A4_HUMAN | 86104.45434 | 0.84 | 0.125 | 1.34E-06 |
| sp|O95478|NSA2_HUMAN | 30199.56065 | 1.41 | 0.16 | 1.26E-13 |
| sp|Q13868|EXOS2_HUMAN | 32978.20183 | 1.1 | 0.104 | 4.57E-05 |
| sp|P26374|RAE2_HUMAN | 75260.63244 | 0.9 | 0.252 | 0.01675 |
| sp|P19525|E2AK2_HUMAN | 62493.78534 | 1.25 | 0.198 | 1.30E-06 |
| sp|Q16401|PSMD5_HUMAN | 56541.49918 | 0.87 | 0.098 | 1.80E-06 |
| sp|O14617|AP3D1_HUMAN | 131141.1768 | 0.72 | 0.125 | 7.64E-10 |
| sp|Q8IVH8|M4K3_HUMAN | 102430.6172 | 0.68 | 0.126 | 1.58E-10 |
| sp|P37840|SYUA_HUMAN | 14433.20851 | 1.72 | 0.262 | 2.64E-14 |
| sp|P82932|RT06_HUMAN | 14256.60136 | 1.12 | 0.241 | 0.06138 |
| sp|Q86TJ2|TAD2B_HUMAN | 48991.8395 | 1.01 | 0.429 | 0.3351 |
| sp|Q9UNF1|MAGD2_HUMAN | 65067.41346 | 0.78 | 0.207 | 0.0001163 |
| sp|Q9Y697|NFS1_HUMAN | 50545.02363 | 1.21 | 0.174 | 1.41E-06 |
| sp|Q9UFH2|DYH17_HUMAN | 512732.6409 | 0.56 | 0.281 | 3.34E-06 |
| sp|Q86X55|CARM1_HUMAN | 66363.62408 | 0.68 | 0.102 | 3.79E-12 |
| sp|Q9NRG0|CHRC1_HUMAN | 14740.46818 | 1.26 | 0.412 | 0.008982 |
| sp|Q9Y3C5|RNF11_HUMAN | 17813.41676 | 0.7 | 0.269 | 1.36E-05 |
| sp|Q9HD40|SPCS_HUMAN | 56413.7561 | 5.13 | 2.441 | 2.47E-14 |
| sp|Q9H269|VPS16_HUMAN | 95641.94886 | 0.65 | 0.095 | 9.26E-14 |
| sp|P62851|RS25_HUMAN | 13772.70951 | 1.29 | 0.219 | 2.71E-07 |
| sp|P10109|ADX_HUMAN | 19647.71187 | 1.39 | 0.26 | 5.45E-09 |
| sp|O15027|SC16A_HUMAN | 253316.1047 | 1.02 | 0.117 | 0.4941 |
| sp|Q9H6T0|ESRP2_HUMAN | 79188.39026 | 1.07 | 0.269 | 0.4385 |
| sp|Q13464|ROCK1_HUMAN | 159084.1189 | 0.94 | 0.067 | 0.0001378 |
| sp|Q2M2I3|FA83E_HUMAN | 51957.89424 | 0.75 | 0.202 | 3.66E-06 |
| sp|Q9NXS2|QPCTL_HUMAN | 43050.17647 | 0.68 | 0.139 | 1.09E-08 |
| sp|Q9BRT3|MIEN1_HUMAN | 12548.22336 | 0.98 | 0.15 | 0.288 |
| sp|O60565|GREM1_HUMAN | 21178.63368 | 0.83 | 0.179 | 6.86E-05 |
| sp|O75821|EIF3G_HUMAN | 35856.01706 | 1.45 | 0.16 | 2.04E-14 |
| sp|Q8NE01|CNNM3_HUMAN | 76510.21861 | 0.61 | 0.132 | 7.59E-12 |
| sp|P08247|SYPH_HUMAN | 34090.53414 | 0.66 | 0.197 | 1.89E-06 |
| sp|P56192|SYMC_HUMAN | 102231.3462 | 0.62 | 0.115 | 2.59E-12 |
| sp|P61952|GBG11_HUMAN | 8571.427793 | 1.6 | 0.428 | 6.84E-09 |
| sp|P05787|K2C8_HUMAN | 53653.12247 | 1.08 | 0.458 | 0.9601 |
| sp|Q96EV8|DTBP1_HUMAN | 39792.30611 | 1.13 | 0.143 | 7.85E-05 |
| sp|Q9Y230|RUVB2_HUMAN | 51277.60815 | 1.27 | 0.088 | 9.73E-15 |
| sp|O00165|HAX1_HUMAN | 31583.04368 | 1.17 | 0.108 | 7.54E-09 |
| sp|Q14739|LBR_HUMAN | 71039.17147 | 0.94 | 0.172 | 0.04156 |
| sp|P08519|APOA_HUMAN | 514719.443 | 1.59 | 0.877 | 0.006655 |
| sp|Q15043|S39AE_HUMAN | 54900.48201 | 0.65 | 0.183 | 5.47E-09 |
| sp|Q3V6T2|GRDN_HUMAN | 216575.3625 | 1.32 | 0.123 | 1.46E-13 |
| sp|Q6ZTI6|RFLA_HUMAN | 23805.00305 | 1.5 | 0.437 | 1.09E-06 |
| sp|Q14676|MDC1_HUMAN | 227651.2074 | 1.04 | 0.169 | 0.4498 |
| sp|P62306|RUXF_HUMAN | 9757.787643 | 0.93 | 0.131 | 0.004672 |
| sp|Q13325|IFIT5_HUMAN | 56249.76621 | 1.08 | 0.304 | 0.4882 |
| sp|O95067|CCNB2_HUMAN | 45519.85665 | 0.9 | 0.186 | 0.006691 |
| sp|Q86WA6|BPHL_HUMAN | 32675.02765 | 1.03 | 0.228 | 0.8919 |
| sp|Q9NPF5|DMAP1_HUMAN | 53112.84995 | 1.2 | 0.117 | 4.13E-09 |
| sp|Q15848|ADIPO_HUMAN | 26493.05164 | 0.94 | 0.237 | 0.07119 |
| sp|P20839|IMDH1_HUMAN | 55751.60292 | 0.92 | 0.061 | 2.30E-06 |
| sp|P15941|MUC1_HUMAN | 122182.1413 | 0.78 | 0.19 | 1.54E-05 |
| sp|P05186|PPBT_HUMAN | 57592.74209 | 0.9 | 0.33 | 0.03083 |
| sp|Q8N1F8|S11IP_HUMAN | 121591.0495 | 1.16 | 0.124 | 9.14E-07 |
| sp|Q68BL8|OLM2B_HUMAN | 84612.87002 | 1.34 | 0.309 | 4.14E-06 |
| sp|O00499|BIN1_HUMAN | 64869.46935 | 1.82 | 0.273 | 2.46E-16 |
| sp|Q9NRN5|OLFL3_HUMAN | 46362.40792 | 2.04 | 1.124 | 1.82E-06 |
| sp|Q96LZ7|RMD2_HUMAN | 47693.29025 | 0.89 | 0.112 | 6.73E-05 |
| sp|P45877|PPIC_HUMAN | 22844.87658 | 0.49 | 0.164 | 5.14E-12 |
| sp|P00709|LALBA_HUMAN | 16652.28541 | 0.75 | 0.289 | 0.0003836 |
| sp|O95835|LATS1_HUMAN | 127285.2135 | 0.98 | 0.234 | 0.312 |
| sp|Q7Z3V4|UBE3B_HUMAN | 124483.7109 | 0.57 | 0.096 | 8.97E-15 |
| sp|P12270|TPR_HUMAN | 267512.2524 | 1.42 | 0.155 | 5.24E-14 |
| sp|P62241|RS8_HUMAN | 24457.25942 | 1.38 | 0.163 | 1.23E-12 |
| sp|Q96DG6|CMBL_HUMAN | 28354.2927 | 0.62 | 0.354 | 3.68E-05 |
| sp|P15502|ELN_HUMAN | 68451.48359 | 1.21 | 0.33 | 0.01007 |
| sp|Q7KZ85|SPT6H_HUMAN | 200185.0601 | 0.96 | 0.097 | 0.03392 |
| sp|Q6GMV2|SMYD5_HUMAN | 48546.39529 | 0.57 | 0.201 | 1.51E-08 |
| sp|P05198|IF2A_HUMAN | 36356.47211 | 1.21 | 0.139 | 2.72E-08 |
| sp|Q14624|ITIH4_HUMAN | 103503.0603 | 1.14 | 0.323 | 0.09984 |
| sp|Q9Y6S9|RPKL1_HUMAN | 60721.81575 | 1.04 | 0.346 | 0.8047 |
| sp|P42285|MTREX_HUMAN | 118737.7586 | 0.58 | 0.079 | 2.20E-16 |
| sp|O75063|XYLK_HUMAN | 46840.98186 | 0.96 | 0.152 | 0.07594 |
| sp|Q9NS69|TOM22_HUMAN | 15493.77407 | 0.7 | 0.102 | 3.92E-12 |
| sp|O95379|TFIP8_HUMAN | 23141.92039 | 0.52 | 0.17 | 6.49E-11 |
| sp|Q9BYJ9|YTHD1_HUMAN | 60988.89737 | 1.16 | 0.232 | 0.003199 |
| sp|Q9H1I8|ASCC2_HUMAN | 87029.54234 | 0.45 | 0.067 | 2.20E-16 |
| sp|P62318|SMD3_HUMAN | 14003.33655 | 1.2 | 0.089 | 5.83E-12 |
| sp|Q9Y4C2|TCAF1_HUMAN | 103125.9348 | 0.72 | 0.088 | 9.67E-13 |
| sp|Q9UKU6|TRHDE_HUMAN | 117421.0156 | 0.53 | 0.339 | 1.17E-06 |
| sp|Q9UNQ2|DIM1_HUMAN | 35481.18563 | 0.66 | 0.125 | 1.82E-11 |
| sp|Q7L5Y1|ENOF1_HUMAN | 50363.57354 | 0.61 | 0.188 | 2.15E-09 |
| sp|Q06141|REG3A_HUMAN | 19763.5027 | 0.5 | 0.243 | 4.26E-06 |
| sp|Q13185|CBX3_HUMAN | 20951.40315 | 1.57 | 0.455 | 1.40E-07 |
| sp|Q9BSJ8|ESYT1_HUMAN | 123275.1309 | 0.81 | 0.101 | 9.36E-09 |
| sp|P33552|CKS2_HUMAN | 9835.970014 | 1.15 | 0.103 | 1.51E-07 |
| sp|Q9P0S3|ORML1_HUMAN | 17342.16332 | 0.59 | 0.213 | 7.00E-09 |
| sp|Q9NX24|NHP2_HUMAN | 17514.03242 | 1.23 | 0.108 | 3.72E-11 |
| sp|Q96A73|P33MX_HUMAN | 33208.00127 | 1.11 | 0.106 | 2.00E-05 |
| sp|Q14457|BECN1_HUMAN | 52358.61848 | 0.57 | 0.095 | 2.61E-15 |
| sp|P22830|HEMH_HUMAN | 48383.76566 | 0.99 | 0.169 | 0.4495 |
| sp|Q8N543|OGFD1_HUMAN | 63644.2965 | 1.32 | 0.284 | 1.96E-06 |
| sp|Q5VYK3|ECM29_HUMAN | 205967.0661 | 0.66 | 0.095 | 1.61E-13 |
| sp|Q8TB61|S35B2_HUMAN | 48035.77811 | 0.7 | 0.19 | 2.21E-07 |
| sp|O43493|TGON2_HUMAN | 50970.3265 | 1.19 | 0.218 | 0.0002412 |
| sp|P50135|HNMT_HUMAN | 33597.7004 | 0.94 | 0.159 | 0.02971 |
| sp|P61106|RAB14_HUMAN | 24092.00296 | 0.97 | 0.16 | 0.2401 |
| sp|Q9BQE3|TBA1C_HUMAN | 50529.70936 | 0.93 | 0.117 | 0.003316 |
| sp|Q00013|EM55_HUMAN | 52473.72202 | 1.62 | 0.277 | 7.31E-13 |
| sp|O60568|PLOD3_HUMAN | 85283.67747 | 0.91 | 0.191 | 0.01246 |
| sp|O75822|EIF3J_HUMAN | 29140.57571 | 1.63 | 0.312 | 1.10E-11 |
| sp|P98160|PGBM_HUMAN | 479234.4991 | 0.88 | 0.168 | 0.000735 |
| sp|P36955|PEDF_HUMAN | 46436.35917 | 1.69 | 0.541 | 2.12E-07 |
| sp|Q06210|GFPT1_HUMAN | 79536.61144 | 0.55 | 0.123 | 3.24E-13 |
| sp|Q7L3B6|CD37L_HUMAN | 39304.9237 | 1.09 | 0.145 | 0.006303 |
| sp|Q6YHU6|THADA_HUMAN | 222868.3307 | 0.73 | 0.413 | 0.0001177 |
| sp|Q8N1Q1|CAH13_HUMAN | 29464.02688 | 1.21 | 0.659 | 0.8019 |
| sp|Q8IW70|T151B_HUMAN | 62703.13172 | 2.51 | 1.422 | 3.17E-07 |
| sp|Q8IWA4|MFN1_HUMAN | 84886.45156 | 0.49 | 0.117 | 3.40E-13 |
| sp|Q9UBW5|BIN2_HUMAN | 61989.87287 | 1.25 | 0.292 | 0.0002086 |
| sp|Q96EY5|MB12A_HUMAN | 29088.83624 | 1.06 | 0.218 | 0.3733 |
| sp|O95427|PIGN_HUMAN | 106293.6802 | 0.75 | 0.151 | 2.52E-07 |
| sp|Q6UXB3|LYPD2_HUMAN | 13772.71099 | 0.78 | 0.305 | 0.002501 |
| sp|Q8WYN0|ATG4A_HUMAN | 46014.69268 | 1.12 | 0.222 | 0.02863 |
| sp|P31153|METK2_HUMAN | 43957.4528 | 0.95 | 0.127 | 0.04974 |
| sp|Q8TDZ2|MICA1_HUMAN | 118866.337 | 0.88 | 0.166 | 0.000378 |
| sp|Q9HD45|TM9S3_HUMAN | 68566.34244 | 0.44 | 0.159 | 1.61E-11 |
| sp|P48436|SOX9_HUMAN | 56198.38868 | 0.9 | 0.364 | 0.03721 |
| sp|Q9NXU5|ARL15_HUMAN | 23242.64818 | 0.85 | 0.133 | 1.16E-05 |
| sp|Q9NUT2|ABCB8_HUMAN | 80775.16003 | 0.64 | 0.121 | 1.06E-11 |
| sp|Q9P2X3|IMPCT_HUMAN | 37005.41711 | 1.32 | 0.169 | 9.95E-11 |
| sp|Q15269|PWP2_HUMAN | 103338.6178 | 0.54 | 0.186 | 2.92E-09 |
| sp|Q9P253|VPS18_HUMAN | 111466.2808 | 0.72 | 0.056 | 2.20E-16 |
| sp|Q96KM6|Z512B_HUMAN | 98723.99997 | 0.97 | 0.121 | 0.1989 |
| sp|O60870|KIN17_HUMAN | 45726.54203 | 1.36 | 0.472 | 0.002207 |
| sp|P49419|AL7A1_HUMAN | 59002.32188 | 0.85 | 0.284 | 0.006472 |
| sp|O95573|ACSL3_HUMAN | 81319.65061 | 0.49 | 0.071 | 2.20E-16 |
| sp|P08590|MYL3_HUMAN | 22070.97888 | 0.83 | 0.207 | 0.0001817 |
| sp|Q8TEU7|RPGF6_HUMAN | 180831.6369 | 1 | 0.11 | 0.8115 |
| sp|Q9BZQ8|NIBAN_HUMAN | 104021.3562 | 0.77 | 0.155 | 6.88E-07 |
| sp|Q8WZ82|OVCA2_HUMAN | 24726.64594 | 0.66 | 0.117 | 5.93E-12 |
| sp|Q53FT3|HIKES_HUMAN | 21652.70321 | 0.74 | 0.198 | 1.76E-06 |
| sp|Q7Z7K6|CENPV_HUMAN | 30365.26947 | 0.9 | 0.169 | 0.002635 |
| sp|Q9NQ89|CL004_HUMAN | 64426.41832 | 0.97 | 0.236 | 0.2112 |
| sp|Q9NUQ9|FA49B_HUMAN | 36991.74465 | 0.88 | 0.175 | 0.001024 |
| sp|P35908|K22E_HUMAN | 65660.31397 | 1.41 | 0.298 | 8.12E-08 |
| sp|Q2M3C7|SPKAP_HUMAN | 189058.0109 | 1.24 | 0.265 | 0.000273 |
| sp|Q16644|MAPK3_HUMAN | 43454.68956 | 1.02 | 0.142 | 0.5994 |
| sp|Q16531|DDB1_HUMAN | 128123.7846 | 0.55 | 0.071 | 2.20E-16 |
| sp|Q96HU1|SGSM3_HUMAN | 86194.03528 | 1.05 | 0.386 | 0.7751 |
| sp|Q8IUR7|ARMC8_HUMAN | 76582.65103 | 1.13 | 0.327 | 0.182 |
| sp|Q9UK76|JUPI1_HUMAN | 15986.90071 | 1.52 | 0.612 | 0.0009853 |
| sp|Q92736|RYR2_HUMAN | 569320.1982 | 1.17 | 0.303 | 0.01983 |
| sp|P62906|RL10A_HUMAN | 24968.58891 | 0.72 | 0.144 | 4.95E-09 |
| sp|P26639|SYTC_HUMAN | 84276.19866 | 0.61 | 0.117 | 8.76E-13 |
| sp|Q15147|PLCB4_HUMAN | 135500.38 | 0.65 | 0.219 | 5.75E-08 |
| sp|P07099|HYEP_HUMAN | 53125.03921 | 0.66 | 0.12 | 1.52E-11 |
| sp|O94776|MTA2_HUMAN | 75699.0057 | 1.23 | 0.162 | 2.10E-07 |
| sp|P30740|ILEU_HUMAN | 42810.7348 | 1.26 | 0.393 | 0.006598 |
| sp|Q5W041|ARMC3_HUMAN | 97124.22435 | 1.03 | 0.231 | 0.9915 |
| sp|P61006|RAB8A_HUMAN | 23806.24246 | 0.54 | 0.103 | 6.22E-15 |
| sp|O60684|IMA7_HUMAN | 60714.72433 | 0.73 | 0.071 | 2.66E-14 |
| sp|Q15759|MK11_HUMAN | 41541.21832 | 1.28 | 0.262 | 5.48E-06 |
| sp|P29558|RBMS1_HUMAN | 44686.93027 | 1.39 | 0.283 | 1.49E-07 |
| sp|P15428|PGDH_HUMAN | 29168.924 | 0.69 | 0.294 | 0.0006958 |
| sp|Q8WXI4|ACO11_HUMAN | 69342.93566 | 0.74 | 0.16 | 1.85E-07 |
| sp|Q2M1P5|KIF7_HUMAN | 151389.2619 | 1.76 | 0.43 | 3.91E-11 |
| sp|Q8NEW0|ZNT7_HUMAN | 41923.44242 | 0.42 | 0.097 | 4.67E-16 |
| sp|Q9Y6H3|ATP23_HUMAN | 28671.92452 | 0.66 | 0.211 | 1.16E-07 |
| sp|Q8NHG7|SVIP_HUMAN | 8533.45371 | 0.48 | 0.163 | 3.77E-11 |
| sp|Q06330|SUH_HUMAN | 56324.80072 | 1.06 | 0.155 | 0.116 |
| sp|Q9UL46|PSME2_HUMAN | 27537.36949 | 0.69 | 0.207 | 7.67E-08 |
| sp|Q93034|CUL5_HUMAN | 91449.61054 | 0.67 | 0.094 | 1.48E-13 |
| sp|Q8NG68|TTL_HUMAN | 43566.20531 | 1.09 | 0.148 | 0.005213 |
| sp|O75935|DCTN3_HUMAN | 21202.28084 | 1.36 | 0.101 | 2.20E-16 |
| sp|Q9BWD3|RTL8A_HUMAN | 13161.69212 | 0.85 | 0.127 | 4.21E-06 |
| sp|P27144|KAD4_HUMAN | 25348.24301 | 1.12 | 0.264 | 0.07865 |
| sp|Q09428|ABCC8_HUMAN | 178457.6364 | 1.67 | 0.8 | 8.56E-05 |
| sp|Q9UPT8|ZC3H4_HUMAN | 140778.6966 | 1.54 | 0.337 | 8.26E-10 |
| sp|Q99828|CIB1_HUMAN | 21842.98227 | 0.88 | 0.162 | 0.0008431 |
| sp|Q9H4P4|RNF41_HUMAN | 36662.00263 | 2.12 | 0.71 | 9.23E-11 |
| sp|P22087|FBRL_HUMAN | 33859.45077 | 1.09 | 0.222 | 0.1199 |
| sp|Q9H4L5|OSBL3_HUMAN | 102111.4941 | 1.23 | 0.158 | 4.93E-08 |
| sp|Q9Y2R9|RT07_HUMAN | 28211.99705 | 1.23 | 0.264 | 0.0005069 |
| sp|Q8ND71|GIMA8_HUMAN | 75680.72193 | 1.02 | 0.124 | 0.7381 |
| sp|Q1KMD3|HNRL2_HUMAN | 85604.37272 | 1.24 | 0.11 | 1.52E-11 |
| sp|P09038|FGF2_HUMAN | 31075.07624 | 1.19 | 0.447 | 0.1061 |
| sp|Q99570|PI3R4_HUMAN | 154300.0797 | 0.73 | 0.094 | 5.56E-12 |
| sp|Q13887|KLF5_HUMAN | 51254.56493 | 1.07 | 0.253 | 0.3522 |
| sp|Q68EM7|RHG17_HUMAN | 95758.46215 | 1.41 | 0.176 | 1.74E-12 |
| sp|Q9H6R4|NOL6_HUMAN | 128350.1956 | 0.9 | 0.141 | 0.001097 |
| sp|Q15750|TAB1_HUMAN | 54877.47456 | 1.42 | 0.105 | 2.20E-16 |
| sp|Q7Z6J9|SEN54_HUMAN | 59278.02247 | 1 | 0.284 | 0.4933 |
| sp|Q8WUF8|F172A_HUMAN | 48152.35071 | 1.25 | 0.256 | 3.70E-05 |
| sp|O76031|CLPX_HUMAN | 69903.99814 | 1.17 | 0.203 | 0.0005491 |
| sp|O00461|GOLI4_HUMAN | 81870.27589 | 0.87 | 0.105 | 1.86E-06 |
| sp|P30566|PUR8_HUMAN | 55577.23825 | 0.62 | 0.093 | 1.63E-14 |
| sp|P08754|GNAI3_HUMAN | 41058.4717 | 0.5 | 0.087 | 2.20E-16 |
| sp|Q9NY15|STAB1_HUMAN | 286914.4115 | 0.99 | 0.234 | 0.4542 |
| sp|Q8NBN3|TM87A_HUMAN | 63770.06181 | 0.77 | 0.089 | 2.10E-11 |
| sp|Q5T5X7|BEND3_HUMAN | 95537.8249 | 0.93 | 0.147 | 0.01372 |
| sp|O95716|RAB3D_HUMAN | 24461.88605 | 0.33 | 0.116 | 5.05E-13 |
| sp|Q00978|IRF9_HUMAN | 44106.96606 | 1.38 | 0.298 | 6.38E-07 |
| sp|Q8TF65|GIPC2_HUMAN | 34486.00623 | 1.19 | 0.404 | 0.1062 |
| sp|Q13740|CD166_HUMAN | 65727.3472 | 1.31 | 0.547 | 0.08287 |
| sp|Q9NZN4|EHD2_HUMAN | 61275.76034 | 0.95 | 0.259 | 0.1356 |
| sp|Q5JTV8|TOIP1_HUMAN | 66361.31722 | 1.11 | 0.133 | 0.0004572 |
| sp|Q9NR19|ACSA_HUMAN | 79594.52829 | 0.63 | 0.109 | 3.49E-13 |
| sp|Q9UHQ4|BAP29_HUMAN | 28398.2076 | 0.64 | 0.088 | 6.74E-15 |
| sp|Q96DT5|DYH11_HUMAN | 524182.0598 | 1.04 | 0.099 | 0.1109 |
| sp|Q96IK1|BOD1_HUMAN | 19223.7063 | 1.38 | 0.233 | 2.46E-09 |
| sp|Q71RG4|TMUB2_HUMAN | 33920.28109 | 0.67 | 0.174 | 1.06E-08 |
| sp|Q9UJV9|DDX41_HUMAN | 70459.24252 | 0.9 | 0.125 | 0.0003022 |
| sp|Q9P219|DAPLE_HUMAN | 229213.3899 | 0.88 | 0.06 | 8.82E-10 |
| sp|P15291|B4GT1_HUMAN | 44273.47393 | 0.6 | 0.125 | 6.73E-12 |
| sp|Q7Z5N4|SDK1_HUMAN | 242970.2991 | 0.81 | 0.124 | 3.57E-07 |
| sp|Q8IWD4|CC117_HUMAN | 30846.20643 | 1.34 | 0.198 | 2.61E-09 |
| sp|Q96R72|OR4K3_HUMAN | 35879.64505 | 1.59 | 0.288 | 4.00E-11 |
| sp|Q8N668|COMD1_HUMAN | 21203.91552 | 1.74 | 0.17 | 2.20E-16 |
| sp|Q15166|PON3_HUMAN | 39792.40397 | 1.1 | 0.437 | 0.7795 |
| sp|P03915|NU5M_HUMAN | 67306.55335 | 0.43 | 0.185 | 5.62E-11 |
| sp|Q9Y2D4|EXC6B_HUMAN | 94864.4096 | 0.84 | 0.183 | 8.71E-05 |
| sp|Q12864|CAD17_HUMAN | 92543.06527 | 0.79 | 0.354 | 0.0008097 |
| sp|Q7Z4S6|KI21A_HUMAN | 188356.3175 | 1.21 | 0.245 | 0.0002167 |
| sp|Q9H9Q4|NHEJ1_HUMAN | 33697.08606 | 0.54 | 0.14 | 3.65E-12 |
| sp|P51114|FXR1_HUMAN | 70002.06197 | 1.28 | 0.168 | 2.18E-09 |
| sp|Q96DF8|ESS2_HUMAN | 52574.80582 | 1.33 | 0.215 | 7.36E-09 |
| sp|Q8IV63|VRK3_HUMAN | 53570.93176 | 0.64 | 0.156 | 9.29E-10 |
| sp|Q9ULJ6|ZMIZ1_HUMAN | 116417.2562 | 0.95 | 0.233 | 0.1143 |
| sp|P54792|DVLP1_HUMAN | 73646.20646 | 1.23 | 0.192 | 4.55E-06 |
| sp|P01009|A1AT_HUMAN | 46860.0733 | 1.42 | 0.362 | 3.95E-07 |
| sp|Q567U6|CCD93_HUMAN | 73418.99731 | 1.13 | 0.166 | 0.000477 |
| sp|P42766|RL35_HUMAN | 14524.54969 | 1.6 | 0.413 | 8.37E-09 |
| sp|P20933|ASPG_HUMAN | 37793.53462 | 1.01 | 0.371 | 0.4908 |
| sp|Q7Z422|SZRD1_HUMAN | 16968.89789 | 1.39 | 0.177 | 5.83E-12 |
| sp|Q92597|NDRG1_HUMAN | 43245.88489 | 0.86 | 0.206 | 0.001519 |
| sp|Q9H1D9|RPC6_HUMAN | 36042.26852 | 1.3 | 0.177 | 1.71E-09 |
| sp|A2A3L6|TTC24_HUMAN | 64023.40901 | 0.65 | 0.151 | 3.10E-09 |
| sp|P14410|SUIS_HUMAN | 210727.4881 | 0.77 | 0.28 | 0.0001427 |
| sp|P00352|AL1A1_HUMAN | 55436.17339 | 0.95 | 0.477 | 0.1132 |
| sp|Q96AB6|NTAN1_HUMAN | 34922.71167 | 1.65 | 0.203 | 2.20E-16 |
| sp|O60318|GANP_HUMAN | 220644.1972 | 0.62 | 0.099 | 1.17E-13 |
| sp|P07900|HS90A_HUMAN | 84987.82106 | 0.89 | 0.16 | 0.0009193 |
| sp|Q6PIY7|GLD2_HUMAN | 56487.58392 | 0.82 | 0.073 | 3.62E-11 |
| sp|P51003|PAPOA_HUMAN | 83229.17739 | 0.71 | 0.049 | 2.20E-16 |
| sp|P20908|CO5A1_HUMAN | 184113.3047 | 1.69 | 0.467 | 6.33E-09 |
| sp|P16591|FER_HUMAN | 95186.97739 | 1.04 | 0.113 | 0.158 |
| sp|Q8WWZ4|ABCAA_HUMAN | 177425.4189 | 2.32 | 2.469 | 0.02177 |
| sp|Q6YHK3|CD109_HUMAN | 162481.6312 | 0.92 | 0.236 | 0.04162 |
| sp|P53801|PTTG_HUMAN | 21090.52884 | 0.73 | 0.109 | 3.03E-11 |
| sp|Q9NQ50|RM40_HUMAN | 24457.17808 | 1.25 | 0.214 | 3.42E-06 |
| sp|Q9NVH1|DJC11_HUMAN | 63505.83094 | 0.69 | 0.137 | 1.57E-09 |
| sp|Q9UBG0|MRC2_HUMAN | 169743.1739 | 1.37 | 0.368 | 1.33E-05 |
| sp|P07910|HNRPC_HUMAN | 33688.54982 | 1.59 | 0.242 | 3.52E-13 |
| sp|O14920|IKKB_HUMAN | 87517.23016 | 0.75 | 0.084 | 1.17E-12 |
| sp|P02776|PLF4_HUMAN | 11104.99113 | 1.43 | 0.384 | 6.24E-06 |
| sp|P54577|SYYC_HUMAN | 59430.21702 | 0.88 | 0.172 | 0.0008646 |
| sp|O43447|PPIH_HUMAN | 19462.71652 | 1.13 | 0.2 | 0.004518 |
| sp|O94788|AL1A2_HUMAN | 57126.0368 | 1.28 | 0.359 | 0.003608 |
| sp|Q15022|SUZ12_HUMAN | 83725.84576 | 0.81 | 0.169 | 1.17E-05 |
| sp|Q96N66|MBOA7_HUMAN | 53396.51099 | 0.43 | 0.07 | 2.20E-16 |
| sp|Q96GA3|LTV1_HUMAN | 55031.29524 | 1.15 | 0.135 | 3.31E-06 |
| sp|Q9Y696|CLIC4_HUMAN | 28963.81973 | 0.79 | 0.134 | 2.32E-07 |
| sp|O15347|HMGB3_HUMAN | 23118.49562 | 1.24 | 0.52 | 0.08581 |
| sp|Q9NWM8|FKB14_HUMAN | 24252.47084 | 1.51 | 0.542 | 4.82E-05 |
| sp|B0I1T2|MYO1G_HUMAN | 117377.3116 | 0.87 | 0.18 | 0.0006426 |
| sp|Q9HD33|RM47_HUMAN | 29584.9988 | 1.14 | 0.218 | 0.009847 |
| sp|Q5JRK9|GGEE3_HUMAN | 12015.67342 | 0.64 | 0.466 | 0.0003813 |
| sp|Q9NZJ9|NUDT4_HUMAN | 20503.31984 | 1.22 | 0.226 | 2.52E-05 |
| sp|P28472|GBRB3_HUMAN | 54404.9367 | 1.39 | 0.397 | 6.70E-06 |
| sp|Q96C11|FGGY_HUMAN | 60620.7078 | 0.74 | 0.167 | 1.08E-06 |
| sp|Q12965|MYO1E_HUMAN | 127534.1958 | 0.89 | 0.133 | 0.0002524 |
| sp|Q96GX9|MTNB_HUMAN | 27716.44584 | 1.09 | 0.208 | 0.04807 |
| sp|P40763|STAT3_HUMAN | 88791.66655 | 0.63 | 0.093 | 1.84E-14 |
| sp|Q96EY1|DNJA3_HUMAN | 53064.73821 | 0.93 | 0.141 | 0.008967 |
| sp|Q9NQG7|HPS4_HUMAN | 77878.81786 | 0.85 | 0.48 | 0.008415 |
| sp|Q6XQN6|PNCB_HUMAN | 58094.22032 | 0.54 | 0.142 | 6.55E-12 |
| sp|A7MCY6|TBKB1_HUMAN | 68554.15946 | 0.93 | 0.141 | 0.009269 |
| sp|P07237|PDIA1_HUMAN | 57461.81299 | 1.25 | 0.204 | 1.55E-06 |
| sp|P51553|IDH3G_HUMAN | 43148.23654 | 1.09 | 0.103 | 0.0002537 |
| sp|Q9BQ39|DDX50_HUMAN | 83066.25556 | 1.21 | 0.158 | 2.72E-07 |
| sp|P22670|RFX1_HUMAN | 105016.9776 | 0.6 | 0.069 | 2.20E-16 |
| sp|Q6UXY1|BI2L2_HUMAN | 59045.58385 | 1.01 | 0.215 | 0.7751 |
| sp|Q99550|MPP9_HUMAN | 133665.7401 | 0.84 | 0.17 | 0.0001058 |
| sp|Q5T200|ZC3HD_HUMAN | 197185.348 | 1.19 | 0.089 | 5.57E-11 |
| sp|P40199|CEAM6_HUMAN | 37438.78531 | 0.93 | 0.454 | 0.08597 |
| sp|Q9H1E3|NUCKS_HUMAN | 27262.02158 | 1.88 | 0.486 | 9.51E-12 |
| sp|Q9Y5Q8|TF3C5_HUMAN | 59971.26492 | 0.63 | 0.098 | 6.01E-14 |
| sp|O43159|RRP8_HUMAN | 51406.37418 | 0.86 | 0.101 | 2.10E-06 |
| sp|P19474|RO52_HUMAN | 55143.49879 | 1.09 | 0.214 | 0.07788 |
| sp|P49585|PCY1A_HUMAN | 42028.94844 | 1.34 | 0.086 | 2.20E-16 |
| sp|Q9Y333|LSM2_HUMAN | 10923.65847 | 1.07 | 0.139 | 0.03198 |
| sp|Q7Z698|SPRE2_HUMAN | 48706.75118 | 1.05 | 0.184 | 0.3523 |
| sp|Q9BT30|ALKB7_HUMAN | 24653.70062 | 1.1 | 0.122 | 0.0007022 |
| sp|Q86VX2|COMD7_HUMAN | 22678.65435 | 0.7 | 0.126 | 1.11E-10 |
| sp|Q9NZJ7|MTCH1_HUMAN | 41841.4076 | 0.52 | 0.116 | 7.56E-14 |
| sp|P22492|H1T_HUMAN | 21987.65654 | 0.8 | 0.275 | 0.003984 |
| sp|Q9C0C2|TB182_HUMAN | 182693.395 | 1.4 | 0.256 | 7.82E-09 |
| sp|Q8N4Q0|PTGR3_HUMAN | 40438.87368 | 1.38 | 0.319 | 8.72E-07 |
| sp|Q9Y314|NOSIP_HUMAN | 33646.18062 | 1.45 | 0.576 | 0.001318 |
| sp|O00471|EXOC5_HUMAN | 82809.13126 | 0.7 | 0.072 | 7.38E-15 |
| sp|Q9NYL4|FKB11_HUMAN | 22319.33758 | 0.9 | 0.192 | 0.005022 |
| sp|Q8IXI2|MIRO1_HUMAN | 71747.27543 | 0.63 | 0.075 | 5.90E-16 |
| sp|Q16798|MAON_HUMAN | 67634.82611 | 0.88 | 0.178 | 0.002568 |
| sp|P10144|GRAB_HUMAN | 28079.48719 | 1.14 | 0.438 | 0.2944 |
| sp|P23443|KS6B1_HUMAN | 59425.9334 | 1.1 | 0.157 | 0.003928 |
| sp|Q9Y485|DMXL1_HUMAN | 341427.1562 | 0.92 | 0.114 | 0.001333 |
| sp|Q9H3H9|TCAL2_HUMAN | 25929.71591 | 1.79 | 0.771 | 3.28E-05 |
| sp|Q9HD47|MOG1_HUMAN | 20588.42338 | 1.55 | 0.289 | 9.20E-11 |
| sp|Q14515|SPRL1_HUMAN | 75998.57323 | 1.51 | 0.556 | 1.65E-05 |
| sp|P43405|KSYK_HUMAN | 72515.46806 | 1.04 | 0.186 | 0.4735 |
| sp|Q99720|SGMR1_HUMAN | 25150.79289 | 0.95 | 0.08 | 0.004716 |
| sp|Q9H6U6|BCAS3_HUMAN | 102465.6017 | 1.36 | 0.137 | 8.93E-14 |
| sp|Q96EY8|MMAB_HUMAN | 27695.10986 | 0.84 | 0.258 | 0.001065 |
| sp|Q9NPI1|BRD7_HUMAN | 74530.35642 | 0.94 | 0.17 | 0.04661 |
| sp|Q96Q89|KI20B_HUMAN | 211850.1018 | 1.14 | 0.365 | 0.1651 |
| sp|Q15126|PMVK_HUMAN | 22134.27754 | 1.13 | 0.117 | 6.28E-06 |
| sp|O43424|GRID2_HUMAN | 114121.4199 | 1.32 | 0.135 | 1.49E-12 |
| sp|P11388|TOP2A_HUMAN | 174999.019 | 0.66 | 0.252 | 3.28E-06 |
| sp|Q9Y5X1|SNX9_HUMAN | 66930.75493 | 0.92 | 0.152 | 0.008619 |
| sp|Q9H832|UBE2Z_HUMAN | 38623.72263 | 1.15 | 0.155 | 0.0001392 |
| sp|Q9BYB0|SHAN3_HUMAN | 185106.7646 | 1.13 | 0.182 | 0.001906 |
| sp|P84243|H33_HUMAN | 15357.51373 | 1.57 | 0.438 | 1.79E-07 |
| sp|Q7Z6L1|TCPR1_HUMAN | 131420.6856 | 0.92 | 0.147 | 0.01091 |
| sp|O14907|TX1B3_HUMAN | 13708.11823 | 1.1 | 0.141 | 0.002789 |
| sp|O76094|SRP72_HUMAN | 75112.17536 | 1.35 | 0.135 | 2.02E-13 |
| sp|Q00722|PLCB2_HUMAN | 134890.2241 | 0.69 | 0.188 | 9.73E-08 |
| sp|Q9H6V9|LDAH_HUMAN | 37732.34286 | 0.61 | 0.092 | 1.12E-14 |
| sp|P48594|SPB4_HUMAN | 44978.5301 | 1.86 | 2.119 | 0.1784 |
| sp|O75976|CBPD_HUMAN | 153900.5491 | 0.67 | 0.135 | 1.96E-10 |
| sp|P49247|RPIA_HUMAN | 33515.0747 | 1.16 | 0.167 | 7.99E-05 |
| sp|Q92692|NECT2_HUMAN | 58144.0788 | 1.04 | 0.218 | 0.636 |
| sp|Q99551|MTEF1_HUMAN | 46187.18237 | 0.98 | 0.223 | 0.3351 |
| sp|P78381|S35A2_HUMAN | 41434.58007 | 0.43 | 0.123 | 4.84E-14 |
| sp|Q8NI08|NCOA7_HUMAN | 106704.4461 | 1.33 | 0.218 | 8.72E-09 |
| sp|P01850|TRBC1_HUMAN | 19967.02146 | 0.84 | 0.237 | 0.0005249 |
| sp|Q96IR7|HPDL_HUMAN | 39685.4721 | 0.68 | 0.092 | 1.35E-13 |
| sp|Q7KZN9|COX15_HUMAN | 46324.54094 | 0.61 | 0.153 | 2.21E-10 |
| sp|Q03405|UPAR_HUMAN | 38588.87068 | 2.22 | 2.264 | 0.0048 |
| sp|Q5VYS8|TUT7_HUMAN | 173270.3933 | 0.96 | 0.172 | 0.1545 |
| sp|P10253|LYAG_HUMAN | 106094.0981 | 1.33 | 0.203 | 2.41E-09 |
| sp|P31997|CEAM8_HUMAN | 38397.18654 | 0.77 | 0.485 | 0.001589 |
| sp|O95166|GBRAP_HUMAN | 13891.26941 | 1.2 | 0.273 | 0.001403 |
| sp|P23743|DGKA_HUMAN | 83927.16809 | 0.86 | 0.175 | 0.0002757 |
| sp|Q8IXT5|RB12B_HUMAN | 118353.9956 | 0.99 | 0.106 | 0.4301 |
| sp|Q8IWV7|UBR1_HUMAN | 203426.7064 | 0.9 | 0.12 | 0.0002189 |
| sp|O43353|RIPK2_HUMAN | 61708.3764 | 1.51 | 0.516 | 2.00E-06 |
| sp|Q9HCP0|KC1G1_HUMAN | 48918.76959 | 0.77 | 0.198 | 8.21E-06 |
| sp|P54578|UBP14_HUMAN | 56471.1863 | 1.02 | 0.144 | 0.7164 |
| sp|Q9H3G5|CPVL_HUMAN | 54396.19881 | 1.02 | 0.256 | 0.8155 |
| sp|P13489|RINI_HUMAN | 51747.76351 | 0.92 | 0.18 | 0.01738 |
| sp|Q8WTS1|ABHD5_HUMAN | 39508.85744 | 1.75 | 0.494 | 2.39E-09 |
| sp|Q02952|AKA12_HUMAN | 191918.8731 | 2.55 | 0.608 | 1.55E-14 |
| sp|Q7Z6J0|SH3R1_HUMAN | 94192.98967 | 1.14 | 0.142 | 2.82E-05 |
| sp|P60510|PP4C_HUMAN | 35609.63583 | 0.91 | 0.246 | 0.03509 |
| sp|Q15370|ELOB_HUMAN | 13220.58356 | 1.53 | 0.18 | 2.98E-15 |
| sp|Q53HC0|CCD92_HUMAN | 37034.33962 | 1.1 | 0.152 | 0.006721 |
| sp|Q8N5C6|SRBD1_HUMAN | 112941.8245 | 0.86 | 0.12 | 1.26E-05 |
| sp|O95400|CD2B2_HUMAN | 37719.26644 | 1.31 | 0.151 | 2.42E-11 |
| sp|Q9UBX7|KLK11_HUMAN | 31762.04826 | 0.88 | 0.21 | 0.002602 |
| sp|Q8WUA8|TSK_HUMAN | 38107.90913 | 1.06 | 0.11 | 0.01577 |
| sp|P18583|SON_HUMAN | 264044.7 | 1.32 | 0.124 | 3.60E-13 |
| sp|Q96C19|EFHD2_HUMAN | 26776.49382 | 1.17 | 0.231 | 0.005126 |
| sp|Q13642|FHL1_HUMAN | 37988.30189 | 0.67 | 0.321 | 1.05E-05 |
| sp|Q93052|LPP_HUMAN | 67111.35906 | 0.76 | 0.221 | 4.67E-06 |
| sp|Q9NQH7|XPP3_HUMAN | 57606.06561 | 0.61 | 0.12 | 1.26E-12 |
| sp|O94763|RMP_HUMAN | 60233.73284 | 0.83 | 0.201 | 0.0002169 |
| sp|Q9NV35|NUD15_HUMAN | 18807.30114 | 1.2 | 0.382 | 0.06709 |
| sp|Q9BXJ0|C1QT5_HUMAN | 25435.68693 | 0.88 | 0.185 | 0.002695 |
| sp|O96005|CLPT1_HUMAN | 76258.54009 | 0.51 | 0.153 | 5.25E-10 |
| sp|P12259|FA5_HUMAN | 252667.9349 | 0.93 | 0.393 | 0.07516 |
| sp|P20711|DDC_HUMAN | 54557.56709 | 0.83 | 0.41 | 0.009279 |
| sp|P26010|ITB7_HUMAN | 89964.99075 | 1.07 | 0.165 | 0.08508 |
| sp|Q5PSV4|BRM1L_HUMAN | 37986.60104 | 1.19 | 0.173 | 1.04E-05 |
| sp|Q9Y3B7|RM11_HUMAN | 20709.45757 | 0.97 | 0.213 | 0.2652 |
| sp|P08473|NEP_HUMAN | 86126.11846 | 0.62 | 0.256 | 2.28E-06 |
| sp|Q6ZUJ8|BCAP_HUMAN | 91121.25999 | 0.91 | 0.212 | 0.01908 |
| sp|A6NCE7|MP3B2_HUMAN | 14657.6722 | 1.39 | 0.295 | 2.03E-07 |
| sp|O43795|MYO1B_HUMAN | 132910.3072 | 0.79 | 0.145 | 3.72E-07 |
| sp|Q15070|OXA1L_HUMAN | 48897.63792 | 0.67 | 0.116 | 3.49E-11 |
| sp|Q6PI48|SYDM_HUMAN | 74068.30274 | 0.74 | 0.159 | 8.66E-08 |
| sp|Q9BSH5|HDHD3_HUMAN | 28192.49199 | 1.01 | 0.197 | 0.9145 |
| sp|Q15075|EEA1_HUMAN | 163318.5351 | 1.53 | 0.319 | 1.34E-10 |
| sp|Q6ZNA5|FRRS1_HUMAN | 66737.64316 | 1.78 | 0.632 | 6.78E-08 |
| sp|P08237|PFKAM_HUMAN | 85965.70007 | 0.61 | 0.11 | 7.73E-13 |
| sp|P35900|K1C20_HUMAN | 48495.93081 | 1.1 | 0.867 | 0.3502 |
| sp|O75208|COQ9_HUMAN | 35639.66048 | 1.1 | 0.199 | 0.02988 |
| sp|Q96G21|IMP4_HUMAN | 33831.57056 | 0.82 | 0.08 | 1.90E-10 |
| sp|Q9UMX5|NENF_HUMAN | 18826.78277 | 1.51 | 0.363 | 8.36E-08 |
| sp|Q14157|UBP2L_HUMAN | 114561.3029 | 1.79 | 0.637 | 1.50E-08 |
| sp|P30711|GSTT1_HUMAN | 27470.612 | 0.85 | 0.38 | 0.01868 |
| sp|Q96CN9|GCC1_HUMAN | 87967.38381 | 1.09 | 0.107 | 0.0003242 |
| sp|Q96PU5|NED4L_HUMAN | 112185.9341 | 0.79 | 0.137 | 9.73E-08 |
| sp|Q9BUL9|RPP25_HUMAN | 20772.62189 | 1.17 | 0.324 | 0.07677 |
| sp|Q12797|ASPH_HUMAN | 86247.60816 | 1.14 | 0.194 | 0.002931 |
| sp|Q00059|TFAM_HUMAN | 29288.24209 | 1.26 | 0.218 | 1.73E-06 |
| sp|Q9UBF8|PI4KB_HUMAN | 92044.70429 | 0.9 | 0.052 | 3.06E-09 |
| sp|Q8N9M5|TM102_HUMAN | 54749.96448 | 0.87 | 0.295 | 0.007873 |
| sp|Q8TCF1|ZFAN1_HUMAN | 31661.49668 | 1.12 | 0.141 | 0.0004376 |
| sp|P17050|NAGAB_HUMAN | 47029.0917 | 0.74 | 0.179 | 1.10E-06 |
| sp|P00738|HPT_HUMAN | 45842.81152 | 1.47 | 0.535 | 0.0002306 |
| sp|Q9Y580|RBM7_HUMAN | 30466.73264 | 1.31 | 0.318 | 3.72E-05 |
| sp|Q9NRG1|PRDC1_HUMAN | 25810.02447 | 1.21 | 0.228 | 0.0002017 |
| sp|Q9Y5P4|C43BP_HUMAN | 71456.91046 | 1.18 | 0.102 | 1.33E-09 |
| sp|Q15485|FCN2_HUMAN | 34417.68338 | 1.27 | 0.738 | 0.8061 |
| sp|Q13823|NOG2_HUMAN | 83813.47521 | 1.37 | 0.205 | 4.15E-10 |
| sp|Q08426|ECHP_HUMAN | 80053.9614 | 0.74 | 0.169 | 1.84E-07 |
| sp|O76070|SYUG_HUMAN | 13304.76361 | 2.01 | 1.056 | 0.0001036 |
| sp|Q9UI12|VATH_HUMAN | 56399.26911 | 0.67 | 0.12 | 2.91E-11 |
| sp|P05141|ADT2_HUMAN | 33041.22626 | 0.45 | 0.125 | 4.68E-14 |
| sp|Q14651|PLSI_HUMAN | 70590.39913 | 0.77 | 0.264 | 0.0001173 |
| sp|P60981|DEST_HUMAN | 18931.63724 | 0.88 | 0.251 | 0.00736 |
| sp|P63098|CANB1_HUMAN | 19383.6079 | 1.44 | 0.25 | 2.37E-10 |
| sp|Q9Y312|AAR2_HUMAN | 43825.19871 | 1.21 | 0.265 | 0.001723 |
| sp|Q9UGR2|Z3H7B_HUMAN | 111651.296 | 1 | 0.07 | 0.948 |
| sp|Q9P299|COPZ2_HUMAN | 23571.97082 | 1.02 | 0.206 | 0.972 |
| sp|P63208|SKP1_HUMAN | 18799.29861 | 1.28 | 0.199 | 8.75E-08 |
| sp|Q15291|RBBP5_HUMAN | 59781.91343 | 0.96 | 0.21 | 0.1641 |
| sp|O96007|MOC2B_HUMAN | 21140.80169 | 0.81 | 0.142 | 3.54E-07 |
| sp|Q99102|MUC4_HUMAN | 233978.4107 | 1.79 | 0.574 | 1.97E-09 |
| sp|Q8IYQ7|THNS1_HUMAN | 84082.4604 | 0.71 | 0.151 | 8.90E-09 |
| sp|P20645|MPRD_HUMAN | 31468.55935 | 0.79 | 0.145 | 3.33E-07 |
| sp|Q0VDG4|SCRN3_HUMAN | 48951.37814 | 1.07 | 0.151 | 0.05592 |
| sp|Q9BQ48|RM34_HUMAN | 10140.57826 | 1.44 | 0.305 | 4.58E-09 |
| sp|P15090|FABP4_HUMAN | 14805.52948 | 4.1 | 2.121 | 9.55E-12 |
| sp|Q9BVV7|TIM21_HUMAN | 28508.91064 | 0.83 | 0.196 | 0.0002959 |
| sp|P35443|TSP4_HUMAN | 108463.9945 | 0.6 | 0.206 | 3.47E-08 |
| sp|Q9Y5F3|PCDB1_HUMAN | 90929.2889 | 0.88 | 0.107 | 1.29E-05 |
| sp|P82909|RT36_HUMAN | 11440.95372 | 1.18 | 0.228 | 0.0008283 |
| sp|O43292|GPAA1_HUMAN | 67904.08304 | 0.57 | 0.104 | 1.45E-13 |
| sp|Q14247|SRC8_HUMAN | 61701.58554 | 1.54 | 0.612 | 1.92E-05 |
| sp|P60604|UB2G2_HUMAN | 18707.11837 | 0.88 | 0.158 | 0.0006606 |
| sp|Q16819|MEP1A_HUMAN | 85430.78603 | 0.72 | 0.259 | 3.27E-05 |
| sp|O15511|ARPC5_HUMAN | 16349.31013 | 1.24 | 0.193 | 6.34E-07 |
| sp|Q9NUL7|DDX28_HUMAN | 59754.61199 | 0.75 | 0.284 | 0.0001381 |
| sp|Q9UJZ1|STML2_HUMAN | 38606.2308 | 1.16 | 0.252 | 0.004761 |
| sp|Q8IWZ3|ANKH1_HUMAN | 271268.3352 | 1.05 | 0.145 | 0.1481 |
| sp|P49961|ENTP1_HUMAN | 58536.32861 | 0.58 | 0.11 | 1.58E-13 |
| sp|O43148|MCES_HUMAN | 55475.50939 | 1.27 | 0.276 | 0.0001053 |
| sp|Q9UIF9|BAZ2A_HUMAN | 212985.7897 | 0.98 | 0.102 | 0.2635 |
| sp|P17931|LEG3_HUMAN | 26175.06414 | 1.24 | 0.279 | 0.000502 |
| sp|Q86UP3|ZFHX4_HUMAN | 398139.3651 | 0.9 | 0.24 | 0.01965 |
| sp|Q92610|ZN592_HUMAN | 140102.8748 | 1.3 | 0.299 | 4.24E-05 |
| sp|Q15773|MLF2_HUMAN | 28168.2153 | 1.19 | 0.228 | 0.0008635 |
| sp|P05455|LA_HUMAN | 46961.21463 | 1.39 | 0.259 | 6.21E-09 |
| sp|O14960|LECT2_HUMAN | 16703.48776 | 1.14 | 0.296 | 0.07719 |
| sp|Q96T60|PNKP_HUMAN | 57535.6802 | 1.1 | 0.152 | 0.006059 |
| sp|P45984|MK09_HUMAN | 48660.30792 | 0.87 | 0.132 | 6.75E-05 |
| sp|Q15418|KS6A1_HUMAN | 83051.91822 | 0.93 | 0.193 | 0.04161 |
| sp|Q92918|M4K1_HUMAN | 92246.58788 | 1.08 | 0.352 | 0.5357 |
| sp|P28799|GRN_HUMAN | 68499.71553 | 1.26 | 0.263 | 2.10E-05 |
| sp|Q5SQS7|SH24B_HUMAN | 51296.29282 | 0.51 | 0.075 | 2.20E-16 |
| sp|Q9NYQ7|CELR3_HUMAN | 362846.9806 | 2.92 | 1.228 | 5.38E-12 |
| sp|O95219|SNX4_HUMAN | 52143.19654 | 1.01 | 0.111 | 0.9163 |
| sp|Q9ULV3|CIZ1_HUMAN | 101048.2124 | 1.42 | 0.312 | 4.43E-08 |
| sp|Q8IZT6|ASPM_HUMAN | 413171.484 | 1.29 | 0.646 | 0.1446 |
| sp|Q9Y2R0|COA3_HUMAN | 11706.11459 | 0.87 | 0.124 | 2.78E-05 |
| sp|P29762|RABP1_HUMAN | 15708.80179 | 0.65 | 0.288 | 4.59E-05 |
| sp|Q8NCG7|DGLB_HUMAN | 74863.9082 | 1.16 | 0.194 | 0.0004216 |
| sp|Q8N357|S35F6_HUMAN | 40455.24871 | 0.74 | 0.167 | 6.43E-07 |
| sp|Q49A26|GLYR1_HUMAN | 61061.16697 | 0.71 | 0.084 | 1.14E-13 |
| sp|A2RU48|SMCO3_HUMAN | 25014.497 | 1.06 | 0.135 | 0.08402 |
| sp|Q13315|ATM_HUMAN | 355518.9707 | 0.72 | 0.099 | 1.43E-11 |
| sp|Q9NRW4|DUS22_HUMAN | 21163.75566 | 0.89 | 0.158 | 0.001135 |
| sp|P69905|HBA_HUMAN | 15286.93649 | 1.33 | 0.407 | 0.0004988 |
| sp|Q01664|TFAP4_HUMAN | 38797.82704 | 1.8 | 0.725 | 1.66E-06 |
| sp|P34949|MPI_HUMAN | 47178.01896 | 1.08 | 0.197 | 0.1235 |
| sp|Q9NXW2|DJB12_HUMAN | 42043.91067 | 1.33 | 0.291 | 3.56E-06 |
| sp|Q9NYK5|RM39_HUMAN | 39181.83121 | 0.8 | 0.135 | 5.73E-07 |
| sp|Q6P1M0|S27A4_HUMAN | 72912.47077 | 0.84 | 0.096 | 3.10E-08 |
| sp|Q8NB16|MLKL_HUMAN | 54996.73841 | 0.76 | 0.212 | 4.19E-06 |
| sp|P43378|PTN9_HUMAN | 68529.04371 | 0.9 | 0.131 | 0.0008771 |
| sp|O43663|PRC1_HUMAN | 72227.85608 | 0.86 | 0.166 | 0.0002479 |
| sp|Q9NP61|ARFG3_HUMAN | 57388.14882 | 1.09 | 0.136 | 0.005864 |
| sp|Q9UL63|MKLN1_HUMAN | 85892.93267 | 1 | 0.141 | 0.6804 |
| sp|Q9Y5S9|RBM8A_HUMAN | 19915.7416 | 1.29 | 0.158 | 2.81E-10 |
| sp|Q9Y295|DRG1_HUMAN | 40783.92023 | 0.64 | 0.142 | 4.18E-10 |
| sp|P32456|GBP2_HUMAN | 67661.57529 | 0.94 | 0.395 | 0.08764 |
| sp|P24043|LAMA2_HUMAN | 352960.1356 | 0.65 | 0.136 | 7.00E-10 |
| sp|Q96DU7|IP3KC_HUMAN | 75883.64124 | 1.29 | 0.224 | 1.69E-07 |
| sp|Q6IQ16|SPOPL_HUMAN | 45284.44929 | 0.82 | 0.204 | 9.45E-05 |
| sp|Q9H3P2|NELFA_HUMAN | 57450.85625 | 1.1 | 0.066 | 8.64E-08 |
| sp|Q9H0A8|COMD4_HUMAN | 22074.42958 | 1.34 | 0.232 | 3.32E-08 |
| sp|Q6ZWJ1|STXB4_HUMAN | 62118.58872 | 1.24 | 0.141 | 8.39E-10 |
| sp|Q13888|TF2H2_HUMAN | 45455.46292 | 0.8 | 0.174 | 1.01E-05 |
| sp|P36404|ARL2_HUMAN | 21017.90716 | 1.04 | 0.157 | 0.4149 |
| sp|Q14444|CAPR1_HUMAN | 78471.07821 | 1.41 | 0.296 | 6.53E-08 |
| sp|P49189|AL9A1_HUMAN | 54661.31472 | 0.77 | 0.156 | 3.68E-07 |
| sp|Q6DD87|ZN787_HUMAN | 41411.00651 | 1.14 | 0.185 | 0.001779 |
| sp|Q9UNM6|PSD13_HUMAN | 43185.2438 | 0.64 | 0.071 | 2.20E-16 |
| sp|Q96H79|ZCCHL_HUMAN | 33948.85027 | 1.48 | 0.7 | 0.0008643 |
| sp|P25705|ATPA_HUMAN | 59809.6259 | 1.07 | 0.124 | 0.01098 |
| sp|Q9UM54|MYO6_HUMAN | 150946.9978 | 0.64 | 0.213 | 8.60E-08 |
| sp|Q96GS4|BORC6_HUMAN | 37584.74919 | 0.98 | 0.117 | 0.2551 |
| sp|Q9H098|F107B_HUMAN | 15530.23982 | 1.22 | 0.432 | 0.05067 |
| sp|Q8TBX8|PI42C_HUMAN | 47423.16036 | 0.94 | 0.107 | 0.007804 |
| sp|Q96RL1|UIMC1_HUMAN | 80857.36687 | 1.68 | 0.279 | 8.97E-14 |
| sp|Q9NZ32|ARP10_HUMAN | 46829.50051 | 0.86 | 0.114 | 2.39E-06 |
| sp|Q9BUH8|BEGIN_HUMAN | 65371.99919 | 1.53 | 1.047 | 0.03733 |
| sp|Q8NC96|NECP1_HUMAN | 29815.16192 | 0.98 | 0.239 | 0.3542 |
| sp|Q8NHH9|ATLA2_HUMAN | 66796.34954 | 0.58 | 0.115 | 1.22E-13 |
| sp|O00410|IPO5_HUMAN | 125014.4068 | 0.57 | 0.066 | 2.20E-16 |
| sp|O75635|SPB7_HUMAN | 43144.59143 | 1.56 | 0.817 | 0.003002 |
| sp|P00751|CFAB_HUMAN | 86829.02902 | 1.05 | 0.311 | 0.9832 |
| sp|P34910|EVI2B_HUMAN | 48960.28311 | 0.98 | 0.314 | 0.2698 |
| sp|P47895|AL1A3_HUMAN | 56853.11057 | 1.49 | 0.389 | 2.84E-07 |
| sp|Q5TA45|INT11_HUMAN | 68341.89226 | 0.88 | 0.186 | 0.001322 |
| sp|A6NC98|CC88B_HUMAN | 165148.07 | 1.16 | 0.22 | 0.001408 |
| sp|O60784|TOM1_HUMAN | 54051.9624 | 1.3 | 0.147 | 2.68E-11 |
| sp|O43294|TGFI1_HUMAN | 51303.3492 | 0.71 | 0.174 | 1.74E-07 |
| sp|Q9P0J0|NDUAD_HUMAN | 16669.58872 | 0.9 | 0.172 | 0.003562 |
| sp|P29466|CASP1_HUMAN | 45795.71203 | 1.34 | 0.392 | 7.16E-05 |
| sp|Q15052|ARHG6_HUMAN | 88679.704 | 1.18 | 0.283 | 0.007373 |
| sp|Q6EMK4|VASN_HUMAN | 72732.91426 | 1.51 | 0.475 | 1.09E-06 |
| sp|P00488|F13A_HUMAN | 83709.78722 | 0.96 | 0.347 | 0.1585 |
| sp|P10809|CH60_HUMAN | 61169.43561 | 1.13 | 0.225 | 0.01385 |
| sp|Q8NFP7|NUD10_HUMAN | 18698.28865 | 2.81 | 1.489 | 4.62E-07 |
| sp|Q8IYR0|CF206_HUMAN | 71642.8482 | 0.86 | 0.163 | 0.0002389 |
| sp|P05362|ICAM1_HUMAN | 58569.24163 | 1.36 | 0.288 | 6.80E-07 |
| sp|Q9UHJ6|SHPK_HUMAN | 51896.19515 | 0.92 | 0.107 | 0.0006464 |
| sp|O94985|CSTN1_HUMAN | 110960.1941 | 1.05 | 0.199 | 0.393 |
| sp|P05496|AT5G1_HUMAN | 14420.51477 | 0.18 | 0.088 | 2.20E-16 |
| sp|P07947|YES_HUMAN | 61257.68842 | 0.87 | 0.138 | 5.18E-05 |
| sp|Q8IZ83|A16A1_HUMAN | 86082.07708 | 0.76 | 0.09 | 1.85E-11 |
| sp|P55735|SEC13_HUMAN | 36013.29698 | 1.3 | 0.221 | 1.72E-07 |
| sp|P53041|PPP5_HUMAN | 57394.4524 | 0.54 | 0.102 | 3.89E-14 |
| sp|P28072|PSB6_HUMAN | 25551.52316 | 0.93 | 0.141 | 0.01004 |
| sp|P82650|RT22_HUMAN | 41407.44998 | 1.3 | 0.269 | 1.21E-05 |
| sp|Q2M2H8|MGAL_HUMAN | 279052.2409 | 0.72 | 0.185 | 1.02E-06 |
| sp|P51571|SSRD_HUMAN | 19139.65988 | 0.6 | 0.058 | 2.20E-16 |
| sp|P57678|GEMI4_HUMAN | 121709.581 | 0.71 | 0.108 | 2.40E-11 |
| sp|Q9NVN8|GNL3L_HUMAN | 66197.87066 | 0.97 | 0.173 | 0.2339 |
| sp|Q9UKX5|ITA11_HUMAN | 134508.8063 | 1.19 | 0.264 | 0.002602 |
| sp|A1L4H1|SRCRL_HUMAN | 168072.6328 | 1.27 | 0.496 | 0.00936 |
| sp|Q6ICB4|SESQ2_HUMAN | 28758.27733 | 0.97 | 0.124 | 0.1943 |
| sp|Q5QJ74|TBCEL_HUMAN | 48659.61371 | 1.54 | 0.247 | 3.26E-12 |
| sp|Q6AZY7|SCAR3_HUMAN | 65591.12693 | 0.84 | 0.38 | 0.00653 |
| sp|Q969Y2|GTPB3_HUMAN | 52521.37996 | 0.98 | 0.249 | 0.3476 |
| sp|P49662|CASP4_HUMAN | 43786.71109 | 1.24 | 0.333 | 0.0005703 |
| sp|Q8WV92|MITD1_HUMAN | 29620.06827 | 0.63 | 0.082 | 1.35E-15 |
| sp|Q4KMQ1|TPRN_HUMAN | 75776.77034 | 0.99 | 0.326 | 0.3569 |
| sp|P62877|RBX1_HUMAN | 12703.89917 | 0.97 | 0.151 | 0.1972 |
| sp|P52209|6PGD_HUMAN | 53601.13586 | 0.59 | 0.095 | 1.14E-14 |
| sp|Q9Y3M8|STA13_HUMAN | 126182.1936 | 0.94 | 0.133 | 0.01921 |
| sp|Q13243|SRSF5_HUMAN | 31341.31629 | 1.35 | 0.353 | 3.48E-05 |
| sp|Q9NQ66|PLCB1_HUMAN | 139316.8154 | 0.87 | 0.179 | 0.0008301 |
| sp|Q8IZ21|PHAR4_HUMAN | 78373.21687 | 0.85 | 0.228 | 0.0009851 |
| sp|Q08170|SRSF4_HUMAN | 56741.36314 | 1.54 | 0.164 | 3.02E-16 |
| sp|Q8N5I2|ARRD1_HUMAN | 46333.84337 | 1.01 | 0.247 | 0.6226 |
| sp|Q13822|ENPP2_HUMAN | 100793.252 | 0.97 | 0.154 | 0.1373 |
| sp|Q6PKG0|LARP1_HUMAN | 123815.3166 | 1.11 | 0.12 | 0.000101 |
| sp|P01911|2B1F_HUMAN | 30271.11828 | 2.59 | 3.364 | 0.1271 |
| sp|Q9H300|PARL_HUMAN | 42543.84515 | 0.7 | 0.139 | 6.94E-10 |
| sp|P49770|EI2BB_HUMAN | 39174.96818 | 0.73 | 0.141 | 1.05E-08 |
| sp|Q9HA72|CAHM2_HUMAN | 36589.78596 | 0.89 | 0.281 | 0.01262 |
| sp|Q99442|SEC62_HUMAN | 46157.25302 | 0.82 | 0.23 | 0.0005057 |
| sp|P26196|DDX6_HUMAN | 54763.44677 | 0.63 | 0.094 | 1.01E-13 |
| sp|Q16576|RBBP7_HUMAN | 48114.29775 | 1.07 | 0.171 | 0.06206 |
| sp|Q9BV36|MELPH_HUMAN | 66575.09146 | 0.65 | 0.219 | 3.00E-06 |
| sp|Q5T1V6|DDX59_HUMAN | 69432.60069 | 0.52 | 0.15 | 1.05E-11 |
| sp|Q99549|MPP8_HUMAN | 97617.97144 | 0.84 | 0.374 | 0.004929 |
| sp|Q3MHD2|LSM12_HUMAN | 21954.22613 | 1.76 | 0.407 | 5.10E-11 |
| sp|O94826|TOM70_HUMAN | 68078.27186 | 1.06 | 0.205 | 0.3314 |
| sp|Q9Y6B6|SAR1B_HUMAN | 22491.53886 | 0.68 | 0.108 | 5.78E-12 |
| sp|Q96JG6|VPS50_HUMAN | 111941.169 | 0.56 | 0.106 | 2.21E-14 |
| sp|Q86U90|YRDC_HUMAN | 29690.43362 | 0.99 | 0.298 | 0.3449 |
| sp|Q9C075|K1C23_HUMAN | 48254.25234 | 0.78 | 0.284 | 0.001073 |
| sp|P14373|TRI27_HUMAN | 59745.24449 | 0.93 | 0.17 | 0.0182 |
| sp|P08779|K1C16_HUMAN | 51560.32077 | 1.97 | 1.017 | 1.95E-05 |
| sp|P31483|TIA1_HUMAN | 43259.70389 | 1.63 | 0.322 | 1.65E-11 |
| sp|O75600|KBL_HUMAN | 45808.40744 | 0.8 | 0.292 | 0.0009783 |
| sp|Q8WUI4|HDAC7_HUMAN | 103586.27 | 1.18 | 0.262 | 0.002786 |
| sp|Q13451|FKBP5_HUMAN | 51674.75061 | 1.29 | 0.142 | 3.24E-11 |
| sp|Q5Y7A7|2B1D_HUMAN | 30313.07115 | 0.99 | 0.311 | 0.3453 |
| sp|O75323|NIPS2_HUMAN | 33931.29218 | 0.79 | 0.237 | 0.0001511 |
| sp|P28289|TMOD1_HUMAN | 40639.91738 | 1.2 | 0.854 | 0.9347 |
| sp|Q6NSJ2|PHLB3_HUMAN | 72590.4917 | 0.71 | 0.114 | 1.07E-10 |
| sp|Q9BV86|NTM1A_HUMAN | 25751.97841 | 1.2 | 0.223 | 0.000119 |
| sp|Q9HB19|PKHA2_HUMAN | 47834.23879 | 1.38 | 0.328 | 1.12E-06 |
| sp|P01766|HV313_HUMAN | 12651.23272 | 1.33 | 0.86 | 0.2701 |
| sp|Q9HAS0|NJMU_HUMAN | 45259.14347 | 0.71 | 0.214 | 1.14E-06 |
| sp|P11279|LAMP1_HUMAN | 45348.99645 | 0.9 | 0.15 | 0.001339 |
| sp|Q13586|STIM1_HUMAN | 77642.38631 | 0.9 | 0.099 | 7.51E-05 |
| sp|O43290|SNUT1_HUMAN | 90353.32048 | 1.56 | 0.357 | 2.50E-09 |
| sp|Q9P0M9|RM27_HUMAN | 16101.74583 | 1.07 | 0.129 | 0.02826 |
| sp|Q9BV40|VAMP8_HUMAN | 11470.01999 | 0.7 | 0.141 | 1.80E-09 |
| sp|Q9NYV4|CDK12_HUMAN | 164720.2313 | 0.81 | 0.113 | 4.67E-08 |
| sp|Q969R5|LMBL2_HUMAN | 80067.84479 | 0.73 | 0.171 | 1.29E-07 |
| sp|Q9UI09|NDUAC_HUMAN | 17085.61791 | 0.83 | 0.264 | 0.002159 |
| sp|O60938|KERA_HUMAN | 40864.07878 | 1.18 | 0.275 | 0.006735 |
| sp|O14867|BACH1_HUMAN | 83826.4531 | 0.9 | 0.098 | 5.50E-05 |
| sp|A0MZ66|SHOT1_HUMAN | 72090.87475 | 1.16 | 0.297 | 0.03781 |
| sp|Q6NVY1|HIBCH_HUMAN | 43778.51573 | 0.68 | 0.216 | 1.90E-06 |
| sp|Q92576|PHF3_HUMAN | 231544.6253 | 1.42 | 0.345 | 2.30E-07 |
| sp|Q9Y3D7|TIM16_HUMAN | 13798.23573 | 0.84 | 0.145 | 9.34E-06 |
[truncated: 95,862 more chars]
