# Supplementary material for: Apolipoprotein C‐II induces EMT to promote gastric cancer peritoneal metastasis via PI3K/AKT/mTOR pathway
Source: Clin Transl Med. 2021 Aug 9;11(8):e522. doi: 10.1002/ctm2.522 (PMC8351524; doi:10.1002/ctm2.522)
Supplement: Supplementary file 17 — Table S2. Compared with the primary GC, 595 proteins in the PM tissues were upregulated among these DEPs. [file CTM2-11-e522-s015.docx]

**Table S2. Compared with the primary GC, 595 proteins in the PM tissues were upregulated among these DEPs.**

| Protein_ID | Mass | Mean_Ratio_  PM-VS-GC | SD_  PM-VS-GC | Pvalue_  PM-VS-GC |
| --- | --- | --- | --- | --- |
| sp\|Q7Z4N2\|TRPM1_HUMAN | 183412.7449 | 8.12 | 2.406 | 2.20E-16 |
| sp\|I1YAP6\|TRI77_HUMAN | 53936.66298 | 7.02 | 3.221 | 2.73E-13 |
| sp\|Q8IXB3\|TARG1_HUMAN | 19338.19162 | 6.33 | 2.515 | 2.20E-16 |
| sp\|Q8N130\|NPT2C_HUMAN | 64289.36355 | 5.55 | 3.145 | 2.61E-11 |
| sp\|Q9HD40\|SPCS_HUMAN | 56413.7561 | 5.13 | 2.441 | 2.47E-14 |
| sp\|Q96QS6\|KPSH2_HUMAN | 43152.39218 | 4.92 | 2.626 | 2.70E-13 |
| sp\|Q5T870\|PRR9_HUMAN | 13903.12075 | 4.58 | 3.021 | 1.23E-10 |
| sp\|Q86YJ7\|AN13B_HUMAN | 70599.87825 | 4.27 | 3.557 | 0.0003462 |
| sp\|P15090\|FABP4_HUMAN | 14805.52948 | 4.1 | 2.121 | 9.55E-12 |
| sp\|P08138\|TNR16_HUMAN | 46732.87921 | 4.07 | 2.008 | 4.73E-13 |
| sp\|Q15847\|ADIRF_HUMAN | 7831.963687 | 3.84 | 2.722 | 4.03E-08 |
| sp\|Q8TDB4\|HUMMR_HUMAN | 25413.25474 | 3.59 | 1.655 | 1.96E-10 |
| sp\|P22676\|CALB2_HUMAN | 31615.60932 | 3.55 | 2.259 | 1.94E-09 |
| sp\|O94769\|ECM2_HUMAN | 80633.53161 | 3.52 | 2.515 | 1.59E-09 |
| sp\|Q96HH4\|TM169_HUMAN | 33970.67822 | 3.46 | 1.576 | 1.58E-10 |
| sp\|P21695\|GPDA_HUMAN | 38152.54346 | 3.41 | 1.291 | 2.65E-13 |
| sp\|P27658\|CO8A1_HUMAN | 73413.15712 | 3.36 | 2.296 | 4.71E-07 |
| sp\|P34982\|OR1D2_HUMAN | 35825.63876 | 3.32 | 2.785 | 0.001055 |
| sp\|P13928\|ANXA8_HUMAN | 37067.72164 | 3.31 | 2.132 | 8.35E-09 |
| sp\|Q30154\|DRB5_HUMAN | 30304.11373 | 3.22 | 2.908 | 2.62E-06 |
| sp\|Q8TF76\|HASP_HUMAN | 89619.32159 | 3.17 | 1.6 | 1.58E-10 |
| sp\|Q63ZE4\|S22AA_HUMAN | 60884.47416 | 3.15 | 2.376 | 8.91E-08 |
| sp\|Q07092\|COGA1_HUMAN | 159459.2192 | 3.11 | 1.92 | 2.08E-09 |
| sp\|P35442\|TSP2_HUMAN | 133767.2214 | 3.11 | 1.24 | 6.59E-11 |
| sp\|P02452\|CO1A1_HUMAN | 139864.9463 | 3.11 | 1.707 | 1.97E-10 |
| sp\|P29373\|RABP2_HUMAN | 15836.07423 | 3.03 | 1.467 | 1.47E-08 |
| sp\|P18827\|SDC1_HUMAN | 32537.88685 | 3 | 2.519 | 0.0001711 |
| sp\|Q9NQS1\|AVEN_HUMAN | 38692.9359 | 2.99 | 0.604 | 2.20E-16 |
| sp\|P23327\|SRCH_HUMAN | 80975.83333 | 2.95 | 2.658 | 2.77E-06 |
| sp\|Q5VST9\|OBSCN_HUMAN | 879611.655 | 2.92 | 2.344 | 6.54E-06 |
| sp\|Q9NYQ7\|CELR3_HUMAN | 362846.9806 | 2.92 | 1.228 | 5.38E-12 |
| sp\|Q8TDR0\|MIPT3_HUMAN | 78908.21555 | 2.83 | 1.309 | 2.60E-10 |
| sp\|Q8NFP7\|NUD10_HUMAN | 18698.28865 | 2.81 | 1.489 | 4.62E-07 |
| sp\|O95670\|VATG2_HUMAN | 13634.97007 | 2.77 | 2.022 | 4.81E-07 |
| sp\|Q9NP74\|PALMD_HUMAN | 62758.73859 | 2.77 | 0.931 | 3.49E-12 |
| sp\|P14209\|CD99_HUMAN | 18875.33875 | 2.77 | 0.969 | 3.02E-13 |
| sp\|P05204\|HMGN2_HUMAN | 9369.046877 | 2.76 | 1.602 | 2.12E-08 |
| sp\|Q9GZU5\|NYX_HUMAN | 52804.61558 | 2.73 | 0.897 | 7.98E-14 |
| sp\|Q04695\|K1C17_HUMAN | 48343.1409 | 2.71 | 2.854 | 0.0009677 |
| sp\|Q9UNZ5\|L10K_HUMAN | 10552.10277 | 2.71 | 2.713 | 0.0001013 |
| sp\|Q96Q06\|PLIN4_HUMAN | 135186.2031 | 2.7 | 1.216 | 5.71E-10 |
| sp\|P08123\|CO1A2_HUMAN | 129730.6257 | 2.66 | 1.032 | 1.10E-11 |
| sp\|P63313\|TYB10_HUMAN | 5004.542395 | 2.64 | 1.006 | 4.88E-12 |
| sp\|A0A075B6I1\|LV460_HUMAN | 13132.32086 | 2.63 | 2.824 | 0.004446 |
| sp\|Q8N4Q1\|MIA40_HUMAN | 16367.19999 | 2.62 | 0.914 | 9.77E-11 |
| sp\|Q9Y3Y2\|CHTOP_HUMAN | 26362.26885 | 2.6 | 1.257 | 1.00E-08 |
| sp\|Q6FHJ7\|SFRP4_HUMAN | 40865.72294 | 2.58 | 1.43 | 0.0001319 |
| sp\|P02533\|K1C14_HUMAN | 51853.50232 | 2.58 | 2.003 | 1.54E-06 |
| sp\|P29966\|MARCS_HUMAN | 31688.91859 | 2.56 | 0.647 | 1.07E-14 |
| sp\|Q02952\|AKA12_HUMAN | 191918.8731 | 2.55 | 0.608 | 1.55E-14 |
| sp\|Q14117\|DPYS_HUMAN | 57088.87828 | 2.54 | 1.635 | 5.27E-05 |
| sp\|Q96LT7\|CI072_HUMAN | 54788.18109 | 2.54 | 2.263 | 0.006608 |
| sp\|Q8IW70\|T151B_HUMAN | 62703.13172 | 2.51 | 1.422 | 3.17E-07 |
| sp\|P10321\|1C07_HUMAN | 41118.12727 | 2.49 | 2.469 | 0.0009277 |
| sp\|P09936\|UCHL1_HUMAN | 25132.57604 | 2.48 | 0.765 | 7.51E-13 |
| sp\|Q9Y5J5\|PHLA3_HUMAN | 14035.47755 | 2.47 | 0.632 | 7.87E-15 |
| sp\|P01705\|LV223_HUMAN | 12038.74996 | 2.47 | 2.224 | 0.01226 |
| sp\|Q9Y5Y7\|LYVE1_HUMAN | 35743.16129 | 2.46 | 1.387 | 2.38E-06 |
| sp\|Q15434\|RBMS2_HUMAN | 44140.76491 | 2.41 | 1.061 | 1.04E-09 |
| sp\|P07585\|PGS2_HUMAN | 40045.95757 | 2.4 | 1.63 | 3.41E-07 |
| sp\|Q96PQ0\|SORC2_HUMAN | 129079.4578 | 2.4 | 0.862 | 2.27E-11 |
| sp\|P16402\|H13_HUMAN | 22318.30861 | 2.38 | 1.669 | 0.00414 |
| sp\|O15523\|DDX3Y_HUMAN | 73546.28242 | 2.36 | 1.448 | 1.06E-06 |
| sp\|Q6ZMR5\|TM11A_HUMAN | 47976.6245 | 2.35 | 1.44 | 3.91E-07 |
| sp\|P05997\|CO5A2_HUMAN | 145772.3292 | 2.35 | 0.968 | 2.49E-10 |
| sp\|Q9Y279\|VSIG4_HUMAN | 44511.24324 | 2.33 | 0.726 | 5.20E-12 |
| sp\|Q92876\|KLK6_HUMAN | 27504.60295 | 2.33 | 1.97 | 0.001586 |
| sp\|Q8WWZ4\|ABCAA_HUMAN | 177425.4189 | 2.32 | 2.469 | 0.02177 |
| sp\|P51884\|LUM_HUMAN | 38728.91284 | 2.31 | 1.362 | 1.30E-07 |
| sp\|Q16082\|HSPB2_HUMAN | 20259.16386 | 2.31 | 0.754 | 6.46E-11 |
| sp\|P04271\|S100B_HUMAN | 10802.08211 | 2.28 | 0.968 | 6.13E-08 |
| sp\|P01861\|IGHG4_HUMAN | 36413.04851 | 2.27 | 1.473 | 8.65E-06 |
| sp\|P02655\|APOC2_HUMAN | 11258.74304 | 2.27 | 1.148 | 6.80E-06 |
| sp\|Q8NH73\|OR4S2_HUMAN | 35814.41856 | 2.27 | 0.985 | 9.22E-09 |
| sp\|P13761\|2B17_HUMAN | 30127.07603 | 2.25 | 1.807 | 0.01058 |
| sp\|P01593\|KVD33_HUMAN | 12993.32244 | 2.24 | 1.178 | 1.18E-07 |
| sp\|Q92729\|PTPRU_HUMAN | 164240.762 | 2.24 | 1.17 | 1.91E-06 |
| sp\|Q9UMX1\|SUFU_HUMAN | 54237.12013 | 2.24 | 0.515 | 5.28E-14 |
| sp\|Q9NZD4\|AHSP_HUMAN | 11814.96985 | 2.23 | 0.697 | 1.01E-10 |
| sp\|I3L1E1\|CS084_HUMAN | 19811.96825 | 2.22 | 1.013 | 6.92E-09 |
| sp\|Q03405\|UPAR_HUMAN | 38588.87068 | 2.22 | 2.264 | 0.0048 |
| sp\|Q9UM22\|EPDR1_HUMAN | 25858.75199 | 2.22 | 0.607 | 8.17E-13 |
| sp\|Q9Y680\|FKBP7_HUMAN | 25931.00088 | 2.22 | 0.635 | 6.63E-12 |
| sp\|Q6UX06\|OLFM4_HUMAN | 57510.89314 | 2.2 | 2.362 | 0.01395 |
| sp\|Q16777\|H2A2C_HUMAN | 13961.83074 | 2.2 | 1.223 | 8.60E-06 |
| sp\|P49747\|COMP_HUMAN | 85412.59322 | 2.19 | 1.013 | 1.16E-07 |
| sp\|P02461\|CO3A1_HUMAN | 139715.433 | 2.19 | 0.801 | 4.52E-09 |
| sp\|Q9HCU0\|CD248_HUMAN | 82785.00788 | 2.18 | 0.611 | 7.57E-12 |
| sp\|P02792\|FRIL_HUMAN | 20046.11312 | 2.17 | 0.99 | 2.20E-08 |
| sp\|Q9BTM1\|H2AJ_HUMAN | 13992.91585 | 2.16 | 1.552 | 0.0001509 |
| sp\|P08670\|VIME_HUMAN | 53658.08957 | 2.15 | 0.574 | 3.14E-13 |
| sp\|Q86YZ3\|HORN_HUMAN | 283121.9613 | 2.13 | 1.782 | 0.0003369 |
| sp\|Q96S42\|NODAL_HUMAN | 40088.02769 | 2.12 | 0.969 | 2.37E-08 |
| sp\|P28330\|ACADL_HUMAN | 48006.48637 | 2.12 | 0.882 | 3.94E-08 |
| sp\|Q63HM2\|PCX4_HUMAN | 133738.2354 | 2.12 | 1.078 | 7.88E-07 |
| sp\|Q9H4P4\|RNF41_HUMAN | 36662.00263 | 2.12 | 0.71 | 9.23E-11 |
| sp\|P21810\|PGS1_HUMAN | 42008.67682 | 2.12 | 0.667 | 2.37E-11 |
| sp\|Q7Z7F7\|RM55_HUMAN | 15101.10683 | 2.12 | 1.217 | 6.23E-06 |
| sp\|P02511\|CRYAB_HUMAN | 20128.40877 | 2.1 | 0.703 | 9.74E-10 |
| sp\|Q68DK7\|MSL1_HUMAN | 67752.87847 | 2.1 | 0.963 | 5.78E-08 |
| sp\|Q13424\|SNTA1_HUMAN | 54186.02479 | 2.09 | 0.894 | 2.13E-06 |
| sp\|Q9P2R6\|RERE_HUMAN | 173439.2192 | 2.09 | 0.783 | 4.01E-09 |
| sp\|Q96DC8\|ECHD3_HUMAN | 32937.0688 | 2.09 | 0.694 | 2.31E-09 |
| sp\|Q9H254\|SPTN4_HUMAN | 289987.0446 | 2.09 | 1.31 | 0.0001953 |
| sp\|P07451\|CAH3_HUMAN | 29805.80315 | 2.08 | 1.016 | 9.36E-08 |
| sp\|P00167\|CYB5_HUMAN | 15302.49613 | 2.07 | 0.717 | 1.60E-09 |
| sp\|Q14011\|CIRBP_HUMAN | 18618.71805 | 2.07 | 0.432 | 7.77E-15 |
| sp\|Q96SL4\|GPX7_HUMAN | 21135.82541 | 2.06 | 0.616 | 3.63E-11 |
| sp\|A0A075B6K4\|LV310_HUMAN | 12586.00696 | 2.06 | 0.86 | 5.70E-09 |
| sp\|O60240\|PLIN1_HUMAN | 56222.89197 | 2.06 | 0.415 | 7.71E-15 |
| sp\|A0A075B6I0\|LV861_HUMAN | 12902.15602 | 2.05 | 1.5 | 0.000101 |
| sp\|P50454\|SERPH_HUMAN | 46507.20959 | 2.05 | 0.626 | 1.02E-10 |
| sp\|P55008\|AIF1_HUMAN | 16674.59802 | 2.05 | 0.571 | 4.75E-12 |
| sp\|Q99541\|PLIN2_HUMAN | 48255.51624 | 2.04 | 1.334 | 0.0003639 |
| sp\|Q9NRN5\|OLFL3_HUMAN | 46362.40792 | 2.04 | 1.124 | 1.82E-06 |
| sp\|Q9UQN3\|CHM2B_HUMAN | 23930.22293 | 2.03 | 0.783 | 2.47E-08 |
| sp\|Q9UL45\|BL1S6_HUMAN | 19827.28937 | 2.03 | 0.487 | 4.35E-13 |
| sp\|O95236\|APOL3_HUMAN | 44631.86332 | 2.03 | 0.532 | 1.46E-12 |
| sp\|P10412\|H14_HUMAN | 21833.99376 | 2.03 | 0.747 | 2.89E-08 |
| sp\|Q9BW62\|KATL1_HUMAN | 55681.3452 | 2.02 | 0.326 | 2.20E-16 |
| sp\|P45381\|ACY2_HUMAN | 36207.269 | 2.02 | 0.697 | 3.24E-09 |
| sp\|Q9P1Y6\|PHRF1_HUMAN | 180250.1002 | 2.02 | 0.357 | 2.20E-16 |
| sp\|Q96PP4\|TSG13_HUMAN | 31796.72378 | 2.01 | 0.888 | 4.02E-07 |
| sp\|Q6UWY5\|OLFL1_HUMAN | 46359.52404 | 2.01 | 1.323 | 8.84E-05 |
| sp\|O95295\|SNAPN_HUMAN | 14903.90372 | 2.01 | 0.382 | 1.25E-15 |
| sp\|O76070\|SYUG_HUMAN | 13304.76361 | 2.01 | 1.056 | 0.0001036 |
| sp\|P10636\|TAU_HUMAN | 79089.84363 | 2 | 0.566 | 9.85E-11 |
| sp\|O60522\|TDRD6_HUMAN | 239827.4539 | 2 | 0.713 | 6.20E-09 |
| sp\|E9PRG8\|CK098_HUMAN | 13771.6388 | 2 | 0.787 | 1.59E-07 |
| sp\|Q9BRP8\|PYM1_HUMAN | 22623.97672 | 2 | 0.649 | 2.25E-10 |
| sp\|Q8TEK3\|DOT1L_HUMAN | 165478.1439 | 2 | 1.002 | 7.87E-06 |
| sp\|O75781\|PALM_HUMAN | 42203.21341 | 2 | 0.543 | 6.66E-12 |
| sp\|Q15714\|T22D1_HUMAN | 109648.9297 | 1.99 | 1.188 | 1.39E-05 |
| sp\|O75891\|AL1L1_HUMAN | 99603.8764 | 1.99 | 0.446 | 3.24E-13 |
| sp\|Q9H694\|BICC1_HUMAN | 105387.1159 | 1.99 | 0.601 | 2.56E-10 |
| sp\|O14531\|DPYL4_HUMAN | 62447.63327 | 1.98 | 0.64 | 6.27E-10 |
| sp\|Q6ZRQ5\|MMS22_HUMAN | 144092.1258 | 1.98 | 0.778 | 8.62E-08 |
| sp\|P13611\|CSPG2_HUMAN | 374567.2778 | 1.98 | 0.446 | 1.36E-12 |
| sp\|Q9BS40\|LXN_HUMAN | 25829.92403 | 1.98 | 0.479 | 8.77E-12 |
| sp\|Q9BUP0\|EFHD1_HUMAN | 27006.85585 | 1.97 | 0.721 | 4.99E-10 |
| sp\|Q495B1\|AKD1A_HUMAN | 57895.58765 | 1.97 | 1.215 | 0.001169 |
| sp\|Q16799\|RTN1_HUMAN | 83833.14156 | 1.97 | 0.538 | 2.94E-11 |
| sp\|Q9Y2S6\|TMA7_HUMAN | 7043.922018 | 1.97 | 0.876 | 1.71E-06 |
| sp\|P08779\|K1C16_HUMAN | 51560.32077 | 1.97 | 1.017 | 1.95E-05 |
| sp\|Q9H0M0\|WWP1_HUMAN | 105973.2863 | 1.96 | 1.016 | 0.0002576 |
| sp\|P07333\|CSF1R_HUMAN | 109094.5703 | 1.96 | 0.947 | 2.26E-07 |
| sp\|P53674\|CRBB1_HUMAN | 28044.89907 | 1.95 | 0.466 | 1.61E-13 |
| sp\|Q9BQI9\|NRIP2_HUMAN | 31806.34751 | 1.95 | 0.586 | 2.62E-10 |
| sp\|P0DOY2\|IGLC2_HUMAN | 11439.60314 | 1.95 | 0.791 | 8.25E-08 |
| sp\|Q96SM3\|CPXM1_HUMAN | 82225.26279 | 1.95 | 0.597 | 1.14E-10 |
| sp\|Q96S96\|PEBP4_HUMAN | 25983.86638 | 1.95 | 1.101 | 5.01E-05 |
| sp\|Q53RD9\|FBLN7_HUMAN | 49037.35843 | 1.94 | 1.237 | 2.91E-05 |
| sp\|Q16626\|MEA1_HUMAN | 19874.40083 | 1.94 | 1.091 | 0.0001034 |
| sp\|P04259\|K2C6B_HUMAN | 60297.38777 | 1.94 | 1.075 | 5.83E-05 |
| sp\|P62857\|RS28_HUMAN | 7875.212297 | 1.94 | 0.784 | 9.05E-08 |
| sp\|Q9H875\|PKRI1_HUMAN | 20965.92735 | 1.93 | 0.868 | 4.95E-06 |
| sp\|Q96HF1\|SFRP2_HUMAN | 34476.1688 | 1.93 | 0.66 | 3.07E-08 |
| sp\|P08493\|MGP_HUMAN | 12498.35276 | 1.93 | 1.11 | 0.0001491 |
| sp\|O43665\|RGS10_HUMAN | 20318.98908 | 1.92 | 0.332 | 3.82E-15 |
| sp\|P24592\|IBP6_HUMAN | 26200.49621 | 1.92 | 0.821 | 3.25E-07 |
| sp\|Q9H9F9\|ARP5_HUMAN | 68806.54566 | 1.92 | 0.682 | 3.90E-09 |
| sp\|Q99584\|S10AD_HUMAN | 11446.07932 | 1.92 | 0.545 | 9.93E-12 |
| sp\|Q13151\|ROA0_HUMAN | 30974.83891 | 1.91 | 1.063 | 8.24E-05 |
| sp\|O75791\|GRAP2_HUMAN | 37924.73999 | 1.91 | 0.988 | 6.84E-05 |
| sp\|P55854\|SUMO3_HUMAN | 11668.73994 | 1.91 | 0.427 | 9.12E-14 |
| sp\|O00193\|SMAP_HUMAN | 20359.22652 | 1.9 | 0.988 | 1.93E-06 |
| sp\|O43181\|NDUS4_HUMAN | 20077.42175 | 1.89 | 0.793 | 4.98E-06 |
| sp\|Q16643\|DREB_HUMAN | 71823.59969 | 1.89 | 0.445 | 8.83E-12 |
| sp\|O94929\|ABLM3_HUMAN | 79444.2542 | 1.89 | 0.589 | 6.54E-10 |
| sp\|Q9UI08\|EVL_HUMAN | 44688.24969 | 1.88 | 0.504 | 7.53E-11 |
| sp\|Q4V9L6\|TM119_HUMAN | 29394.72454 | 1.88 | 0.999 | 4.91E-05 |
| sp\|Q9H147\|TDIF1_HUMAN | 37256.97853 | 1.88 | 0.26 | 2.20E-16 |
| sp\|P80723\|BASP1_HUMAN | 22662.00121 | 1.88 | 0.431 | 1.04E-12 |
| sp\|P01706\|LV211_HUMAN | 12789.07376 | 1.88 | 0.6 | 8.09E-09 |
| sp\|P49863\|GRAK_HUMAN | 29358.90069 | 1.88 | 0.601 | 8.95E-09 |
| sp\|Q76N32\|CEP68_HUMAN | 81946.4686 | 1.88 | 0.857 | 1.34E-06 |
| sp\|Q96PP8\|GBP5_HUMAN | 67127.17702 | 1.88 | 1.627 | 0.004592 |
| sp\|Q9H1E3\|NUCKS_HUMAN | 27262.02158 | 1.88 | 0.486 | 9.51E-12 |
| sp\|P06312\|KV401_HUMAN | 13467.62507 | 1.88 | 0.601 | 8.25E-10 |
| sp\|P08047\|SP1_HUMAN | 81252.82897 | 1.87 | 0.324 | 4.42E-15 |
| sp\|O14602\|IF1AY_HUMAN | 16528.30078 | 1.87 | 0.77 | 6.52E-06 |
| sp\|P20774\|MIME_HUMAN | 34225.00284 | 1.87 | 1.01 | 4.65E-06 |
| sp\|O75339\|CILP1_HUMAN | 134742.8455 | 1.87 | 0.753 | 2.26E-08 |
| sp\|Q9H7E9\|CH033_HUMAN | 25301.01465 | 1.87 | 0.509 | 2.59E-10 |
| sp\|Q96J88\|ESIP1_HUMAN | 36923.99295 | 1.87 | 0.545 | 1.67E-10 |
| sp\|Q13342\|SP140_HUMAN | 100251.768 | 1.87 | 0.694 | 8.42E-07 |
| sp\|Q5U5Q3\|MEX3C_HUMAN | 69989.43178 | 1.87 | 0.468 | 1.06E-10 |
| sp\|P13674\|P4HA1_HUMAN | 61278.12396 | 1.87 | 0.525 | 4.39E-10 |
| sp\|Q6NZI2\|CAVN1_HUMAN | 43431.80874 | 1.86 | 0.384 | 4.03E-12 |
| sp\|Q9NVA2\|SEP11_HUMAN | 49634.29069 | 1.86 | 0.321 | 1.48E-14 |
| sp\|O75348\|VATG1_HUMAN | 13845.11542 | 1.86 | 0.633 | 2.17E-09 |
| sp\|A0A0C4DH68\|KV224_HUMAN | 13166.66975 | 1.86 | 0.874 | 6.25E-05 |
| sp\|P61956\|SUMO2_HUMAN | 10903.37423 | 1.86 | 0.32 | 2.32E-15 |
| sp\|P54725\|RD23A_HUMAN | 39623.65917 | 1.86 | 0.277 | 2.20E-16 |
| sp\|P17096\|HMGA1_HUMAN | 11651.13157 | 1.86 | 0.922 | 4.44E-05 |
| sp\|P52594\|AGFG1_HUMAN | 58491.26064 | 1.86 | 0.825 | 7.16E-06 |
| sp\|Q9BPZ3\|PAIP2_HUMAN | 15013.88916 | 1.85 | 0.498 | 4.17E-10 |
| sp\|Q8IUX7\|AEBP1_HUMAN | 131570.2643 | 1.85 | 0.536 | 6.07E-10 |
| sp\|O14682\|ENC1_HUMAN | 67266.56822 | 1.84 | 0.452 | 1.63E-11 |
| sp\|Q9UKY7\|CDV3_HUMAN | 27300.34959 | 1.84 | 0.636 | 6.99E-08 |
| sp\|Q8IWU6\|SULF1_HUMAN | 102369.9872 | 1.84 | 0.702 | 1.46E-06 |
| sp\|P0C7T5\|ATX1L_HUMAN | 73755.95887 | 1.83 | 0.325 | 4.65E-15 |
| sp\|Q99983\|OMD_HUMAN | 49955.40552 | 1.83 | 0.664 | 3.61E-07 |
| sp\|Q9UQP3\|TENN_HUMAN | 145750.2312 | 1.83 | 1.206 | 0.0009921 |
| sp\|Q9H446\|RWDD1_HUMAN | 27904.37237 | 1.83 | 0.3 | 9.10E-16 |
| sp\|Q96PU8\|QKI_HUMAN | 37742.75894 | 1.83 | 0.287 | 7.58E-16 |
| sp\|O60307\|MAST3_HUMAN | 143829.1026 | 1.82 | 0.474 | 5.57E-11 |
| sp\|Q5QJE6\|TDIF2_HUMAN | 84799.00424 | 1.82 | 0.68 | 1.29E-07 |
| sp\|P23025\|XPA_HUMAN | 31728.89901 | 1.82 | 0.761 | 5.24E-06 |
| sp\|O75607\|NPM3_HUMAN | 19598.49799 | 1.82 | 0.762 | 4.21E-05 |
| sp\|Q70UQ0\|IKIP_HUMAN | 39380.99198 | 1.82 | 0.644 | 2.47E-07 |
| sp\|P23381\|SYWC_HUMAN | 53455.72504 | 1.82 | 1.582 | 0.0135 |
| sp\|P60903\|S10AA_HUMAN | 11291.54361 | 1.82 | 0.399 | 1.03E-12 |
| sp\|Q96S99\|PKHF1_HUMAN | 31840.95574 | 1.82 | 0.271 | 2.59E-16 |
| sp\|Q8N3Y1\|FBXW8_HUMAN | 67846.78961 | 1.82 | 0.451 | 1.58E-10 |
| sp\|O00499\|BIN1_HUMAN | 64869.46935 | 1.82 | 0.273 | 2.46E-16 |
| sp\|Q495T6\|MMEL1_HUMAN | 89976.31941 | 1.82 | 0.593 | 1.05E-07 |
| sp\|Q86WV1\|SKAP1_HUMAN | 41673.89 | 1.82 | 0.606 | 2.10E-08 |
| sp\|Q16836\|HCDH_HUMAN | 34310.9471 | 1.81 | 0.642 | 2.40E-07 |
| sp\|Q5HYK7\|SH319_HUMAN | 87023.93735 | 1.81 | 0.472 | 1.89E-10 |
| sp\|H7BZ55\|CRCC2_HUMAN | 186882.2984 | 1.81 | 1.262 | 0.002008 |
| sp\|Q32P28\|P3H1_HUMAN | 84178.39806 | 1.8 | 0.481 | 4.67E-10 |
| sp\|Q08495\|DEMA_HUMAN | 45582.25677 | 1.8 | 0.334 | 8.92E-14 |
| sp\|P16989\|YBOX3_HUMAN | 40048.01526 | 1.8 | 0.234 | 2.20E-16 |
| sp\|P98082\|DAB2_HUMAN | 82493.03253 | 1.8 | 0.412 | 7.68E-12 |
| sp\|Q01664\|TFAP4_HUMAN | 38797.82704 | 1.8 | 0.725 | 1.66E-06 |
| sp\|P20290\|BTF3_HUMAN | 22193.43005 | 1.8 | 0.449 | 1.06E-10 |
| sp\|Q8TBZ0\|CC110_HUMAN | 97217.35972 | 1.79 | 1.166 | 0.0005742 |
| sp\|A0A087WW87\|KV240_HUMAN | 13397.62633 | 1.79 | 1.086 | 8.59E-05 |
| sp\|P01701\|LV151_HUMAN | 12451.02976 | 1.79 | 0.444 | 8.50E-11 |
| sp\|O43715\|TRIA1_HUMAN | 8990.185277 | 1.79 | 0.778 | 1.80E-06 |
| sp\|Q8WZ42\|TITIN_HUMAN | 3842885.76 | 1.79 | 0.867 | 0.00012 |
| sp\|Q14257\|RCN2_HUMAN | 36892.65357 | 1.79 | 0.271 | 3.57E-16 |
| sp\|P08729\|K2C7_HUMAN | 51393.3185 | 1.79 | 1.125 | 0.01546 |
| sp\|Q9H3H9\|TCAL2_HUMAN | 25929.71591 | 1.79 | 0.771 | 3.28E-05 |
| sp\|Q14157\|UBP2L_HUMAN | 114561.3029 | 1.79 | 0.637 | 1.50E-08 |
| sp\|Q99102\|MUC4_HUMAN | 233978.4107 | 1.79 | 0.574 | 1.97E-09 |
| sp\|P27816\|MAP4_HUMAN | 121425.0087 | 1.79 | 0.252 | 2.27E-16 |
| sp\|O95810\|CAVN2_HUMAN | 47183.59925 | 1.79 | 0.533 | 1.24E-08 |
| sp\|Q8NEF9\|SRFB1_HUMAN | 48700.45152 | 1.78 | 0.49 | 1.99E-09 |
| sp\|Q15772\|SPEG_HUMAN | 356846.614 | 1.78 | 0.485 | 1.36E-10 |
| sp\|P13647\|K2C5_HUMAN | 62550.05227 | 1.78 | 0.492 | 1.07E-09 |
| sp\|P05534\|1A24_HUMAN | 40930.12816 | 1.78 | 0.917 | 0.000289 |
| sp\|P13501\|CCL5_HUMAN | 10250.15385 | 1.78 | 0.462 | 6.64E-11 |
| sp\|Q6ZNA5\|FRRS1_HUMAN | 66737.64316 | 1.78 | 0.632 | 6.78E-08 |
| sp\|P55209\|NP1L1_HUMAN | 45613.03048 | 1.78 | 0.246 | 2.20E-16 |
| sp\|Q92569\|P55G_HUMAN | 54680.98611 | 1.77 | 0.635 | 5.82E-08 |
| sp\|P23497\|SP100_HUMAN | 101476.5471 | 1.77 | 0.314 | 2.12E-13 |
| sp\|Q13526\|PIN1_HUMAN | 18328.00262 | 1.77 | 0.458 | 1.37E-10 |
| sp\|Q5T619\|ZN648_HUMAN | 63765.61688 | 1.77 | 0.829 | 1.71E-05 |
| sp\|C9JLW8\|MCRI1_HUMAN | 10895.6023 | 1.77 | 0.185 | 2.20E-16 |
| sp\|P48681\|NEST_HUMAN | 177770.1877 | 1.77 | 0.284 | 2.76E-15 |
| sp\|P00813\|ADA_HUMAN | 41005.75309 | 1.76 | 0.25 | 3.07E-16 |
| sp\|P02652\|APOA2_HUMAN | 11263.92973 | 1.76 | 0.639 | 2.80E-07 |
| sp\|Q13480\|GAB1_HUMAN | 77177.73396 | 1.76 | 0.435 | 8.70E-11 |
| sp\|Q2M1P5\|KIF7_HUMAN | 151389.2619 | 1.76 | 0.43 | 3.91E-11 |
| sp\|Q3MHD2\|LSM12_HUMAN | 21954.22613 | 1.76 | 0.407 | 5.10E-11 |
| sp\|Q8IXS6\|PALM2_HUMAN | 42369.33004 | 1.76 | 0.548 | 2.34E-08 |
| sp\|Q7Z7G0\|TARSH_HUMAN | 119235.0061 | 1.76 | 0.996 | 0.0001124 |
| sp\|Q8N3V7\|SYNPO_HUMAN | 99897.31704 | 1.76 | 0.434 | 2.88E-10 |
| sp\|P78537\|BL1S1_HUMAN | 17290.86941 | 1.75 | 0.308 | 2.07E-13 |
| sp\|P67809\|YBOX1_HUMAN | 35884.66505 | 1.75 | 0.466 | 7.26E-10 |
| sp\|Q8WVE0\|EFMT1_HUMAN | 24871.00576 | 1.75 | 0.159 | 2.20E-16 |
| sp\|Q6UXH1\|CREL2_HUMAN | 40314.63838 | 1.75 | 0.468 | 1.01E-10 |
| sp\|Q8WTS1\|ABHD5_HUMAN | 39508.85744 | 1.75 | 0.494 | 2.39E-09 |
| sp\|P00568\|KAD1_HUMAN | 21717.33963 | 1.75 | 0.399 | 4.92E-11 |
| sp\|P16104\|H2AX_HUMAN | 15117.40454 | 1.74 | 0.563 | 2.20E-08 |
| sp\|Q9P2D7\|DYH1_HUMAN | 491139.8177 | 1.74 | 0.817 | 9.62E-06 |
| sp\|A6NMZ7\|CO6A6_HUMAN | 248654.3659 | 1.74 | 0.632 | 5.00E-08 |
| sp\|Q9GZV4\|IF5A2_HUMAN | 17106.35598 | 1.74 | 0.241 | 2.20E-16 |
| sp\|Q7Z401\|MYCPP_HUMAN | 211772.9003 | 1.74 | 0.598 | 9.65E-08 |
| sp\|Q5TCZ1\|SPD2A_HUMAN | 125878.7881 | 1.74 | 0.317 | 6.81E-13 |
| sp\|Q8N668\|COMD1_HUMAN | 21203.91552 | 1.74 | 0.17 | 2.20E-16 |
| sp\|P51888\|PRELP_HUMAN | 44163.29495 | 1.74 | 1.038 | 0.0001697 |
| sp\|Q9Y5J9\|TIM8B_HUMAN | 9547.611566 | 1.73 | 0.647 | 3.34E-06 |
| sp\|P31323\|KAP3_HUMAN | 46654.07392 | 1.73 | 0.557 | 7.87E-08 |
| sp\|Q9P2B4\|CT2NL_HUMAN | 70552.95552 | 1.73 | 0.227 | 2.20E-16 |
| sp\|Q9NQ92\|COPRS_HUMAN | 20206.99981 | 1.73 | 0.696 | 5.60E-05 |
| sp\|Q99439\|CNN2_HUMAN | 34056.43994 | 1.73 | 0.541 | 1.91E-07 |
| sp\|Q16774\|KGUA_HUMAN | 21751.2126 | 1.72 | 0.195 | 2.20E-16 |
| sp\|Q5T3U5\|MRP7_HUMAN | 163048.5367 | 1.72 | 1.43 | 0.04558 |
| sp\|Q9ULD9\|ZN608_HUMAN | 163116.7601 | 1.72 | 0.53 | 0.02022 |
| sp\|Q9NSI8\|SAMN1_HUMAN | 41949.48034 | 1.72 | 1.052 | 0.0004751 |
| sp\|P82979\|SARNP_HUMAN | 23695.44041 | 1.72 | 0.71 | 5.93E-07 |
| sp\|Q96CW6\|S7A6O_HUMAN | 35216.78853 | 1.72 | 0.27 | 1.16E-14 |
| sp\|Q16270\|IBP7_HUMAN | 30119.8177 | 1.72 | 0.405 | 3.09E-11 |
| sp\|P46821\|MAP1B_HUMAN | 271647.4507 | 1.72 | 0.355 | 1.48E-11 |
| sp\|Q9BT43\|RPC7L_HUMAN | 25300.42772 | 1.72 | 0.416 | 1.36E-09 |
| sp\|O43236\|SEPT4_HUMAN | 55501.78383 | 1.72 | 0.448 | 2.49E-10 |
| sp\|Q9NRB3\|CHSTC_HUMAN | 48651.03794 | 1.72 | 0.894 | 0.0001745 |
| sp\|Q9BQ61\|TRIR_HUMAN | 18447.24512 | 1.72 | 0.797 | 0.0001358 |
| sp\|P37840\|SYUA_HUMAN | 14433.20851 | 1.72 | 0.262 | 2.64E-14 |
| sp\|Q13627\|DYR1A_HUMAN | 86025.16619 | 1.72 | 0.791 | 0.0001304 |
| sp\|P14902\|I23O1_HUMAN | 45735.35229 | 1.71 | 1.248 | 0.004415 |
| sp\|P51608\|MECP2_HUMAN | 52561.70853 | 1.71 | 0.406 | 1.79E-10 |
| sp\|Q9Y2W1\|TR150_HUMAN | 108640.0278 | 1.71 | 0.451 | 1.39E-09 |
| sp\|Q13123\|RED_HUMAN | 65657.79999 | 1.71 | 0.38 | 2.78E-11 |
| sp\|A0A075B6J9\|LV218_HUMAN | 12499.98392 | 1.71 | 0.665 | 8.77E-06 |
| sp\|O15117\|FYB1_HUMAN | 85545.20731 | 1.71 | 0.458 | 2.52E-09 |
| sp\|Q9BTL3\|RAMAC_HUMAN | 14354.65426 | 1.71 | 0.229 | 3.51E-16 |
| sp\|Q8IVM0\|CCD50_HUMAN | 35896.00776 | 1.71 | 0.217 | 2.20E-16 |
| sp\|Q8NCF5\|NF2IP_HUMAN | 45941.80191 | 1.7 | 0.649 | 3.20E-06 |
| sp\|Q99961\|SH3G1_HUMAN | 41674.11851 | 1.7 | 0.19 | 2.20E-16 |
| sp\|P04908\|H2A1B_HUMAN | 14108.93938 | 1.7 | 0.576 | 6.89E-08 |
| sp\|Q53F19\|NCBP3_HUMAN | 70645.36358 | 1.7 | 0.805 | 0.00024 |
| sp\|P54259\|ATN1_HUMAN | 125490.6503 | 1.69 | 0.24 | 4.74E-15 |
| sp\|P62841\|RS15_HUMAN | 17011.16275 | 1.69 | 0.459 | 5.53E-09 |
| sp\|Q07021\|C1QBP_HUMAN | 31723.74809 | 1.69 | 0.467 | 5.16E-09 |
| sp\|P41208\|CETN2_HUMAN | 19707.86431 | 1.69 | 0.392 | 1.04E-10 |
| sp\|Q13148\|TADBP_HUMAN | 45035.42453 | 1.69 | 0.499 | 8.90E-09 |
| sp\|Q86SQ7\|SDCG8_HUMAN | 83410.95764 | 1.69 | 0.205 | 2.20E-16 |
| sp\|P36955\|PEDF_HUMAN | 46436.35917 | 1.69 | 0.541 | 2.12E-07 |
| sp\|P20908\|CO5A1_HUMAN | 184113.3047 | 1.69 | 0.467 | 6.33E-09 |
| sp\|Q6ZS72\|PEAK3_HUMAN | 51029.89904 | 1.69 | 1.261 | 0.007338 |
| sp\|P14649\|MYL6B_HUMAN | 22845.72614 | 1.68 | 0.351 | 2.38E-12 |
| sp\|Q8WW12\|PCNP_HUMAN | 18895.38927 | 1.68 | 0.508 | 1.13E-07 |
| sp\|Q9UM47\|NOTC3_HUMAN | 256622.0841 | 1.68 | 0.522 | 7.15E-08 |
| sp\|P55036\|PSMD4_HUMAN | 40921.27378 | 1.68 | 0.259 | 2.40E-14 |
| sp\|O15014\|ZN609_HUMAN | 152334.134 | 1.68 | 0.415 | 8.40E-10 |
| sp\|Q99932\|SPAG8_HUMAN | 51772.73079 | 1.68 | 0.66 | 7.34E-06 |
| sp\|P43487\|RANG_HUMAN | 23448.62422 | 1.68 | 0.483 | 1.29E-08 |
| sp\|O95218\|ZRAB2_HUMAN | 37820.18281 | 1.68 | 0.832 | 3.36E-05 |
| sp\|Q9P270\|SLAI2_HUMAN | 62715.35236 | 1.68 | 0.495 | 5.47E-08 |
| sp\|O00763\|ACACB_HUMAN | 278342.7525 | 1.68 | 0.467 | 3.83E-09 |
| sp\|Q9Y2D5\|AKAP2_HUMAN | 94983.91253 | 1.68 | 0.179 | 2.20E-16 |
| sp\|P16403\|H12_HUMAN | 21333.73698 | 1.68 | 0.654 | 1.05E-05 |
| sp\|Q96GM8\|TOE1_HUMAN | 57349.29879 | 1.68 | 0.334 | 1.23E-12 |
| sp\|Q96RL1\|UIMC1_HUMAN | 80857.36687 | 1.68 | 0.279 | 8.97E-14 |
| sp\|Q641Q2\|WAC2A_HUMAN | 147247.945 | 1.67 | 0.305 | 1.63E-12 |
| sp\|Q9UQ03\|COR2B_HUMAN | 55470.40276 | 1.67 | 0.443 | 3.28E-09 |
| sp\|Q76M96\|CCD80_HUMAN | 108487.0664 | 1.67 | 0.325 | 6.49E-12 |
| sp\|P01709\|LV208_HUMAN | 12469.92707 | 1.67 | 0.29 | 2.85E-13 |
| sp\|Q8IXM2\|BAP18_HUMAN | 17928.32794 | 1.67 | 0.309 | 1.75E-12 |
| sp\|Q16890\|TPD53_HUMAN | 22474.07721 | 1.67 | 0.629 | 3.06E-05 |
| sp\|Q9BQI0\|AIF1L_HUMAN | 17095.63241 | 1.67 | 0.644 | 7.64E-06 |
| sp\|Q9BU40\|CRDL1_HUMAN | 53054.4315 | 1.67 | 0.532 | 1.63E-07 |
| sp\|Q96B54\|ZN428_HUMAN | 20849.09123 | 1.67 | 0.366 | 1.29E-10 |
| sp\|Q09428\|ABCC8_HUMAN | 178457.6364 | 1.67 | 0.8 | 8.56E-05 |
| sp\|P28300\|LYOX_HUMAN | 47580.76705 | 1.66 | 0.264 | 7.07E-14 |
| sp\|Q8IUD2\|RB6I2_HUMAN | 128217.6593 | 1.66 | 0.333 | 3.34E-12 |
| sp\|O15460\|P4HA2_HUMAN | 61244.764 | 1.66 | 0.565 | 7.91E-07 |
| sp\|P14060\|3BHS1_HUMAN | 42492.10918 | 1.66 | 0.874 | 0.0006481 |
| sp\|P21741\|MK_HUMAN | 16127.22353 | 1.66 | 1.061 | 0.008968 |
| sp\|Q9NUQ6\|SPS2L_HUMAN | 62185.91311 | 1.66 | 0.287 | 4.03E-13 |
| sp\|Q96EV2\|RBM33_HUMAN | 130172.849 | 1.66 | 0.708 | 2.08E-06 |
| sp\|P02545\|LMNA_HUMAN | 74361.80498 | 1.66 | 0.64 | 4.17E-07 |
| sp\|P00325\|ADH1B_HUMAN | 40665.90789 | 1.65 | 0.66 | 6.67E-05 |
| sp\|P16401\|H15_HUMAN | 22548.45774 | 1.65 | 0.714 | 0.0006315 |
| sp\|P01011\|AACT_HUMAN | 47773.59027 | 1.65 | 0.79 | 0.001254 |
| sp\|Q96CP2\|FWCH2_HUMAN | 14650.57666 | 1.65 | 0.652 | 3.20E-06 |
| sp\|Q0VF96\|CGNL1_HUMAN | 149540.7993 | 1.65 | 0.558 | 1.30E-06 |
| sp\|Q8WTS6\|SETD7_HUMAN | 41019.23479 | 1.65 | 0.411 | 5.24E-09 |
| sp\|Q86W92\|LIPB1_HUMAN | 114504.6092 | 1.65 | 0.386 | 8.87E-10 |
| sp\|Q09666\|AHNK_HUMAN | 629194.5684 | 1.65 | 0.346 | 2.77E-11 |
| sp\|A0A0G2JS06\|LV539_HUMAN | 13538.72825 | 1.65 | 0.481 | 3.85E-08 |
| sp\|Q15121\|PEA15_HUMAN | 15069.79821 | 1.65 | 0.242 | 5.64E-15 |
| sp\|Q13813\|SPTN1_HUMAN | 285144.4999 | 1.65 | 0.388 | 1.83E-10 |
| sp\|Q96AB6\|NTAN1_HUMAN | 34922.71167 | 1.65 | 0.203 | 2.20E-16 |
| sp\|Q8IWC1\|MA7D3_HUMAN | 98692.31919 | 1.65 | 0.449 | 7.98E-09 |
| sp\|Q01105\|SET_HUMAN | 33450.69491 | 1.64 | 0.371 | 1.41E-10 |
| sp\|P20962\|PTMS_HUMAN | 11505.17723 | 1.64 | 0.296 | 1.67E-12 |
| sp\|Q16514\|TAF12_HUMAN | 18009.24314 | 1.64 | 0.62 | 2.58E-05 |
| sp\|O15540\|FABP7_HUMAN | 14975.50136 | 1.64 | 0.612 | 2.97E-05 |
| sp\|P02679\|FIBG_HUMAN | 52088.09243 | 1.64 | 1.215 | 0.01557 |
| sp\|Q9NQ48\|LZTL1_HUMAN | 34610.11946 | 1.64 | 0.379 | 4.68E-09 |
| sp\|Q07507\|DERM_HUMAN | 24541.00394 | 1.64 | 0.891 | 0.000406 |
| sp\|Q14934\|NFAC4_HUMAN | 96112.61744 | 1.64 | 0.323 | 1.25E-11 |
| sp\|Q96CV9\|OPTN_HUMAN | 66262.31332 | 1.64 | 0.245 | 1.83E-14 |
| sp\|Q99757\|THIOM_HUMAN | 18524.68235 | 1.64 | 0.749 | 0.001497 |
| sp\|P01834\|IGKC_HUMAN | 11910.82213 | 1.64 | 0.442 | 1.28E-08 |
| sp\|Q9C0D6\|FHDC1_HUMAN | 125635.7815 | 1.63 | 0.473 | 2.27E-08 |
| sp\|Q92791\|SC65_HUMAN | 50844.38955 | 1.63 | 0.394 | 3.54E-09 |
| sp\|Q86YR7\|MF2L2_HUMAN | 128149.1365 | 1.63 | 0.732 | 9.56E-05 |
| sp\|Q00688\|FKBP3_HUMAN | 25200.32787 | 1.63 | 0.275 | 2.36E-13 |
| sp\|Q96AY3\|FKB10_HUMAN | 64699.36852 | 1.63 | 0.453 | 4.64E-08 |
| sp\|Q8NBK3\|SUMF1_HUMAN | 41139.76486 | 1.63 | 0.443 | 2.29E-09 |
| sp\|Q6NY19\|KANK3_HUMAN | 88980.63946 | 1.63 | 0.247 | 1.35E-14 |
| sp\|O75822\|EIF3J_HUMAN | 29140.57571 | 1.63 | 0.312 | 1.10E-11 |
| sp\|P31483\|TIA1_HUMAN | 43259.70389 | 1.63 | 0.322 | 1.65E-11 |
| sp\|A0A075B6H9\|LV469_HUMAN | 12918.25787 | 1.63 | 0.576 | 5.16E-06 |
| sp\|P02794\|FRIH_HUMAN | 21365.34547 | 1.63 | 0.67 | 3.72E-06 |
| sp\|P06858\|LIPL_HUMAN | 53681.10462 | 1.63 | 0.342 | 6.43E-11 |
| sp\|Q8N0X7\|SPART_HUMAN | 73339.99369 | 1.63 | 0.295 | 1.80E-12 |
| sp\|Q9H668\|STN1_HUMAN | 42416.65771 | 1.63 | 0.379 | 1.36E-09 |
| sp\|B7ZAP0\|RBG10_HUMAN | 29229.92327 | 1.63 | 0.314 | 2.23E-12 |
| sp\|Q14696\|MESD_HUMAN | 26213.35823 | 1.63 | 0.355 | 1.20E-10 |
| sp\|Q9NUP1\|BL1S4_HUMAN | 23489.51867 | 1.62 | 0.255 | 2.34E-13 |
| sp\|Q9Y3C1\|NOP16_HUMAN | 21214.11686 | 1.62 | 0.443 | 9.13E-08 |
| sp\|P09668\|CATH_HUMAN | 38035.28367 | 1.62 | 0.577 | 1.65E-05 |
| sp\|Q8N474\|SFRP1_HUMAN | 36256.38681 | 1.62 | 0.46 | 6.12E-08 |
| sp\|Q12888\|TP53B_HUMAN | 215477.4503 | 1.62 | 0.118 | 2.20E-16 |
| sp\|P40424\|PBX1_HUMAN | 46863.86971 | 1.62 | 0.392 | 1.82E-09 |
| sp\|O75828\|CBR3_HUMAN | 31211.85925 | 1.62 | 0.293 | 2.69E-12 |
| sp\|Q2T9J0\|TYSD1_HUMAN | 60279.69392 | 1.62 | 0.464 | 2.91E-08 |
| sp\|P61218\|RPAB2_HUMAN | 14508.00196 | 1.62 | 0.259 | 1.89E-13 |
| sp\|Q00013\|EM55_HUMAN | 52473.72202 | 1.62 | 0.277 | 7.31E-13 |
| sp\|Q8WUH1\|CHUR_HUMAN | 16652.78811 | 1.62 | 0.467 | 6.73E-08 |
| sp\|P16671\|CD36_HUMAN | 53571.46078 | 1.62 | 0.453 | 3.83E-08 |
| sp\|O95503\|CBX6_HUMAN | 44081.34475 | 1.62 | 0.385 | 4.92E-09 |
| sp\|Q9H8L6\|MMRN2_HUMAN | 105010.1271 | 1.62 | 0.589 | 1.98E-07 |
| sp\|Q86WU2\|LDHD_HUMAN | 55673.02338 | 1.61 | 1.094 | 0.03004 |
| sp\|Q9BYV8\|CEP41_HUMAN | 41552.94966 | 1.61 | 0.243 | 4.58E-14 |
| sp\|Q9NZ63\|TLS1_HUMAN | 33706.15556 | 1.61 | 0.403 | 3.93E-09 |
| sp\|Q5EBL4\|RIPL1_HUMAN | 47118.0099 | 1.61 | 0.268 | 2.34E-12 |
| sp\|Q96RK0\|CIC_HUMAN | 164271.3105 | 1.61 | 0.714 | 7.20E-06 |
| sp\|Q9UBR2\|CATZ_HUMAN | 34512.46869 | 1.61 | 0.402 | 2.22E-09 |
| sp\|P31949\|S10AB_HUMAN | 11828.85746 | 1.61 | 0.279 | 2.24E-12 |
| sp\|P54727\|RD23B_HUMAN | 43183.63029 | 1.61 | 0.185 | 2.20E-16 |
| sp\|Q14249\|NUCG_HUMAN | 32753.07993 | 1.61 | 0.524 | 1.61E-07 |
| sp\|Q15742\|NAB2_HUMAN | 56826.14752 | 1.61 | 0.253 | 8.21E-14 |
| sp\|P98179\|RBM3_HUMAN | 17141.9767 | 1.61 | 0.214 | 5.71E-15 |
| sp\|P09132\|SRP19_HUMAN | 16355.50685 | 1.6 | 0.47 | 1.07E-07 |
| sp\|Q8IXK0\|PHC2_HUMAN | 91323.00078 | 1.6 | 0.431 | 7.89E-09 |
| sp\|Q9UHB7\|AFF4_HUMAN | 127762.8979 | 1.6 | 0.242 | 1.19E-13 |
| sp\|P02144\|MYG_HUMAN | 17211.96569 | 1.6 | 0.752 | 0.0003446 |
| sp\|Q86VS8\|HOOK3_HUMAN | 83455.57631 | 1.6 | 0.255 | 6.78E-13 |
| sp\|P0DP23\|CALM1_HUMAN | 16808.82375 | 1.6 | 0.31 | 9.10E-12 |
| sp\|P15927\|RFA2_HUMAN | 29324.48316 | 1.6 | 0.244 | 1.79E-13 |
| sp\|P28906\|CD34_HUMAN | 41072.53372 | 1.6 | 0.67 | 4.37E-06 |
| sp\|P35527\|K1C9_HUMAN | 62236.89002 | 1.6 | 0.598 | 8.95E-06 |
| sp\|P61952\|GBG11_HUMAN | 8571.427793 | 1.6 | 0.428 | 6.84E-09 |
| sp\|P42766\|RL35_HUMAN | 14524.54969 | 1.6 | 0.413 | 8.37E-09 |
| sp\|O00115\|DNS2A_HUMAN | 40050.72737 | 1.6 | 0.351 | 1.50E-10 |
| sp\|Q8WUH6\|TM263_HUMAN | 11723.09038 | 1.6 | 0.869 | 0.004604 |
| sp\|P02746\|C1QB_HUMAN | 26914.56379 | 1.6 | 0.683 | 0.000125 |
| sp\|P0DOX8\|IGL1_HUMAN | 23083.17961 | 1.59 | 0.399 | 6.96E-09 |
| sp\|Q95IE3\|2B1C_HUMAN | 30183.18606 | 1.59 | 0.632 | 4.60E-05 |
| sp\|P63165\|SUMO1_HUMAN | 11588.71641 | 1.59 | 0.466 | 2.95E-08 |
| sp\|O75475\|PSIP1_HUMAN | 60162.71206 | 1.59 | 0.246 | 3.87E-13 |
| sp\|P49207\|RL34_HUMAN | 13494.54755 | 1.59 | 0.335 | 3.48E-10 |
| sp\|Q9UNH7\|SNX6_HUMAN | 46886.85277 | 1.59 | 0.203 | 2.16E-15 |
| sp\|Q8IYB3\|SRRM1_HUMAN | 102313.1396 | 1.59 | 0.407 | 9.73E-09 |
| sp\|P08519\|APOA_HUMAN | 514719.443 | 1.59 | 0.877 | 0.006655 |
| sp\|Q96R72\|OR4K3_HUMAN | 35879.64505 | 1.59 | 0.288 | 4.00E-11 |
| sp\|P07910\|HNRPC_HUMAN | 33688.54982 | 1.59 | 0.242 | 3.52E-13 |
| sp\|Q6UX71\|PXDC2_HUMAN | 60097.74808 | 1.59 | 0.501 | 5.41E-07 |
| sp\|Q92804\|RBP56_HUMAN | 62003.17767 | 1.59 | 0.493 | 3.46E-07 |
| sp\|Q96GP6\|SREC2_HUMAN | 96866.56512 | 1.58 | 0.545 | 1.57E-06 |
| sp\|P62699\|YPEL5_HUMAN | 14042.91995 | 1.58 | 0.186 | 9.26E-16 |
| sp\|Q13332\|PTPRS_HUMAN | 218141.2741 | 1.58 | 0.449 | 3.36E-08 |
| sp\|Q9BYD6\|RM01_HUMAN | 37095.48114 | 1.58 | 0.426 | 7.21E-08 |
| sp\|Q8TD55\|PKHO2_HUMAN | 53641.21833 | 1.58 | 0.187 | 1.22E-15 |
| sp\|Q9BXL7\|CAR11_HUMAN | 134493.778 | 1.58 | 0.618 | 0.0004619 |
| sp\|P12107\|COBA1_HUMAN | 181562.9094 | 1.58 | 0.539 | 1.61E-05 |
| sp\|Q14103\|HNRPD_HUMAN | 38563.35645 | 1.58 | 0.216 | 1.00E-14 |
| sp\|P49354\|FNTA_HUMAN | 44477.13106 | 1.58 | 0.189 | 3.34E-16 |
| sp\|Q5JTB6\|PLAC9_HUMAN | 10398.27754 | 1.58 | 0.99 | 0.007865 |
| sp\|Q9H6E5\|STPAP_HUMAN | 94967.82945 | 1.58 | 0.397 | 1.55E-08 |
| sp\|P0DOX7\|IGK_HUMAN | 23631.51608 | 1.58 | 0.37 | 5.55E-09 |
| sp\|P35269\|T2FA_HUMAN | 58244.07497 | 1.58 | 0.396 | 1.61E-08 |
| sp\|P20810\|ICAL_HUMAN | 76907.4072 | 1.58 | 0.276 | 2.29E-12 |
| sp\|Q8TBN0\|R3GEF_HUMAN | 43161.78706 | 1.57 | 0.287 | 4.47E-11 |
| sp\|Q15642\|CIP4_HUMAN | 68520.2756 | 1.57 | 0.148 | 2.20E-16 |
| sp\|P62253\|UB2G1_HUMAN | 19592.91995 | 1.57 | 0.384 | 2.44E-09 |
| sp\|Q14005\|IL16_HUMAN | 142958.3327 | 1.57 | 0.525 | 1.05E-06 |
| sp\|Q15417\|CNN3_HUMAN | 36543.83819 | 1.57 | 0.586 | 1.27E-05 |
| sp\|Q13501\|SQSTM_HUMAN | 48437.15866 | 1.57 | 0.45 | 2.83E-07 |
| sp\|O43399\|TPD54_HUMAN | 22263.28191 | 1.57 | 0.289 | 5.02E-12 |
| sp\|P62917\|RL8_HUMAN | 28217.36407 | 1.57 | 0.243 | 4.00E-13 |
| sp\|P16189\|1A31_HUMAN | 41245.30892 | 1.57 | 0.35 | 9.39E-10 |
| sp\|Q13185\|CBX3_HUMAN | 20951.40315 | 1.57 | 0.455 | 1.40E-07 |
| sp\|P84243\|H33_HUMAN | 15357.51373 | 1.57 | 0.438 | 1.79E-07 |
| sp\|Q9Y5Z4\|HEBP2_HUMAN | 22843.17859 | 1.57 | 0.345 | 1.79E-09 |
| sp\|Q9Y520\|PRC2C_HUMAN | 317327.5531 | 1.57 | 0.203 | 7.88E-15 |
| sp\|Q14767\|LTBP2_HUMAN | 204028.3236 | 1.57 | 0.544 | 1.52E-05 |
| sp\|P14317\|HCLS1_HUMAN | 54077.00705 | 1.56 | 0.48 | 3.75E-07 |
| sp\|Q96P44\|COLA1_HUMAN | 100086.9823 | 1.56 | 0.587 | 8.19E-06 |
| sp\|P46937\|YAP1_HUMAN | 54466.48738 | 1.56 | 0.272 | 1.26E-11 |
| sp\|Q8NEY8\|PPHLN_HUMAN | 52801.11954 | 1.56 | 0.182 | 2.49E-16 |
| sp\|P42765\|THIM_HUMAN | 42335.80702 | 1.56 | 0.283 | 2.77E-11 |
| sp\|Q8NI22\|MCFD2_HUMAN | 16475.83025 | 1.56 | 0.37 | 2.01E-08 |
| sp\|Q9UPN4\|CP131_HUMAN | 122513.2991 | 1.56 | 0.386 | 4.18E-09 |
| sp\|Q16695\|H31T_HUMAN | 15594.54531 | 1.56 | 0.786 | 0.001616 |
| sp\|Q8N511\|TM199_HUMAN | 23211.57059 | 1.56 | 0.575 | 1.06E-05 |
| sp\|Q9Y2B2\|PIGL_HUMAN | 28779.9105 | 1.56 | 0.199 | 1.13E-14 |
| sp\|Q15050\|RRS1_HUMAN | 41207.1776 | 1.56 | 0.539 | 2.23E-05 |
| sp\|P31947\|1433S_HUMAN | 27852.72578 | 1.56 | 0.759 | 0.007427 |
| sp\|O75635\|SPB7_HUMAN | 43144.59143 | 1.56 | 0.817 | 0.003002 |
| sp\|O43290\|SNUT1_HUMAN | 90353.32048 | 1.56 | 0.357 | 2.50E-09 |
| sp\|P52298\|NCBP2_HUMAN | 18142.83876 | 1.56 | 0.305 | 5.76E-11 |
| sp\|Q15811\|ITSN1_HUMAN | 196137.263 | 1.56 | 0.232 | 4.52E-13 |
| sp\|P18859\|ATP5J_HUMAN | 12561.593 | 1.55 | 0.51 | 1.97E-05 |
| sp\|Q96QR8\|PURB_HUMAN | 33373.532 | 1.55 | 0.212 | 4.13E-14 |
| sp\|P09486\|SPRC_HUMAN | 35447.02038 | 1.55 | 0.32 | 5.15E-10 |
| sp\|Q15063\|POSTN_HUMAN | 93864.50068 | 1.55 | 0.445 | 4.39E-06 |
| sp\|P61981\|1433G_HUMAN | 28437.96658 | 1.55 | 0.201 | 1.48E-14 |
| sp\|Q8IVL6\|P3H3_HUMAN | 82565.92131 | 1.55 | 0.459 | 1.42E-06 |
| sp\|Q96BQ5\|CC127_HUMAN | 30968.06259 | 1.55 | 0.407 | 8.91E-08 |
| sp\|P42566\|EPS15_HUMAN | 99204.0168 | 1.55 | 0.103 | 2.20E-16 |
| sp\|Q2TAA2\|IAH1_HUMAN | 28019.20662 | 1.55 | 0.305 | 2.06E-10 |
| sp\|O43852\|CALU_HUMAN | 37179.57522 | 1.55 | 0.211 | 8.46E-14 |
| sp\|Q13442\|HAP28_HUMAN | 20599.61995 | 1.55 | 0.521 | 6.67E-06 |
| sp\|Q9HD47\|MOG1_HUMAN | 20588.42338 | 1.55 | 0.289 | 9.20E-11 |
| sp\|P62328\|TYB4_HUMAN | 5031.505674 | 1.55 | 0.333 | 5.31E-10 |
| sp\|Q4ZHG4\|FNDC1_HUMAN | 205871.2833 | 1.55 | 0.63 | 0.0001943 |
| sp\|Q7Z4V5\|HDGR2_HUMAN | 74425.28355 | 1.55 | 0.293 | 2.39E-10 |
| sp\|P00519\|ABL1_HUMAN | 123576.6259 | 1.54 | 0.49 | 3.10E-06 |
| sp\|A0A0B4J1V0\|HV315_HUMAN | 13070.53226 | 1.54 | 0.573 | 4.44E-05 |
| sp\|Q8TDH9\|BL1S5_HUMAN | 21748.88382 | 1.54 | 0.232 | 4.97E-13 |
| sp\|Q96EP5\|DAZP1_HUMAN | 43566.13115 | 1.54 | 0.234 | 2.49E-13 |
| sp\|Q9UHB6\|LIMA1_HUMAN | 85611.51962 | 1.54 | 0.325 | 4.13E-09 |
| sp\|O15514\|RPB4_HUMAN | 16397.15737 | 1.54 | 0.223 | 1.62E-13 |
| sp\|P07355\|ANXA2_HUMAN | 38789.89006 | 1.54 | 0.314 | 8.05E-11 |
| sp\|Q9NWB6\|ARGL1_HUMAN | 33178.81654 | 1.54 | 0.201 | 6.46E-14 |
| sp\|Q9UM13\|APC10_HUMAN | 21391.78115 | 1.54 | 0.255 | 6.67E-12 |
| sp\|Q7Z4H3\|HDDC2_HUMAN | 23528.70363 | 1.54 | 0.181 | 1.38E-15 |
| sp\|P62750\|RL23A_HUMAN | 17666.12362 | 1.54 | 0.254 | 4.55E-12 |
| sp\|P54687\|BCAT1_HUMAN | 43490.72943 | 1.54 | 0.481 | 6.85E-06 |
| sp\|P26583\|HMGB2_HUMAN | 24171.77968 | 1.54 | 0.471 | 2.42E-06 |
| sp\|O95926\|SYF2_HUMAN | 28743.81866 | 1.54 | 0.519 | 1.17E-05 |
| sp\|O15232\|MATN3_HUMAN | 54361.33733 | 1.54 | 0.391 | 3.61E-08 |
| sp\|Q3KQU3\|MA7D1_HUMAN | 93088.21739 | 1.54 | 0.135 | 2.20E-16 |
| sp\|Q9UK45\|LSM7_HUMAN | 11691.03919 | 1.54 | 0.253 | 6.72E-13 |
| sp\|Q9C0A0\|CNTP4_HUMAN | 147160.2259 | 1.54 | 0.347 | 1.49E-09 |
| sp\|P16949\|STMN1_HUMAN | 17273.9431 | 1.54 | 0.393 | 8.79E-08 |
| sp\|P02675\|FIBB_HUMAN | 56558.50782 | 1.54 | 1.137 | 0.04943 |
| sp\|Q9NVZ3\|NECP2_HUMAN | 28417.33386 | 1.54 | 0.256 | 1.80E-11 |
| sp\|Q9UPT8\|ZC3H4_HUMAN | 140778.6966 | 1.54 | 0.337 | 8.26E-10 |
| sp\|Q14247\|SRC8_HUMAN | 61701.58554 | 1.54 | 0.612 | 1.92E-05 |
| sp\|Q5QJ74\|TBCEL_HUMAN | 48659.61371 | 1.54 | 0.247 | 3.26E-12 |
| sp\|Q08170\|SRSF4_HUMAN | 56741.36314 | 1.54 | 0.164 | 3.02E-16 |
| sp\|P16333\|NCK1_HUMAN | 43047.48815 | 1.54 | 0.075 | 2.20E-16 |
| sp\|Q8WXI9\|P66B_HUMAN | 65544.11355 | 1.54 | 0.406 | 4.64E-08 |
| sp\|Q06547\|GABP1_HUMAN | 42552.61405 | 1.54 | 0.207 | 5.77E-14 |
| sp\|Q969G5\|CAVN3_HUMAN | 27666.52805 | 1.54 | 0.386 | 1.33E-08 |
| sp\|Q8TEH3\|DEN1A_HUMAN | 111117.1229 | 1.53 | 0.413 | 1.33E-07 |
| sp\|O60331\|PI51C_HUMAN | 73481.58785 | 1.53 | 0.486 | 2.11E-06 |
| sp\|P02549\|SPTA1_HUMAN | 281021.3365 | 1.53 | 0.28 | 2.48E-11 |
| sp\|O00422\|SAP18_HUMAN | 17588.97887 | 1.53 | 0.348 | 4.43E-09 |
| sp\|O95865\|DDAH2_HUMAN | 29892.5524 | 1.53 | 0.36 | 1.14E-09 |
| sp\|Q13188\|STK3_HUMAN | 56532.07759 | 1.53 | 0.285 | 4.08E-11 |
| sp\|P67936\|TPM4_HUMAN | 28600.5234 | 1.53 | 0.417 | 1.20E-07 |
| sp\|P13645\|K1C10_HUMAN | 59001.76894 | 1.53 | 0.538 | 5.93E-05 |
| sp\|Q9H492\|MLP3A_HUMAN | 14302.4006 | 1.53 | 0.629 | 0.0001651 |
| sp\|O60687\|SRPX2_HUMAN | 54174.17567 | 1.53 | 0.35 | 1.14E-08 |
| sp\|P04264\|K2C1_HUMAN | 66152.05474 | 1.53 | 0.648 | 6.73E-05 |
| sp\|P01700\|LV147_HUMAN | 12428.95937 | 1.53 | 0.306 | 1.60E-10 |
| sp\|O60232\|SSA27_HUMAN | 21898.84748 | 1.53 | 0.361 | 1.39E-08 |
| sp\|O94813\|SLIT2_HUMAN | 175785.2847 | 1.53 | 0.44 | 9.83E-07 |
| sp\|Q13574\|DGKZ_HUMAN | 125685.9165 | 1.53 | 0.641 | 0.0001544 |
| sp\|Q15293\|RCN1_HUMAN | 38848.15404 | 1.53 | 0.226 | 1.91E-13 |
| sp\|P07108\|ACBP_HUMAN | 10020.01039 | 1.53 | 0.369 | 1.74E-07 |
| sp\|Q9HCU8\|DPOD4_HUMAN | 12578.34191 | 1.53 | 0.304 | 1.98E-10 |
| sp\|O94875\|SRBS2_HUMAN | 125152.5451 | 1.53 | 0.835 | 0.001769 |
| sp\|P62070\|RRAS2_HUMAN | 23594.79886 | 1.53 | 0.417 | 1.37E-07 |
| sp\|O14979\|HNRDL_HUMAN | 46562.14074 | 1.53 | 0.255 | 3.59E-11 |
| sp\|Q15370\|ELOB_HUMAN | 13220.58356 | 1.53 | 0.18 | 2.98E-15 |
| sp\|Q15075\|EEA1_HUMAN | 163318.5351 | 1.53 | 0.319 | 1.34E-10 |
| sp\|Q9BUH8\|BEGIN_HUMAN | 65371.99919 | 1.53 | 1.047 | 0.03733 |
| sp\|Q9Y3E1\|HDGR3_HUMAN | 22645.02076 | 1.53 | 0.447 | 4.06E-07 |
| sp\|Q8NDX5\|PHC3_HUMAN | 106875.8494 | 1.53 | 0.19 | 5.09E-15 |
| sp\|Q13111\|CAF1A_HUMAN | 108023.0119 | 1.53 | 0.253 | 5.61E-12 |
| sp\|O75886\|STAM2_HUMAN | 58508.8613 | 1.53 | 0.214 | 7.47E-14 |
| sp\|Q969H8\|MYDGF_HUMAN | 18879.34609 | 1.52 | 0.202 | 1.26E-13 |
| sp\|Q9UQ84\|EXO1_HUMAN | 95167.01298 | 1.52 | 0.205 | 3.48E-14 |
| sp\|P02671\|FIBA_HUMAN | 95637.68349 | 1.52 | 1.056 | 0.0343 |
| sp\|Q9NW68\|BSDC1_HUMAN | 47401.01921 | 1.52 | 0.189 | 1.37E-14 |
| sp\|O60496\|DOK2_HUMAN | 45731.85935 | 1.52 | 0.322 | 5.87E-09 |
| sp\|Q96SB3\|NEB2_HUMAN | 89290.75267 | 1.52 | 0.191 | 3.33E-14 |
| sp\|L0R819\|ASURF_HUMAN | 11224.8933 | 1.52 | 0.268 | 6.28E-11 |
| sp\|Q86W25\|NAL13_HUMAN | 121126.4642 | 1.52 | 0.679 | 0.0005786 |
| sp\|O95684\|FR1OP_HUMAN | 43134.96038 | 1.52 | 0.378 | 3.34E-08 |
| sp\|Q96I15\|SCLY_HUMAN | 48385.70579 | 1.52 | 0.207 | 4.69E-14 |
| sp\|Q9H6S1\|AZI2_HUMAN | 45401.89868 | 1.52 | 0.271 | 2.46E-11 |
| sp\|Q9NPA8\|ENY2_HUMAN | 11617.34564 | 1.52 | 0.543 | 1.30E-05 |
| sp\|Q9P2E9\|RRBP1_HUMAN | 152745.8206 | 1.52 | 0.331 | 5.41E-09 |
| sp\|Q96D15\|RCN3_HUMAN | 37451.98567 | 1.52 | 0.361 | 1.75E-08 |
| sp\|Q15654\|TRIP6_HUMAN | 51720.0092 | 1.52 | 0.274 | 2.22E-11 |
| sp\|Q9UK76\|JUPI1_HUMAN | 15986.90071 | 1.52 | 0.612 | 0.0009853 |
| sp\|P60983\|GMFB_HUMAN | 16855.5396 | 1.51 | 0.225 | 5.02E-13 |
| sp\|Q6WCQ1\|MPRIP_HUMAN | 117241.7661 | 1.51 | 0.195 | 1.61E-14 |
| sp\|P52566\|GDIR2_HUMAN | 23012.61569 | 1.51 | 0.309 | 1.98E-09 |
| sp\|P23434\|GCSH_HUMAN | 19082.69394 | 1.51 | 0.447 | 4.01E-06 |
| sp\|P06753\|TPM3_HUMAN | 32968.78844 | 1.51 | 0.107 | 2.20E-16 |
| sp\|Q8WVJ2\|NUDC2_HUMAN | 17817.79738 | 1.51 | 0.389 | 5.80E-08 |
| sp\|P04433\|KV311_HUMAN | 12663.3363 | 1.51 | 0.464 | 6.62E-06 |
| sp\|Q92871\|PMM1_HUMAN | 30052.15279 | 1.51 | 0.303 | 1.42E-09 |
| sp\|O14562\|UBFD1_HUMAN | 33456.88145 | 1.51 | 0.288 | 5.11E-10 |
| sp\|P09382\|LEG1_HUMAN | 15030.31755 | 1.51 | 0.382 | 1.32E-07 |
| sp\|Q9ULI1\|NWD2_HUMAN | 200173.2929 | 1.51 | 0.352 | 4.66E-08 |
| sp\|Q9NYL9\|TMOD3_HUMAN | 39723.35381 | 1.51 | 0.193 | 1.71E-14 |
| sp\|O75718\|CRTAP_HUMAN | 47141.16308 | 1.51 | 0.409 | 5.53E-07 |
| sp\|P78559\|MAP1A_HUMAN | 306762.6911 | 1.51 | 0.3 | 2.40E-10 |
| sp\|Q6NXS1\|IPP2B_HUMAN | 23131.05729 | 1.51 | 0.162 | 6.21E-16 |
| sp\|Q03252\|LMNB2_HUMAN | 70001.73037 | 1.51 | 0.392 | 2.34E-08 |
| sp\|Q96A19\|C102A_HUMAN | 62768.12344 | 1.51 | 0.374 | 1.13E-08 |
| sp\|Q9NWM8\|FKB14_HUMAN | 24252.47084 | 1.51 | 0.542 | 4.82E-05 |
| sp\|Q14515\|SPRL1_HUMAN | 75998.57323 | 1.51 | 0.556 | 1.65E-05 |
| sp\|O43353\|RIPK2_HUMAN | 61708.3764 | 1.51 | 0.516 | 2.00E-06 |
| sp\|Q9UMX5\|NENF_HUMAN | 18826.78277 | 1.51 | 0.363 | 8.36E-08 |
| sp\|Q6EMK4\|VASN_HUMAN | 72732.91426 | 1.51 | 0.475 | 1.09E-06 |
| sp\|P09234\|RU1C_HUMAN | 17534.29152 | 1.51 | 0.326 | 2.16E-09 |
| sp\|Q9NR99\|MXRA5_HUMAN | 314046.7365 | 1.51 | 0.357 | 1.18E-07 |
| sp\|Q9Y5T5\|UBP16_HUMAN | 94918.41389 | 1.51 | 0.343 | 2.21E-08 |
| sp\|Q96BJ3\|AIDA_HUMAN | 35154.34698 | 1.51 | 0.268 | 3.63E-11 |
