# Supplementary material for: Apolipoprotein C‐II induces EMT to promote gastric cancer peritoneal metastasis via PI3K/AKT/mTOR pathway
Source: Clin Transl Med. 2021 Aug 9;11(8):e522. doi: 10.1002/ctm2.522 (PMC8351524; doi:10.1002/ctm2.522)
Supplement: Supplementary file 18 — Table S3. Compared with the primary GC, 1050 proteins in the PM tissues were downregulated among these DEPs. [file CTM2-11-e522-s006.docx]

**Table S3. Compared with the primary GC, 1050 proteins in the PM tissues were downregulated among these DEPs.**

| Protein_ID | Mass | Mean_Ratio_  PM-VS-GC | SD_  PM-VS-GC | Pvalue_  PM-VS-GC |
| --- | --- | --- | --- | --- |
| sp\|Q8N5I3\|KCNRG_HUMAN | 31181.14 | 0.54 | 0.092 | 3.69E-15 |
| sp\|Q9HCM4\|E41L5_HUMAN | 82357.11 | 0.62 | 0.209 | 1.17E-08 |
| sp\|P08218\|CEL2B_HUMAN | 29400.26 | 0.5 | 0.296 | 7.15E-07 |
| sp\|P21860\|ERBB3_HUMAN | 151405.6 | 0.62 | 0.247 | 5.81E-07 |
| sp\|Q9H9C1\|SPE39_HUMAN | 57180.32 | 0.6 | 0.055 | 2.20E-16 |
| sp\|O14874\|BCKD_HUMAN | 46598.27 | 0.64 | 0.205 | 3.24E-08 |
| sp\|P46977\|STT3A_HUMAN | 81086.07 | 0.51 | 0.129 | 9.16E-13 |
| sp\|P54803\|GALC_HUMAN | 77281.84 | 0.57 | 0.207 | 1.69E-08 |
| sp\|P19021\|AMD_HUMAN | 109100.6 | 0.66 | 0.225 | 3.92E-07 |
| sp\|Q7L523\|RRAGA_HUMAN | 36923.69 | 0.6 | 0.04 | 2.20E-16 |
| sp\|Q6DD88\|ATLA3_HUMAN | 60941.58 | 0.58 | 0.058 | 2.20E-16 |
| sp\|O43847\|NRDC_HUMAN | 132625.9 | 0.55 | 0.119 | 1.79E-13 |
| sp\|P49326\|FMO5_HUMAN | 60620.07 | 0.5 | 0.094 | 3.73E-15 |
| sp\|Q5T9L3\|WLS_HUMAN | 62877.93 | 0.49 | 0.098 | 9.57E-16 |
| sp\|Q7Z4Q2\|HEAT3_HUMAN | 75771.45 | 0.46 | 0.143 | 6.83E-12 |
| sp\|P41235\|HNF4A_HUMAN | 53474.05 | 0.6 | 0.238 | 7.07E-08 |
| sp\|Q9H4H8\|FA83D_HUMAN | 64822.5 | 0.55 | 0.143 | 1.21E-11 |
| sp\|Q96GG9\|DCNL1_HUMAN | 30315.13 | 0.65 | 0.17 | 3.01E-08 |
| sp\|P17213\|BPI_HUMAN | 54075.14 | 0.5 | 0.313 | 3.25E-06 |
| sp\|Q7Z460\|CLAP1_HUMAN | 170354.5 | 0.62 | 0.069 | 2.20E-16 |
| sp\|P57088\|TMM33_HUMAN | 28283.93 | 0.61 | 0.127 | 9.98E-11 |
| sp\|Q9NRX1\|PNO1_HUMAN | 28059.91 | 0.64 | 0.192 | 2.61E-08 |
| sp\|P46734\|MP2K3_HUMAN | 39617.05 | 0.57 | 0.13 | 1.97E-12 |
| sp\|Q9UJ70\|NAGK_HUMAN | 37676.04 | 0.65 | 0.127 | 4.18E-11 |
| sp\|Q11206\|SIA4C_HUMAN | 38231.12 | 0.6 | 0.233 | 9.76E-07 |
| sp\|P22894\|MMP8_HUMAN | 53531.55 | 0.55 | 0.34 | 2.08E-06 |
| sp\|P54646\|AAPK2_HUMAN | 62831.78 | 0.66 | 0.157 | 2.12E-09 |
| sp\|Q9Y6Q1\|CAN6_HUMAN | 75365.94 | 0.49 | 0.167 | 2.57E-10 |
| sp\|P29536\|LMOD1_HUMAN | 67142 | 0.41 | 0.215 | 3.29E-10 |
| sp\|P30679\|GNA15_HUMAN | 43978.33 | 0.65 | 0.132 | 8.96E-11 |
| sp\|O95352\|ATG7_HUMAN | 79031.74 | 0.6 | 0.076 | 2.20E-16 |
| sp\|Q9BUR5\|MIC26_HUMAN | 22366.56 | 0.62 | 0.151 | 3.86E-10 |
| sp\|Q8WVQ1\|CANT1_HUMAN | 44907.72 | 0.58 | 0.102 | 5.85E-14 |
| sp\|P10301\|RRAS_HUMAN | 23618.94 | 0.64 | 0.334 | 4.37E-06 |
| sp\|Q9BTW9\|TBCD_HUMAN | 134264.9 | 0.58 | 0.091 | 2.60E-15 |
| sp\|O75110\|ATP9A_HUMAN | 119970.7 | 0.6 | 0.135 | 1.81E-11 |
| sp\|P55786\|PSA_HUMAN | 103877 | 0.61 | 0.069 | 2.20E-16 |
| sp\|P54136\|SYRC_HUMAN | 76111.15 | 0.62 | 0.117 | 1.86E-12 |
| sp\|Q8N335\|GPD1L_HUMAN | 39003.15 | 0.66 | 0.154 | 1.06E-08 |
| sp\|Q8NFL0\|B3GN7_HUMAN | 46452.86 | 0.55 | 0.216 | 7.73E-08 |
| sp\|Q10472\|GALT1_HUMAN | 65071.75 | 0.55 | 0.088 | 7.66E-16 |
| sp\|Q8WXE0\|CSKI2_HUMAN | 127257.6 | 0.47 | 0.073 | 2.20E-16 |
| sp\|P22748\|CAH4_HUMAN | 35276.99 | 0.65 | 0.13 | 2.21E-11 |
| sp\|Q3MIR4\|CC50B_HUMAN | 39354.51 | 0.65 | 0.172 | 1.02E-08 |
| sp\|P23526\|SAHH_HUMAN | 48237.41 | 0.62 | 0.111 | 4.41E-13 |
| sp\|P14923\|PLAK_HUMAN | 82416 | 0.6 | 0.248 | 2.18E-07 |
| sp\|Q92828\|COR2A_HUMAN | 60220.51 | 0.49 | 0.255 | 1.45E-08 |
| sp\|Q2M389\|WASC4_HUMAN | 137324.6 | 0.64 | 0.066 | 2.20E-16 |
| sp\|O76038\|SEGN_HUMAN | 32172.16 | 0.48 | 0.258 | 7.87E-08 |
| sp\|Q9H0U4\|RAB1B_HUMAN | 22310.28 | 0.61 | 0.105 | 1.49E-13 |
| sp\|Q9BSR8\|YIPF4_HUMAN | 27104.32 | 0.56 | 0.112 | 8.86E-14 |
| sp\|Q3SXM5\|HSDL1_HUMAN | 37359.26 | 0.61 | 0.103 | 1.90E-13 |
| sp\|O43490\|PROM1_HUMAN | 98376.53 | 0.46 | 0.246 | 7.22E-09 |
| sp\|P57735\|RAB25_HUMAN | 23577.03 | 0.57 | 0.203 | 4.04E-09 |
| sp\|Q16706\|MA2A1_HUMAN | 131723.6 | 0.53 | 0.112 | 3.09E-14 |
| sp\|Q9NYA4\|MTMR4_HUMAN | 136271.8 | 0.53 | 0.09 | 8.20E-16 |
| sp\|O60518\|RNBP6_HUMAN | 126154.9 | 0.62 | 0.146 | 9.65E-11 |
| sp\|P42126\|ECI1_HUMAN | 33062.33 | 0.64 | 0.203 | 1.17E-07 |
| sp\|A1A5B4\|ANO9_HUMAN | 91168.97 | 0.5 | 0.221 | 2.97E-09 |
| sp\|Q9UBC5\|MYO1A_HUMAN | 119220.1 | 0.46 | 0.177 | 6.42E-11 |
| sp\|P04839\|CY24B_HUMAN | 66187.92 | 0.54 | 0.192 | 1.82E-09 |
| sp\|Q6NUK1\|SCMC1_HUMAN | 53530.42 | 0.59 | 0.16 | 2.66E-10 |
| sp\|Q96QI5\|HS3S6_HUMAN | 37486.62 | 0.57 | 0.179 | 2.15E-10 |
| sp\|Q05D32\|CTSL2_HUMAN | 53119.2 | 0.43 | 0.094 | 2.80E-16 |
| sp\|Q9UGC7\|RF1ML_HUMAN | 43668.81 | 0.59 | 0.164 | 1.32E-08 |
| sp\|Q02978\|M2OM_HUMAN | 34192.91 | 0.47 | 0.132 | 2.19E-13 |
| sp\|P50148\|GNAQ_HUMAN | 42382.41 | 0.62 | 0.143 | 1.68E-10 |
| sp\|O76024\|WFS1_HUMAN | 101349.4 | 0.6 | 0.177 | 3.82E-09 |
| sp\|Q9H0V1\|TM168_HUMAN | 80938.74 | 0.65 | 0.075 | 1.01E-15 |
| sp\|O60832\|DKC1_HUMAN | 58075.95 | 0.64 | 0.098 | 1.11E-13 |
| sp\|Q9HC62\|SENP2_HUMAN | 68591.97 | 0.6 | 0.24 | 3.88E-07 |
| sp\|Q9H5V8\|CDCP1_HUMAN | 94279.89 | 0.54 | 0.188 | 1.91E-08 |
| sp\|Q6ZNC4\|ZN704_HUMAN | 45799.64 | 0.52 | 0.112 | 1.15E-13 |
| sp\|Q9BT73\|PSMG3_HUMAN | 13249.12 | 0.52 | 0.05 | 2.20E-16 |
| sp\|O15397\|IPO8_HUMAN | 120926.7 | 0.57 | 0.092 | 3.47E-15 |
| sp\|Q9BZJ0\|CRNL1_HUMAN | 100884.3 | 0.65 | 0.145 | 7.16E-10 |
| sp\|P57105\|SYJ2B_HUMAN | 15900.15 | 0.62 | 0.112 | 1.47E-12 |
| sp\|Q8TEM1\|PO210_HUMAN | 205877 | 0.65 | 0.102 | 2.74E-13 |
| sp\|Q8IZA0\|K319L_HUMAN | 116651 | 0.43 | 0.173 | 7.49E-12 |
| sp\|Q6GPI1\|CTRB2_HUMAN | 28514.34 | 0.61 | 0.238 | 3.67E-07 |
| sp\|P17927\|CR1_HUMAN | 230455.5 | 0.66 | 0.371 | 4.18E-05 |
| sp\|Q96K19\|RN170_HUMAN | 30233.35 | 0.63 | 0.169 | 5.82E-09 |
| sp\|P23634\|AT2B4_HUMAN | 139012.2 | 0.64 | 0.214 | 6.45E-08 |
| sp\|O60575\|ISK4_HUMAN | 9771.877 | 0.43 | 0.278 | 1.76E-07 |
| sp\|O43772\|MCAT_HUMAN | 33246.22 | 0.53 | 0.103 | 2.92E-14 |
| sp\|P55061\|BI1_HUMAN | 26786.99 | 0.28 | 0.086 | 2.20E-16 |
| sp\|Q9H6E4\|CC134_HUMAN | 26582.97 | 0.64 | 0.297 | 2.99E-06 |
| sp\|Q96AM1\|MRGRF_HUMAN | 39154.08 | 0.4 | 0.228 | 8.72E-10 |
| sp\|P13987\|CD59_HUMAN | 14777.02 | 0.55 | 0.12 | 2.23E-13 |
| sp\|O14734\|ACOT8_HUMAN | 36329.19 | 0.66 | 0.2 | 4.17E-07 |
| sp\|Q7Z2K6\|ERMP1_HUMAN | 101004.7 | 0.52 | 0.184 | 9.29E-10 |
| sp\|Q10469\|MGAT2_HUMAN | 52183.57 | 0.45 | 0.204 | 1.36E-07 |
| sp\|Q7L5L3\|GDPD3_HUMAN | 36953.87 | 0.55 | 0.305 | 1.73E-06 |
| sp\|Q96P70\|IPO9_HUMAN | 116840.1 | 0.64 | 0.082 | 2.15E-15 |
| sp\|O75964\|ATP5L_HUMAN | 11403.23 | 0.45 | 0.146 | 1.95E-12 |
| sp\|Q15041\|AR6P1_HUMAN | 23500.49 | 0.39 | 0.106 | 1.75E-15 |
| sp\|P56470\|LEG4_HUMAN | 36014.22 | 0.41 | 0.16 | 2.13E-11 |
| sp\|O94832\|MYO1D_HUMAN | 116908.8 | 0.59 | 0.14 | 6.45E-11 |
| sp\|Q9BYZ8\|REG4_HUMAN | 18598.96 | 0.56 | 0.121 | 3.69E-12 |
| sp\|P16615\|AT2A2_HUMAN | 116318.2 | 0.48 | 0.077 | 2.20E-16 |
| sp\|P52292\|IMA1_HUMAN | 58150.07 | 0.64 | 0.34 | 2.21E-05 |
| sp\|Q8IY22\|CMIP_HUMAN | 87682.64 | 0.43 | 0.068 | 2.20E-16 |
| sp\|A0A0C4DH34\|HV428_HUMAN | 13211.69 | 0.47 | 0.187 | 2.65E-10 |
| sp\|Q9Y2K7\|KDM2A_HUMAN | 135141.4 | 0.62 | 0.16 | 2.47E-10 |
| sp\|P61619\|S61A1_HUMAN | 52668.67 | 0.51 | 0.111 | 3.05E-14 |
| sp\|P52788\|SPSY_HUMAN | 41679.95 | 0.63 | 0.129 | 2.13E-11 |
| sp\|Q9ULC4\|MCTS1_HUMAN | 20751.9 | 0.65 | 0.071 | 3.89E-16 |
| sp\|P03886\|NU1M_HUMAN | 35618.99 | 0.44 | 0.151 | 1.32E-11 |
| sp\|P01130\|LDLR_HUMAN | 98887.98 | 0.57 | 0.117 | 2.01E-13 |
| sp\|P63218\|GBG5_HUMAN | 7409.837 | 0.62 | 0.168 | 2.16E-09 |
| sp\|Q04912\|RON_HUMAN | 154692.1 | 0.52 | 0.133 | 3.49E-12 |
| sp\|Q5VYY1\|ANR22_HUMAN | 22215.5 | 0.62 | 0.275 | 3.85E-06 |
| sp\|Q8N5M1\|ATPF2_HUMAN | 32961.98 | 0.57 | 0.105 | 2.96E-14 |
| sp\|O75448\|MED24_HUMAN | 111698.3 | 0.52 | 0.122 | 1.73E-13 |
| sp\|P10721\|KIT_HUMAN | 111144.6 | 0.47 | 0.258 | 5.71E-09 |
| sp\|Q16873\|LTC4S_HUMAN | 16652.18 | 0.56 | 0.119 | 1.14E-12 |
| sp\|Q6ZMG9\|CERS6_HUMAN | 45355.8 | 0.54 | 0.222 | 1.30E-07 |
| sp\|P48729\|KC1A_HUMAN | 39099.96 | 0.53 | 0.117 | 2.86E-14 |
| sp\|O43617\|TPPC3_HUMAN | 20414.2 | 0.64 | 0.148 | 7.53E-10 |
| sp\|P13688\|CEAM1_HUMAN | 57962.87 | 0.66 | 0.322 | 3.30E-05 |
| sp\|Q5W0V3\|F16B1_HUMAN | 87568.46 | 0.66 | 0.128 | 1.00E-10 |
| sp\|Q9Y2J8\|PADI2_HUMAN | 76238.61 | 0.46 | 0.099 | 7.26E-15 |
| sp\|Q14409\|GLPK3_HUMAN | 61396.05 | 0.56 | 0.143 | 1.51E-11 |
| sp\|Q6UX01\|LMBRL_HUMAN | 55839.62 | 0.66 | 0.173 | 3.43E-08 |
| sp\|Q13838\|DX39B_HUMAN | 49398.1 | 0.54 | 0.064 | 2.20E-16 |
| sp\|O43242\|PSMD3_HUMAN | 61035.52 | 0.58 | 0.117 | 3.88E-13 |
| sp\|O75052\|CAPON_HUMAN | 56438.91 | 0.62 | 0.211 | 2.49E-07 |
| sp\|Q8N1B4\|VPS52_HUMAN | 82493.74 | 0.6 | 0.082 | 7.47E-16 |
| sp\|Q14376\|GALE_HUMAN | 38638.4 | 0.53 | 0.191 | 2.44E-08 |
| sp\|Q9Y5M8\|SRPRB_HUMAN | 29893.84 | 0.38 | 0.113 | 3.79E-14 |
| sp\|Q5H8C1\|FREM1_HUMAN | 245807.2 | 0.56 | 0.094 | 1.77E-15 |
| sp\|P37837\|TALDO_HUMAN | 37669.52 | 0.56 | 0.092 | 1.38E-15 |
| sp\|Q9UM00\|TMCO1_HUMAN | 21371.23 | 0.57 | 0.145 | 8.70E-12 |
| sp\|Q9Y2E8\|SL9A8_HUMAN | 65589.35 | 0.56 | 0.094 | 3.14E-15 |
| sp\|P26572\|MGAT1_HUMAN | 51113.5 | 0.63 | 0.088 | 6.80E-15 |
| sp\|Q5NDL2\|EOGT_HUMAN | 62808.08 | 0.66 | 0.107 | 7.39E-13 |
| sp\|O75164\|KDM4A_HUMAN | 122562.8 | 0.47 | 0.069 | 2.20E-16 |
| sp\|Q9UBB6\|NCDN_HUMAN | 80221.16 | 0.58 | 0.063 | 2.20E-16 |
| sp\|Q15648\|MED1_HUMAN | 169324.5 | 0.55 | 0.056 | 2.20E-16 |
| sp\|Q9H9Y6\|RPA2_HUMAN | 129781.3 | 0.53 | 0.116 | 1.28E-13 |
| sp\|Q93088\|BHMT1_HUMAN | 45408.01 | 0.59 | 0.107 | 3.89E-13 |
| sp\|Q13085\|ACACA_HUMAN | 267077.5 | 0.66 | 0.119 | 1.86E-10 |
| sp\|Q96BQ1\|FAM3D_HUMAN | 25213.87 | 0.47 | 0.205 | 8.43E-10 |
| sp\|Q7LG56\|RIR2B_HUMAN | 40920.52 | 0.64 | 0.129 | 3.12E-11 |
| sp\|P62140\|PP1B_HUMAN | 37942.92 | 0.66 | 0.069 | 2.20E-16 |
| sp\|O15127\|SCAM2_HUMAN | 37063.5 | 0.55 | 0.089 | 3.33E-16 |
| sp\|Q9BTY2\|FUCO2_HUMAN | 54356.24 | 0.45 | 0.061 | 2.20E-16 |
| sp\|Q9NR28\|DBLOH_HUMAN | 27323.8 | 0.62 | 0.087 | 4.47E-15 |
| sp\|Q10570\|CPSF1_HUMAN | 162018.5 | 0.63 | 0.098 | 9.36E-14 |
| sp\|P53621\|COPA_HUMAN | 139779.5 | 0.66 | 0.089 | 3.17E-14 |
| sp\|Q8N465\|D2HDH_HUMAN | 57046.46 | 0.56 | 0.148 | 8.18E-11 |
| sp\|P18283\|GPX2_HUMAN | 22151.18 | 0.62 | 0.377 | 4.64E-06 |
| sp\|Q96A29\|FUCT1_HUMAN | 40277.97 | 0.53 | 0.141 | 1.18E-12 |
| sp\|P20594\|ANPRB_HUMAN | 117785.3 | 0.53 | 0.188 | 6.94E-09 |
| sp\|Q8IVU3\|HERC6_HUMAN | 116516.9 | 0.66 | 0.263 | 4.35E-05 |
| sp\|P62244\|RS15A_HUMAN | 14926 | 0.49 | 0.072 | 2.20E-16 |
| sp\|P21217\|FUT3_HUMAN | 42471.38 | 0.6 | 0.175 | 5.09E-10 |
| sp\|Q13423\|NNTM_HUMAN | 114545.8 | 0.63 | 0.125 | 8.17E-12 |
| sp\|Q92629\|SGCD_HUMAN | 32374.97 | 0.51 | 0.191 | 1.76E-10 |
| sp\|Q8N0U8\|VKORL_HUMAN | 20089.77 | 0.45 | 0.11 | 6.45E-15 |
| sp\|Q92845\|KIFA3_HUMAN | 91926.74 | 0.61 | 0.235 | 7.01E-08 |
| sp\|Q9NRD5\|PICK1_HUMAN | 47008.79 | 0.55 | 0.168 | 1.50E-10 |
| sp\|P53794\|SC5A3_HUMAN | 80478.61 | 0.57 | 0.173 | 3.49E-09 |
| sp\|Q13155\|AIMP2_HUMAN | 35650.4 | 0.66 | 0.113 | 3.29E-12 |
| sp\|O75112\|LDB3_HUMAN | 78208.34 | 0.65 | 0.31 | 8.55E-07 |
| sp\|Q9Y6R4\|M3K4_HUMAN | 183262.2 | 0.57 | 0.07 | 2.20E-16 |
| sp\|P19801\|AOC1_HUMAN | 85704.84 | 0.61 | 0.207 | 1.37E-08 |
| sp\|P61009\|SPCS3_HUMAN | 20339.55 | 0.54 | 0.203 | 2.76E-09 |
| sp\|Q13637\|RAB32_HUMAN | 25191.77 | 0.54 | 0.117 | 1.02E-13 |
| sp\|P20337\|RAB3B_HUMAN | 24952.19 | 0.58 | 0.216 | 4.04E-07 |
| sp\|Q9UI14\|PRAF1_HUMAN | 20787.8 | 0.64 | 0.105 | 4.70E-13 |
| sp\|Q6P1X5\|TAF2_HUMAN | 138347.6 | 0.49 | 0.066 | 2.20E-16 |
| sp\|Q8WZA1\|PMGT1_HUMAN | 75756.46 | 0.54 | 0.099 | 2.40E-15 |
| sp\|Q8NBX0\|SCPDL_HUMAN | 47445.57 | 0.59 | 0.156 | 2.80E-10 |
| sp\|Q9Y2G8\|DJC16_HUMAN | 91029.68 | 0.61 | 0.172 | 1.70E-09 |
| sp\|P13498\|CY24A_HUMAN | 21095.17 | 0.64 | 0.32 | 3.02E-06 |
| sp\|Q9UIQ6\|LCAP_HUMAN | 117769.4 | 0.47 | 0.066 | 2.20E-16 |
| sp\|O94876\|TMCC1_HUMAN | 72419.6 | 0.65 | 0.127 | 4.71E-11 |
| sp\|P17405\|ASM_HUMAN | 70899.73 | 0.57 | 0.119 | 1.51E-12 |
| sp\|P40394\|ADH7_HUMAN | 42234.56 | 0.59 | 0.271 | 8.42E-05 |
| sp\|P39656\|OST48_HUMAN | 50922.02 | 0.63 | 0.106 | 7.18E-13 |
| sp\|Q9NW08\|RPC2_HUMAN | 129223.9 | 0.63 | 0.076 | 4.12E-16 |
| sp\|P13224\|GP1BB_HUMAN | 22255.97 | 0.58 | 0.233 | 3.73E-07 |
| sp\|Q6NXT4\|ZNT6_HUMAN | 51406.9 | 0.63 | 0.147 | 1.21E-10 |
| sp\|Q86YL5\|TDRP_HUMAN | 20429.32 | 0.64 | 0.25 | 3.82E-07 |
| sp\|P51153\|RAB13_HUMAN | 22969.82 | 0.58 | 0.125 | 1.22E-12 |
| sp\|Q9NR48\|ASH1L_HUMAN | 336156.3 | 0.57 | 0.078 | 2.20E-16 |
| sp\|Q9BZH6\|WDR11_HUMAN | 138404.7 | 0.38 | 0.13 | 8.48E-14 |
| sp\|P63092\|GNAS2_HUMAN | 46074.06 | 0.66 | 0.082 | 5.25E-15 |
| sp\|O75298\|RTN2_HUMAN | 59437.6 | 0.57 | 0.201 | 2.29E-09 |
| sp\|P21266\|GSTM3_HUMAN | 26980.3 | 0.47 | 0.179 | 4.10E-11 |
| sp\|P13637\|AT1A3_HUMAN | 113084.4 | 0.62 | 0.236 | 1.85E-07 |
| sp\|Q13488\|VPP3_HUMAN | 93631.62 | 0.66 | 0.164 | 2.92E-09 |
| sp\|Q16537\|2A5E_HUMAN | 55045.63 | 0.52 | 0.17 | 2.59E-11 |
| sp\|Q2UY09\|COSA1_HUMAN | 117422.2 | 0.62 | 0.292 | 8.92E-06 |
| sp\|Q9H0U3\|MAGT1_HUMAN | 38392.55 | 0.53 | 0.084 | 2.20E-16 |
| sp\|Q96FX7\|TRM61_HUMAN | 31686 | 0.61 | 0.104 | 7.93E-14 |
| sp\|Q58EX2\|SDK2_HUMAN | 240142.1 | 0.52 | 0.203 | 6.35E-09 |
| sp\|Q8WVD5\|RN141_HUMAN | 26070.6 | 0.64 | 0.118 | 8.83E-12 |
| sp\|Q13882\|PTK6_HUMAN | 52353.39 | 0.4 | 0.199 | 5.88E-12 |
| sp\|Q9BUE0\|MED18_HUMAN | 23742.95 | 0.65 | 0.245 | 9.23E-07 |
| sp\|Q9HBI0\|PARVG_HUMAN | 37557.96 | 0.36 | 0.097 | 2.32E-16 |
| sp\|Q8N766\|EMC1_HUMAN | 112127.3 | 0.66 | 0.1 | 1.20E-12 |
| sp\|Q96G23\|CERS2_HUMAN | 44943.36 | 0.57 | 0.099 | 4.56E-15 |
| sp\|Q15008\|PSMD6_HUMAN | 45769.37 | 0.65 | 0.095 | 7.37E-14 |
| sp\|P47897\|SYQ_HUMAN | 88637.21 | 0.66 | 0.092 | 7.60E-14 |
| sp\|P08195\|4F2_HUMAN | 68161.8 | 0.61 | 0.08 | 2.72E-16 |
| sp\|Q9Y257\|KCNK6_HUMAN | 33992.88 | 0.61 | 0.116 | 4.31E-13 |
| sp\|O43776\|SYNC_HUMAN | 63739.83 | 0.63 | 0.202 | 2.89E-08 |
| sp\|O75436\|VP26A_HUMAN | 38241.78 | 0.58 | 0.059 | 2.20E-16 |
| sp\|O14772\|FPGT_HUMAN | 68916.98 | 0.48 | 0.159 | 8.65E-12 |
| sp\|Q9BZF1\|OSBL8_HUMAN | 101741.5 | 0.65 | 0.11 | 1.72E-12 |
| sp\|Q14728\|MFS10_HUMAN | 48689 | 0.49 | 0.101 | 3.41E-15 |
| sp\|Q9NVH2\|INT7_HUMAN | 108002.5 | 0.39 | 0.056 | 2.20E-16 |
| sp\|P60033\|CD81_HUMAN | 26458.36 | 0.43 | 0.158 | 1.10E-11 |
| sp\|Q8NBQ5\|DHB11_HUMAN | 33238.85 | 0.52 | 0.105 | 1.05E-13 |
| sp\|Q9Y3V2\|RWDD3_HUMAN | 30791 | 0.56 | 0.313 | 8.38E-08 |
| sp\|O60762\|DPM1_HUMAN | 29654.79 | 0.48 | 0.146 | 2.07E-12 |
| sp\|Q9H081\|MIS12_HUMAN | 24448.44 | 0.65 | 0.155 | 5.41E-10 |
| sp\|Q9H8H3\|MET7A_HUMAN | 28795.74 | 0.53 | 0.242 | 7.67E-08 |
| sp\|O95294\|RASL1_HUMAN | 90853.5 | 0.45 | 0.226 | 1.03E-08 |
| sp\|Q9Y6K5\|OAS3_HUMAN | 122842.7 | 0.65 | 0.189 | 3.47E-08 |
| sp\|Q13232\|NDK3_HUMAN | 19213 | 0.63 | 0.163 | 6.49E-09 |
| sp\|Q9NTJ5\|SAC1_HUMAN | 67419.19 | 0.55 | 0.109 | 7.06E-14 |
| sp\|Q8IYS2\|K2013_HUMAN | 69665.69 | 0.66 | 0.096 | 6.51E-13 |
| sp\|Q14997\|PSME4_HUMAN | 213518.5 | 0.62 | 0.114 | 4.19E-12 |
| sp\|P05164\|PERM_HUMAN | 84766.19 | 0.65 | 0.319 | 1.69E-05 |
| sp\|Q9HC21\|TPC_HUMAN | 35869.46 | 0.57 | 0.093 | 5.23E-15 |
| sp\|Q8N3U4\|STAG2_HUMAN | 142414.9 | 0.57 | 0.059 | 2.20E-16 |
| sp\|Q14BN4\|SLMAP_HUMAN | 95976.92 | 0.62 | 0.221 | 1.57E-07 |
| sp\|Q9BXJ9\|NAA15_HUMAN | 102444.2 | 0.65 | 0.103 | 1.92E-13 |
| sp\|A6NHL2\|TBAL3_HUMAN | 50657.02 | 0.24 | 0.052 | 2.20E-16 |
| sp\|Q92538\|GBF1_HUMAN | 208349.4 | 0.63 | 0.131 | 2.82E-11 |
| sp\|P20702\|ITAX_HUMAN | 128928.1 | 0.66 | 0.129 | 6.72E-11 |
| sp\|Q8IZV5\|RDH10_HUMAN | 38728.67 | 0.36 | 0.152 | 1.11E-12 |
| sp\|P17661\|DESM_HUMAN | 53542.16 | 0.52 | 0.245 | 4.41E-07 |
| sp\|Q13614\|MTMR2_HUMAN | 73887.23 | 0.65 | 0.089 | 3.43E-14 |
| sp\|Q6UXV4\|MIC27_HUMAN | 29293.31 | 0.64 | 0.103 | 5.84E-13 |
| sp\|Q9UKD2\|MRT4_HUMAN | 27638.94 | 0.58 | 0.235 | 7.87E-08 |
| sp\|Q8N9Z2\|CC71L_HUMAN | 26455.04 | 0.64 | 0.188 | 7.14E-08 |
| sp\|Q03426\|KIME_HUMAN | 42976.46 | 0.56 | 0.142 | 1.71E-11 |
| sp\|O60831\|PRAF2_HUMAN | 19569.64 | 0.53 | 0.103 | 5.73E-15 |
| sp\|Q96AA3\|RFT1_HUMAN | 60848.6 | 0.58 | 0.087 | 1.96E-15 |
| sp\|P04424\|ARLY_HUMAN | 51892.4 | 0.54 | 0.123 | 3.56E-13 |
| sp\|P62341\|SELT_HUMAN | 22463.39 | 0.58 | 0.093 | 5.63E-15 |
| sp\|P51993\|FUT6_HUMAN | 42270.93 | 0.47 | 0.091 | 2.20E-16 |
| sp\|Q969L2\|MAL2_HUMAN | 19322.77 | 0.58 | 0.155 | 1.16E-10 |
| sp\|Q99643\|C560_HUMAN | 18807.86 | 0.38 | 0.106 | 1.14E-15 |
| sp\|Q6ZN84\|CCD81_HUMAN | 76758.54 | 0.63 | 0.174 | 5.02E-10 |
| sp\|P20132\|SDHL_HUMAN | 34927.35 | 0.46 | 0.138 | 2.41E-13 |
| sp\|P35221\|CTNA1_HUMAN | 100674.8 | 0.63 | 0.184 | 2.91E-08 |
| sp\|Q643R3\|LPCT4_HUMAN | 57621.45 | 0.38 | 0.197 | 7.12E-11 |
| sp\|Q9H1C7\|CYTM1_HUMAN | 10947.88 | 0.59 | 0.257 | 1.99E-06 |
| sp\|Q5VTQ0\|TT39B_HUMAN | 77857.81 | 0.33 | 0.097 | 2.20E-16 |
| sp\|Q96BX8\|MOB3A_HUMAN | 25657.94 | 0.47 | 0.115 | 7.30E-14 |
| sp\|O75882\|ATRN_HUMAN | 163432.3 | 0.53 | 0.098 | 1.65E-15 |
| sp\|Q7Z4L5\|TT21B_HUMAN | 152361.7 | 0.62 | 0.121 | 3.30E-12 |
| sp\|P35222\|CTNB1_HUMAN | 86051.44 | 0.6 | 0.174 | 1.53E-09 |
| sp\|Q9ULE4\|F184B_HUMAN | 121863.6 | 0.65 | 0.209 | 1.64E-07 |
| sp\|Q00610\|CLH1_HUMAN | 193242.2 | 0.55 | 0.079 | 2.20E-16 |
| sp\|Q9NVJ2\|ARL8B_HUMAN | 21735.22 | 0.53 | 0.047 | 2.20E-16 |
| sp\|Q14139\|UBE4A_HUMAN | 123547.4 | 0.64 | 0.083 | 1.72E-15 |
| sp\|P05026\|AT1B1_HUMAN | 35420.01 | 0.44 | 0.199 | 2.45E-11 |
| sp\|Q99895\|CTRC_HUMAN | 30074.07 | 0.63 | 0.296 | 6.71E-06 |
| sp\|Q9NSE4\|SYIM_HUMAN | 114670.4 | 0.64 | 0.109 | 5.97E-13 |
| sp\|P09488\|GSTM1_HUMAN | 25905.13 | 0.61 | 0.371 | 3.05E-05 |
| sp\|P42345\|MTOR_HUMAN | 290741.4 | 0.66 | 0.047 | 2.20E-16 |
| sp\|P09471\|GNAO_HUMAN | 40577.1 | 0.58 | 0.222 | 2.30E-08 |
| sp\|P17301\|ITA2_HUMAN | 130450.3 | 0.58 | 0.163 | 1.32E-09 |
| sp\|P12724\|ECP_HUMAN | 18868.53 | 0.64 | 0.199 | 1.16E-06 |
| sp\|Q96H20\|SNF8_HUMAN | 28942.13 | 0.58 | 0.126 | 9.86E-13 |
| sp\|Q14722\|KCAB1_HUMAN | 46972.04 | 0.64 | 0.193 | 3.23E-09 |
| sp\|P16422\|EPCAM_HUMAN | 35575.94 | 0.48 | 0.306 | 3.00E-07 |
| sp\|O43660\|PLRG1_HUMAN | 57482.22 | 0.65 | 0.118 | 7.29E-12 |
| sp\|O00629\|IMA3_HUMAN | 58345.82 | 0.32 | 0.305 | 1.25E-08 |
| sp\|P07098\|LIPG_HUMAN | 45361.76 | 0.3 | 0.143 | 2.88E-13 |
| sp\|Q13617\|CUL2_HUMAN | 87535.99 | 0.63 | 0.068 | 2.20E-16 |
| sp\|Q6ZS30\|NBEL1_HUMAN | 310216.6 | 0.62 | 0.184 | 2.49E-08 |
| sp\|O75051\|PLXA2_HUMAN | 214201.7 | 0.52 | 0.157 | 3.34E-10 |
| sp\|Q5K4L6\|S27A3_HUMAN | 74227.21 | 0.62 | 0.115 | 5.60E-12 |
| sp\|Q9UPU5\|UBP24_HUMAN | 297182.4 | 0.56 | 0.113 | 1.03E-13 |
| sp\|P49748\|ACADV_HUMAN | 70726.57 | 0.64 | 0.176 | 1.82E-08 |
| sp\|Q8TAD4\|ZNT5_HUMAN | 84885.96 | 0.66 | 0.145 | 4.28E-10 |
| sp\|O43556\|SGCE_HUMAN | 50200.26 | 0.48 | 0.175 | 2.06E-11 |
| sp\|Q6UXG2\|K1324_HUMAN | 114427.1 | 0.55 | 0.205 | 6.03E-09 |
| sp\|P98194\|AT2C1_HUMAN | 101634.7 | 0.65 | 0.096 | 2.87E-13 |
| sp\|Q9GZQ3\|COMD5_HUMAN | 24807.94 | 0.59 | 0.085 | 1.62E-15 |
| sp\|Q13496\|MTM1_HUMAN | 70382.74 | 0.62 | 0.103 | 5.98E-14 |
| sp\|Q9UBY8\|CLN8_HUMAN | 33146.8 | 0.53 | 0.159 | 8.42E-12 |
| sp\|P02786\|TFR1_HUMAN | 85256.1 | 0.56 | 0.126 | 6.05E-13 |
| sp\|Q8IUR0\|TPPC5_HUMAN | 20866.18 | 0.55 | 0.13 | 1.23E-12 |
| sp\|Q5T4S7\|UBR4_HUMAN | 580529.1 | 0.62 | 0.054 | 2.20E-16 |
| sp\|Q8N3Y7\|RDHE2_HUMAN | 34567.97 | 0.41 | 0.365 | 3.74E-07 |
| sp\|P35613\|BASI_HUMAN | 42555.2 | 0.58 | 0.104 | 3.99E-14 |
| sp\|O43264\|ZW10_HUMAN | 89610.18 | 0.58 | 0.124 | 1.56E-12 |
| sp\|Q6P1R4\|DUS1L_HUMAN | 54318.53 | 0.66 | 0.163 | 3.47E-09 |
| sp\|Q9HD89\|RETN_HUMAN | 12077.71 | 0.61 | 0.448 | 2.19E-05 |
| sp\|P43652\|AFAM_HUMAN | 70944.73 | 0.63 | 0.157 | 1.48E-09 |
| sp\|Q8NC44\|RETR2_HUMAN | 58118.26 | 0.62 | 0.126 | 8.27E-12 |
| sp\|Q15286\|RAB35_HUMAN | 23277.86 | 0.54 | 0.067 | 2.20E-16 |
| sp\|P08962\|CD63_HUMAN | 26456.4 | 0.42 | 0.183 | 5.05E-11 |
| sp\|O95622\|ADCY5_HUMAN | 140739 | 0.6 | 0.131 | 1.74E-11 |
| sp\|P36222\|CH3L1_HUMAN | 42979.54 | 0.63 | 0.383 | 2.44E-05 |
| sp\|Q9H9S4\|CB39L_HUMAN | 39215.83 | 0.3 | 0.179 | 2.74E-12 |
| sp\|P62837\|UB2D2_HUMAN | 16934.5 | 0.56 | 0.073 | 2.20E-16 |
| sp\|Q5QGZ9\|CL12A_HUMAN | 31236.58 | 0.64 | 0.365 | 2.57E-05 |
| sp\|Q9UBF2\|COPG2_HUMAN | 98682 | 0.58 | 0.065 | 2.20E-16 |
| sp\|O43414\|ERI3_HUMAN | 37708.86 | 0.42 | 0.182 | 5.41E-12 |
| sp\|P25092\|GUC2C_HUMAN | 124332.1 | 0.62 | 0.269 | 3.09E-06 |
| sp\|O14735\|CDIPT_HUMAN | 23847.25 | 0.47 | 0.178 | 5.88E-11 |
| sp\|Q8N2G8\|GHDC_HUMAN | 58039.5 | 0.65 | 0.108 | 1.54E-12 |
| sp\|Q96E52\|OMA1_HUMAN | 60747.69 | 0.46 | 0.223 | 1.89E-08 |
| sp\|Q8IZR5\|CKLF4_HUMAN | 26078.37 | 0.58 | 0.237 | 4.63E-07 |
| sp\|Q9UPR3\|SMG5_HUMAN | 115433.9 | 0.44 | 0.06 | 2.20E-16 |
| sp\|P08236\|BGLR_HUMAN | 75008.81 | 0.63 | 0.122 | 3.08E-12 |
| sp\|Q8TB96\|TIP_HUMAN | 68445.61 | 0.58 | 0.124 | 2.03E-12 |
| sp\|P08910\|ABHD2_HUMAN | 49063.41 | 0.5 | 0.095 | 1.45E-15 |
| sp\|Q7Z304\|MAMC2_HUMAN | 78571.21 | 0.51 | 0.129 | 8.34E-14 |
| sp\|Q9Y399\|RT02_HUMAN | 33495.17 | 0.46 | 0.12 | 6.41E-14 |
| sp\|P25024\|CXCR1_HUMAN | 40317.06 | 0.66 | 0.372 | 4.81E-05 |
| sp\|P61026\|RAB10_HUMAN | 22736.66 | 0.24 | 0.047 | 2.20E-16 |
| sp\|Q8N1S5\|S39AB_HUMAN | 35355.38 | 0.59 | 0.156 | 1.15E-08 |
| sp\|Q99698\|LYST_HUMAN | 434151.3 | 0.55 | 0.064 | 2.20E-16 |
| sp\|Q96AX2\|RAB37_HUMAN | 25009.55 | 0.58 | 0.102 | 3.67E-14 |
| sp\|Q5T3F8\|CSCL2_HUMAN | 96474.72 | 0.66 | 0.075 | 1.74E-15 |
| sp\|Q86UD3\|MARH3_HUMAN | 29208.68 | 0.46 | 0.163 | 8.86E-11 |
| sp\|P26641\|EF1G_HUMAN | 50411.26 | 0.59 | 0.112 | 3.25E-13 |
| sp\|O14727\|APAF_HUMAN | 144068.9 | 0.64 | 0.168 | 1.47E-09 |
| sp\|Q15283\|RASA2_HUMAN | 97903.42 | 0.63 | 0.116 | 2.01E-12 |
| sp\|P53992\|SC24C_HUMAN | 119770.7 | 0.59 | 0.103 | 1.27E-14 |
| sp\|O75355\|ENTP3_HUMAN | 59790.52 | 0.56 | 0.239 | 5.78E-07 |
| sp\|Q9UL18\|AGO1_HUMAN | 98274.54 | 0.59 | 0.142 | 5.68E-11 |
| sp\|O95822\|DCMC_HUMAN | 55349.89 | 0.59 | 0.187 | 5.47E-09 |
| sp\|Q96A65\|EXOC4_HUMAN | 111151.8 | 0.57 | 0.101 | 2.24E-14 |
| sp\|Q9NRG9\|AAAS_HUMAN | 60373.64 | 0.6 | 0.113 | 4.53E-13 |
| sp\|Q8NAV1\|PR38A_HUMAN | 37663.46 | 0.62 | 0.105 | 2.52E-13 |
| sp\|P08134\|RHOC_HUMAN | 22316.37 | 0.42 | 0.083 | 2.20E-16 |
| sp\|O15270\|SPTC2_HUMAN | 63550.11 | 0.55 | 0.146 | 1.08E-11 |
| sp\|Q6ZRP7\|QSOX2_HUMAN | 78202.88 | 0.61 | 0.136 | 2.30E-11 |
| sp\|P31513\|FMO3_HUMAN | 60603.6 | 0.56 | 0.121 | 7.28E-13 |
| sp\|Q9Y376\|CAB39_HUMAN | 39996.8 | 0.37 | 0.06 | 2.20E-16 |
| sp\|P11166\|GTR1_HUMAN | 54372.65 | 0.66 | 0.202 | 8.97E-08 |
| sp\|Q9NZJ5\|E2AK3_HUMAN | 126145.9 | 0.64 | 0.1 | 9.40E-14 |
| sp\|P17858\|PFKAL_HUMAN | 85744.45 | 0.61 | 0.15 | 4.90E-10 |
| sp\|Q86Y39\|NDUAB_HUMAN | 15052.61 | 0.36 | 0.153 | 2.18E-11 |
| sp\|P18124\|RL7_HUMAN | 29246.21 | 0.65 | 0.107 | 1.37E-12 |
| sp\|Q6P996\|PDXD1_HUMAN | 87546.64 | 0.64 | 0.2 | 4.16E-08 |
| sp\|P12931\|SRC_HUMAN | 60292.18 | 0.52 | 0.17 | 7.51E-11 |
| sp\|P10319\|1B58_HUMAN | 40579.14 | 0.62 | 0.376 | 5.11E-05 |
| sp\|Q9BYX2\|TBD2A_HUMAN | 106242.4 | 0.54 | 0.206 | 5.43E-09 |
| sp\|P11766\|ADHX_HUMAN | 40535.69 | 0.57 | 0.134 | 2.77E-12 |
| sp\|P51798\|CLCN7_HUMAN | 89117.62 | 0.58 | 0.143 | 2.78E-11 |
| sp\|Q8NFT2\|STEA2_HUMAN | 56286.75 | 0.65 | 0.296 | 1.09E-05 |
| sp\|P55011\|S12A2_HUMAN | 132030 | 0.57 | 0.148 | 3.43E-12 |
| sp\|Q96MH6\|TMM68_HUMAN | 37667.64 | 0.66 | 0.168 | 6.91E-09 |
| sp\|O15061\|SYNEM_HUMAN | 172987.4 | 0.66 | 0.282 | 2.53E-05 |
| sp\|Q9UK99\|FBX3_HUMAN | 55362.74 | 0.47 | 0.095 | 9.38E-16 |
| sp\|Q9Y4D8\|HECD4_HUMAN | 444692.1 | 0.59 | 0.121 | 1.74E-12 |
| sp\|Q93099\|HGD_HUMAN | 50597.84 | 0.65 | 0.122 | 8.10E-11 |
| sp\|O14965\|AURKA_HUMAN | 46161.98 | 0.55 | 0.182 | 8.06E-10 |
| sp\|O75363\|BCAS1_HUMAN | 61938.57 | 0.57 | 0.28 | 1.98E-06 |
| sp\|P18085\|ARF4_HUMAN | 20593.73 | 0.66 | 0.089 | 3.02E-14 |
| sp\|P07478\|TRY2_HUMAN | 26909.2 | 0.5 | 0.257 | 5.30E-07 |
| sp\|Q13087\|PDIA2_HUMAN | 58493.81 | 0.62 | 0.337 | 7.48E-05 |
| sp\|Q9NP80\|PLPL8_HUMAN | 89201.32 | 0.61 | 0.092 | 1.94E-14 |
| sp\|P41091\|IF2G_HUMAN | 51629.42 | 0.62 | 0.11 | 6.47E-13 |
| sp\|Q9NRK6\|ABCBA_HUMAN | 79479.08 | 0.62 | 0.112 | 4.74E-13 |
| sp\|Q9NRP0\|OSTC_HUMAN | 16913.87 | 0.64 | 0.114 | 1.89E-12 |
| sp\|Q9NRZ7\|PLCC_HUMAN | 43904.93 | 0.47 | 0.159 | 8.10E-12 |
| sp\|Q5SWX8\|ODR4_HUMAN | 51680 | 0.35 | 0.126 | 4.21E-14 |
| sp\|Q15120\|PDK3_HUMAN | 47062.12 | 0.62 | 0.123 | 3.33E-11 |
| sp\|P61163\|ACTZ_HUMAN | 42682.94 | 0.64 | 0.152 | 1.60E-09 |
| sp\|P52790\|HXK3_HUMAN | 100597.7 | 0.66 | 0.272 | 3.44E-06 |
| sp\|P11216\|PYGB_HUMAN | 97300.77 | 0.5 | 0.124 | 4.71E-13 |
| sp\|Q7Z404\|TMC4_HUMAN | 80051.43 | 0.46 | 0.152 | 5.02E-12 |
| sp\|P12236\|ADT3_HUMAN | 33055.26 | 0.4 | 0.122 | 1.22E-13 |
| sp\|Q92973\|TNPO1_HUMAN | 103753.3 | 0.5 | 0.069 | 2.20E-16 |
| sp\|Q6P1A2\|MBOA5_HUMAN | 56493.28 | 0.36 | 0.115 | 9.19E-13 |
| sp\|Q53GQ0\|DHB12_HUMAN | 34398.22 | 0.51 | 0.14 | 5.55E-13 |
| sp\|Q9H4A6\|GOLP3_HUMAN | 34056.6 | 0.66 | 0.171 | 2.13E-09 |
| sp\|Q86TM6\|SYVN1_HUMAN | 68135.5 | 0.63 | 0.153 | 9.56E-10 |
| sp\|O00116\|ADAS_HUMAN | 73646.04 | 0.64 | 0.146 | 2.79E-10 |
| sp\|Q15784\|NDF2_HUMAN | 41716.46 | 0.63 | 0.189 | 7.18E-09 |
| sp\|Q9H1P3\|OSBL2_HUMAN | 55661.47 | 0.52 | 0.093 | 9.12E-16 |
| sp\|Q14165\|MLEC_HUMAN | 32366.64 | 0.53 | 0.091 | 2.20E-16 |
| sp\|Q5JS54\|PSMG4_HUMAN | 13804.98 | 0.64 | 0.079 | 6.89E-16 |
| sp\|Q9UID3\|VPS51_HUMAN | 86882.56 | 0.65 | 0.094 | 1.24E-13 |
| sp\|Q7Z3C6\|ATG9A_HUMAN | 95338.38 | 0.45 | 0.147 | 1.52E-12 |
| sp\|O14672\|ADA10_HUMAN | 86122.45 | 0.63 | 0.13 | 1.58E-10 |
| sp\|O75787\|RENR_HUMAN | 38965.31 | 0.64 | 0.11 | 1.07E-12 |
| sp\|P14324\|FPPS_HUMAN | 48739.81 | 0.52 | 0.119 | 1.69E-13 |
| sp\|P39900\|MMP12_HUMAN | 54120.2 | 0.64 | 0.32 | 7.26E-06 |
| sp\|Q15124\|PGM5_HUMAN | 62737.9 | 0.49 | 0.183 | 2.01E-10 |
| sp\|Q96H55\|MYO19_HUMAN | 110758.2 | 0.59 | 0.099 | 7.54E-14 |
| sp\|P41252\|SYIC_HUMAN | 145699.5 | 0.58 | 0.127 | 3.07E-12 |
| sp\|Q9HBY0\|NOX3_HUMAN | 65730.33 | 0.37 | 0.153 | 8.05E-12 |
| sp\|P19075\|TSN8_HUMAN | 26692.73 | 0.61 | 0.417 | 2.67E-05 |
| sp\|Q7Z392\|TPC11_HUMAN | 130433.6 | 0.65 | 0.09 | 2.98E-14 |
| sp\|P60953\|CDC42_HUMAN | 21569.14 | 0.66 | 0.097 | 2.56E-13 |
| sp\|P30520\|PURA2_HUMAN | 50446.91 | 0.62 | 0.136 | 1.37E-11 |
| sp\|Q8IXH7\|NELFD_HUMAN | 66813.56 | 0.55 | 0.099 | 1.08E-14 |
| sp\|Q96K49\|TM87B_HUMAN | 64160.96 | 0.64 | 0.173 | 1.25E-09 |
| sp\|Q9H0R3\|TM222_HUMAN | 23595.58 | 0.63 | 0.247 | 4.28E-07 |
| sp\|Q9Y6A9\|SPCS1_HUMAN | 11836.13 | 0.56 | 0.154 | 7.14E-11 |
| sp\|Q15393\|SF3B3_HUMAN | 136557.1 | 0.63 | 0.128 | 1.36E-11 |
| sp\|O60635\|TSN1_HUMAN | 26892.38 | 0.45 | 0.187 | 1.43E-10 |
| sp\|O00423\|EMAL1_HUMAN | 90756.07 | 0.63 | 0.167 | 9.46E-10 |
| sp\|Q6NUK4\|REEP3_HUMAN | 29227.23 | 0.52 | 0.081 | 2.20E-16 |
| sp\|P05556\|ITB1_HUMAN | 91646.24 | 0.65 | 0.092 | 1.17E-13 |
| sp\|P06744\|G6PI_HUMAN | 63317.32 | 0.52 | 0.053 | 2.20E-16 |
| sp\|Q8IX04\|UEVLD_HUMAN | 52498.13 | 0.63 | 0.095 | 3.69E-14 |
| sp\|Q14689\|DIP2A_HUMAN | 172352.9 | 0.61 | 0.109 | 9.83E-13 |
| sp\|Q9Y624\|JAM1_HUMAN | 32943.63 | 0.63 | 0.135 | 1.96E-11 |
| sp\|P53007\|TXTP_HUMAN | 34315.12 | 0.5 | 0.14 | 2.57E-12 |
| sp\|O95870\|ABHGA_HUMAN | 63812.31 | 0.58 | 0.09 | 2.40E-15 |
| sp\|P03897\|NU3M_HUMAN | 13216.06 | 0.52 | 0.163 | 4.60E-11 |
| sp\|P05388\|RLA0_HUMAN | 34404.85 | 0.49 | 0.114 | 3.75E-14 |
| sp\|Q5T5C0\|STXB5_HUMAN | 129070.5 | 0.65 | 0.148 | 1.92E-10 |
| sp\|P19367\|HXK1_HUMAN | 103542.6 | 0.55 | 0.113 | 9.79E-14 |
| sp\|Q6UW68\|TM205_HUMAN | 21450.99 | 0.6 | 0.069 | 2.20E-16 |
| sp\|Q9NUU6\|OTULL_HUMAN | 42549.7 | 0.51 | 0.132 | 2.11E-12 |
| sp\|Q9NV88\|INT9_HUMAN | 74775.11 | 0.54 | 0.155 | 1.02E-10 |
| sp\|Q9BQ13\|KCD14_HUMAN | 29782.11 | 0.59 | 0.31 | 2.53E-05 |
| sp\|Q8IZ81\|ELMD2_HUMAN | 35204.8 | 0.59 | 0.083 | 1.32E-15 |
| sp\|A7E2V4\|ZSWM8_HUMAN | 199321.8 | 0.55 | 0.143 | 8.78E-12 |
| sp\|Q330K2\|NDUF6_HUMAN | 38533.08 | 0.49 | 0.095 | 1.35E-15 |
| sp\|Q9NXJ5\|PGPI_HUMAN | 23561.62 | 0.6 | 0.113 | 3.66E-13 |
| sp\|Q86XK7\|VSIG1_HUMAN | 42223.2 | 0.58 | 0.36 | 5.94E-05 |
| sp\|Q01813\|PFKAP_HUMAN | 86435.86 | 0.66 | 0.149 | 4.36E-10 |
| sp\|Q14C86\|GAPD1_HUMAN | 166341 | 0.59 | 0.102 | 2.87E-14 |
| sp\|O95050\|INMT_HUMAN | 29481.61 | 0.56 | 0.246 | 1.33E-08 |
| sp\|Q13541\|4EBP1_HUMAN | 12668.13 | 0.58 | 0.281 | 5.89E-07 |
| sp\|Q6PIU2\|NCEH1_HUMAN | 46045.82 | 0.59 | 0.198 | 5.47E-09 |
| sp\|Q3SY17\|S2552_HUMAN | 34023.99 | 0.36 | 0.074 | 2.20E-16 |
| sp\|Q6P4A8\|PLBL1_HUMAN | 63481.28 | 0.56 | 0.153 | 2.02E-11 |
| sp\|P05106\|ITB3_HUMAN | 90175.6 | 0.59 | 0.194 | 2.10E-08 |
| sp\|P15151\|PVR_HUMAN | 45769.01 | 0.54 | 0.18 | 9.46E-10 |
| sp\|P00846\|ATP6_HUMAN | 24782.85 | 0.62 | 0.164 | 1.99E-09 |
| sp\|O14975\|S27A2_HUMAN | 71047.62 | 0.5 | 0.202 | 3.25E-10 |
| sp\|P11217\|PYGM_HUMAN | 97468.79 | 0.58 | 0.124 | 1.95E-12 |
| sp\|Q9H9B4\|SFXN1_HUMAN | 35863.44 | 0.65 | 0.088 | 1.61E-14 |
| sp\|Q9BXS9\|S26A6_HUMAN | 83352.15 | 0.52 | 0.288 | 3.92E-07 |
| sp\|Q14999\|CUL7_HUMAN | 192789.9 | 0.58 | 0.132 | 5.11E-12 |
| sp\|Q14232\|EI2BA_HUMAN | 33957.85 | 0.59 | 0.096 | 4.13E-15 |
| sp\|Q9BXJ8\|T120A_HUMAN | 41021.97 | 0.42 | 0.122 | 3.62E-14 |
| sp\|Q9Y262\|EIF3L_HUMAN | 66894.48 | 0.64 | 0.11 | 1.50E-12 |
| sp\|Q92621\|NU205_HUMAN | 230153.2 | 0.59 | 0.126 | 2.67E-12 |
| sp\|P20338\|RAB4A_HUMAN | 24641.31 | 0.55 | 0.094 | 3.54E-15 |
| sp\|Q70CQ2\|UBP34_HUMAN | 409714.1 | 0.66 | 0.237 | 4.73E-07 |
| sp\|Q13618\|CUL3_HUMAN | 89425.66 | 0.61 | 0.056 | 2.20E-16 |
| sp\|Q96JA1\|LRIG1_HUMAN | 120673.8 | 0.62 | 0.16 | 5.56E-10 |
| sp\|Q8NDH3\|PEPL1_HUMAN | 56719.8 | 0.62 | 0.073 | 2.20E-16 |
| sp\|Q5SZK8\|FREM2_HUMAN | 352572.8 | 0.66 | 0.205 | 2.13E-07 |
| sp\|Q8WXD2\|SCG3_HUMAN | 52954.87 | 0.36 | 0.192 | 4.96E-09 |
| sp\|O14656\|TOR1A_HUMAN | 38108.6 | 0.43 | 0.123 | 8.46E-14 |
| sp\|Q10471\|GALT2_HUMAN | 65414.73 | 0.51 | 0.077 | 2.20E-16 |
| sp\|O15321\|TM9S1_HUMAN | 69311.52 | 0.6 | 0.1 | 2.47E-14 |
| sp\|Q9UFN0\|NPS3A_HUMAN | 28544.57 | 0.63 | 0.127 | 6.64E-11 |
| sp\|Q13098\|CSN1_HUMAN | 56053.37 | 0.63 | 0.073 | 2.20E-16 |
| sp\|P53611\|PGTB2_HUMAN | 37566.55 | 0.65 | 0.172 | 5.70E-09 |
| sp\|Q6R327\|RICTR_HUMAN | 194188.6 | 0.58 | 0.095 | 8.27E-15 |
| sp\|P55060\|XPO2_HUMAN | 111126.6 | 0.57 | 0.094 | 3.26E-15 |
| sp\|P12235\|ADT1_HUMAN | 33253.29 | 0.59 | 0.236 | 1.04E-07 |
| sp\|Q7Z3E5\|ARMC9_HUMAN | 92484.24 | 0.56 | 0.067 | 2.20E-16 |
| sp\|Q9BZG1\|RAB34_HUMAN | 29464.09 | 0.53 | 0.093 | 5.56E-16 |
| sp\|Q05315\|LEG10_HUMAN | 16538.25 | 0.36 | 0.124 | 2.22E-12 |
| sp\|Q8IV36\|HID1_HUMAN | 89297.75 | 0.48 | 0.204 | 8.72E-10 |
| sp\|P27449\|VATL_HUMAN | 15707.45 | 0.36 | 0.085 | 2.20E-16 |
| sp\|P62736\|ACTA_HUMAN | 42362.95 | 0.58 | 0.237 | 7.48E-07 |
| sp\|O43657\|TSN6_HUMAN | 28154.29 | 0.53 | 0.33 | 6.25E-07 |
| sp\|P28845\|DHI1_HUMAN | 32589.71 | 0.53 | 0.276 | 7.18E-08 |
| sp\|Q9UKR5\|ERG28_HUMAN | 15949.57 | 0.48 | 0.119 | 3.79E-14 |
| sp\|O14925\|TIM23_HUMAN | 22082.05 | 0.61 | 0.122 | 5.84E-12 |
| sp\|B7ZAQ6\|GPHRA_HUMAN | 53148.98 | 0.57 | 0.129 | 6.55E-13 |
| sp\|O95139\|NDUB6_HUMAN | 15461.12 | 0.56 | 0.135 | 8.61E-12 |
| sp\|Q86VP6\|CAND1_HUMAN | 137981.3 | 0.48 | 0.083 | 2.20E-16 |
| sp\|Q96GW9\|SYMM_HUMAN | 67443.13 | 0.62 | 0.128 | 3.15E-11 |
| sp\|P51911\|CNN1_HUMAN | 33302.61 | 0.56 | 0.281 | 5.53E-07 |
| sp\|P53396\|ACLY_HUMAN | 121656.2 | 0.65 | 0.067 | 2.20E-16 |
| sp\|Q9UJS0\|CMC2_HUMAN | 74509.78 | 0.51 | 0.116 | 4.67E-14 |
| sp\|P29622\|KAIN_HUMAN | 48664.21 | 0.66 | 0.17 | 2.15E-09 |
| sp\|P54868\|HMCS2_HUMAN | 57094.6 | 0.63 | 0.366 | 5.30E-06 |
| sp\|Q8WVT3\|TPC12_HUMAN | 79763.28 | 0.53 | 0.13 | 1.14E-12 |
| sp\|Q9UJ83\|HACL1_HUMAN | 64410.62 | 0.63 | 0.325 | 7.34E-06 |
| sp\|P10153\|RNAS2_HUMAN | 18837.26 | 0.6 | 0.16 | 6.58E-09 |
| sp\|Q86X52\|CHSS1_HUMAN | 92334.96 | 0.52 | 0.278 | 1.78E-07 |
| sp\|Q9Y2Y8\|PRG3_HUMAN | 26169.3 | 0.53 | 0.191 | 1.44E-08 |
| sp\|Q12905\|ILF2_HUMAN | 43245.26 | 0.59 | 0.091 | 3.26E-15 |
| sp\|P51159\|RB27A_HUMAN | 25119.35 | 0.56 | 0.17 | 3.23E-10 |
| sp\|Q8N5B7\|CERS5_HUMAN | 46331.35 | 0.64 | 0.121 | 3.54E-12 |
| sp\|Q5TBA9\|FRY_HUMAN | 342062.3 | 0.59 | 0.134 | 1.03E-11 |
| sp\|Q9NSB2\|KRT84_HUMAN | 65923.88 | 0.46 | 0.22 | 5.30E-09 |
| sp\|Q13769\|THOC5_HUMAN | 79181.2 | 0.64 | 0.43 | 0.00013 |
| sp\|O15438\|MRP3_HUMAN | 170641.9 | 0.66 | 0.213 | 5.08E-07 |
| sp\|P01031\|CO5_HUMAN | 189878.8 | 0.62 | 0.248 | 4.01E-07 |
| sp\|Q8IYJ1\|CPNE9_HUMAN | 62263.26 | 0.56 | 0.173 | 3.18E-10 |
| sp\|Q9UBV7\|B4GT7_HUMAN | 37820.16 | 0.62 | 0.055 | 2.20E-16 |
| sp\|Q5VWZ2\|LYPL1_HUMAN | 26566.53 | 0.65 | 0.094 | 5.74E-14 |
| sp\|Q9UNL2\|SSRG_HUMAN | 21049.19 | 0.43 | 0.248 | 2.22E-09 |
| sp\|Q86SF2\|GALT7_HUMAN | 76007.27 | 0.66 | 0.253 | 6.81E-06 |
| sp\|Q13572\|ITPK1_HUMAN | 46144.31 | 0.61 | 0.195 | 1.12E-08 |
| sp\|Q9Y6C9\|MTCH2_HUMAN | 33918.09 | 0.57 | 0.131 | 4.60E-12 |
| sp\|Q14508\|WFDC2_HUMAN | 13935.23 | 0.58 | 0.417 | 3.14E-06 |
| sp\|Q6P5S2\|LEG1H_HUMAN | 38225.68 | 0.65 | 0.312 | 0.0006861 |
| sp\|Q969N2\|PIGT_HUMAN | 66210.14 | 0.59 | 0.124 | 1.99E-12 |
| sp\|O95834\|EMAL2_HUMAN | 71585.43 | 0.65 | 0.137 | 3.83E-10 |
| sp\|P62330\|ARF6_HUMAN | 20165.47 | 0.54 | 0.11 | 1.85E-13 |
| sp\|Q6UX53\|MET7B_HUMAN | 28251.46 | 0.49 | 0.156 | 1.35E-11 |
| sp\|Q99973\|TEP1_HUMAN | 293482.2 | 0.66 | 0.105 | 7.60E-13 |
| sp\|Q04656\|ATP7A_HUMAN | 164733.2 | 0.64 | 0.116 | 8.16E-12 |
| sp\|O14972\|DSCR3_HUMAN | 33427.47 | 0.63 | 0.109 | 1.44E-12 |
| sp\|Q9UBV8\|PEF1_HUMAN | 30628.4 | 0.63 | 0.065 | 2.20E-16 |
| sp\|Q6V1P9\|PCD23_HUMAN | 323726.3 | 0.53 | 0.154 | 5.18E-10 |
| sp\|Q12769\|NU160_HUMAN | 164336.8 | 0.64 | 0.118 | 3.36E-12 |
| sp\|O14791\|APOL1_HUMAN | 43985.91 | 0.5 | 0.13 | 3.37E-12 |
| sp\|P08842\|STS_HUMAN | 66287.37 | 0.65 | 0.195 | 2.18E-07 |
| sp\|Q9HCJ6\|VAT1L_HUMAN | 46194.26 | 0.53 | 0.205 | 1.84E-08 |
| sp\|P13727\|PRG2_HUMAN | 25855.5 | 0.59 | 0.184 | 9.07E-08 |
| sp\|Q9HB90\|RRAGC_HUMAN | 44576.97 | 0.62 | 0.13 | 6.40E-11 |
| sp\|Q9Y5P6\|GMPPB_HUMAN | 40360.79 | 0.64 | 0.184 | 1.58E-08 |
| sp\|P09960\|LKHA4_HUMAN | 69850.46 | 0.56 | 0.071 | 2.20E-16 |
| sp\|Q99460\|PSMD1_HUMAN | 106777.1 | 0.65 | 0.094 | 7.62E-14 |
| sp\|B2RTY4\|MYO9A_HUMAN | 294899.2 | 0.59 | 0.164 | 6.35E-10 |
| sp\|Q9Y5Z0\|BACE2_HUMAN | 56582.88 | 0.65 | 0.139 | 1.22E-10 |
| sp\|L0R6Q1\|S35U4_HUMAN | 11164.9 | 0.63 | 0.058 | 2.20E-16 |
| sp\|Q99571\|P2RX4_HUMAN | 44121.21 | 0.6 | 0.105 | 5.73E-14 |
| sp\|Q9UKK6\|NXT1_HUMAN | 16046.7 | 0.52 | 0.156 | 2.62E-11 |
| sp\|Q7Z6Z7\|HUWE1_HUMAN | 485505.1 | 0.66 | 0.093 | 9.88E-14 |
| sp\|O96008\|TOM40_HUMAN | 38193.26 | 0.51 | 0.164 | 5.33E-11 |
| sp\|O14880\|MGST3_HUMAN | 16715.64 | 0.51 | 0.141 | 2.82E-12 |
| sp\|Q86YS7\|C2CD5_HUMAN | 111898.1 | 0.64 | 0.151 | 2.17E-09 |
| sp\|O43747\|AP1G1_HUMAN | 92130.44 | 0.55 | 0.075 | 2.20E-16 |
| sp\|Q8IWT6\|LRC8A_HUMAN | 95033.02 | 0.59 | 0.058 | 2.20E-16 |
| sp\|P50993\|AT1A2_HUMAN | 113486.9 | 0.62 | 0.224 | 4.18E-08 |
| sp\|Q02083\|NAAA_HUMAN | 40193.92 | 0.52 | 0.156 | 7.05E-11 |
| sp\|Q9NUB1\|ACS2L_HUMAN | 75646.3 | 0.55 | 0.17 | 4.29E-10 |
| sp\|Q6Y1H2\|HACD2_HUMAN | 28389.15 | 0.66 | 0.148 | 1.02E-09 |
| sp\|Q2WGJ9\|FR1L6_HUMAN | 210412.6 | 0.65 | 0.229 | 3.25E-05 |
| sp\|O00194\|RB27B_HUMAN | 24802.3 | 0.59 | 0.232 | 6.49E-06 |
| sp\|O60462\|NRP2_HUMAN | 106114.5 | 0.61 | 0.068 | 2.20E-16 |
| sp\|Q68CQ4\|DIEXF_HUMAN | 87324.9 | 0.64 | 0.166 | 1.29E-09 |
| sp\|Q8NBU5\|ATAD1_HUMAN | 41042.11 | 0.63 | 0.116 | 5.25E-12 |
| sp\|Q96BI1\|S22AI_HUMAN | 45254.32 | 0.53 | 0.131 | 5.60E-12 |
| sp\|Q9NW15\|ANO10_HUMAN | 76775.01 | 0.54 | 0.052 | 2.20E-16 |
| sp\|P56199\|ITA1_HUMAN | 132286.3 | 0.63 | 0.154 | 2.51E-10 |
| sp\|O75746\|CMC1_HUMAN | 75095.99 | 0.51 | 0.107 | 1.55E-15 |
| sp\|Q969X5\|ERGI1_HUMAN | 32952.63 | 0.65 | 0.171 | 4.79E-09 |
| sp\|Q13362\|2A5G_HUMAN | 61402.71 | 0.63 | 0.064 | 2.20E-16 |
| sp\|Q7L7V1\|DHX32_HUMAN | 85771.34 | 0.62 | 0.261 | 8.36E-07 |
| sp\|P15309\|PPAP_HUMAN | 44861.59 | 0.48 | 0.33 | 4.02E-08 |
| sp\|Q86U38\|NOP9_HUMAN | 70117.72 | 0.66 | 0.157 | 1.40E-09 |
| sp\|Q969P0\|IGSF8_HUMAN | 65602.74 | 0.66 | 0.182 | 5.44E-08 |
| sp\|Q969U7\|PSMG2_HUMAN | 29758.17 | 0.59 | 0.294 | 2.44E-07 |
| sp\|Q9NVA1\|UQCC1_HUMAN | 34958.75 | 0.48 | 0.118 | 1.25E-13 |
| sp\|Q5XXA6\|ANO1_HUMAN | 115012.9 | 0.62 | 0.229 | 2.20E-06 |
| sp\|Q14204\|DYHC1_HUMAN | 534790.9 | 0.56 | 0.062 | 2.20E-16 |
| sp\|P63096\|GNAI1_HUMAN | 40887.43 | 0.49 | 0.18 | 7.05E-11 |
| sp\|Q9NV96\|CC50A_HUMAN | 41038.77 | 0.55 | 0.15 | 3.02E-11 |
| sp\|O15111\|IKKA_HUMAN | 85707.38 | 0.42 | 0.06 | 2.20E-16 |
| sp\|O43520\|AT8B1_HUMAN | 144783.6 | 0.47 | 0.169 | 2.15E-11 |
| sp\|P49720\|PSB3_HUMAN | 23200.55 | 0.6 | 0.184 | 1.05E-09 |
| sp\|P05109\|S10A8_HUMAN | 10866.66 | 0.56 | 0.361 | 5.59E-06 |
| sp\|Q6NUJ1\|SAPL1_HUMAN | 58452.83 | 0.62 | 0.286 | 0.0002721 |
| sp\|Q14914\|PTGR1_HUMAN | 36056.8 | 0.6 | 0.242 | 5.30E-07 |
| sp\|P28074\|PSB5_HUMAN | 28615.26 | 0.66 | 0.069 | 2.20E-16 |
| sp\|Q5EB52\|MEST_HUMAN | 38844.52 | 0.59 | 0.155 | 2.43E-10 |
| sp\|Q8IVN8\|SBSPO_HUMAN | 30998.04 | 0.48 | 0.452 | 5.10E-07 |
| sp\|Q6ZUT6\|CCD9B_HUMAN | 57785.01 | 0.5 | 0.459 | 1.35E-06 |
| sp\|Q8IX19\|MCEM1_HUMAN | 21425.03 | 0.45 | 0.272 | 7.66E-08 |
| sp\|Q9NZ01\|TECR_HUMAN | 36391.92 | 0.4 | 0.138 | 7.79E-14 |
| sp\|Q5ST30\|SYVM_HUMAN | 119537.2 | 0.64 | 0.112 | 9.77E-12 |
| sp\|Q8N5M9\|JAGN1_HUMAN | 21093.11 | 0.31 | 0.147 | 1.03E-12 |
| sp\|P35219\|CAH8_HUMAN | 33219.62 | 0.48 | 0.359 | 1.80E-07 |
| sp\|Q96RL7\|VP13A_HUMAN | 361854.1 | 0.54 | 0.089 | 1.37E-15 |
| sp\|P67775\|PP2AA_HUMAN | 36123.6 | 0.63 | 0.204 | 1.65E-08 |
| sp\|P78310\|CXAR_HUMAN | 40556.58 | 0.48 | 0.199 | 2.40E-10 |
| sp\|P61803\|DAD1_HUMAN | 12641.61 | 0.53 | 0.122 | 2.40E-13 |
| sp\|P00973\|OAS1_HUMAN | 46608.57 | 0.61 | 0.498 | 1.03E-05 |
| sp\|Q9NVD7\|PARVA_HUMAN | 42256.44 | 0.63 | 0.117 | 2.57E-12 |
| sp\|O14896\|IRF6_HUMAN | 53647.82 | 0.59 | 0.156 | 1.62E-10 |
| sp\|P20742\|PZP_HUMAN | 165224.1 | 0.61 | 0.214 | 1.70E-08 |
| sp\|Q8TCT9\|HM13_HUMAN | 41728.65 | 0.56 | 0.148 | 3.33E-11 |
| sp\|Q7Z4I7\|LIMS2_HUMAN | 40753.26 | 0.61 | 0.059 | 2.20E-16 |
| sp\|P07384\|CAN1_HUMAN | 82447.41 | 0.56 | 0.09 | 6.82E-16 |
| sp\|O15427\|MOT4_HUMAN | 50046.12 | 0.39 | 0.146 | 3.97E-12 |
| sp\|Q9H4L4\|SENP3_HUMAN | 65577.91 | 0.65 | 0.11 | 2.66E-12 |
| sp\|Q9Y5U9\|IR3IP_HUMAN | 9001.95 | 0.39 | 0.055 | 2.20E-16 |
| sp\|Q8IUH4\|ZDH13_HUMAN | 71936.55 | 0.46 | 0.108 | 3.05E-15 |
| sp\|O14828\|SCAM3_HUMAN | 38643.47 | 0.65 | 0.101 | 2.84E-13 |
| sp\|Q7L3T8\|SYPM_HUMAN | 54236.5 | 0.63 | 0.166 | 2.39E-09 |
| sp\|Q9HCG8\|CWC22_HUMAN | 105953.7 | 0.32 | 0.088 | 2.20E-16 |
| sp\|P11413\|G6PD_HUMAN | 59657.15 | 0.64 | 0.146 | 5.31E-10 |
| sp\|Q92979\|NEP1_HUMAN | 26913.23 | 0.57 | 0.066 | 2.20E-16 |
| sp\|Q00325\|MPCP_HUMAN | 40506.9 | 0.45 | 0.114 | 2.13E-14 |
| sp\|P62487\|RPB7_HUMAN | 19434.93 | 0.63 | 0.105 | 7.92E-13 |
| sp\|P10620\|MGST1_HUMAN | 17626.23 | 0.61 | 0.14 | 4.43E-11 |
| sp\|Q96IX5\|USMG5_HUMAN | 6492.384 | 0.48 | 0.091 | 2.20E-16 |
| sp\|Q8IXM6\|NRM_HUMAN | 29513.14 | 0.61 | 0.116 | 9.44E-13 |
| sp\|O00148\|DX39A_HUMAN | 49593.14 | 0.63 | 0.148 | 5.48E-10 |
| sp\|Q9GZP9\|DERL2_HUMAN | 27645.32 | 0.55 | 0.3 | 1.36E-06 |
| sp\|Q9UDW1\|QCR9_HUMAN | 7285.815 | 0.35 | 0.144 | 4.88E-12 |
| sp\|P42704\|LPPRC_HUMAN | 158984.6 | 0.57 | 0.148 | 8.25E-11 |
| sp\|Q969M3\|YIPF5_HUMAN | 28351.76 | 0.63 | 0.229 | 2.41E-07 |
| sp\|Q96CW5\|GCP3_HUMAN | 104286.4 | 0.5 | 0.069 | 2.20E-16 |
| sp\|P04844\|RPN2_HUMAN | 69337 | 0.65 | 0.102 | 2.81E-13 |
| sp\|Q99487\|PAFA2_HUMAN | 44559.21 | 0.57 | 0.135 | 1.23E-11 |
| sp\|Q96HE7\|ERO1A_HUMAN | 55195.36 | 0.66 | 0.128 | 3.16E-10 |
| sp\|P56134\|ATPK_HUMAN | 11006.73 | 0.58 | 0.215 | 6.29E-08 |
| sp\|Q7L1Q6\|BZW1_HUMAN | 48165.8 | 0.63 | 0.084 | 2.42E-15 |
| sp\|Q15437\|SC23B_HUMAN | 87374.87 | 0.65 | 0.163 | 1.85E-09 |
| sp\|Q9H3U1\|UN45A_HUMAN | 104248 | 0.57 | 0.088 | 8.54E-16 |
| sp\|Q15363\|TMED2_HUMAN | 22842.41 | 0.66 | 0.134 | 1.43E-10 |
| sp\|Q7L5N7\|PCAT2_HUMAN | 60778.62 | 0.59 | 0.053 | 2.20E-16 |
| sp\|O00462\|MANBA_HUMAN | 101782.2 | 0.61 | 0.187 | 1.71E-08 |
| sp\|P25788\|PSA3_HUMAN | 28625.16 | 0.66 | 0.096 | 1.88E-13 |
| sp\|Q6UW02\|CP20A_HUMAN | 52723.13 | 0.61 | 0.147 | 5.76E-11 |
| sp\|Q9Y4P3\|TBL2_HUMAN | 50375.42 | 0.66 | 0.128 | 4.09E-11 |
| sp\|Q9Y2T2\|AP3M1_HUMAN | 47233.47 | 0.57 | 0.098 | 1.43E-14 |
| sp\|Q9Y6D5\|BIG2_HUMAN | 204400.1 | 0.47 | 0.136 | 9.82E-14 |
| sp\|Q9H074\|PAIP1_HUMAN | 53929.27 | 0.57 | 0.099 | 3.70E-15 |
| sp\|O75503\|CLN5_HUMAN | 41850.77 | 0.66 | 0.129 | 5.77E-11 |
| sp\|P16152\|CBR1_HUMAN | 30622.96 | 0.53 | 0.158 | 3.98E-10 |
| sp\|Q96K37\|S35E1_HUMAN | 45067.86 | 0.61 | 0.539 | 1.10E-05 |
| sp\|Q3KQZ1\|S2535_HUMAN | 32626.65 | 0.41 | 0.178 | 3.10E-08 |
| sp\|Q96DM3\|RMC1_HUMAN | 75649.88 | 0.6 | 0.171 | 1.38E-09 |
| sp\|Q15172\|2A5A_HUMAN | 56482.27 | 0.66 | 0.098 | 2.27E-13 |
| sp\|Q8TAC1\|RFESD_HUMAN | 18074.75 | 0.63 | 0.269 | 5.01E-06 |
| sp\|P05451\|REG1A_HUMAN | 19099.92 | 0.51 | 0.269 | 2.96E-06 |
| sp\|P61278\|SMS_HUMAN | 12994.45 | 0.64 | 0.292 | 0.0002875 |
| sp\|Q9NX57\|RAB20_HUMAN | 26584.3 | 0.66 | 0.186 | 7.59E-08 |
| sp\|Q12996\|CSTF3_HUMAN | 83306.89 | 0.6 | 0.097 | 4.63E-14 |
| sp\|Q8NEZ5\|FBX22_HUMAN | 45260.07 | 0.55 | 0.109 | 1.36E-14 |
| sp\|Q9BW27\|NUP85_HUMAN | 75808.38 | 0.65 | 0.095 | 3.86E-14 |
| sp\|P23396\|RS3_HUMAN | 26824.48 | 0.59 | 0.09 | 5.85E-15 |
| sp\|O14980\|XPO1_HUMAN | 124428.5 | 0.56 | 0.063 | 2.20E-16 |
| sp\|Q96D53\|COQ8B_HUMAN | 60754.71 | 0.66 | 0.185 | 3.34E-08 |
| sp\|Q8WU67\|ABHD3_HUMAN | 46474.16 | 0.47 | 0.19 | 6.55E-10 |
| sp\|Q8N9V3\|WSDU1_HUMAN | 54076.4 | 0.65 | 0.151 | 9.47E-10 |
| sp\|P61326\|MGN_HUMAN | 17191.8 | 0.65 | 0.159 | 1.45E-09 |
| sp\|Q6PCE3\|PGM2L_HUMAN | 71347.83 | 0.51 | 0.059 | 2.20E-16 |
| sp\|Q8WWM9\|CYGB_HUMAN | 21487.01 | 0.66 | 0.123 | 7.20E-11 |
| sp\|Q8WUD6\|CHPT1_HUMAN | 45676.38 | 0.55 | 0.107 | 7.14E-14 |
| sp\|Q16585\|SGCB_HUMAN | 35078.77 | 0.66 | 0.132 | 5.46E-11 |
| sp\|Q13144\|EI2BE_HUMAN | 81052.58 | 0.64 | 0.273 | 2.82E-06 |
| sp\|P29992\|GNA11_HUMAN | 42363.61 | 0.57 | 0.141 | 1.15E-11 |
| sp\|Q9Y2G3\|AT11B_HUMAN | 135397.4 | 0.62 | 0.102 | 1.16E-13 |
| sp\|P61626\|LYSC_HUMAN | 16964.45 | 0.55 | 0.222 | 2.37E-07 |
| sp\|Q9NVV4\|PAPD1_HUMAN | 66967.31 | 0.63 | 0.168 | 8.08E-09 |
| sp\|Q8NG11\|TSN14_HUMAN | 31336.82 | 0.52 | 0.118 | 3.42E-13 |
| sp\|Q9NQG6\|MID51_HUMAN | 51698.84 | 0.65 | 0.132 | 1.12E-10 |
| sp\|Q6UXY8\|TMC5_HUMAN | 115617.9 | 0.61 | 0.207 | 6.21E-08 |
| sp\|P11678\|PERE_HUMAN | 81940.49 | 0.47 | 0.154 | 4.69E-10 |
| sp\|Q9NP58\|ABCB6_HUMAN | 94378.36 | 0.64 | 0.068 | 2.20E-16 |
| sp\|Q9NXN4\|GDAP2_HUMAN | 56570.6 | 0.64 | 0.081 | 6.11E-16 |
| sp\|P03905\|NU4M_HUMAN | 51699.61 | 0.52 | 0.158 | 3.75E-10 |
| sp\|P20336\|RAB3A_HUMAN | 25178.17 | 0.31 | 0.139 | 4.40E-12 |
| sp\|Q9H583\|HEAT1_HUMAN | 244364.1 | 0.57 | 0.117 | 1.13E-12 |
| sp\|Q9NZ08\|ERAP1_HUMAN | 107718.5 | 0.53 | 0.168 | 2.11E-10 |
| sp\|Q9H479\|FN3K_HUMAN | 35358.94 | 0.62 | 0.182 | 2.06E-08 |
| sp\|Q9BVK2\|ALG8_HUMAN | 60771.23 | 0.61 | 0.146 | 1.38E-10 |
| sp\|P08263\|GSTA1_HUMAN | 25653.67 | 0.62 | 0.36 | 0.0001066 |
| sp\|Q14642\|I5P1_HUMAN | 48340.92 | 0.63 | 0.123 | 3.16E-12 |
| sp\|Q96EK7\|F120B_HUMAN | 105750.8 | 0.47 | 0.137 | 1.21E-12 |
| sp\|P54760\|EPHB4_HUMAN | 109722.9 | 0.53 | 0.088 | 6.17E-16 |
| sp\|Q86SZ2\|TPC6B_HUMAN | 18181.29 | 0.52 | 0.057 | 2.20E-16 |
| sp\|Q9UNX4\|WDR3_HUMAN | 107097.2 | 0.62 | 0.111 | 1.69E-12 |
| sp\|Q99808\|S29A1_HUMAN | 50738.56 | 0.65 | 0.194 | 9.22E-08 |
| sp\|P04066\|FUCO_HUMAN | 53921.82 | 0.51 | 0.146 | 5.09E-11 |
| sp\|Q12999\|TSN31_HUMAN | 23647.07 | 0.41 | 0.178 | 3.64E-10 |
| sp\|P20020\|AT2B1_HUMAN | 135551.2 | 0.55 | 0.105 | 3.14E-14 |
| sp\|Q8NCS4\|TM35B_HUMAN | 16969.44 | 0.23 | 0.058 | 2.20E-16 |
| sp\|Q9P0V3\|SH3B4_HUMAN | 108379.4 | 0.55 | 0.191 | 5.06E-10 |
| sp\|P36871\|PGM1_HUMAN | 61677.63 | 0.55 | 0.091 | 6.31E-16 |
| sp\|P62068\|UBP46_HUMAN | 43138.39 | 0.39 | 0.076 | 2.20E-16 |
| sp\|P27105\|STOM_HUMAN | 31863.79 | 0.62 | 0.133 | 3.11E-11 |
| sp\|Q6NUQ4\|TM214_HUMAN | 77938.76 | 0.61 | 0.117 | 1.38E-12 |
| sp\|Q9BXP2\|S12A9_HUMAN | 96714.87 | 0.27 | 0.132 | 2.09E-14 |
| sp\|P28288\|ABCD3_HUMAN | 75922.76 | 0.5 | 0.142 | 1.12E-12 |
| sp\|O95994\|AGR2_HUMAN | 20005.63 | 0.63 | 0.324 | 6.91E-05 |
| sp\|P21796\|VDAC1_HUMAN | 30849.6 | 0.54 | 0.189 | 7.14E-10 |
| sp\|Q9Y6R7\|FCGBP_HUMAN | 596425.2 | 0.54 | 0.221 | 6.48E-07 |
| sp\|P79483\|DRB3_HUMAN | 30210.26 | 0.51 | 0.236 | 3.03E-08 |
| sp\|Q9Y3A6\|TMED5_HUMAN | 26084.22 | 0.59 | 0.141 | 1.62E-11 |
| sp\|P35749\|MYH11_HUMAN | 228036.2 | 0.49 | 0.214 | 1.70E-08 |
| sp\|Q9P2K6\|KLH42_HUMAN | 57440.64 | 0.54 | 0.158 | 1.76E-10 |
| sp\|Q02094\|RHAG_HUMAN | 44435.78 | 0.36 | 0.108 | 1.43E-15 |
| sp\|O43813\|LANC1_HUMAN | 45976.84 | 0.61 | 0.145 | 9.47E-12 |
| sp\|Q8WWC4\|MAIP1_HUMAN | 32905.13 | 0.5 | 0.111 | 2.14E-14 |
| sp\|Q7Z7H5\|TMED4_HUMAN | 26079.39 | 0.61 | 0.19 | 7.62E-09 |
| sp\|O15484\|CAN5_HUMAN | 74074.01 | 0.55 | 0.091 | 2.27E-15 |
| sp\|O15439\|MRP4_HUMAN | 150326 | 0.58 | 0.131 | 5.16E-12 |
| sp\|Q8WY22\|BRI3B_HUMAN | 27913.87 | 0.35 | 0.12 | 3.98E-14 |
| sp\|Q9Y2Q0\|AT8A1_HUMAN | 132579 | 0.62 | 0.154 | 8.24E-10 |
| sp\|P0DJD8\|PEPA3_HUMAN | 42330.74 | 0.64 | 0.209 | 1.66E-06 |
| sp\|P00403\|COX2_HUMAN | 25701.27 | 0.62 | 0.182 | 1.01E-08 |
| sp\|Q969E2\|SCAM4_HUMAN | 26034.85 | 0.63 | 0.133 | 1.14E-10 |
| sp\|P51606\|RENBP_HUMAN | 49465.29 | 0.62 | 0.113 | 1.52E-12 |
| sp\|O00391\|QSOX1_HUMAN | 83305.8 | 0.58 | 0.165 | 5.25E-10 |
| sp\|Q9UBX3\|DIC_HUMAN | 31700.49 | 0.63 | 0.169 | 1.41E-09 |
| sp\|Q8WTT2\|NOC3L_HUMAN | 92927.73 | 0.61 | 0.112 | 1.27E-12 |
| sp\|Q9NUS5\|AP5S1_HUMAN | 22660.95 | 0.66 | 0.084 | 1.05E-14 |
| sp\|Q9UNA1\|RHG26_HUMAN | 92899.84 | 0.63 | 0.104 | 6.25E-13 |
| sp\|P0CK96\|S352B_HUMAN | 44073.15 | 0.48 | 0.152 | 6.39E-12 |
| sp\|O43451\|MGA_HUMAN | 211013.1 | 0.61 | 0.311 | 5.36E-06 |
| sp\|Q93008\|USP9X_HUMAN | 295496.9 | 0.62 | 0.057 | 2.20E-16 |
| sp\|P20340\|RAB6A_HUMAN | 23673.95 | 0.61 | 0.075 | 2.20E-16 |
| sp\|Q9Y2A7\|NCKP1_HUMAN | 130000 | 0.49 | 0.08 | 2.20E-16 |
| sp\|Q7Z3U7\|MON2_HUMAN | 192329.1 | 0.49 | 0.07 | 2.20E-16 |
| sp\|P16233\|LIPP_HUMAN | 51904.57 | 0.5 | 0.215 | 1.93E-09 |
| sp\|Q9P2R3\|ANFY1_HUMAN | 129896.7 | 0.64 | 0.104 | 2.38E-13 |
| sp\|Q9Y2D2\|S35A3_HUMAN | 36114.34 | 0.47 | 0.189 | 5.18E-11 |
| sp\|P37268\|FDFT_HUMAN | 48579.38 | 0.64 | 0.192 | 6.09E-07 |
| sp\|P51648\|AL3A2_HUMAN | 55251.05 | 0.61 | 0.165 | 1.34E-08 |
| sp\|Q8TCJ2\|STT3B_HUMAN | 94222.9 | 0.61 | 0.093 | 2.93E-14 |
| sp\|P54709\|AT1B3_HUMAN | 31816.21 | 0.65 | 0.104 | 7.12E-13 |
| sp\|Q6PI78\|TMM65_HUMAN | 25748.55 | 0.43 | 0.099 | 2.33E-15 |
| sp\|P53618\|COPB_HUMAN | 108196.2 | 0.62 | 0.08 | 1.42E-15 |
| sp\|Q15746\|MYLK_HUMAN | 213301.8 | 0.61 | 0.256 | 3.36E-07 |
| sp\|Q8N2U0\|TM256_HUMAN | 11830.17 | 0.52 | 0.121 | 1.60E-13 |
| sp\|P04114\|APOB_HUMAN | 516633.4 | 0.52 | 0.222 | 4.69E-08 |
| sp\|Q15631\|TSN_HUMAN | 26262.81 | 0.55 | 0.072 | 2.20E-16 |
| sp\|Q9NWD8\|TM248_HUMAN | 35467.67 | 0.56 | 0.407 | 4.21E-05 |
| sp\|Q7L576\|CYFP1_HUMAN | 146724.3 | 0.6 | 0.099 | 3.72E-14 |
| sp\|O00442\|RTCA_HUMAN | 39806.59 | 0.45 | 0.075 | 2.20E-16 |
| sp\|Q6P3X3\|TTC27_HUMAN | 97864.71 | 0.5 | 0.072 | 2.20E-16 |
| sp\|Q96NY7\|CLIC6_HUMAN | 73177.79 | 0.64 | 0.152 | 1.77E-09 |
| sp\|P40616\|ARL1_HUMAN | 20500.47 | 0.41 | 0.129 | 1.47E-13 |
| sp\|Q9UHQ9\|NB5R1_HUMAN | 34226.24 | 0.6 | 0.131 | 3.85E-11 |
| sp\|Q14108\|SCRB2_HUMAN | 54693.62 | 0.49 | 0.16 | 6.71E-11 |
| sp\|P61586\|RHOA_HUMAN | 22078.19 | 0.66 | 0.086 | 9.09E-15 |
| sp\|Q9Y487\|VPP2_HUMAN | 99083.74 | 0.6 | 0.103 | 4.24E-14 |
| sp\|Q8TAG9\|EXOC6_HUMAN | 94385.85 | 0.65 | 0.111 | 2.41E-12 |
| sp\|Q8TBH0\|ARRD2_HUMAN | 44675.32 | 0.48 | 0.217 | 3.08E-08 |
| sp\|Q9HBH0\|RHOF_HUMAN | 23991.36 | 0.58 | 0.081 | 4.13E-16 |
| sp\|Q3LXA3\|TKFC_HUMAN | 59234.15 | 0.63 | 0.158 | 1.74E-09 |
| sp\|Q96A57\|TM230_HUMAN | 13162.07 | 0.59 | 0.341 | 1.78E-06 |
| sp\|O75844\|FACE1_HUMAN | 55044.63 | 0.53 | 0.099 | 1.23E-14 |
| sp\|Q15125\|EBP_HUMAN | 26545.83 | 0.63 | 0.101 | 2.06E-13 |
| sp\|P08648\|ITA5_HUMAN | 115587.2 | 0.63 | 0.243 | 2.15E-06 |
| sp\|Q9GZM5\|YIPF3_HUMAN | 38432.66 | 0.53 | 0.142 | 1.46E-12 |
| sp\|Q12933\|TRAF2_HUMAN | 57572.35 | 0.62 | 0.091 | 9.98E-15 |
| sp\|P31327\|CPSM_HUMAN | 165957.2 | 0.54 | 0.276 | 1.62E-06 |
| sp\|Q8TB36\|GDAP1_HUMAN | 41529.7 | 0.51 | 0.128 | 1.96E-13 |
| sp\|Q9UBD5\|ORC3_HUMAN | 83266.85 | 0.66 | 0.19 | 2.56E-07 |
| sp\|Q96F07\|CYFP2_HUMAN | 150280.1 | 0.65 | 0.139 | 1.77E-10 |
| sp\|O15042\|SR140_HUMAN | 118656.8 | 0.65 | 0.071 | 2.20E-16 |
| sp\|Q9Y394\|DHRS7_HUMAN | 38655.26 | 0.46 | 0.125 | 2.54E-12 |
| sp\|Q9H9A6\|LRC40_HUMAN | 68702.31 | 0.64 | 0.086 | 1.61E-14 |
| sp\|E7EW31\|PROB1_HUMAN | 107289.9 | 0.48 | 0.273 | 6.20E-07 |
| sp\|P16050\|LOX15_HUMAN | 75479.78 | 0.58 | 0.186 | 3.91E-08 |
| sp\|O00232\|PSD12_HUMAN | 53251.71 | 0.55 | 0.087 | 2.49E-16 |
| sp\|O75131\|CPNE3_HUMAN | 60929.44 | 0.59 | 0.105 | 2.40E-13 |
| sp\|Q9NZD2\|GLTP_HUMAN | 23987.48 | 0.52 | 0.098 | 6.82E-15 |
| sp\|Q3LI76\|KR151_HUMAN | 15805.72 | 0.6 | 0.065 | 2.20E-16 |
| sp\|Q96E17\|RAB3C_HUMAN | 26145.7 | 0.46 | 0.183 | 6.26E-10 |
| sp\|Q9H7F0\|AT133_HUMAN | 139759.7 | 0.61 | 0.245 | 6.83E-08 |
| sp\|Q5H9R7\|PP6R3_HUMAN | 98558.99 | 0.49 | 0.103 | 9.05E-15 |
| sp\|Q7Z449\|CP2U1_HUMAN | 62385.47 | 0.57 | 0.138 | 1.84E-11 |
| sp\|Q8NCL4\|GALT6_HUMAN | 72007.45 | 0.49 | 0.277 | 1.79E-06 |
| sp\|Q9UFH2\|DYH17_HUMAN | 512732.6 | 0.56 | 0.281 | 3.34E-06 |
| sp\|Q9H269\|VPS16_HUMAN | 95641.95 | 0.65 | 0.095 | 9.26E-14 |
| sp\|Q8NE01\|CNNM3_HUMAN | 76510.22 | 0.61 | 0.132 | 7.59E-12 |
| sp\|P08247\|SYPH_HUMAN | 34090.53 | 0.66 | 0.197 | 1.89E-06 |
| sp\|P56192\|SYMC_HUMAN | 102231.3 | 0.62 | 0.115 | 2.59E-12 |
| sp\|Q15043\|S39AE_HUMAN | 54900.48 | 0.65 | 0.183 | 5.47E-09 |
| sp\|P45877\|PPIC_HUMAN | 22844.88 | 0.49 | 0.164 | 5.14E-12 |
| sp\|Q7Z3V4\|UBE3B_HUMAN | 124483.7 | 0.57 | 0.096 | 8.97E-15 |
| sp\|Q96DG6\|CMBL_HUMAN | 28354.29 | 0.62 | 0.354 | 3.68E-05 |
| sp\|Q6GMV2\|SMYD5_HUMAN | 48546.4 | 0.57 | 0.201 | 1.51E-08 |
| sp\|P42285\|MTREX_HUMAN | 118737.8 | 0.58 | 0.079 | 2.20E-16 |
| sp\|O95379\|TFIP8_HUMAN | 23141.92 | 0.52 | 0.17 | 6.49E-11 |
| sp\|Q9H1I8\|ASCC2_HUMAN | 87029.54 | 0.45 | 0.067 | 2.20E-16 |
| sp\|Q9UKU6\|TRHDE_HUMAN | 117421 | 0.53 | 0.339 | 1.17E-06 |
| sp\|Q9UNQ2\|DIM1_HUMAN | 35481.19 | 0.66 | 0.125 | 1.82E-11 |
| sp\|Q7L5Y1\|ENOF1_HUMAN | 50363.57 | 0.61 | 0.188 | 2.15E-09 |
| sp\|Q06141\|REG3A_HUMAN | 19763.5 | 0.5 | 0.243 | 4.26E-06 |
| sp\|Q9P0S3\|ORML1_HUMAN | 17342.16 | 0.59 | 0.213 | 7.00E-09 |
| sp\|Q14457\|BECN1_HUMAN | 52358.62 | 0.57 | 0.095 | 2.61E-15 |
| sp\|Q5VYK3\|ECM29_HUMAN | 205967.1 | 0.66 | 0.095 | 1.61E-13 |
| sp\|Q06210\|GFPT1_HUMAN | 79536.61 | 0.55 | 0.123 | 3.24E-13 |
| sp\|Q8IWA4\|MFN1_HUMAN | 84886.45 | 0.49 | 0.117 | 3.40E-13 |
| sp\|Q9HD45\|TM9S3_HUMAN | 68566.34 | 0.44 | 0.159 | 1.61E-11 |
| sp\|Q9NUT2\|ABCB8_HUMAN | 80775.16 | 0.64 | 0.121 | 1.06E-11 |
| sp\|Q15269\|PWP2_HUMAN | 103338.6 | 0.54 | 0.186 | 2.92E-09 |
| sp\|O95573\|ACSL3_HUMAN | 81319.65 | 0.49 | 0.071 | 2.20E-16 |
| sp\|Q8WZ82\|OVCA2_HUMAN | 24726.65 | 0.66 | 0.117 | 5.93E-12 |
| sp\|Q16531\|DDB1_HUMAN | 128123.8 | 0.55 | 0.071 | 2.20E-16 |
| sp\|P26639\|SYTC_HUMAN | 84276.2 | 0.61 | 0.117 | 8.76E-13 |
| sp\|Q15147\|PLCB4_HUMAN | 135500.4 | 0.65 | 0.219 | 5.75E-08 |
| sp\|P07099\|HYEP_HUMAN | 53125.04 | 0.66 | 0.12 | 1.52E-11 |
| sp\|P61006\|RAB8A_HUMAN | 23806.24 | 0.54 | 0.103 | 6.22E-15 |
| sp\|Q8NEW0\|ZNT7_HUMAN | 41923.44 | 0.42 | 0.097 | 4.67E-16 |
| sp\|Q9Y6H3\|ATP23_HUMAN | 28671.92 | 0.66 | 0.211 | 1.16E-07 |
| sp\|Q8NHG7\|SVIP_HUMAN | 8533.454 | 0.48 | 0.163 | 3.77E-11 |
| sp\|P30566\|PUR8_HUMAN | 55577.24 | 0.62 | 0.093 | 1.63E-14 |
| sp\|P08754\|GNAI3_HUMAN | 41058.47 | 0.5 | 0.087 | 2.20E-16 |
| sp\|O95716\|RAB3D_HUMAN | 24461.89 | 0.33 | 0.116 | 5.05E-13 |
| sp\|Q9NR19\|ACSA_HUMAN | 79594.53 | 0.63 | 0.109 | 3.49E-13 |
| sp\|Q9UHQ4\|BAP29_HUMAN | 28398.21 | 0.64 | 0.088 | 6.74E-15 |
| sp\|P15291\|B4GT1_HUMAN | 44273.47 | 0.6 | 0.125 | 6.73E-12 |
| sp\|P03915\|NU5M_HUMAN | 67306.55 | 0.43 | 0.185 | 5.62E-11 |
| sp\|Q9H9Q4\|NHEJ1_HUMAN | 33697.09 | 0.54 | 0.14 | 3.65E-12 |
| sp\|Q8IV63\|VRK3_HUMAN | 53570.93 | 0.64 | 0.156 | 9.29E-10 |
| sp\|A2A3L6\|TTC24_HUMAN | 64023.41 | 0.65 | 0.151 | 3.10E-09 |
| sp\|O60318\|GANP_HUMAN | 220644.2 | 0.62 | 0.099 | 1.17E-13 |
| sp\|Q96N66\|MBOA7_HUMAN | 53396.51 | 0.43 | 0.07 | 2.20E-16 |
| sp\|Q5JRK9\|GGEE3_HUMAN | 12015.67 | 0.64 | 0.466 | 0.0003813 |
| sp\|P40763\|STAT3_HUMAN | 88791.67 | 0.63 | 0.093 | 1.84E-14 |
| sp\|Q6XQN6\|PNCB_HUMAN | 58094.22 | 0.54 | 0.142 | 6.55E-12 |
| sp\|P22670\|RFX1_HUMAN | 105017 | 0.6 | 0.069 | 2.20E-16 |
| sp\|Q9Y5Q8\|TF3C5_HUMAN | 59971.26 | 0.63 | 0.098 | 6.01E-14 |
| sp\|Q9NZJ7\|MTCH1_HUMAN | 41841.41 | 0.52 | 0.116 | 7.56E-14 |
| sp\|Q8IXI2\|MIRO1_HUMAN | 71747.28 | 0.63 | 0.075 | 5.90E-16 |
| sp\|P11388\|TOP2A_HUMAN | 174999 | 0.66 | 0.252 | 3.28E-06 |
| sp\|Q9H6V9\|LDAH_HUMAN | 37732.34 | 0.61 | 0.092 | 1.12E-14 |
| sp\|P78381\|S35A2_HUMAN | 41434.58 | 0.43 | 0.123 | 4.84E-14 |
| sp\|Q7KZN9\|COX15_HUMAN | 46324.54 | 0.61 | 0.153 | 2.21E-10 |
| sp\|Q9NQH7\|XPP3_HUMAN | 57606.07 | 0.61 | 0.12 | 1.26E-12 |
| sp\|O96005\|CLPT1_HUMAN | 76258.54 | 0.51 | 0.153 | 5.25E-10 |
| sp\|P08473\|NEP_HUMAN | 86126.12 | 0.62 | 0.256 | 2.28E-06 |
| sp\|P08237\|PFKAM_HUMAN | 85965.7 | 0.61 | 0.11 | 7.73E-13 |
| sp\|P05141\|ADT2_HUMAN | 33041.23 | 0.45 | 0.125 | 4.68E-14 |
| sp\|P35443\|TSP4_HUMAN | 108464 | 0.6 | 0.206 | 3.47E-08 |
| sp\|O43292\|GPAA1_HUMAN | 67904.08 | 0.57 | 0.104 | 1.45E-13 |
| sp\|P49961\|ENTP1_HUMAN | 58536.33 | 0.58 | 0.11 | 1.58E-13 |
| sp\|Q5SQS7\|SH24B_HUMAN | 51296.29 | 0.51 | 0.075 | 2.20E-16 |
| sp\|P29762\|RABP1_HUMAN | 15708.8 | 0.65 | 0.288 | 4.59E-05 |
| sp\|Q9Y295\|DRG1_HUMAN | 40783.92 | 0.64 | 0.142 | 4.18E-10 |
| sp\|P24043\|LAMA2_HUMAN | 352960.1 | 0.65 | 0.136 | 7.00E-10 |
| sp\|Q9UNM6\|PSD13_HUMAN | 43185.24 | 0.64 | 0.071 | 2.20E-16 |
| sp\|Q9UM54\|MYO6_HUMAN | 150947 | 0.64 | 0.213 | 8.60E-08 |
| sp\|Q8NHH9\|ATLA2_HUMAN | 66796.35 | 0.58 | 0.115 | 1.22E-13 |
| sp\|O00410\|IPO5_HUMAN | 125014.4 | 0.57 | 0.066 | 2.20E-16 |
| sp\|P05496\|AT5G1_HUMAN | 14420.51 | 0.18 | 0.088 | 2.20E-16 |
| sp\|P53041\|PPP5_HUMAN | 57394.45 | 0.54 | 0.102 | 3.89E-14 |
| sp\|P51571\|SSRD_HUMAN | 19139.66 | 0.6 | 0.058 | 2.20E-16 |
| sp\|Q8WV92\|MITD1_HUMAN | 29620.07 | 0.63 | 0.082 | 1.35E-15 |
| sp\|P52209\|6PGD_HUMAN | 53601.14 | 0.59 | 0.095 | 1.14E-14 |
| sp\|P26196\|DDX6_HUMAN | 54763.45 | 0.63 | 0.094 | 1.01E-13 |
| sp\|Q9BV36\|MELPH_HUMAN | 66575.09 | 0.65 | 0.219 | 3.00E-06 |
| sp\|Q5T1V6\|DDX59_HUMAN | 69432.6 | 0.52 | 0.15 | 1.05E-11 |
| sp\|Q96JG6\|VPS50_HUMAN | 111941.2 | 0.56 | 0.106 | 2.21E-14 |
| sp\|P01019\|ANGT_HUMAN | 53387.61 | 0.6 | 0.172 | 4.25E-10 |
| sp\|Q14435\|GALT3_HUMAN | 73401.18 | 0.42 | 0.236 | 1.36E-09 |
| sp\|Q9HBG6\|IF122_HUMAN | 143769.3 | 0.64 | 0.154 | 1.54E-09 |
| sp\|Q9H920\|RN121_HUMAN | 38295.42 | 0.51 | 0.127 | 3.75E-12 |
| sp\|Q9HAR2\|AGRL3_HUMAN | 163573.2 | 0.64 | 0.224 | 1.04E-07 |
| sp\|O43861\|ATP9B_HUMAN | 130741.5 | 0.61 | 0.128 | 9.50E-12 |
| sp\|Q09161\|NCBP1_HUMAN | 92845.92 | 0.48 | 0.092 | 6.76E-16 |
| sp\|O75691\|UTP20_HUMAN | 320787.1 | 0.61 | 0.102 | 9.97E-14 |
| sp\|Q96SQ9\|CP2S1_HUMAN | 55991.31 | 0.59 | 0.161 | 2.30E-09 |
| sp\|P43007\|SATT_HUMAN | 56068.77 | 0.34 | 0.158 | 6.34E-13 |
| sp\|Q02742\|GCNT1_HUMAN | 50261.69 | 0.6 | 0.227 | 9.44E-07 |
| sp\|Q9Y673\|ALG5_HUMAN | 37132.45 | 0.6 | 0.137 | 3.05E-11 |
| sp\|Q9Y6E2\|BZW2_HUMAN | 48342.05 | 0.55 | 0.116 | 1.68E-13 |
| sp\|P21359\|NF1_HUMAN | 322741.9 | 0.44 | 0.08 | 2.20E-16 |
| sp\|P54855\|UDB15_HUMAN | 61491.51 | 0.62 | 0.242 | 1.30E-05 |
| sp\|O14732\|IMPA2_HUMAN | 31739.22 | 0.62 | 0.125 | 2.19E-11 |
| sp\|O95858\|TSN15_HUMAN | 33866.11 | 0.53 | 0.207 | 1.29E-08 |
| sp\|Q92832\|NELL1_HUMAN | 94687.86 | 0.65 | 0.063 | 2.20E-16 |
| sp\|P43358\|MAGA4_HUMAN | 35087.54 | 0.48 | 0.22 | 8.08E-08 |
| sp\|O60760\|HPGDS_HUMAN | 23481.92 | 0.56 | 0.19 | 8.61E-10 |
| sp\|P62314\|SMD1_HUMAN | 13255.35 | 0.62 | 0.127 | 1.63E-12 |
| sp\|Q9NS86\|LANC2_HUMAN | 51658.75 | 0.66 | 0.079 | 3.68E-15 |
| sp\|Q09328\|MGT5A_HUMAN | 85610.35 | 0.38 | 0.159 | 2.69E-12 |
| sp\|P08240\|SRPRA_HUMAN | 70205.4 | 0.65 | 0.106 | 2.73E-12 |
| sp\|O95070\|YIF1A_HUMAN | 32029.53 | 0.42 | 0.122 | 1.96E-14 |
| sp\|Q9C0I1\|MTMRC_HUMAN | 87158.18 | 0.61 | 0.087 | 3.09E-15 |
| sp\|Q92747\|ARC1A_HUMAN | 42094.89 | 0.59 | 0.122 | 7.85E-12 |
| sp\|Q8N4A0\|GALT4_HUMAN | 67460.82 | 0.59 | 0.101 | 8.82E-14 |
| sp\|Q9H8M1\|CQ10B_HUMAN | 27491.2 | 0.63 | 0.083 | 2.04E-15 |
| sp\|Q9Y617\|SERC_HUMAN | 40777.95 | 0.53 | 0.243 | 2.97E-08 |
| sp\|P38435\|VKGC_HUMAN | 88057.05 | 0.64 | 0.125 | 3.45E-11 |
| sp\|P20061\|TCO1_HUMAN | 48671.2 | 0.55 | 0.112 | 1.67E-13 |
| sp\|Q9HCJ1\|ANKH_HUMAN | 54700.6 | 0.64 | 0.457 | 9.20E-05 |
| sp\|Q00765\|REEP5_HUMAN | 21689.12 | 0.55 | 0.124 | 1.19E-12 |
| sp\|Q9HCL2\|GPAT1_HUMAN | 94743.85 | 0.49 | 0.088 | 2.20E-16 |
| sp\|A0A0B4J248\|TVA11_HUMAN | 12014.65 | 0.62 | 0.149 | 2.35E-10 |
| sp\|Q9Y277\|VDAC3_HUMAN | 30963.4 | 0.34 | 0.076 | 2.20E-16 |
| sp\|Q9NX02\|NALP2_HUMAN | 122871.2 | 0.65 | 0.265 | 3.34E-05 |
| sp\|Q9ULS5\|TMCC3_HUMAN | 54246.78 | 0.48 | 0.119 | 8.24E-14 |
| sp\|P51809\|VAMP7_HUMAN | 25243.09 | 0.52 | 0.121 | 2.62E-13 |
| sp\|Q2KHT3\|CL16A_HUMAN | 118591.8 | 0.57 | 0.158 | 3.02E-10 |
| sp\|Q9Y679\|AUP1_HUMAN | 46196.18 | 0.66 | 0.102 | 9.84E-13 |
| sp\|Q96QK1\|VPS35_HUMAN | 92429.22 | 0.56 | 0.052 | 2.20E-16 |
| sp\|Q9P2P5\|HECW2_HUMAN | 176611.6 | 0.66 | 0.377 | 0.0001257 |
| sp\|Q13505\|MTX1_HUMAN | 51640.65 | 0.63 | 0.243 | 5.68E-07 |
| sp\|Q86X10\|RLGPB_HUMAN | 168441.9 | 0.61 | 0.08 | 2.51E-16 |
| sp\|Q9H4A4\|AMPB_HUMAN | 73215.71 | 0.63 | 0.101 | 2.19E-13 |
| sp\|Q9UP83\|COG5_HUMAN | 93122.96 | 0.65 | 0.073 | 2.88E-16 |
| sp\|Q9NZ45\|CISD1_HUMAN | 12344.26 | 0.6 | 0.142 | 3.86E-11 |
| sp\|O43592\|XPOT_HUMAN | 111129.8 | 0.48 | 0.066 | 2.20E-16 |
| sp\|P59998\|ARPC4_HUMAN | 19750.34 | 0.64 | 0.105 | 3.87E-13 |
| sp\|Q96CN7\|ISOC1_HUMAN | 32483.22 | 0.61 | 0.131 | 3.56E-11 |
| sp\|Q5TH69\|BIG3_HUMAN | 243844.3 | 0.66 | 0.168 | 3.00E-08 |
| sp\|P00326\|ADH1G_HUMAN | 40678.99 | 0.6 | 0.379 | 0.0001278 |
| sp\|O60287\|NPA1P_HUMAN | 256490 | 0.66 | 0.109 | 3.50E-12 |
| sp\|P05023\|AT1A1_HUMAN | 114117.4 | 0.55 | 0.123 | 2.05E-13 |
| sp\|Q6P1Q0\|LTMD1_HUMAN | 42144.57 | 0.5 | 0.134 | 3.62E-13 |
| sp\|Q9BV23\|ABHD6_HUMAN | 38743.87 | 0.59 | 0.097 | 1.03E-14 |
| sp\|O95470\|SGPL1_HUMAN | 64034.86 | 0.6 | 0.13 | 1.16E-11 |
| sp\|P05546\|HEP2_HUMAN | 57187.26 | 0.64 | 0.215 | 2.24E-07 |
| sp\|Q03113\|GNA12_HUMAN | 44404.44 | 0.54 | 0.178 | 1.44E-10 |
| sp\|O76095\|JTB_HUMAN | 16956.55 | 0.48 | 0.221 | 1.59E-09 |
| sp\|Q6KCM7\|SCMC2_HUMAN | 52953.26 | 0.59 | 0.154 | 1.74E-10 |
| sp\|Q70J99\|UN13D_HUMAN | 124270.1 | 0.64 | 0.159 | 2.14E-09 |
| sp\|P15529\|MCP_HUMAN | 44727.06 | 0.59 | 0.087 | 3.70E-15 |
| sp\|Q7Z6B7\|SRGP1_HUMAN | 125080.7 | 0.59 | 0.15 | 3.15E-10 |
| sp\|P14735\|IDE_HUMAN | 118673.6 | 0.66 | 0.145 | 6.09E-10 |
| sp\|Q16563\|SYPL1_HUMAN | 28870.82 | 0.48 | 0.074 | 2.20E-16 |
| sp\|Q15031\|SYLM_HUMAN | 102805.3 | 0.59 | 0.177 | 5.52E-10 |
| sp\|Q07812\|BAX_HUMAN | 21266.79 | 0.55 | 0.08 | 2.20E-16 |
| sp\|P47712\|PA24A_HUMAN | 85679.4 | 0.65 | 0.159 | 1.44E-08 |
| sp\|O15260\|SURF4_HUMAN | 30583.86 | 0.63 | 0.05 | 2.20E-16 |
| sp\|O95407\|TNF6B_HUMAN | 33837.74 | 0.59 | 0.145 | 5.17E-11 |
| sp\|Q9UKV8\|AGO2_HUMAN | 98382.34 | 0.56 | 0.07 | 2.20E-16 |
| sp\|Q9Y6N5\|SQOR_HUMAN | 50195.95 | 0.62 | 0.156 | 2.00E-10 |
| sp\|Q99766\|ATP5S_HUMAN | 25458.8 | 0.35 | 0.086 | 2.20E-16 |
| sp\|Q6IA17\|SIGIR_HUMAN | 46202.68 | 0.6 | 0.093 | 1.07E-14 |
| sp\|O60921\|HUS1_HUMAN | 32051.58 | 0.62 | 0.145 | 4.10E-10 |
| sp\|P00505\|AATM_HUMAN | 47868.46 | 0.56 | 0.126 | 1.41E-12 |
| sp\|Q9UBM7\|DHCR7_HUMAN | 55177.03 | 0.53 | 0.182 | 8.89E-10 |
| sp\|P21926\|CD9_HUMAN | 25951.18 | 0.39 | 0.131 | 6.79E-12 |
| sp\|Q08722\|CD47_HUMAN | 35571.88 | 0.55 | 0.081 | 2.20E-16 |
| sp\|Q9H1E5\|TMX4_HUMAN | 39251.8 | 0.6 | 0.162 | 2.54E-10 |
| sp\|P00338\|LDHA_HUMAN | 36932.46 | 0.54 | 0.108 | 2.07E-14 |
| sp\|P78346\|RPP30_HUMAN | 29683.82 | 0.45 | 0.191 | 8.03E-10 |
| sp\|Q9NZL4\|HPBP1_HUMAN | 40000.75 | 0.52 | 0.109 | 3.14E-14 |
| sp\|P21283\|VATC1_HUMAN | 44067.01 | 0.64 | 0.092 | 2.14E-14 |
| sp\|Q96JJ7\|TMX3_HUMAN | 52163.02 | 0.66 | 0.109 | 1.40E-12 |
| sp\|P21980\|TGM2_HUMAN | 78402.09 | 0.54 | 0.122 | 9.39E-13 |
| sp\|Q9Y5S1\|TRPV2_HUMAN | 86820.39 | 0.55 | 0.112 | 1.41E-13 |
| sp\|Q9BVK6\|TMED9_HUMAN | 27356.22 | 0.63 | 0.136 | 2.62E-11 |
| sp\|Q93084\|AT2A3_HUMAN | 115425.9 | 0.52 | 0.115 | 3.50E-13 |
| sp\|Q8N9U0\|TAC2N_HUMAN | 55744.53 | 0.46 | 0.207 | 8.63E-11 |
| sp\|P78368\|KC1G2_HUMAN | 47808.32 | 0.58 | 0.116 | 2.88E-13 |
| sp\|P45880\|VDAC2_HUMAN | 32041.73 | 0.48 | 0.122 | 1.93E-13 |
| sp\|Q7Z6K3\|PTAR1_HUMAN | 46700.13 | 0.46 | 0.096 | 4.86E-16 |
| sp\|O43149\|ZZEF1_HUMAN | 335749.4 | 0.66 | 0.062 | 2.20E-16 |
| sp\|A5YKK6\|CNOT1_HUMAN | 269087.8 | 0.61 | 0.067 | 2.20E-16 |
| sp\|P06400\|RB_HUMAN | 106929 | 0.64 | 0.262 | 2.31E-07 |
| sp\|P07195\|LDHB_HUMAN | 36882.24 | 0.63 | 0.169 | 2.61E-09 |
| sp\|Q5VYS4\|MEDAG_HUMAN | 34435.54 | 0.48 | 0.218 | 6.84E-09 |
| sp\|Q05655\|KPCD_HUMAN | 78634.32 | 0.59 | 0.128 | 1.44E-12 |
| sp\|Q6BDS2\|URFB1_HUMAN | 160564.2 | 0.64 | 0.246 | 5.64E-06 |
| sp\|Q04771\|ACVR1_HUMAN | 58295.11 | 0.56 | 0.195 | 3.82E-09 |
| sp\|Q96S97\|MYADM_HUMAN | 36030.58 | 0.57 | 0.2 | 1.48E-08 |
| sp\|Q9BX97\|PLVAP_HUMAN | 51114.34 | 0.61 | 0.185 | 7.30E-10 |
| sp\|O94915\|FRYL_HUMAN | 342159 | 0.6 | 0.126 | 1.18E-12 |
| sp\|Q13825\|AUHM_HUMAN | 35853.27 | 0.59 | 0.188 | 1.15E-08 |
| sp\|Q6L9W6\|B4GN3_HUMAN | 115341.5 | 0.65 | 0.129 | 4.20E-11 |
| sp\|P08118\|MSMB_HUMAN | 13465.31 | 0.49 | 0.255 | 2.31E-07 |
| sp\|P56556\|NDUA6_HUMAN | 15108.88 | 0.66 | 0.147 | 1.99E-09 |
| sp\|Q8N9N2\|ASCC1_HUMAN | 45975.79 | 0.62 | 0.091 | 7.49E-15 |
| sp\|Q02338\|BDH_HUMAN | 38513.6 | 0.5 | 0.187 | 7.80E-10 |
| sp\|O14657\|TOR1B_HUMAN | 38278.94 | 0.39 | 0.069 | 2.20E-16 |
| sp\|O15118\|NPC1_HUMAN | 144849.6 | 0.63 | 0.195 | 1.45E-08 |
| sp\|Q9NX76\|CKLF6_HUMAN | 20729.64 | 0.46 | 0.179 | 4.00E-10 |
| sp\|O95498\|VNN2_HUMAN | 59131.71 | 0.64 | 0.233 | 8.06E-07 |
| sp\|P0DI81\|TPC2A_HUMAN | 16416.05 | 0.62 | 0.072 | 2.20E-16 |
| sp\|Q969Z3\|MARC2_HUMAN | 38550.92 | 0.65 | 0.27 | 1.29E-05 |
| sp\|Q92604\|LGAT1_HUMAN | 43271.36 | 0.44 | 0.098 | 1.88E-15 |
| sp\|Q6IC98\|GRAM4_HUMAN | 66747.49 | 0.66 | 0.101 | 1.07E-12 |
| sp\|Q9NPH0\|PPA6_HUMAN | 49349.91 | 0.37 | 0.092 | 2.20E-16 |
| sp\|Q96G03\|PGM2_HUMAN | 68735.58 | 0.64 | 0.072 | 2.20E-16 |
| sp\|A0AVT1\|UBA6_HUMAN | 119188.5 | 0.66 | 0.082 | 6.13E-15 |
| sp\|Q8IYT8\|ULK2_HUMAN | 114258.1 | 0.55 | 0.087 | 7.81E-16 |
| sp\|O75694\|NU155_HUMAN | 156678.9 | 0.65 | 0.093 | 3.11E-14 |
| sp\|O75643\|U520_HUMAN | 245988.2 | 0.63 | 0.075 | 3.65E-16 |
| sp\|Q8TC44\|POC1B_HUMAN | 54186.29 | 0.47 | 0.075 | 2.20E-16 |
| sp\|Q9Y4A5\|TRRAP_HUMAN | 441747.9 | 0.63 | 0.05 | 2.20E-16 |
| sp\|Q99523\|SORT_HUMAN | 92960.83 | 0.49 | 0.092 | 5.30E-16 |
| sp\|P15088\|CBPA3_HUMAN | 48905.88 | 0.56 | 0.236 | 1.29E-08 |
| sp\|Q5VW32\|BROX_HUMAN | 46941.83 | 0.6 | 0.161 | 1.28E-10 |
| sp\|O14684\|PTGES_HUMAN | 17301.07 | 0.55 | 0.247 | 3.07E-08 |
| sp\|O94855\|SC24D_HUMAN | 114458 | 0.64 | 0.105 | 4.82E-13 |
| sp\|Q15907\|RB11B_HUMAN | 24569.52 | 0.52 | 0.054 | 2.20E-16 |
| sp\|Q7L8C5\|SYT13_HUMAN | 47464.44 | 0.6 | 0.189 | 2.16E-08 |
| sp\|Q9BVC6\|TM109_HUMAN | 26175.57 | 0.57 | 0.11 | 1.07E-13 |
| sp\|Q9H201\|EPN3_HUMAN | 68333.29 | 0.46 | 0.169 | 1.61E-11 |
| sp\|P08246\|ELNE_HUMAN | 29109.01 | 0.57 | 0.161 | 6.79E-10 |
| sp\|P13716\|HEM2_HUMAN | 36709.58 | 0.57 | 0.085 | 3.32E-16 |
| sp\|O14744\|ANM5_HUMAN | 73303.8 | 0.66 | 0.075 | 1.69E-15 |
| sp\|P49759\|CLK1_HUMAN | 57977.74 | 0.53 | 0.173 | 1.20E-09 |
| sp\|Q9H3Z4\|DNJC5_HUMAN | 22914.62 | 0.56 | 0.186 | 1.63E-09 |
| sp\|Q9H5U6\|ZCHC4_HUMAN | 60606.86 | 0.58 | 0.224 | 6.57E-09 |
| sp\|Q15758\|AAAT_HUMAN | 57000.41 | 0.48 | 0.248 | 1.50E-07 |
| sp\|Q6PGP7\|TTC37_HUMAN | 177466.6 | 0.65 | 0.131 | 9.22E-11 |
| sp\|Q8N6Q3\|CD177_HUMAN | 47911.05 | 0.57 | 0.246 | 9.94E-08 |
| sp\|P21291\|CSRP1_HUMAN | 21391.07 | 0.66 | 0.295 | 1.37E-05 |
| sp\|Q9H8J5\|MANS1_HUMAN | 47275.83 | 0.64 | 0.182 | 8.49E-08 |
| sp\|Q92544\|TM9S4_HUMAN | 75192.93 | 0.49 | 0.125 | 3.86E-14 |
| sp\|Q9Y6B7\|AP4B1_HUMAN | 84556.19 | 0.52 | 0.115 | 5.21E-14 |
| sp\|Q6VUC0\|AP2E_HUMAN | 46678.12 | 0.5 | 0.25 | 8.23E-09 |
| sp\|P12814\|ACTN1_HUMAN | 103544.8 | 0.58 | 0.139 | 5.76E-11 |
| sp\|P10515\|ODP2_HUMAN | 69448.26 | 0.6 | 0.127 | 1.53E-11 |
| sp\|Q93073\|SBP2L_HUMAN | 122765.5 | 0.63 | 0.186 | 6.65E-09 |
| sp\|P78527\|PRKDC_HUMAN | 473730.8 | 0.58 | 0.094 | 1.44E-14 |
| sp\|Q53GD3\|CTL4_HUMAN | 80722.97 | 0.65 | 0.452 | 8.92E-06 |
| sp\|Q9UKA2\|FBXL4_HUMAN | 71629.72 | 0.53 | 0.147 | 1.55E-11 |
| sp\|Q15526\|SURF1_HUMAN | 33463.45 | 0.51 | 0.103 | 2.93E-14 |
| sp\|Q9H9E3\|COG4_HUMAN | 89977.76 | 0.62 | 0.108 | 1.89E-13 |
| sp\|P24158\|PRTN3_HUMAN | 28227.43 | 0.66 | 0.296 | 1.22E-05 |
| sp\|Q6NXT6\|TAPT1_HUMAN | 64884.52 | 0.52 | 0.08 | 2.20E-16 |
| sp\|P53680\|AP2S1_HUMAN | 17159.75 | 0.66 | 0.087 | 2.38E-14 |
| sp\|Q9H3K2\|GHITM_HUMAN | 37333.66 | 0.64 | 0.188 | 3.97E-08 |
| sp\|O60488\|ACSL4_HUMAN | 80202.14 | 0.49 | 0.142 | 1.34E-12 |
| sp\|P50416\|CPT1A_HUMAN | 88976.98 | 0.59 | 0.131 | 1.34E-11 |
| sp\|P05155\|IC1_HUMAN | 55329.47 | 0.61 | 0.173 | 1.20E-09 |
| sp\|Q9H7B4\|SMYD3_HUMAN | 50358.05 | 0.62 | 0.123 | 3.73E-11 |
| sp\|P09917\|LOX5_HUMAN | 78656.54 | 0.62 | 0.121 | 5.64E-12 |
| sp\|Q9BPX6\|MICU1_HUMAN | 54697.61 | 0.64 | 0.138 | 1.67E-10 |
| sp\|Q8N2K0\|ABD12_HUMAN | 45506.35 | 0.66 | 0.16 | 4.55E-08 |
| sp\|P20936\|RASA1_HUMAN | 117223.8 | 0.57 | 0.104 | 2.58E-14 |
| sp\|O14683\|P5I11_HUMAN | 21079.48 | 0.66 | 0.123 | 3.38E-11 |
| sp\|Q16850\|CP51A_HUMAN | 57150.61 | 0.49 | 0.126 | 1.17E-12 |
| sp\|Q9C0A1\|ZFHX2_HUMAN | 277406.7 | 0.65 | 0.189 | 5.97E-08 |
| sp\|Q9BR39\|JPH2_HUMAN | 74386.39 | 0.49 | 0.328 | 1.20E-07 |
| sp\|Q9Y512\|SAM50_HUMAN | 52324.46 | 0.58 | 0.113 | 8.41E-13 |
| sp\|P51572\|BAP31_HUMAN | 28013.02 | 0.6 | 0.135 | 7.54E-12 |
| sp\|Q8TCT7\|SPP2B_HUMAN | 65666.79 | 0.51 | 0.106 | 1.21E-14 |
| sp\|Q9ULD0\|OGDHL_HUMAN | 115245.8 | 0.66 | 0.173 | 7.47E-09 |
| sp\|Q9H6K4\|OPA3_HUMAN | 20136.56 | 0.46 | 0.15 | 7.15E-12 |
| sp\|Q92930\|RAB8B_HUMAN | 23722.13 | 0.61 | 0.099 | 6.85E-14 |
| sp\|Q53EU6\|GPAT3_HUMAN | 49168.67 | 0.61 | 0.259 | 1.66E-07 |
| sp\|Q9H6F2\|TM38A_HUMAN | 33676.25 | 0.58 | 0.297 | 5.72E-05 |
| sp\|Q13075\|BIRC1_HUMAN | 161855.6 | 0.44 | 0.156 | 2.38E-12 |
| sp\|P06737\|PYGL_HUMAN | 97468.17 | 0.66 | 0.222 | 2.66E-07 |
| sp\|Q8N201\|INT1_HUMAN | 246348.4 | 0.63 | 0.097 | 4.17E-14 |
| sp\|P48651\|PTSS1_HUMAN | 56157.35 | 0.39 | 0.079 | 2.20E-16 |
| sp\|P24557\|THAS_HUMAN | 61145.37 | 0.49 | 0.082 | 2.20E-16 |
| sp\|Q14653\|IRF3_HUMAN | 47513.73 | 0.62 | 0.19 | 3.62E-09 |
| sp\|Q2PPJ7\|RGPA2_HUMAN | 213070 | 0.5 | 0.104 | 1.32E-15 |
| sp\|Q2TAA5\|ALG11_HUMAN | 56395.16 | 0.66 | 0.154 | 3.97E-09 |
| sp\|Q7L2H7\|EIF3M_HUMAN | 42913.97 | 0.41 | 0.096 | 3.32E-16 |
| sp\|Q9Y6A1\|POMT1_HUMAN | 85662.93 | 0.62 | 0.148 | 1.41E-10 |
| sp\|Q12788\|TBL3_HUMAN | 90328.97 | 0.65 | 0.095 | 9.49E-14 |
| sp\|Q9BUN8\|DERL1_HUMAN | 28877.81 | 0.65 | 0.119 | 6.83E-12 |
| sp\|Q9Y2C3\|B3GT5_HUMAN | 36660.63 | 0.36 | 0.087 | 2.20E-16 |
| sp\|Q9Y5W7\|SNX14_HUMAN | 111062.7 | 0.49 | 0.149 | 6.49E-12 |
| sp\|Q9NRF8\|PYRG2_HUMAN | 66302.08 | 0.65 | 0.092 | 8.11E-14 |
| sp\|Q86TI2\|DPP9_HUMAN | 98981.29 | 0.64 | 0.11 | 1.98E-12 |
| sp\|Q8NFG4\|FLCN_HUMAN | 65383.35 | 0.64 | 0.193 | 9.47E-08 |
| sp\|Q5VW36\|FOCAD_HUMAN | 202092.5 | 0.56 | 0.118 | 4.09E-13 |
| sp\|P46781\|RS9_HUMAN | 22616.57 | 0.63 | 0.093 | 3.65E-14 |
| sp\|Q7Z7M9\|GALT5_HUMAN | 107036.8 | 0.65 | 0.212 | 1.44E-07 |
| sp\|Q96CW1\|AP2M1_HUMAN | 49947.14 | 0.6 | 0.08 | 4.81E-16 |
| sp\|Q7Z4G4\|TRM11_HUMAN | 53768.28 | 0.62 | 0.073 | 2.20E-16 |
| sp\|P48509\|CD151_HUMAN | 29113.62 | 0.62 | 0.073 | 2.20E-16 |
| sp\|Q96GC9\|VMP1_HUMAN | 46589 | 0.44 | 0.111 | 2.04E-14 |
