# Supplementary material for: Apolipoprotein C‐II induces EMT to promote gastric cancer peritoneal metastasis via PI3K/AKT/mTOR pathway
Source: Clin Transl Med. 2021 Aug 9;11(8):e522. doi: 10.1002/ctm2.522 (PMC8351524; doi:10.1002/ctm2.522)
Supplement: Supplementary file 19 — Table S4. GO enrichment analysis of all identified protein‐specific information and visualization results. [file CTM2-11-e522-s016.docx]

**Table S4.** **GO enrichment analysis of all identified protein-specific information and visualization results.**

| Ontology | Class | number_of_Homo_sapiens |
| --- | --- | --- |
| biological_process | behavior | 180 |
| biological_process | biological adhesion | 668 |
| biological_process | biological phase | 3 |
| biological_process | biological regulation | 4735 |
| biological_process | cell aggregation | 9 |
| biological_process | cell killing | 72 |
| biological_process | cell proliferation | 907 |
| biological_process | cellular component organization or biogenesis | 3345 |
| biological_process | cellular process | 6570 |
| biological_process | detoxification | 28 |
| biological_process | developmental process | 2542 |
| biological_process | growth | 426 |
| biological_process | immune system process | 1488 |
| biological_process | localization | 3061 |
| biological_process | locomotion | 772 |
| biological_process | metabolic process | 5029 |
| biological_process | multi-organism process | 1134 |
| biological_process | multicellular organismal process | 2824 |
| biological_process | negative regulation of biological process | 2303 |
| biological_process | nitrogen utilization | 2 |
| biological_process | pigmentation | 46 |
| biological_process | positive regulation of biological process | 2557 |
| biological_process | presynaptic process involved in chemical synaptic transmission | 51 |
| biological_process | regulation of biological process | 4491 |
| biological_process | reproduction | 466 |
| biological_process | reproductive process | 466 |
| biological_process | response to stimulus | 3704 |
| biological_process | rhythmic process | 106 |
| biological_process | signaling | 2445 |
| cellular_component | cell | 6990 |
| cellular_component | cell junction | 664 |
| cellular_component | cell part | 6984 |
| cellular_component | extracellular region | 2463 |
| cellular_component | extracellular region part | 2308 |
| cellular_component | macromolecular complex | 2794 |
| cellular_component | membrane | 3827 |
| cellular_component | membrane part | 2251 |
| cellular_component | membrane-enclosed lumen | 2925 |
| cellular_component | nucleoid | 36 |
| cellular_component | organelle | 6398 |
| cellular_component | organelle part | 4824 |
| cellular_component | other organism | 60 |
| cellular_component | other organism part | 60 |
| cellular_component | supramolecular complex | 423 |
| cellular_component | synapse | 347 |
| cellular_component | synapse part | 274 |
| cellular_component | virion | 58 |
| cellular_component | virion part | 58 |
| molecular_function | antioxidant activity | 58 |
| molecular_function | binding | 5631 |
| molecular_function | catalytic activity | 3119 |
| molecular_function | hijacked molecular function | 40 |
| molecular_function | molecular carrier activity | 20 |
| molecular_function | molecular function regulator | 769 |
| molecular_function | molecular transducer activity | 292 |
| molecular_function | protein tag | 7 |
| molecular_function | signal transducer activity | 375 |
| molecular_function | structural molecule activity | 408 |
| molecular_function | transcription regulator activity | 441 |
| molecular_function | translation regulator activity | 25 |
| molecular_function | transporter activity | 435 |
