# Supplementary material for: Apolipoprotein C‐II induces EMT to promote gastric cancer peritoneal metastasis via PI3K/AKT/mTOR pathway
Source: Clin Transl Med. 2021 Aug 9;11(8):e522. doi: 10.1002/ctm2.522 (PMC8351524; doi:10.1002/ctm2.522)
Supplement: Supplementary file 20 — Table S5. GO enrichment analysis for the DEPs. [file CTM2-11-e522-s020.docx]

**Table S5. GO enrichment analysis for the DEPs.**

| Ontoloty | GO term | Number of Proteins |
| --- | --- | --- |
| biological_process | behavior | 57 |
| biological_process | biological adhesion | 146 |
| biological_process | biological regulation | 975 |
| biological_process | cell aggregation | 3 |
| biological_process | cell killing | 14 |
| biological_process | cell proliferation | 196 |
| biological_process | cellular component organization or biogenesis | 671 |
| biological_process | cellular process | 1359 |
| biological_process | detoxification | 7 |
| biological_process | developmental process | 552 |
| biological_process | growth | 91 |
| biological_process | immune system process | 319 |
| biological_process | localization | 735 |
| biological_process | locomotion | 165 |
| biological_process | metabolic process | 1018 |
| biological_process | multi-organism process | 232 |
| biological_process | multicellular organismal process | 639 |
| biological_process | negative regulation of biological process | 454 |
| biological_process | nitrogen utilization | 1 |
| biological_process | pigmentation | 19 |
| biological_process | positive regulation of biological process | 518 |
| biological_process | presynaptic process involved in chemical synaptic transmission | 15 |
| biological_process | regulation of biological process | 914 |
| biological_process | reproduction | 108 |
| biological_process | reproductive process | 108 |
| biological_process | response to stimulus | 788 |
| biological_process | rhythmic process | 26 |
| biological_process | signaling | 508 |
| cellular_component | cell | 1470 |
| cellular_component | cell junction | 145 |
| cellular_component | cell part | 1470 |
| cellular_component | extracellular region | 613 |
| cellular_component | extracellular region part | 569 |
| cellular_component | macromolecular complex | 542 |
| cellular_component | membrane | 915 |
| cellular_component | membrane part | 653 |
| cellular_component | membrane-enclosed lumen | 517 |
| cellular_component | nucleoid | 6 |
| cellular_component | organelle | 1353 |
| cellular_component | organelle part | 1041 |
| cellular_component | other organism | 9 |
| cellular_component | other organism part | 9 |
| cellular_component | supramolecular complex | 98 |
| cellular_component | synapse | 90 |
| cellular_component | synapse part | 72 |
| cellular_component | virion | 10 |
| cellular_component | virion part | 10 |
| molecular_function | antioxidant activity | 10 |
| molecular_function | binding | 1103 |
| molecular_function | catalytic activity | 650 |
| molecular_function | hijacked molecular function | 15 |
| molecular_function | molecular carrier activity | 4 |
| molecular_function | molecular function regulator | 142 |
| molecular_function | molecular transducer activity | 65 |
| molecular_function | protein tag | 5 |
| molecular_function | signal transducer activity | 77 |
| molecular_function | structural molecule activity | 93 |
| molecular_function | transcription regulator activity | 53 |
| molecular_function | translation regulator activity | 6 |
| molecular_function | transporter activity | 159 |
