# Supplementary material for: Apolipoprotein C‐II induces EMT to promote gastric cancer peritoneal metastasis via PI3K/AKT/mTOR pathway
Source: Clin Transl Med. 2021 Aug 9;11(8):e522. doi: 10.1002/ctm2.522 (PMC8351524; doi:10.1002/ctm2.522)
Supplement: Supplementary file 21 — Table S6. KOG analysis of all identified proteins. [file CTM2-11-e522-s025.docx]

**Table S6****. KOG analysis of all identified proteins.**

| Code | Functional-Categories | Family | Protein-Number |
| --- | --- | --- | --- |
| A | RNA processing and modification | INFORMATION STORAGE AND PROCESSING | 413 |
| B | Chromatin structure and dynamics | INFORMATION STORAGE AND PROCESSING | 188 |
| C | Energy production and conversion | METABOLISM | 241 |
| D | Cell cycle control, cell division, chromosome partitioning | CELLULAR PROCESSES AND SIGNALING | 288 |
| E | Amino acid transport and metabolism | METABOLISM | 224 |
| F | Nucleotide transport and metabolism | METABOLISM | 129 |
| G | Carbohydrate transport and metabolism | METABOLISM | 220 |
| H | Coenzyme transport and metabolism | METABOLISM | 69 |
| I | Lipid transport and metabolism | METABOLISM | 288 |
| J | Translation, ribosomal structure and biogenesis | INFORMATION STORAGE AND PROCESSING | 379 |
| K | Transcription | INFORMATION STORAGE AND PROCESSING | 450 |
| L | Replication, recombination and repair | INFORMATION STORAGE AND PROCESSING | 146 |
| M | Cell wall/membrane/envelope biogenesis | CELLULAR PROCESSES AND SIGNALING | 95 |
| N | Cell motility | CELLULAR PROCESSES AND SIGNALING | 18 |
| O | Posttranslational modification, protein turnover, chaperones | CELLULAR PROCESSES AND SIGNALING | 852 |
| P | Inorganic ion transport and metabolism | METABOLISM | 193 |
| Q | Secondary metabolites biosynthesis, transport and catabolism | METABOLISM | 75 |
| R | General function prediction only | POORLY CHARACTERIZED | 1407 |
| S | Function unknown | POORLY CHARACTERIZED | 625 |
| T | Signal transduction mechanisms | CELLULAR PROCESSES AND SIGNALING | 1379 |
| U | Intracellular trafficking, secretion, and vesicular transport | CELLULAR PROCESSES AND SIGNALING | 621 |
| V | Defense mechanisms | CELLULAR PROCESSES AND SIGNALING | 163 |
| W | Extracellular structures | CELLULAR PROCESSES AND SIGNALING | 204 |
| Y | Nuclear structure | CELLULAR PROCESSES AND SIGNALING | 72 |
| Z | Cytoskeleton | CELLULAR PROCESSES AND SIGNALING | 420 |
