# Supplementary material for: Apolipoprotein C‐II induces EMT to promote gastric cancer peritoneal metastasis via PI3K/AKT/mTOR pathway
Source: Clin Transl Med. 2021 Aug 9;11(8):e522. doi: 10.1002/ctm2.522 (PMC8351524; doi:10.1002/ctm2.522)
Supplement: Supplementary file 23 — Table S8. KEGG enrichment analysis of all identified proteins. [file CTM2-11-e522-s027.docx]

**Table S8. KEGG enrichment analysis of all identified proteins.**

| Pathway_level1 | Pathway_level2 | Number_of_Proteins |
| --- | --- | --- |
| Cellular Processes | Cell growth and death | 413 |
| Cellular Processes | Cell motility | 163 |
| Cellular Processes | Cellular community - eukaryotes | 373 |
| Cellular Processes | Transport and catabolism | 627 |
| Environmental Information Processing | Membrane transport | 20 |
| Environmental Information Processing | Signal transduction | 996 |
| Environmental Information Processing | Signaling molecules and interaction | 235 |
| Genetic Information Processing | Folding, sorting and degradation | 405 |
| Genetic Information Processing | Replication and repair | 107 |
| Genetic Information Processing | Transcription | 191 |
| Genetic Information Processing | Translation | 429 |
| Human Diseases | Cancers: Overview | 679 |
| Human Diseases | Cancers: Specific types | 300 |
| Human Diseases | Cardiovascular diseases | 252 |
| Human Diseases | Drug resistance: Antineoplastic | 146 |
| Human Diseases | Endocrine and metabolic diseases | 316 |
| Human Diseases | Immune diseases | 173 |
| Human Diseases | Infectious diseases: Bacterial | 385 |
| Human Diseases | Infectious diseases: Parasitic | 248 |
| Human Diseases | Infectious diseases: Viral | 701 |
| Human Diseases | Neurodegenerative diseases | 274 |
| Human Diseases | Substance dependence | 106 |
| Metabolism | Amino acid metabolism | 222 |
| Metabolism | Biosynthesis of other secondary metabolites | 8 |
| Metabolism | Carbohydrate metabolism | 300 |
| Metabolism | Energy metabolism | 127 |
| Metabolism | Global and overview maps | 921 |
| Metabolism | Glycan biosynthesis and metabolism | 151 |
| Metabolism | Lipid metabolism | 230 |
| Metabolism | Metabolism of cofactors and vitamins | 152 |
| Metabolism | Metabolism of other amino acids | 92 |
| Metabolism | Metabolism of terpenoids and polyketides | 21 |
| Metabolism | Nucleotide metabolism | 112 |
| Metabolism | Xenobiotics biodegradation and metabolism | 76 |
| Organismal Systems | Aging | 112 |
| Organismal Systems | Circulatory system | 136 |
| Organismal Systems | Development | 190 |
| Organismal Systems | Digestive system | 228 |
| Organismal Systems | Endocrine system | 496 |
| Organismal Systems | Environmental adaptation | 218 |
| Organismal Systems | Excretory system | 95 |
| Organismal Systems | Immune system | 736 |
| Organismal Systems | Nervous system | 283 |
| Organismal Systems | Sensory system | 92 |
