# Supplementary material for: Apolipoprotein C‐II induces EMT to promote gastric cancer peritoneal metastasis via PI3K/AKT/mTOR pathway
Source: Clin Transl Med. 2021 Aug 9;11(8):e522. doi: 10.1002/ctm2.522 (PMC8351524; doi:10.1002/ctm2.522)
Supplement: Supplementary file 24 — Table S9. KEGG enrichment analysis of the DEPs. [file CTM2-11-e522-s014.docx]

**Table S9. KEGG enrichment analysis the DEPs.**

| Pathway_level1 | Pathway_level2 | Number_of_Proteins |
| --- | --- | --- |
| Cellular Processes | Cell growth and death | 90 |
| Cellular Processes | Cell motility | 26 |
| Cellular Processes | Cellular community - eukaryotes | 62 |
| Cellular Processes | Transport and catabolism | 131 |
| Environmental Information Processing | Membrane transport | 9 |
| Environmental Information Processing | Signal transduction | 200 |
| Environmental Information Processing | Signaling molecules and interaction | 54 |
| Genetic Information Processing | Folding, sorting and degradation | 80 |
| Genetic Information Processing | Replication and repair | 18 |
| Genetic Information Processing | Transcription | 32 |
| Genetic Information Processing | Translation | 86 |
| Human Diseases | Cancers: Overview | 139 |
| Human Diseases | Cancers: Specific types | 52 |
| Human Diseases | Cardiovascular diseases | 56 |
| Human Diseases | Drug resistance: Antineoplastic | 34 |
| Human Diseases | Endocrine and metabolic diseases | 64 |
| Human Diseases | Immune diseases | 37 |
| Human Diseases | Infectious diseases: Bacterial | 65 |
| Human Diseases | Infectious diseases: Parasitic | 46 |
| Human Diseases | Infectious diseases: Viral | 134 |
| Human Diseases | Neurodegenerative diseases | 68 |
| Human Diseases | Substance dependence | 19 |
| Metabolism | Amino acid metabolism | 41 |
| Metabolism | Biosynthesis of other secondary metabolites | 3 |
| Metabolism | Carbohydrate metabolism | 67 |
| Metabolism | Energy metabolism | 30 |
| Metabolism | Global and overview maps | 221 |
| Metabolism | Glycan biosynthesis and metabolism | 47 |
| Metabolism | Lipid metabolism | 82 |
| Metabolism | Metabolism of cofactors and vitamins | 26 |
| Metabolism | Metabolism of other amino acids | 23 |
| Metabolism | Metabolism of terpenoids and polyketides | 7 |
| Metabolism | Nucleotide metabolism | 19 |
| Metabolism | Xenobiotics biodegradation and metabolism | 24 |
| Organismal Systems | Aging | 11 |
| Organismal Systems | Circulatory system | 35 |
| Organismal Systems | Development | 34 |
| Organismal Systems | Digestive system | 78 |
| Organismal Systems | Endocrine system | 117 |
| Organismal Systems | Environmental adaptation | 43 |
| Organismal Systems | Excretory system | 27 |
| Organismal Systems | Immune system | 136 |
| Organismal Systems | Nervous system | 58 |
| Organismal Systems | Sensory system | 19 |
