# Supplementary material for: Apolipoprotein C‐II induces EMT to promote gastric cancer peritoneal metastasis via PI3K/AKT/mTOR pathway
Source: Clin Transl Med. 2021 Aug 9;11(8):e522. doi: 10.1002/ctm2.522 (PMC8351524; doi:10.1002/ctm2.522)
Supplement: Supplementary file 25 — Table S10. Subcellular localizations of the DEPs via bioinformatic tools (WoLF PSORT). [file CTM2-11-e522-s001.docx]

**Table S10.** **Subcellular localizations of the DEPs via bioinformatic tools (WoLF PSORT).**

| SeqID | Subcellular_location | Description |
| --- | --- | --- |
| sp\|P16104\|H2AX_HUMAN | nucl | Histone H2AX OS=Homo sapiens OX=9606 GN=H2AFX PE=1 SV=2 |
| sp\|Q8N5I3\|KCNRG_HUMAN | mito | Potassium channel regulatory protein OS=Homo sapiens OX=9606 GN=KCNRG PE=1 SV=1 |
| sp\|Q01105\|SET_HUMAN | nucl | Protein SET OS=Homo sapiens OX=9606 GN=SET PE=1 SV=3 |
| sp\|Q8TBN0\|R3GEF_HUMAN | cyto | Guanine nucleotide exchange factor for Rab-3A OS=Homo sapiens OX=9606 GN=RAB3IL1 PE=1 SV=1 |
| sp\|Q92569\|P55G_HUMAN | cyto | Phosphatidylinositol 3-kinase regulatory subunit gamma OS=Homo sapiens OX=9606 GN=PIK3R3 PE=1 SV=2 |
| sp\|P18859\|ATP5J_HUMAN | mito | ATP synthase-coupling factor 6, mitochondrial OS=Homo sapiens OX=9606 GN=ATP5PF PE=1 SV=1 |
| sp\|Q9HCM4\|E41L5_HUMAN | nucl | Band 4.1-like protein 5 OS=Homo sapiens OX=9606 GN=EPB41L5 PE=1 SV=3 |
| sp\|P08218\|CEL2B_HUMAN | extr | Chymotrypsin-like elastase family member 2B OS=Homo sapiens OX=9606 GN=CELA2B PE=2 SV=2 |
| sp\|P21860\|ERBB3_HUMAN | extr | Receptor tyrosine-protein kinase erbB-3 OS=Homo sapiens OX=9606 GN=ERBB3 PE=1 SV=1 |
| sp\|P00519\|ABL1_HUMAN | nucl | Tyrosine-protein kinase ABL1 OS=Homo sapiens OX=9606 GN=ABL1 PE=1 SV=4 |
| sp\|Q9H9C1\|SPE39_HUMAN | nucl | Spermatogenesis-defective protein 39 homolog OS=Homo sapiens OX=9606 GN=VIPAS39 PE=1 SV=1 |
| sp\|O14874\|BCKD_HUMAN | mito | [3-methyl-2-oxobutanoate dehydrogenase [lipoamide]] kinase, mitochondrial OS=Homo sapiens OX=9606 GN=BCKDK PE=1 SV=2 |
| sp\|P46977\|STT3A_HUMAN | plas | Dolichyl-diphosphooligosaccharide--protein glycosyltransferase subunit STT3A OS=Homo sapiens OX=9606 GN=STT3A PE=1 SV=2 |
| sp\|Q6NZI2\|CAVN1_HUMAN | nucl | Caveolae-associated protein 1 OS=Homo sapiens OX=9606 GN=CAVIN1 PE=1 SV=1 |
| sp\|P54803\|GALC_HUMAN | cyto | Galactocerebrosidase OS=Homo sapiens OX=9606 GN=GALC PE=1 SV=3 |
| sp\|P19021\|AMD_HUMAN | plas | Peptidyl-glycine alpha-amidating monooxygenase OS=Homo sapiens OX=9606 GN=PAM PE=1 SV=2 |
| sp\|Q7L523\|RRAGA_HUMAN | mito | Ras-related GTP-binding protein A OS=Homo sapiens OX=9606 GN=RRAGA PE=1 SV=1 |
| sp\|Q6DD88\|ATLA3_HUMAN | plas | Atlastin-3 OS=Homo sapiens OX=9606 GN=ATL3 PE=1 SV=1 |
| sp\|O43847\|NRDC_HUMAN | mito | Nardilysin OS=Homo sapiens OX=9606 GN=NRDC PE=1 SV=3 |
| sp\|P49326\|FMO5_HUMAN | plas | Dimethylaniline monooxygenase [N-oxide-forming] 5 OS=Homo sapiens OX=9606 GN=FMO5 PE=1 SV=2 |
| sp\|Q5T9L3\|WLS_HUMAN | plas | Protein wntless homolog OS=Homo sapiens OX=9606 GN=WLS PE=1 SV=2 |
| sp\|Q7Z4Q2\|HEAT3_HUMAN | nucl | HEAT repeat-containing protein 3 OS=Homo sapiens OX=9606 GN=HEATR3 PE=1 SV=2 |
| sp\|P41235\|HNF4A_HUMAN | nucl | Hepatocyte nuclear factor 4-alpha OS=Homo sapiens OX=9606 GN=HNF4A PE=1 SV=3 |
| sp\|Q16774\|KGUA_HUMAN | mito | Guanylate kinase OS=Homo sapiens OX=9606 GN=GUK1 PE=1 SV=2 |
| sp\|Q9H4H8\|FA83D_HUMAN | nucl | Protein FAM83D OS=Homo sapiens OX=9606 GN=FAM83D PE=1 SV=3 |
| sp\|Q9C0D6\|FHDC1_HUMAN | nucl | FH2 domain-containing protein 1 OS=Homo sapiens OX=9606 GN=FHDC1 PE=1 SV=2 |
| sp\|Q96GG9\|DCNL1_HUMAN | cyto | DCN1-like protein 1 OS=Homo sapiens OX=9606 GN=DCUN1D1 PE=1 SV=1 |
| sp\|P17213\|BPI_HUMAN | extr | Bactericidal permeability-increasing protein OS=Homo sapiens OX=9606 GN=BPI PE=1 SV=4 |
| sp\|Q7Z460\|CLAP1_HUMAN | nucl | CLIP-associating protein 1 OS=Homo sapiens OX=9606 GN=CLASP1 PE=1 SV=1 |
| sp\|P57088\|TMM33_HUMAN | plas | Transmembrane protein 33 OS=Homo sapiens OX=9606 GN=TMEM33 PE=1 SV=2 |
| sp\|Q9NRX1\|PNO1_HUMAN | cyto | RNA-binding protein PNO1 OS=Homo sapiens OX=9606 GN=PNO1 PE=1 SV=1 |
| sp\|P16402\|H13_HUMAN | nucl | Histone H1.3 OS=Homo sapiens OX=9606 GN=HIST1H1D PE=1 SV=2 |
| sp\|P46734\|MP2K3_HUMAN | nucl | Dual specificity mitogen-activated protein kinase kinase 3 OS=Homo sapiens OX=9606 GN=MAP2K3 PE=1 SV=2 |
| sp\|Q9UJ70\|NAGK_HUMAN | cysk | N-acetyl-D-glucosamine kinase OS=Homo sapiens OX=9606 GN=NAGK PE=1 SV=4 |
| sp\|Q11206\|SIA4C_HUMAN | plas | CMP-N-acetylneuraminate-beta-galactosamide-alpha-2,3-sialyltransferase 4 OS=Homo sapiens OX=9606 GN=ST3GAL4 PE=2 SV=1 |
| sp\|P22894\|MMP8_HUMAN | extr | Neutrophil collagenase OS=Homo sapiens OX=9606 GN=MMP8 PE=1 SV=1 |
| sp\|Q9P2D7\|DYH1_HUMAN | cyto | Dynein heavy chain 1, axonemal OS=Homo sapiens OX=9606 GN=DNAH1 PE=1 SV=5 |
| sp\|P54646\|AAPK2_HUMAN | cyto | 5'-AMP-activated protein kinase catalytic subunit alpha-2 OS=Homo sapiens OX=9606 GN=PRKAA2 PE=1 SV=2 |
| sp\|Q5T3U5\|MRP7_HUMAN | plas | Multidrug resistance-associated protein 7 OS=Homo sapiens OX=9606 GN=ABCC10 PE=1 SV=1 |
| sp\|P14317\|HCLS1_HUMAN | cyto | Hematopoietic lineage cell-specific protein OS=Homo sapiens OX=9606 GN=HCLS1 PE=1 SV=3 |
| sp\|Q9Y6Q1\|CAN6_HUMAN | cyto | Calpain-6 OS=Homo sapiens OX=9606 GN=CAPN6 PE=1 SV=2 |
| sp\|P29536\|LMOD1_HUMAN | nucl | Leiomodin-1 OS=Homo sapiens OX=9606 GN=LMOD1 PE=1 SV=3 |
| sp\|I3L1E1\|CS084_HUMAN | cyto | Uncharacterized protein C19orf84 OS=Homo sapiens OX=9606 GN=C19orf84 PE=4 SV=1 |
| sp\|P30679\|GNA15_HUMAN | mito | Guanine nucleotide-binding protein subunit alpha-15 OS=Homo sapiens OX=9606 GN=GNA15 PE=1 SV=2 |
| sp\|O95352\|ATG7_HUMAN | cyto | Ubiquitin-like modifier-activating enzyme ATG7 OS=Homo sapiens OX=9606 GN=ATG7 PE=1 SV=1 |
| sp\|Q9BUR5\|MIC26_HUMAN | mito | MICOS complex subunit MIC26 OS=Homo sapiens OX=9606 GN=APOO PE=1 SV=1 |
| sp\|Q8WVQ1\|CANT1_HUMAN | extr | Soluble calcium-activated nucleotidase 1 OS=Homo sapiens OX=9606 GN=CANT1 PE=1 SV=1 |
| sp\|P10301\|RRAS_HUMAN | nucl | Ras-related protein R-Ras OS=Homo sapiens OX=9606 GN=RRAS PE=1 SV=1 |
| sp\|Q9BTW9\|TBCD_HUMAN | cyto | Tubulin-specific chaperone D OS=Homo sapiens OX=9606 GN=TBCD PE=1 SV=2 |
| sp\|Q8TEH3\|DEN1A_HUMAN | cyto | DENN domain-containing protein 1A OS=Homo sapiens OX=9606 GN=DENND1A PE=1 SV=2 |
| sp\|O75110\|ATP9A_HUMAN | plas | Probable phospholipid-transporting ATPase IIA OS=Homo sapiens OX=9606 GN=ATP9A PE=1 SV=3 |
| sp\|P55786\|PSA_HUMAN | mito | Puromycin-sensitive aminopeptidase OS=Homo sapiens OX=9606 GN=NPEPPS PE=1 SV=2 |
| sp\|P54136\|SYRC_HUMAN | cyto | Arginine--tRNA ligase, cytoplasmic OS=Homo sapiens OX=9606 GN=RARS PE=1 SV=2 |
| sp\|Q8N335\|GPD1L_HUMAN | cyto | Glycerol-3-phosphate dehydrogenase 1-like protein OS=Homo sapiens OX=9606 GN=GPD1L PE=1 SV=1 |
| sp\|Q8NFL0\|B3GN7_HUMAN | extr | UDP-GlcNAc:betaGal beta-1,3-N-acetylglucosaminyltransferase 7 OS=Homo sapiens OX=9606 GN=B3GNT7 PE=2 SV=1 |
| sp\|Q10472\|GALT1_HUMAN | golg | Polypeptide N-acetylgalactosaminyltransferase 1 OS=Homo sapiens OX=9606 GN=GALNT1 PE=1 SV=1 |
| sp\|P0DOX8\|IGL1_HUMAN | nucl | Immunoglobulin lambda-1 light chain OS=Homo sapiens OX=9606 PE=1 SV=1 |
| sp\|Q9ULD9\|ZN608_HUMAN | nucl | Zinc finger protein 608 OS=Homo sapiens OX=9606 GN=ZNF608 PE=1 SV=4 |
| sp\|Q8WXE0\|CSKI2_HUMAN | nucl | Caskin-2 OS=Homo sapiens OX=9606 GN=CASKIN2 PE=1 SV=2 |
| sp\|P22748\|CAH4_HUMAN | extr | Carbonic anhydrase 4 OS=Homo sapiens OX=9606 GN=CA4 PE=1 SV=2 |
| sp\|Q9Y5J9\|TIM8B_HUMAN | extr | Mitochondrial import inner membrane translocase subunit Tim8 B OS=Homo sapiens OX=9606 GN=TIMM8B PE=1 SV=1 |
| sp\|Q6ZMR5\|TM11A_HUMAN | golg | Transmembrane protease serine 11A OS=Homo sapiens OX=9606 GN=TMPRSS11A PE=1 SV=1 |
| sp\|Q3MIR4\|CC50B_HUMAN | plas | Cell cycle control protein 50B OS=Homo sapiens OX=9606 GN=TMEM30B PE=1 SV=1 |
| sp\|P23526\|SAHH_HUMAN | cyto | Adenosylhomocysteinase OS=Homo sapiens OX=9606 GN=AHCY PE=1 SV=4 |
| sp\|P14923\|PLAK_HUMAN | cyto | Junction plakoglobin OS=Homo sapiens OX=9606 GN=JUP PE=1 SV=3 |
| sp\|Q92828\|COR2A_HUMAN | nucl | Coronin-2A OS=Homo sapiens OX=9606 GN=CORO2A PE=1 SV=2 |
| sp\|Q2M389\|WASC4_HUMAN | cyto | WASH complex subunit 4 OS=Homo sapiens OX=9606 GN=WASHC4 PE=1 SV=2 |
| sp\|P31323\|KAP3_HUMAN | cyto | cAMP-dependent protein kinase type II-beta regulatory subunit OS=Homo sapiens OX=9606 GN=PRKAR2B PE=1 SV=3 |
| sp\|O60331\|PI51C_HUMAN | nucl | Phosphatidylinositol 4-phosphate 5-kinase type-1 gamma OS=Homo sapiens OX=9606 GN=PIP5K1C PE=1 SV=2 |
| sp\|O76038\|SEGN_HUMAN | cyto | Secretagogin OS=Homo sapiens OX=9606 GN=SCGN PE=2 SV=2 |
| sp\|Q9H0U4\|RAB1B_HUMAN | cyto | Ras-related protein Rab-1B OS=Homo sapiens OX=9606 GN=RAB1B PE=1 SV=1 |
| sp\|Q9BSR8\|YIPF4_HUMAN | plas | Protein YIPF4 OS=Homo sapiens OX=9606 GN=YIPF4 PE=1 SV=1 |
| sp\|P14649\|MYL6B_HUMAN | cyto | Myosin light chain 6B OS=Homo sapiens OX=9606 GN=MYL6B PE=1 SV=1 |
| sp\|A0A0B4J1V0\|HV315_HUMAN | extr | Immunoglobulin heavy variable 3-15 OS=Homo sapiens OX=9606 GN=IGHV3-15 PE=3 SV=1 |
| sp\|Q3SXM5\|HSDL1_HUMAN | plas | Inactive hydroxysteroid dehydrogenase-like protein 1 OS=Homo sapiens OX=9606 GN=HSDL1 PE=1 SV=3 |
| sp\|O43490\|PROM1_HUMAN | plas | Prominin-1 OS=Homo sapiens OX=9606 GN=PROM1 PE=1 SV=1 |
| sp\|P57735\|RAB25_HUMAN | cysk | Ras-related protein Rab-25 OS=Homo sapiens OX=9606 GN=RAB25 PE=1 SV=2 |
| sp\|O60307\|MAST3_HUMAN | nucl | Microtubule-associated serine/threonine-protein kinase 3 OS=Homo sapiens OX=9606 GN=MAST3 PE=1 SV=2 |
| sp\|Q16706\|MA2A1_HUMAN | plas | Alpha-mannosidase 2 OS=Homo sapiens OX=9606 GN=MAN2A1 PE=1 SV=2 |
| sp\|Q9NYA4\|MTMR4_HUMAN | nucl | Myotubularin-related protein 4 OS=Homo sapiens OX=9606 GN=MTMR4 PE=1 SV=2 |
| sp\|Q53RD9\|FBLN7_HUMAN | extr | Fibulin-7 OS=Homo sapiens OX=9606 GN=FBLN7 PE=2 SV=1 |
| sp\|O60518\|RNBP6_HUMAN | cyto | Ran-binding protein 6 OS=Homo sapiens OX=9606 GN=RANBP6 PE=1 SV=2 |
| sp\|P42126\|ECI1_HUMAN | mito | Enoyl-CoA delta isomerase 1, mitochondrial OS=Homo sapiens OX=9606 GN=ECI1 PE=1 SV=1 |
| sp\|A1A5B4\|ANO9_HUMAN | plas | Anoctamin-9 OS=Homo sapiens OX=9606 GN=ANO9 PE=1 SV=3 |
| sp\|Q9UBC5\|MYO1A_HUMAN | cyto | Unconventional myosin-Ia OS=Homo sapiens OX=9606 GN=MYO1A PE=1 SV=1 |
| sp\|Q8TDH9\|BL1S5_HUMAN | cyto | Biogenesis of lysosome-related organelles complex 1 subunit 5 OS=Homo sapiens OX=9606 GN=BLOC1S5 PE=1 SV=1 |
| sp\|P04839\|CY24B_HUMAN | plas | Cytochrome b-245 heavy chain OS=Homo sapiens OX=9606 GN=CYBB PE=1 SV=2 |
| sp\|Q6NUK1\|SCMC1_HUMAN | cyto | Calcium-binding mitochondrial carrier protein SCaMC-1 OS=Homo sapiens OX=9606 GN=SLC25A24 PE=1 SV=2 |
| sp\|Q96HH4\|TM169_HUMAN | plas | Transmembrane protein 169 OS=Homo sapiens OX=9606 GN=TMEM169 PE=2 SV=1 |
| sp\|P23497\|SP100_HUMAN | nucl | Nuclear autoantigen Sp-100 OS=Homo sapiens OX=9606 GN=SP100 PE=1 SV=3 |
| sp\|Q96QI5\|HS3S6_HUMAN | plas | Heparan sulfate glucosamine 3-O-sulfotransferase 6 OS=Homo sapiens OX=9606 GN=HS3ST6 PE=1 SV=2 |
| sp\|Q05D32\|CTSL2_HUMAN | nucl | CTD small phosphatase-like protein 2 OS=Homo sapiens OX=9606 GN=CTDSPL2 PE=1 SV=2 |
| sp\|Q9UI08\|EVL_HUMAN | nucl | Ena/VASP-like protein OS=Homo sapiens OX=9606 GN=EVL PE=1 SV=2 |
| sp\|Q9UGC7\|RF1ML_HUMAN | mito | Peptide chain release factor 1-like, mitochondrial OS=Homo sapiens OX=9606 GN=MTRF1L PE=1 SV=1 |
| sp\|Q02978\|M2OM_HUMAN | cyto | Mitochondrial 2-oxoglutarate/malate carrier protein OS=Homo sapiens OX=9606 GN=SLC25A11 PE=1 SV=3 |
| sp\|P49747\|COMP_HUMAN | extr | Cartilage oligomeric matrix protein OS=Homo sapiens OX=9606 GN=COMP PE=1 SV=2 |
| sp\|P00325\|ADH1B_HUMAN | cyto | Alcohol dehydrogenase 1B OS=Homo sapiens OX=9606 GN=ADH1B PE=1 SV=2 |
| sp\|P60983\|GMFB_HUMAN | cyto | Glia maturation factor beta OS=Homo sapiens OX=9606 GN=GMFB PE=1 SV=2 |
| sp\|P50148\|GNAQ_HUMAN | cysk | Guanine nucleotide-binding protein G(q) subunit alpha OS=Homo sapiens OX=9606 GN=GNAQ PE=1 SV=4 |
| sp\|Q6WCQ1\|MPRIP_HUMAN | nucl | Myosin phosphatase Rho-interacting protein OS=Homo sapiens OX=9606 GN=MPRIP PE=1 SV=3 |
| sp\|O76024\|WFS1_HUMAN | plas | Wolframin OS=Homo sapiens OX=9606 GN=WFS1 PE=1 SV=2 |
| sp\|Q9H0V1\|TM168_HUMAN | plas | Transmembrane protein 168 OS=Homo sapiens OX=9606 GN=TMEM168 PE=2 SV=2 |
| sp\|O60832\|DKC1_HUMAN | cyto | H/ACA ribonucleoprotein complex subunit DKC1 OS=Homo sapiens OX=9606 GN=DKC1 PE=1 SV=3 |
| sp\|Q9HC62\|SENP2_HUMAN | mito | Sentrin-specific protease 2 OS=Homo sapiens OX=9606 GN=SENP2 PE=1 SV=3 |
| sp\|Q9H5V8\|CDCP1_HUMAN | extr | CUB domain-containing protein 1 OS=Homo sapiens OX=9606 GN=CDCP1 PE=1 SV=3 |
| sp\|Q6ZNC4\|ZN704_HUMAN | nucl | Zinc finger protein 704 OS=Homo sapiens OX=9606 GN=ZNF704 PE=1 SV=1 |
| sp\|P27658\|CO8A1_HUMAN | E.R. | Collagen alpha-1(VIII) chain OS=Homo sapiens OX=9606 GN=COL8A1 PE=1 SV=2 |
| sp\|Q9BT73\|PSMG3_HUMAN | mito | Proteasome assembly chaperone 3 OS=Homo sapiens OX=9606 GN=PSMG3 PE=1 SV=1 |
| sp\|O15397\|IPO8_HUMAN | cyto | Importin-8 OS=Homo sapiens OX=9606 GN=IPO8 PE=1 SV=2 |
| sp\|P54259\|ATN1_HUMAN | nucl | Atrophin-1 OS=Homo sapiens OX=9606 GN=ATN1 PE=1 SV=3 |
| sp\|Q9BZJ0\|CRNL1_HUMAN | nucl | Crooked neck-like protein 1 OS=Homo sapiens OX=9606 GN=CRNKL1 PE=1 SV=4 |
| sp\|O14531\|DPYL4_HUMAN | cyto | Dihydropyrimidinase-related protein 4 OS=Homo sapiens OX=9606 GN=DPYSL4 PE=1 SV=2 |
| sp\|P57105\|SYJ2B_HUMAN | cyto | Synaptojanin-2-binding protein OS=Homo sapiens OX=9606 GN=SYNJ2BP PE=1 SV=2 |
| sp\|Q8TEM1\|PO210_HUMAN | plas | Nuclear pore membrane glycoprotein 210 OS=Homo sapiens OX=9606 GN=NUP210 PE=1 SV=3 |
| sp\|O43665\|RGS10_HUMAN | nucl | Regulator of G-protein signaling 10 OS=Homo sapiens OX=9606 GN=RGS10 PE=1 SV=2 |
| sp\|Q8N4Q1\|MIA40_HUMAN | extr | Mitochondrial intermembrane space import and assembly protein 40 OS=Homo sapiens OX=9606 GN=CHCHD4 PE=1 SV=1 |
| sp\|Q13526\|PIN1_HUMAN | cyto | Peptidyl-prolyl cis-trans isomerase NIMA-interacting 1 OS=Homo sapiens OX=9606 GN=PIN1 PE=1 SV=1 |
| sp\|P09132\|SRP19_HUMAN | cyto | Signal recognition particle 19 kDa protein OS=Homo sapiens OX=9606 GN=SRP19 PE=1 SV=3 |
| sp\|Q8IZA0\|K319L_HUMAN | nucl | Dyslexia-associated protein KIAA0319-like protein OS=Homo sapiens OX=9606 GN=KIAA0319L PE=1 SV=2 |
| sp\|Q6GPI1\|CTRB2_HUMAN | extr | Chymotrypsinogen B2 OS=Homo sapiens OX=9606 GN=CTRB2 PE=2 SV=2 |
| sp\|Q95IE3\|2B1C_HUMAN | extr | HLA class II histocompatibility antigen, DRB1-12 beta chain OS=Homo sapiens OX=9606 GN=HLA-DRB1 PE=1 SV=1 |
| sp\|P17927\|CR1_HUMAN | extr | Complement receptor type 1 OS=Homo sapiens OX=9606 GN=CR1 PE=1 SV=3 |
| sp\|Q96K19\|RN170_HUMAN | plas | E3 ubiquitin-protein ligase RNF170 OS=Homo sapiens OX=9606 GN=RNF170 PE=1 SV=2 |
| sp\|Q8WW12\|PCNP_HUMAN | cyto | PEST proteolytic signal-containing nuclear protein OS=Homo sapiens OX=9606 GN=PCNP PE=1 SV=2 |
| sp\|P23634\|AT2B4_HUMAN | plas | Plasma membrane calcium-transporting ATPase 4 OS=Homo sapiens OX=9606 GN=ATP2B4 PE=1 SV=2 |
| sp\|O60575\|ISK4_HUMAN | extr | Serine protease inhibitor Kazal-type 4 OS=Homo sapiens OX=9606 GN=SPINK4 PE=3 SV=1 |
| sp\|O43772\|MCAT_HUMAN | plas | Mitochondrial carnitine/acylcarnitine carrier protein OS=Homo sapiens OX=9606 GN=SLC25A20 PE=1 SV=1 |
| sp\|P55061\|BI1_HUMAN | plas | Bax inhibitor 1 OS=Homo sapiens OX=9606 GN=TMBIM6 PE=1 SV=2 |
| sp\|Q9H6E4\|CC134_HUMAN | E.R. | Coiled-coil domain-containing protein 134 OS=Homo sapiens OX=9606 GN=CCDC134 PE=1 SV=1 |
| sp\|Q96AM1\|MRGRF_HUMAN | plas | Mas-related G-protein coupled receptor member F OS=Homo sapiens OX=9606 GN=MRGPRF PE=2 SV=1 |
| sp\|P04271\|S100B_HUMAN | cyto | Protein S100-B OS=Homo sapiens OX=9606 GN=S100B PE=1 SV=2 |
| sp\|P13987\|CD59_HUMAN | extr | CD59 glycoprotein OS=Homo sapiens OX=9606 GN=CD59 PE=1 SV=1 |
| sp\|O14734\|ACOT8_HUMAN | cyto | Acyl-coenzyme A thioesterase 8 OS=Homo sapiens OX=9606 GN=ACOT8 PE=1 SV=1 |
| sp\|Q7Z2K6\|ERMP1_HUMAN | plas | Endoplasmic reticulum metallopeptidase 1 OS=Homo sapiens OX=9606 GN=ERMP1 PE=1 SV=2 |
| sp\|Q10469\|MGAT2_HUMAN | golg | Alpha-1,6-mannosyl-glycoprotein 2-beta-N-acetylglucosaminyltransferase OS=Homo sapiens OX=9606 GN=MGAT2 PE=1 SV=1 |
| sp\|Q7L5L3\|GDPD3_HUMAN | extr | Lysophospholipase D GDPD3 OS=Homo sapiens OX=9606 GN=GDPD3 PE=2 SV=3 |
| sp\|Q96P70\|IPO9_HUMAN | cyto | Importin-9 OS=Homo sapiens OX=9606 GN=IPO9 PE=1 SV=3 |
| sp\|O75964\|ATP5L_HUMAN | cyto | ATP synthase subunit g, mitochondrial OS=Homo sapiens OX=9606 GN=ATP5MG PE=1 SV=3 |
| sp\|Q96EP5\|DAZP1_HUMAN | nucl | DAZ-associated protein 1 OS=Homo sapiens OX=9606 GN=DAZAP1 PE=1 SV=1 |
| sp\|Q15041\|AR6P1_HUMAN | plas | ADP-ribosylation factor-like protein 6-interacting protein 1 OS=Homo sapiens OX=9606 GN=ARL6IP1 PE=1 SV=2 |
| sp\|Q8NEF9\|SRFB1_HUMAN | nucl | Serum response factor-binding protein 1 OS=Homo sapiens OX=9606 GN=SRFBP1 PE=1 SV=1 |
| sp\|P53674\|CRBB1_HUMAN | cyto_nucl | Beta-crystallin B1 OS=Homo sapiens OX=9606 GN=CRYBB1 PE=1 SV=2 |
| sp\|P56470\|LEG4_HUMAN | cyto_nucl | Galectin-4 OS=Homo sapiens OX=9606 GN=LGALS4 PE=1 SV=1 |
| sp\|O94832\|MYO1D_HUMAN | cyto | Unconventional myosin-Id OS=Homo sapiens OX=9606 GN=MYO1D PE=1 SV=2 |
| sp\|Q9BYZ8\|REG4_HUMAN | extr | Regenerating islet-derived protein 4 OS=Homo sapiens OX=9606 GN=REG4 PE=1 SV=1 |
| sp\|P16615\|AT2A2_HUMAN | plas | Sarcoplasmic/endoplasmic reticulum calcium ATPase 2 OS=Homo sapiens OX=9606 GN=ATP2A2 PE=1 SV=1 |
| sp\|P62841\|RS15_HUMAN | cyto | 40S ribosomal protein S15 OS=Homo sapiens OX=9606 GN=RPS15 PE=1 SV=2 |
| sp\|P52292\|IMA1_HUMAN | cyto_nucl | Importin subunit alpha-1 OS=Homo sapiens OX=9606 GN=KPNA2 PE=1 SV=1 |
| sp\|Q8IY22\|CMIP_HUMAN | cyto | C-Maf-inducing protein OS=Homo sapiens OX=9606 GN=CMIP PE=1 SV=3 |
| sp\|A0A0C4DH34\|HV428_HUMAN | extr | Immunoglobulin heavy variable 4-28 OS=Homo sapiens OX=9606 GN=IGHV4-28 PE=3 SV=1 |
| sp\|Q9Y2K7\|KDM2A_HUMAN | cyto_nucl | Lysine-specific demethylase 2A OS=Homo sapiens OX=9606 GN=KDM2A PE=1 SV=3 |
| sp\|P02792\|FRIL_HUMAN | cyto | Ferritin light chain OS=Homo sapiens OX=9606 GN=FTL PE=1 SV=2 |
| sp\|P61619\|S61A1_HUMAN | plas | Protein transport protein Sec61 subunit alpha isoform 1 OS=Homo sapiens OX=9606 GN=SEC61A1 PE=1 SV=2 |
| sp\|P52788\|SPSY_HUMAN | cyto | Spermine synthase OS=Homo sapiens OX=9606 GN=SMS PE=1 SV=2 |
| sp\|P28300\|LYOX_HUMAN | extr | Protein-lysine 6-oxidase OS=Homo sapiens OX=9606 GN=LOX PE=1 SV=2 |
| sp\|Q9ULC4\|MCTS1_HUMAN | cyto | Malignant T-cell-amplified sequence 1 OS=Homo sapiens OX=9606 GN=MCTS1 PE=1 SV=1 |
| sp\|Q9UHB6\|LIMA1_HUMAN | nucl | LIM domain and actin-binding protein 1 OS=Homo sapiens OX=9606 GN=LIMA1 PE=1 SV=1 |
| sp\|P03886\|NU1M_HUMAN | plas | NADH-ubiquinone oxidoreductase chain 1 OS=Homo sapiens OX=9606 GN=MT-ND1 PE=1 SV=1 |
| sp\|P01130\|LDLR_HUMAN | pero | Low-density lipoprotein receptor OS=Homo sapiens OX=9606 GN=LDLR PE=1 SV=1 |
| sp\|P63218\|GBG5_HUMAN | extr | Guanine nucleotide-binding protein G(I)/G(S)/G(O) subunit gamma-5 OS=Homo sapiens OX=9606 GN=GNG5 PE=1 SV=3 |
| sp\|Q04912\|RON_HUMAN | extr | Macrophage-stimulating protein receptor OS=Homo sapiens OX=9606 GN=MST1R PE=1 SV=3 |
| sp\|Q5VYY1\|ANR22_HUMAN | cyto | Ankyrin repeat domain-containing protein 22 OS=Homo sapiens OX=9606 GN=ANKRD22 PE=2 SV=1 |
| sp\|Q07021\|C1QBP_HUMAN | mito | Complement component 1 Q subcomponent-binding protein, mitochondrial OS=Homo sapiens OX=9606 GN=C1QBP PE=1 SV=1 |
| sp\|Q8N5M1\|ATPF2_HUMAN | mito | ATP synthase mitochondrial F1 complex assembly factor 2 OS=Homo sapiens OX=9606 GN=ATPAF2 PE=1 SV=1 |
| sp\|O75448\|MED24_HUMAN | cyto | Mediator of RNA polymerase II transcription subunit 24 OS=Homo sapiens OX=9606 GN=MED24 PE=1 SV=1 |
| sp\|P10721\|KIT_HUMAN | E.R. | Mast/stem cell growth factor receptor Kit OS=Homo sapiens OX=9606 GN=KIT PE=1 SV=1 |
| sp\|O15514\|RPB4_HUMAN | cyto | DNA-directed RNA polymerase II subunit RPB4 OS=Homo sapiens OX=9606 GN=POLR2D PE=1 SV=1 |
| sp\|Q16873\|LTC4S_HUMAN | extr_plas | Leukotriene C4 synthase OS=Homo sapiens OX=9606 GN=LTC4S PE=1 SV=1 |
| sp\|Q9UM47\|NOTC3_HUMAN | plas | Neurogenic locus notch homolog protein 3 OS=Homo sapiens OX=9606 GN=NOTCH3 PE=1 SV=2 |
| sp\|O43181\|NDUS4_HUMAN | mito | NADH dehydrogenase [ubiquinone] iron-sulfur protein 4, mitochondrial OS=Homo sapiens OX=9606 GN=NDUFS4 PE=1 SV=1 |
| sp\|Q9H875\|PKRI1_HUMAN | nucl | PRKR-interacting protein 1 OS=Homo sapiens OX=9606 GN=PRKRIP1 PE=1 SV=1 |
| sp\|Q6ZMG9\|CERS6_HUMAN | plas | Ceramide synthase 6 OS=Homo sapiens OX=9606 GN=CERS6 PE=1 SV=1 |
| sp\|P48729\|KC1A_HUMAN | cyto | Casein kinase I isoform alpha OS=Homo sapiens OX=9606 GN=CSNK1A1 PE=1 SV=2 |
| sp\|O43617\|TPPC3_HUMAN | mito | Trafficking protein particle complex subunit 3 OS=Homo sapiens OX=9606 GN=TRAPPC3 PE=1 SV=1 |
| sp\|P13688\|CEAM1_HUMAN | pero | Carcinoembryonic antigen-related cell adhesion molecule 1 OS=Homo sapiens OX=9606 GN=CEACAM1 PE=1 SV=2 |
| sp\|Q5W0V3\|F16B1_HUMAN | cyto | Protein FAM160B1 OS=Homo sapiens OX=9606 GN=FAM160B1 PE=1 SV=1 |
| sp\|Q15772\|SPEG_HUMAN | plas | Striated muscle preferentially expressed protein kinase OS=Homo sapiens OX=9606 GN=SPEG PE=1 SV=4 |
| sp\|P09936\|UCHL1_HUMAN | cyto | Ubiquitin carboxyl-terminal hydrolase isozyme L1 OS=Homo sapiens OX=9606 GN=UCHL1 PE=1 SV=2 |
| sp\|Q96GP6\|SREC2_HUMAN | plas | Scavenger receptor class F member 2 OS=Homo sapiens OX=9606 GN=SCARF2 PE=1 SV=5 |
| sp\|Q9Y2J8\|PADI2_HUMAN | mito | Protein-arginine deiminase type-2 OS=Homo sapiens OX=9606 GN=PADI2 PE=1 SV=2 |
| sp\|Q14409\|GLPK3_HUMAN | pero | Glycerol kinase 3 OS=Homo sapiens OX=9606 GN=GK3P PE=2 SV=2 |
| sp\|Q6UX01\|LMBRL_HUMAN | plas | Protein LMBR1L OS=Homo sapiens OX=9606 GN=LMBR1L PE=1 SV=2 |
| sp\|Q13838\|DX39B_HUMAN | cyto_nucl | Spliceosome RNA helicase DDX39B OS=Homo sapiens OX=9606 GN=DDX39B PE=1 SV=1 |
| sp\|O15523\|DDX3Y_HUMAN | nucl | ATP-dependent RNA helicase DDX3Y OS=Homo sapiens OX=9606 GN=DDX3Y PE=1 SV=2 |
| sp\|O43242\|PSMD3_HUMAN | cyto | 26S proteasome non-ATPase regulatory subunit 3 OS=Homo sapiens OX=9606 GN=PSMD3 PE=1 SV=2 |
| sp\|O75052\|CAPON_HUMAN | nucl | Carboxyl-terminal PDZ ligand of neuronal nitric oxide synthase protein OS=Homo sapiens OX=9606 GN=NOS1AP PE=1 SV=3 |
| sp\|Q8N1B4\|VPS52_HUMAN | cyto | Vacuolar protein sorting-associated protein 52 homolog OS=Homo sapiens OX=9606 GN=VPS52 PE=1 SV=1 |
| sp\|Q14376\|GALE_HUMAN | cyto | UDP-glucose 4-epimerase OS=Homo sapiens OX=9606 GN=GALE PE=1 SV=2 |
| sp\|Q9Y5M8\|SRPRB_HUMAN | cyto | Signal recognition particle receptor subunit beta OS=Homo sapiens OX=9606 GN=SRPRB PE=1 SV=3 |
| sp\|Q5H8C1\|FREM1_HUMAN | extr | FRAS1-related extracellular matrix protein 1 OS=Homo sapiens OX=9606 GN=FREM1 PE=1 SV=3 |
| sp\|P37837\|TALDO_HUMAN | mito | Transaldolase OS=Homo sapiens OX=9606 GN=TALDO1 PE=1 SV=2 |
| sp\|Q9UM00\|TMCO1_HUMAN | extr | Calcium load-activated calcium channel OS=Homo sapiens OX=9606 GN=TMCO1 PE=1 SV=1 |
| sp\|Q9Y2E8\|SL9A8_HUMAN | plas | Sodium/hydrogen exchanger 8 OS=Homo sapiens OX=9606 GN=SLC9A8 PE=1 SV=4 |
| sp\|P02549\|SPTA1_HUMAN | cyto | Spectrin alpha chain, erythrocytic 1 OS=Homo sapiens OX=9606 GN=SPTA1 PE=1 SV=5 |
| sp\|Q07092\|COGA1_HUMAN | cyto | Collagen alpha-1(XVI) chain OS=Homo sapiens OX=9606 GN=COL16A1 PE=1 SV=2 |
| sp\|P62699\|YPEL5_HUMAN | cyto | Protein yippee-like 5 OS=Homo sapiens OX=9606 GN=YPEL5 PE=1 SV=1 |
| sp\|Q8NCF5\|NF2IP_HUMAN | nucl | NFATC2-interacting protein OS=Homo sapiens OX=9606 GN=NFATC2IP PE=1 SV=1 |
| sp\|P26572\|MGAT1_HUMAN | extr | Alpha-1,3-mannosyl-glycoprotein 2-beta-N-acetylglucosaminyltransferase OS=Homo sapiens OX=9606 GN=MGAT1 PE=1 SV=2 |
| sp\|Q5NDL2\|EOGT_HUMAN | E.R. | EGF domain-specific O-linked N-acetylglucosamine transferase OS=Homo sapiens OX=9606 GN=EOGT PE=1 SV=1 |
| sp\|O75164\|KDM4A_HUMAN | nucl | Lysine-specific demethylase 4A OS=Homo sapiens OX=9606 GN=KDM4A PE=1 SV=2 |
| sp\|Q9UBB6\|NCDN_HUMAN | plas | Neurochondrin OS=Homo sapiens OX=9606 GN=NCDN PE=1 SV=1 |
| sp\|Q15648\|MED1_HUMAN | nucl | Mediator of RNA polymerase II transcription subunit 1 OS=Homo sapiens OX=9606 GN=MED1 PE=1 SV=4 |
| sp\|Q9H9Y6\|RPA2_HUMAN | cyto | DNA-directed RNA polymerase I subunit RPA2 OS=Homo sapiens OX=9606 GN=POLR1B PE=1 SV=2 |
| sp\|Q93088\|BHMT1_HUMAN | cyto | Betaine--homocysteine S-methyltransferase 1 OS=Homo sapiens OX=9606 GN=BHMT PE=1 SV=2 |
| sp\|Q13085\|ACACA_HUMAN | cyto | Acetyl-CoA carboxylase 1 OS=Homo sapiens OX=9606 GN=ACACA PE=1 SV=2 |
| sp\|Q96BQ1\|FAM3D_HUMAN | extr | Protein FAM3D OS=Homo sapiens OX=9606 GN=FAM3D PE=1 SV=1 |
| sp\|P22676\|CALB2_HUMAN | cyto | Calretinin OS=Homo sapiens OX=9606 GN=CALB2 PE=2 SV=2 |
| sp\|Q7LG56\|RIR2B_HUMAN | cyto | Ribonucleoside-diphosphate reductase subunit M2 B OS=Homo sapiens OX=9606 GN=RRM2B PE=1 SV=1 |
| sp\|P62140\|PP1B_HUMAN | cyto | Serine/threonine-protein phosphatase PP1-beta catalytic subunit OS=Homo sapiens OX=9606 GN=PPP1CB PE=1 SV=3 |
| sp\|Q9Y5J5\|PHLA3_HUMAN | cyto | Pleckstrin homology-like domain family A member 3 OS=Homo sapiens OX=9606 GN=PHLDA3 PE=1 SV=1 |
| sp\|O15127\|SCAM2_HUMAN | plas | Secretory carrier-associated membrane protein 2 OS=Homo sapiens OX=9606 GN=SCAMP2 PE=1 SV=2 |
| sp\|P52566\|GDIR2_HUMAN | cyto | Rho GDP-dissociation inhibitor 2 OS=Homo sapiens OX=9606 GN=ARHGDIB PE=1 SV=3 |
| sp\|Q9BTY2\|FUCO2_HUMAN | extr | Plasma alpha-L-fucosidase OS=Homo sapiens OX=9606 GN=FUCA2 PE=1 SV=2 |
| sp\|Q9NR28\|DBLOH_HUMAN | mito | Diablo homolog, mitochondrial OS=Homo sapiens OX=9606 GN=DIABLO PE=1 SV=1 |
| sp\|Q10570\|CPSF1_HUMAN | nucl | Cleavage and polyadenylation specificity factor subunit 1 OS=Homo sapiens OX=9606 GN=CPSF1 PE=1 SV=2 |
| sp\|P53621\|COPA_HUMAN | cyto | Coatomer subunit alpha OS=Homo sapiens OX=9606 GN=COPA PE=1 SV=2 |
| sp\|Q9H0M0\|WWP1_HUMAN | nucl | NEDD4-like E3 ubiquitin-protein ligase WWP1 OS=Homo sapiens OX=9606 GN=WWP1 PE=1 SV=1 |
| sp\|Q8N465\|D2HDH_HUMAN | mito | D-2-hydroxyglutarate dehydrogenase, mitochondrial OS=Homo sapiens OX=9606 GN=D2HGDH PE=1 SV=3 |
| sp\|P18283\|GPX2_HUMAN | cyto | Glutathione peroxidase 2 OS=Homo sapiens OX=9606 GN=GPX2 PE=1 SV=3 |
| sp\|Q96A29\|FUCT1_HUMAN | plas | GDP-fucose transporter 1 OS=Homo sapiens OX=9606 GN=SLC35C1 PE=1 SV=1 |
| sp\|P20594\|ANPRB_HUMAN | E.R. | Atrial natriuretic peptide receptor 2 OS=Homo sapiens OX=9606 GN=NPR2 PE=1 SV=1 |
| sp\|P13761\|2B17_HUMAN | extr | HLA class II histocompatibility antigen, DRB1-7 beta chain OS=Homo sapiens OX=9606 GN=HLA-DRB1 PE=1 SV=1 |
| sp\|Q8IVU3\|HERC6_HUMAN | nucl | Probable E3 ubiquitin-protein ligase HERC6 OS=Homo sapiens OX=9606 GN=HERC6 PE=1 SV=2 |
| sp\|Q9Y5Y7\|LYVE1_HUMAN | mito | Lymphatic vessel endothelial hyaluronic acid receptor 1 OS=Homo sapiens OX=9606 GN=LYVE1 PE=1 SV=2 |
| sp\|P62244\|RS15A_HUMAN | cyto | 40S ribosomal protein S15a OS=Homo sapiens OX=9606 GN=RPS15A PE=1 SV=2 |
| sp\|Q9NSI8\|SAMN1_HUMAN | nucl | SAM domain-containing protein SAMSN-1 OS=Homo sapiens OX=9606 GN=SAMSN1 PE=1 SV=1 |
| sp\|P21217\|FUT3_HUMAN | extr | Galactoside 3(4)-L-fucosyltransferase OS=Homo sapiens OX=9606 GN=FUT3 PE=2 SV=1 |
| sp\|Q13423\|NNTM_HUMAN | plas | NAD(P) transhydrogenase, mitochondrial OS=Homo sapiens OX=9606 GN=NNT PE=1 SV=3 |
| sp\|Q96QR8\|PURB_HUMAN | cyto | Transcriptional activator protein Pur-beta OS=Homo sapiens OX=9606 GN=PURB PE=1 SV=3 |
| sp\|Q92629\|SGCD_HUMAN | mito | Delta-sarcoglycan OS=Homo sapiens OX=9606 GN=SGCD PE=1 SV=2 |
| sp\|Q8N0U8\|VKORL_HUMAN | plas | Vitamin K epoxide reductase complex subunit 1-like protein 1 OS=Homo sapiens OX=9606 GN=VKORC1L1 PE=1 SV=2 |
| sp\|Q92845\|KIFA3_HUMAN | cyto | Kinesin-associated protein 3 OS=Homo sapiens OX=9606 GN=KIFAP3 PE=1 SV=2 |
| sp\|Q9NRD5\|PICK1_HUMAN | cyto | PRKCA-binding protein OS=Homo sapiens OX=9606 GN=PICK1 PE=1 SV=2 |
| sp\|Q8TBZ0\|CC110_HUMAN | nucl | Coiled-coil domain-containing protein 110 OS=Homo sapiens OX=9606 GN=CCDC110 PE=1 SV=1 |
| sp\|P53794\|SC5A3_HUMAN | plas | Sodium/myo-inositol cotransporter OS=Homo sapiens OX=9606 GN=SLC5A3 PE=1 SV=2 |
| sp\|Q969H8\|MYDGF_HUMAN | extr | Myeloid-derived growth factor OS=Homo sapiens OX=9606 GN=MYDGF PE=1 SV=1 |
| sp\|Q15434\|RBMS2_HUMAN | nucl | RNA-binding motif, single-stranded-interacting protein 2 OS=Homo sapiens OX=9606 GN=RBMS2 PE=1 SV=1 |
| sp\|Q13155\|AIMP2_HUMAN | cyto | Aminoacyl tRNA synthase complex-interacting multifunctional protein 2 OS=Homo sapiens OX=9606 GN=AIMP2 PE=1 SV=2 |
| sp\|O75112\|LDB3_HUMAN | nucl | LIM domain-binding protein 3 OS=Homo sapiens OX=9606 GN=LDB3 PE=1 SV=2 |
| sp\|Q9Y6R4\|M3K4_HUMAN | plas | Mitogen-activated protein kinase kinase kinase 4 OS=Homo sapiens OX=9606 GN=MAP3K4 PE=1 SV=2 |
| sp\|P19801\|AOC1_HUMAN | extr | Amiloride-sensitive amine oxidase [copper-containing] OS=Homo sapiens OX=9606 GN=AOC1 PE=1 SV=4 |
| sp\|P61009\|SPCS3_HUMAN | extr | Signal peptidase complex subunit 3 OS=Homo sapiens OX=9606 GN=SPCS3 PE=1 SV=1 |
| sp\|Q13637\|RAB32_HUMAN | cyto | Ras-related protein Rab-32 OS=Homo sapiens OX=9606 GN=RAB32 PE=1 SV=3 |
| sp\|Q9P2B4\|CT2NL_HUMAN | nucl | CTTNBP2 N-terminal-like protein OS=Homo sapiens OX=9606 GN=CTTNBP2NL PE=1 SV=2 |
| sp\|P20337\|RAB3B_HUMAN | cyto | Ras-related protein Rab-3B OS=Homo sapiens OX=9606 GN=RAB3B PE=1 SV=2 |
| sp\|Q86WU2\|LDHD_HUMAN | mito | Probable D-lactate dehydrogenase, mitochondrial OS=Homo sapiens OX=9606 GN=LDHD PE=1 SV=1 |
| sp\|Q9UI14\|PRAF1_HUMAN | plas | Prenylated Rab acceptor protein 1 OS=Homo sapiens OX=9606 GN=RABAC1 PE=1 SV=1 |
| sp\|P35442\|TSP2_HUMAN | extr | Thrombospondin-2 OS=Homo sapiens OX=9606 GN=THBS2 PE=1 SV=2 |
| sp\|Q6P1X5\|TAF2_HUMAN | nucl | Transcription initiation factor TFIID subunit 2 OS=Homo sapiens OX=9606 GN=TAF2 PE=1 SV=3 |
| sp\|Q8WZA1\|PMGT1_HUMAN | plas | Protein O-linked-mannose beta-1,2-N-acetylglucosaminyltransferase 1 OS=Homo sapiens OX=9606 GN=POMGNT1 PE=1 SV=2 |
| sp\|Q8NBX0\|SCPDL_HUMAN | cyto | Saccharopine dehydrogenase-like oxidoreductase OS=Homo sapiens OX=9606 GN=SCCPDH PE=1 SV=1 |
| sp\|P07355\|ANXA2_HUMAN | cyto | Annexin A2 OS=Homo sapiens OX=9606 GN=ANXA2 PE=1 SV=2 |
| sp\|O00422\|SAP18_HUMAN | nucl | Histone deacetylase complex subunit SAP18 OS=Homo sapiens OX=9606 GN=SAP18 PE=1 SV=1 |
| sp\|Q9Y2G8\|DJC16_HUMAN | E.R. | DnaJ homolog subfamily C member 16 OS=Homo sapiens OX=9606 GN=DNAJC16 PE=2 SV=3 |
| sp\|P13498\|CY24A_HUMAN | plas | Cytochrome b-245 light chain OS=Homo sapiens OX=9606 GN=CYBA PE=1 SV=3 |
| sp\|Q641Q2\|WAC2A_HUMAN | nucl | WASH complex subunit 2A OS=Homo sapiens OX=9606 GN=WASHC2A PE=1 SV=3 |
| sp\|Q9NWB6\|ARGL1_HUMAN | nucl | Arginine and glutamate-rich protein 1 OS=Homo sapiens OX=9606 GN=ARGLU1 PE=1 SV=1 |
| sp\|Q9UIQ6\|LCAP_HUMAN | cyto | Leucyl-cystinyl aminopeptidase OS=Homo sapiens OX=9606 GN=LNPEP PE=1 SV=3 |
| sp\|Q9UQ84\|EXO1_HUMAN | nucl | Exonuclease 1 OS=Homo sapiens OX=9606 GN=EXO1 PE=1 SV=2 |
| sp\|P20962\|PTMS_HUMAN | nucl | Parathymosin OS=Homo sapiens OX=9606 GN=PTMS PE=1 SV=2 |
| sp\|O94876\|TMCC1_HUMAN | plas | Transmembrane and coiled-coil domains protein 1 OS=Homo sapiens OX=9606 GN=TMCC1 PE=1 SV=3 |
| sp\|P23434\|GCSH_HUMAN | mito | Glycine cleavage system H protein, mitochondrial OS=Homo sapiens OX=9606 GN=GCSH PE=1 SV=2 |
| sp\|Q6ZRQ5\|MMS22_HUMAN | cyto | Protein MMS22-like OS=Homo sapiens OX=9606 GN=MMS22L PE=1 SV=3 |
| sp\|P17405\|ASM_HUMAN | plas | Sphingomyelin phosphodiesterase OS=Homo sapiens OX=9606 GN=SMPD1 PE=1 SV=5 |
| sp\|P40394\|ADH7_HUMAN | cyto | Alcohol dehydrogenase class 4 mu/sigma chain OS=Homo sapiens OX=9606 GN=ADH7 PE=1 SV=2 |
| sp\|P39656\|OST48_HUMAN | plas | Dolichyl-diphosphooligosaccharide--protein glycosyltransferase 48 kDa subunit OS=Homo sapiens OX=9606 GN=DDOST PE=1 SV=4 |
| sp\|Q9NW08\|RPC2_HUMAN | cyto | DNA-directed RNA polymerase III subunit RPC2 OS=Homo sapiens OX=9606 GN=POLR3B PE=1 SV=2 |
| sp\|P13224\|GP1BB_HUMAN | extr | Platelet glycoprotein Ib beta chain OS=Homo sapiens OX=9606 GN=GP1BB PE=1 SV=1 |
| sp\|O95670\|VATG2_HUMAN | nucl | V-type proton ATPase subunit G 2 OS=Homo sapiens OX=9606 GN=ATP6V1G2 PE=1 SV=1 |
| sp\|Q6NXT4\|ZNT6_HUMAN | plas | Zinc transporter 6 OS=Homo sapiens OX=9606 GN=SLC30A6 PE=1 SV=2 |
| sp\|Q86YL5\|TDRP_HUMAN | cyto | Testis development-related protein OS=Homo sapiens OX=9606 GN=TDRP PE=1 SV=2 |
| sp\|P55036\|PSMD4_HUMAN | cysk | 26S proteasome non-ATPase regulatory subunit 4 OS=Homo sapiens OX=9606 GN=PSMD4 PE=1 SV=1 |
| sp\|P51153\|RAB13_HUMAN | cyto | Ras-related protein Rab-13 OS=Homo sapiens OX=9606 GN=RAB13 PE=1 SV=1 |
| sp\|P01593\|KVD33_HUMAN | extr | Immunoglobulin kappa variable 1D-33 OS=Homo sapiens OX=9606 GN=IGKV1D-33 PE=1 SV=2 >sp\|P01594\|KV133_HUMAN Immunoglobulin kappa variable 1-33 OS=Homo sapiens OX=9606 GN=IGKV1-33 PE=1 SV=2 |
| sp\|Q9NR48\|ASH1L_HUMAN | nucl | Histone-lysine N-methyltransferase ASH1L OS=Homo sapiens OX=9606 GN=ASH1L PE=1 SV=2 |
| sp\|Q9BZH6\|WDR11_HUMAN | nucl | WD repeat-containing protein 11 OS=Homo sapiens OX=9606 GN=WDR11 PE=1 SV=1 |
| sp\|P63092\|GNAS2_HUMAN | cyto | Guanine nucleotide-binding protein G(s) subunit alpha isoforms short OS=Homo sapiens OX=9606 GN=GNAS PE=1 SV=1 |
| sp\|O75298\|RTN2_HUMAN | plas | Reticulon-2 OS=Homo sapiens OX=9606 GN=RTN2 PE=1 SV=1 |
| sp\|P82979\|SARNP_HUMAN | nucl | SAP domain-containing ribonucleoprotein OS=Homo sapiens OX=9606 GN=SARNP PE=1 SV=3 |
| sp\|P21266\|GSTM3_HUMAN | cyto | Glutathione S-transferase Mu 3 OS=Homo sapiens OX=9606 GN=GSTM3 PE=1 SV=3 |
| sp\|Q96CW6\|S7A6O_HUMAN | mito | Probable RNA polymerase II nuclear localization protein SLC7A6OS OS=Homo sapiens OX=9606 GN=SLC7A6OS PE=1 SV=2 |
| sp\|P13637\|AT1A3_HUMAN | plas | Sodium/potassium-transporting ATPase subunit alpha-3 OS=Homo sapiens OX=9606 GN=ATP1A3 PE=1 SV=3 |
| sp\|Q9UM13\|APC10_HUMAN | cyto | Anaphase-promoting complex subunit 10 OS=Homo sapiens OX=9606 GN=ANAPC10 PE=1 SV=1 |
| sp\|Q13488\|VPP3_HUMAN | plas | V-type proton ATPase 116 kDa subunit a isoform 3 OS=Homo sapiens OX=9606 GN=TCIRG1 PE=1 SV=3 |
| sp\|Q16537\|2A5E_HUMAN | cyto | Serine/threonine-protein phosphatase 2A 56 kDa regulatory subunit epsilon isoform OS=Homo sapiens OX=9606 GN=PPP2R5E PE=1 SV=1 |
| sp\|Q2UY09\|COSA1_HUMAN | mito | Collagen alpha-1(XXVIII) chain OS=Homo sapiens OX=9606 GN=COL28A1 PE=2 SV=2 |
| sp\|Q9H0U3\|MAGT1_HUMAN | plas | Magnesium transporter protein 1 OS=Homo sapiens OX=9606 GN=MAGT1 PE=1 SV=1 |
| sp\|Q96FX7\|TRM61_HUMAN | nucl | tRNA (adenine(58)-N(1))-methyltransferase catalytic subunit TRMT61A OS=Homo sapiens OX=9606 GN=TRMT61A PE=1 SV=1 |
| sp\|Q58EX2\|SDK2_HUMAN | pero | Protein sidekick-2 OS=Homo sapiens OX=9606 GN=SDK2 PE=1 SV=3 |
| sp\|P09486\|SPRC_HUMAN | extr | SPARC OS=Homo sapiens OX=9606 GN=SPARC PE=1 SV=1 |
| sp\|Q8WVD5\|RN141_HUMAN | nucl | RING finger protein 141 OS=Homo sapiens OX=9606 GN=RNF141 PE=1 SV=1 |
| sp\|Q13424\|SNTA1_HUMAN | mito | Alpha-1-syntrophin OS=Homo sapiens OX=9606 GN=SNTA1 PE=1 SV=1 |
| sp\|P08123\|CO1A2_HUMAN | extr | Collagen alpha-2(I) chain OS=Homo sapiens OX=9606 GN=COL1A2 PE=1 SV=7 |
| sp\|Q13882\|PTK6_HUMAN | cyto | Protein-tyrosine kinase 6 OS=Homo sapiens OX=9606 GN=PTK6 PE=1 SV=1 |
| sp\|Q9BUE0\|MED18_HUMAN | cyto | Mediator of RNA polymerase II transcription subunit 18 OS=Homo sapiens OX=9606 GN=MED18 PE=1 SV=1 |
| sp\|Q9HBI0\|PARVG_HUMAN | cyto | Gamma-parvin OS=Homo sapiens OX=9606 GN=PARVG PE=1 SV=1 |
| sp\|Q8N766\|EMC1_HUMAN | pero | ER membrane protein complex subunit 1 OS=Homo sapiens OX=9606 GN=EMC1 PE=1 SV=1 |
| sp\|Q96G23\|CERS2_HUMAN | plas | Ceramide synthase 2 OS=Homo sapiens OX=9606 GN=CERS2 PE=1 SV=1 |
| sp\|Q15008\|PSMD6_HUMAN | cyto | 26S proteasome non-ATPase regulatory subunit 6 OS=Homo sapiens OX=9606 GN=PSMD6 PE=1 SV=1 |
| sp\|Q96SL4\|GPX7_HUMAN | extr | Glutathione peroxidase 7 OS=Homo sapiens OX=9606 GN=GPX7 PE=1 SV=1 |
| sp\|Q96S42\|NODAL_HUMAN | extr | Nodal homolog OS=Homo sapiens OX=9606 GN=NODAL PE=1 SV=2 |
| sp\|P47897\|SYQ_HUMAN | cyto | Glutamine--tRNA ligase OS=Homo sapiens OX=9606 GN=QARS PE=1 SV=1 |
| sp\|Q16514\|TAF12_HUMAN | cyto | Transcription initiation factor TFIID subunit 12 OS=Homo sapiens OX=9606 GN=TAF12 PE=1 SV=1 |
| sp\|P08195\|4F2_HUMAN | E.R. | 4F2 cell-surface antigen heavy chain OS=Homo sapiens OX=9606 GN=SLC3A2 PE=1 SV=3 |
| sp\|Q9Y257\|KCNK6_HUMAN | plas | Potassium channel subfamily K member 6 OS=Homo sapiens OX=9606 GN=KCNK6 PE=1 SV=1 |
| sp\|O43776\|SYNC_HUMAN | cyto | Asparagine--tRNA ligase, cytoplasmic OS=Homo sapiens OX=9606 GN=NARS PE=1 SV=1 |
| sp\|O75436\|VP26A_HUMAN | cyto | Vacuolar protein sorting-associated protein 26A OS=Homo sapiens OX=9606 GN=VPS26A PE=1 SV=2 |
| sp\|O14772\|FPGT_HUMAN | cyto | Fucose-1-phosphate guanylyltransferase OS=Homo sapiens OX=9606 GN=FPGT PE=1 SV=3 |
| sp\|A0A087WW87\|KV240_HUMAN | extr | Immunoglobulin kappa variable 2-40 OS=Homo sapiens OX=9606 GN=IGKV2-40 PE=3 SV=2 >sp\|P01614\|KVD40_HUMAN Immunoglobulin kappa variable 2D-40 OS=Homo sapiens OX=9606 GN=IGKV2D-40 PE=1 SV=2 |
| sp\|Q9BZF1\|OSBL8_HUMAN | cyto | Oxysterol-binding protein-related protein 8 OS=Homo sapiens OX=9606 GN=OSBPL8 PE=1 SV=3 |
| sp\|Q14728\|MFS10_HUMAN | plas | Major facilitator superfamily domain-containing protein 10 OS=Homo sapiens OX=9606 GN=MFSD10 PE=1 SV=1 |
| sp\|Q9NVH2\|INT7_HUMAN | plas | Integrator complex subunit 7 OS=Homo sapiens OX=9606 GN=INTS7 PE=1 SV=1 |
| sp\|P02671\|FIBA_HUMAN | extr | Fibrinogen alpha chain OS=Homo sapiens OX=9606 GN=FGA PE=1 SV=2 |
| sp\|Q96PP4\|TSG13_HUMAN | nucl | Testis-specific gene 13 protein OS=Homo sapiens OX=9606 GN=TSGA13 PE=2 SV=1 |
| sp\|P60033\|CD81_HUMAN | plas | CD81 antigen OS=Homo sapiens OX=9606 GN=CD81 PE=1 SV=1 |
| sp\|Q6UWY5\|OLFL1_HUMAN | E.R. | Olfactomedin-like protein 1 OS=Homo sapiens OX=9606 GN=OLFML1 PE=1 SV=2 |
| sp\|Q8NBQ5\|DHB11_HUMAN | E.R. | Estradiol 17-beta-dehydrogenase 11 OS=Homo sapiens OX=9606 GN=HSD17B11 PE=1 SV=3 |
| sp\|Q9Y3V2\|RWDD3_HUMAN | cyto | RWD domain-containing protein 3 OS=Homo sapiens OX=9606 GN=RWDD3 PE=1 SV=4 |
| sp\|O60762\|DPM1_HUMAN | nucl | Dolichol-phosphate mannosyltransferase subunit 1 OS=Homo sapiens OX=9606 GN=DPM1 PE=1 SV=1 |
| sp\|Q9UQ03\|COR2B_HUMAN | nucl | Coronin-2B OS=Homo sapiens OX=9606 GN=CORO2B PE=1 SV=4 |
| sp\|Q9BPZ3\|PAIP2_HUMAN | cyto_nucl | Polyadenylate-binding protein-interacting protein 2 OS=Homo sapiens OX=9606 GN=PAIP2 PE=1 SV=1 |
| sp\|Q86YJ7\|AN13B_HUMAN | nucl | Ankyrin repeat domain-containing protein 13B OS=Homo sapiens OX=9606 GN=ANKRD13B PE=1 SV=4 |
| sp\|Q9H081\|MIS12_HUMAN | cyto | Protein MIS12 homolog OS=Homo sapiens OX=9606 GN=MIS12 PE=1 SV=1 |
| sp\|Q9Y279\|VSIG4_HUMAN | plas | V-set and immunoglobulin domain-containing protein 4 OS=Homo sapiens OX=9606 GN=VSIG4 PE=1 SV=1 |
| sp\|Q16270\|IBP7_HUMAN | extr | Insulin-like growth factor-binding protein 7 OS=Homo sapiens OX=9606 GN=IGFBP7 PE=1 SV=1 |
| sp\|Q9H8H3\|MET7A_HUMAN | extr | Methyltransferase-like protein 7A OS=Homo sapiens OX=9606 GN=METTL7A PE=1 SV=1 |
| sp\|O95294\|RASL1_HUMAN | nucl | RasGAP-activating-like protein 1 OS=Homo sapiens OX=9606 GN=RASAL1 PE=1 SV=3 |
| sp\|Q4V9L6\|TM119_HUMAN | extr | Transmembrane protein 119 OS=Homo sapiens OX=9606 GN=TMEM119 PE=1 SV=1 |
| sp\|Q9Y6K5\|OAS3_HUMAN | cyto_nucl | 2'-5'-oligoadenylate synthase 3 OS=Homo sapiens OX=9606 GN=OAS3 PE=1 SV=3 |
| sp\|Q13232\|NDK3_HUMAN | extr | Nucleoside diphosphate kinase 3 OS=Homo sapiens OX=9606 GN=NME3 PE=1 SV=2 |
| sp\|Q15642\|CIP4_HUMAN | nucl | Cdc42-interacting protein 4 OS=Homo sapiens OX=9606 GN=TRIP10 PE=1 SV=3 |
| sp\|Q5QJE6\|TDIF2_HUMAN | nucl | Deoxynucleotidyltransferase terminal-interacting protein 2 OS=Homo sapiens OX=9606 GN=DNTTIP2 PE=1 SV=2 |
| sp\|Q9NTJ5\|SAC1_HUMAN | plas | Phosphatidylinositide phosphatase SAC1 OS=Homo sapiens OX=9606 GN=SACM1L PE=1 SV=2 |
| sp\|O94769\|ECM2_HUMAN | extr | Extracellular matrix protein 2 OS=Homo sapiens OX=9606 GN=ECM2 PE=2 SV=1 |
| sp\|O95865\|DDAH2_HUMAN | cyto_nucl | N(G),N(G)-dimethylarginine dimethylaminohydrolase 2 OS=Homo sapiens OX=9606 GN=DDAH2 PE=1 SV=1 |
| sp\|Q8IYS2\|K2013_HUMAN | plas | Uncharacterized protein KIAA2013 OS=Homo sapiens OX=9606 GN=KIAA2013 PE=1 SV=1 |
| sp\|Q14997\|PSME4_HUMAN | nucl | Proteasome activator complex subunit 4 OS=Homo sapiens OX=9606 GN=PSME4 PE=1 SV=2 |
| sp\|Q9NW68\|BSDC1_HUMAN | cyto | BSD domain-containing protein 1 OS=Homo sapiens OX=9606 GN=BSDC1 PE=1 SV=1 |
| sp\|P05164\|PERM_HUMAN | extr | Myeloperoxidase OS=Homo sapiens OX=9606 GN=MPO PE=1 SV=1 |
| sp\|O60496\|DOK2_HUMAN | nucl | Docking protein 2 OS=Homo sapiens OX=9606 GN=DOK2 PE=1 SV=2 |
| sp\|Q9HC21\|TPC_HUMAN | cyto | Mitochondrial thiamine pyrophosphate carrier OS=Homo sapiens OX=9606 GN=SLC25A19 PE=1 SV=1 |
| sp\|Q8N3U4\|STAG2_HUMAN | nucl | Cohesin subunit SA-2 OS=Homo sapiens OX=9606 GN=STAG2 PE=1 SV=3 |
| sp\|Q7Z4H3\|HDDC2_HUMAN | mito | HD domain-containing protein 2 OS=Homo sapiens OX=9606 GN=HDDC2 PE=1 SV=1 |
| sp\|Q14BN4\|SLMAP_HUMAN | pero | Sarcolemmal membrane-associated protein OS=Homo sapiens OX=9606 GN=SLMAP PE=1 SV=1 |
| sp\|Q76M96\|CCD80_HUMAN | mito | Coiled-coil domain-containing protein 80 OS=Homo sapiens OX=9606 GN=CCDC80 PE=1 SV=1 |
| sp\|Q9BXJ9\|NAA15_HUMAN | cyto | N-alpha-acetyltransferase 15, NatA auxiliary subunit OS=Homo sapiens OX=9606 GN=NAA15 PE=1 SV=1 |
| sp\|A6NHL2\|TBAL3_HUMAN | cysk | Tubulin alpha chain-like 3 OS=Homo sapiens OX=9606 GN=TUBAL3 PE=1 SV=2 |
| sp\|Q92538\|GBF1_HUMAN | cyto | Golgi-specific brefeldin A-resistance guanine nucleotide exchange factor 1 OS=Homo sapiens OX=9606 GN=GBF1 PE=1 SV=2 |
| sp\|P20702\|ITAX_HUMAN | extr | Integrin alpha-X OS=Homo sapiens OX=9606 GN=ITGAX PE=1 SV=3 |
| sp\|Q9BTM1\|H2AJ_HUMAN | nucl | Histone H2A.J OS=Homo sapiens OX=9606 GN=H2AFJ PE=1 SV=1 |
| sp\|Q9BUP0\|EFHD1_HUMAN | nucl | EF-hand domain-containing protein D1 OS=Homo sapiens OX=9606 GN=EFHD1 PE=1 SV=1 |
| sp\|Q5T619\|ZN648_HUMAN | nucl | Zinc finger protein 648 OS=Homo sapiens OX=9606 GN=ZNF648 PE=2 SV=1 |
| sp\|Q8IZV5\|RDH10_HUMAN | extr | Retinol dehydrogenase 10 OS=Homo sapiens OX=9606 GN=RDH10 PE=1 SV=1 |
| sp\|O15014\|ZN609_HUMAN | nucl | Zinc finger protein 609 OS=Homo sapiens OX=9606 GN=ZNF609 PE=1 SV=2 |
| sp\|P17661\|DESM_HUMAN | mito | Desmin OS=Homo sapiens OX=9606 GN=DES PE=1 SV=3 |
| sp\|Q13614\|MTMR2_HUMAN | nucl | Myotubularin-related protein 2 OS=Homo sapiens OX=9606 GN=MTMR2 PE=1 SV=4 |
| sp\|P63165\|SUMO1_HUMAN | cyto | Small ubiquitin-related modifier 1 OS=Homo sapiens OX=9606 GN=SUMO1 PE=1 SV=1 |
| sp\|Q6UXV4\|MIC27_HUMAN | mito | MICOS complex subunit MIC27 OS=Homo sapiens OX=9606 GN=APOOL PE=1 SV=1 |
| sp\|Q9UKD2\|MRT4_HUMAN | nucl | mRNA turnover protein 4 homolog OS=Homo sapiens OX=9606 GN=MRTO4 PE=1 SV=2 |
| sp\|Q8N9Z2\|CC71L_HUMAN | mito | Coiled-coil domain-containing protein 71L OS=Homo sapiens OX=9606 GN=CCDC71L PE=1 SV=2 |
| sp\|Q03426\|KIME_HUMAN | extr | Mevalonate kinase OS=Homo sapiens OX=9606 GN=MVK PE=1 SV=1 |
| sp\|O60831\|PRAF2_HUMAN | plas | PRA1 family protein 2 OS=Homo sapiens OX=9606 GN=PRAF2 PE=1 SV=1 |
| sp\|Q96AA3\|RFT1_HUMAN | plas | Protein RFT1 homolog OS=Homo sapiens OX=9606 GN=RFT1 PE=1 SV=1 |
| sp\|A0A075B6I0\|LV861_HUMAN | extr | Immunoglobulin lambda variable 8-61 OS=Homo sapiens OX=9606 GN=IGLV8-61 PE=3 SV=7 |
| sp\|P04424\|ARLY_HUMAN | cyto | Argininosuccinate lyase OS=Homo sapiens OX=9606 GN=ASL PE=1 SV=4 |
| sp\|P62341\|SELT_HUMAN | plas | Thioredoxin reductase-like selenoprotein T OS=Homo sapiens OX=9606 GN=SELENOT PE=1 SV=2 |
| sp\|P51993\|FUT6_HUMAN | extr | Alpha-(1,3)-fucosyltransferase 6 OS=Homo sapiens OX=9606 GN=FUT6 PE=1 SV=1 |
| sp\|Q99932\|SPAG8_HUMAN | nucl | Sperm-associated antigen 8 OS=Homo sapiens OX=9606 GN=SPAG8 PE=1 SV=3 |
| sp\|O15540\|FABP7_HUMAN | cyto | Fatty acid-binding protein, brain OS=Homo sapiens OX=9606 GN=FABP7 PE=1 SV=3 |
| sp\|Q969L2\|MAL2_HUMAN | plas | Protein MAL2 OS=Homo sapiens OX=9606 GN=MAL2 PE=1 SV=1 |
| sp\|Q99643\|C560_HUMAN | plas | Succinate dehydrogenase cytochrome b560 subunit, mitochondrial OS=Homo sapiens OX=9606 GN=SDHC PE=1 SV=1 |
| sp\|Q6ZN84\|CCD81_HUMAN | cyto | Coiled-coil domain-containing protein 81 OS=Homo sapiens OX=9606 GN=CCDC81 PE=2 SV=2 |
| sp\|O95295\|SNAPN_HUMAN | cyto | SNARE-associated protein Snapin OS=Homo sapiens OX=9606 GN=SNAPIN PE=1 SV=1 |
| sp\|P20132\|SDHL_HUMAN | cyto | L-serine dehydratase/L-threonine deaminase OS=Homo sapiens OX=9606 GN=SDS PE=1 SV=2 |
| sp\|P35221\|CTNA1_HUMAN | nucl | Catenin alpha-1 OS=Homo sapiens OX=9606 GN=CTNNA1 PE=1 SV=1 |
| sp\|Q643R3\|LPCT4_HUMAN | plas | Lysophospholipid acyltransferase LPCAT4 OS=Homo sapiens OX=9606 GN=LPCAT4 PE=1 SV=1 |
| sp\|Q9H1C7\|CYTM1_HUMAN | extr | Cysteine-rich and transmembrane domain-containing protein 1 OS=Homo sapiens OX=9606 GN=CYSTM1 PE=1 SV=1 |
| sp\|Q96SB3\|NEB2_HUMAN | nucl | Neurabin-2 OS=Homo sapiens OX=9606 GN=PPP1R9B PE=1 SV=2 |
| sp\|Q5VTQ0\|TT39B_HUMAN | nucl | Tetratricopeptide repeat protein 39B OS=Homo sapiens OX=9606 GN=TTC39B PE=1 SV=4 |
| sp\|Q96BX8\|MOB3A_HUMAN | nucl | MOB kinase activator 3A OS=Homo sapiens OX=9606 GN=MOB3A PE=1 SV=1 |
| sp\|O75882\|ATRN_HUMAN | plas | Attractin OS=Homo sapiens OX=9606 GN=ATRN PE=1 SV=2 |
| sp\|Q7Z4L5\|TT21B_HUMAN | cyto | Tetratricopeptide repeat protein 21B OS=Homo sapiens OX=9606 GN=TTC21B PE=1 SV=2 |
| sp\|Q13188\|STK3_HUMAN | cyto | Serine/threonine-protein kinase 3 OS=Homo sapiens OX=9606 GN=STK3 PE=1 SV=2 |
| sp\|P14902\|I23O1_HUMAN | cyto | Indoleamine 2,3-dioxygenase 1 OS=Homo sapiens OX=9606 GN=IDO1 PE=1 SV=1 |
| sp\|P43487\|RANG_HUMAN | cyto | Ran-specific GTPase-activating protein OS=Homo sapiens OX=9606 GN=RANBP1 PE=1 SV=1 |
| sp\|P35222\|CTNB1_HUMAN | cyto_nucl | Catenin beta-1 OS=Homo sapiens OX=9606 GN=CTNNB1 PE=1 SV=1 |
| sp\|Q9ULE4\|F184B_HUMAN | nucl | Protein FAM184B OS=Homo sapiens OX=9606 GN=FAM184B PE=2 SV=3 |
| sp\|P10636\|TAU_HUMAN | nucl | Microtubule-associated protein tau OS=Homo sapiens OX=9606 GN=MAPT PE=1 SV=5 |
| sp\|P23025\|XPA_HUMAN | nucl | DNA repair protein complementing XP-A cells OS=Homo sapiens OX=9606 GN=XPA PE=1 SV=1 |
| sp\|Q00610\|CLH1_HUMAN | cyto | Clathrin heavy chain 1 OS=Homo sapiens OX=9606 GN=CLTC PE=1 SV=5 |
| sp\|P08047\|SP1_HUMAN | nucl | Transcription factor Sp1 OS=Homo sapiens OX=9606 GN=SP1 PE=1 SV=3 |
| sp\|P51608\|MECP2_HUMAN | nucl | Methyl-CpG-binding protein 2 OS=Homo sapiens OX=9606 GN=MECP2 PE=1 SV=1 |
| sp\|Q9NVJ2\|ARL8B_HUMAN | cyto | ADP-ribosylation factor-like protein 8B OS=Homo sapiens OX=9606 GN=ARL8B PE=1 SV=1 |
| sp\|Q14139\|UBE4A_HUMAN | plas | Ubiquitin conjugation factor E4 A OS=Homo sapiens OX=9606 GN=UBE4A PE=1 SV=2 |
| sp\|P06753\|TPM3_HUMAN | cyto | Tropomyosin alpha-3 chain OS=Homo sapiens OX=9606 GN=TPM3 PE=1 SV=2 |
| sp\|P05026\|AT1B1_HUMAN | plas | Sodium/potassium-transporting ATPase subunit beta-1 OS=Homo sapiens OX=9606 GN=ATP1B1 PE=1 SV=1 |
| sp\|Q9UQN3\|CHM2B_HUMAN | cyto | Charged multivesicular body protein 2b OS=Homo sapiens OX=9606 GN=CHMP2B PE=1 SV=1 |
| sp\|Q99895\|CTRC_HUMAN | extr | Chymotrypsin-C OS=Homo sapiens OX=9606 GN=CTRC PE=1 SV=2 |
| sp\|Q9NSE4\|SYIM_HUMAN | mito | Isoleucine--tRNA ligase, mitochondrial OS=Homo sapiens OX=9606 GN=IARS2 PE=1 SV=2 |
| sp\|Q9Y2W1\|TR150_HUMAN | nucl | Thyroid hormone receptor-associated protein 3 OS=Homo sapiens OX=9606 GN=THRAP3 PE=1 SV=2 |
| sp\|L0R819\|ASURF_HUMAN | nucl | ASNSD1 upstream open reading frame protein OS=Homo sapiens OX=9606 GN=ASDURF PE=1 SV=1 |
| sp\|P09488\|GSTM1_HUMAN | cyto | Glutathione S-transferase Mu 1 OS=Homo sapiens OX=9606 GN=GSTM1 PE=1 SV=3 |
| sp\|P62750\|RL23A_HUMAN | cyto | 60S ribosomal protein L23a OS=Homo sapiens OX=9606 GN=RPL23A PE=1 SV=1 |
| sp\|Q32P28\|P3H1_HUMAN | extr | Prolyl 3-hydroxylase 1 OS=Homo sapiens OX=9606 GN=P3H1 PE=1 SV=2 |
| sp\|Q9H147\|TDIF1_HUMAN | nucl | Deoxynucleotidyltransferase terminal-interacting protein 1 OS=Homo sapiens OX=9606 GN=DNTTIP1 PE=1 SV=2 |
| sp\|P42345\|MTOR_HUMAN | plas | Serine/threonine-protein kinase mTOR OS=Homo sapiens OX=9606 GN=MTOR PE=1 SV=1 |
| sp\|P09471\|GNAO_HUMAN | cyto_mito | Guanine nucleotide-binding protein G(o) subunit alpha OS=Homo sapiens OX=9606 GN=GNAO1 PE=1 SV=4 |
| sp\|P17301\|ITA2_HUMAN | plas | Integrin alpha-2 OS=Homo sapiens OX=9606 GN=ITGA2 PE=1 SV=1 |
| sp\|P12724\|ECP_HUMAN | extr | Eosinophil cationic protein OS=Homo sapiens OX=9606 GN=RNASE3 PE=1 SV=2 |
| sp\|O14682\|ENC1_HUMAN | mito | Ectoderm-neural cortex protein 1 OS=Homo sapiens OX=9606 GN=ENC1 PE=1 SV=2 |
| sp\|Q96H20\|SNF8_HUMAN | cyto | Vacuolar-sorting protein SNF8 OS=Homo sapiens OX=9606 GN=SNF8 PE=1 SV=1 |
| sp\|Q14722\|KCAB1_HUMAN | nucl | Voltage-gated potassium channel subunit beta-1 OS=Homo sapiens OX=9606 GN=KCNAB1 PE=1 SV=1 |
| sp\|P16422\|EPCAM_HUMAN | extr | Epithelial cell adhesion molecule OS=Homo sapiens OX=9606 GN=EPCAM PE=1 SV=2 |
| sp\|O43660\|PLRG1_HUMAN | nucl | Pleiotropic regulator 1 OS=Homo sapiens OX=9606 GN=PLRG1 PE=1 SV=1 |
| sp\|O00629\|IMA3_HUMAN | cyto | Importin subunit alpha-3 OS=Homo sapiens OX=9606 GN=KPNA4 PE=1 SV=1 |
| sp\|P07098\|LIPG_HUMAN | extr | Gastric triacylglycerol lipase OS=Homo sapiens OX=9606 GN=LIPF PE=1 SV=1 |
| sp\|Q13617\|CUL2_HUMAN | cyto | Cullin-2 OS=Homo sapiens OX=9606 GN=CUL2 PE=1 SV=2 |
| sp\|P28330\|ACADL_HUMAN | mito | Long-chain specific acyl-CoA dehydrogenase, mitochondrial OS=Homo sapiens OX=9606 GN=ACADL PE=1 SV=2 |
| sp\|Q6ZS30\|NBEL1_HUMAN | cyto | Neurobeachin-like protein 1 OS=Homo sapiens OX=9606 GN=NBEAL1 PE=2 SV=3 |
| sp\|O75051\|PLXA2_HUMAN | plas | Plexin-A2 OS=Homo sapiens OX=9606 GN=PLXNA2 PE=1 SV=4 |
| sp\|Q5K4L6\|S27A3_HUMAN | E.R. | Solute carrier family 27 member 3 OS=Homo sapiens OX=9606 GN=SLC27A3 PE=2 SV=4 |
| sp\|Q9UPU5\|UBP24_HUMAN | cyto | Ubiquitin carboxyl-terminal hydrolase 24 OS=Homo sapiens OX=9606 GN=USP24 PE=1 SV=3 |
| sp\|P49748\|ACADV_HUMAN | mito | Very long-chain specific acyl-CoA dehydrogenase, mitochondrial OS=Homo sapiens OX=9606 GN=ACADVL PE=1 SV=1 |
| sp\|Q8TAD4\|ZNT5_HUMAN | plas | Zinc transporter 5 OS=Homo sapiens OX=9606 GN=SLC30A5 PE=1 SV=1 |
| sp\|O43556\|SGCE_HUMAN | plas | Epsilon-sarcoglycan OS=Homo sapiens OX=9606 GN=SGCE PE=1 SV=6 |
| sp\|P29373\|RABP2_HUMAN | cyto | Cellular retinoic acid-binding protein 2 OS=Homo sapiens OX=9606 GN=CRABP2 PE=1 SV=2 |
| sp\|Q13123\|RED_HUMAN | nucl | Protein Red OS=Homo sapiens OX=9606 GN=IK PE=1 SV=3 |
| sp\|Q6UXG2\|K1324_HUMAN | nucl | UPF0577 protein KIAA1324 OS=Homo sapiens OX=9606 GN=KIAA1324 PE=2 SV=2 |
| sp\|Q13332\|PTPRS_HUMAN | E.R. | Receptor-type tyrosine-protein phosphatase S OS=Homo sapiens OX=9606 GN=PTPRS PE=1 SV=3 |
| sp\|P41208\|CETN2_HUMAN | nucl | Centrin-2 OS=Homo sapiens OX=9606 GN=CETN2 PE=1 SV=1 |
| sp\|P98194\|AT2C1_HUMAN | plas | Calcium-transporting ATPase type 2C member 1 OS=Homo sapiens OX=9606 GN=ATP2C1 PE=1 SV=3 |
| sp\|Q9GZQ3\|COMD5_HUMAN | cyto_nucl | COMM domain-containing protein 5 OS=Homo sapiens OX=9606 GN=COMMD5 PE=1 SV=1 |
| sp\|P67936\|TPM4_HUMAN | cyto | Tropomyosin alpha-4 chain OS=Homo sapiens OX=9606 GN=TPM4 PE=1 SV=3 |
| sp\|Q13496\|MTM1_HUMAN | cyto | Myotubularin OS=Homo sapiens OX=9606 GN=MTM1 PE=1 SV=2 |
| sp\|Q9UBY8\|CLN8_HUMAN | plas | Protein CLN8 OS=Homo sapiens OX=9606 GN=CLN8 PE=1 SV=3 |
| sp\|P02786\|TFR1_HUMAN | plas | Transferrin receptor protein 1 OS=Homo sapiens OX=9606 GN=TFRC PE=1 SV=2 |
| sp\|Q8IUR0\|TPPC5_HUMAN | mito | Trafficking protein particle complex subunit 5 OS=Homo sapiens OX=9606 GN=TRAPPC5 PE=1 SV=1 |
| sp\|Q5T4S7\|UBR4_HUMAN | plas | E3 ubiquitin-protein ligase UBR4 OS=Homo sapiens OX=9606 GN=UBR4 PE=1 SV=1 |
| sp\|P02679\|FIBG_HUMAN | mito | Fibrinogen gamma chain OS=Homo sapiens OX=9606 GN=FGG PE=1 SV=3 |
| sp\|Q9NVA2\|SEP11_HUMAN | nucl | Septin-11 OS=Homo sapiens OX=9606 GN=SEPT11 PE=1 SV=3 |
| sp\|P80723\|BASP1_HUMAN | nucl | Brain acid soluble protein 1 OS=Homo sapiens OX=9606 GN=BASP1 PE=1 SV=2 |
| sp\|Q8N3Y7\|RDHE2_HUMAN | extr | Epidermal retinol dehydrogenase 2 OS=Homo sapiens OX=9606 GN=SDR16C5 PE=2 SV=2 |
| sp\|P35613\|BASI_HUMAN | E.R. | Basigin OS=Homo sapiens OX=9606 GN=BSG PE=1 SV=2 |
| sp\|O43264\|ZW10_HUMAN | cyto | Centromere/kinetochore protein zw10 homolog OS=Homo sapiens OX=9606 GN=ZW10 PE=1 SV=3 |
| sp\|Q6P1R4\|DUS1L_HUMAN | cyto | tRNA-dihydrouridine(16/17) synthase [NAD(P)(+)]-like OS=Homo sapiens OX=9606 GN=DUS1L PE=1 SV=1 |
| sp\|Q9HD89\|RETN_HUMAN | extr | Resistin OS=Homo sapiens OX=9606 GN=RETN PE=1 SV=1 |
| sp\|P43652\|AFAM_HUMAN | extr | Afamin OS=Homo sapiens OX=9606 GN=AFM PE=1 SV=1 |
| sp\|Q9BYD6\|RM01_HUMAN | mito | 39S ribosomal protein L1, mitochondrial OS=Homo sapiens OX=9606 GN=MRPL1 PE=1 SV=2 |
| sp\|Q86W25\|NAL13_HUMAN | cyto | NACHT, LRR and PYD domains-containing protein 13 OS=Homo sapiens OX=9606 GN=NLRP13 PE=2 SV=2 |
| sp\|Q8NC44\|RETR2_HUMAN | plas | Reticulophagy regulator 2 OS=Homo sapiens OX=9606 GN=RETREG2 PE=1 SV=3 |
| sp\|Q15286\|RAB35_HUMAN | cyto | Ras-related protein Rab-35 OS=Homo sapiens OX=9606 GN=RAB35 PE=1 SV=1 |
| sp\|P08962\|CD63_HUMAN | plas | CD63 antigen OS=Homo sapiens OX=9606 GN=CD63 PE=1 SV=2 |
| sp\|O95622\|ADCY5_HUMAN | plas | Adenylate cyclase type 5 OS=Homo sapiens OX=9606 GN=ADCY5 PE=1 SV=3 |
| sp\|P16401\|H15_HUMAN | nucl | Histone H1.5 OS=Homo sapiens OX=9606 GN=HIST1H1B PE=1 SV=3 |
| sp\|Q08495\|DEMA_HUMAN | nucl | Dematin OS=Homo sapiens OX=9606 GN=DMTN PE=1 SV=3 |
| sp\|A0A075B6J9\|LV218_HUMAN | extr | Immunoglobulin lambda variable 2-18 OS=Homo sapiens OX=9606 GN=IGLV2-18 PE=3 SV=2 |
| sp\|O14602\|IF1AY_HUMAN | cyto_nucl | Eukaryotic translation initiation factor 1A, Y-chromosomal OS=Homo sapiens OX=9606 GN=EIF1AY PE=1 SV=4 |
| sp\|P20774\|MIME_HUMAN | extr | Mimecan OS=Homo sapiens OX=9606 GN=OGN PE=1 SV=1 |
| sp\|P13645\|K1C10_HUMAN | nucl | Keratin, type I cytoskeletal 10 OS=Homo sapiens OX=9606 GN=KRT10 PE=1 SV=6 |
| sp\|P36222\|CH3L1_HUMAN | extr | Chitinase-3-like protein 1 OS=Homo sapiens OX=9606 GN=CHI3L1 PE=1 SV=2 |
| sp\|Q9H9S4\|CB39L_HUMAN | cyto | Calcium-binding protein 39-like OS=Homo sapiens OX=9606 GN=CAB39L PE=1 SV=3 |
| sp\|Q8IXK0\|PHC2_HUMAN | nucl | Polyhomeotic-like protein 2 OS=Homo sapiens OX=9606 GN=PHC2 PE=1 SV=1 |
| sp\|P62837\|UB2D2_HUMAN | cyto | Ubiquitin-conjugating enzyme E2 D2 OS=Homo sapiens OX=9606 GN=UBE2D2 PE=1 SV=1 |
| sp\|Q5QGZ9\|CL12A_HUMAN | cyto | C-type lectin domain family 12 member A OS=Homo sapiens OX=9606 GN=CLEC12A PE=1 SV=3 |
| sp\|O75475\|PSIP1_HUMAN | nucl | PC4 and SFRS1-interacting protein OS=Homo sapiens OX=9606 GN=PSIP1 PE=1 SV=1 |
| sp\|Q9UBF2\|COPG2_HUMAN | cyto | Coatomer subunit gamma-2 OS=Homo sapiens OX=9606 GN=COPG2 PE=1 SV=1 |
| sp\|O43414\|ERI3_HUMAN | cyto | ERI1 exoribonuclease 3 OS=Homo sapiens OX=9606 GN=ERI3 PE=1 SV=2 |
| sp\|P25092\|GUC2C_HUMAN | pero | Heat-stable enterotoxin receptor OS=Homo sapiens OX=9606 GN=GUCY2C PE=1 SV=2 |
| sp\|O14735\|CDIPT_HUMAN | plas | CDP-diacylglycerol--inositol 3-phosphatidyltransferase OS=Homo sapiens OX=9606 GN=CDIPT PE=1 SV=1 |
| sp\|P34982\|OR1D2_HUMAN | plas | Olfactory receptor 1D2 OS=Homo sapiens OX=9606 GN=OR1D2 PE=1 SV=2 |
| sp\|Q8N2G8\|GHDC_HUMAN | extr | GH3 domain-containing protein OS=Homo sapiens OX=9606 GN=GHDC PE=1 SV=2 |
| sp\|Q96E52\|OMA1_HUMAN | plas | Metalloendopeptidase OMA1, mitochondrial OS=Homo sapiens OX=9606 GN=OMA1 PE=1 SV=1 |
| sp\|O75339\|CILP1_HUMAN | extr | Cartilage intermediate layer protein 1 OS=Homo sapiens OX=9606 GN=CILP PE=1 SV=4 |
| sp\|Q8IZR5\|CKLF4_HUMAN | plas | CKLF-like MARVEL transmembrane domain-containing protein 4 OS=Homo sapiens OX=9606 GN=CMTM4 PE=1 SV=1 |
| sp\|Q9UPR3\|SMG5_HUMAN | cyto | Protein SMG5 OS=Homo sapiens OX=9606 GN=SMG5 PE=1 SV=3 |
| sp\|O75348\|VATG1_HUMAN | nucl | V-type proton ATPase subunit G 1 OS=Homo sapiens OX=9606 GN=ATP6V1G1 PE=1 SV=3 |
| sp\|P08236\|BGLR_HUMAN | nucl | Beta-glucuronidase OS=Homo sapiens OX=9606 GN=GUSB PE=1 SV=2 |
| sp\|O95684\|FR1OP_HUMAN | cyto | FGFR1 oncogene partner OS=Homo sapiens OX=9606 GN=FGFR1OP PE=1 SV=1 |
| sp\|P13647\|K2C5_HUMAN | nucl | Keratin, type II cytoskeletal 5 OS=Homo sapiens OX=9606 GN=KRT5 PE=1 SV=3 |
| sp\|Q8TB96\|TIP_HUMAN | plas | T-cell immunomodulatory protein OS=Homo sapiens OX=9606 GN=ITFG1 PE=1 SV=1 |
| sp\|P08910\|ABHD2_HUMAN | extr | Monoacylglycerol lipase ABHD2 OS=Homo sapiens OX=9606 GN=ABHD2 PE=1 SV=1 |
| sp\|Q9NQS1\|AVEN_HUMAN | nucl | Cell death regulator Aven OS=Homo sapiens OX=9606 GN=AVEN PE=1 SV=1 |
| sp\|Q7Z304\|MAMC2_HUMAN | extr | MAM domain-containing protein 2 OS=Homo sapiens OX=9606 GN=MAMDC2 PE=2 SV=3 |
| sp\|Q9Y399\|RT02_HUMAN | mito | 28S ribosomal protein S2, mitochondrial OS=Homo sapiens OX=9606 GN=MRPS2 PE=1 SV=1 |
| sp\|P25024\|CXCR1_HUMAN | plas | C-X-C chemokine receptor type 1 OS=Homo sapiens OX=9606 GN=CXCR1 PE=1 SV=2 |
| sp\|P61026\|RAB10_HUMAN | cyto | Ras-related protein Rab-10 OS=Homo sapiens OX=9606 GN=RAB10 PE=1 SV=1 |
| sp\|Q16643\|DREB_HUMAN | nucl | Drebrin OS=Homo sapiens OX=9606 GN=DBN1 PE=1 SV=4 |
| sp\|Q8N1S5\|S39AB_HUMAN | plas | Zinc transporter ZIP11 OS=Homo sapiens OX=9606 GN=SLC39A11 PE=2 SV=3 |
| sp\|P54687\|BCAT1_HUMAN | cyto | Branched-chain-amino-acid aminotransferase, cytosolic OS=Homo sapiens OX=9606 GN=BCAT1 PE=1 SV=3 |
| sp\|P24592\|IBP6_HUMAN | extr | Insulin-like growth factor-binding protein 6 OS=Homo sapiens OX=9606 GN=IGFBP6 PE=1 SV=1 |
| sp\|Q9NQ48\|LZTL1_HUMAN | cyto | Leucine zipper transcription factor-like protein 1 OS=Homo sapiens OX=9606 GN=LZTFL1 PE=1 SV=1 |
| sp\|P01709\|LV208_HUMAN | extr | Immunoglobulin lambda variable 2-8 OS=Homo sapiens OX=9606 GN=IGLV2-8 PE=1 SV=2 |
| sp\|Q99698\|LYST_HUMAN | plas | Lysosomal-trafficking regulator OS=Homo sapiens OX=9606 GN=LYST PE=1 SV=3 |
| sp\|Q96AX2\|RAB37_HUMAN | cyto | Ras-related protein Rab-37 OS=Homo sapiens OX=9606 GN=RAB37 PE=1 SV=3 |
| sp\|Q5T3F8\|CSCL2_HUMAN | plas | CSC1-like protein 2 OS=Homo sapiens OX=9606 GN=TMEM63B PE=1 SV=1 |
| sp\|Q15063\|POSTN_HUMAN | E.R. | Periostin OS=Homo sapiens OX=9606 GN=POSTN PE=1 SV=2 |
| sp\|P62253\|UB2G1_HUMAN | cyto | Ubiquitin-conjugating enzyme E2 G1 OS=Homo sapiens OX=9606 GN=UBE2G1 PE=1 SV=3 |
| sp\|Q86UD3\|MARH3_HUMAN | plas | E3 ubiquitin-protein ligase MARCH3 OS=Homo sapiens OX=9606 GN=MARCH3 PE=1 SV=1 |
| sp\|P26641\|EF1G_HUMAN | cyto | Elongation factor 1-gamma OS=Homo sapiens OX=9606 GN=EEF1G PE=1 SV=3 |
| sp\|O14727\|APAF_HUMAN | cyto | Apoptotic protease-activating factor 1 OS=Homo sapiens OX=9606 GN=APAF1 PE=1 SV=2 |
| sp\|Q15283\|RASA2_HUMAN | cyto | Ras GTPase-activating protein 2 OS=Homo sapiens OX=9606 GN=RASA2 PE=1 SV=3 |
| sp\|Q9H492\|MLP3A_HUMAN | nucl | Microtubule-associated proteins 1A/1B light chain 3A OS=Homo sapiens OX=9606 GN=MAP1LC3A PE=1 SV=2 |
| sp\|O60687\|SRPX2_HUMAN | extr | Sushi repeat-containing protein SRPX2 OS=Homo sapiens OX=9606 GN=SRPX2 PE=1 SV=1 |
| sp\|P26583\|HMGB2_HUMAN | nucl | High mobility group protein B2 OS=Homo sapiens OX=9606 GN=HMGB2 PE=1 SV=2 |
| sp\|Q9BW62\|KATL1_HUMAN | cyto | Katanin p60 ATPase-containing subunit A-like 1 OS=Homo sapiens OX=9606 GN=KATNAL1 PE=1 SV=1 |
| sp\|P53992\|SC24C_HUMAN | nucl | Protein transport protein Sec24C OS=Homo sapiens OX=9606 GN=SEC24C PE=1 SV=3 |
| sp\|Q99961\|SH3G1_HUMAN | nucl | Endophilin-A2 OS=Homo sapiens OX=9606 GN=SH3GL1 PE=1 SV=1 |
| sp\|O75355\|ENTP3_HUMAN | plas | Ectonucleoside triphosphate diphosphohydrolase 3 OS=Homo sapiens OX=9606 GN=ENTPD3 PE=1 SV=2 |
| sp\|Q9UL18\|AGO1_HUMAN | cyto | Protein argonaute-1 OS=Homo sapiens OX=9606 GN=AGO1 PE=1 SV=3 |
| sp\|O95822\|DCMC_HUMAN | mito | Malonyl-CoA decarboxylase, mitochondrial OS=Homo sapiens OX=9606 GN=MLYCD PE=1 SV=3 |
| sp\|Q96A65\|EXOC4_HUMAN | nucl | Exocyst complex component 4 OS=Homo sapiens OX=9606 GN=EXOC4 PE=1 SV=1 |
| sp\|Q9NRG9\|AAAS_HUMAN | cyto | Aladin OS=Homo sapiens OX=9606 GN=AAAS PE=1 SV=1 |
| sp\|A0A075B6I1\|LV460_HUMAN | extr | Immunoglobulin lambda variable 4-60 OS=Homo sapiens OX=9606 GN=IGLV4-60 PE=3 SV=1 |
| sp\|Q8NAV1\|PR38A_HUMAN | nucl | Pre-mRNA-splicing factor 38A OS=Homo sapiens OX=9606 GN=PRPF38A PE=1 SV=1 |
| sp\|P04264\|K2C1_HUMAN | nucl | Keratin, type II cytoskeletal 1 OS=Homo sapiens OX=9606 GN=KRT1 PE=1 SV=6 |
| sp\|Q8TD55\|PKHO2_HUMAN | nucl | Pleckstrin homology domain-containing family O member 2 OS=Homo sapiens OX=9606 GN=PLEKHO2 PE=1 SV=1 |
| sp\|P08134\|RHOC_HUMAN | cyto | Rho-related GTP-binding protein RhoC OS=Homo sapiens OX=9606 GN=RHOC PE=1 SV=1 |
| sp\|O15270\|SPTC2_HUMAN | cyto | Serine palmitoyltransferase 2 OS=Homo sapiens OX=9606 GN=SPTLC2 PE=1 SV=1 |
| sp\|Q6ZRP7\|QSOX2_HUMAN | plas | Sulfhydryl oxidase 2 OS=Homo sapiens OX=9606 GN=QSOX2 PE=1 SV=3 |
| sp\|Q14117\|DPYS_HUMAN | cyto | Dihydropyrimidinase OS=Homo sapiens OX=9606 GN=DPYS PE=1 SV=1 |
| sp\|Q7Z4N2\|TRPM1_HUMAN | plas | Transient receptor potential cation channel subfamily M member 1 OS=Homo sapiens OX=9606 GN=TRPM1 PE=1 SV=2 |
| sp\|P31513\|FMO3_HUMAN | plas | Dimethylaniline monooxygenase [N-oxide-forming] 3 OS=Homo sapiens OX=9606 GN=FMO3 PE=1 SV=5 |
| sp\|Q9Y376\|CAB39_HUMAN | cyto | Calcium-binding protein 39 OS=Homo sapiens OX=9606 GN=CAB39 PE=1 SV=1 |
| sp\|Q16836\|HCDH_HUMAN | mito | Hydroxyacyl-coenzyme A dehydrogenase, mitochondrial OS=Homo sapiens OX=9606 GN=HADH PE=1 SV=3 |
| sp\|Q9BXL7\|CAR11_HUMAN | nucl | Caspase recruitment domain-containing protein 11 OS=Homo sapiens OX=9606 GN=CARD11 PE=1 SV=3 |
| sp\|P61981\|1433G_HUMAN | cyto | 14-3-3 protein gamma OS=Homo sapiens OX=9606 GN=YWHAG PE=1 SV=2 |
| sp\|O95218\|ZRAB2_HUMAN | nucl | Zinc finger Ran-binding domain-containing protein 2 OS=Homo sapiens OX=9606 GN=ZRANB2 PE=1 SV=2 |
| sp\|P11166\|GTR1_HUMAN | plas | Solute carrier family 2, facilitated glucose transporter member 1 OS=Homo sapiens OX=9606 GN=SLC2A1 PE=1 SV=2 |
| sp\|Q9NZJ5\|E2AK3_HUMAN | plas | Eukaryotic translation initiation factor 2-alpha kinase 3 OS=Homo sapiens OX=9606 GN=EIF2AK3 PE=1 SV=3 |
| sp\|P46821\|MAP1B_HUMAN | nucl | Microtubule-associated protein 1B OS=Homo sapiens OX=9606 GN=MAP1B PE=1 SV=2 |
| sp\|P17858\|PFKAL_HUMAN | cyto | ATP-dependent 6-phosphofructokinase, liver type OS=Homo sapiens OX=9606 GN=PFKL PE=1 SV=6 |
| sp\|Q86Y39\|NDUAB_HUMAN | mito | NADH dehydrogenase [ubiquinone] 1 alpha subcomplex subunit 11 OS=Homo sapiens OX=9606 GN=NDUFA11 PE=1 SV=3 |
| sp\|P01706\|LV211_HUMAN | extr | Immunoglobulin lambda variable 2-11 OS=Homo sapiens OX=9606 GN=IGLV2-11 PE=1 SV=2 |
| sp\|P18124\|RL7_HUMAN | cyto | 60S ribosomal protein L7 OS=Homo sapiens OX=9606 GN=RPL7 PE=1 SV=1 |
| sp\|Q6P996\|PDXD1_HUMAN | cyto | Pyridoxal-dependent decarboxylase domain-containing protein 1 OS=Homo sapiens OX=9606 GN=PDXDC1 PE=1 SV=2 |
| sp\|P12931\|SRC_HUMAN | cyto | Proto-oncogene tyrosine-protein kinase Src OS=Homo sapiens OX=9606 GN=SRC PE=1 SV=3 |
| sp\|P01700\|LV147_HUMAN | extr | Immunoglobulin lambda variable 1-47 OS=Homo sapiens OX=9606 GN=IGLV1-47 PE=1 SV=2 |
| sp\|P07333\|CSF1R_HUMAN | extr | Macrophage colony-stimulating factor 1 receptor OS=Homo sapiens OX=9606 GN=CSF1R PE=1 SV=2 |
| sp\|Q495B1\|AKD1A_HUMAN | cyto | Ankyrin repeat and death domain-containing protein 1A OS=Homo sapiens OX=9606 GN=ANKDD1A PE=2 SV=2 |
| sp\|P10319\|1B58_HUMAN | extr | HLA class I histocompatibility antigen, B-58 alpha chain OS=Homo sapiens OX=9606 GN=HLA-B PE=1 SV=1 |
| sp\|P01011\|AACT_HUMAN | extr | Alpha-1-antichymotrypsin OS=Homo sapiens OX=9606 GN=SERPINA3 PE=1 SV=2 |
| sp\|Q9BYX2\|TBD2A_HUMAN | cyto | TBC1 domain family member 2A OS=Homo sapiens OX=9606 GN=TBC1D2 PE=1 SV=3 |
| sp\|P11766\|ADHX_HUMAN | cyto | Alcohol dehydrogenase class-3 OS=Homo sapiens OX=9606 GN=ADH5 PE=1 SV=4 |
| sp\|P51798\|CLCN7_HUMAN | plas | H(+)/Cl(-) exchange transporter 7 OS=Homo sapiens OX=9606 GN=CLCN7 PE=1 SV=2 |
| sp\|Q8NFT2\|STEA2_HUMAN | plas | Metalloreductase STEAP2 OS=Homo sapiens OX=9606 GN=STEAP2 PE=1 SV=3 |
| sp\|P55011\|S12A2_HUMAN | plas | Solute carrier family 12 member 2 OS=Homo sapiens OX=9606 GN=SLC12A2 PE=1 SV=1 |
| sp\|A6NMZ7\|CO6A6_HUMAN | E.R. | Collagen alpha-6(VI) chain OS=Homo sapiens OX=9606 GN=COL6A6 PE=1 SV=2 |
| sp\|Q96MH6\|TMM68_HUMAN | E.R. | Transmembrane protein 68 OS=Homo sapiens OX=9606 GN=TMEM68 PE=2 SV=2 |
| sp\|O95926\|SYF2_HUMAN | nucl | Pre-mRNA-splicing factor SYF2 OS=Homo sapiens OX=9606 GN=SYF2 PE=1 SV=1 |
| sp\|O75607\|NPM3_HUMAN | extr | Nucleoplasmin-3 OS=Homo sapiens OX=9606 GN=NPM3 PE=1 SV=3 |
| sp\|O15061\|SYNEM_HUMAN | nucl | Synemin OS=Homo sapiens OX=9606 GN=SYNM PE=1 SV=2 |
| sp\|P02452\|CO1A1_HUMAN | extr | Collagen alpha-1(I) chain OS=Homo sapiens OX=9606 GN=COL1A1 PE=1 SV=5 |
| sp\|Q9NUP1\|BL1S4_HUMAN | nucl | Biogenesis of lysosome-related organelles complex 1 subunit 4 OS=Homo sapiens OX=9606 GN=BLOC1S4 PE=1 SV=1 |
| sp\|Q9UK99\|FBX3_HUMAN | cyto | F-box only protein 3 OS=Homo sapiens OX=9606 GN=FBXO3 PE=1 SV=3 |
| sp\|Q9Y4D8\|HECD4_HUMAN | plas | Probable E3 ubiquitin-protein ligase HECTD4 OS=Homo sapiens OX=9606 GN=HECTD4 PE=1 SV=5 |
| sp\|Q93099\|HGD_HUMAN | cyto | Homogentisate 1,2-dioxygenase OS=Homo sapiens OX=9606 GN=HGD PE=1 SV=2 |
| sp\|O14965\|AURKA_HUMAN | cyto | Aurora kinase A OS=Homo sapiens OX=9606 GN=AURKA PE=1 SV=2 |
| sp\|O75363\|BCAS1_HUMAN | cyto | Breast carcinoma-amplified sequence 1 OS=Homo sapiens OX=9606 GN=BCAS1 PE=1 SV=2 |
| sp\|O60232\|SSA27_HUMAN | extr | Sjoegren syndrome/scleroderma autoantigen 1 OS=Homo sapiens OX=9606 GN=SSSCA1 PE=1 SV=1 |
| sp\|P05534\|1A24_HUMAN | extr | HLA class I histocompatibility antigen, A-24 alpha chain OS=Homo sapiens OX=9606 GN=HLA-A PE=1 SV=2 |
| sp\|Q8IUX7\|AEBP1_HUMAN | E.R. | Adipocyte enhancer-binding protein 1 OS=Homo sapiens OX=9606 GN=AEBP1 PE=1 SV=1 |
| sp\|P18085\|ARF4_HUMAN | cyto | ADP-ribosylation factor 4 OS=Homo sapiens OX=9606 GN=ARF4 PE=1 SV=3 |
| sp\|Q9P270\|SLAI2_HUMAN | nucl | SLAIN motif-containing protein 2 OS=Homo sapiens OX=9606 GN=SLAIN2 PE=1 SV=2 |
| sp\|P07478\|TRY2_HUMAN | extr | Trypsin-2 OS=Homo sapiens OX=9606 GN=PRSS2 PE=1 SV=1 |
| sp\|O15232\|MATN3_HUMAN | extr | Matrilin-3 OS=Homo sapiens OX=9606 GN=MATN3 PE=1 SV=2 |
| sp\|Q13087\|PDIA2_HUMAN | extr | Protein disulfide-isomerase A2 OS=Homo sapiens OX=9606 GN=PDIA2 PE=1 SV=2 |
| sp\|Q9NP80\|PLPL8_HUMAN | mito | Calcium-independent phospholipase A2-gamma OS=Homo sapiens OX=9606 GN=PNPLA8 PE=1 SV=1 |
| sp\|P41091\|IF2G_HUMAN | cyto | Eukaryotic translation initiation factor 2 subunit 3 OS=Homo sapiens OX=9606 GN=EIF2S3 PE=1 SV=3 |
| sp\|Q9NRK6\|ABCBA_HUMAN | plas | ATP-binding cassette sub-family B member 10, mitochondrial OS=Homo sapiens OX=9606 GN=ABCB10 PE=1 SV=2 |
| sp\|Q8TF76\|HASP_HUMAN | plas | Serine/threonine-protein kinase haspin OS=Homo sapiens OX=9606 GN=HASPIN PE=1 SV=3 |
| sp\|Q9NRP0\|OSTC_HUMAN | plas | Oligosaccharyltransferase complex subunit OSTC OS=Homo sapiens OX=9606 GN=OSTC PE=1 SV=1 |
| sp\|Q9NRZ7\|PLCC_HUMAN | extr | 1-acyl-sn-glycerol-3-phosphate acyltransferase gamma OS=Homo sapiens OX=9606 GN=AGPAT3 PE=1 SV=1 |
| sp\|P01701\|LV151_HUMAN | extr | Immunoglobulin lambda variable 1-51 OS=Homo sapiens OX=9606 GN=IGLV1-51 PE=1 SV=2 |
| sp\|Q5SWX8\|ODR4_HUMAN | plas | Protein odr-4 homolog OS=Homo sapiens OX=9606 GN=ODR4 PE=1 SV=1 |
| sp\|I1YAP6\|TRI77_HUMAN | nucl | Tripartite motif-containing protein 77 OS=Homo sapiens OX=9606 GN=TRIM77 PE=2 SV=2 |
| sp\|P49207\|RL34_HUMAN | nucl | 60S ribosomal protein L34 OS=Homo sapiens OX=9606 GN=RPL34 PE=1 SV=3 |
| sp\|P16989\|YBOX3_HUMAN | nucl | Y-box-binding protein 3 OS=Homo sapiens OX=9606 GN=YBX3 PE=1 SV=4 |
| sp\|Q15120\|PDK3_HUMAN | mito | [Pyruvate dehydrogenase (acetyl-transferring)] kinase isozyme 3, mitochondrial OS=Homo sapiens OX=9606 GN=PDK3 PE=1 SV=1 |
| sp\|P61163\|ACTZ_HUMAN | cysk | Alpha-centractin OS=Homo sapiens OX=9606 GN=ACTR1A PE=1 SV=1 |
| sp\|P52790\|HXK3_HUMAN | cyto | Hexokinase-3 OS=Homo sapiens OX=9606 GN=HK3 PE=1 SV=2 |
| sp\|P11216\|PYGB_HUMAN | cyto | Glycogen phosphorylase, brain form OS=Homo sapiens OX=9606 GN=PYGB PE=1 SV=5 |
| sp\|Q7Z404\|TMC4_HUMAN | plas | Transmembrane channel-like protein 4 OS=Homo sapiens OX=9606 GN=TMC4 PE=2 SV=3 |
| sp\|P12236\|ADT3_HUMAN | cyto | ADP/ATP translocase 3 OS=Homo sapiens OX=9606 GN=SLC25A6 PE=1 SV=4 |
| sp\|P78537\|BL1S1_HUMAN | mito | Biogenesis of lysosome-related organelles complex 1 subunit 1 OS=Homo sapiens OX=9606 GN=BLOC1S1 PE=1 SV=2 |
| sp\|Q92973\|TNPO1_HUMAN | cyto | Transportin-1 OS=Homo sapiens OX=9606 GN=TNPO1 PE=1 SV=2 |
| sp\|A0A0C4DH68\|KV224_HUMAN | extr | Immunoglobulin kappa variable 2-24 OS=Homo sapiens OX=9606 GN=IGKV2-24 PE=3 SV=1 |
| sp\|Q6P1A2\|MBOA5_HUMAN | plas | Lysophospholipid acyltransferase 5 OS=Homo sapiens OX=9606 GN=LPCAT3 PE=1 SV=1 |
| sp\|P50454\|SERPH_HUMAN | extr | Serpin H1 OS=Homo sapiens OX=9606 GN=SERPINH1 PE=1 SV=2 |
| sp\|Q53GQ0\|DHB12_HUMAN | cyto | Very-long-chain 3-oxoacyl-CoA reductase OS=Homo sapiens OX=9606 GN=HSD17B12 PE=1 SV=2 |
| sp\|Q15847\|ADIRF_HUMAN | cyto | Adipogenesis regulatory factor OS=Homo sapiens OX=9606 GN=ADIRF PE=1 SV=1 |
| sp\|Q9H4A6\|GOLP3_HUMAN | mito | Golgi phosphoprotein 3 OS=Homo sapiens OX=9606 GN=GOLPH3 PE=1 SV=1 |
| sp\|Q86TM6\|SYVN1_HUMAN | plas | E3 ubiquitin-protein ligase synoviolin OS=Homo sapiens OX=9606 GN=SYVN1 PE=1 SV=2 |
| sp\|Q8IVL6\|P3H3_HUMAN | extr | Prolyl 3-hydroxylase 3 OS=Homo sapiens OX=9606 GN=P3H3 PE=1 SV=1 |
| sp\|O00116\|ADAS_HUMAN | cyto | Alkyldihydroxyacetonephosphate synthase, peroxisomal OS=Homo sapiens OX=9606 GN=AGPS PE=1 SV=1 |
| sp\|P00813\|ADA_HUMAN | cyto | Adenosine deaminase OS=Homo sapiens OX=9606 GN=ADA PE=1 SV=3 |
| sp\|Q15784\|NDF2_HUMAN | nucl | Neurogenic differentiation factor 2 OS=Homo sapiens OX=9606 GN=NEUROD2 PE=2 SV=2 |
| sp\|Q96I15\|SCLY_HUMAN | cyto | Selenocysteine lyase OS=Homo sapiens OX=9606 GN=SCLY PE=1 SV=4 |
| sp\|Q9H1P3\|OSBL2_HUMAN | nucl | Oxysterol-binding protein-related protein 2 OS=Homo sapiens OX=9606 GN=OSBPL2 PE=1 SV=1 |
| sp\|Q99541\|PLIN2_HUMAN | mito | Perilipin-2 OS=Homo sapiens OX=9606 GN=PLIN2 PE=1 SV=2 |
| sp\|Q14165\|MLEC_HUMAN | plas | Malectin OS=Homo sapiens OX=9606 GN=MLEC PE=1 SV=1 |
| sp\|Q9UHB7\|AFF4_HUMAN | nucl | AF4/FMR2 family member 4 OS=Homo sapiens OX=9606 GN=AFF4 PE=1 SV=1 |
| sp\|Q9BYV8\|CEP41_HUMAN | nucl | Centrosomal protein of 41 kDa OS=Homo sapiens OX=9606 GN=CEP41 PE=1 SV=1 |
| sp\|Q5JS54\|PSMG4_HUMAN | nucl | Proteasome assembly chaperone 4 OS=Homo sapiens OX=9606 GN=PSMG4 PE=1 SV=2 |
| sp\|Q07507\|DERM_HUMAN | extr | Dermatopontin OS=Homo sapiens OX=9606 GN=DPT PE=1 SV=2 |
| sp\|Q14005\|IL16_HUMAN | nucl | Pro-interleukin-16 OS=Homo sapiens OX=9606 GN=IL16 PE=1 SV=4 |
| sp\|Q9UID3\|VPS51_HUMAN | nucl | Vacuolar protein sorting-associated protein 51 homolog OS=Homo sapiens OX=9606 GN=VPS51 PE=1 SV=2 |
| sp\|Q7Z3C6\|ATG9A_HUMAN | plas | Autophagy-related protein 9A OS=Homo sapiens OX=9606 GN=ATG9A PE=1 SV=3 |
| sp\|Q8TDR0\|MIPT3_HUMAN | nucl | TRAF3-interacting protein 1 OS=Homo sapiens OX=9606 GN=TRAF3IP1 PE=1 SV=1 |
| sp\|O14672\|ADA10_HUMAN | plas | Disintegrin and metalloproteinase domain-containing protein 10 OS=Homo sapiens OX=9606 GN=ADAM10 PE=1 SV=1 |
| sp\|Q8WVJ2\|NUDC2_HUMAN | cyto | NudC domain-containing protein 2 OS=Homo sapiens OX=9606 GN=NUDCD2 PE=1 SV=1 |
| sp\|O75787\|RENR_HUMAN | E.R. | Renin receptor OS=Homo sapiens OX=9606 GN=ATP6AP2 PE=1 SV=2 |
| sp\|P14324\|FPPS_HUMAN | mito | Farnesyl pyrophosphate synthase OS=Homo sapiens OX=9606 GN=FDPS PE=1 SV=4 |
| sp\|Q8IXM2\|BAP18_HUMAN | nucl | Chromatin complexes subunit BAP18 OS=Homo sapiens OX=9606 GN=BAP18 PE=1 SV=1 |
| sp\|P39900\|MMP12_HUMAN | extr | Macrophage metalloelastase OS=Homo sapiens OX=9606 GN=MMP12 PE=1 SV=1 |
| sp\|Q15124\|PGM5_HUMAN | cysk | Phosphoglucomutase-like protein 5 OS=Homo sapiens OX=9606 GN=PGM5 PE=1 SV=2 |
| sp\|Q96H55\|MYO19_HUMAN | nucl | Unconventional myosin-XIX OS=Homo sapiens OX=9606 GN=MYO19 PE=1 SV=2 |
| sp\|P41252\|SYIC_HUMAN | cyto | Isoleucine--tRNA ligase, cytoplasmic OS=Homo sapiens OX=9606 GN=IARS PE=1 SV=2 |
| sp\|Q9HBY0\|NOX3_HUMAN | plas | NADPH oxidase 3 OS=Homo sapiens OX=9606 GN=NOX3 PE=1 SV=1 |
| sp\|P19075\|TSN8_HUMAN | plas | Tetraspanin-8 OS=Homo sapiens OX=9606 GN=TSPAN8 PE=1 SV=1 |
| sp\|Q7Z392\|TPC11_HUMAN | cyto | Trafficking protein particle complex subunit 11 OS=Homo sapiens OX=9606 GN=TRAPPC11 PE=1 SV=2 |
| sp\|P60953\|CDC42_HUMAN | cyto | Cell division control protein 42 homolog OS=Homo sapiens OX=9606 GN=CDC42 PE=1 SV=2 |
| sp\|P30520\|PURA2_HUMAN | cyto | Adenylosuccinate synthetase isozyme 2 OS=Homo sapiens OX=9606 GN=ADSS PE=1 SV=3 |
| sp\|P04433\|KV311_HUMAN | extr | Immunoglobulin kappa variable 3-11 OS=Homo sapiens OX=9606 GN=IGKV3-11 PE=1 SV=1 |
| sp\|Q8IXH7\|NELFD_HUMAN | cyto | Negative elongation factor C/D OS=Homo sapiens OX=9606 GN=NELFCD PE=1 SV=2 |
| sp\|Q9NZ63\|TLS1_HUMAN | nucl | Telomere length and silencing protein 1 homolog OS=Homo sapiens OX=9606 GN=C9orf78 PE=1 SV=1 |
| sp\|Q96K49\|TM87B_HUMAN | plas | Transmembrane protein 87B OS=Homo sapiens OX=9606 GN=TMEM87B PE=1 SV=1 |
| sp\|Q9H0R3\|TM222_HUMAN | cyto | Transmembrane protein 222 OS=Homo sapiens OX=9606 GN=TMEM222 PE=1 SV=2 |
| sp\|Q9Y6A9\|SPCS1_HUMAN | plas | Signal peptidase complex subunit 1 OS=Homo sapiens OX=9606 GN=SPCS1 PE=1 SV=4 |
| sp\|Q3KQU3\|MA7D1_HUMAN | nucl | MAP7 domain-containing protein 1 OS=Homo sapiens OX=9606 GN=MAP7D1 PE=1 SV=1 |
| sp\|Q15393\|SF3B3_HUMAN | plas | Splicing factor 3B subunit 3 OS=Homo sapiens OX=9606 GN=SF3B3 PE=1 SV=4 |
| sp\|O60635\|TSN1_HUMAN | plas | Tetraspanin-1 OS=Homo sapiens OX=9606 GN=TSPAN1 PE=1 SV=2 |
| sp\|O00423\|EMAL1_HUMAN | nucl | Echinoderm microtubule-associated protein-like 1 OS=Homo sapiens OX=9606 GN=EML1 PE=1 SV=3 |
| sp\|P12107\|COBA1_HUMAN | mito | Collagen alpha-1(XI) chain OS=Homo sapiens OX=9606 GN=COL11A1 PE=1 SV=4 |
| sp\|Q8IUD2\|RB6I2_HUMAN | nucl | ELKS/Rab6-interacting/CAST family member 1 OS=Homo sapiens OX=9606 GN=ERC1 PE=1 SV=1 |
| sp\|Q6NUK4\|REEP3_HUMAN | mito | Receptor expression-enhancing protein 3 OS=Homo sapiens OX=9606 GN=REEP3 PE=1 SV=1 |
| sp\|Q9BT43\|RPC7L_HUMAN | mito | DNA-directed RNA polymerase III subunit RPC7-like OS=Homo sapiens OX=9606 GN=POLR3GL PE=1 SV=1 |
| sp\|P05556\|ITB1_HUMAN | extr | Integrin beta-1 OS=Homo sapiens OX=9606 GN=ITGB1 PE=1 SV=2 |
| sp\|Q9UK45\|LSM7_HUMAN | mito | U6 snRNA-associated Sm-like protein LSm7 OS=Homo sapiens OX=9606 GN=LSM7 PE=1 SV=1 |
| sp\|P06744\|G6PI_HUMAN | cyto | Glucose-6-phosphate isomerase OS=Homo sapiens OX=9606 GN=GPI PE=1 SV=4 |
| sp\|Q8IX04\|UEVLD_HUMAN | cyto | Ubiquitin-conjugating enzyme E2 variant 3 OS=Homo sapiens OX=9606 GN=UEVLD PE=1 SV=2 |
| sp\|P61956\|SUMO2_HUMAN | cyto | Small ubiquitin-related modifier 2 OS=Homo sapiens OX=9606 GN=SUMO2 PE=1 SV=3 |
| sp\|Q14689\|DIP2A_HUMAN | plas | Disco-interacting protein 2 homolog A OS=Homo sapiens OX=9606 GN=DIP2A PE=1 SV=2 |
| sp\|Q5EBL4\|RIPL1_HUMAN | cyto | RILP-like protein 1 OS=Homo sapiens OX=9606 GN=RILPL1 PE=1 SV=1 |
| sp\|Q9Y624\|JAM1_HUMAN | extr | Junctional adhesion molecule A OS=Homo sapiens OX=9606 GN=F11R PE=1 SV=1 |
| sp\|Q96RK0\|CIC_HUMAN | nucl | Protein capicua homolog OS=Homo sapiens OX=9606 GN=CIC PE=1 SV=2 |
| sp\|P53007\|TXTP_HUMAN | plas | Tricarboxylate transport protein, mitochondrial OS=Homo sapiens OX=9606 GN=SLC25A1 PE=1 SV=2 |
| sp\|O95870\|ABHGA_HUMAN | mito | Protein ABHD16A OS=Homo sapiens OX=9606 GN=ABHD16A PE=1 SV=3 |
| sp\|Q9NZD4\|AHSP_HUMAN | cyto | Alpha-hemoglobin-stabilizing protein OS=Homo sapiens OX=9606 GN=AHSP PE=1 SV=1 |
| sp\|P03897\|NU3M_HUMAN | plas | NADH-ubiquinone oxidoreductase chain 3 OS=Homo sapiens OX=9606 GN=MT-ND3 PE=1 SV=1 |
| sp\|P05388\|RLA0_HUMAN | cyto_nucl | 60S acidic ribosomal protein P0 OS=Homo sapiens OX=9606 GN=RPLP0 PE=1 SV=1 |
| sp\|Q96P44\|COLA1_HUMAN | extr | Collagen alpha-1(XXI) chain OS=Homo sapiens OX=9606 GN=COL21A1 PE=2 SV=1 |
| sp\|Q5T5C0\|STXB5_HUMAN | nucl | Syntaxin-binding protein 5 OS=Homo sapiens OX=9606 GN=STXBP5 PE=1 SV=1 |
| sp\|P19367\|HXK1_HUMAN | cyto | Hexokinase-1 OS=Homo sapiens OX=9606 GN=HK1 PE=1 SV=3 |
| sp\|O43236\|SEPT4_HUMAN | cyto | Septin-4 OS=Homo sapiens OX=9606 GN=SEPT4 PE=1 SV=1 |
| sp\|Q6UW68\|TM205_HUMAN | extr | Transmembrane protein 205 OS=Homo sapiens OX=9606 GN=TMEM205 PE=1 SV=1 |
| sp\|Q9NUU6\|OTULL_HUMAN | plas | Inactive ubiquitin thioesterase OTULINL OS=Homo sapiens OX=9606 GN=OTULINL PE=2 SV=1 |
| sp\|Q9NV88\|INT9_HUMAN | nucl | Integrator complex subunit 9 OS=Homo sapiens OX=9606 GN=INTS9 PE=1 SV=2 |
| sp\|Q15417\|CNN3_HUMAN | cyto | Calponin-3 OS=Homo sapiens OX=9606 GN=CNN3 PE=1 SV=1 |
| sp\|P63313\|TYB10_HUMAN | cyto | Thymosin beta-10 OS=Homo sapiens OX=9606 GN=TMSB10 PE=1 SV=2 |
| sp\|Q9BQ13\|KCD14_HUMAN | nucl | BTB/POZ domain-containing protein KCTD14 OS=Homo sapiens OX=9606 GN=KCTD14 PE=1 SV=2 |
| sp\|Q8IZ81\|ELMD2_HUMAN | cyto | ELMO domain-containing protein 2 OS=Homo sapiens OX=9606 GN=ELMOD2 PE=1 SV=1 |
| sp\|A7E2V4\|ZSWM8_HUMAN | plas | Zinc finger SWIM domain-containing protein 8 OS=Homo sapiens OX=9606 GN=ZSWIM8 PE=1 SV=1 |
| sp\|Q15714\|T22D1_HUMAN | nucl | TSC22 domain family protein 1 OS=Homo sapiens OX=9606 GN=TSC22D1 PE=1 SV=3 |
| sp\|Q330K2\|NDUF6_HUMAN | mito | NADH dehydrogenase (ubiquinone) complex I, assembly factor 6 OS=Homo sapiens OX=9606 GN=NDUFAF6 PE=1 SV=2 |
| sp\|Q9NXJ5\|PGPI_HUMAN | extr | Pyroglutamyl-peptidase 1 OS=Homo sapiens OX=9606 GN=PGPEP1 PE=1 SV=1 |
| sp\|Q86XK7\|VSIG1_HUMAN | plas | V-set and immunoglobulin domain-containing protein 1 OS=Homo sapiens OX=9606 GN=VSIG1 PE=1 SV=1 |
| sp\|O94813\|SLIT2_HUMAN | extr | Slit homolog 2 protein OS=Homo sapiens OX=9606 GN=SLIT2 PE=1 SV=1 |
| sp\|Q01813\|PFKAP_HUMAN | cyto | ATP-dependent 6-phosphofructokinase, platelet type OS=Homo sapiens OX=9606 GN=PFKP PE=1 SV=2 |
| sp\|Q9C0A0\|CNTP4_HUMAN | plas | Contactin-associated protein-like 4 OS=Homo sapiens OX=9606 GN=CNTNAP4 PE=1 SV=3 |
| sp\|Q14C86\|GAPD1_HUMAN | plas | GTPase-activating protein and VPS9 domain-containing protein 1 OS=Homo sapiens OX=9606 GN=GAPVD1 PE=1 SV=2 |
| sp\|O95050\|INMT_HUMAN | plas | Indolethylamine N-methyltransferase OS=Homo sapiens OX=9606 GN=INMT PE=1 SV=3 |
| sp\|A0A075B6K4\|LV310_HUMAN | extr | Immunoglobulin lambda variable 3-10 OS=Homo sapiens OX=9606 GN=IGLV3-10 PE=3 SV=2 |
| sp\|Q96BQ5\|CC127_HUMAN | cyto_nucl | Coiled-coil domain-containing protein 127 OS=Homo sapiens OX=9606 GN=CCDC127 PE=1 SV=1 |
| sp\|Q13541\|4EBP1_HUMAN | mito | Eukaryotic translation initiation factor 4E-binding protein 1 OS=Homo sapiens OX=9606 GN=EIF4EBP1 PE=1 SV=3 |
| sp\|Q6PIU2\|NCEH1_HUMAN | extr | Neutral cholesterol ester hydrolase 1 OS=Homo sapiens OX=9606 GN=NCEH1 PE=1 SV=3 |
| sp\|Q14103\|HNRPD_HUMAN | nucl | Heterogeneous nuclear ribonucleoprotein D0 OS=Homo sapiens OX=9606 GN=HNRNPD PE=1 SV=1 |
| sp\|Q3SY17\|S2552_HUMAN | cyto | Solute carrier family 25 member 52 OS=Homo sapiens OX=9606 GN=SLC25A52 PE=2 SV=2 |
| sp\|Q6P4A8\|PLBL1_HUMAN | extr | Phospholipase B-like 1 OS=Homo sapiens OX=9606 GN=PLBD1 PE=1 SV=2 |
| sp\|P05106\|ITB3_HUMAN | plas | Integrin beta-3 OS=Homo sapiens OX=9606 GN=ITGB3 PE=1 SV=2 |
| sp\|Q16799\|RTN1_HUMAN | nucl | Reticulon-1 OS=Homo sapiens OX=9606 GN=RTN1 PE=1 SV=1 |
| sp\|P15151\|PVR_HUMAN | plas | Poliovirus receptor OS=Homo sapiens OX=9606 GN=PVR PE=1 SV=2 |
| sp\|P00846\|ATP6_HUMAN | plas | ATP synthase subunit a OS=Homo sapiens OX=9606 GN=MT-ATP6 PE=1 SV=1 |
| sp\|P49863\|GRAK_HUMAN | extr | Granzyme K OS=Homo sapiens OX=9606 GN=GZMK PE=1 SV=1 |
| sp\|O14975\|S27A2_HUMAN | E.R. | Very long-chain acyl-CoA synthetase OS=Homo sapiens OX=9606 GN=SLC27A2 PE=1 SV=2 |
| sp\|P11217\|PYGM_HUMAN | cyto | Glycogen phosphorylase, muscle form OS=Homo sapiens OX=9606 GN=PYGM PE=1 SV=6 |
| sp\|Q9H9B4\|SFXN1_HUMAN | cyto | Sideroflexin-1 OS=Homo sapiens OX=9606 GN=SFXN1 PE=1 SV=4 |
| sp\|Q9BXS9\|S26A6_HUMAN | plas | Solute carrier family 26 member 6 OS=Homo sapiens OX=9606 GN=SLC26A6 PE=1 SV=1 |
| sp\|Q14999\|CUL7_HUMAN | cyto_nucl | Cullin-7 OS=Homo sapiens OX=9606 GN=CUL7 PE=1 SV=2 |
| sp\|Q96CP2\|FWCH2_HUMAN | nucl | FLYWCH family member 2 OS=Homo sapiens OX=9606 GN=FLYWCH2 PE=1 SV=1 |
| sp\|Q14232\|EI2BA_HUMAN | cyto | Translation initiation factor eIF-2B subunit alpha OS=Homo sapiens OX=9606 GN=EIF2B1 PE=1 SV=1 |
| sp\|Q9BXJ8\|T120A_HUMAN | plas | Transmembrane protein 120A OS=Homo sapiens OX=9606 GN=TMEM120A PE=1 SV=1 |
| sp\|Q9Y262\|EIF3L_HUMAN | cyto | Eukaryotic translation initiation factor 3 subunit L OS=Homo sapiens OX=9606 GN=EIF3L PE=1 SV=1 |
| sp\|P04908\|H2A1B_HUMAN | nucl | Histone H2A type 1-B/E OS=Homo sapiens OX=9606 GN=HIST1H2AB PE=1 SV=2 |
| sp\|Q92621\|NU205_HUMAN | plas | Nuclear pore complex protein Nup205 OS=Homo sapiens OX=9606 GN=NUP205 PE=1 SV=3 |
| sp\|P20338\|RAB4A_HUMAN | cyto | Ras-related protein Rab-4A OS=Homo sapiens OX=9606 GN=RAB4A PE=1 SV=3 |
| sp\|Q70CQ2\|UBP34_HUMAN | plas | Ubiquitin carboxyl-terminal hydrolase 34 OS=Homo sapiens OX=9606 GN=USP34 PE=1 SV=2 |
| sp\|P13611\|CSPG2_HUMAN | mito | Versican core protein OS=Homo sapiens OX=9606 GN=VCAN PE=1 SV=3 |
| sp\|Q92729\|PTPRU_HUMAN | extr | Receptor-type tyrosine-protein phosphatase U OS=Homo sapiens OX=9606 GN=PTPRU PE=1 SV=2 |
| sp\|Q13618\|CUL3_HUMAN | nucl | Cullin-3 OS=Homo sapiens OX=9606 GN=CUL3 PE=1 SV=2 |
| sp\|Q96JA1\|LRIG1_HUMAN | extr | Leucine-rich repeats and immunoglobulin-like domains protein 1 OS=Homo sapiens OX=9606 GN=LRIG1 PE=1 SV=2 |
| sp\|Q8NDH3\|PEPL1_HUMAN | nucl | Probable aminopeptidase NPEPL1 OS=Homo sapiens OX=9606 GN=NPEPL1 PE=1 SV=3 |
| sp\|Q5SZK8\|FREM2_HUMAN | plas | FRAS1-related extracellular matrix protein 2 OS=Homo sapiens OX=9606 GN=FREM2 PE=1 SV=2 |
| sp\|Q8WXD2\|SCG3_HUMAN | E.R. | Secretogranin-3 OS=Homo sapiens OX=9606 GN=SCG3 PE=1 SV=3 |
| sp\|O14656\|TOR1A_HUMAN | E.R. | Torsin-1A OS=Homo sapiens OX=9606 GN=TOR1A PE=1 SV=1 |
| sp\|Q10471\|GALT2_HUMAN | extr | Polypeptide N-acetylgalactosaminyltransferase 2 OS=Homo sapiens OX=9606 GN=GALNT2 PE=1 SV=1 |
| sp\|O15321\|TM9S1_HUMAN | plas | Transmembrane 9 superfamily member 1 OS=Homo sapiens OX=9606 GN=TM9SF1 PE=2 SV=2 |
| sp\|Q9UFN0\|NPS3A_HUMAN | mito | Protein NipSnap homolog 3A OS=Homo sapiens OX=9606 GN=NIPSNAP3A PE=1 SV=2 |
| sp\|Q13098\|CSN1_HUMAN | cyto | COP9 signalosome complex subunit 1 OS=Homo sapiens OX=9606 GN=GPS1 PE=1 SV=4 |
| sp\|P53611\|PGTB2_HUMAN | cysk | Geranylgeranyl transferase type-2 subunit beta OS=Homo sapiens OX=9606 GN=RABGGTB PE=1 SV=2 |
| sp\|Q6R327\|RICTR_HUMAN | nucl | Rapamycin-insensitive companion of mTOR OS=Homo sapiens OX=9606 GN=RICTOR PE=1 SV=1 |
| sp\|P55060\|XPO2_HUMAN | cyto_nucl | Exportin-2 OS=Homo sapiens OX=9606 GN=CSE1L PE=1 SV=3 |
| sp\|P12235\|ADT1_HUMAN | mito | ADP/ATP translocase 1 OS=Homo sapiens OX=9606 GN=SLC25A4 PE=1 SV=4 |
| sp\|Q7Z3E5\|ARMC9_HUMAN | cysk | LisH domain-containing protein ARMC9 OS=Homo sapiens OX=9606 GN=ARMC9 PE=1 SV=3 |
| sp\|Q9BZG1\|RAB34_HUMAN | cyto | Ras-related protein Rab-34 OS=Homo sapiens OX=9606 GN=RAB34 PE=1 SV=1 |
| sp\|P42566\|EPS15_HUMAN | nucl | Epidermal growth factor receptor substrate 15 OS=Homo sapiens OX=9606 GN=EPS15 PE=1 SV=2 |
| sp\|Q05315\|LEG10_HUMAN | cyto | Galectin-10 OS=Homo sapiens OX=9606 GN=CLC PE=1 SV=3 |
| sp\|Q8IV36\|HID1_HUMAN | cyto | Protein HID1 OS=Homo sapiens OX=9606 GN=HID1 PE=1 SV=1 |
| sp\|P27449\|VATL_HUMAN | plas | V-type proton ATPase 16 kDa proteolipid subunit OS=Homo sapiens OX=9606 GN=ATP6V0C PE=1 SV=1 |
| sp\|O43715\|TRIA1_HUMAN | extr | TP53-regulated inhibitor of apoptosis 1 OS=Homo sapiens OX=9606 GN=TRIAP1 PE=1 SV=1 |
| sp\|Q9Y3Y2\|CHTOP_HUMAN | mito | Chromatin target of PRMT1 protein OS=Homo sapiens OX=9606 GN=CHTOP PE=1 SV=2 |
| sp\|P62736\|ACTA_HUMAN | cysk | Actin, aortic smooth muscle OS=Homo sapiens OX=9606 GN=ACTA2 PE=1 SV=1 |
| sp\|O43657\|TSN6_HUMAN | plas | Tetraspanin-6 OS=Homo sapiens OX=9606 GN=TSPAN6 PE=1 SV=1 |
| sp\|P28845\|DHI1_HUMAN | pero | Corticosteroid 11-beta-dehydrogenase isozyme 1 OS=Homo sapiens OX=9606 GN=HSD11B1 PE=1 SV=3 |
| sp\|Q9UKR5\|ERG28_HUMAN | plas | Probable ergosterol biosynthetic protein 28 OS=Homo sapiens OX=9606 GN=ERG28 PE=1 SV=1 |
| sp\|O14925\|TIM23_HUMAN | cyto | Mitochondrial import inner membrane translocase subunit Tim23 OS=Homo sapiens OX=9606 GN=TIMM23 PE=1 SV=1 |
| sp\|B7ZAQ6\|GPHRA_HUMAN | plas | Golgi pH regulator A OS=Homo sapiens OX=9606 GN=GPR89A PE=1 SV=2 >sp\|P0CG08\|GPHRB_HUMAN Golgi pH regulator B OS=Homo sapiens OX=9606 GN=GPR89B PE=1 SV=1 |
| sp\|O95139\|NDUB6_HUMAN | cyto | NADH dehydrogenase [ubiquinone] 1 beta subcomplex subunit 6 OS=Homo sapiens OX=9606 GN=NDUFB6 PE=1 SV=3 |
| sp\|P02652\|APOA2_HUMAN | extr | Apolipoprotein A-II OS=Homo sapiens OX=9606 GN=APOA2 PE=1 SV=1 |
| sp\|P51884\|LUM_HUMAN | extr | Lumican OS=Homo sapiens OX=9606 GN=LUM PE=1 SV=2 |
| sp\|Q86VP6\|CAND1_HUMAN | cyto | Cullin-associated NEDD8-dissociated protein 1 OS=Homo sapiens OX=9606 GN=CAND1 PE=1 SV=2 |
| sp\|Q96GW9\|SYMM_HUMAN | mito | Methionine--tRNA ligase, mitochondrial OS=Homo sapiens OX=9606 GN=MARS2 PE=1 SV=2 |
| sp\|P51911\|CNN1_HUMAN | cyto | Calponin-1 OS=Homo sapiens OX=9606 GN=CNN1 PE=1 SV=2 |
| sp\|P53396\|ACLY_HUMAN | cyto | ATP-citrate synthase OS=Homo sapiens OX=9606 GN=ACLY PE=1 SV=3 |
| sp\|Q9UJS0\|CMC2_HUMAN | cyto | Calcium-binding mitochondrial carrier protein Aralar2 OS=Homo sapiens OX=9606 GN=SLC25A13 PE=1 SV=2 |
| sp\|P46937\|YAP1_HUMAN | nucl | Transcriptional coactivator YAP1 OS=Homo sapiens OX=9606 GN=YAP1 PE=1 SV=2 |
| sp\|P02144\|MYG_HUMAN | cyto | Myoglobin OS=Homo sapiens OX=9606 GN=MB PE=1 SV=2 |
| sp\|P29622\|KAIN_HUMAN | plas | Kallistatin OS=Homo sapiens OX=9606 GN=SERPINA4 PE=1 SV=3 |
| sp\|P54868\|HMCS2_HUMAN | mito | Hydroxymethylglutaryl-CoA synthase, mitochondrial OS=Homo sapiens OX=9606 GN=HMGCS2 PE=1 SV=1 |
| sp\|Q9GZU5\|NYX_HUMAN | extr | Nyctalopin OS=Homo sapiens OX=9606 GN=NYX PE=1 SV=1 |
| sp\|Q9Y3C1\|NOP16_HUMAN | nucl | Nucleolar protein 16 OS=Homo sapiens OX=9606 GN=NOP16 PE=1 SV=2 |
| sp\|Q8WVT3\|TPC12_HUMAN | nucl | Trafficking protein particle complex subunit 12 OS=Homo sapiens OX=9606 GN=TRAPPC12 PE=1 SV=3 |
| sp\|Q9UJ83\|HACL1_HUMAN | plas | 2-hydroxyacyl-CoA lyase 1 OS=Homo sapiens OX=9606 GN=HACL1 PE=1 SV=2 |
| sp\|Q13501\|SQSTM_HUMAN | nucl | Sequestosome-1 OS=Homo sapiens OX=9606 GN=SQSTM1 PE=1 SV=1 |
| sp\|P10153\|RNAS2_HUMAN | extr | Non-secretory ribonuclease OS=Homo sapiens OX=9606 GN=RNASE2 PE=1 SV=2 |
| sp\|Q92871\|PMM1_HUMAN | cyto | Phosphomannomutase 1 OS=Homo sapiens OX=9606 GN=PMM1 PE=1 SV=2 |
| sp\|Q86X52\|CHSS1_HUMAN | E.R. | Chondroitin sulfate synthase 1 OS=Homo sapiens OX=9606 GN=CHSY1 PE=1 SV=3 |
| sp\|Q9Y2Y8\|PRG3_HUMAN | extr | Proteoglycan 3 OS=Homo sapiens OX=9606 GN=PRG3 PE=1 SV=2 |
| sp\|Q30154\|DRB5_HUMAN | extr | HLA class II histocompatibility antigen, DR beta 5 chain OS=Homo sapiens OX=9606 GN=HLA-DRB5 PE=1 SV=1 |
| sp\|Q12905\|ILF2_HUMAN | cyto | Interleukin enhancer-binding factor 2 OS=Homo sapiens OX=9606 GN=ILF2 PE=1 SV=2 |
| sp\|P51159\|RB27A_HUMAN | cyto | Ras-related protein Rab-27A OS=Homo sapiens OX=9606 GN=RAB27A PE=1 SV=3 |
| sp\|Q8N5B7\|CERS5_HUMAN | plas | Ceramide synthase 5 OS=Homo sapiens OX=9606 GN=CERS5 PE=2 SV=1 |
| sp\|Q5TBA9\|FRY_HUMAN | plas | Protein furry homolog OS=Homo sapiens OX=9606 GN=FRY PE=1 SV=1 |
| sp\|O00763\|ACACB_HUMAN | plas | Acetyl-CoA carboxylase 2 OS=Homo sapiens OX=9606 GN=ACACB PE=1 SV=3 |
| sp\|Q9NSB2\|KRT84_HUMAN | mito | Keratin, type II cuticular Hb4 OS=Homo sapiens OX=9606 GN=KRT84 PE=2 SV=2 |
| sp\|O60240\|PLIN1_HUMAN | nucl | Perilipin-1 OS=Homo sapiens OX=9606 GN=PLIN1 PE=1 SV=2 |
| sp\|P49354\|FNTA_HUMAN | cyto | Protein farnesyltransferase/geranylgeranyltransferase type-1 subunit alpha OS=Homo sapiens OX=9606 GN=FNTA PE=1 SV=1 |
| sp\|Q14934\|NFAC4_HUMAN | nucl | Nuclear factor of activated T-cells, cytoplasmic 4 OS=Homo sapiens OX=9606 GN=NFATC4 PE=1 SV=2 |
| sp\|P09668\|CATH_HUMAN | extr | Pro-cathepsin H OS=Homo sapiens OX=9606 GN=CTSH PE=1 SV=4 |
| sp\|Q5T870\|PRR9_HUMAN | extr | Proline-rich protein 9 OS=Homo sapiens OX=9606 GN=PRR9 PE=4 SV=1 |
| sp\|Q9Y2D5\|AKAP2_HUMAN | nucl | A-kinase anchor protein 2 OS=Homo sapiens OX=9606 GN=AKAP2 PE=1 SV=3 |
| sp\|Q8NEY8\|PPHLN_HUMAN | nucl | Periphilin-1 OS=Homo sapiens OX=9606 GN=PPHLN1 PE=1 SV=2 |
| sp\|Q13769\|THOC5_HUMAN | nucl | THO complex subunit 5 homolog OS=Homo sapiens OX=9606 GN=THOC5 PE=1 SV=2 |
| sp\|P98082\|DAB2_HUMAN | nucl | Disabled homolog 2 OS=Homo sapiens OX=9606 GN=DAB2 PE=1 SV=3 |
| sp\|Q9H7E9\|CH033_HUMAN | nucl | UPF0488 protein C8orf33 OS=Homo sapiens OX=9606 GN=C8orf33 PE=1 SV=1 |
| sp\|O15438\|MRP3_HUMAN | plas | Canalicular multispecific organic anion transporter 2 OS=Homo sapiens OX=9606 GN=ABCC3 PE=1 SV=3 |
| sp\|P01031\|CO5_HUMAN | E.R. | Complement C5 OS=Homo sapiens OX=9606 GN=C5 PE=1 SV=4 |
| sp\|P42765\|THIM_HUMAN | mito | 3-ketoacyl-CoA thiolase, mitochondrial OS=Homo sapiens OX=9606 GN=ACAA2 PE=1 SV=2 |
| sp\|P00167\|CYB5_HUMAN | cyto | Cytochrome b5 OS=Homo sapiens OX=9606 GN=CYB5A PE=1 SV=2 |
| sp\|Q8IYJ1\|CPNE9_HUMAN | cyto | Copine-9 OS=Homo sapiens OX=9606 GN=CPNE9 PE=1 SV=3 |
| sp\|Q9UBR2\|CATZ_HUMAN | extr | Cathepsin Z OS=Homo sapiens OX=9606 GN=CTSZ PE=1 SV=1 |
| sp\|Q9UBV7\|B4GT7_HUMAN | cyto | Beta-1,4-galactosyltransferase 7 OS=Homo sapiens OX=9606 GN=B4GALT7 PE=1 SV=1 |
| sp\|O60522\|TDRD6_HUMAN | plas | Tudor domain-containing protein 6 OS=Homo sapiens OX=9606 GN=TDRD6 PE=2 SV=2 |
| sp\|Q5VWZ2\|LYPL1_HUMAN | cyto | Lysophospholipase-like protein 1 OS=Homo sapiens OX=9606 GN=LYPLAL1 PE=1 SV=3 |
| sp\|Q86VS8\|HOOK3_HUMAN | nucl | Protein Hook homolog 3 OS=Homo sapiens OX=9606 GN=HOOK3 PE=1 SV=2 |
| sp\|Q9UNL2\|SSRG_HUMAN | plas | Translocon-associated protein subunit gamma OS=Homo sapiens OX=9606 GN=SSR3 PE=1 SV=1 |
| sp\|Q86SF2\|GALT7_HUMAN | E.R. | N-acetylgalactosaminyltransferase 7 OS=Homo sapiens OX=9606 GN=GALNT7 PE=1 SV=1 |
| sp\|Q04695\|K1C17_HUMAN | mito | Keratin, type I cytoskeletal 17 OS=Homo sapiens OX=9606 GN=KRT17 PE=1 SV=2 |
| sp\|Q13572\|ITPK1_HUMAN | cyto | Inositol-tetrakisphosphate 1-kinase OS=Homo sapiens OX=9606 GN=ITPK1 PE=1 SV=2 |
| sp\|Q9Y6C9\|MTCH2_HUMAN | extr | Mitochondrial carrier homolog 2 OS=Homo sapiens OX=9606 GN=MTCH2 PE=1 SV=1 |
| sp\|Q14508\|WFDC2_HUMAN | extr | WAP four-disulfide core domain protein 2 OS=Homo sapiens OX=9606 GN=WFDC2 PE=1 SV=2 |
| sp\|Q9P2R6\|RERE_HUMAN | nucl | Arginine-glutamic acid dipeptide repeats protein OS=Homo sapiens OX=9606 GN=RERE PE=1 SV=2 |
| sp\|Q0VF96\|CGNL1_HUMAN | nucl | Cingulin-like protein 1 OS=Homo sapiens OX=9606 GN=CGNL1 PE=1 SV=2 |
| sp\|P54725\|RD23A_HUMAN | mito | UV excision repair protein RAD23 homolog A OS=Homo sapiens OX=9606 GN=RAD23A PE=1 SV=1 |
| sp\|P16403\|H12_HUMAN | nucl | Histone H1.2 OS=Homo sapiens OX=9606 GN=HIST1H1C PE=1 SV=2 |
| sp\|Q6P5S2\|LEG1H_HUMAN | extr | Protein LEG1 homolog OS=Homo sapiens OX=9606 GN=LEG1 PE=1 SV=2 |
| sp\|Q969N2\|PIGT_HUMAN | plas | GPI transamidase component PIG-T OS=Homo sapiens OX=9606 GN=PIGT PE=1 SV=1 |
| sp\|Q76N32\|CEP68_HUMAN | nucl | Centrosomal protein of 68 kDa OS=Homo sapiens OX=9606 GN=CEP68 PE=1 SV=2 |
| sp\|O95834\|EMAL2_HUMAN | plas | Echinoderm microtubule-associated protein-like 2 OS=Homo sapiens OX=9606 GN=EML2 PE=1 SV=1 |
| sp\|Q13574\|DGKZ_HUMAN | nucl | Diacylglycerol kinase zeta OS=Homo sapiens OX=9606 GN=DGKZ PE=1 SV=3 |
| sp\|P62330\|ARF6_HUMAN | cyto | ADP-ribosylation factor 6 OS=Homo sapiens OX=9606 GN=ARF6 PE=1 SV=2 |
| sp\|O00193\|SMAP_HUMAN | nucl | Small acidic protein OS=Homo sapiens OX=9606 GN=SMAP PE=1 SV=1 |
| sp\|Q6UX53\|MET7B_HUMAN | extr | Methyltransferase-like protein 7B OS=Homo sapiens OX=9606 GN=METTL7B PE=1 SV=2 |
| sp\|Q99973\|TEP1_HUMAN | nucl | Telomerase protein component 1 OS=Homo sapiens OX=9606 GN=TEP1 PE=1 SV=2 |
| sp\|P67809\|YBOX1_HUMAN | nucl | Nuclease-sensitive element-binding protein 1 OS=Homo sapiens OX=9606 GN=YBX1 PE=1 SV=3 |
| sp\|Q04656\|ATP7A_HUMAN | plas | Copper-transporting ATPase 1 OS=Homo sapiens OX=9606 GN=ATP7A PE=1 SV=4 |
| sp\|O14972\|DSCR3_HUMAN | cyto | Down syndrome critical region protein 3 OS=Homo sapiens OX=9606 GN=DSCR3 PE=2 SV=1 |
| sp\|Q9UBV8\|PEF1_HUMAN | nucl | Peflin OS=Homo sapiens OX=9606 GN=PEF1 PE=1 SV=1 |
| sp\|Q6V1P9\|PCD23_HUMAN | cyto | Protocadherin-23 OS=Homo sapiens OX=9606 GN=DCHS2 PE=2 SV=1 |
| sp\|Q12769\|NU160_HUMAN | cyto_nucl | Nuclear pore complex protein Nup160 OS=Homo sapiens OX=9606 GN=NUP160 PE=1 SV=3 |
| sp\|O14791\|APOL1_HUMAN | E.R. | Apolipoprotein L1 OS=Homo sapiens OX=9606 GN=APOL1 PE=1 SV=5 |
| sp\|P08842\|STS_HUMAN | plas | Steryl-sulfatase OS=Homo sapiens OX=9606 GN=STS PE=1 SV=2 |
| sp\|Q6FHJ7\|SFRP4_HUMAN | extr | Secreted frizzled-related protein 4 OS=Homo sapiens OX=9606 GN=SFRP4 PE=1 SV=2 |
| sp\|Q9HCJ6\|VAT1L_HUMAN | cyto | Synaptic vesicle membrane protein VAT-1 homolog-like OS=Homo sapiens OX=9606 GN=VAT1L PE=1 SV=2 |
| sp\|Q8NI22\|MCFD2_HUMAN | mito | Multiple coagulation factor deficiency protein 2 OS=Homo sapiens OX=9606 GN=MCFD2 PE=1 SV=1 |
| sp\|P13727\|PRG2_HUMAN | extr | Bone marrow proteoglycan OS=Homo sapiens OX=9606 GN=PRG2 PE=1 SV=2 |
| sp\|Q9HB90\|RRAGC_HUMAN | cyto | Ras-related GTP-binding protein C OS=Homo sapiens OX=9606 GN=RRAGC PE=1 SV=1 |
| sp\|P0DP23\|CALM1_HUMAN | cyto_nucl | Calmodulin-1 OS=Homo sapiens OX=9606 GN=CALM1 PE=1 SV=1 >sp\|P0DP24\|CALM2_HUMAN Calmodulin-2 OS=Homo sapiens OX=9606 GN=CALM2 PE=1 SV=1 >sp\|P0DP25\|CALM3_HUMAN Calmodulin-3 OS=Homo sapiens OX=9606 GN=CALM3 PE=1 SV=1 |
| sp\|Q9Y5P6\|GMPPB_HUMAN | mito | Mannose-1-phosphate guanyltransferase beta OS=Homo sapiens OX=9606 GN=GMPPB PE=1 SV=2 |
| sp\|P09960\|LKHA4_HUMAN | cyto | Leukotriene A-4 hydrolase OS=Homo sapiens OX=9606 GN=LTA4H PE=1 SV=2 |
| sp\|Q99460\|PSMD1_HUMAN | cyto | 26S proteasome non-ATPase regulatory subunit 1 OS=Homo sapiens OX=9606 GN=PSMD1 PE=1 SV=2 |
| sp\|B2RTY4\|MYO9A_HUMAN | nucl | Unconventional myosin-IXa OS=Homo sapiens OX=9606 GN=MYO9A PE=1 SV=2 |
| sp\|P07585\|PGS2_HUMAN | extr | Decorin OS=Homo sapiens OX=9606 GN=DCN PE=1 SV=1 |
| sp\|Q9Y5Z0\|BACE2_HUMAN | plas | Beta-secretase 2 OS=Homo sapiens OX=9606 GN=BACE2 PE=1 SV=1 |
| sp\|Q9UNH7\|SNX6_HUMAN | cyto | Sorting nexin-6 OS=Homo sapiens OX=9606 GN=SNX6 PE=1 SV=1 |
| sp\|L0R6Q1\|S35U4_HUMAN | cyto | SLC35A4 upstream open reading frame protein OS=Homo sapiens OX=9606 GN=SLC35A4 PE=3 SV=1 |
| sp\|Q9UL45\|BL1S6_HUMAN | nucl | Biogenesis of lysosome-related organelles complex 1 subunit 6 OS=Homo sapiens OX=9606 GN=BLOC1S6 PE=1 SV=1 |
| sp\|Q99571\|P2RX4_HUMAN | plas | P2X purinoceptor 4 OS=Homo sapiens OX=9606 GN=P2RX4 PE=1 SV=2 |
| sp\|Q9UKK6\|NXT1_HUMAN | nucl | NTF2-related export protein 1 OS=Homo sapiens OX=9606 GN=NXT1 PE=1 SV=1 |
| sp\|Q7Z6Z7\|HUWE1_HUMAN | plas | E3 ubiquitin-protein ligase HUWE1 OS=Homo sapiens OX=9606 GN=HUWE1 PE=1 SV=3 |
| sp\|O96008\|TOM40_HUMAN | nucl | Mitochondrial import receptor subunit TOM40 homolog OS=Homo sapiens OX=9606 GN=TOMM40 PE=1 SV=1 |
| sp\|O14880\|MGST3_HUMAN | extr | Microsomal glutathione S-transferase 3 OS=Homo sapiens OX=9606 GN=MGST3 PE=1 SV=1 |
| sp\|Q86YS7\|C2CD5_HUMAN | nucl | C2 domain-containing protein 5 OS=Homo sapiens OX=9606 GN=C2CD5 PE=1 SV=1 |
| sp\|O43747\|AP1G1_HUMAN | mito | AP-1 complex subunit gamma-1 OS=Homo sapiens OX=9606 GN=AP1G1 PE=1 SV=5 |
| sp\|Q8IWT6\|LRC8A_HUMAN | plas | Volume-regulated anion channel subunit LRRC8A OS=Homo sapiens OX=9606 GN=LRRC8A PE=1 SV=1 |
| sp\|P50993\|AT1A2_HUMAN | plas | Sodium/potassium-transporting ATPase subunit alpha-2 OS=Homo sapiens OX=9606 GN=ATP1A2 PE=1 SV=1 |
| sp\|Q02083\|NAAA_HUMAN | extr | N-acylethanolamine-hydrolyzing acid amidase OS=Homo sapiens OX=9606 GN=NAAA PE=1 SV=3 |
| sp\|Q9NUB1\|ACS2L_HUMAN | mito | Acetyl-coenzyme A synthetase 2-like, mitochondrial OS=Homo sapiens OX=9606 GN=ACSS1 PE=1 SV=2 |
| sp\|Q6Y1H2\|HACD2_HUMAN | plas | Very-long-chain (3R)-3-hydroxyacyl-CoA dehydratase 2 OS=Homo sapiens OX=9606 GN=HACD2 PE=1 SV=1 |
| sp\|Q2WGJ9\|FR1L6_HUMAN | cyto | Fer-1-like protein 6 OS=Homo sapiens OX=9606 GN=FER1L6 PE=2 SV=2 |
| sp\|P05204\|HMGN2_HUMAN | nucl | Non-histone chromosomal protein HMG-17 OS=Homo sapiens OX=9606 GN=HMGN2 PE=1 SV=3 |
| sp\|O00194\|RB27B_HUMAN | cyto | Ras-related protein Rab-27B OS=Homo sapiens OX=9606 GN=RAB27B PE=1 SV=4 |
| sp\|O60462\|NRP2_HUMAN | pero | Neuropilin-2 OS=Homo sapiens OX=9606 GN=NRP2 PE=1 SV=3 |
| sp\|Q9H6S1\|AZI2_HUMAN | nucl | 5-azacytidine-induced protein 2 OS=Homo sapiens OX=9606 GN=AZI2 PE=1 SV=1 |
| sp\|O14562\|UBFD1_HUMAN | cyto | Ubiquitin domain-containing protein UBFD1 OS=Homo sapiens OX=9606 GN=UBFD1 PE=1 SV=2 |
| sp\|Q92791\|SC65_HUMAN | extr | Endoplasmic reticulum protein SC65 OS=Homo sapiens OX=9606 GN=P3H4 PE=1 SV=1 |
| sp\|Q68CQ4\|DIEXF_HUMAN | nucl | Digestive organ expansion factor homolog OS=Homo sapiens OX=9606 GN=DIEXF PE=1 SV=2 |
| sp\|O15117\|FYB1_HUMAN | nucl | FYN-binding protein 1 OS=Homo sapiens OX=9606 GN=FYB1 PE=1 SV=2 |
| sp\|Q8NBU5\|ATAD1_HUMAN | plas | ATPase family AAA domain-containing protein 1 OS=Homo sapiens OX=9606 GN=ATAD1 PE=1 SV=1 |
| sp\|P21695\|GPDA_HUMAN | cyto | Glycerol-3-phosphate dehydrogenase [NAD(+)], cytoplasmic OS=Homo sapiens OX=9606 GN=GPD1 PE=1 SV=4 |
| sp\|Q96J88\|ESIP1_HUMAN | mito | Epithelial-stromal interaction protein 1 OS=Homo sapiens OX=9606 GN=EPSTI1 PE=2 SV=2 |
| sp\|Q96BI1\|S22AI_HUMAN | plas | Solute carrier family 22 member 18 OS=Homo sapiens OX=9606 GN=SLC22A18 PE=1 SV=3 |
| sp\|Q63HM2\|PCX4_HUMAN | plas | Pecanex-like protein 4 OS=Homo sapiens OX=9606 GN=PCNX4 PE=1 SV=4 |
| sp\|Q86YR7\|MF2L2_HUMAN | nucl | Probable guanine nucleotide exchange factor MCF2L2 OS=Homo sapiens OX=9606 GN=MCF2L2 PE=2 SV=3 |
| sp\|Q9NW15\|ANO10_HUMAN | plas | Anoctamin-10 OS=Homo sapiens OX=9606 GN=ANO10 PE=1 SV=2 |
| sp\|Q16626\|MEA1_HUMAN | cyto | Male-enhanced antigen 1 OS=Homo sapiens OX=9606 GN=MEA1 PE=1 SV=2 |
| sp\|P56199\|ITA1_HUMAN | plas | Integrin alpha-1 OS=Homo sapiens OX=9606 GN=ITGA1 PE=1 SV=2 |
| sp\|O75746\|CMC1_HUMAN | cyto | Calcium-binding mitochondrial carrier protein Aralar1 OS=Homo sapiens OX=9606 GN=SLC25A12 PE=1 SV=2 |
| sp\|Q969X5\|ERGI1_HUMAN | plas | Endoplasmic reticulum-Golgi intermediate compartment protein 1 OS=Homo sapiens OX=9606 GN=ERGIC1 PE=1 SV=1 |
| sp\|Q13362\|2A5G_HUMAN | cyto | Serine/threonine-protein phosphatase 2A 56 kDa regulatory subunit gamma isoform OS=Homo sapiens OX=9606 GN=PPP2R5C PE=1 SV=3 |
| sp\|Q8N474\|SFRP1_HUMAN | plas | Secreted frizzled-related protein 1 OS=Homo sapiens OX=9606 GN=SFRP1 PE=1 SV=1 |
| sp\|Q7L7V1\|DHX32_HUMAN | cyto | Putative pre-mRNA-splicing factor ATP-dependent RNA helicase DHX32 OS=Homo sapiens OX=9606 GN=DHX32 PE=1 SV=1 |
| sp\|P15309\|PPAP_HUMAN | extr | Prostatic acid phosphatase OS=Homo sapiens OX=9606 GN=ACPP PE=1 SV=3 |
| sp\|Q86U38\|NOP9_HUMAN | nucl | Nucleolar protein 9 OS=Homo sapiens OX=9606 GN=NOP9 PE=1 SV=1 |
| sp\|Q969P0\|IGSF8_HUMAN | extr | Immunoglobulin superfamily member 8 OS=Homo sapiens OX=9606 GN=IGSF8 PE=1 SV=1 |
| sp\|O94929\|ABLM3_HUMAN | nucl | Actin-binding LIM protein 3 OS=Homo sapiens OX=9606 GN=ABLIM3 PE=1 SV=3 |
| sp\|Q15293\|RCN1_HUMAN | extr | Reticulocalbin-1 OS=Homo sapiens OX=9606 GN=RCN1 PE=1 SV=1 |
| sp\|O75891\|AL1L1_HUMAN | cyto | Cytosolic 10-formyltetrahydrofolate dehydrogenase OS=Homo sapiens OX=9606 GN=ALDH1L1 PE=1 SV=2 |
| sp\|P04259\|K2C6B_HUMAN | nucl | Keratin, type II cytoskeletal 6B OS=Homo sapiens OX=9606 GN=KRT6B PE=1 SV=5 |
| sp\|P05997\|CO5A2_HUMAN | E.R. | Collagen alpha-2(V) chain OS=Homo sapiens OX=9606 GN=COL5A2 PE=1 SV=3 |
| sp\|Q969U7\|PSMG2_HUMAN | cyto | Proteasome assembly chaperone 2 OS=Homo sapiens OX=9606 GN=PSMG2 PE=1 SV=1 |
| sp\|Q9NVA1\|UQCC1_HUMAN | mito | Ubiquinol-cytochrome-c reductase complex assembly factor 1 OS=Homo sapiens OX=9606 GN=UQCC1 PE=1 SV=3 |
| sp\|Q96GM8\|TOE1_HUMAN | nucl | Target of EGR1 protein 1 OS=Homo sapiens OX=9606 GN=TOE1 PE=1 SV=1 |
| sp\|Q5XXA6\|ANO1_HUMAN | plas | Anoctamin-1 OS=Homo sapiens OX=9606 GN=ANO1 PE=1 SV=1 |
| sp\|Q14204\|DYHC1_HUMAN | cyto | Cytoplasmic dynein 1 heavy chain 1 OS=Homo sapiens OX=9606 GN=DYNC1H1 PE=1 SV=5 |
| sp\|P63096\|GNAI1_HUMAN | cyto | Guanine nucleotide-binding protein G(i) subunit alpha-1 OS=Homo sapiens OX=9606 GN=GNAI1 PE=1 SV=2 |
| sp\|Q9NV96\|CC50A_HUMAN | plas | Cell cycle control protein 50A OS=Homo sapiens OX=9606 GN=TMEM30A PE=1 SV=1 |
| sp\|P16949\|STMN1_HUMAN | nucl | Stathmin OS=Homo sapiens OX=9606 GN=STMN1 PE=1 SV=3 |
| sp\|O15111\|IKKA_HUMAN | cyto | Inhibitor of nuclear factor kappa-B kinase subunit alpha OS=Homo sapiens OX=9606 GN=CHUK PE=1 SV=2 |
| sp\|P09382\|LEG1_HUMAN | extr | Galectin-1 OS=Homo sapiens OX=9606 GN=LGALS1 PE=1 SV=2 |
| sp\|O43520\|AT8B1_HUMAN | plas | Phospholipid-transporting ATPase IC OS=Homo sapiens OX=9606 GN=ATP8B1 PE=1 SV=3 |
| sp\|P49720\|PSB3_HUMAN | cyto | Proteasome subunit beta type-3 OS=Homo sapiens OX=9606 GN=PSMB3 PE=1 SV=2 |
| sp\|Q8WTS6\|SETD7_HUMAN | cyto | Histone-lysine N-methyltransferase SETD7 OS=Homo sapiens OX=9606 GN=SETD7 PE=1 SV=1 |
| sp\|P05109\|S10A8_HUMAN | mito | Protein S100-A8 OS=Homo sapiens OX=9606 GN=S100A8 PE=1 SV=1 |
| sp\|Q9UPN4\|CP131_HUMAN | cyto_nucl | Centrosomal protein of 131 kDa OS=Homo sapiens OX=9606 GN=CEP131 PE=1 SV=3 |
| sp\|O15460\|P4HA2_HUMAN | extr | Prolyl 4-hydroxylase subunit alpha-2 OS=Homo sapiens OX=9606 GN=P4HA2 PE=1 SV=1 |
| sp\|Q96LT7\|CI072_HUMAN | cyto_mito | Guanine nucleotide exchange C9orf72 OS=Homo sapiens OX=9606 GN=C9orf72 PE=1 SV=2 |
| sp\|Q6NUJ1\|SAPL1_HUMAN | extr | Proactivator polypeptide-like 1 OS=Homo sapiens OX=9606 GN=PSAPL1 PE=2 SV=2 |
| sp\|Q14914\|PTGR1_HUMAN | cyto | Prostaglandin reductase 1 OS=Homo sapiens OX=9606 GN=PTGR1 PE=1 SV=2 |
| sp\|P28074\|PSB5_HUMAN | nucl | Proteasome subunit beta type-5 OS=Homo sapiens OX=9606 GN=PSMB5 PE=1 SV=3 |
| sp\|Q5EB52\|MEST_HUMAN | extr | Mesoderm-specific transcript homolog protein OS=Homo sapiens OX=9606 GN=MEST PE=2 SV=2 |
| sp\|Q8IVN8\|SBSPO_HUMAN | extr | Somatomedin-B and thrombospondin type-1 domain-containing protein OS=Homo sapiens OX=9606 GN=SBSPON PE=1 SV=2 |
| sp\|Q6ZUT6\|CCD9B_HUMAN | extr | Coiled-coil domain-containing protein 9B OS=Homo sapiens OX=9606 GN=CCDC9B PE=1 SV=1 |
| sp\|Q8IX19\|MCEM1_HUMAN | plas | Mast cell-expressed membrane protein 1 OS=Homo sapiens OX=9606 GN=MCEMP1 PE=1 SV=1 |
| sp\|C9JLW8\|MCRI1_HUMAN | nucl | Mapk-regulated corepressor-interacting protein 1 OS=Homo sapiens OX=9606 GN=MCRIP1 PE=1 SV=1 |
| sp\|Q9NZ01\|TECR_HUMAN | cyto | Very-long-chain enoyl-CoA reductase OS=Homo sapiens OX=9606 GN=TECR PE=1 SV=1 |
| sp\|Q5ST30\|SYVM_HUMAN | mito | Valine--tRNA ligase, mitochondrial OS=Homo sapiens OX=9606 GN=VARS2 PE=1 SV=2 |
| sp\|Q70UQ0\|IKIP_HUMAN | plas | Inhibitor of nuclear factor kappa-B kinase-interacting protein OS=Homo sapiens OX=9606 GN=IKBIP PE=1 SV=1 |
| sp\|Q8IXB3\|TARG1_HUMAN | plas | Trafficking regulator of GLUT4 1 OS=Homo sapiens OX=9606 GN=TRARG1 PE=2 SV=2 |
| sp\|Q8N5M9\|JAGN1_HUMAN | plas | Protein jagunal homolog 1 OS=Homo sapiens OX=9606 GN=JAGN1 PE=1 SV=1 |
| sp\|Q86W92\|LIPB1_HUMAN | nucl | Liprin-beta-1 OS=Homo sapiens OX=9606 GN=PPFIBP1 PE=1 SV=2 |
| sp\|P35219\|CAH8_HUMAN | cysk | Carbonic anhydrase-related protein OS=Homo sapiens OX=9606 GN=CA8 PE=1 SV=3 |
| sp\|Q96RL7\|VP13A_HUMAN | plas | Vacuolar protein sorting-associated protein 13A OS=Homo sapiens OX=9606 GN=VPS13A PE=1 SV=2 |
| sp\|P67775\|PP2AA_HUMAN | cyto | Serine/threonine-protein phosphatase 2A catalytic subunit alpha isoform OS=Homo sapiens OX=9606 GN=PPP2CA PE=1 SV=1 |
| sp\|P78310\|CXAR_HUMAN | plas | Coxsackievirus and adenovirus receptor OS=Homo sapiens OX=9606 GN=CXADR PE=1 SV=1 |
| sp\|Q9ULI1\|NWD2_HUMAN | cyto | NACHT and WD repeat domain-containing protein 2 OS=Homo sapiens OX=9606 GN=NWD2 PE=2 SV=3 |
| sp\|P61803\|DAD1_HUMAN | plas | Dolichyl-diphosphooligosaccharide--protein glycosyltransferase subunit DAD1 OS=Homo sapiens OX=9606 GN=DAD1 PE=1 SV=3 |
| sp\|Q8IYB3\|SRRM1_HUMAN | nucl | Serine/arginine repetitive matrix protein 1 OS=Homo sapiens OX=9606 GN=SRRM1 PE=1 SV=2 |
| sp\|P00973\|OAS1_HUMAN | cyto | 2'-5'-oligoadenylate synthase 1 OS=Homo sapiens OX=9606 GN=OAS1 PE=1 SV=4 |
| sp\|Q12888\|TP53B_HUMAN | nucl | TP53-binding protein 1 OS=Homo sapiens OX=9606 GN=TP53BP1 PE=1 SV=2 |
| sp\|Q9NVD7\|PARVA_HUMAN | cyto_nucl | Alpha-parvin OS=Homo sapiens OX=9606 GN=PARVA PE=1 SV=1 |
| sp\|O14896\|IRF6_HUMAN | mito | Interferon regulatory factor 6 OS=Homo sapiens OX=9606 GN=IRF6 PE=1 SV=1 |
| sp\|P20742\|PZP_HUMAN | extr | Pregnancy zone protein OS=Homo sapiens OX=9606 GN=PZP PE=1 SV=4 |
| sp\|Q9NPA8\|ENY2_HUMAN | cyto | Transcription and mRNA export factor ENY2 OS=Homo sapiens OX=9606 GN=ENY2 PE=1 SV=1 |
| sp\|Q5JTB6\|PLAC9_HUMAN | extr | Placenta-specific protein 9 OS=Homo sapiens OX=9606 GN=PLAC9 PE=1 SV=1 |
| sp\|Q9H694\|BICC1_HUMAN | nucl | Protein bicaudal C homolog 1 OS=Homo sapiens OX=9606 GN=BICC1 PE=1 SV=2 |
| sp\|Q8TCT9\|HM13_HUMAN | plas | Minor histocompatibility antigen H13 OS=Homo sapiens OX=9606 GN=HM13 PE=1 SV=1 |
| sp\|Q7Z4I7\|LIMS2_HUMAN | nucl | LIM and senescent cell antigen-like-containing domain protein 2 OS=Homo sapiens OX=9606 GN=LIMS2 PE=1 SV=1 |
| sp\|P07384\|CAN1_HUMAN | nucl | Calpain-1 catalytic subunit OS=Homo sapiens OX=9606 GN=CAPN1 PE=1 SV=1 |
| sp\|O15427\|MOT4_HUMAN | plas | Monocarboxylate transporter 4 OS=Homo sapiens OX=9606 GN=SLC16A3 PE=1 SV=1 |
| sp\|Q9H4L4\|SENP3_HUMAN | cyto_nucl | Sentrin-specific protease 3 OS=Homo sapiens OX=9606 GN=SENP3 PE=1 SV=2 |
| sp\|Q9Y5U9\|IR3IP_HUMAN | plas | Immediate early response 3-interacting protein 1 OS=Homo sapiens OX=9606 GN=IER3IP1 PE=1 SV=1 |
| sp\|Q8IUH4\|ZDH13_HUMAN | plas | Palmitoyltransferase ZDHHC13 OS=Homo sapiens OX=9606 GN=ZDHHC13 PE=1 SV=3 |
| sp\|O14828\|SCAM3_HUMAN | plas | Secretory carrier-associated membrane protein 3 OS=Homo sapiens OX=9606 GN=SCAMP3 PE=1 SV=3 |
| sp\|Q9GZV4\|IF5A2_HUMAN | cyto | Eukaryotic translation initiation factor 5A-2 OS=Homo sapiens OX=9606 GN=EIF5A2 PE=1 SV=3 |
| sp\|Q7L3T8\|SYPM_HUMAN | mito | Probable proline--tRNA ligase, mitochondrial OS=Homo sapiens OX=9606 GN=PARS2 PE=1 SV=1 |
| sp\|Q9HCG8\|CWC22_HUMAN | nucl | Pre-mRNA-splicing factor CWC22 homolog OS=Homo sapiens OX=9606 GN=CWC22 PE=1 SV=3 |
| sp\|P11413\|G6PD_HUMAN | cyto | Glucose-6-phosphate 1-dehydrogenase OS=Homo sapiens OX=9606 GN=G6PD PE=1 SV=4 |
| sp\|Q92979\|NEP1_HUMAN | nucl | Ribosomal RNA small subunit methyltransferase NEP1 OS=Homo sapiens OX=9606 GN=EMG1 PE=1 SV=4 |
| sp\|Q96QS6\|KPSH2_HUMAN | cyto | Serine/threonine-protein kinase H2 OS=Homo sapiens OX=9606 GN=PSKH2 PE=2 SV=1 |
| sp\|P01705\|LV223_HUMAN | extr | Immunoglobulin lambda variable 2-23 OS=Homo sapiens OX=9606 GN=IGLV2-23 PE=1 SV=2 |
| sp\|Q00325\|MPCP_HUMAN | nucl | Phosphate carrier protein, mitochondrial OS=Homo sapiens OX=9606 GN=SLC25A3 PE=1 SV=2 |
| sp\|Q8N130\|NPT2C_HUMAN | plas | Sodium-dependent phosphate transport protein 2C OS=Homo sapiens OX=9606 GN=SLC34A3 PE=1 SV=2 |
| sp\|Q00688\|FKBP3_HUMAN | cyto | Peptidyl-prolyl cis-trans isomerase FKBP3 OS=Homo sapiens OX=9606 GN=FKBP3 PE=1 SV=1 |
| sp\|P62487\|RPB7_HUMAN | cysk | DNA-directed RNA polymerase II subunit RPB7 OS=Homo sapiens OX=9606 GN=POLR2G PE=1 SV=1 |
| sp\|P10620\|MGST1_HUMAN | cyto_nucl | Microsomal glutathione S-transferase 1 OS=Homo sapiens OX=9606 GN=MGST1 PE=1 SV=1 |
| sp\|Q9NYL9\|TMOD3_HUMAN | cyto | Tropomodulin-3 OS=Homo sapiens OX=9606 GN=TMOD3 PE=1 SV=1 |
| sp\|Q96IX5\|USMG5_HUMAN | cyto | Up-regulated during skeletal muscle growth protein 5 OS=Homo sapiens OX=9606 GN=ATP5MD PE=1 SV=1 |
| sp\|Q9P2E9\|RRBP1_HUMAN | E.R. | Ribosome-binding protein 1 OS=Homo sapiens OX=9606 GN=RRBP1 PE=1 SV=5 |
| sp\|Q8IXM6\|NRM_HUMAN | plas | Nurim OS=Homo sapiens OX=9606 GN=NRM PE=1 SV=1 |
| sp\|O00148\|DX39A_HUMAN | cyto | ATP-dependent RNA helicase DDX39A OS=Homo sapiens OX=9606 GN=DDX39A PE=1 SV=2 |
| sp\|P31949\|S10AB_HUMAN | cyto | Protein S100-A11 OS=Homo sapiens OX=9606 GN=S100A11 PE=1 SV=2 |
| sp\|Q9GZP9\|DERL2_HUMAN | plas | Derlin-2 OS=Homo sapiens OX=9606 GN=DERL2 PE=1 SV=1 |
| sp\|Q9UDW1\|QCR9_HUMAN | mito | Cytochrome b-c1 complex subunit 9 OS=Homo sapiens OX=9606 GN=UQCR10 PE=1 SV=3 |
| sp\|Q13148\|TADBP_HUMAN | nucl | TAR DNA-binding protein 43 OS=Homo sapiens OX=9606 GN=TARDBP PE=1 SV=1 |
| sp\|P42704\|LPPRC_HUMAN | mito | Leucine-rich PPR motif-containing protein, mitochondrial OS=Homo sapiens OX=9606 GN=LRPPRC PE=1 SV=3 |
| sp\|P23381\|SYWC_HUMAN | cyto | Tryptophan--tRNA ligase, cytoplasmic OS=Homo sapiens OX=9606 GN=WARS PE=1 SV=2 |
| sp\|Q969M3\|YIPF5_HUMAN | plas | Protein YIPF5 OS=Homo sapiens OX=9606 GN=YIPF5 PE=1 SV=1 |
| sp\|Q96CW5\|GCP3_HUMAN | nucl | Gamma-tubulin complex component 3 OS=Homo sapiens OX=9606 GN=TUBGCP3 PE=1 SV=2 |
| sp\|Q16695\|H31T_HUMAN | nucl | Histone H3.1t OS=Homo sapiens OX=9606 GN=HIST3H3 PE=1 SV=3 |
| sp\|P04844\|RPN2_HUMAN | extr | Dolichyl-diphosphooligosaccharide--protein glycosyltransferase subunit 2 OS=Homo sapiens OX=9606 GN=RPN2 PE=1 SV=3 |
| sp\|Q99487\|PAFA2_HUMAN | mito | Platelet-activating factor acetylhydrolase 2, cytoplasmic OS=Homo sapiens OX=9606 GN=PAFAH2 PE=1 SV=1 |
| sp\|Q96CV9\|OPTN_HUMAN | cyto | Optineurin OS=Homo sapiens OX=9606 GN=OPTN PE=1 SV=3 |
| sp\|P40424\|PBX1_HUMAN | nucl | Pre-B-cell leukemia transcription factor 1 OS=Homo sapiens OX=9606 GN=PBX1 PE=1 SV=1 |
| sp\|P02675\|FIBB_HUMAN | extr | Fibrinogen beta chain OS=Homo sapiens OX=9606 GN=FGB PE=1 SV=2 |
| sp\|Q96HE7\|ERO1A_HUMAN | extr | ERO1-like protein alpha OS=Homo sapiens OX=9606 GN=ERO1A PE=1 SV=2 |
| sp\|Q9H9F9\|ARP5_HUMAN | nucl | Actin-related protein 5 OS=Homo sapiens OX=9606 GN=ACTR5 PE=1 SV=2 |
| sp\|P56134\|ATPK_HUMAN | cyto | ATP synthase subunit f, mitochondrial OS=Homo sapiens OX=9606 GN=ATP5MF PE=1 SV=3 |
| sp\|Q13480\|GAB1_HUMAN | nucl | GRB2-associated-binding protein 1 OS=Homo sapiens OX=9606 GN=GAB1 PE=1 SV=2 |
| sp\|Q99757\|THIOM_HUMAN | mito | Thioredoxin, mitochondrial OS=Homo sapiens OX=9606 GN=TXN2 PE=1 SV=2 |
| sp\|Q7L1Q6\|BZW1_HUMAN | nucl | Basic leucine zipper and W2 domain-containing protein 1 OS=Homo sapiens OX=9606 GN=BZW1 PE=1 SV=1 |
| sp\|Q8WVE0\|EFMT1_HUMAN | cyto | EEF1A lysine methyltransferase 1 OS=Homo sapiens OX=9606 GN=EEF1AKMT1 PE=1 SV=1 |
| sp\|Q15437\|SC23B_HUMAN | cyto_nucl | Protein transport protein Sec23B OS=Homo sapiens OX=9606 GN=SEC23B PE=1 SV=2 |
| sp\|Q9H3U1\|UN45A_HUMAN | cyto | Protein unc-45 homolog A OS=Homo sapiens OX=9606 GN=UNC45A PE=1 SV=1 |
| sp\|Q15363\|TMED2_HUMAN | extr | Transmembrane emp24 domain-containing protein 2 OS=Homo sapiens OX=9606 GN=TMED2 PE=1 SV=1 |
| sp\|E9PRG8\|CK098_HUMAN | nucl | Uncharacterized protein C11orf98 OS=Homo sapiens OX=9606 GN=C11orf98 PE=4 SV=1 |
| sp\|Q7L5N7\|PCAT2_HUMAN | mito | Lysophosphatidylcholine acyltransferase 2 OS=Homo sapiens OX=9606 GN=LPCAT2 PE=1 SV=1 |
| sp\|O00462\|MANBA_HUMAN | extr | Beta-mannosidase OS=Homo sapiens OX=9606 GN=MANBA PE=2 SV=3 |
| sp\|P25788\|PSA3_HUMAN | cyto | Proteasome subunit alpha type-3 OS=Homo sapiens OX=9606 GN=PSMA3 PE=1 SV=2 |
| sp\|Q6UW02\|CP20A_HUMAN | E.R. | Cytochrome P450 20A1 OS=Homo sapiens OX=9606 GN=CYP20A1 PE=1 SV=1 |
| sp\|P07108\|ACBP_HUMAN | cyto | Acyl-CoA-binding protein OS=Homo sapiens OX=9606 GN=DBI PE=1 SV=2 |
| sp\|Q6UXH1\|CREL2_HUMAN | extr | Cysteine-rich with EGF-like domain protein 2 OS=Homo sapiens OX=9606 GN=CRELD2 PE=1 SV=1 |
| sp\|Q8N511\|TM199_HUMAN | plas | Transmembrane protein 199 OS=Homo sapiens OX=9606 GN=TMEM199 PE=1 SV=1 |
| sp\|P60903\|S10AA_HUMAN | cyto | Protein S100-A10 OS=Homo sapiens OX=9606 GN=S100A10 PE=1 SV=2 |
| sp\|Q96PQ0\|SORC2_HUMAN | plas | VPS10 domain-containing receptor SorCS2 OS=Homo sapiens OX=9606 GN=SORCS2 PE=1 SV=3 |
| sp\|Q96DC8\|ECHD3_HUMAN | mito | Enoyl-CoA hydratase domain-containing protein 3, mitochondrial OS=Homo sapiens OX=9606 GN=ECHDC3 PE=1 SV=2 |
| sp\|Q9Y4P3\|TBL2_HUMAN | extr | Transducin beta-like protein 2 OS=Homo sapiens OX=9606 GN=TBL2 PE=1 SV=1 |
| sp\|P62857\|RS28_HUMAN | mito | 40S ribosomal protein S28 OS=Homo sapiens OX=9606 GN=RPS28 PE=1 SV=1 |
| sp\|Q9Y2T2\|AP3M1_HUMAN | cyto | AP-3 complex subunit mu-1 OS=Homo sapiens OX=9606 GN=AP3M1 PE=1 SV=1 |
| sp\|Q9Y6D5\|BIG2_HUMAN | cyto | Brefeldin A-inhibited guanine nucleotide-exchange protein 2 OS=Homo sapiens OX=9606 GN=ARFGEF2 PE=1 SV=3 |
| sp\|Q9H074\|PAIP1_HUMAN | cyto_nucl | Polyadenylate-binding protein-interacting protein 1 OS=Homo sapiens OX=9606 GN=PAIP1 PE=1 SV=1 |
| sp\|O75503\|CLN5_HUMAN | cyto | Ceroid-lipofuscinosis neuronal protein 5 OS=Homo sapiens OX=9606 GN=CLN5 PE=1 SV=2 |
| sp\|P16152\|CBR1_HUMAN | cyto | Carbonyl reductase [NADPH] 1 OS=Homo sapiens OX=9606 GN=CBR1 PE=1 SV=3 |
| sp\|Q96K37\|S35E1_HUMAN | plas | Solute carrier family 35 member E1 OS=Homo sapiens OX=9606 GN=SLC35E1 PE=1 SV=2 |
| sp\|Q3KQZ1\|S2535_HUMAN | extr | Solute carrier family 25 member 35 OS=Homo sapiens OX=9606 GN=SLC25A35 PE=2 SV=1 |
| sp\|Q96DM3\|RMC1_HUMAN | cyto | Regulator of MON1-CCZ1 complex OS=Homo sapiens OX=9606 GN=RMC1 PE=1 SV=2 |
| sp\|Q15172\|2A5A_HUMAN | cyto | Serine/threonine-protein phosphatase 2A 56 kDa regulatory subunit alpha isoform OS=Homo sapiens OX=9606 GN=PPP2R5A PE=1 SV=1 |
| sp\|Q9Y2B2\|PIGL_HUMAN | extr | N-acetylglucosaminyl-phosphatidylinositol de-N-acetylase OS=Homo sapiens OX=9606 GN=PIGL PE=1 SV=1 |
| sp\|Q8TAC1\|RFESD_HUMAN | cyto | Rieske domain-containing protein OS=Homo sapiens OX=9606 GN=RFESD PE=1 SV=1 |
| sp\|O75828\|CBR3_HUMAN | mito | Carbonyl reductase [NADPH] 3 OS=Homo sapiens OX=9606 GN=CBR3 PE=1 SV=3 |
| sp\|Q96AY3\|FKB10_HUMAN | extr | Peptidyl-prolyl cis-trans isomerase FKBP10 OS=Homo sapiens OX=9606 GN=FKBP10 PE=1 SV=1 |
| sp\|P05451\|REG1A_HUMAN | extr | Lithostathine-1-alpha OS=Homo sapiens OX=9606 GN=REG1A PE=1 SV=3 |
| sp\|P61278\|SMS_HUMAN | extr | Somatostatin OS=Homo sapiens OX=9606 GN=SST PE=1 SV=1 |
| sp\|P15927\|RFA2_HUMAN | cyto | Replication protein A 32 kDa subunit OS=Homo sapiens OX=9606 GN=RPA2 PE=1 SV=1 |
| sp\|Q9NX57\|RAB20_HUMAN | cyto | Ras-related protein Rab-20 OS=Homo sapiens OX=9606 GN=RAB20 PE=1 SV=1 |
| sp\|Q9NRB3\|CHSTC_HUMAN | golg | Carbohydrate sulfotransferase 12 OS=Homo sapiens OX=9606 GN=CHST12 PE=2 SV=2 |
| sp\|Q2T9J0\|TYSD1_HUMAN | mito | Peroxisomal leader peptide-processing protease OS=Homo sapiens OX=9606 GN=TYSND1 PE=1 SV=3 |
| sp\|O75718\|CRTAP_HUMAN | extr | Cartilage-associated protein OS=Homo sapiens OX=9606 GN=CRTAP PE=1 SV=1 |
| sp\|Q12996\|CSTF3_HUMAN | cyto | Cleavage stimulation factor subunit 3 OS=Homo sapiens OX=9606 GN=CSTF3 PE=1 SV=1 |
| sp\|Q14257\|RCN2_HUMAN | extr | Reticulocalbin-2 OS=Homo sapiens OX=9606 GN=RCN2 PE=1 SV=1 |
| sp\|Q8NEZ5\|FBX22_HUMAN | cyto | F-box only protein 22 OS=Homo sapiens OX=9606 GN=FBXO22 PE=1 SV=1 |
| sp\|Q9BW27\|NUP85_HUMAN | cyto | Nuclear pore complex protein Nup85 OS=Homo sapiens OX=9606 GN=NUP85 PE=1 SV=1 |
| sp\|P23396\|RS3_HUMAN | cyto | 40S ribosomal protein S3 OS=Homo sapiens OX=9606 GN=RPS3 PE=1 SV=2 |
| sp\|O14980\|XPO1_HUMAN | cyto | Exportin-1 OS=Homo sapiens OX=9606 GN=XPO1 PE=1 SV=1 |
| sp\|Q96D53\|COQ8B_HUMAN | nucl | Atypical kinase COQ8B, mitochondrial OS=Homo sapiens OX=9606 GN=COQ8B PE=1 SV=2 |
| sp\|Q8WU67\|ABHD3_HUMAN | mito | Phospholipase ABHD3 OS=Homo sapiens OX=9606 GN=ABHD3 PE=1 SV=2 |
| sp\|P55008\|AIF1_HUMAN | cyto | Allograft inflammatory factor 1 OS=Homo sapiens OX=9606 GN=AIF1 PE=1 SV=1 |
| sp\|Q09666\|AHNK_HUMAN | nucl | Neuroblast differentiation-associated protein AHNAK OS=Homo sapiens OX=9606 GN=AHNAK PE=1 SV=2 |
| sp\|Q8N9V3\|WSDU1_HUMAN | extr | WD repeat, SAM and U-box domain-containing protein 1 OS=Homo sapiens OX=9606 GN=WDSUB1 PE=1 SV=3 |
| sp\|P61326\|MGN_HUMAN | cyto | Protein mago nashi homolog OS=Homo sapiens OX=9606 GN=MAGOH PE=1 SV=1 |
| sp\|Q6PCE3\|PGM2L_HUMAN | cyto | Glucose 1,6-bisphosphate synthase OS=Homo sapiens OX=9606 GN=PGM2L1 PE=1 SV=3 |
| sp\|Q8WWM9\|CYGB_HUMAN | cyto | Cytoglobin OS=Homo sapiens OX=9606 GN=CYGB PE=1 SV=1 |
| sp\|Q2TAA2\|IAH1_HUMAN | extr | Isoamyl acetate-hydrolyzing esterase 1 homolog OS=Homo sapiens OX=9606 GN=IAH1 PE=1 SV=1 |
| sp\|Q8WUD6\|CHPT1_HUMAN | plas | Cholinephosphotransferase 1 OS=Homo sapiens OX=9606 GN=CHPT1 PE=1 SV=1 |
| sp\|P08138\|TNR16_HUMAN | extr | Tumor necrosis factor receptor superfamily member 16 OS=Homo sapiens OX=9606 GN=NGFR PE=1 SV=1 |
| sp\|Q16585\|SGCB_HUMAN | plas | Beta-sarcoglycan OS=Homo sapiens OX=9606 GN=SGCB PE=1 SV=1 |
| sp\|Q15050\|RRS1_HUMAN | nucl | Ribosome biogenesis regulatory protein homolog OS=Homo sapiens OX=9606 GN=RRS1 PE=1 SV=2 |
| sp\|Q13144\|EI2BE_HUMAN | nucl | Translation initiation factor eIF-2B subunit epsilon OS=Homo sapiens OX=9606 GN=EIF2B5 PE=1 SV=3 |
| sp\|P29992\|GNA11_HUMAN | cyto | Guanine nucleotide-binding protein subunit alpha-11 OS=Homo sapiens OX=9606 GN=GNA11 PE=1 SV=2 |
| sp\|Q9Y2G3\|AT11B_HUMAN | plas | Probable phospholipid-transporting ATPase IF OS=Homo sapiens OX=9606 GN=ATP11B PE=1 SV=2 |
| sp\|P61626\|LYSC_HUMAN | extr | Lysozyme C OS=Homo sapiens OX=9606 GN=LYZ PE=1 SV=1 |
| sp\|Q9NVV4\|PAPD1_HUMAN | mito | Poly(A) RNA polymerase, mitochondrial OS=Homo sapiens OX=9606 GN=MTPAP PE=1 SV=1 |
| sp\|P78559\|MAP1A_HUMAN | nucl | Microtubule-associated protein 1A OS=Homo sapiens OX=9606 GN=MAP1A PE=1 SV=6 |
| sp\|Q8NG11\|TSN14_HUMAN | plas | Tetraspanin-14 OS=Homo sapiens OX=9606 GN=TSPAN14 PE=1 SV=1 |
| sp\|Q9NQG6\|MID51_HUMAN | cyto | Mitochondrial dynamics protein MID51 OS=Homo sapiens OX=9606 GN=MIEF1 PE=1 SV=1 |
| sp\|Q96S99\|PKHF1_HUMAN | cyto_nucl | Pleckstrin homology domain-containing family F member 1 OS=Homo sapiens OX=9606 GN=PLEKHF1 PE=1 SV=3 |
| sp\|Q6UXY8\|TMC5_HUMAN | plas | Transmembrane channel-like protein 5 OS=Homo sapiens OX=9606 GN=TMC5 PE=2 SV=3 |
| sp\|P11678\|PERE_HUMAN | extr | Eosinophil peroxidase OS=Homo sapiens OX=9606 GN=EPX PE=1 SV=2 |
| sp\|Q8TDB4\|HUMMR_HUMAN | mito | Protein MGARP OS=Homo sapiens OX=9606 GN=MGARP PE=1 SV=1 |
| sp\|P45381\|ACY2_HUMAN | cyto | Aspartoacylase OS=Homo sapiens OX=9606 GN=ASPA PE=1 SV=1 |
| sp\|Q9NP58\|ABCB6_HUMAN | plas | ATP-binding cassette sub-family B member 6, mitochondrial OS=Homo sapiens OX=9606 GN=ABCB6 PE=1 SV=1 |
| sp\|P13501\|CCL5_HUMAN | extr | C-C motif chemokine 5 OS=Homo sapiens OX=9606 GN=CCL5 PE=1 SV=3 |
| sp\|Q9NXN4\|GDAP2_HUMAN | cyto | Ganglioside-induced differentiation-associated protein 2 OS=Homo sapiens OX=9606 GN=GDAP2 PE=1 SV=1 |
| sp\|P03905\|NU4M_HUMAN | plas | NADH-ubiquinone oxidoreductase chain 4 OS=Homo sapiens OX=9606 GN=MT-ND4 PE=1 SV=1 |
| sp\|P20336\|RAB3A_HUMAN | cysk | Ras-related protein Rab-3A OS=Homo sapiens OX=9606 GN=RAB3A PE=1 SV=1 |
| sp\|Q9H583\|HEAT1_HUMAN | plas | HEAT repeat-containing protein 1 OS=Homo sapiens OX=9606 GN=HEATR1 PE=1 SV=3 |
| sp\|Q9NZ08\|ERAP1_HUMAN | extr | Endoplasmic reticulum aminopeptidase 1 OS=Homo sapiens OX=9606 GN=ERAP1 PE=1 SV=3 |
| sp\|Q9H479\|FN3K_HUMAN | nucl | Fructosamine-3-kinase OS=Homo sapiens OX=9606 GN=FN3K PE=1 SV=1 |
| sp\|P14060\|3BHS1_HUMAN | plas | 3 beta-hydroxysteroid dehydrogenase/Delta 5-->4-isomerase type 1 OS=Homo sapiens OX=9606 GN=HSD3B1 PE=1 SV=2 |
| sp\|Q9BVK2\|ALG8_HUMAN | plas | Probable dolichyl pyrophosphate Glc1Man9GlcNAc2 alpha-1,3-glucosyltransferase OS=Homo sapiens OX=9606 GN=ALG8 PE=1 SV=2 |
| sp\|P08263\|GSTA1_HUMAN | cyto | Glutathione S-transferase A1 OS=Homo sapiens OX=9606 GN=GSTA1 PE=1 SV=3 |
| sp\|Q9HCU8\|DPOD4_HUMAN | nucl | DNA polymerase delta subunit 4 OS=Homo sapiens OX=9606 GN=POLD4 PE=1 SV=1 |
| sp\|Q14642\|I5P1_HUMAN | extr | Type I inositol 1,4,5-trisphosphate 5-phosphatase OS=Homo sapiens OX=9606 GN=INPP5A PE=1 SV=1 |
| sp\|A0A0G2JS06\|LV539_HUMAN | extr | Immunoglobulin lambda variable 5-39 OS=Homo sapiens OX=9606 GN=IGLV5-39 PE=3 SV=1 |
| sp\|Q96EK7\|F120B_HUMAN | plas | Constitutive coactivator of peroxisome proliferator-activated receptor gamma OS=Homo sapiens OX=9606 GN=FAM120B PE=1 SV=1 |
| sp\|Q9NQ92\|COPRS_HUMAN | cyto_nucl | Coordinator of PRMT5 and differentiation stimulator OS=Homo sapiens OX=9606 GN=COPRS PE=1 SV=3 |
| sp\|P54760\|EPHB4_HUMAN | extr | Ephrin type-B receptor 4 OS=Homo sapiens OX=9606 GN=EPHB4 PE=1 SV=2 |
| sp\|Q86SZ2\|TPC6B_HUMAN | cyto | Trafficking protein particle complex subunit 6B OS=Homo sapiens OX=9606 GN=TRAPPC6B PE=1 SV=1 |
| sp\|O95236\|APOL3_HUMAN | cyto | Apolipoprotein L3 OS=Homo sapiens OX=9606 GN=APOL3 PE=1 SV=3 |
| sp\|Q9UNX4\|WDR3_HUMAN | cyto | WD repeat-containing protein 3 OS=Homo sapiens OX=9606 GN=WDR3 PE=1 SV=1 |
| sp\|P10412\|H14_HUMAN | nucl | Histone H1.4 OS=Homo sapiens OX=9606 GN=HIST1H1E PE=1 SV=2 |
| sp\|Q99808\|S29A1_HUMAN | plas | Equilibrative nucleoside transporter 1 OS=Homo sapiens OX=9606 GN=SLC29A1 PE=1 SV=3 |
| sp\|P04066\|FUCO_HUMAN | extr | Tissue alpha-L-fucosidase OS=Homo sapiens OX=9606 GN=FUCA1 PE=1 SV=4 |
| sp\|Q12999\|TSN31_HUMAN | plas | Tetraspanin-31 OS=Homo sapiens OX=9606 GN=TSPAN31 PE=2 SV=1 |
| sp\|P20020\|AT2B1_HUMAN | plas | Plasma membrane calcium-transporting ATPase 1 OS=Homo sapiens OX=9606 GN=ATP2B1 PE=1 SV=4 |
| sp\|Q8NCS4\|TM35B_HUMAN | plas | Transmembrane protein 35B OS=Homo sapiens OX=9606 GN=TMEM35B PE=1 SV=1 |
| sp\|Q9P0V3\|SH3B4_HUMAN | nucl | SH3 domain-binding protein 4 OS=Homo sapiens OX=9606 GN=SH3BP4 PE=1 SV=1 |
| sp\|P17096\|HMGA1_HUMAN | nucl | High mobility group protein HMG-I/HMG-Y OS=Homo sapiens OX=9606 GN=HMGA1 PE=1 SV=3 |
| sp\|P36871\|PGM1_HUMAN | cyto | Phosphoglucomutase-1 OS=Homo sapiens OX=9606 GN=PGM1 PE=1 SV=3 |
| sp\|Q5VST9\|OBSCN_HUMAN | cyto | Obscurin OS=Homo sapiens OX=9606 GN=OBSCN PE=1 SV=3 |
| sp\|P62068\|UBP46_HUMAN | cyto | Ubiquitin carboxyl-terminal hydrolase 46 OS=Homo sapiens OX=9606 GN=USP46 PE=1 SV=1 |
| sp\|Q96HF1\|SFRP2_HUMAN | extr | Secreted frizzled-related protein 2 OS=Homo sapiens OX=9606 GN=SFRP2 PE=1 SV=2 |
| sp\|P27105\|STOM_HUMAN | cyto | Erythrocyte band 7 integral membrane protein OS=Homo sapiens OX=9606 GN=STOM PE=1 SV=3 |
| sp\|Q6NUQ4\|TM214_HUMAN | cyto | Transmembrane protein 214 OS=Homo sapiens OX=9606 GN=TMEM214 PE=1 SV=2 |
| sp\|Q9BXP2\|S12A9_HUMAN | plas | Solute carrier family 12 member 9 OS=Homo sapiens OX=9606 GN=SLC12A9 PE=1 SV=1 |
| sp\|P28288\|ABCD3_HUMAN | mito | ATP-binding cassette sub-family D member 3 OS=Homo sapiens OX=9606 GN=ABCD3 PE=1 SV=1 |
| sp\|O95994\|AGR2_HUMAN | extr | Anterior gradient protein 2 homolog OS=Homo sapiens OX=9606 GN=AGR2 PE=1 SV=1 |
| sp\|P21796\|VDAC1_HUMAN | cyto | Voltage-dependent anion-selective channel protein 1 OS=Homo sapiens OX=9606 GN=VDAC1 PE=1 SV=2 |
| sp\|Q86SQ7\|SDCG8_HUMAN | nucl | Serologically defined colon cancer antigen 8 OS=Homo sapiens OX=9606 GN=SDCCAG8 PE=1 SV=1 |
| sp\|Q9Y6R7\|FCGBP_HUMAN | extr | IgGFc-binding protein OS=Homo sapiens OX=9606 GN=FCGBP PE=1 SV=3 |
| sp\|P79483\|DRB3_HUMAN | extr | HLA class II histocompatibility antigen, DR beta 3 chain OS=Homo sapiens OX=9606 GN=HLA-DRB3 PE=1 SV=1 |
| sp\|Q6NXS1\|IPP2B_HUMAN | nucl | Protein phosphatase inhibitor 2 family member B OS=Homo sapiens OX=9606 GN=PPP1R2B PE=1 SV=2 |
| sp\|O94875\|SRBS2_HUMAN | nucl | Sorbin and SH3 domain-containing protein 2 OS=Homo sapiens OX=9606 GN=SORBS2 PE=1 SV=3 |
| sp\|Q7Z401\|MYCPP_HUMAN | cyto | C-myc promoter-binding protein OS=Homo sapiens OX=9606 GN=DENND4A PE=1 SV=2 |
| sp\|P01861\|IGHG4_HUMAN | mito | Immunoglobulin heavy constant gamma 4 OS=Homo sapiens OX=9606 GN=IGHG4 PE=1 SV=1 |
| sp\|Q9Y3A6\|TMED5_HUMAN | extr | Transmembrane emp24 domain-containing protein 5 OS=Homo sapiens OX=9606 GN=TMED5 PE=1 SV=1 |
| sp\|P35749\|MYH11_HUMAN | nucl | Myosin-11 OS=Homo sapiens OX=9606 GN=MYH11 PE=1 SV=3 |
| sp\|Q9P2K6\|KLH42_HUMAN | nucl | Kelch-like protein 42 OS=Homo sapiens OX=9606 GN=KLHL42 PE=1 SV=2 |
| sp\|Q8NBK3\|SUMF1_HUMAN | extr | Formylglycine-generating enzyme OS=Homo sapiens OX=9606 GN=SUMF1 PE=1 SV=3 |
| sp\|Q8N3Y1\|FBXW8_HUMAN | nucl | F-box/WD repeat-containing protein 8 OS=Homo sapiens OX=9606 GN=FBXW8 PE=1 SV=2 |
| sp\|Q02094\|RHAG_HUMAN | plas | Ammonium transporter Rh type A OS=Homo sapiens OX=9606 GN=RHAG PE=1 SV=2 |
| sp\|Q96PP8\|GBP5_HUMAN | cyto | Guanylate-binding protein 5 OS=Homo sapiens OX=9606 GN=GBP5 PE=1 SV=1 |
| sp\|P28906\|CD34_HUMAN | mito | Hematopoietic progenitor cell antigen CD34 OS=Homo sapiens OX=9606 GN=CD34 PE=1 SV=2 |
| sp\|O43813\|LANC1_HUMAN | cyto | Glutathione S-transferase LANCL1 OS=Homo sapiens OX=9606 GN=LANCL1 PE=1 SV=1 |
| sp\|Q15121\|PEA15_HUMAN | cyto | Astrocytic phosphoprotein PEA-15 OS=Homo sapiens OX=9606 GN=PEA15 PE=1 SV=2 |
| sp\|Q8WWC4\|MAIP1_HUMAN | mito | m-AAA protease-interacting protein 1, mitochondrial OS=Homo sapiens OX=9606 GN=MAIP1 PE=1 SV=1 |
| sp\|Q7Z7H5\|TMED4_HUMAN | extr | Transmembrane emp24 domain-containing protein 4 OS=Homo sapiens OX=9606 GN=TMED4 PE=1 SV=1 |
| sp\|O15484\|CAN5_HUMAN | cyto_nucl | Calpain-5 OS=Homo sapiens OX=9606 GN=CAPN5 PE=1 SV=2 |
| sp\|O15439\|MRP4_HUMAN | plas | Multidrug resistance-associated protein 4 OS=Homo sapiens OX=9606 GN=ABCC4 PE=1 SV=3 |
| sp\|Q8WY22\|BRI3B_HUMAN | extr | BRI3-binding protein OS=Homo sapiens OX=9606 GN=BRI3BP PE=1 SV=1 |
| sp\|O43852\|CALU_HUMAN | extr | Calumenin OS=Homo sapiens OX=9606 GN=CALU PE=1 SV=2 |
| sp\|Q9Y2Q0\|AT8A1_HUMAN | plas | Phospholipid-transporting ATPase IA OS=Homo sapiens OX=9606 GN=ATP8A1 PE=1 SV=1 |
| sp\|P35527\|K1C9_HUMAN | nucl | Keratin, type I cytoskeletal 9 OS=Homo sapiens OX=9606 GN=KRT9 PE=1 SV=3 |
| sp\|P0DJD8\|PEPA3_HUMAN | extr | Pepsin A-3 OS=Homo sapiens OX=9606 GN=PGA3 PE=1 SV=1 |
| sp\|P00403\|COX2_HUMAN | plas | Cytochrome c oxidase subunit 2 OS=Homo sapiens OX=9606 GN=MT-CO2 PE=1 SV=1 |
| sp\|Q86YZ3\|HORN_HUMAN | nucl | Hornerin OS=Homo sapiens OX=9606 GN=HRNR PE=1 SV=2 |
| sp\|Q969E2\|SCAM4_HUMAN | plas | Secretory carrier-associated membrane protein 4 OS=Homo sapiens OX=9606 GN=SCAMP4 PE=1 SV=1 |
| sp\|P51606\|RENBP_HUMAN | cyto | N-acylglucosamine 2-epimerase OS=Homo sapiens OX=9606 GN=RENBP PE=1 SV=2 |
| sp\|P52594\|AGFG1_HUMAN | nucl | Arf-GAP domain and FG repeat-containing protein 1 OS=Homo sapiens OX=9606 GN=AGFG1 PE=1 SV=2 |
| sp\|O00391\|QSOX1_HUMAN | golg | Sulfhydryl oxidase 1 OS=Homo sapiens OX=9606 GN=QSOX1 PE=1 SV=3 |
| sp\|Q9UBX3\|DIC_HUMAN | mito | Mitochondrial dicarboxylate carrier OS=Homo sapiens OX=9606 GN=SLC25A10 PE=1 SV=2 |
| sp\|Q8WTT2\|NOC3L_HUMAN | nucl | Nucleolar complex protein 3 homolog OS=Homo sapiens OX=9606 GN=NOC3L PE=1 SV=1 |
| sp\|P0C7T5\|ATX1L_HUMAN | nucl | Ataxin-1-like OS=Homo sapiens OX=9606 GN=ATXN1L PE=1 SV=1 |
| sp\|Q9NUS5\|AP5S1_HUMAN | mito | AP-5 complex subunit sigma-1 OS=Homo sapiens OX=9606 GN=AP5S1 PE=1 SV=1 |
| sp\|Q9UNA1\|RHG26_HUMAN | cyto | Rho GTPase-activating protein 26 OS=Homo sapiens OX=9606 GN=ARHGAP26 PE=1 SV=1 |
| sp\|P0CK96\|S352B_HUMAN | plas | Solute carrier family 35 member E2B OS=Homo sapiens OX=9606 GN=SLC35E2B PE=2 SV=1 |
| sp\|O43451\|MGA_HUMAN | golg | Maltase-glucoamylase, intestinal OS=Homo sapiens OX=9606 GN=MGAM PE=1 SV=5 |
| sp\|Q93008\|USP9X_HUMAN | plas | Probable ubiquitin carboxyl-terminal hydrolase FAF-X OS=Homo sapiens OX=9606 GN=USP9X PE=1 SV=3 |
| sp\|Q9UMX1\|SUFU_HUMAN | cyto | Suppressor of fused homolog OS=Homo sapiens OX=9606 GN=SUFU PE=1 SV=2 |
| sp\|P20340\|RAB6A_HUMAN | cyto | Ras-related protein Rab-6A OS=Homo sapiens OX=9606 GN=RAB6A PE=1 SV=3 |
| sp\|Q9Y2S6\|TMA7_HUMAN | nucl | Translation machinery-associated protein 7 OS=Homo sapiens OX=9606 GN=TMA7 PE=1 SV=1 |
| sp\|Q9Y2A7\|NCKP1_HUMAN | nucl | Nck-associated protein 1 OS=Homo sapiens OX=9606 GN=NCKAP1 PE=1 SV=1 |
| sp\|Q7Z3U7\|MON2_HUMAN | plas | Protein MON2 homolog OS=Homo sapiens OX=9606 GN=MON2 PE=1 SV=3 |
| sp\|Q9NP74\|PALMD_HUMAN | nucl | Palmdelphin OS=Homo sapiens OX=9606 GN=PALMD PE=1 SV=1 |
| sp\|Q03252\|LMNB2_HUMAN | nucl | Lamin-B2 OS=Homo sapiens OX=9606 GN=LMNB2 PE=1 SV=4 |
| sp\|P16233\|LIPP_HUMAN | extr | Pancreatic triacylglycerol lipase OS=Homo sapiens OX=9606 GN=PNLIP PE=1 SV=1 |
| sp\|Q9P2R3\|ANFY1_HUMAN | cyto | Rabankyrin-5 OS=Homo sapiens OX=9606 GN=ANKFY1 PE=1 SV=2 |
| sp\|Q16082\|HSPB2_HUMAN | cyto_nucl | Heat shock protein beta-2 OS=Homo sapiens OX=9606 GN=HSPB2 PE=1 SV=2 |
| sp\|Q9Y2D2\|S35A3_HUMAN | plas | UDP-N-acetylglucosamine transporter OS=Homo sapiens OX=9606 GN=SLC35A3 PE=1 SV=1 |
| sp\|P37268\|FDFT_HUMAN | cyto | Squalene synthase OS=Homo sapiens OX=9606 GN=FDFT1 PE=1 SV=1 |
| sp\|P51648\|AL3A2_HUMAN | cyto | Fatty aldehyde dehydrogenase OS=Homo sapiens OX=9606 GN=ALDH3A2 PE=1 SV=1 |
| sp\|Q8TCJ2\|STT3B_HUMAN | plas | Dolichyl-diphosphooligosaccharide--protein glycosyltransferase subunit STT3B OS=Homo sapiens OX=9606 GN=STT3B PE=1 SV=1 |
| sp\|P08670\|VIME_HUMAN | nucl | Vimentin OS=Homo sapiens OX=9606 GN=VIM PE=1 SV=4 |
| sp\|Q16890\|TPD53_HUMAN | cyto | Tumor protein D53 OS=Homo sapiens OX=9606 GN=TPD52L1 PE=1 SV=1 |
| sp\|P54709\|AT1B3_HUMAN | cyto | Sodium/potassium-transporting ATPase subunit beta-3 OS=Homo sapiens OX=9606 GN=ATP1B3 PE=1 SV=1 |
| sp\|Q9HCU0\|CD248_HUMAN | pero | Endosialin OS=Homo sapiens OX=9606 GN=CD248 PE=1 SV=1 |
| sp\|Q9NVZ3\|NECP2_HUMAN | cyto | Adaptin ear-binding coat-associated protein 2 OS=Homo sapiens OX=9606 GN=NECAP2 PE=1 SV=1 |
| sp\|Q13442\|HAP28_HUMAN | nucl | 28 kDa heat- and acid-stable phosphoprotein OS=Homo sapiens OX=9606 GN=PDAP1 PE=1 SV=1 |
| sp\|Q6PI78\|TMM65_HUMAN | plas | Transmembrane protein 65 OS=Homo sapiens OX=9606 GN=TMEM65 PE=1 SV=2 |
| sp\|P53618\|COPB_HUMAN | cysk | Coatomer subunit beta OS=Homo sapiens OX=9606 GN=COPB1 PE=1 SV=3 |
| sp\|Q15746\|MYLK_HUMAN | cyto | Myosin light chain kinase, smooth muscle OS=Homo sapiens OX=9606 GN=MYLK PE=1 SV=4 |
| sp\|Q8N2U0\|TM256_HUMAN | mito | Transmembrane protein 256 OS=Homo sapiens OX=9606 GN=TMEM256 PE=3 SV=1 |
| sp\|P04114\|APOB_HUMAN | E.R. | Apolipoprotein B-100 OS=Homo sapiens OX=9606 GN=APOB PE=1 SV=2 |
| sp\|O43399\|TPD54_HUMAN | cyto_nucl | Tumor protein D54 OS=Homo sapiens OX=9606 GN=TPD52L2 PE=1 SV=2 |
| sp\|Q15631\|TSN_HUMAN | cyto | Translin OS=Homo sapiens OX=9606 GN=TSN PE=1 SV=1 |
| sp\|Q9NWD8\|TM248_HUMAN | plas | Transmembrane protein 248 OS=Homo sapiens OX=9606 GN=TMEM248 PE=1 SV=1 |
| sp\|P08729\|K2C7_HUMAN | mito | Keratin, type II cytoskeletal 7 OS=Homo sapiens OX=9606 GN=KRT7 PE=1 SV=5 |
| sp\|Q96D15\|RCN3_HUMAN | extr | Reticulocalbin-3 OS=Homo sapiens OX=9606 GN=RCN3 PE=1 SV=1 |
| sp\|P31947\|1433S_HUMAN | cyto_nucl | 14-3-3 protein sigma OS=Homo sapiens OX=9606 GN=SFN PE=1 SV=1 |
| sp\|Q7L576\|CYFP1_HUMAN | cyto | Cytoplasmic FMR1-interacting protein 1 OS=Homo sapiens OX=9606 GN=CYFIP1 PE=1 SV=1 |
| sp\|Q13151\|ROA0_HUMAN | nucl | Heterogeneous nuclear ribonucleoprotein A0 OS=Homo sapiens OX=9606 GN=HNRNPA0 PE=1 SV=1 |
| sp\|O00442\|RTCA_HUMAN | E.R._mito | RNA 3'-terminal phosphate cyclase OS=Homo sapiens OX=9606 GN=RTCA PE=1 SV=1 |
| sp\|Q13813\|SPTN1_HUMAN | nucl | Spectrin alpha chain, non-erythrocytic 1 OS=Homo sapiens OX=9606 GN=SPTAN1 PE=1 SV=3 |
| sp\|Q96A19\|C102A_HUMAN | nucl | Coiled-coil domain-containing protein 102A OS=Homo sapiens OX=9606 GN=CCDC102A PE=1 SV=2 |
| sp\|Q5TCZ1\|SPD2A_HUMAN | nucl | SH3 and PX domain-containing protein 2A OS=Homo sapiens OX=9606 GN=SH3PXD2A PE=1 SV=1 |
| sp\|Q6P3X3\|TTC27_HUMAN | cyto | Tetratricopeptide repeat protein 27 OS=Homo sapiens OX=9606 GN=TTC27 PE=1 SV=1 |
| sp\|Q96NY7\|CLIC6_HUMAN | nucl | Chloride intracellular channel protein 6 OS=Homo sapiens OX=9606 GN=CLIC6 PE=2 SV=3 |
| sp\|P40616\|ARL1_HUMAN | extr | ADP-ribosylation factor-like protein 1 OS=Homo sapiens OX=9606 GN=ARL1 PE=1 SV=1 |
| sp\|Q9UHQ9\|NB5R1_HUMAN | extr | NADH-cytochrome b5 reductase 1 OS=Homo sapiens OX=9606 GN=CYB5R1 PE=1 SV=1 |
| sp\|Q14108\|SCRB2_HUMAN | E.R. | Lysosome membrane protein 2 OS=Homo sapiens OX=9606 GN=SCARB2 PE=1 SV=2 |
| sp\|P61586\|RHOA_HUMAN | cyto | Transforming protein RhoA OS=Homo sapiens OX=9606 GN=RHOA PE=1 SV=1 |
| sp\|Q92876\|KLK6_HUMAN | extr | Kallikrein-6 OS=Homo sapiens OX=9606 GN=KLK6 PE=1 SV=1 |
| sp\|Q9Y487\|VPP2_HUMAN | plas | V-type proton ATPase 116 kDa subunit a isoform 2 OS=Homo sapiens OX=9606 GN=ATP6V0A2 PE=1 SV=2 |
| sp\|Q8TAG9\|EXOC6_HUMAN | cyto_nucl | Exocyst complex component 6 OS=Homo sapiens OX=9606 GN=EXOC6 PE=1 SV=3 |
| sp\|Q8TBH0\|ARRD2_HUMAN | nucl | Arrestin domain-containing protein 2 OS=Homo sapiens OX=9606 GN=ARRDC2 PE=1 SV=2 |
| sp\|Q9HBH0\|RHOF_HUMAN | extr | Rho-related GTP-binding protein RhoF OS=Homo sapiens OX=9606 GN=RHOF PE=1 SV=1 |
| sp\|Q3LXA3\|TKFC_HUMAN | extr | Triokinase/FMN cyclase OS=Homo sapiens OX=9606 GN=TKFC PE=1 SV=2 |
| sp\|Q96A57\|TM230_HUMAN | plas | Transmembrane protein 230 OS=Homo sapiens OX=9606 GN=TMEM230 PE=1 SV=1 |
| sp\|P61218\|RPAB2_HUMAN | cyto_nucl | DNA-directed RNA polymerases I, II, and III subunit RPABC2 OS=Homo sapiens OX=9606 GN=POLR2F PE=1 SV=1 |
| sp\|P62917\|RL8_HUMAN | cyto | 60S ribosomal protein L8 OS=Homo sapiens OX=9606 GN=RPL8 PE=1 SV=2 |
| sp\|P10321\|1C07_HUMAN | extr | HLA class I histocompatibility antigen, Cw-7 alpha chain OS=Homo sapiens OX=9606 GN=HLA-C PE=1 SV=3 |
| sp\|O75844\|FACE1_HUMAN | plas | CAAX prenyl protease 1 homolog OS=Homo sapiens OX=9606 GN=ZMPSTE24 PE=1 SV=2 |
| sp\|Q15125\|EBP_HUMAN | plas | 3-beta-hydroxysteroid-Delta(8),Delta(7)-isomerase OS=Homo sapiens OX=9606 GN=EBP PE=1 SV=3 |
| sp\|P08648\|ITA5_HUMAN | nucl | Integrin alpha-5 OS=Homo sapiens OX=9606 GN=ITGA5 PE=1 SV=2 |
| sp\|Q9GZM5\|YIPF3_HUMAN | plas | Protein YIPF3 OS=Homo sapiens OX=9606 GN=YIPF3 PE=1 SV=1 |
| sp\|Q12933\|TRAF2_HUMAN | nucl | TNF receptor-associated factor 2 OS=Homo sapiens OX=9606 GN=TRAF2 PE=1 SV=2 |
| sp\|P62070\|RRAS2_HUMAN | cyto | Ras-related protein R-Ras2 OS=Homo sapiens OX=9606 GN=RRAS2 PE=1 SV=1 |
| sp\|Q9BQI0\|AIF1L_HUMAN | cyto | Allograft inflammatory factor 1-like OS=Homo sapiens OX=9606 GN=AIF1L PE=1 SV=1 |
| sp\|P31327\|CPSM_HUMAN | mito | Carbamoyl-phosphate synthase [ammonia], mitochondrial OS=Homo sapiens OX=9606 GN=CPS1 PE=1 SV=2 |
| sp\|Q8TB36\|GDAP1_HUMAN | cyto | Ganglioside-induced differentiation-associated protein 1 OS=Homo sapiens OX=9606 GN=GDAP1 PE=1 SV=3 |
| sp\|Q9UBD5\|ORC3_HUMAN | nucl | Origin recognition complex subunit 3 OS=Homo sapiens OX=9606 GN=ORC3 PE=1 SV=1 |
| sp\|O14979\|HNRDL_HUMAN | nucl | Heterogeneous nuclear ribonucleoprotein D-like OS=Homo sapiens OX=9606 GN=HNRNPDL PE=1 SV=3 |
| sp\|Q96F07\|CYFP2_HUMAN | cyto | Cytoplasmic FMR1-interacting protein 2 OS=Homo sapiens OX=9606 GN=CYFIP2 PE=1 SV=2 |
| sp\|O15042\|SR140_HUMAN | nucl | U2 snRNP-associated SURP motif-containing protein OS=Homo sapiens OX=9606 GN=U2SURP PE=1 SV=2 |
| sp\|Q9Y394\|DHRS7_HUMAN | E.R. | Dehydrogenase/reductase SDR family member 7 OS=Homo sapiens OX=9606 GN=DHRS7 PE=1 SV=1 |
| sp\|Q99439\|CNN2_HUMAN | cyto | Calponin-2 OS=Homo sapiens OX=9606 GN=CNN2 PE=1 SV=4 |
| sp\|Q9H9A6\|LRC40_HUMAN | cyto | Leucine-rich repeat-containing protein 40 OS=Homo sapiens OX=9606 GN=LRRC40 PE=1 SV=1 |
| sp\|Q9BU40\|CRDL1_HUMAN | extr | Chordin-like protein 1 OS=Homo sapiens OX=9606 GN=CHRDL1 PE=1 SV=1 |
| sp\|E7EW31\|PROB1_HUMAN | mito | Proline-rich basic protein 1 OS=Homo sapiens OX=9606 GN=PROB1 PE=2 SV=2 |
| sp\|P16050\|LOX15_HUMAN | cyto | Arachidonate 15-lipoxygenase OS=Homo sapiens OX=9606 GN=ALOX15 PE=1 SV=3 |
| sp\|P21741\|MK_HUMAN | extr | Midkine OS=Homo sapiens OX=9606 GN=MDK PE=1 SV=1 |
| sp\|Q9BQ61\|TRIR_HUMAN | nucl | Telomerase RNA component interacting RNase OS=Homo sapiens OX=9606 GN=TRIR PE=1 SV=1 |
| sp\|O00232\|PSD12_HUMAN | cyto | 26S proteasome non-ATPase regulatory subunit 12 OS=Homo sapiens OX=9606 GN=PSMD12 PE=1 SV=3 |
| sp\|O75131\|CPNE3_HUMAN | extr | Copine-3 OS=Homo sapiens OX=9606 GN=CPNE3 PE=1 SV=1 |
| sp\|Q9BRP8\|PYM1_HUMAN | cyto | Partner of Y14 and mago OS=Homo sapiens OX=9606 GN=PYM1 PE=1 SV=1 |
| sp\|Q9NZD2\|GLTP_HUMAN | cyto | Glycolipid transfer protein OS=Homo sapiens OX=9606 GN=GLTP PE=1 SV=3 |
| sp\|Q3LI76\|KR151_HUMAN | extr | Keratin-associated protein 15-1 OS=Homo sapiens OX=9606 GN=KRTAP15-1 PE=1 SV=2 |
| sp\|Q15654\|TRIP6_HUMAN | nucl | Thyroid receptor-interacting protein 6 OS=Homo sapiens OX=9606 GN=TRIP6 PE=1 SV=3 |
| sp\|P16189\|1A31_HUMAN | extr | HLA class I histocompatibility antigen, A-31 alpha chain OS=Homo sapiens OX=9606 GN=HLA-A PE=1 SV=2 |
| sp\|Q96E17\|RAB3C_HUMAN | cyto | Ras-related protein Rab-3C OS=Homo sapiens OX=9606 GN=RAB3C PE=2 SV=1 |
| sp\|Q9H7F0\|AT133_HUMAN | plas | Probable cation-transporting ATPase 13A3 OS=Homo sapiens OX=9606 GN=ATP13A3 PE=1 SV=4 |
| sp\|Q9H6E5\|STPAP_HUMAN | nucl | Speckle targeted PIP5K1A-regulated poly(A) polymerase OS=Homo sapiens OX=9606 GN=TUT1 PE=1 SV=2 |
| sp\|Q5H9R7\|PP6R3_HUMAN | nucl | Serine/threonine-protein phosphatase 6 regulatory subunit 3 OS=Homo sapiens OX=9606 GN=PPP6R3 PE=1 SV=2 |
| sp\|Q7Z449\|CP2U1_HUMAN | plas | Cytochrome P450 2U1 OS=Homo sapiens OX=9606 GN=CYP2U1 PE=1 SV=1 |
| sp\|Q8NCL4\|GALT6_HUMAN | extr | Polypeptide N-acetylgalactosaminyltransferase 6 OS=Homo sapiens OX=9606 GN=GALNT6 PE=2 SV=2 |
| sp\|Q96B54\|ZN428_HUMAN | nucl | Zinc finger protein 428 OS=Homo sapiens OX=9606 GN=ZNF428 PE=1 SV=2 |
| sp\|Q6NY19\|KANK3_HUMAN | nucl | KN motif and ankyrin repeat domain-containing protein 3 OS=Homo sapiens OX=9606 GN=KANK3 PE=1 SV=1 |
| sp\|P37840\|SYUA_HUMAN | extr | Alpha-synuclein OS=Homo sapiens OX=9606 GN=SNCA PE=1 SV=1 |
| sp\|Q9UFH2\|DYH17_HUMAN | plas | Dynein heavy chain 17, axonemal OS=Homo sapiens OX=9606 GN=DNAH17 PE=1 SV=3 |
| sp\|Q9HD40\|SPCS_HUMAN | cyto | O-phosphoseryl-tRNA(Sec) selenium transferase OS=Homo sapiens OX=9606 GN=SEPSECS PE=1 SV=2 |
| sp\|Q9H269\|VPS16_HUMAN | nucl | Vacuolar protein sorting-associated protein 16 homolog OS=Homo sapiens OX=9606 GN=VPS16 PE=1 SV=2 |
| sp\|Q8NE01\|CNNM3_HUMAN | plas | Metal transporter CNNM3 OS=Homo sapiens OX=9606 GN=CNNM3 PE=1 SV=1 |
| sp\|P08247\|SYPH_HUMAN | plas | Synaptophysin OS=Homo sapiens OX=9606 GN=SYP PE=1 SV=3 |
| sp\|P56192\|SYMC_HUMAN | pero | Methionine--tRNA ligase, cytoplasmic OS=Homo sapiens OX=9606 GN=MARS PE=1 SV=2 |
| sp\|P61952\|GBG11_HUMAN | cyto | Guanine nucleotide-binding protein G(I)/G(S)/G(O) subunit gamma-11 OS=Homo sapiens OX=9606 GN=GNG11 PE=1 SV=1 |
| sp\|P08519\|APOA_HUMAN | extr | Apolipoprotein(a) OS=Homo sapiens OX=9606 GN=LPA PE=1 SV=1 |
| sp\|Q15043\|S39AE_HUMAN | plas | Zinc transporter ZIP14 OS=Homo sapiens OX=9606 GN=SLC39A14 PE=1 SV=3 |
| sp\|O00499\|BIN1_HUMAN | cyto | Myc box-dependent-interacting protein 1 OS=Homo sapiens OX=9606 GN=BIN1 PE=1 SV=1 |
| sp\|Q9NRN5\|OLFL3_HUMAN | extr | Olfactomedin-like protein 3 OS=Homo sapiens OX=9606 GN=OLFML3 PE=2 SV=1 |
| sp\|P45877\|PPIC_HUMAN | E.R. | Peptidyl-prolyl cis-trans isomerase C OS=Homo sapiens OX=9606 GN=PPIC PE=1 SV=1 |
| sp\|Q7Z3V4\|UBE3B_HUMAN | cyto | Ubiquitin-protein ligase E3B OS=Homo sapiens OX=9606 GN=UBE3B PE=1 SV=3 |
| sp\|Q96DG6\|CMBL_HUMAN | cyto | Carboxymethylenebutenolidase homolog OS=Homo sapiens OX=9606 GN=CMBL PE=1 SV=1 |
| sp\|Q6GMV2\|SMYD5_HUMAN | extr | SET and MYND domain-containing protein 5 OS=Homo sapiens OX=9606 GN=SMYD5 PE=1 SV=2 |
| sp\|P42285\|MTREX_HUMAN | cyto | Exosome RNA helicase MTR4 OS=Homo sapiens OX=9606 GN=MTREX PE=1 SV=3 |
| sp\|O95379\|TFIP8_HUMAN | cyto | Tumor necrosis factor alpha-induced protein 8 OS=Homo sapiens OX=9606 GN=TNFAIP8 PE=1 SV=1 |
| sp\|Q9H1I8\|ASCC2_HUMAN | cyto | Activating signal cointegrator 1 complex subunit 2 OS=Homo sapiens OX=9606 GN=ASCC2 PE=1 SV=3 |
| sp\|Q9UKU6\|TRHDE_HUMAN | plas | Thyrotropin-releasing hormone-degrading ectoenzyme OS=Homo sapiens OX=9606 GN=TRHDE PE=2 SV=1 |
| sp\|Q9UNQ2\|DIM1_HUMAN | cyto | Probable dimethyladenosine transferase OS=Homo sapiens OX=9606 GN=DIMT1 PE=1 SV=1 |
| sp\|Q7L5Y1\|ENOF1_HUMAN | mito | Mitochondrial enolase superfamily member 1 OS=Homo sapiens OX=9606 GN=ENOSF1 PE=1 SV=1 |
| sp\|Q06141\|REG3A_HUMAN | extr | Regenerating islet-derived protein 3-alpha OS=Homo sapiens OX=9606 GN=REG3A PE=1 SV=1 |
| sp\|Q13185\|CBX3_HUMAN | nucl | Chromobox protein homolog 3 OS=Homo sapiens OX=9606 GN=CBX3 PE=1 SV=4 |
| sp\|Q9P0S3\|ORML1_HUMAN | cyto | ORM1-like protein 1 OS=Homo sapiens OX=9606 GN=ORMDL1 PE=1 SV=1 |
| sp\|Q14457\|BECN1_HUMAN | cyto | Beclin-1 OS=Homo sapiens OX=9606 GN=BECN1 PE=1 SV=2 |
| sp\|Q5VYK3\|ECM29_HUMAN | nucl | Proteasome adapter and scaffold protein ECM29 OS=Homo sapiens OX=9606 GN=ECPAS PE=1 SV=2 |
| sp\|Q00013\|EM55_HUMAN | nucl | 55 kDa erythrocyte membrane protein OS=Homo sapiens OX=9606 GN=MPP1 PE=1 SV=2 |
| sp\|O75822\|EIF3J_HUMAN | nucl | Eukaryotic translation initiation factor 3 subunit J OS=Homo sapiens OX=9606 GN=EIF3J PE=1 SV=2 |
| sp\|P36955\|PEDF_HUMAN | plas | Pigment epithelium-derived factor OS=Homo sapiens OX=9606 GN=SERPINF1 PE=1 SV=4 |
| sp\|Q06210\|GFPT1_HUMAN | mito | Glutamine--fructose-6-phosphate aminotransferase [isomerizing] 1 OS=Homo sapiens OX=9606 GN=GFPT1 PE=1 SV=3 |
| sp\|Q8IW70\|T151B_HUMAN | plas | Transmembrane protein 151B OS=Homo sapiens OX=9606 GN=TMEM151B PE=2 SV=2 |
| sp\|Q8IWA4\|MFN1_HUMAN | mito | Mitofusin-1 OS=Homo sapiens OX=9606 GN=MFN1 PE=1 SV=3 |
| sp\|Q9HD45\|TM9S3_HUMAN | plas | Transmembrane 9 superfamily member 3 OS=Homo sapiens OX=9606 GN=TM9SF3 PE=1 SV=2 |
| sp\|Q9NUT2\|ABCB8_HUMAN | plas | ATP-binding cassette sub-family B member 8, mitochondrial OS=Homo sapiens OX=9606 GN=ABCB8 PE=1 SV=3 |
| sp\|Q15269\|PWP2_HUMAN | mito | Periodic tryptophan protein 2 homolog OS=Homo sapiens OX=9606 GN=PWP2 PE=2 SV=2 |
| sp\|O95573\|ACSL3_HUMAN | extr | Long-chain-fatty-acid--CoA ligase 3 OS=Homo sapiens OX=9606 GN=ACSL3 PE=1 SV=3 |
| sp\|Q8WZ82\|OVCA2_HUMAN | mito | Esterase OVCA2 OS=Homo sapiens OX=9606 GN=OVCA2 PE=1 SV=1 |
| sp\|Q16531\|DDB1_HUMAN | cyto | DNA damage-binding protein 1 OS=Homo sapiens OX=9606 GN=DDB1 PE=1 SV=1 |
| sp\|Q9UK76\|JUPI1_HUMAN | nucl | Jupiter microtubule associated homolog 1 OS=Homo sapiens OX=9606 GN=JPT1 PE=1 SV=3 |
| sp\|P26639\|SYTC_HUMAN | cyto | Threonine--tRNA ligase, cytoplasmic OS=Homo sapiens OX=9606 GN=TARS PE=1 SV=3 |
| sp\|Q15147\|PLCB4_HUMAN | nucl | 1-phosphatidylinositol 4,5-bisphosphate phosphodiesterase beta-4 OS=Homo sapiens OX=9606 GN=PLCB4 PE=1 SV=3 |
| sp\|P07099\|HYEP_HUMAN | E.R. | Epoxide hydrolase 1 OS=Homo sapiens OX=9606 GN=EPHX1 PE=1 SV=1 |
| sp\|P61006\|RAB8A_HUMAN | cyto | Ras-related protein Rab-8A OS=Homo sapiens OX=9606 GN=RAB8A PE=1 SV=1 |
| sp\|Q2M1P5\|KIF7_HUMAN | nucl | Kinesin-like protein KIF7 OS=Homo sapiens OX=9606 GN=KIF7 PE=1 SV=2 |
| sp\|Q8NEW0\|ZNT7_HUMAN | plas | Zinc transporter 7 OS=Homo sapiens OX=9606 GN=SLC30A7 PE=2 SV=1 |
| sp\|Q9Y6H3\|ATP23_HUMAN | extr | Mitochondrial inner membrane protease ATP23 homolog OS=Homo sapiens OX=9606 GN=ATP23 PE=1 SV=3 |
| sp\|Q8NHG7\|SVIP_HUMAN | mito | Small VCP/p97-interacting protein OS=Homo sapiens OX=9606 GN=SVIP PE=1 SV=1 |
| sp\|Q09428\|ABCC8_HUMAN | plas | ATP-binding cassette sub-family C member 8 OS=Homo sapiens OX=9606 GN=ABCC8 PE=1 SV=6 |
| sp\|Q9UPT8\|ZC3H4_HUMAN | nucl | Zinc finger CCCH domain-containing protein 4 OS=Homo sapiens OX=9606 GN=ZC3H4 PE=1 SV=3 |
| sp\|Q9H4P4\|RNF41_HUMAN | nucl | E3 ubiquitin-protein ligase NRDP1 OS=Homo sapiens OX=9606 GN=RNF41 PE=1 SV=2 |
| sp\|P30566\|PUR8_HUMAN | cyto | Adenylosuccinate lyase OS=Homo sapiens OX=9606 GN=ADSL PE=1 SV=2 |
| sp\|P08754\|GNAI3_HUMAN | cyto | Guanine nucleotide-binding protein G(k) subunit alpha OS=Homo sapiens OX=9606 GN=GNAI3 PE=1 SV=3 |
| sp\|O95716\|RAB3D_HUMAN | cyto | Ras-related protein Rab-3D OS=Homo sapiens OX=9606 GN=RAB3D PE=1 SV=1 |
| sp\|Q9NR19\|ACSA_HUMAN | cyto | Acetyl-coenzyme A synthetase, cytoplasmic OS=Homo sapiens OX=9606 GN=ACSS2 PE=1 SV=1 |
| sp\|Q9UHQ4\|BAP29_HUMAN | E.R. | B-cell receptor-associated protein 29 OS=Homo sapiens OX=9606 GN=BCAP29 PE=1 SV=2 |
| sp\|P15291\|B4GT1_HUMAN | mito | Beta-1,4-galactosyltransferase 1 OS=Homo sapiens OX=9606 GN=B4GALT1 PE=1 SV=5 |
| sp\|Q96R72\|OR4K3_HUMAN | plas | Olfactory receptor 4K3 OS=Homo sapiens OX=9606 GN=OR4K3 PE=3 SV=3 |
| sp\|Q8N668\|COMD1_HUMAN | cyto | COMM domain-containing protein 1 OS=Homo sapiens OX=9606 GN=COMMD1 PE=1 SV=1 |
| sp\|P03915\|NU5M_HUMAN | plas | NADH-ubiquinone oxidoreductase chain 5 OS=Homo sapiens OX=9606 GN=MT-ND5 PE=1 SV=2 |
| sp\|Q9H9Q4\|NHEJ1_HUMAN | nucl | Non-homologous end-joining factor 1 OS=Homo sapiens OX=9606 GN=NHEJ1 PE=1 SV=1 |
| sp\|Q8IV63\|VRK3_HUMAN | nucl | Inactive serine/threonine-protein kinase VRK3 OS=Homo sapiens OX=9606 GN=VRK3 PE=1 SV=2 |
| sp\|P42766\|RL35_HUMAN | cyto | 60S ribosomal protein L35 OS=Homo sapiens OX=9606 GN=RPL35 PE=1 SV=2 |
| sp\|A2A3L6\|TTC24_HUMAN | nucl | Tetratricopeptide repeat protein 24 OS=Homo sapiens OX=9606 GN=TTC24 PE=4 SV=1 |
| sp\|Q96AB6\|NTAN1_HUMAN | cyto | Protein N-terminal asparagine amidohydrolase OS=Homo sapiens OX=9606 GN=NTAN1 PE=1 SV=3 |
| sp\|O60318\|GANP_HUMAN | nucl | Germinal-center associated nuclear protein OS=Homo sapiens OX=9606 GN=MCM3AP PE=1 SV=2 |
| sp\|P20908\|CO5A1_HUMAN | extr | Collagen alpha-1(V) chain OS=Homo sapiens OX=9606 GN=COL5A1 PE=1 SV=3 |
| sp\|Q8WWZ4\|ABCAA_HUMAN | plas | ATP-binding cassette sub-family A member 10 OS=Homo sapiens OX=9606 GN=ABCA10 PE=2 SV=3 |
| sp\|P07910\|HNRPC_HUMAN | nucl | Heterogeneous nuclear ribonucleoproteins C1/C2 OS=Homo sapiens OX=9606 GN=HNRNPC PE=1 SV=4 |
| sp\|Q96N66\|MBOA7_HUMAN | plas | Lysophospholipid acyltransferase 7 OS=Homo sapiens OX=9606 GN=MBOAT7 PE=1 SV=2 |
| sp\|Q9NWM8\|FKB14_HUMAN | extr | Peptidyl-prolyl cis-trans isomerase FKBP14 OS=Homo sapiens OX=9606 GN=FKBP14 PE=1 SV=1 |
| sp\|Q5JRK9\|GGEE3_HUMAN | cyto_nucl | Putative G antigen family E member 3 OS=Homo sapiens OX=9606 GN=PAGE2B PE=3 SV=1 |
| sp\|P40763\|STAT3_HUMAN | cyto_nucl | Signal transducer and activator of transcription 3 OS=Homo sapiens OX=9606 GN=STAT3 PE=1 SV=2 |
| sp\|Q6XQN6\|PNCB_HUMAN | nucl | Nicotinate phosphoribosyltransferase OS=Homo sapiens OX=9606 GN=NAPRT PE=1 SV=2 |
| sp\|P22670\|RFX1_HUMAN | nucl | MHC class II regulatory factor RFX1 OS=Homo sapiens OX=9606 GN=RFX1 PE=1 SV=2 |
| sp\|Q9H1E3\|NUCKS_HUMAN | nucl | Nuclear ubiquitous casein and cyclin-dependent kinase substrate 1 OS=Homo sapiens OX=9606 GN=NUCKS1 PE=1 SV=1 |
| sp\|Q9Y5Q8\|TF3C5_HUMAN | cyto | General transcription factor 3C polypeptide 5 OS=Homo sapiens OX=9606 GN=GTF3C5 PE=1 SV=2 |
| sp\|Q9NZJ7\|MTCH1_HUMAN | plas | Mitochondrial carrier homolog 1 OS=Homo sapiens OX=9606 GN=MTCH1 PE=1 SV=1 |
| sp\|Q8IXI2\|MIRO1_HUMAN | cyto | Mitochondrial Rho GTPase 1 OS=Homo sapiens OX=9606 GN=RHOT1 PE=1 SV=2 |
| sp\|Q9H3H9\|TCAL2_HUMAN | nucl | Transcription elongation factor A protein-like 2 OS=Homo sapiens OX=9606 GN=TCEAL2 PE=2 SV=1 |
| sp\|Q9HD47\|MOG1_HUMAN | extr | Ran guanine nucleotide release factor OS=Homo sapiens OX=9606 GN=RANGRF PE=1 SV=1 |
| sp\|Q14515\|SPRL1_HUMAN | extr | SPARC-like protein 1 OS=Homo sapiens OX=9606 GN=SPARCL1 PE=1 SV=2 |
| sp\|P11388\|TOP2A_HUMAN | nucl | DNA topoisomerase 2-alpha OS=Homo sapiens OX=9606 GN=TOP2A PE=1 SV=3 |
| sp\|P84243\|H33_HUMAN | nucl | Histone H3.3 OS=Homo sapiens OX=9606 GN=H3F3A PE=1 SV=2 |
| sp\|Q9H6V9\|LDAH_HUMAN | cyto | Lipid droplet-associated hydrolase OS=Homo sapiens OX=9606 GN=LDAH PE=1 SV=1 |
| sp\|P78381\|S35A2_HUMAN | plas | UDP-galactose translocator OS=Homo sapiens OX=9606 GN=SLC35A2 PE=1 SV=1 |
| sp\|Q7KZN9\|COX15_HUMAN | plas | Cytochrome c oxidase assembly protein COX15 homolog OS=Homo sapiens OX=9606 GN=COX15 PE=1 SV=1 |
| sp\|Q03405\|UPAR_HUMAN | extr | Urokinase plasminogen activator surface receptor OS=Homo sapiens OX=9606 GN=PLAUR PE=1 SV=1 |
| sp\|O43353\|RIPK2_HUMAN | cyto | Receptor-interacting serine/threonine-protein kinase 2 OS=Homo sapiens OX=9606 GN=RIPK2 PE=1 SV=2 |
| sp\|Q8WTS1\|ABHD5_HUMAN | cyto | 1-acylglycerol-3-phosphate O-acyltransferase ABHD5 OS=Homo sapiens OX=9606 GN=ABHD5 PE=1 SV=1 |
| sp\|Q02952\|AKA12_HUMAN | nucl | A-kinase anchor protein 12 OS=Homo sapiens OX=9606 GN=AKAP12 PE=1 SV=4 |
| sp\|Q15370\|ELOB_HUMAN | cyto | Elongin-B OS=Homo sapiens OX=9606 GN=ELOB PE=1 SV=1 |
| sp\|Q9NQH7\|XPP3_HUMAN | mito | Xaa-Pro aminopeptidase 3 OS=Homo sapiens OX=9606 GN=XPNPEP3 PE=1 SV=1 |
| sp\|O96005\|CLPT1_HUMAN | plas | Cleft lip and palate transmembrane protein 1 OS=Homo sapiens OX=9606 GN=CLPTM1 PE=1 SV=1 |
| sp\|P08473\|NEP_HUMAN | plas | Neprilysin OS=Homo sapiens OX=9606 GN=MME PE=1 SV=2 |
| sp\|Q15075\|EEA1_HUMAN | nucl | Early endosome antigen 1 OS=Homo sapiens OX=9606 GN=EEA1 PE=1 SV=2 |
| sp\|Q6ZNA5\|FRRS1_HUMAN | plas | Ferric-chelate reductase 1 OS=Homo sapiens OX=9606 GN=FRRS1 PE=2 SV=2 |
| sp\|P08237\|PFKAM_HUMAN | cyto | ATP-dependent 6-phosphofructokinase, muscle type OS=Homo sapiens OX=9606 GN=PFKM PE=1 SV=2 |
| sp\|Q9UMX5\|NENF_HUMAN | extr | Neudesin OS=Homo sapiens OX=9606 GN=NENF PE=1 SV=1 |
| sp\|Q14157\|UBP2L_HUMAN | nucl | Ubiquitin-associated protein 2-like OS=Homo sapiens OX=9606 GN=UBAP2L PE=1 SV=2 |
| sp\|O76070\|SYUG_HUMAN | cyto | Gamma-synuclein OS=Homo sapiens OX=9606 GN=SNCG PE=1 SV=2 |
| sp\|P05141\|ADT2_HUMAN | cyto | ADP/ATP translocase 2 OS=Homo sapiens OX=9606 GN=SLC25A5 PE=1 SV=7 |
| sp\|Q99102\|MUC4_HUMAN | plas | Mucin-4 OS=Homo sapiens OX=9606 GN=MUC4 PE=1 SV=4 |
| sp\|P15090\|FABP4_HUMAN | cyto | Fatty acid-binding protein, adipocyte OS=Homo sapiens OX=9606 GN=FABP4 PE=1 SV=3 |
| sp\|P35443\|TSP4_HUMAN | extr | Thrombospondin-4 OS=Homo sapiens OX=9606 GN=THBS4 PE=1 SV=2 |
| sp\|O43292\|GPAA1_HUMAN | plas | Glycosylphosphatidylinositol anchor attachment 1 protein OS=Homo sapiens OX=9606 GN=GPAA1 PE=1 SV=3 |
| sp\|Q14247\|SRC8_HUMAN | cyto | Src substrate cortactin OS=Homo sapiens OX=9606 GN=CTTN PE=1 SV=2 |
| sp\|P49961\|ENTP1_HUMAN | plas | Ectonucleoside triphosphate diphosphohydrolase 1 OS=Homo sapiens OX=9606 GN=ENTPD1 PE=1 SV=1 |
| sp\|Q5SQS7\|SH24B_HUMAN | cyto | SH2 domain-containing protein 4B OS=Homo sapiens OX=9606 GN=SH2D4B PE=2 SV=1 |
| sp\|Q9NYQ7\|CELR3_HUMAN | plas | Cadherin EGF LAG seven-pass G-type receptor 3 OS=Homo sapiens OX=9606 GN=CELSR3 PE=1 SV=2 |
| sp\|P29762\|RABP1_HUMAN | cyto | Cellular retinoic acid-binding protein 1 OS=Homo sapiens OX=9606 GN=CRABP1 PE=1 SV=2 |
| sp\|Q01664\|TFAP4_HUMAN | nucl | Transcription factor AP-4 OS=Homo sapiens OX=9606 GN=TFAP4 PE=1 SV=2 |
| sp\|Q9Y295\|DRG1_HUMAN | cyto | Developmentally-regulated GTP-binding protein 1 OS=Homo sapiens OX=9606 GN=DRG1 PE=1 SV=1 |
| sp\|P24043\|LAMA2_HUMAN | extr | Laminin subunit alpha-2 OS=Homo sapiens OX=9606 GN=LAMA2 PE=1 SV=4 |
| sp\|Q9UNM6\|PSD13_HUMAN | cyto | 26S proteasome non-ATPase regulatory subunit 13 OS=Homo sapiens OX=9606 GN=PSMD13 PE=1 SV=2 |
| sp\|Q9UM54\|MYO6_HUMAN | cyto | Unconventional myosin-VI OS=Homo sapiens OX=9606 GN=MYO6 PE=1 SV=4 |
| sp\|Q96RL1\|UIMC1_HUMAN | nucl | BRCA1-A complex subunit RAP80 OS=Homo sapiens OX=9606 GN=UIMC1 PE=1 SV=2 |
| sp\|Q9BUH8\|BEGIN_HUMAN | nucl | Brain-enriched guanylate kinase-associated protein OS=Homo sapiens OX=9606 GN=BEGAIN PE=1 SV=1 |
| sp\|Q8NHH9\|ATLA2_HUMAN | plas | Atlastin-2 OS=Homo sapiens OX=9606 GN=ATL2 PE=1 SV=2 |
| sp\|O00410\|IPO5_HUMAN | cyto | Importin-5 OS=Homo sapiens OX=9606 GN=IPO5 PE=1 SV=4 |
| sp\|O75635\|SPB7_HUMAN | cyto | Serpin B7 OS=Homo sapiens OX=9606 GN=SERPINB7 PE=1 SV=1 |
| sp\|Q6EMK4\|VASN_HUMAN | extr | Vasorin OS=Homo sapiens OX=9606 GN=VASN PE=1 SV=1 |
| sp\|Q8NFP7\|NUD10_HUMAN | nucl | Diphosphoinositol polyphosphate phosphohydrolase 3-alpha OS=Homo sapiens OX=9606 GN=NUDT10 PE=1 SV=1 |
| sp\|P05496\|AT5G1_HUMAN | extr | ATP synthase F(0) complex subunit C1, mitochondrial OS=Homo sapiens OX=9606 GN=ATP5MC1 PE=1 SV=2 |
| sp\|P53041\|PPP5_HUMAN | cyto | Serine/threonine-protein phosphatase 5 OS=Homo sapiens OX=9606 GN=PPP5C PE=1 SV=1 |
| sp\|P51571\|SSRD_HUMAN | plas | Translocon-associated protein subunit delta OS=Homo sapiens OX=9606 GN=SSR4 PE=1 SV=1 |
| sp\|Q5QJ74\|TBCEL_HUMAN | cyto_nucl | Tubulin-specific chaperone cofactor E-like protein OS=Homo sapiens OX=9606 GN=TBCEL PE=1 SV=2 |
| sp\|Q8WV92\|MITD1_HUMAN | cyto | MIT domain-containing protein 1 OS=Homo sapiens OX=9606 GN=MITD1 PE=1 SV=1 |
| sp\|P52209\|6PGD_HUMAN | cyto_nucl | 6-phosphogluconate dehydrogenase, decarboxylating OS=Homo sapiens OX=9606 GN=PGD PE=1 SV=3 |
| sp\|Q08170\|SRSF4_HUMAN | nucl | Serine/arginine-rich splicing factor 4 OS=Homo sapiens OX=9606 GN=SRSF4 PE=1 SV=2 |
| sp\|P26196\|DDX6_HUMAN | nucl | Probable ATP-dependent RNA helicase DDX6 OS=Homo sapiens OX=9606 GN=DDX6 PE=1 SV=2 |
| sp\|Q9BV36\|MELPH_HUMAN | nucl | Melanophilin OS=Homo sapiens OX=9606 GN=MLPH PE=1 SV=1 |
| sp\|Q5T1V6\|DDX59_HUMAN | cyto_nucl | Probable ATP-dependent RNA helicase DDX59 OS=Homo sapiens OX=9606 GN=DDX59 PE=1 SV=1 |
| sp\|Q3MHD2\|LSM12_HUMAN | cyto_nucl | Protein LSM12 homolog OS=Homo sapiens OX=9606 GN=LSM12 PE=1 SV=2 |
| sp\|Q96JG6\|VPS50_HUMAN | nucl | Syndetin OS=Homo sapiens OX=9606 GN=VPS50 PE=1 SV=3 |
| sp\|P08779\|K1C16_HUMAN | nucl | Keratin, type I cytoskeletal 16 OS=Homo sapiens OX=9606 GN=KRT16 PE=1 SV=4 |
| sp\|P31483\|TIA1_HUMAN | nucl | Nucleolysin TIA-1 isoform p40 OS=Homo sapiens OX=9606 GN=TIA1 PE=1 SV=3 |
| sp\|O43290\|SNUT1_HUMAN | nucl | U4/U6.U5 tri-snRNP-associated protein 1 OS=Homo sapiens OX=9606 GN=SART1 PE=1 SV=1 |
| sp\|P01019\|ANGT_HUMAN | extr | Angiotensinogen OS=Homo sapiens OX=9606 GN=AGT PE=1 SV=1 |
| sp\|Q14435\|GALT3_HUMAN | golg | Polypeptide N-acetylgalactosaminyltransferase 3 OS=Homo sapiens OX=9606 GN=GALNT3 PE=1 SV=2 |
| sp\|Q9UM22\|EPDR1_HUMAN | extr | Mammalian ependymin-related protein 1 OS=Homo sapiens OX=9606 GN=EPDR1 PE=1 SV=2 |
| sp\|Q9BQI9\|NRIP2_HUMAN | cyto | Nuclear receptor-interacting protein 2 OS=Homo sapiens OX=9606 GN=NRIP2 PE=1 SV=3 |
| sp\|Q9HBG6\|IF122_HUMAN | cyto | Intraflagellar transport protein 122 homolog OS=Homo sapiens OX=9606 GN=IFT122 PE=1 SV=2 |
| sp\|P54727\|RD23B_HUMAN | cyto | UV excision repair protein RAD23 homolog B OS=Homo sapiens OX=9606 GN=RAD23B PE=1 SV=1 |
| sp\|Q9H920\|RN121_HUMAN | plas | RING finger protein 121 OS=Homo sapiens OX=9606 GN=RNF121 PE=1 SV=1 |
| sp\|P07451\|CAH3_HUMAN | cyto | Carbonic anhydrase 3 OS=Homo sapiens OX=9606 GN=CA3 PE=1 SV=3 |
| sp\|P20290\|BTF3_HUMAN | nucl | Transcription factor BTF3 OS=Homo sapiens OX=9606 GN=BTF3 PE=1 SV=1 |
| sp\|Q9HAR2\|AGRL3_HUMAN | plas | Adhesion G protein-coupled receptor L3 OS=Homo sapiens OX=9606 GN=ADGRL3 PE=1 SV=2 |
| sp\|Q99983\|OMD_HUMAN | plas | Osteomodulin OS=Homo sapiens OX=9606 GN=OMD PE=1 SV=1 |
| sp\|O43861\|ATP9B_HUMAN | plas | Probable phospholipid-transporting ATPase IIB OS=Homo sapiens OX=9606 GN=ATP9B PE=2 SV=4 |
| sp\|Q09161\|NCBP1_HUMAN | nucl | Nuclear cap-binding protein subunit 1 OS=Homo sapiens OX=9606 GN=NCBP1 PE=1 SV=1 |
| sp\|O75691\|UTP20_HUMAN | nucl | Small subunit processome component 20 homolog OS=Homo sapiens OX=9606 GN=UTP20 PE=1 SV=3 |
| sp\|Q9Y680\|FKBP7_HUMAN | extr | Peptidyl-prolyl cis-trans isomerase FKBP7 OS=Homo sapiens OX=9606 GN=FKBP7 PE=1 SV=2 |
| sp\|Q96SQ9\|CP2S1_HUMAN | extr | Cytochrome P450 2S1 OS=Homo sapiens OX=9606 GN=CYP2S1 PE=1 SV=2 |
| sp\|P43007\|SATT_HUMAN | plas | Neutral amino acid transporter A OS=Homo sapiens OX=9606 GN=SLC1A4 PE=1 SV=1 |
| sp\|P0DOY2\|IGLC2_HUMAN | extr | Immunoglobulin lambda constant 2 OS=Homo sapiens OX=9606 GN=IGLC2 PE=1 SV=1 |
| sp\|Q02742\|GCNT1_HUMAN | plas | Beta-1,3-galactosyl-O-glycosyl-glycoprotein beta-1,6-N-acetylglucosaminyltransferase OS=Homo sapiens OX=9606 GN=GCNT1 PE=1 SV=2 |
| sp\|Q9Y673\|ALG5_HUMAN | E.R. | Dolichyl-phosphate beta-glucosyltransferase OS=Homo sapiens OX=9606 GN=ALG5 PE=1 SV=1 |
| sp\|Q9Y6E2\|BZW2_HUMAN | nucl | Basic leucine zipper and W2 domain-containing protein 2 OS=Homo sapiens OX=9606 GN=BZW2 PE=1 SV=1 |
| sp\|P0DOX7\|IGK_HUMAN | nucl | Immunoglobulin kappa light chain OS=Homo sapiens OX=9606 PE=1 SV=1 |
| sp\|Q53F19\|NCBP3_HUMAN | nucl | Nuclear cap-binding protein subunit 3 OS=Homo sapiens OX=9606 GN=NCBP3 PE=1 SV=2 |
| sp\|P21359\|NF1_HUMAN | nucl | Neurofibromin OS=Homo sapiens OX=9606 GN=NF1 PE=1 SV=2 |
| sp\|Q96Q06\|PLIN4_HUMAN | mito | Perilipin-4 OS=Homo sapiens OX=9606 GN=PLIN4 PE=1 SV=2 |
| sp\|O00115\|DNS2A_HUMAN | extr | Deoxyribonuclease-2-alpha OS=Homo sapiens OX=9606 GN=DNASE2 PE=1 SV=2 |
| sp\|P54855\|UDB15_HUMAN | pero | UDP-glucuronosyltransferase 2B15 OS=Homo sapiens OX=9606 GN=UGT2B15 PE=1 SV=3 |
| sp\|P02533\|K1C14_HUMAN | nucl | Keratin, type I cytoskeletal 14 OS=Homo sapiens OX=9606 GN=KRT14 PE=1 SV=4 |
| sp\|P48681\|NEST_HUMAN | nucl | Nestin OS=Homo sapiens OX=9606 GN=NES PE=1 SV=2 |
| sp\|O14732\|IMPA2_HUMAN | cysk | Inositol monophosphatase 2 OS=Homo sapiens OX=9606 GN=IMPA2 PE=1 SV=1 |
| sp\|O95858\|TSN15_HUMAN | plas | Tetraspanin-15 OS=Homo sapiens OX=9606 GN=TSPAN15 PE=1 SV=1 |
| sp\|Q13342\|SP140_HUMAN | nucl | Nuclear body protein SP140 OS=Homo sapiens OX=9606 GN=SP140 PE=1 SV=2 |
| sp\|P16333\|NCK1_HUMAN | nucl | Cytoplasmic protein NCK1 OS=Homo sapiens OX=9606 GN=NCK1 PE=1 SV=1 |
| sp\|Q92832\|NELL1_HUMAN | extr | Protein kinase C-binding protein NELL1 OS=Homo sapiens OX=9606 GN=NELL1 PE=1 SV=4 |
| sp\|P43358\|MAGA4_HUMAN | cyto | Melanoma-associated antigen 4 OS=Homo sapiens OX=9606 GN=MAGEA4 PE=1 SV=2 |
| sp\|O60760\|HPGDS_HUMAN | cyto | Hematopoietic prostaglandin D synthase OS=Homo sapiens OX=9606 GN=HPGDS PE=1 SV=3 |
| sp\|P62314\|SMD1_HUMAN | nucl | Small nuclear ribonucleoprotein Sm D1 OS=Homo sapiens OX=9606 GN=SNRPD1 PE=1 SV=1 |
| sp\|Q9NS86\|LANC2_HUMAN | cyto | LanC-like protein 2 OS=Homo sapiens OX=9606 GN=LANCL2 PE=1 SV=1 |
| sp\|Q9NUQ6\|SPS2L_HUMAN | nucl | SPATS2-like protein OS=Homo sapiens OX=9606 GN=SPATS2L PE=1 SV=2 |
| sp\|Q09328\|MGT5A_HUMAN | E.R. | Alpha-1,6-mannosylglycoprotein 6-beta-N-acetylglucosaminyltransferase A OS=Homo sapiens OX=9606 GN=MGAT5 PE=1 SV=1 |
| sp\|P27816\|MAP4_HUMAN | nucl | Microtubule-associated protein 4 OS=Homo sapiens OX=9606 GN=MAP4 PE=1 SV=3 |
| sp\|P08240\|SRPRA_HUMAN | cyto | Signal recognition particle receptor subunit alpha OS=Homo sapiens OX=9606 GN=SRPRA PE=1 SV=2 |
| sp\|O95070\|YIF1A_HUMAN | plas | Protein YIF1A OS=Homo sapiens OX=9606 GN=YIF1A PE=1 SV=2 |
| sp\|Q9C0I1\|MTMRC_HUMAN | cyto | Myotubularin-related protein 12 OS=Homo sapiens OX=9606 GN=MTMR12 PE=1 SV=2 |
| sp\|Q92747\|ARC1A_HUMAN | cyto_nucl | Actin-related protein 2/3 complex subunit 1A OS=Homo sapiens OX=9606 GN=ARPC1A PE=2 SV=2 |
| sp\|Q8N4A0\|GALT4_HUMAN | E.R. | Polypeptide N-acetylgalactosaminyltransferase 4 OS=Homo sapiens OX=9606 GN=GALNT4 PE=1 SV=2 |
| sp\|Q9H8M1\|CQ10B_HUMAN | mito | Coenzyme Q-binding protein COQ10 homolog B, mitochondrial OS=Homo sapiens OX=9606 GN=COQ10B PE=2 SV=1 |
| sp\|P51888\|PRELP_HUMAN | extr | Prolargin OS=Homo sapiens OX=9606 GN=PRELP PE=1 SV=1 |
| sp\|Q9Y617\|SERC_HUMAN | cyto | Phosphoserine aminotransferase OS=Homo sapiens OX=9606 GN=PSAT1 PE=1 SV=2 |
| sp\|A0A075B6H9\|LV469_HUMAN | extr | Immunoglobulin lambda variable 4-69 OS=Homo sapiens OX=9606 GN=IGLV4-69 PE=1 SV=1 |
| sp\|P38435\|VKGC_HUMAN | nucl | Vitamin K-dependent gamma-carboxylase OS=Homo sapiens OX=9606 GN=GGCX PE=1 SV=2 |
| sp\|P20061\|TCO1_HUMAN | extr | Transcobalamin-1 OS=Homo sapiens OX=9606 GN=TCN1 PE=1 SV=2 |
| sp\|Q9HCJ1\|ANKH_HUMAN | plas | Progressive ankylosis protein homolog OS=Homo sapiens OX=9606 GN=ANKH PE=1 SV=2 |
| sp\|Q00765\|REEP5_HUMAN | cyto | Receptor expression-enhancing protein 5 OS=Homo sapiens OX=9606 GN=REEP5 PE=1 SV=3 |
| sp\|Q9HCL2\|GPAT1_HUMAN | cyto | Glycerol-3-phosphate acyltransferase 1, mitochondrial OS=Homo sapiens OX=9606 GN=GPAM PE=1 SV=3 |
| sp\|A0A0B4J248\|TVA11_HUMAN | extr | T cell receptor alpha variable 1-1 OS=Homo sapiens OX=9606 GN=TRAV1-1 PE=3 SV=1 |
| sp\|Q9Y277\|VDAC3_HUMAN | cyto | Voltage-dependent anion-selective channel protein 3 OS=Homo sapiens OX=9606 GN=VDAC3 PE=1 SV=1 |
| sp\|Q6UX06\|OLFM4_HUMAN | extr | Olfactomedin-4 OS=Homo sapiens OX=9606 GN=OLFM4 PE=1 SV=1 |
| sp\|Q9NX02\|NALP2_HUMAN | cyto | NACHT, LRR and PYD domains-containing protein 2 OS=Homo sapiens OX=9606 GN=NLRP2 PE=1 SV=1 |
| sp\|P06312\|KV401_HUMAN | extr | Immunoglobulin kappa variable 4-1 OS=Homo sapiens OX=9606 GN=IGKV4-1 PE=1 SV=1 |
| sp\|P23327\|SRCH_HUMAN | extr | Sarcoplasmic reticulum histidine-rich calcium-binding protein OS=Homo sapiens OX=9606 GN=HRC PE=1 SV=1 |
| sp\|Q9ULS5\|TMCC3_HUMAN | plas | Transmembrane and coiled-coil domain protein 3 OS=Homo sapiens OX=9606 GN=TMCC3 PE=1 SV=3 |
| sp\|Q8TEK3\|DOT1L_HUMAN | nucl | Histone-lysine N-methyltransferase, H3 lysine-79 specific OS=Homo sapiens OX=9606 GN=DOT1L PE=1 SV=3 |
| sp\|P51809\|VAMP7_HUMAN | pero | Vesicle-associated membrane protein 7 OS=Homo sapiens OX=9606 GN=VAMP7 PE=1 SV=3 |
| sp\|P14209\|CD99_HUMAN | extr | CD99 antigen OS=Homo sapiens OX=9606 GN=CD99 PE=1 SV=1 |
| sp\|Q2KHT3\|CL16A_HUMAN | nucl | Protein CLEC16A OS=Homo sapiens OX=9606 GN=CLEC16A PE=1 SV=2 |
| sp\|Q9Y679\|AUP1_HUMAN | plas | Ancient ubiquitous protein 1 OS=Homo sapiens OX=9606 GN=AUP1 PE=1 SV=2 |
| sp\|Q96QK1\|VPS35_HUMAN | cyto | Vacuolar protein sorting-associated protein 35 OS=Homo sapiens OX=9606 GN=VPS35 PE=1 SV=2 |
| sp\|Q9P2P5\|HECW2_HUMAN | mito | E3 ubiquitin-protein ligase HECW2 OS=Homo sapiens OX=9606 GN=HECW2 PE=1 SV=2 |
| sp\|Q13505\|MTX1_HUMAN | nucl | Metaxin-1 OS=Homo sapiens OX=9606 GN=MTX1 PE=1 SV=3 |
| sp\|Q86X10\|RLGPB_HUMAN | cyto | Ral GTPase-activating protein subunit beta OS=Homo sapiens OX=9606 GN=RALGAPB PE=1 SV=1 |
| sp\|Q9H4A4\|AMPB_HUMAN | nucl | Aminopeptidase B OS=Homo sapiens OX=9606 GN=RNPEP PE=1 SV=2 |
| sp\|Q6UX71\|PXDC2_HUMAN | pero | Plexin domain-containing protein 2 OS=Homo sapiens OX=9606 GN=PLXDC2 PE=1 SV=1 |
| sp\|Q9UP83\|COG5_HUMAN | nucl | Conserved oligomeric Golgi complex subunit 5 OS=Homo sapiens OX=9606 GN=COG5 PE=1 SV=3 |
| sp\|Q9NZ45\|CISD1_HUMAN | extr | CDGSH iron-sulfur domain-containing protein 1 OS=Homo sapiens OX=9606 GN=CISD1 PE=1 SV=1 |
| sp\|O43592\|XPOT_HUMAN | cyto | Exportin-T OS=Homo sapiens OX=9606 GN=XPOT PE=1 SV=2 |
| sp\|P59998\|ARPC4_HUMAN | mito | Actin-related protein 2/3 complex subunit 4 OS=Homo sapiens OX=9606 GN=ARPC4 PE=1 SV=3 |
| sp\|Q96CN7\|ISOC1_HUMAN | cyto | Isochorismatase domain-containing protein 1 OS=Homo sapiens OX=9606 GN=ISOC1 PE=1 SV=3 |
| sp\|Q5TH69\|BIG3_HUMAN | nucl | Brefeldin A-inhibited guanine nucleotide-exchange protein 3 OS=Homo sapiens OX=9606 GN=ARFGEF3 PE=1 SV=3 |
| sp\|P00326\|ADH1G_HUMAN | cyto | Alcohol dehydrogenase 1C OS=Homo sapiens OX=9606 GN=ADH1C PE=1 SV=2 |
| sp\|O60287\|NPA1P_HUMAN | plas | Nucleolar pre-ribosomal-associated protein 1 OS=Homo sapiens OX=9606 GN=URB1 PE=1 SV=4 |
| sp\|P02794\|FRIH_HUMAN | cyto | Ferritin heavy chain OS=Homo sapiens OX=9606 GN=FTH1 PE=1 SV=2 |
| sp\|P02461\|CO3A1_HUMAN | extr | Collagen alpha-1(III) chain OS=Homo sapiens OX=9606 GN=COL3A1 PE=1 SV=4 |
| sp\|Q9BTL3\|RAMAC_HUMAN | extr | RNA guanine-N7 methyltransferase activating subunit OS=Homo sapiens OX=9606 GN=RAMAC PE=1 SV=1 |
| sp\|P06858\|LIPL_HUMAN | extr | Lipoprotein lipase OS=Homo sapiens OX=9606 GN=LPL PE=1 SV=1 |
| sp\|P02511\|CRYAB_HUMAN | mito | Alpha-crystallin B chain OS=Homo sapiens OX=9606 GN=CRYAB PE=1 SV=2 |
| sp\|P05023\|AT1A1_HUMAN | plas | Sodium/potassium-transporting ATPase subunit alpha-1 OS=Homo sapiens OX=9606 GN=ATP1A1 PE=1 SV=1 |
| sp\|Q6P1Q0\|LTMD1_HUMAN | mito | LETM1 domain-containing protein 1 OS=Homo sapiens OX=9606 GN=LETMD1 PE=1 SV=1 |
| sp\|Q14249\|NUCG_HUMAN | extr | Endonuclease G, mitochondrial OS=Homo sapiens OX=9606 GN=ENDOG PE=1 SV=4 |
| sp\|Q8WXI9\|P66B_HUMAN | cyto | Transcriptional repressor p66-beta OS=Homo sapiens OX=9606 GN=GATAD2B PE=1 SV=1 |
| sp\|Q9BV23\|ABHD6_HUMAN | extr | Monoacylglycerol lipase ABHD6 OS=Homo sapiens OX=9606 GN=ABHD6 PE=1 SV=1 |
| sp\|O95470\|SGPL1_HUMAN | cyto | Sphingosine-1-phosphate lyase 1 OS=Homo sapiens OX=9606 GN=SGPL1 PE=1 SV=3 |
| sp\|P05546\|HEP2_HUMAN | extr | Heparin cofactor 2 OS=Homo sapiens OX=9606 GN=SERPIND1 PE=1 SV=3 |
| sp\|Q63ZE4\|S22AA_HUMAN | plas | Solute carrier family 22 member 10 OS=Homo sapiens OX=9606 GN=SLC22A10 PE=2 SV=2 |
| sp\|Q03113\|GNA12_HUMAN | mito | Guanine nucleotide-binding protein subunit alpha-12 OS=Homo sapiens OX=9606 GN=GNA12 PE=1 SV=4 |
| sp\|O76095\|JTB_HUMAN | extr | Protein JTB OS=Homo sapiens OX=9606 GN=JTB PE=1 SV=1 |
| sp\|Q6KCM7\|SCMC2_HUMAN | cyto | Calcium-binding mitochondrial carrier protein SCaMC-2 OS=Homo sapiens OX=9606 GN=SLC25A25 PE=1 SV=1 |
| sp\|Q8WUH6\|TM263_HUMAN | cyto | Transmembrane protein 263 OS=Homo sapiens OX=9606 GN=TMEM263 PE=1 SV=1 |
| sp\|Q70J99\|UN13D_HUMAN | nucl | Protein unc-13 homolog D OS=Homo sapiens OX=9606 GN=UNC13D PE=1 SV=1 |
| sp\|P15529\|MCP_HUMAN | plas | Membrane cofactor protein OS=Homo sapiens OX=9606 GN=CD46 PE=1 SV=3 |
| sp\|P08493\|MGP_HUMAN | extr | Matrix Gla protein OS=Homo sapiens OX=9606 GN=MGP PE=1 SV=2 |
| sp\|Q7Z6B7\|SRGP1_HUMAN | nucl | SLIT-ROBO Rho GTPase-activating protein 1 OS=Homo sapiens OX=9606 GN=SRGAP1 PE=1 SV=1 |
| sp\|P52298\|NCBP2_HUMAN | cyto | Nuclear cap-binding protein subunit 2 OS=Homo sapiens OX=9606 GN=NCBP2 PE=1 SV=1 |
| sp\|P14735\|IDE_HUMAN | mito | Insulin-degrading enzyme OS=Homo sapiens OX=9606 GN=IDE PE=1 SV=4 |
| sp\|Q16563\|SYPL1_HUMAN | plas | Synaptophysin-like protein 1 OS=Homo sapiens OX=9606 GN=SYPL1 PE=1 SV=1 |
| sp\|Q5HYK7\|SH319_HUMAN | nucl | SH3 domain-containing protein 19 OS=Homo sapiens OX=9606 GN=SH3D19 PE=1 SV=2 |
| sp\|Q9UQP3\|TENN_HUMAN | extr | Tenascin-N OS=Homo sapiens OX=9606 GN=TNN PE=1 SV=2 |
| sp\|Q15031\|SYLM_HUMAN | mito | Probable leucine--tRNA ligase, mitochondrial OS=Homo sapiens OX=9606 GN=LARS2 PE=1 SV=2 |
| sp\|Q07812\|BAX_HUMAN | plas | Apoptosis regulator BAX OS=Homo sapiens OX=9606 GN=BAX PE=1 SV=1 |
| sp\|P47712\|PA24A_HUMAN | nucl | Cytosolic phospholipase A2 OS=Homo sapiens OX=9606 GN=PLA2G4A PE=1 SV=2 |
| sp\|Q9UKY7\|CDV3_HUMAN | nucl | Protein CDV3 homolog OS=Homo sapiens OX=9606 GN=CDV3 PE=1 SV=1 |
| sp\|O15260\|SURF4_HUMAN | plas | Surfeit locus protein 4 OS=Homo sapiens OX=9606 GN=SURF4 PE=1 SV=3 |
| sp\|Q9BS40\|LXN_HUMAN | cyto | Latexin OS=Homo sapiens OX=9606 GN=LXN PE=1 SV=2 |
| sp\|O95407\|TNF6B_HUMAN | extr | Tumor necrosis factor receptor superfamily member 6B OS=Homo sapiens OX=9606 GN=TNFRSF6B PE=1 SV=1 |
| sp\|Q9UKV8\|AGO2_HUMAN | cyto | Protein argonaute-2 OS=Homo sapiens OX=9606 GN=AGO2 PE=1 SV=3 |
| sp\|Q9Y6N5\|SQOR_HUMAN | mito | Sulfide:quinone oxidoreductase, mitochondrial OS=Homo sapiens OX=9606 GN=SQOR PE=1 SV=1 |
| sp\|Q99766\|ATP5S_HUMAN | extr | ATP synthase subunit s, mitochondrial OS=Homo sapiens OX=9606 GN=DMAC2L PE=1 SV=3 |
| sp\|Q8IXS6\|PALM2_HUMAN | cyto_nucl | Paralemmin-2 OS=Homo sapiens OX=9606 GN=PALM2 PE=1 SV=3 |
| sp\|Q6IA17\|SIGIR_HUMAN | plas | Single Ig IL-1-related receptor OS=Homo sapiens OX=9606 GN=SIGIRR PE=1 SV=3 |
| sp\|P13928\|ANXA8_HUMAN | cyto | Annexin A8 OS=Homo sapiens OX=9606 GN=ANXA8 PE=1 SV=3 |
| sp\|O60921\|HUS1_HUMAN | cyto | Checkpoint protein HUS1 OS=Homo sapiens OX=9606 GN=HUS1 PE=1 SV=1 |
| sp\|Q9Y5Z4\|HEBP2_HUMAN | cyto | Heme-binding protein 2 OS=Homo sapiens OX=9606 GN=HEBP2 PE=1 SV=1 |
| sp\|Q96EV2\|RBM33_HUMAN | nucl | RNA-binding protein 33 OS=Homo sapiens OX=9606 GN=RBM33 PE=1 SV=3 |
| sp\|Q16777\|H2A2C_HUMAN | nucl | Histone H2A type 2-C OS=Homo sapiens OX=9606 GN=HIST2H2AC PE=1 SV=4 |
| sp\|Q8N0X7\|SPART_HUMAN | nucl | Spartin OS=Homo sapiens OX=9606 GN=SPART PE=1 SV=1 |
| sp\|P00505\|AATM_HUMAN | mito | Aspartate aminotransferase, mitochondrial OS=Homo sapiens OX=9606 GN=GOT2 PE=1 SV=3 |
| sp\|Q9UBM7\|DHCR7_HUMAN | plas | 7-dehydrocholesterol reductase OS=Homo sapiens OX=9606 GN=DHCR7 PE=1 SV=1 |
| sp\|Q9Y3E1\|HDGR3_HUMAN | nucl | Hepatoma-derived growth factor-related protein 3 OS=Homo sapiens OX=9606 GN=HDGFL3 PE=1 SV=1 |
| sp\|Q96SM3\|CPXM1_HUMAN | extr | Probable carboxypeptidase X1 OS=Homo sapiens OX=9606 GN=CPXM1 PE=2 SV=2 |
| sp\|P21926\|CD9_HUMAN | plas | CD9 antigen OS=Homo sapiens OX=9606 GN=CD9 PE=1 SV=4 |
| sp\|Q08722\|CD47_HUMAN | plas | Leukocyte surface antigen CD47 OS=Homo sapiens OX=9606 GN=CD47 PE=1 SV=1 |
| sp\|Q9H1E5\|TMX4_HUMAN | extr | Thioredoxin-related transmembrane protein 4 OS=Homo sapiens OX=9606 GN=TMX4 PE=1 SV=1 |
| sp\|P00338\|LDHA_HUMAN | cyto | L-lactate dehydrogenase A chain OS=Homo sapiens OX=9606 GN=LDHA PE=1 SV=2 |
| sp\|P09234\|RU1C_HUMAN | nucl | U1 small nuclear ribonucleoprotein C OS=Homo sapiens OX=9606 GN=SNRPC PE=1 SV=1 |
| sp\|P78346\|RPP30_HUMAN | cyto | Ribonuclease P protein subunit p30 OS=Homo sapiens OX=9606 GN=RPP30 PE=1 SV=1 |
| sp\|Q9NZL4\|HPBP1_HUMAN | nucl | Hsp70-binding protein 1 OS=Homo sapiens OX=9606 GN=HSPBP1 PE=1 SV=2 |
| sp\|P21283\|VATC1_HUMAN | cyto | V-type proton ATPase subunit C 1 OS=Homo sapiens OX=9606 GN=ATP6V1C1 PE=1 SV=4 |
| sp\|Q96JJ7\|TMX3_HUMAN | plas | Protein disulfide-isomerase TMX3 OS=Homo sapiens OX=9606 GN=TMX3 PE=1 SV=2 |
| sp\|P21980\|TGM2_HUMAN | cyto | Protein-glutamine gamma-glutamyltransferase 2 OS=Homo sapiens OX=9606 GN=TGM2 PE=1 SV=2 |
| sp\|O95810\|CAVN2_HUMAN | cyto | Caveolae-associated protein 2 OS=Homo sapiens OX=9606 GN=CAVIN2 PE=1 SV=3 |
| sp\|Q9Y5S1\|TRPV2_HUMAN | plas | Transient receptor potential cation channel subfamily V member 2 OS=Homo sapiens OX=9606 GN=TRPV2 PE=1 SV=1 |
| sp\|Q7Z7G0\|TARSH_HUMAN | extr | Target of Nesh-SH3 OS=Homo sapiens OX=9606 GN=ABI3BP PE=1 SV=1 |
| sp\|Q9BVK6\|TMED9_HUMAN | pero | Transmembrane emp24 domain-containing protein 9 OS=Homo sapiens OX=9606 GN=TMED9 PE=1 SV=2 |
| sp\|Q92804\|RBP56_HUMAN | nucl | TATA-binding protein-associated factor 2N OS=Homo sapiens OX=9606 GN=TAF15 PE=1 SV=1 |
| sp\|Q93084\|AT2A3_HUMAN | plas | Sarcoplasmic/endoplasmic reticulum calcium ATPase 3 OS=Homo sapiens OX=9606 GN=ATP2A3 PE=1 SV=2 |
| sp\|P35269\|T2FA_HUMAN | nucl | General transcription factor IIF subunit 1 OS=Homo sapiens OX=9606 GN=GTF2F1 PE=1 SV=2 |
| sp\|Q8N9U0\|TAC2N_HUMAN | nucl | Tandem C2 domains nuclear protein OS=Homo sapiens OX=9606 GN=TC2N PE=1 SV=2 |
| sp\|P78368\|KC1G2_HUMAN | cyto_nucl | Casein kinase I isoform gamma-2 OS=Homo sapiens OX=9606 GN=CSNK1G2 PE=1 SV=1 |
| sp\|P45880\|VDAC2_HUMAN | cyto | Voltage-dependent anion-selective channel protein 2 OS=Homo sapiens OX=9606 GN=VDAC2 PE=1 SV=2 |
| sp\|Q7Z6K3\|PTAR1_HUMAN | cysk | Protein prenyltransferase alpha subunit repeat-containing protein 1 OS=Homo sapiens OX=9606 GN=PTAR1 PE=1 SV=2 |
| sp\|Q99584\|S10AD_HUMAN | cyto | Protein S100-A13 OS=Homo sapiens OX=9606 GN=S100A13 PE=1 SV=1 |
| sp\|O43149\|ZZEF1_HUMAN | plas | Zinc finger ZZ-type and EF-hand domain-containing protein 1 OS=Homo sapiens OX=9606 GN=ZZEF1 PE=1 SV=6 |
| sp\|A5YKK6\|CNOT1_HUMAN | cyto | CCR4-NOT transcription complex subunit 1 OS=Homo sapiens OX=9606 GN=CNOT1 PE=1 SV=2 |
| sp\|Q13627\|DYR1A_HUMAN | nucl | Dual specificity tyrosine-phosphorylation-regulated kinase 1A OS=Homo sapiens OX=9606 GN=DYRK1A PE=1 SV=2 |
| sp\|P06400\|RB_HUMAN | nucl | Retinoblastoma-associated protein OS=Homo sapiens OX=9606 GN=RB1 PE=1 SV=2 |
| sp\|P20810\|ICAL_HUMAN | nucl | Calpastatin OS=Homo sapiens OX=9606 GN=CAST PE=1 SV=4 |
| sp\|P07195\|LDHB_HUMAN | cyto | L-lactate dehydrogenase B chain OS=Homo sapiens OX=9606 GN=LDHB PE=1 SV=2 |
| sp\|Q5VYS4\|MEDAG_HUMAN | nucl | Mesenteric estrogen-dependent adipogenesis protein OS=Homo sapiens OX=9606 GN=MEDAG PE=2 SV=1 |
| sp\|Q05655\|KPCD_HUMAN | cyto | Protein kinase C delta type OS=Homo sapiens OX=9606 GN=PRKCD PE=1 SV=2 |
| sp\|Q96S96\|PEBP4_HUMAN | extr | Phosphatidylethanolamine-binding protein 4 OS=Homo sapiens OX=9606 GN=PEBP4 PE=1 SV=3 |
| sp\|Q6BDS2\|URFB1_HUMAN | nucl | UHRF1-binding protein 1 OS=Homo sapiens OX=9606 GN=UHRF1BP1 PE=1 SV=1 |
| sp\|Q04771\|ACVR1_HUMAN | plas | Activin receptor type-1 OS=Homo sapiens OX=9606 GN=ACVR1 PE=1 SV=1 |
| sp\|Q06547\|GABP1_HUMAN | cysk | GA-binding protein subunit beta-1 OS=Homo sapiens OX=9606 GN=GABPB1 PE=1 SV=2 |
| sp\|Q96S97\|MYADM_HUMAN | plas | Myeloid-associated differentiation marker OS=Homo sapiens OX=9606 GN=MYADM PE=1 SV=2 |
| sp\|Q9BX97\|PLVAP_HUMAN | plas | Plasmalemma vesicle-associated protein OS=Homo sapiens OX=9606 GN=PLVAP PE=2 SV=1 |
| sp\|O94915\|FRYL_HUMAN | plas | Protein furry homolog-like OS=Homo sapiens OX=9606 GN=FRYL PE=1 SV=2 |
| sp\|Q969G5\|CAVN3_HUMAN | extr | Caveolae-associated protein 3 OS=Homo sapiens OX=9606 GN=CAVIN3 PE=1 SV=3 |
| sp\|P62328\|TYB4_HUMAN | nucl | Thymosin beta-4 OS=Homo sapiens OX=9606 GN=TMSB4X PE=1 SV=2 |
| sp\|Q13825\|AUHM_HUMAN | mito | Methylglutaconyl-CoA hydratase, mitochondrial OS=Homo sapiens OX=9606 GN=AUH PE=1 SV=1 |
| sp\|Q6L9W6\|B4GN3_HUMAN | mito | Beta-1,4-N-acetylgalactosaminyltransferase 3 OS=Homo sapiens OX=9606 GN=B4GALNT3 PE=1 SV=2 |
| sp\|Q8WUH1\|CHUR_HUMAN | mito | Protein Churchill OS=Homo sapiens OX=9606 GN=CHURC1 PE=1 SV=2 |
| sp\|P08118\|MSMB_HUMAN | extr | Beta-microseminoprotein OS=Homo sapiens OX=9606 GN=MSMB PE=1 SV=1 |
| sp\|P02545\|LMNA_HUMAN | nucl | Prelamin-A/C OS=Homo sapiens OX=9606 GN=LMNA PE=1 SV=1 |
| sp\|P02746\|C1QB_HUMAN | extr | Complement C1q subcomponent subunit B OS=Homo sapiens OX=9606 GN=C1QB PE=1 SV=3 |
| sp\|P56556\|NDUA6_HUMAN | mito | NADH dehydrogenase [ubiquinone] 1 alpha subcomplex subunit 6 OS=Homo sapiens OX=9606 GN=NDUFA6 PE=1 SV=4 |
| sp\|Q8NDX5\|PHC3_HUMAN | nucl | Polyhomeotic-like protein 3 OS=Homo sapiens OX=9606 GN=PHC3 PE=1 SV=1 |
| sp\|Q8N9N2\|ASCC1_HUMAN | cyto | Activating signal cointegrator 1 complex subunit 1 OS=Homo sapiens OX=9606 GN=ASCC1 PE=1 SV=1 |
| sp\|Q02338\|BDH_HUMAN | mito | D-beta-hydroxybutyrate dehydrogenase, mitochondrial OS=Homo sapiens OX=9606 GN=BDH1 PE=1 SV=3 |
| sp\|Q8IWU6\|SULF1_HUMAN | E.R. | Extracellular sulfatase Sulf-1 OS=Homo sapiens OX=9606 GN=SULF1 PE=1 SV=1 |
| sp\|P01834\|IGKC_HUMAN | extr | Immunoglobulin kappa constant OS=Homo sapiens OX=9606 GN=IGKC PE=1 SV=2 |
| sp\|Q9H668\|STN1_HUMAN | extr | CST complex subunit STN1 OS=Homo sapiens OX=9606 GN=STN1 PE=1 SV=2 |
| sp\|O14657\|TOR1B_HUMAN | extr | Torsin-1B OS=Homo sapiens OX=9606 GN=TOR1B PE=1 SV=2 |
| sp\|O15118\|NPC1_HUMAN | plas | NPC intracellular cholesterol transporter 1 OS=Homo sapiens OX=9606 GN=NPC1 PE=1 SV=2 |
| sp\|B7ZAP0\|RBG10_HUMAN | cyto | Rab GTPase-activating protein 1-like, isoform 10 OS=Homo sapiens OX=9606 GN=RABGAP1L PE=1 SV=1 |
| sp\|Q9NX76\|CKLF6_HUMAN | plas | CKLF-like MARVEL transmembrane domain-containing protein 6 OS=Homo sapiens OX=9606 GN=CMTM6 PE=1 SV=1 |
| sp\|O95498\|VNN2_HUMAN | plas | Vascular non-inflammatory molecule 2 OS=Homo sapiens OX=9606 GN=VNN2 PE=1 SV=3 |
| sp\|P0DI81\|TPC2A_HUMAN | cyto | Trafficking protein particle complex subunit 2 OS=Homo sapiens OX=9606 GN=TRAPPC2 PE=1 SV=1 >sp\|P0DI82\|TPC2B_HUMAN Trafficking protein particle complex subunit 2B OS=Homo sapiens OX=9606 GN=TRAPPC2B PE=1 SV=1 |
| sp\|Q969Z3\|MARC2_HUMAN | mito | Mitochondrial amidoxime reducing component 2 OS=Homo sapiens OX=9606 GN=MARC2 PE=1 SV=1 |
| sp\|Q4ZHG4\|FNDC1_HUMAN | extr | Fibronectin type III domain-containing protein 1 OS=Homo sapiens OX=9606 GN=FNDC1 PE=2 SV=4 |
| sp\|Q92604\|LGAT1_HUMAN | plas | Acyl-CoA:lysophosphatidylglycerol acyltransferase 1 OS=Homo sapiens OX=9606 GN=LPGAT1 PE=1 SV=1 |
| sp\|Q6IC98\|GRAM4_HUMAN | plas | GRAM domain-containing protein 4 OS=Homo sapiens OX=9606 GN=GRAMD4 PE=1 SV=1 |
| sp\|Q9H446\|RWDD1_HUMAN | cyto | RWD domain-containing protein 1 OS=Homo sapiens OX=9606 GN=RWDD1 PE=1 SV=1 |
| sp\|Q5U5Q3\|MEX3C_HUMAN | nucl | RNA-binding E3 ubiquitin-protein ligase MEX3C OS=Homo sapiens OX=9606 GN=MEX3C PE=1 SV=3 |
| sp\|Q9NPH0\|PPA6_HUMAN | mito | Lysophosphatidic acid phosphatase type 6 OS=Homo sapiens OX=9606 GN=ACP6 PE=1 SV=3 |
| sp\|Q96G03\|PGM2_HUMAN | nucl | Phosphoglucomutase-2 OS=Homo sapiens OX=9606 GN=PGM2 PE=1 SV=4 |
| sp\|A0AVT1\|UBA6_HUMAN | nucl | Ubiquitin-like modifier-activating enzyme 6 OS=Homo sapiens OX=9606 GN=UBA6 PE=1 SV=1 |
| sp\|Q8IYT8\|ULK2_HUMAN | nucl | Serine/threonine-protein kinase ULK2 OS=Homo sapiens OX=9606 GN=ULK2 PE=1 SV=3 |
| sp\|O75694\|NU155_HUMAN | cyto | Nuclear pore complex protein Nup155 OS=Homo sapiens OX=9606 GN=NUP155 PE=1 SV=1 |
| sp\|O75643\|U520_HUMAN | cyto | U5 small nuclear ribonucleoprotein 200 kDa helicase OS=Homo sapiens OX=9606 GN=SNRNP200 PE=1 SV=2 |
| sp\|H7BZ55\|CRCC2_HUMAN | cyto_nucl | Putative ciliary rootlet coiled-coil protein 2 OS=Homo sapiens OX=9606 GN=CROCC2 PE=5 SV=3 |
| sp\|Q15811\|ITSN1_HUMAN | cyto_nucl | Intersectin-1 OS=Homo sapiens OX=9606 GN=ITSN1 PE=1 SV=3 |
| sp\|Q8TC44\|POC1B_HUMAN | nucl | POC1 centriolar protein homolog B OS=Homo sapiens OX=9606 GN=POC1B PE=1 SV=1 |
| sp\|Q7Z4V5\|HDGR2_HUMAN | nucl | Hepatoma-derived growth factor-related protein 2 OS=Homo sapiens OX=9606 GN=HDGFL2 PE=1 SV=1 |
| sp\|Q9UNZ5\|L10K_HUMAN | nucl | Leydig cell tumor 10 kDa protein homolog OS=Homo sapiens OX=9606 GN=C19orf53 PE=1 SV=1 |
| sp\|Q9Y4A5\|TRRAP_HUMAN | plas | Transformation/transcription domain-associated protein OS=Homo sapiens OX=9606 GN=TRRAP PE=1 SV=3 |
| sp\|Q99523\|SORT_HUMAN | plas | Sortilin OS=Homo sapiens OX=9606 GN=SORT1 PE=1 SV=3 |
| sp\|Q8IWC1\|MA7D3_HUMAN | nucl | MAP7 domain-containing protein 3 OS=Homo sapiens OX=9606 GN=MAP7D3 PE=1 SV=2 |
| sp\|P15088\|CBPA3_HUMAN | extr | Mast cell carboxypeptidase A OS=Homo sapiens OX=9606 GN=CPA3 PE=1 SV=2 |
| sp\|Q5VW32\|BROX_HUMAN | mito | BRO1 domain-containing protein BROX OS=Homo sapiens OX=9606 GN=BROX PE=1 SV=1 |
| sp\|O14684\|PTGES_HUMAN | extr | Prostaglandin E synthase OS=Homo sapiens OX=9606 GN=PTGES PE=1 SV=2 |
| sp\|O94855\|SC24D_HUMAN | cyto | Protein transport protein Sec24D OS=Homo sapiens OX=9606 GN=SEC24D PE=1 SV=2 |
| sp\|O75791\|GRAP2_HUMAN | cyto | GRB2-related adapter protein 2 OS=Homo sapiens OX=9606 GN=GRAP2 PE=1 SV=1 |
| sp\|Q9NR99\|MXRA5_HUMAN | extr | Matrix-remodeling-associated protein 5 OS=Homo sapiens OX=9606 GN=MXRA5 PE=1 SV=3 |
| sp\|Q15907\|RB11B_HUMAN | cysk | Ras-related protein Rab-11B OS=Homo sapiens OX=9606 GN=RAB11B PE=1 SV=4 |
| sp\|Q7L8C5\|SYT13_HUMAN | extr | Synaptotagmin-13 OS=Homo sapiens OX=9606 GN=SYT13 PE=1 SV=1 |
| sp\|Q15742\|NAB2_HUMAN | nucl | NGFI-A-binding protein 2 OS=Homo sapiens OX=9606 GN=NAB2 PE=1 SV=1 |
| sp\|P16671\|CD36_HUMAN | plas | Platelet glycoprotein 4 OS=Homo sapiens OX=9606 GN=CD36 PE=1 SV=2 |
| sp\|Q8N3V7\|SYNPO_HUMAN | nucl | Synaptopodin OS=Homo sapiens OX=9606 GN=SYNPO PE=1 SV=2 |
| sp\|Q9BVC6\|TM109_HUMAN | plas | Transmembrane protein 109 OS=Homo sapiens OX=9606 GN=TMEM109 PE=1 SV=1 |
| sp\|Q13111\|CAF1A_HUMAN | nucl | Chromatin assembly factor 1 subunit A OS=Homo sapiens OX=9606 GN=CHAF1A PE=1 SV=3 |
| sp\|Q9H201\|EPN3_HUMAN | nucl | Epsin-3 OS=Homo sapiens OX=9606 GN=EPN3 PE=2 SV=1 |
| sp\|P08246\|ELNE_HUMAN | extr | Neutrophil elastase OS=Homo sapiens OX=9606 GN=ELANE PE=1 SV=1 |
| sp\|P13716\|HEM2_HUMAN | mito | Delta-aminolevulinic acid dehydratase OS=Homo sapiens OX=9606 GN=ALAD PE=1 SV=1 |
| sp\|O14744\|ANM5_HUMAN | cyto | Protein arginine N-methyltransferase 5 OS=Homo sapiens OX=9606 GN=PRMT5 PE=1 SV=4 |
| sp\|Q14696\|MESD_HUMAN | extr | LRP chaperone MESD OS=Homo sapiens OX=9606 GN=MESD PE=1 SV=2 |
| sp\|P49759\|CLK1_HUMAN | nucl | Dual specificity protein kinase CLK1 OS=Homo sapiens OX=9606 GN=CLK1 PE=1 SV=2 |
| sp\|O95503\|CBX6_HUMAN | nucl | Chromobox protein homolog 6 OS=Homo sapiens OX=9606 GN=CBX6 PE=1 SV=1 |
| sp\|Q9H3Z4\|DNJC5_HUMAN | cyto | DnaJ homolog subfamily C member 5 OS=Homo sapiens OX=9606 GN=DNAJC5 PE=1 SV=1 |
| sp\|Q9H5U6\|ZCHC4_HUMAN | cyto_nucl | rRNA N6-adenosine-methyltransferase ZCCHC4 OS=Homo sapiens OX=9606 GN=ZCCHC4 PE=1 SV=3 |
| sp\|Q15758\|AAAT_HUMAN | plas | Neutral amino acid transporter B(0) OS=Homo sapiens OX=9606 GN=SLC1A5 PE=1 SV=2 |
| sp\|Q6PGP7\|TTC37_HUMAN | cyto | Tetratricopeptide repeat protein 37 OS=Homo sapiens OX=9606 GN=TTC37 PE=1 SV=1 |
| sp\|Q8N6Q3\|CD177_HUMAN | extr | CD177 antigen OS=Homo sapiens OX=9606 GN=CD177 PE=1 SV=2 |
| sp\|P21291\|CSRP1_HUMAN | nucl | Cysteine and glycine-rich protein 1 OS=Homo sapiens OX=9606 GN=CSRP1 PE=1 SV=3 |
| sp\|Q9H8J5\|MANS1_HUMAN | extr_plas | MANSC domain-containing protein 1 OS=Homo sapiens OX=9606 GN=MANSC1 PE=2 SV=1 |
| sp\|Q92544\|TM9S4_HUMAN | plas | Transmembrane 9 superfamily member 4 OS=Homo sapiens OX=9606 GN=TM9SF4 PE=1 SV=2 |
| sp\|Q9Y6B7\|AP4B1_HUMAN | cyto_nucl | AP-4 complex subunit beta-1 OS=Homo sapiens OX=9606 GN=AP4B1 PE=1 SV=2 |
| sp\|Q6VUC0\|AP2E_HUMAN | nucl | Transcription factor AP-2-epsilon OS=Homo sapiens OX=9606 GN=TFAP2E PE=2 SV=1 |
| sp\|P21810\|PGS1_HUMAN | mito | Biglycan OS=Homo sapiens OX=9606 GN=BGN PE=1 SV=2 |
| sp\|P12814\|ACTN1_HUMAN | cyto_nucl | Alpha-actinin-1 OS=Homo sapiens OX=9606 GN=ACTN1 PE=1 SV=2 |
| sp\|P10515\|ODP2_HUMAN | mito | Dihydrolipoyllysine-residue acetyltransferase component of pyruvate dehydrogenase complex, mitochondrial OS=Homo sapiens OX=9606 GN=DLAT PE=1 SV=3 |
| sp\|Q93073\|SBP2L_HUMAN | nucl | Selenocysteine insertion sequence-binding protein 2-like OS=Homo sapiens OX=9606 GN=SECISBP2L PE=1 SV=3 |
| sp\|P78527\|PRKDC_HUMAN | cyto | DNA-dependent protein kinase catalytic subunit OS=Homo sapiens OX=9606 GN=PRKDC PE=1 SV=3 |
| sp\|Q53GD3\|CTL4_HUMAN | plas | Choline transporter-like protein 4 OS=Homo sapiens OX=9606 GN=SLC44A4 PE=1 SV=2 |
| sp\|Q9UKA2\|FBXL4_HUMAN | mito | F-box/LRR-repeat protein 4 OS=Homo sapiens OX=9606 GN=FBXL4 PE=1 SV=2 |
| sp\|Q15526\|SURF1_HUMAN | mito | Surfeit locus protein 1 OS=Homo sapiens OX=9606 GN=SURF1 PE=1 SV=1 |
| sp\|Q9H9E3\|COG4_HUMAN | cyto | Conserved oligomeric Golgi complex subunit 4 OS=Homo sapiens OX=9606 GN=COG4 PE=1 SV=3 |
| sp\|P24158\|PRTN3_HUMAN | extr | Myeloblastin OS=Homo sapiens OX=9606 GN=PRTN3 PE=1 SV=3 |
| sp\|Q6NXT6\|TAPT1_HUMAN | plas | Transmembrane anterior posterior transformation protein 1 homolog OS=Homo sapiens OX=9606 GN=TAPT1 PE=1 SV=1 |
| sp\|P53680\|AP2S1_HUMAN | mito | AP-2 complex subunit sigma OS=Homo sapiens OX=9606 GN=AP2S1 PE=1 SV=2 |
| sp\|Q9H3K2\|GHITM_HUMAN | plas | Growth hormone-inducible transmembrane protein OS=Homo sapiens OX=9606 GN=GHITM PE=1 SV=2 |
| sp\|O60488\|ACSL4_HUMAN | E.R. | Long-chain-fatty-acid--CoA ligase 4 OS=Homo sapiens OX=9606 GN=ACSL4 PE=1 SV=2 |
| sp\|P50416\|CPT1A_HUMAN | cyto | Carnitine O-palmitoyltransferase 1, liver isoform OS=Homo sapiens OX=9606 GN=CPT1A PE=1 SV=2 |
| sp\|P29966\|MARCS_HUMAN | nucl | Myristoylated alanine-rich C-kinase substrate OS=Homo sapiens OX=9606 GN=MARCKS PE=1 SV=4 |
| sp\|P05155\|IC1_HUMAN | extr | Plasma protease C1 inhibitor OS=Homo sapiens OX=9606 GN=SERPING1 PE=1 SV=2 |
| sp\|Q9H7B4\|SMYD3_HUMAN | cyto | Histone-lysine N-methyltransferase SMYD3 OS=Homo sapiens OX=9606 GN=SMYD3 PE=1 SV=4 |
| sp\|P02655\|APOC2_HUMAN | extr | Apolipoprotein C-II OS=Homo sapiens OX=9606 GN=APOC2 PE=1 SV=1 |
| sp\|P09917\|LOX5_HUMAN | cyto | Arachidonate 5-lipoxygenase OS=Homo sapiens OX=9606 GN=ALOX5 PE=1 SV=2 |
| sp\|Q9BPX6\|MICU1_HUMAN | mito | Calcium uptake protein 1, mitochondrial OS=Homo sapiens OX=9606 GN=MICU1 PE=1 SV=1 |
| sp\|P55209\|NP1L1_HUMAN | cyto_nucl | Nucleosome assembly protein 1-like 1 OS=Homo sapiens OX=9606 GN=NAP1L1 PE=1 SV=1 |
| sp\|Q8N2K0\|ABD12_HUMAN | plas | Monoacylglycerol lipase ABHD12 OS=Homo sapiens OX=9606 GN=ABHD12 PE=1 SV=2 |
| sp\|Q9P1Y6\|PHRF1_HUMAN | nucl | PHD and RING finger domain-containing protein 1 OS=Homo sapiens OX=9606 GN=PHRF1 PE=1 SV=3 |
| sp\|P18827\|SDC1_HUMAN | extr | Syndecan-1 OS=Homo sapiens OX=9606 GN=SDC1 PE=1 SV=3 |
| sp\|P20936\|RASA1_HUMAN | cyto | Ras GTPase-activating protein 1 OS=Homo sapiens OX=9606 GN=RASA1 PE=1 SV=1 |
| sp\|O14683\|P5I11_HUMAN | plas | Tumor protein p53-inducible protein 11 OS=Homo sapiens OX=9606 GN=TP53I11 PE=1 SV=2 |
| sp\|Q16850\|CP51A_HUMAN | E.R. | Lanosterol 14-alpha demethylase OS=Homo sapiens OX=9606 GN=CYP51A1 PE=1 SV=3 |
| sp\|P00568\|KAD1_HUMAN | cyto | Adenylate kinase isoenzyme 1 OS=Homo sapiens OX=9606 GN=AK1 PE=1 SV=3 |
| sp\|Q9C0A1\|ZFHX2_HUMAN | nucl | Zinc finger homeobox protein 2 OS=Homo sapiens OX=9606 GN=ZFHX2 PE=1 SV=3 |
| sp\|Q8NH73\|OR4S2_HUMAN | plas | Olfactory receptor 4S2 OS=Homo sapiens OX=9606 GN=OR4S2 PE=3 SV=2 |
| sp\|P55854\|SUMO3_HUMAN | cyto | Small ubiquitin-related modifier 3 OS=Homo sapiens OX=9606 GN=SUMO3 PE=1 SV=2 |
| sp\|Q6ZS72\|PEAK3_HUMAN | cyto | Uncharacterized protein PEAK3 OS=Homo sapiens OX=9606 GN=PEAK3 PE=2 SV=1 |
| sp\|Q8IVM0\|CCD50_HUMAN | nucl | Coiled-coil domain-containing protein 50 OS=Homo sapiens OX=9606 GN=CCDC50 PE=1 SV=1 |
| sp\|Q14011\|CIRBP_HUMAN | nucl | Cold-inducible RNA-binding protein OS=Homo sapiens OX=9606 GN=CIRBP PE=1 SV=1 |
| sp\|Q9BR39\|JPH2_HUMAN | pero | Junctophilin-2 OS=Homo sapiens OX=9606 GN=JPH2 PE=1 SV=2 |
| sp\|Q9Y512\|SAM50_HUMAN | cyto | Sorting and assembly machinery component 50 homolog OS=Homo sapiens OX=9606 GN=SAMM50 PE=1 SV=3 |
| sp\|Q96PU8\|QKI_HUMAN | cyto | Protein quaking OS=Homo sapiens OX=9606 GN=QKI PE=1 SV=1 |
| sp\|P51572\|BAP31_HUMAN | E.R. | B-cell receptor-associated protein 31 OS=Homo sapiens OX=9606 GN=BCAP31 PE=1 SV=3 |
| sp\|Q9Y5T5\|UBP16_HUMAN | nucl | Ubiquitin carboxyl-terminal hydrolase 16 OS=Homo sapiens OX=9606 GN=USP16 PE=1 SV=1 |
| sp\|Q7Z7F7\|RM55_HUMAN | mito | 39S ribosomal protein L55, mitochondrial OS=Homo sapiens OX=9606 GN=MRPL55 PE=1 SV=1 |
| sp\|Q8TCT7\|SPP2B_HUMAN | plas | Signal peptide peptidase-like 2B OS=Homo sapiens OX=9606 GN=SPPL2B PE=1 SV=2 |
| sp\|P98179\|RBM3_HUMAN | nucl | RNA-binding protein 3 OS=Homo sapiens OX=9606 GN=RBM3 PE=1 SV=1 |
| sp\|Q9ULD0\|OGDHL_HUMAN | mito | 2-oxoglutarate dehydrogenase-like, mitochondrial OS=Homo sapiens OX=9606 GN=OGDHL PE=1 SV=3 |
| sp\|Q68DK7\|MSL1_HUMAN | nucl | Male-specific lethal 1 homolog OS=Homo sapiens OX=9606 GN=MSL1 PE=1 SV=3 |
| sp\|Q9H6K4\|OPA3_HUMAN | mito | Optic atrophy 3 protein OS=Homo sapiens OX=9606 GN=OPA3 PE=1 SV=1 |
| sp\|Q96BJ3\|AIDA_HUMAN | nucl | Axin interactor, dorsalization-associated protein OS=Homo sapiens OX=9606 GN=AIDA PE=1 SV=1 |
| sp\|Q92930\|RAB8B_HUMAN | cyto | Ras-related protein Rab-8B OS=Homo sapiens OX=9606 GN=RAB8B PE=1 SV=2 |
| sp\|Q9H8L6\|MMRN2_HUMAN | extr | Multimerin-2 OS=Homo sapiens OX=9606 GN=MMRN2 PE=1 SV=2 |
| sp\|Q53EU6\|GPAT3_HUMAN | plas | Glycerol-3-phosphate acyltransferase 3 OS=Homo sapiens OX=9606 GN=GPAT3 PE=1 SV=2 |
| sp\|Q9H6F2\|TM38A_HUMAN | plas | Trimeric intracellular cation channel type A OS=Homo sapiens OX=9606 GN=TMEM38A PE=1 SV=1 |
| sp\|Q13075\|BIRC1_HUMAN | cyto | Baculoviral IAP repeat-containing protein 1 OS=Homo sapiens OX=9606 GN=NAIP PE=1 SV=3 |
| sp\|P06737\|PYGL_HUMAN | cyto | Glycogen phosphorylase, liver form OS=Homo sapiens OX=9606 GN=PYGL PE=1 SV=4 |
| sp\|Q8N201\|INT1_HUMAN | plas | Integrator complex subunit 1 OS=Homo sapiens OX=9606 GN=INTS1 PE=1 SV=2 |
| sp\|P48651\|PTSS1_HUMAN | plas | Phosphatidylserine synthase 1 OS=Homo sapiens OX=9606 GN=PTDSS1 PE=1 SV=1 |
| sp\|P24557\|THAS_HUMAN | plas | Thromboxane-A synthase OS=Homo sapiens OX=9606 GN=TBXAS1 PE=1 SV=3 |
| sp\|Q495T6\|MMEL1_HUMAN | cyto | Membrane metallo-endopeptidase-like 1 OS=Homo sapiens OX=9606 GN=MMEL1 PE=2 SV=2 |
| sp\|Q14653\|IRF3_HUMAN | cyto | Interferon regulatory factor 3 OS=Homo sapiens OX=9606 GN=IRF3 PE=1 SV=1 |
| sp\|Q2PPJ7\|RGPA2_HUMAN | plas | Ral GTPase-activating protein subunit alpha-2 OS=Homo sapiens OX=9606 GN=RALGAPA2 PE=1 SV=2 |
| sp\|O75781\|PALM_HUMAN | nucl | Paralemmin-1 OS=Homo sapiens OX=9606 GN=PALM PE=1 SV=2 |
| sp\|Q2TAA5\|ALG11_HUMAN | plas | GDP-Man:Man(3)GlcNAc(2)-PP-Dol alpha-1,2-mannosyltransferase OS=Homo sapiens OX=9606 GN=ALG11 PE=1 SV=2 |
| sp\|Q7L2H7\|EIF3M_HUMAN | cyto | Eukaryotic translation initiation factor 3 subunit M OS=Homo sapiens OX=9606 GN=EIF3M PE=1 SV=1 |
| sp\|Q9Y6A1\|POMT1_HUMAN | plas | Protein O-mannosyl-transferase 1 OS=Homo sapiens OX=9606 GN=POMT1 PE=1 SV=3 |
| sp\|Q12788\|TBL3_HUMAN | cyto | Transducin beta-like protein 3 OS=Homo sapiens OX=9606 GN=TBL3 PE=1 SV=2 |
| sp\|Q9BUN8\|DERL1_HUMAN | plas | Derlin-1 OS=Homo sapiens OX=9606 GN=DERL1 PE=1 SV=1 |
| sp\|Q9Y2C3\|B3GT5_HUMAN | extr | Beta-1,3-galactosyltransferase 5 OS=Homo sapiens OX=9606 GN=B3GALT5 PE=2 SV=1 |
| sp\|Q9H254\|SPTN4_HUMAN | nucl | Spectrin beta chain, non-erythrocytic 4 OS=Homo sapiens OX=9606 GN=SPTBN4 PE=1 SV=2 |
| sp\|O75886\|STAM2_HUMAN | nucl | Signal transducing adapter molecule 2 OS=Homo sapiens OX=9606 GN=STAM2 PE=1 SV=1 |
| sp\|Q9Y5W7\|SNX14_HUMAN | plas | Sorting nexin-14 OS=Homo sapiens OX=9606 GN=SNX14 PE=1 SV=3 |
| sp\|Q9NRF8\|PYRG2_HUMAN | pero | CTP synthase 2 OS=Homo sapiens OX=9606 GN=CTPS2 PE=1 SV=1 |
| sp\|Q9Y520\|PRC2C_HUMAN | nucl | Protein PRRC2C OS=Homo sapiens OX=9606 GN=PRRC2C PE=1 SV=4 |
| sp\|Q86TI2\|DPP9_HUMAN | cyto | Dipeptidyl peptidase 9 OS=Homo sapiens OX=9606 GN=DPP9 PE=1 SV=3 |
| sp\|Q8NFG4\|FLCN_HUMAN | nucl | Folliculin OS=Homo sapiens OX=9606 GN=FLCN PE=1 SV=1 |
| sp\|Q5VW36\|FOCAD_HUMAN | plas | Focadhesin OS=Homo sapiens OX=9606 GN=FOCAD PE=1 SV=1 |
| sp\|P46781\|RS9_HUMAN | cyto | 40S ribosomal protein S9 OS=Homo sapiens OX=9606 GN=RPS9 PE=1 SV=3 |
| sp\|Q86WV1\|SKAP1_HUMAN | cyto_nucl | Src kinase-associated phosphoprotein 1 OS=Homo sapiens OX=9606 GN=SKAP1 PE=1 SV=3 |
| sp\|P13674\|P4HA1_HUMAN | E.R. | Prolyl 4-hydroxylase subunit alpha-1 OS=Homo sapiens OX=9606 GN=P4HA1 PE=1 SV=2 |
| sp\|Q7Z7M9\|GALT5_HUMAN | extr | Polypeptide N-acetylgalactosaminyltransferase 5 OS=Homo sapiens OX=9606 GN=GALNT5 PE=1 SV=1 |
| sp\|Q96CW1\|AP2M1_HUMAN | cyto | AP-2 complex subunit mu OS=Homo sapiens OX=9606 GN=AP2M1 PE=1 SV=2 |
| sp\|Q7Z4G4\|TRM11_HUMAN | cyto | tRNA (guanine(10)-N2)-methyltransferase homolog OS=Homo sapiens OX=9606 GN=TRMT11 PE=1 SV=1 |
| sp\|P48509\|CD151_HUMAN | plas | CD151 antigen OS=Homo sapiens OX=9606 GN=CD151 PE=1 SV=3 |
| sp\|Q96GC9\|VMP1_HUMAN | plas | Vacuole membrane protein 1 OS=Homo sapiens OX=9606 GN=VMP1 PE=1 SV=1 |
| sp\|Q14767\|LTBP2_HUMAN | mito | Latent-transforming growth factor beta-binding protein 2 OS=Homo sapiens OX=9606 GN=LTBP2 PE=1 SV=3 |
