# Supplementary material for: Apolipoprotein C‐II induces EMT to promote gastric cancer peritoneal metastasis via PI3K/AKT/mTOR pathway
Source: Clin Transl Med. 2021 Aug 9;11(8):e522. doi: 10.1002/ctm2.522 (PMC8351524; doi:10.1002/ctm2.522)
Supplement: Supplementary file 26 — Table S11. GO_REGULATION_OF_LIPID_METABOLIC_PROCESS. [file CTM2-11-e522-s024.docx]

**Table S11. GO_REGULATION_OF_LIPID_METABOLIC_PROCESS.**

| WASHC1 |  | SIN3B |  | KIT |  | REST |
| --- | --- | --- | --- | --- | --- | --- |
| MIR548P |  | CTDNEP1 |  | KPNB1 |  | FAM3A |
| NR1H3 |  | SIRT4 |  | TNFAIP8L3 | | ELOVL5 |
| CDK4 |  | SIRT1 |  | LDLR |  | RORA |
| ZMPSTE24 | | MLYCD |  | LEP |  | RORC |
| STUB1 |  | PIK3R5 |  | FADS1 |  | RXRA |
| RACK1 |  | SEC14L2 |  | LSS |  | SC5D |
| VAV3 |  | FPR2 |  | LYN |  | SCD |
| PIBF1 |  | RAB38 |  | MIR138-1 |  | CEACAM1 |
| CARM1 |  | MTOR |  | MIR138-2 |  | CCL19 |
| NCOA2 |  | FSHB |  | MIR182 |  | CCL21 |
| NPC2 |  | NR5A1 |  | MIR183 |  | NOD2 |
| SORBS1 |  | ETFBKMT |  | MIR185 |  | LMF1 |
| ERLIN1 |  | CNEP1R1 |  | MIR30C1 |  | ARV1 |
| PMVK |  | SIN3A |  | MIR30C2 |  | P2RY12 |
| CGA |  | LDLRAP1 |  | MIR33A |  | BCL11B |
| PPARGC1A | | ZBTB20 |  | MIR96 |  | BMP2 |
| GLIPR1 |  | TIAM2 |  | ME1 |  | BMP5 |
| ERLIN2 |  | FGF21 |  | ASAH1 |  | BMP6 |
| DDX20 |  | GFI1 |  | PLIN5 |  | SNAI2 |
| TREX1 |  | GHSR |  | MVD |  | MTMR9 |
| CHRM5 |  | GIP |  | MVK |  | SMARCD3 |
| PIK3IP1 |  | ANKRD1 |  | ATP1A1 |  | SMPD2 |
| LACTB |  | DKKL1 |  | NFE2L1 |  | SNAI1 |
| C1QTNF2 |  | DKK3 |  | NFKB1 |  | SNCA |
| CISH |  | TNFRSF21 |  | NFYA |  | SOD1 |
| APOA5 |  | GNB3 |  | NFYB |  | SP1 |
| AGAP2 |  | GPLD1 |  | NFYC |  | SQLE |
| CLCN2 |  | GPER1 |  | NPAS2 |  | SRC |
| SERPINA3 |  | GPS2 |  | G0S2 |  | BRCA1 |
| PLIN2 |  | WDR91 |  | ACOX1 |  | SREBF1 |
| CCR7 |  | ORMDL2 |  | GAL |  | SREBF2 |
| WDR81 |  | DNAJC15 |  | MLXIPL |  | STAR |
| ACER1 |  | GSTZ1 |  | ADIPOR1 |  | STAT5B |
| CNR1 |  | GRHL1 |  | ABHD5 |  | SULT2A1 |
| FITM2 |  | NRBF2 |  | ANGPTL4 |  | TAZ |
| AADAC |  | ANXA1 |  | INSIG2 |  | TBL1X |
| ADM |  | SOCS7 |  | GOLM1 |  | TEK |
| STARD4 |  | PIK3R4 |  | MBTPS2 |  | TGFB1 |
| CPT1A |  | STOML2 |  | PRKAG2 |  | TSPO |
| CPT2 |  | ACACA |  | PDGFA |  | THRSP |
| CREB1 |  | HMGCR |  | PDGFB |  | TM7SF2 |
| CREBBP |  | HMGCS1 |  | PDGFRA |  | TNF |
| CREBL2 |  | HMGCS2 |  | PDGFRB |  | TNFRSF1A |
| SAMD8 |  | HNF4A |  | LSR |  | C3 |
| SERPINA12 | | ACACB |  | PDK1 |  | TWIST1 |
| PIK3R6 |  | ACADL |  | PDK2 |  | TXNRD1 |
| SIK1 |  | APOA1 |  | PDK3 |  | NR1H2 |
| ERFE |  | HTR2A |  | PDK4 |  | VAV2 |
| DAB2IP |  | HTR2B |  | ABCB4 |  | VDR |
| CYP1A1 |  | HTR2C |  | PIK3CG |  | SF1 |
| CYP4A11 |  | APOA2 |  | PIK3R1 |  | PSAPL1 |
| CYP7A1 |  | APOA4 |  | PIK3R2 |  | ZP3 |
| TTC39B |  | APOB |  | TM6SF2 |  | ELOVL6 |
| CYP27B1 |  | APOBEC1 |  | WNT4 |  | ADIPOR2 |
| CYP51A1 |  | ZFP69 |  | POR |  | TBL1XR1 |
| DGKQ |  | ID2 |  | UGT1A8 |  | LPCAT1 |
| DHCR7 |  | ACADM |  | UGT1A9 |  | PANK2 |
| DRD3 |  | MALRD1 |  | PPARA |  | NR4A3 |
| AGT |  | APOC1 |  | UGT1A1 |  | CHD9 |
| AGTR1 |  | IDH1 |  | PPARD |  | OPA3 |
| ABCA1 |  | IDI1 |  | PPARG |  | CAPN2 |
| EEF1A2 |  | APOC2 |  | MED1 |  | CCDC3 |
| EGR1 |  | APOC3 |  | PDP1 |  | LONP2 |
| EPHA8 |  | HSD17B13 | | LAMTOR1 |  | PLA2G6 |
| EPHX2 |  | IFNG |  | PDPR |  | SLA2 |
| AKT1 |  | APOD |  | AVP |  | NSMAF |
| AKT2 |  | APOE |  | APPL2 |  | DGAT2 |
| ALAS1 |  | IGFBP7 |  | AVPR1A |  | PIK3R3 |
| F2 |  | CCN1 |  | PPP2R5A |  | DGKZ |
| ABCD1 |  | IL1B |  | FBXW7 |  | SLC45A3 |
| FABP1 |  | INS |  | SMPD3 |  | HELZ2 |
| FABP3 |  | INSIG1 |  | PRKAA1 |  | CAV1 |
| FABP5 |  | IRS1 |  | AMBRA1 |  | PLPP1 |
| ACSL1 |  | EIF6 |  | PRKAA2 |  | AKR1C3 |
| ACSL3 |  | ACADVL |  | PRKAB2 |  | NCOA1 |
| PTK2B |  | ARF1 |  | PRKCD |  | SOCS1 |
| FASN |  | ENHO |  | PRKD1 |  | IRS2 |
| TYSND1 |  | SLC27A1 |  | ANGPTL8 |  | MBTPS1 |
| ADGRF5 |  | ORMDL3 |  | PROX1 |  | EDF1 |
| FDFT1 |  | GGPS1 |  | PSAP |  | MTMR1 |
| FDPS |  | CD36 |  | PNPLA2 |  | PEX11A |
| FGF1 |  | SCARB1 |  | PPP4R3B |  | SOCS2 |
| FGF2 |  | H2AFY |  | ABHD6 |  | HDAC3 |
| ABCD2 |  | NR1D1 |  | PTGS2 |  | CCKBR |
| FGFR3 |  | NCOR1 |  | PTK2 |  | MTMR3 |
| FGFR4 |  | NCOR2 |  | DISP3 |  | MTMR2 |
| FGR |  | ABCG1 |  | PDP2 |  | SOCS3 |
| FHL2 |  | SOCS5 |  | GPAM |  | RGN |
| ATG14 |  | TGS1 |  | TRIB3 |  | MTMR4 |
| SCAP |  | RUBCN |  | BBS4 |  | CD19 |
| WDTC1 |  | CD81 |  | MID1IP1 |  | SOCS6 |
| NCOA6 |  | LPGAT1 |  | RAN |  | KLF4 |
| RGL1 |  | FGF19 |  | RB1 |  | ADIPOQ |
| FLT1 |  | NR1H4 |  | RBL1 |  | ORMDL1 |
| FLT3 |  | NR1D2 |  | RBL2 |  |  |
