# Supplementary material for: Apolipoprotein C‐II induces EMT to promote gastric cancer peritoneal metastasis via PI3K/AKT/mTOR pathway
Source: Clin Transl Med. 2021 Aug 9;11(8):e522. doi: 10.1002/ctm2.522 (PMC8351524; doi:10.1002/ctm2.522)
Supplement: Supplementary file 27 — Table S12. Associations between serum APOC2 expression and clinical pathological characteristics in patients with GC [file CTM2-11-e522-s026.docx]

**Table S12. Associations between serum APOC2 expression and clinical pathological characteristics in patients with GC**

| **Variables** | **Cases**  **(Total=66)** | | **Concentration (mg/dL)** | |  | ***P* value** |
| --- | --- | --- | --- | --- | --- | --- |
| Age at diagnosis |  | |  | |  |  |
| <60 | 30(45.5) | | 3.25±1.359 | |  | 0.742 |
| ≥60 | 36(54.5) | | 3.35±1.234 | |  |  |
| Gender |  | |  | |  |  |
| Male | 45(68.2) | | 3.32±1.277 | |  | 0.903 |
| Female | 21(31.8) | | 3.28±1.328 | |  |  |
| Tumor location |  | |  | |  |  |
| Cardia | 20(30.3) | | 3.29±1.561 | |  | 0.900 |
| Body | 17(25.8) | | 3.20±1.320 | |  |  |
| Antrum | 29(43.9) | | 3.38±1.076 | |  |  |
| Histologic type |  | |  | |  |  |
| Intestinal | 28(42.4) | | 2.98±1.130 | |  | 0.086 |
| Mixed | 17(25.8) | | 3.24±1.203 | |  |  |
| Diffuse | 21(31.8) | | 3.79±1.438 | |  |  |
| Tumor size (cm) |  | |  | |  |  |
| <4 | 29(43.9) | | 3.28±1.211 | |  | 0.905 |
| ≥4 | 37(56.1) | | 3.32±1.353 | |  |  |
| AJCC stage |  | |  | |  |  |
| I+II | 24(36.4) | | 2.85±1.236 | |  | 0.030^*^ |
| III+IV | 42(63.6) | | 3.56±1.252 | |  |  |
| T stage |  | |  | |  |  |
| T1+T2 | 19(28.8) | | 2.79±1.027 | |  | 0.038^*^ |
| T3+T4 | 47(71.2) | | 3.51±1.327 | |  |  |
| N stage |  | |  | |  |  |
| N0+N1 | 29(43.9) | | 2.91±1.276 | |  | 0.025^*^ |
| N2+N3 | 37(56.1) | | 3.62±1.217 | |  |  |
| M stage |  | |  | |  |  |
| M0 | 59(89.4) | 3.18±1.220 | |  | | 0.020^*^ |
| M1 | 7(10.6) | 4.36±1.415 | |  |  |  |
| Nervous invasion |  |  | |  | |  |
| Positive | 39(59.1) | 3.51±1.345 | |  | | 0.117 |
| Negative | 27(40.9) | 3.01±1.148 | |  |  |  |
| Venous invasion |  |  | |  | |  |
| Positive | 27(40.9) | 3.41±1.065 | |  | | 0.585 |
| Negative | 39(59.1) | 3.23±1.424 | |  |  |  |

***p<0.05**
